# Wacker Oxidation of Trisubstituted Alkenes: Pd(II)‐Catalyzed Oxidative Ring Expansion of Exocyclic *α,β*‐Unsaturated Carbonyl to 2‐Fluoro‐1,3‐Dicarbonyl Compounds

**DOI:** 10.1002/anie.202511478

**Published:** 2025-08-11

**Authors:** Vincent Goëlo, Qian Wang, Jieping Zhu

**Affiliations:** ^1^ Laboratory of Synthesis and Natural Products (LSPN) Institute of Chemical Sciences and Engineering Ecole Polytechnique Fédérale de Lausanne EPFL‐SB‐ISIC‐LSPN BCH 5304 Lausanne 1015 Switzerland

**Keywords:** Dyotropic rearrangement, Fluorination, High valent palladium, Ring expansion, Wacker oxidation

## Abstract

The Wacker oxidation is a powerful synthetic method widely employed for the transformation of monosubstituted alkenes into methyl ketones. Its substrate scope has progressively expanded to include internal disubstituted and, more recently, *gem*‐disubstituted alkenes. Herein, we report the first examples of Wacker‐type oxidation of trisubstituted alkenes under Pd(II)/Pd(IV) catalysis. In the presence of Selectfluor (2.3 equiv) and a catalytic amount of Pd(MeCN)_4_(BF_4_)_2_ (10 mol%), exocyclic trisubstituted *α*,*β*‐unsaturated carbonyl compounds undergo a fluorinative one‐carbon ring expansion to deliver 2‐fluoro‐1,3‐dicarbonyl compounds in good yields. The reaction displays broad functional group tolerance, including alkyl halide, aryl halide, alkyl tosylate, hydroxyl, carboxylic acid, ester, secondary amide, ketone, cyano, and *N*‐phthalimide. While a semi‐pinacol rearrangement of the Pd(IV) intermediate is a plausible mechanistic pathway given the excellent nucleofugality of the Pd(IV) atom, preliminary studies suggest that a 1,2‐alkyl/Pd(IV) dyotropic rearrangement is operative in product formation.

## Graphical abstract

First examples of Pd(II)‐catalyzed Wacker oxidation of trisubstituted alkenes, coupled with electrophilic fluorination, enable a one‐carbon ring expansion of exocyclic α,β‐unsaturated carbonyls to 2‐fluoro‐2‐acyl‐cycloalkanones.

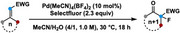

The oxidation of ethylene to acetaldehyde under Pd(II)/Cu(II)/O_2_ catalysis is an industrial process developed by the chemical company Wacker Chemie in 1959.^[^ ^1^, ^2^, ^3^ ^]^ The reaction proceeds through a sequence of regioselective hydroxypalladation, *β*‐hydride elimination of the resulting Pd(II) complex, and subsequent tautomerization of the enol intermediate (Scheme 1a).^[^ ^4^, ^5^ ^]^ This Pd(II)‐catalyzed oxidative transformation has since evolved into a reliable method for the conversion of monosubstituted alkenes to methyl ketones and has become a widely utilized tool in the synthesis of natural products and pharmaceuticals.^[^ ^6^, ^7^, ^8^ ^]^ The reaction has also been extended to disubstituted internal alkenes. However, structurally nonbiased, unsymmetrical alkenes generally afford a mixture of two regioisomeric ketones (Scheme 1b‐1). To achieve high regioselectivity, the presence of a coordinating group,^[^ ^9^, ^10^, ^11^, ^12^, ^13^, ^14^, ^15^, ^16^ ^]^ or an electron‐polarizing substituent^[^ ^17^ ^]^ is typically required. For instance, Tsuji and co‐workers developed conditions that enable the selective conversion of methyl (*E*)‐hex‐2‐enoate to methyl‐3‐oxohexanoate (Scheme 1b‐2).^[^ ^17^ ^]^

**Scheme 1:**
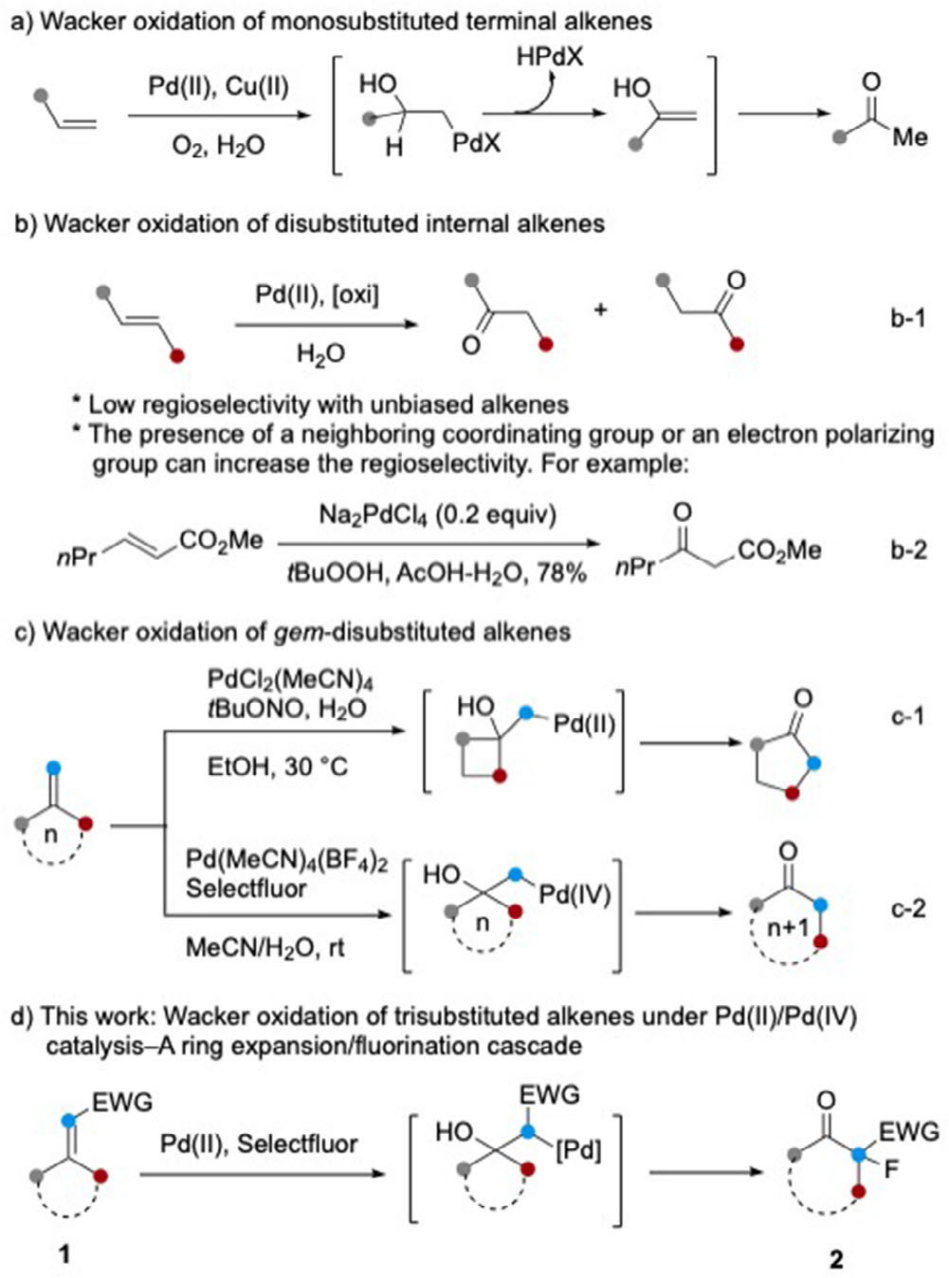
The Wacker oxidation: From monosubstituted alkenes to internal disubstituted, *gem*‐disubstituted, and trisubstituted olefins.

**Scheme 2:**
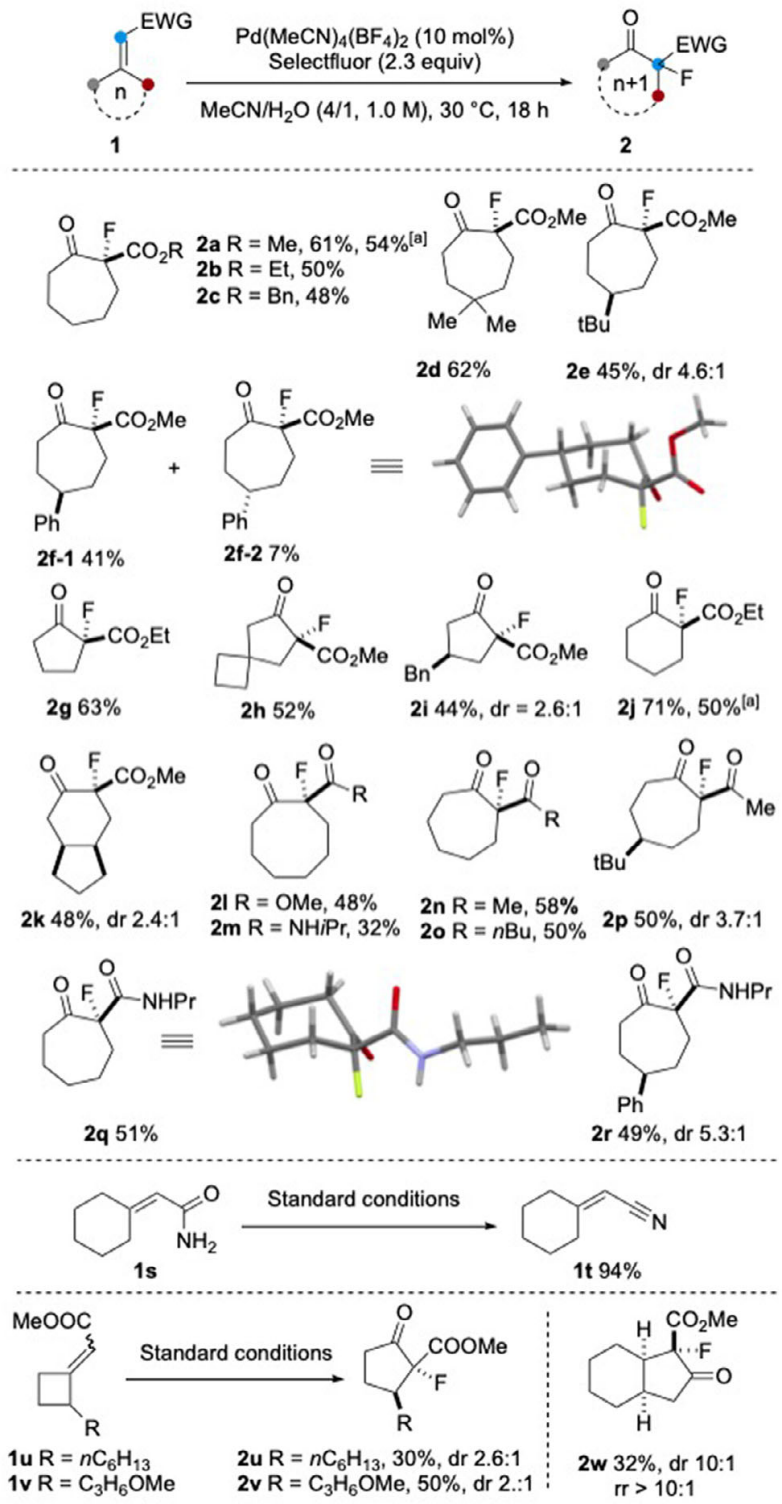
Reaction was conducted at 0.2 mmol scale. ^a)^ Reaction was conducted at 2.0 mmol scale. All reactions were performed in a single run.

In 1977, Boontanonda and Grigg reported that methylenecyclobutanes were converted to cyclopentanone derivatives under the standard Wacker conditions (PdCl_2_, CuCl_2_, O_2_, H_2_O).^[^ ^18^ ^]^ In 2022, Wahl and co‐workers found that the use of *tert*‐butyl nitrite was crucial for achieving this transformation.^[^ ^19^ ^]^ Under their optimized conditions [PdCl_2_(MeCN)_2_‐*t*BuONO‐EtOH‐H_2_O, 30 °C], the oxidative ring expansion of diversely substituted methylenecyclobutanes proceeded smoothly to afford cyclopentanone derivatives in excellent yields (Scheme 1c‐1). A strain‐releasing semi‐pinacol rearrangement of the Pd(II) intermediate, formed via hydroxypalladation of the exocyclic double bond, was proposed to explain the reaction outcome. Concurrently, our group reported that a Pd(II)/Pd(IV) catalytic system is applicable to both acyclic *gem*‐disubstituted alkenes and methylenecycloalkanes, enabling the synthesis of one‐carbon chain‐extended ketones and ring‐expanded cycloalkanones, respectively (Scheme 1c‐2).^[^ ^20^ ^]^ It is worth noting that highly toxic and hazardous reagents, including lead tetraacetate,^[^ ^21^ ^]^ thallium nitrate,^[^ ^22^, ^23^ ^]^ and cyanogen azide (a primary explosive),^[^ ^24^ ^]^ have been employed as stoichiometric oxidants for the ring expansion of methylenecycloalkanes. Furthermore, hydroxy(tosyloxy)iodobenzene (Koser's reagent) has been used for one‐carbon chain extension of *α*‐substituted styrenes.^[^ ^25^ ^]^

Trisubstituted alkenes have not been explored as substrates for the Wacker oxidation. In their studies on ring expansion of methylenecycloalkanes using cyanogen azide,^[^ ^24^ ^]^ McMurry and coworkers showed that methyl 2‐cyclohexylideneacetate failed to undergo the desired transformation. As part of our ongoing project focusing on the development of domino processes incorporating a Pd(IV) based‐dyotropic rearrangement,^[^ ^20^, ^26^, ^27^, ^28^, ^29^, ^30^, ^31^, ^32^, ^33^, ^34^, ^35^ ^]^ we report herein that exocyclic *α*,*β*‐unsaturated carbonyl compounds undergo oxidative one‐carbon ring enlargement, under Pd(II)/Pd(IV) catalytic conditions, to afford *α*‐fluorinated *β*‐dicarbonyl compounds (Scheme 1d). Although one‐carbon ring expansion of cycloalkanones to *β*‐keto esters can be realized through Brønsted acid‐catalyzed reaction with methyl diazoacetate,^[^ ^36^ ^]^ an additional step is required to convert these products into the corresponding fluorinated *β*‐dicarbonyl compounds.^[^ ^37^, ^38^ ^]^ Notably, methyl 2‐diazo‐2‐fluoroacetate, while conceptually attractive for the synthesis of *α*‐fluoro‐*β*‐keto esters, has never been synthesized. Computational studies suggest that this compound is inherently unstable.^[^ ^39^, ^40^ ^]^

Methyl 2‐cyclohexylideneacetate (**1a**) was selected as a model substrate. Submitting it to our previously reported conditions for the oxidative rearrangement of 1,1‐disubstituted alkenes [Pd(MeCN)_4_(BF_4_)_2_ (10 mol%), Selectfluor (1.2 equiv), MeCN/H_2_O (v/v = 4:1, *c* 0.2 M), RT, 2 h],^[^ ^20^ ^]^ the reaction proceeded sluggishly. After 48 hours, a mixture of **1a**, **2a,** and **3a** was obtained in a ratio of 2:3:1. Monitoring the reaction progress suggested that compounds **2a** and **3a** were generated concurrently and a control experiment indicated that **3a** underwent fluorination with Selectfluor in the presence of Pd(II) catalyst to efficiently generate **2a**. We therefore focused our efforts on improving the yield of **2a**, which required the use of at least two equivalents of Selectfluor. The optimal conditions consisted of performing the reaction of **1a** with Selectfluor (2.3 equiv) in a mixed solvent [MeCN/H_2_O (v/v = 4:1, *c* 1.0 M)] in the presence of a catalytic amount of Pd(MeCN)_4_(BF_4_)_2_ (10 mol%) at 30 °C for 8 h. Under these conditions, a full conversion of **1a** was achieved, affording **2a** in 61% isolated yield, along with 5% of diol **4a**. (Table 1, entry 1). Key observations from the optimization studies are as follows: a) Alternative oxidants such as NFSI and Oxone were ineffective (entries 2 and 3); b) Increasing the MeCN content relative to H_2_O accelerated the reaction, but also promoted side reactions, resulting in a lower yield of **2a** (entry 4); c) Reaction concentration played a critical role. At 0.2 M, conversion remained low. At 0.5 M, full conversion was reached after 16 h, but **2a** was obtained in only 35% yield with significant decomposition (entry 5); d) Addition of base was detrimental suggesting that the acidic conditions are necessary for the reaction to proceed efficiently (entry 6); e) Reducing the Pd(II) loading to 5 mol% afforded **2a** in only 36% yield, along with partial recovery of starting material **1a** (entry 7); f) A varied amount of 1,2‐diol **4a** was formed under certain reaction conditions. We note that Pd(II)‐catalyzed dihydroxylation and diacetoxylation of electron‐rich alkenes, involving a Pd(IV) intermediate, have been previously reported.^[^ ^41^, ^42^, ^43^, ^44^ ^]^

**Table 1:** Survey of reaction conditions.^a)^

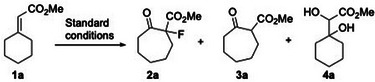		
Entry	Deviation from standard conditions	Results
1	–	61% of **2a**, 5% of **4a**
2	NFSI instead of Selectfluor	∼ 90% **1a**, no products
3	Oxone instead of Selectfluor	∼ 90% **1a**, trace of **3a**
4	MeCN/H_2_O (v/v 8:1), 2 h	43% of **2a**
5	*c* 0.5 M, 16 h	35% of **2a**
6	Add base (KOAc, K_2_CO_3_, K_3_PO_4_)	∼ 90% **1a**, no products
7	5 mol% of Pd(MeCN)_4_(BF_4_)_2_	36% of **2a**+**1a**

Selectfluor = 1‐chloromethyl‐4‐fluoro‐1,4‐diazoniabicyclo[2.2.2]octane bis(tetrafluoroborate); NFSI = *N*‐fluorobenzenesulfonimide.

^a)^ Standard conditions: **1a** (0.2 mmol), Pd(MeCN)_4_(BF_4_)_2_ (10 mol%), Selectfluor (2.3 equiv), MeCN/H_2_O (4/1, *c* 1.0 M), 30 °C, 8 h.

With the optimized conditions in hand, we next explored the substrate scope by varying the ring size and the nature of the electron‐withdrawing group (Scheme 2). In addition to the methyl ester, the ethyl and benzyl esters of 2‐cyclohexylideneacetate were successfully converted to ring‐expanded products **2b** and **2c**, respectively. Substitution at the C_4_ position was well tolerated, as demonstrated by the formation of products **2d**‐**2f**. In the case of C_4_ monosubstituted substrates, two diastereoisomers were formed with moderate diastereoselectivities (**2e**, **2f**). The relative stereochemistry of the two diastereomers were determined by NMR analysis and that of the minor isomer **2f**‐**2** was further confirmed by X‐ray crystallography.^[^ ^45^ ^]^ The reaction also proceeded smoothly with alkyl cyclobutylideneacetates, cyclopentylideneacetates and alkyl cycloheptylideneacetates, affording the corresponding 2‐fluoro‐2‐alkoxycarbonylcycloalkan‐1‐ones (**2g**‐**2m**). The seven‐ to eight‐membered ring expansion observed in **2l** and **2m** is particularly noteworthy, given the well‐documented synthetic challenge associated with accessing cyclooctanes relative to cycloheptanes. We note that a significant amount of diol (18%, see also Scheme 6b) was isolated when the reaction of **1j** was performed on a 2.0 mmol scale. However, no further optimization was undertaken at this stage to suppress diol formation in the scale‐up experiment.

Furthermore, exocyclic *α*,*β*‐unsaturated amides (**1m**, **1q**, **1r**) and ketones (**1n**‐**1p**) underwent fluorinative ring expansion to deliver products **2m**‐**2r**, respectively. However, when primary amide **1s** was subjected to the reaction conditions, it underwent dehydration to afford *α*,*β*‐unsaturated nitrile **1t** in 94% yield. ^[^ ^46^, ^47^, ^48^, ^49^, ^50^ ^]^

Substrates **1u‐1w**, bearing an alkyl substituent at the allylic position, were converted to **2u‐2w**, respectively, as mixtures of two diastereomers. Excellent diastereoselectivity was observed in the case of **2w**. Notably, in all cases, only a single regioisomer resulting from the migration of the more substituted carbon was isolated.

The functional group tolerance of the reaction was next evaluated. As shown in Scheme 3, a wide range of functionalities, including a free hydroxyl group (**2x**), *p*‐toluenesulfonate (**2y**), halogens (**2z**, **2aa**, **2ae**), a cyano group (**2ab**), phthalimide (**2ac**), amide (**2ad**), ester (**2ae**, **2ag**), free carboxylic acid (**2af**), and ketone (**2ah**), were all tolerated under the reaction conditions. The presence of these functional groups offers useful handles for further derivatizations of the resulting cycloheptanones. A heterocycle, exemplified by methyl 2‐(tetrahydro‐4*H*‐pyran‐4‐ylidene)acetate, was also successfully transformed into a functionalized 5‐oxooxepane (**2ai**) in 58% yield. Finally, the *α*,*β*‐unsaturated ester **1aj**, derived from 5*α*‐androstane‐3,17‐dione, underwent ring expansion to afford two regioisomeric products **2aj** and **2aj’** in a 1:1 ratio. Although no regioselectivity was observed during the migration process, the diastereoselectivities of the two products were good (7:1 and 5:1, respectively).

**Scheme 3:**
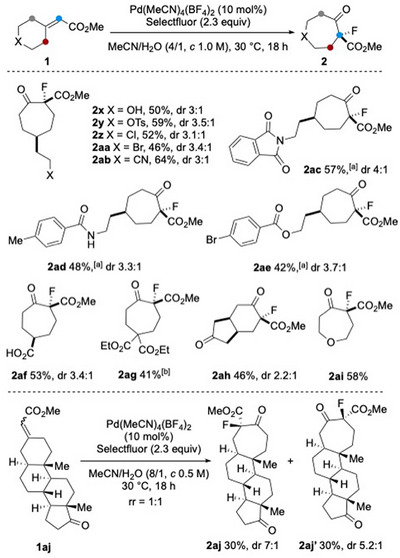
Functional group tolerance. ^a)^ A mixed solvent MeCN/H_2_O (v/v = 8:1, c 0.5 M) was used to increase the solubility of the starting materials; ^b)^ 48 h. rr regioisomeric ratio. All reactions were performed in a single run.

Finally, no reaction was observed with tetrasubstituted alkenes **1ak** and **1al** (Figure 1). Similarly, acyclic alkene **1am** suffered from low conversion along with degradation, while an electron‐rich trisubstituted alkene (**1an**) afforded a complex mixture under standard conditions.

**Figure 1:**
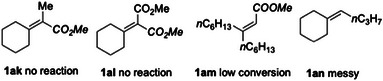
Failed substrates.

To gain insight into the reaction mechanism, several control experiments were carried out. Subjecting substrate **1a** to the reaction conditions with one equivalent of the Pd salt without Selectfluor failed to produce the rearranged product **3a** (Scheme 4a). Similarly, omitting the Pd salt under otherwise standard conditions resulted in no formation of compound **2a** (Scheme 4b). These observations suggested that the alkyl‐Pd(II) intermediate generated after hydroxypalladation of alkene is not competent to undergo the 1,2‐rearrangement in the absence of Selectfluor.

**Scheme 4:**
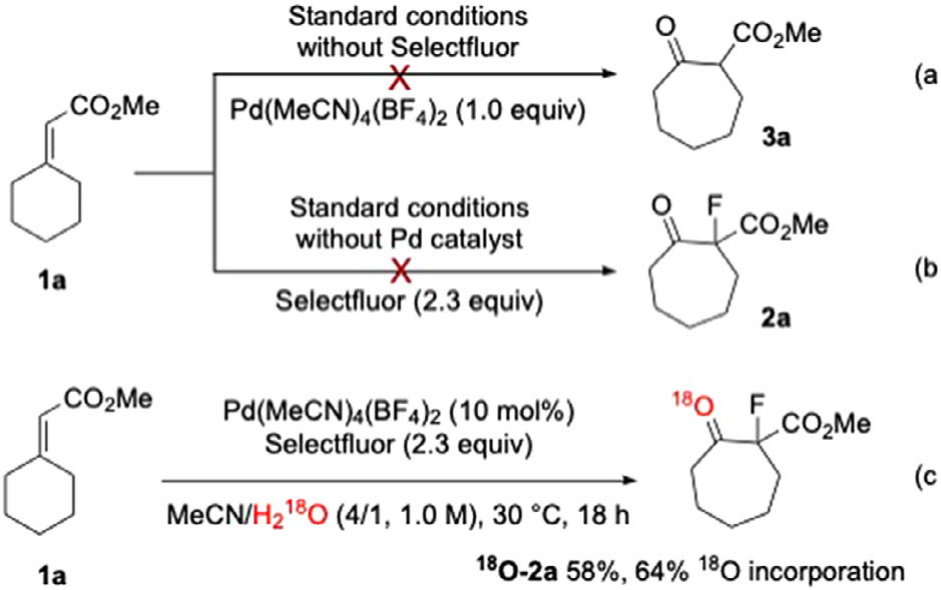
Control experiments.

To determine the origin of the oxygen atom in the product, an isotope labelling experiment was performed using ^18^O‐labelled H_2_O (97% ^18^O) as co‐solvent of MeCN. Under these conditions, **1a** was converted to **^18^O‐2a** in 58% yield with 64% ^18^O incorporation (Scheme 4c). The reduced ^18^O content could be attributed to the hydration of ketone during aqueous work‐up and column chromatographic purification. The result indicates that water serves as the oxygen source in this transformation.

Building on the results of aforementioned control experiments, plausible reaction pathways are depicted in Scheme 5. Hydroxypalladation of the double bond in **1** would afford intermediate **A‐1**, which might be in equilibrium with its palladium enolate form through the *ƞ* ^3^‐complex **A‐2**. Oxidation of the Pd(II) species with Selectfluor would generate Pd(IV) complex **B**, from which two pathways could be envisaged. A semi‐pinacol rearrangement of **B** leveraging the excellent nucleofugality of the Pd(IV) centre^[^ ^51^, ^52^ ^]^ would furnish the ring‐expanded 2‐methoxycarbonylcycloalkan‐1‐one **3**. Alternatively, a 1,2‐C(sp^3^)/Pd(IV) dyotropic rearrangement would generate one‐carbon homologated cycloalkane **C**, which would then be converted to *β*‐keto ester **3** through direct elimination of Pd(II) species or via *α*‐fluorohydrine intermediate. Finally, electrophilic fluorination of **3** by Selectfluor would yield the observed product **2**.^[^ ^37^, ^38^ ^]^

**Scheme 5:**
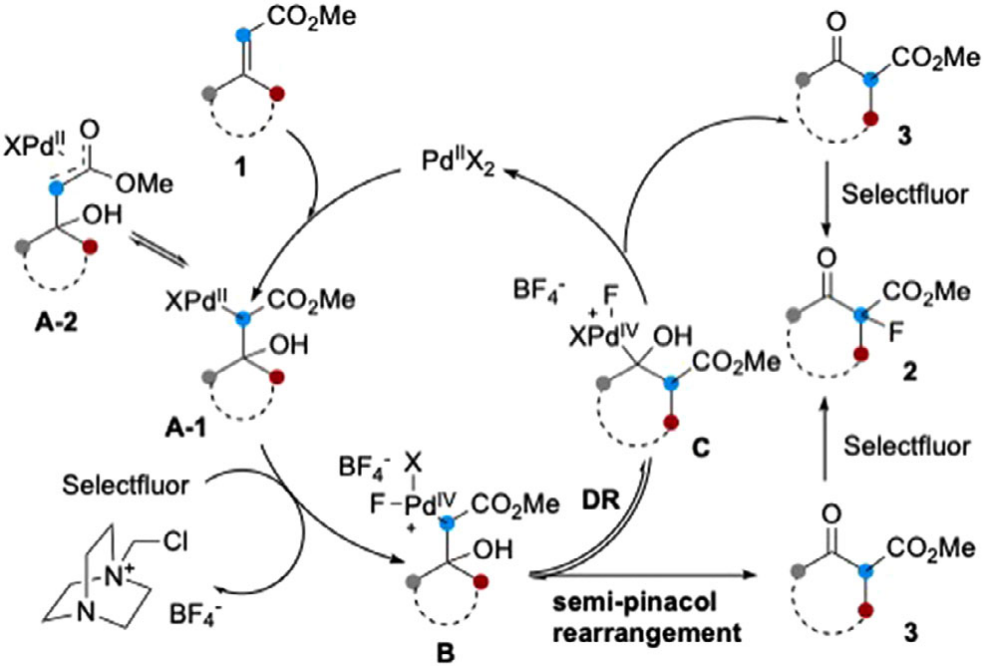
Possible reaction pathways. DR, dyotropic rearrangement.

While no isolable intermediate was expected in the semi‐pinacol rearrangement of **B** to **3**, several intermediates are, in principle, observable during the conversion of **B** to **3** via dyotropic rearrangement. To explore this possibility, the reaction conditions were slightly modified to facilitate the isolation of a rearranged intermediate. Gratifyingly, treatment of **1a** in acetonitrile in the presence of benzoic acid (2.0 equiv) under otherwise identical conditions afforded the ring‐expanded *O*‐benzoyl *α*‐fluorohydrine **5** in 9% yield. The low yield was attributed to low conversion and partial decomposition of the product during purification by preparative TLC (Scheme 6a). Transesterification of **5** (NaOMe, MeOH) afforded, via presumably the fluorohydrine **6**, **3a** in 91% yield.

**Scheme 6:**
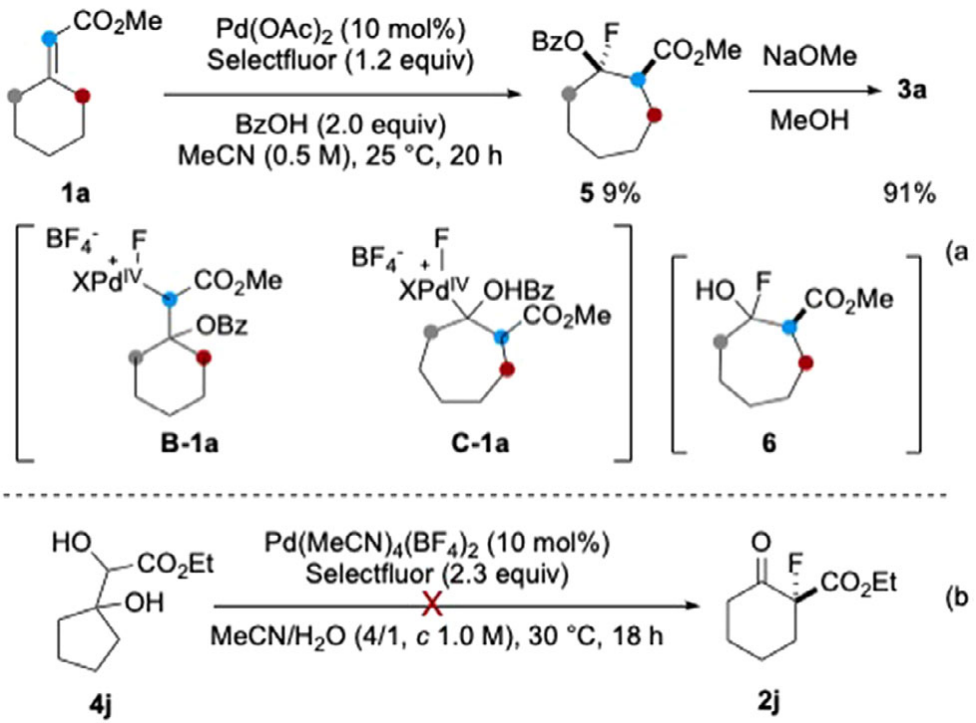
Mechanistic studies. a) Isolation of the dyotropic rearrangement intermediate **5**; b) Diol **4j** is not the precursor of rearranged product **2j**.

The formation of **5** can be rationalized by a domino sequence involving benzoyloxypalladation of the double bond followed by oxidation of the C(sp^3^)‐Pd(II) to C(sp^3^)‐Pd(IV) species (**B‐1a**), its subsequent dyotropic rearrangement to **C‐1a** and finally C‐F bond‐forming reductive elimination. While the result supports the feasibility of the dyotropic rearrangement pathway, it does not exclude the possibility of the semi‐pinacol rearrangement manifold.

Remarkably, the present dyotropic rearrangement involves migration of the Pd(IV) atom from the *α*‐ to the *β*‐position of the carbonyl group, a process that appears thermodynamically unfavourable. However, assuming the rearrangement is reversible and the activation energy for the subsequent transformation of intermediate **C**, including direct elimination of Pd(II) species or C─F bond‐forming reductive elimination, is lower than that of the competing pathways from intermediate **B** (such as the semi‐pinacol rearrangement or S_N_2 displacement of the Pd(IV) centre), the equilibrium would be driven towards intermediate **C** and ultimately pull the reaction toward the desired product via the dyotropic rearrangement pathway.

The 1,2‐diol **4j** was isolated as a byproduct in the scale‐up synthesis of compound **2j**. When subjected to the standard conditions, **4j** was largely recovered along with some decomposition (Scheme 6b). This experiment suggested that the diol **4j** is not an intermediate en route to the formation of the observed product **2j**.

In summary, we have developed the first examples of Wacker‐type oxidation of trisubstituted alkenes under Pd(II)/Pd(IV) catalysis. The Pd(II)‐catalyzed reaction of exocyclic trisubstituted *α,β*‐unsaturated carbonyl compounds with Selectfluor efficiently afforded ring expanded 2‐fluoro‐2‐acyl‐cycloalkan‐1‐ones. One C─C, one C─F, and one C═O bonds were formed in this transformation. The reaction conditions exhibited broad functional group tolerance, accommodating alkyl halide, aryl halide, alkyl tosylate, hydroxyl, carboxylic acid, ester, secondary amide, ketone, cyano, and *N‐*phthalimide.(Supporting information)

## Supporting Information

The authors have cited additional references within the Supporting Information.^[^ ^53^, ^54^, ^55^, ^56^, ^57^, ^58^, ^59^, ^60^, ^61^, ^62^, ^63^, ^64^, ^65^, ^66^, ^67^, ^68^, ^69^, ^70^ ^]^

## Conflict of Interests

The authors declare no conflict of interest.

## Supporting information



Supporting Information

Supporting Information

## Data Availability

The data that support the findings of this study are available in the supplementary material of this article.
