# Supplementary material for: Wacker Oxidation of Trisubstituted Alkenes: Pd(II)‐Catalyzed Oxidative Ring Expansion of Exocyclic α,β‐Unsaturated Carbonyl to 2‐Fluoro‐1,3‐Dicarbonyl Compounds
Source: Angew Chem Int Ed Engl. 2025 Aug 11;64(40):e202511478. doi: 10.1002/anie.202511478 (PMC12462759; doi:10.1002/anie.202511478)
Supplement: Supplementary file 1 — Supporting Information [file ANIE-64-e202511478-s001.pdf]

Supporting information

Wacker Oxidation of Trisubstituted Alkenes: Pd(II)-Catalyzed Oxidative Ring Expansion of Exocyclic *α,β*-Unsaturated Carbonyl to 2-Fluoro-1,3-Dicarbonyl Compounds

Vincent Goëlo, Qian Wang and Jieping Zhu*

Laboratory of Synthesis and Natural Products (LSPN), Institute of Chemical Sciences and Engineering, Ecole Polytechnique Fédérale de Lausanne, EPFL-SB-ISIC-LSPN, BCH5304, 1015 Lausanne

*Correspondence to: jieping.zhu@epfl.ch

Contents

1) General information S2

2) Experimental procedures and characterization data S3

A) General procedures S3

B) Starting material preparation and characterization data S5

C) Dyotropic rearrangement: oxidative ring expansion to *α*-fluorinated *β*-dicarbonyl products S40

D) Starting materials of failed examples S82

E) References S87

3) Copies of NMR Spectra S88

4) X-Ray crystallographic data S303

1) General information

Reagents and solvents were purchased from commercial sources and preserved under argon. More sensitive compounds were stored in a desiccator or in the glovebox if required. Reagents were used as received without further purification unless otherwise noted. All reactions were performed under nitrogen (or argon) and stirring unless otherwise noted. When needed, glassware was dried overnight in an oven (150 °C). Solvents indicated as dry were either purchased as such, distilled prior to use, or dried by a passage through a column of anhydrous alumina or copper using a Puresolv MD 5 from Innovative Technology Inc., based on the Grubbs' design. Flash column chromatography was performed using Silicycle SiliaFlash® P60 230-400 mesh. Reactions were monitored using Merck Kieselgel 60F254 aluminum. TLC's were revealed by UV fluorescence (254 nm) then with either $KMnO_4$ or phosphomolybdic acid. NMR spectra were recorded on AV2 400 MHz, AV2 600 MHz or AV2 800 MHz Brüker spectrometers at room temperature. ^{1}H frequency is at 400.13 MHz, ^{13}C frequency is at 100.62 MHz. Chemical shifts (δ) were reported in parts per million (ppm) relative to residual solvent peaks rounded to the nearest 0.01 for proton and 0.1 for carbon (ref: $CDCl_3$ [^{1}H: 7.26, ^{13}C: 77.16 ppm].). Coupling constants (*J*) were reported in Hz to the nearest 0.1 Hz. Peak multiplicity was indicated as follows: s (singlet), d (doublet), t (triplet), q (quartet), p (quintet), m (multiplet) and br (broad). Attribution of peaks was done using the multiplicities and integrals of the peaks. COSY, HSQC, HMBC and NOESY experiments were used when needed to confirm the attribution. IR spectra were recorded in a Jasco FT/IR-4X spectrometer outfitted with a PIKE technology MIRacleTM ATR accessory as neat films compressed onto a Zinc Selenide window. The spectra are reported in cm^{-1}. Abbreviations used are: w (weak), m (medium), s (strong) and br (broad). The accurate masses were measured by the mass spectrometry service of the EPFL by ESI-TOF using a QTOF Ultima from Waters. Melting points were determined using a Stuart SMP30. Specific optical rotations $[\alpha]_D$ were measured with a Jasco P-2000 polarimeter (589 nm). Enantiomeric excesses were determined with a 1260 Infinity II SFC System from Agilent using chiral stationary phase columns by comparing the samples with the corresponding racemic samples, column and elution details specified in each entry.

List of Abbreviations:

TLC – thin-layer chromatography; FCC – flash column chromatography.

2) Experimental procedures and characterization data

A) General procedures

General procedure A1: preparation of the trisubstituted *α,β*-unsaturated carbonyl starting materials:

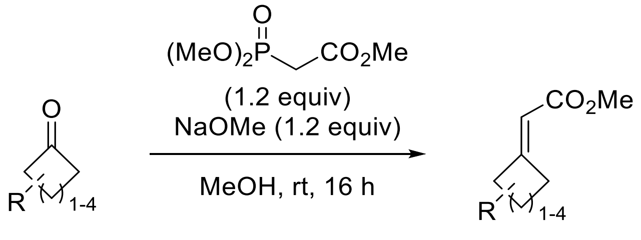

1

Trialkoxyphosphonoacetate (1.2 equiv) was slowly added to a solution of NaOMe (5.4 M in MeOH, 1.2 equiv) diluted in dry MeOH [1.0 M] at room temperature and the mixture was stirred for 45 minutes. Ketone derivative (1.0 equiv) was then slowly added (by syringe if liquid, neat or diluted with a small volume of dry MeOH if needed, portionwise if solid) and the reaction mixture was stirred for 16 h. The mixture was then poured into a saturated NH_4Cl solution and extracted three times with EtOAc. The combined organic layers were washed with brine, dry over Na_2SO_4, filtered and the volatiles were removed under reduced pressure. The crude product was purified by FCC (pentane/Et_2O or hexane/EtOAc) to give the corresponding *α,β*-unsaturated carbonyl product.

General procedure A2: preparation of the trisubstituted *α,β*-unsaturated carbonyl starting materials that tend to isomerize with procedure A1:

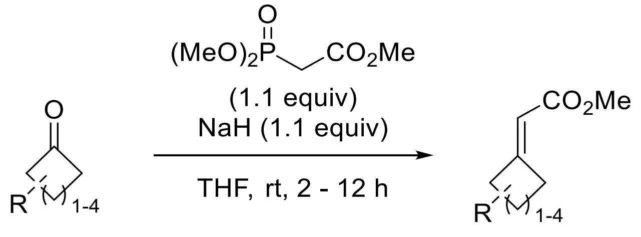

1

Trialkoxyphosphonoacetate (1.1 equiv) was slowly added to a solution of NaH (60% wt in mineral oil, 1.1 equiv) in dry THF [1.0 M] at room temperature and the mixture was stirred for

45 minutes. Ketone derivative (1.0 equiv) was diluted in dry THF [1.0 M] and slowly added to the reaction mixture. The mixture was stirred at room temperature for 2 – 12 h, then poured into saturated NH_4Cl solution and extracted three times with EtOAc. The combined organic layers were washed with brine, dry over Na_2SO_4, filtered and the volatiles were removed under reduced pressure. The crude product was purified by FCC (hexane/Et_2O or hexane/EtOAc) to give the *α,β*-unsaturated carbonyl product **1**.

General procedure B: oxidative ring expansion rearrangement of the trisubstituted *α,β*-unsaturated carbonyl compounds:

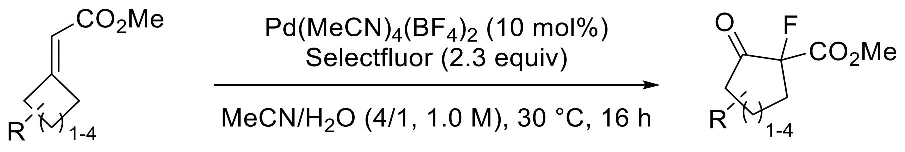

1 **2**

In a test tube was weighed *α,β*-unsaturated carbonyl compound **1** (0.2 mmol, 1.0 equiv), Selectfluor (163 mg, 0.46 mmol, 2.3 equiv) and $Pd(MeCN)_4(BF_4)_2$ (9.0 mg, 20 µmol, 10 mol%). $MeCN/H_2O$ (4/1 v/v, 200 µL, 1.0 M) was added, the tube was capped and the yellow/orange slurry was stirred at 30 °C for 16 h (up to 48 h for less reactive/ less soluble substrates). The resulting light-yellow solution was diluted with EtOAc (2.0 mL), filtered over a pad of silica gel, washed with EtOAc. The volatiles were removed under reduced pressure and the crude mixture was purified by FCC (hexane/EtOAc or pentane/Et_2O) to give the corresponding fluorinated *β*-dicarbonyl product **2**.

Variation of conditions: for less soluble compounds in $MeCN/H_2O$ (4/1 v/v, 200 µL, 1.0 M), the reactions were performed using $MeCN/H_2O$ (8/1 v/v, 400 µL, 0.5 M) at 30 °C.

B) Starting material preparation and characterization data

methyl 2-cyclohexylideneacetate (1a)

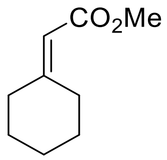

1a

Compound **1a** (2.84 g, 18.4 mmol, 92%) was prepared according to general procedure A1.

The analytical data were in agreement with those reported in the literature.[1]

Colorless oil.

Rf = 0.45 (1/9 Et_2O/Hexane).

^{1}H NMR (400 MHz, $CDCl_3$) δ 5.60 (p, *J* = 1.2 Hz, 1H), 3.67 (s, 3H), 2.85 – 2.79 (m, 2H), 2.21-2.17 (m, 2H), 1.69 – 1.55 (m, 6H).

^{13}C NMR (101 MHz, $CDCl_3$) δ 167.4, 164.1, 112.7, 50.9, 38.1, 30.0, 28.8, 28.0, 26.4.

HRMS (ESI/QTOF) m/z: $[M + H]^+$ Calcd for $C_9H_{15}O_2^+$ 155.1067; Found 155.1069.

IR (ν_{max}, cm^{-1}) 2932 (w), 2855 (w), 1716 (m), 1647 (m), 1433 (w), 1380 (w), 1271 (w), 1238 (w), 1206 (s), 1155 (s), 1129 (m), 1029 (m), 1022 (m), 931 (w), 851 (m), 755 (w).

ethyl 2-cyclohexylideneacetate (1b)

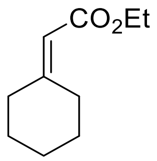

1b

Compound **1b** (327 mg, 1.94 mmol, 97%) was prepared according to general procedure A2.

The analytical data were in agreement with those reported in the literature.[2]

Colorless oil.

Rf = 0.50 (1/10 Et_2O/Hexane).

^{1}H NMR (400 MHz, $CDCl_3$) δ 5.59 (p, *J* = 1.1 Hz, 1H), 4.13 (q, *J* = 7.1 Hz, 2H), 2.85 – 2.79 (m, 2H), 2.21 – 2.16 (m, 2H), 1.69 – 1.55 (m, 6H), 1.26 (t, *J* = 7.1 Hz, 3H).

^{13}C NMR (101 MHz, $CDCl_3$) δ 167.0, 163.7, 113.2, 59.6, 38.1, 30.0, 28.7, 27.9, 26.4, 14.5.

HRMS (Sicrit plasma/LTQ-Orbitrap) m/z: $[M + H]^+$ Calcd for $C_{10}H_{17}O_2^+$ 169.1223; Found 169.1222.

IR (ν_{max}, cm^{-1}) 2980 (w), 2930 (w), 2856 (w), 1712 (m), 1647 (m), 1446 (w), 1379 (w), 1308 (w), 1270 (w), 1237 (w), 1205 (m), 1153 (s), 1128 (m), 1103 (w), 1038 (m), 994 (w), 866 (w), 851 (w).

benzyl 2-cyclohexylideneacetate (1c)

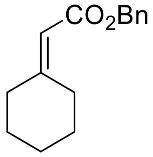

1c

A solution of cyclohexanone (207 µL, 2.0 mmol, 1.0 equiv) and benzyl (triphenylphosphoranylidene)acetate (902 mg, 2.2 mmol, 1.1 equiv) in dry toluene (6.0 mL, 0.33 M) was refluxed in a sealed tube for 48 h before removing volatiles under reduced pressure. The crude product was purified by FCC (1/15 EtOAc/hexane) to give compound **1c** (281 mg, 1.22 mmol, 61%) as colorless oil.

The analytical data were in agreement with those reported in the literature.[3]

Colorless oil.

Rf = 0.56 (1/12 EtOAc/Hexane).

^{1}H NMR (400 MHz, $CDCl_3$) δ 7.41 – 7.28 (m, 5H), 5.67 (s, 1H), 5.14 (s, 2H), 2.85 (t, *J* = 5.8 Hz, 2H), 2.20 (t, *J* = 5.9 Hz, 2H), 1.71 – 1.55 (m, 6H).

^{13}C NMR (101 MHz, $CDCl_3$) δ 166.7, 164.6, 136.6, 128.6, 128.3, 128.1, 112.8, 65.5, 38.2, 30.1, 28.8, 28.0, 26.4.

methyl 2-(4,4-dimethylcyclohexylidene)acetate (1d)

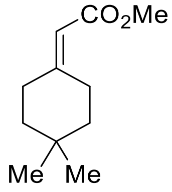

1d

Compound **1d** (160 mg, 0.88 mmol, 88%) was prepared according to general procedure A1.

Colorless oil.

Rf = 0.65 (1/11 EtOAc/Hexane).

^{1}H NMR (400 MHz, $CDCl_3$) δ 5.61 (t, *J* = 1.3 Hz, 1H), 3.67 (s, 3H), 2.84 (ddd, *J* = 7.8, 4.9, 1.2 Hz, 2H), 2.21 (ddd, *J* = 7.8, 5.0, 1.2 Hz, 2H), 1.45-1.40 (m, 4H), 0.97 (s, 6H).

^{13}C NMR (101 MHz, $CDCl_3$) δ 167.4, 164.1, 112.7, 50.9, 40.9, 40.2, 34.0, 30.4, 28.1, 25.8.

HRMS (nanochip-ESI/LTQ-Orbitrap) m/z: $[M + H]^+$ Calcd for $C_{11}H_{19}O_2^+$ 183.1380; Found 183.1388.

IR (ν_{max}, cm^{-1}) 2949 (w), 2915 (w), 2865 (w), 2849 (w), 1716 (s), 1649 (m), 1458 (w), 1433 (m), 1388 (m), 1376 (w), 1308 (w), 1268 (w), 1218 (m), 1162 (s), 1141 (s), 1032 (m), 999 (w), 964 (w), 920 (w), 860 (m), 789 (w), 732 (w), 684 (w).

methyl 2-(4-(tert-butyl)cyclohexylidene)acetate (1e)

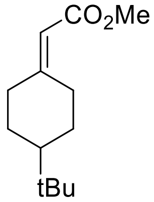

1e

Compound **1e** (2.1 g, 9.9 mmol, 99%) was prepared according to general procedure A1.

The analytical data were in agreement with those reported in the literature.[4]

Colorless crystals to colorless oil (**Mp** close to rt).

Rf = 0.45 (1/20 EtOAc/Hexane).

^{1}**H NMR** (400 MHz, $CDCl_3$) δ 5.59 (t, *J* = 1.8 Hz, 1H), 3.90 – 3.82 (m, 1H), 3.67 (s, 3H), 2.31 (dq, *J* = 13.3, 2.9 Hz, 1H), 2.21 – 2.10 (m, 1H), 1.97-1.90 (m, 2H), 1.87 – 1.77 (m, 1H), 1.26 (tt, *J* = 11.8, 2.9 Hz, 1H), 1.22 – 1.05 (m, 2H), 0.85 (s, 9H).

^{13}**C NMR** (101 MHz, $CDCl_3$) δ 167.4, 164.1, 112.4, 50.9, 47.9, 38.0, 32.6, 29.7, 29.4, 28.6, 27.7.

HRMS (nanochip-ESI/LTQ-Orbitrap) m/z: $[M + H]^+$ Calcd for $C_{13}H_{23}O_2^+$ 211.1693; Found 211.1702.

IR (ν_{max}, cm^{-1}) 2947 (w), 2866 (w), 2843 (w), 1716 (m), 1651 (m), 1469 (w), 1446 (w), 1433 (w), 1382 (w), 1365 (w), 1317 (w), 1269 (w), 1247 (m), 1212 (w), 1184 (m), 1163 (s), 1143 (s), 1092 (w), 1028 (m), 937 (w), 900 (w), 861 (m), 765 (w), 728 (w), 662 (w).

methyl 2-(4-phenylcyclohexylidene)acetate (1f)

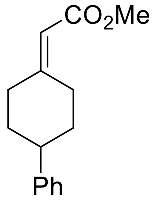

1f

Compound **1f** (216.5 mg, 0.94 mmol, 94%) was prepared according to general procedure A1.

The analytical data were in agreement with those reported in the literature.[5]

White crystals.

Rf = 0.50 (1/20 EtOAc/Hexane).

^{1}**H NMR** (400 MHz, $CDCl_3$) δ 7.33 – 7.27 (m, 2H), 7.23 – 7.16 (m, 3H), 5.69 (apparent t, *J* = 1.7 Hz, 1H), 4.01 – 3.93 (m, 1H), 3.71 (s, 3H), 2.79 (tt, *J* = 12.2, 3.5 Hz, 1H), 2.45 – 2.31 (m, 2H), 2.12 – 1.99 (m, 3H), 1.65 (qd, *J* = 12.6, 4.4 Hz, 2H).

^{13}**C NMR** (101 MHz, $CDCl_3$) δ 167.3, 162.4, 146.1, 128.6, 126.9, 126.4, 113.5, 51.1, 44.2, 37.8, 35.7, 34.9, 29.6.

HRMS (Sicrit plasma/LTQ-Orbitrap) m/z: $[M + H]^+$ Calcd for $C_{15}H_{19}O_2^+$ 231.1380; Found 231.1379.

IR (ν_{max}, cm^{-1}) 3027 (w), 2992 (w), 2941 (w), 2921 (w), 2854 (w), 1737 (m), 1712 (s), 1647 (m), 1490 (w), 1433 (m), 1377 (w), 1310 (w), 1271 (m), 1245 (m), 1172 (s), 1141 (s), 1088 (m), 1053 (m), 1025 (s), 967 (w), 936 (w), 901 (w), 861 (m), 809 (w), 762 (s), 720 (m), 703 (s).

Mp = 64 – 66 °C.

ethyl 2-cyclobutylideneacetate (1g)

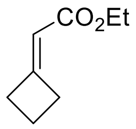

1g

Compound **1g** (280 mg, 2.0 mmol, 41%) was prepared according to general procedure A2.

The analytical data were in agreement with those reported in the literature.[6]

Colorless oil.

Rf = 0.45 (1/9 EtOAc/Hexane).

^{1}H NMR (400 MHz, $CDCl_3$) δ 5.57 (p, *J* = 2.3 Hz, 1H), 4.13 (q, *J* = 7.1 Hz, 2H), 3.16 – 3.08 (m, 2H), 2.86 – 2.78 (m, 2H), 2.08 (p, *J* = 7.9 Hz, 2H), 1.25 (t, *J* = 7.1 Hz, 3H).

^{13}C NMR (101 MHz, $CDCl_3$) δ 167.7, 166.7, 112.5, 59.7, 33.9, 32.5, 17.8, 14.5.

HRMS (Sicrit plasma/LTQ-Orbitrap) m/z: $[M + H]^+$ Calcd for $C_8H_{13}O_2^+$ 141.0910; Found 141.0910.

IR (ν_{max}, cm^{-1}) 2980 (w), 2958 (w), 2921 (w), 1712 (s), 1673 (m), 1367 (m), 1334 (m), 1264 (m), 1243 (m), 1221 (m), 1184 (s), 1086 (s), 1038 (m), 851 (m).

methyl 2-(spiro[3.3]heptan-2-ylidene)acetate (1h)

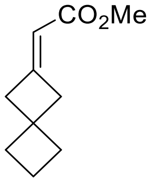

1h

Compound **1h** (261.4 mg, 1.57 mmol, 79%) was prepared according to general procedure A1.

Colorless oil.

Rf = 0.50 (1/9 Et_2O/Hexane).

^{1}H NMR (400 MHz, $CDCl_3$) δ 5.61 (p, *J* = 2.3 Hz, 1H), 3.67 (s, 3H), 3.10 – 3.06 (m, 2H), 2.80 – 2.76 (m, 2H), 2.08 – 2.00 (m, 4H), 1.88 – 1.78 (m, 2H).

^{13}C NMR (101 MHz, $CDCl_3$) δ 167.1, 163.6, 112.8, 51.0, 46.3, 45.0, 40.0, 34.5, 16.4.

HRMS (Sicrit plasma/LTQ-Orbitrap) m/z: $[M + H]^+$ Calcd for $C_{10}H_{15}O_2^+$ 167.1067; Found 167.1063.

IR (ν_{max}, cm^{-1}) 2949 (w), 2849 (w), 1717 (s), 1677 (m), 1434 (m), 1397 (w), 1341 (m), 1317 (w), 1268 (m), 1249 (w), 1193 (s), 1152 (m), 1085 (m), 1023 (m), 908 (w), 873 (m), 812 (w), 762 (w), 720 (w), 673 (w).

methyl 2-(3-benzylcyclobutylidene)acetate (1i)

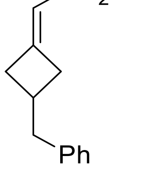

1i

Compound **1i** (53.2 mg, 0.246 mmol, 82%) was prepared according to general procedure A2.

Colorless oil.

Rf = 0.32 (1/12 Et_2O/Hexane).

^{1}**H NMR** (600 MHz, $CDCl_3$) δ 7.31 – 7.27 (m, 2H), 7.22 – 7.19 (m, 1H), 7.18 – 7.15 (m, 2H), 5.65 (p, *J* = 2.3 Hz, 1H), 3.68 (s, 3H), 3.30 – 3.23 (m, 1H), 2.94 – 2.88 (m, 1H), 2.86 – 2.78 (m, 3H), 2.76 – 2.67 (m, 1H), 2.58 – 2.52 (m, 1H).

^{13}**C NMR** (151 MHz, $CDCl_3$) δ 167.1, 164.4, 140.4, 128.7, 128.5, 126.2, 113.0, 51.0, 42.3, 39.3, 37.9, 32.5.

HRMS (Sicrit plasma/LTQ-Orbitrap) m/z: $[M + H]^+$ Calcd for $C_{14}H_{17}O_2^+$ 217.1223; Found 217.1223.

IR (ν_{max}, cm^{-1}) 3060 (w), 3026 (w), 2948 (w), 2911 (w), 2843 (w), 1715 (s), 1675 (m), 1435 (w), 1343 (m), 1268 (m), 1195 (s), 1098 (m), 1032 (w), 859 (w), 749 (w), 725 (w), 701 (m).

ethyl 2-cyclopentylideneacetate (1j)

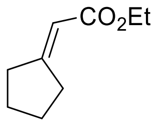

1j

Compound **1j** (716 mg, 4.64 mmol, 93%) was prepared according to general procedure A2. The analytical data were in agreement with those reported in the literature.[7]

Colorless oil.

Rf = 0.46 (1/9 Et_2O/Hexane).

^{1}**H NMR** (400 MHz, $CDCl_3$) δ 5.79 (p, *J* = 2.4 Hz, 1H), 4.14 (q, *J* = 7.1 Hz, 2H), 2.78-2.73 (m, 2H), 2.46 – 2.39 (m, 2H), 1.79 – 1.70 (m, 2H), 1.69 – 1.60 (m, 2H), 1.26 (t, *J* = 7.1, 3H).

^{13}**C NMR** (101 MHz, $CDCl_3$) δ 169.2, 167.1, 111.8, 59.6, 36.1, 32.8, 26.6, 25.6, 14.5.

HRMS (ESI/QTOF) m/z: $[M + H]^+$ Calcd for $C_9H_{15}O_2^+$ 155.1067; Found 155.1067.

IR (ν_{max}, cm^{-1}) 2960 (w), 2871 (w), 1710 (s), 1652 (m), 1451 (w), 1420 (w), 1370 (w), 1350 (w), 1302 (w), 1265 (w), 1229 (w), 1195 (s), 1152 (w), 1119 (s), 1040 (m), 858 (m).

methyl 2-((3aR,6aS)-hexahydropentalen-2(1H)-ylidene)acetate (1k)

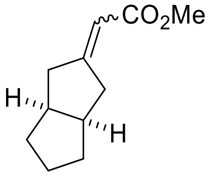

1k

Compound **1k** (71.6 mg, 0.40 mmol, 80%) was prepared according to general procedure A2 from corresponding ketone made with reported procedure.[8]

Colorless oil.

Rf = 0.55 (1/9 EtOAc/Hexane).

^{1}H NMR (400 MHz, $CDCl_3$) δ 5.73 (p, *J* = 2.1 Hz, 1H), 3.68 (s, 3H), 2.96 (ddt, *J* = 18.9, 9.0, 2.0 Hz, 1H), 2.72 – 2.54 (m, 3H), 2.53 – 2.42 (m, 1H), 2.24 (ddt, *J* = 17.2, 4.2, 1.7 Hz, 1H), 1.79 (dddd, *J* = 15.8, 12.6, 7.7, 6.4 Hz, 2H), 1.70 – 1.59 (m, 1H), 1.52 (dtt, *J* = 12.7, 7.7, 6.6 Hz, 1H), 1.41 – 1.33 (m, 1H), 1.33 – 1.23 (m, 1H).

^{13}C NMR (101 MHz, $CDCl_3$) δ 170.2, 167.5, 111.4, 51.0, 43.8, 42.5, 42.1, 38.7, 33.8, 33.1, 25.8.

HRMS (ESI/QTOF) m/z: $[M + H]^+$ Calcd for $C_{11}H_{17}O_2^+$ 181.1223; Found 181.1224.

IR (ν_{max}, cm^{-1}) 2944 (m), 2864 (w), 1714 (s), 1656 (m), 1433 (m), 1361 (m), 1278 (w), 1206 (s), 1157 (m), 1125 (s), 1025 (m), 944 (w), 912 (w), 860 (m), 808 (w), 725 (w).

methyl 2-cycloheptylideneacetate (1l)

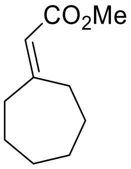

1l

Compound **1l** (120 mg, 0.72 mmol, 72%) was prepared according to general procedure A2.

The analytical data were in agreement with those reported in the literature.[1]

Colorless oil.

Rf = 0.46 (1/9 Et_2O/Hexane).

^{1}H NMR (400 MHz, $CDCl_3$) δ 5.66 (p, *J* = 1.3 Hz, 1H), 3.67 (s, 3H), 2.90 – 2.83 (m, 2H), 2.39 – 2.33 (m, 2H), 1.72 – 1.59 (m, 4H), 1.57 – 1.47 (m, 4H).

^{13}C NMR (101 MHz, $CDCl_3$) δ 167.3, 167.2, 115.3, 50.8, 39.1, 32.2, 29.9, 29.2, 28.2, 26.7.

HRMS (nanochip-ESI/LTQ-Orbitrap) m/z: $[M + H]^+$ Calcd for $C_{10}H_{17}O_2^+$ 169.1223; Found 169.1224.

IR (ν_{max}, cm^{-1}) 2924 (m), 2853 (w), 1714 (s), 1631 (m), 1433 (m), 1379 (w), 1241 (m), 1194 (m), 1174 (m), 1146 (s), 1027 (m), 924 (w), 874 (w), 845 (w).

2-cycloheptylidene-N-propylacetamide (1m)

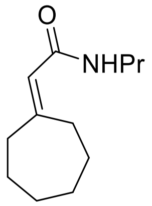

1m

A solution of KOH (101 mg, 1.8 mmol, 1.2 equiv) in MeOH/H_2O (3/1 v/v, 1.8 mL, 1.0 M) was added to a solution of **1l** (252 mg, 1.5 mmol, 1.0 equiv) in THF (0.5 mL). The reaction mixture was stirred at rt for 16 h. After completion, volatiles were removed under reduced pressure. The residue was treated with 1 N aqueous NaOH solution, and the aqueous layer was washed twice with DCM. The aqueous phase was then acidified to pH 1 with 1 N aqueous HCl solution and extracted three times with DCM. The combined organic extracts were washed with brine, dry over Na_2SO_4, filtered and concentrated under reduced pressure to afford the pure corresponding carboxylic acid (180 mg, 1.2 mmol, 78%) as a colorless oil which was used without further purification.

To a solution of acid (154.2 mg, 1.0 mmol, 1.0 equiv), DIPEA (523 µL, 3.0 mmol, 3.0 equiv) and propylamine (123 µL, 1.5 mmol, 1.5 equiv) in DCM (10 mL), was added HATU (570.3 mg, 1.5 mmol, 1.5 equiv) portionwise and the mixture was stirred at rt for 2 h. A saturated aqueous solution of NH_4Cl was then added and the mixture was extracted three times with DCM. The combined organic layers were washed with brine, dried over Na_2SO_4, filtered and

concentrated under reduced pressure. The crude product was purified by FCC (2/3 EtOAc/Hexane) to give amide **1m** (169 mg, 865 µmol, 87%) as a white solid.

White solid.

Rf = 0.48 (2/3 EtOAc/Hexane).

^{1}H NMR (400 MHz, $CDCl_3$) δ 5.52 (p, *J* = 1.3 Hz, 1H), 5.48 (brs, 1H), 3.25 – 3.18 (m, 2H), 2.88 – 2.84 (m, 2H), 2.31 – 2.26 (m, 2H), 1.69 – 1.57 (m, 4H), 1.55 – 1.46 (m, 6H), 0.91 (t, *J* = 7.4 Hz, 3H).

^{13}C NMR (101 MHz, $CDCl_3$) δ 167.2, 160.4, 118.3, 41.0, 38.9, 31.6, 29.9, 29.1, 28.3, 27.0, 23.1, 11.6.

HRMS (ESI/QTOF) m/z: $[M + H]^+$ Calcd for $C_{12}H_{22}NO^+$ 196.1696; Found 196.1697.

IR (ν_{max}, cm^{-1}) 3295 (w), 2963 (w), 2923 (s), 2853 (m), 1648 (s), 1624 (s), 1538 (s), 1456 (m), 1442 (m), 1257 (m), 1211 (m), 1167 (w), 1151 (w), 954 (w), 908 (m), 877 (w), 843 (w), 730 (s).

Mp = 37 – 39 °C.

1-cyclohexylidenepropan-2-one (1n)

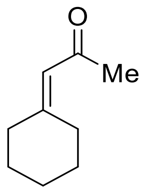

1n

Compound **1n** (260 mg, 1.88 mmol, 94%) was prepared according to reported procedure.[9]

The analytical datas were in agreement with the reported literature.[9]

Colorless oil.

Rf = 0.50 (1/4 Et_2O/Hexane).

^{1}H NMR (400 MHz, $CDCl_3$) δ 5.98 (s, 1H), 2.80 – 2.75 (m, 2H), 2.16 (s, 3H), 2.18 – 2.11 (m, 2H), 1.69 – 1.62 (m, 2H), 1.62-1.57 (m, 4H).

^{13}C NMR (101 MHz, $CDCl_3$) δ 199.6, 162.0, 121.5, 38.2, 32.0, 30.0, 28.9, 28.0, 26.4.

HRMS (nanochip-ESI/LTQ-Orbitrap) m/z: $[M + H]^+$ Calcd for $C_9H_{15}O^+$ 139.1117; Found 139.1124.

IR (ν_{max}, cm^{-1}) 2929 (m), 2855 (w), 1685 (s), 1618 (s), 1445 (m), 1383 (w), 1352 (m), 1267 (w), 1236 (w), 1202 (m), 1016 (w), 961 (m), 935 (w), 919 (w), 874 (w), 858 (w), 834 (w), 774 (w), 732 (w), 663 (m), 602 (m).

1-(hex-1-yn-1-yl)cyclohexyl acetate (S1)

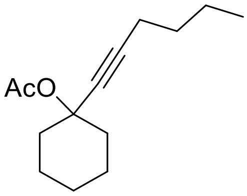

S1

Compound S**1** (480 mg, 2.2 mmol, 72%) was prepared according to reported procedure.[10]

The analytical data were in agreement with those reported in the literature.[10]

Colorless oil.

Rf = 0.45 (1/9 EtOAc/Hexane).

^{1}H NMR (400 MHz, $CDCl_3$) δ 2.23 (t, J = 7.0 Hz, 2H), 2.13 – 2.04 (m, 2H), 2.01 (s, 3H), 1.83-1.76 (m, 2H), 1.64 – 1.55 (m, 4H), 1.54 – 1.45 (m, 3H), 1.45 – 1.36 (m, 2H), 1.36 – 1.22 (m, 1H), 0.90 (t, J = 7.2 Hz, 3H).

^{13}C NMR (101 MHz, $CDCl_3$) δ 169.4, 87.0, 80.2, 76.2, 37.5, 30.9, 25.4, 22.9, 22.3, 22.1, 18.6, 13.7.

HRMS (APCI/QTOF) m/z: $[M + Na]^+$ Calcd for $C_{14}H_{22}NaO_2^+$ 245.1512; Found 245.1502.

IR (ν_{max}, cm^{-1}) 2933 (m), 2860 (w), 1743 (s), 1449 (w), 1366 (m), 1301 (w), 1264 (w), 1227 (s), 1185 (w), 1129 (w), 1017 (m), 961 (w), 911 (w), 840 (w), 815 (w), 745 (w).

1-cyclohexylidenehexan-2-one (1o)

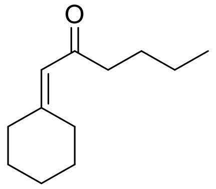

1o

Compound **1o** (179 mg, 0.99 mmol, 92%) was prepared from **S1** according to reported procedure.[10]

The analytical data were in agreement with those reported in the literature.[10]

Colorless oil.

Rf = 0.32 (1/20 EtOAc/Hexane).

^{1}H NMR (400 MHz, $CDCl_3$) δ 5.96 (p, *J* = 1.2 Hz, 1H), 2.82 – 2.76 (m, 2H), 2.43 – 2.37 (m, 2H), 2.17 – 2.13 (m, 2H), 1.69-1.52 (m, 8H), 1.36 – 1.27 (m, 2H), 0.90 (t, *J* = 7.3 Hz, 3H).

^{13}C NMR (101 MHz, $CDCl_3$) δ 202.3, 161.6, 121.2, 44.4, 38.2, 30.1, 29.0, 28.1, 26.6, 26.4, 22.6, 14.1.

HRMS (ESI/QTOF) m/z: $[M + H]^+$ Calcd for $C_{12}H_{21}O^+$ 181.1587; Found 181.1590.

IR (ν_{max}, cm^{-1}) 2929 (s), 2857 (m), 1684 (s), 1619 (s), 1446 (m), 1384 (w), 1342 (w), 1236 (m), 1135 (m), 1062 (m), 982 (w), 916 (m), 842 (w), 732 (s).

1-(4-(tert-butyl)cyclohexylidene)propan-2-one (1p)

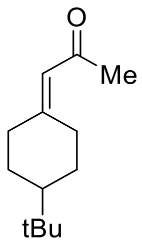

1p

Dimethylacetylmethylphosphonate (415 µL, 3.0 mmol, 1.5 equiv) was added dropwise at 0 °C to a solution of KOH (168 mg, 3.0 mmol, 1.5 equiv) in a mixture of EtOH/H_2O (4/1 v/v, 6.1 mL) and the mixture was stirred for 10 minutes. 4-*tert*-Butyl cyclohexanone (309 mg, 2.0

mmol, 1.0 equiv) was then added and the reaction mixture was stirred at rt for 36 h. The reaction mixture was then diluted with diethyl ether and water and the aqueous phase was extracted with diethyl ether three times. The combined organic phases were washed with brine, dried over Na_2SO_4, filtered and the volatiles were removed under reduced pressure. The residue was purified by FCC (1/9 EtOAc/Hexane) to afford compound **1p** (369 mg, 1.9 mmol, 95%) as a light-yellow oil.

Light-yellow oil.

Rf = 0.50 (1/9 EtOAc/Hexane).

^{1}H NMR (400 MHz, $CDCl_3$) δ 5.98 (t, J = 1.8 Hz, 1H), 3.86 – 3.78 (m, 1H), 2.27 – 2.11 (m, 2H), 2.16 (s, 3H), 1.98-1.88 (m, 2H), 1.84-1.75 (m, 1H), 1.26 (tt, J = 11.6, 2.9 Hz, 1H), 1.22 – 1.04 (m, 2H), 0.85 (s, 9H).

^{13}C NMR (101 MHz, $CDCl_3$) δ 199.6, 162.1, 121.2, 47.9, 38.1, 32.6, 32.0, 29.7, 29.5, 28.6, 27.7.

HRMS (nanochip-ESI/LTQ-Orbitrap) m/z: $[M + H]^+$ Calcd for $C_{13}H_{23}O^+$ 195.1743; Found 195.1752.

IR (ν_{max}, cm^{-1}) 2948 (s), 2866 (m), 2843 (w), 1714 (w), 1686 (s), 1620 (s), 1478 (w), 1469 (w), 1444 (m), 1393 (m), 1364 (s), 1354 (m), 1266 (w), 1243 (m), 1227 (m), 1186 (s), 1163 (s), 1015 (w), 967 (m), 956 (m), 842 (m), 764 (w), 674 (m).

2-cyclohexylideneacetic acid (S2)

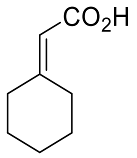

S2

A solution of KOH (1.25 g, 22.2 mmol, 1.2 equiv) in MeOH/H_2O (3/1 v/v, 18.5 mL, 1.0 M) was added to **1a** (2.85 g, 18.5 mmol, 1.0 equiv). The reaction mixture was stirred at rt for 16 h. After completion, volatiles were removed under reduced pressure. The residue was treated with 1 N aqueous NaOH solution, and the aqueous layer was washed twice with DCM. The aqueous phase was then acidified to pH 1 with 1 N aqueous HCl solution and extracted three times with

DCM. The combined organic extracts were washed with brine, dry over Na_2SO_4, filtered and concentrated under reduced pressure to afford the pure corresponding carboxylic acid (2.58 g, 18.4 mmol, 99%) as white crystals which was used without further purification.

The analytical data were in agreement with those reported in the literature.[11]

2-cyclohexylidene-N-propylacetamide (1q)

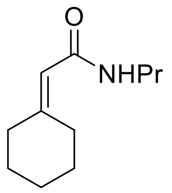

1q

To a solution of acid **S2** (140.2 mg, 1.0 mmol, 1.0 equiv), DIPEA (523 µL, 3.0 mmol, 3.0 equiv) and propylamine (123 µL, 1.5 mmol, 1.5 equiv) in DCM (10 mL), was added HATU (570.3 mg, 1.5 mmol, 1.5 equiv) portionwise and the mixture was stirred at rt for 4 h. A saturated aqueous solution of NH_4Cl was then added and the mixture was extracted three times with DCM. The combined organic layers were washed with brine, dried over Na_2SO_4, filtered and concentrated under reduced pressure. The crude product was purified by FCC (1/1 EtOAc/Hexane) to give amide **1q** (170 mg, 0.94 µmol, 94%) as a white solid.

White solid.

Rf = 0.55 (1/1 EtOAc/Hexane).

^{1}H NMR (400 MHz, $CDCl_3$) δ 5.48 (t, J = 1.2 Hz, 1H), 5.45 (br s, 1H), 3.27 – 3.19 (m, 2H), 2.80-2.77 (m, 2H), 2.15 – 2.10 (m, 2H), 1.66 – 1.55 (m, 6H), 1.52 (sext, J = 7.3 Hz, 2H), 0.92 (t, J = 7.4 Hz, 3H).

^{13}C NMR (101 MHz, $CDCl_3$) δ 167.3, 157.4, 116.0, 41.0, 37.9, 29.8, 28.7, 27.9, 26.5, 23.1, 11.6.

HRMS (nanochip-ESI/LTQ-Orbitrap) m/z: $[M + H]^+$ Calcd for $C_{11}H_{20}NO^+$ 182.1539; Found 182.1548.

IR (ν_{max}, cm^{-1}) 3290 (w), 3068 (w), 2961 (w), 2927 (s), 2854 (m), 1658 (s), 1629 (s), 1540 (s), 1446 (m), 1381 (w), 1311 (w), 1273 (m), 1247 (s), 1226 (s), 1186 (m), 1151 (w), 967 (w), 935 (w), 865 (w), 851 (m), 761 (w).

Mp = 38 – 40 °C.

N-methyl-2-(4-phenylcyclohexylidene)acetamide (1r)

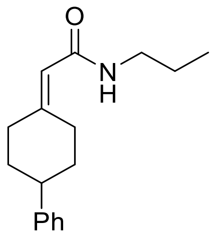

1r

A solution of KOH (276 mg, 4.93 mmol, 1.2 equiv) in MeOH/H_2O (3/1 v/v, 4.9 mL, 1.0 M) was added to **1f** (947 mg, 4.1 mmol, 1.0 equiv). The reaction mixture was stirred at rt for 16 h. After completion, volatiles were removed under reduced pressure. The residue was treated with 1 N aqueous NaOH solution, and the aqueous layer was washed twice with DCM. The aqueous phase was then acidified to pH 1 with 1 N aqueous HCl solution and extracted three times with DCM. The combined organic extracts were washed with brine, dry over Na_2SO_4, filtered and concentrated under reduced pressure to afford the pure corresponding carboxylic acid (1.05 g, 4.9 mmol, 99%) as white crystals which was used without further purification.

To a solution of acid (433 mg, 2.0 mmol, 1.0 equiv), DIPEA (1.05 mL, 6.0 mmol, 3.0 equiv) and propylamine (246 µL, 3.0 mmol, 1.5 equiv) in DCM (20 mL), was added HATU (1.14 g, 3.0 mmol, 1.5 equiv) portionwise and the mixture was stirred at rt for 4 h. A saturated aqueous solution of NH_4Cl was then added and the mixture was extracted three times with DCM. The combined organic layers were washed with brine, dried over Na_2SO_4, filtered and concentrated under reduced pressure. The crude product was purified by FCC (1/1 EtOAc/Hexane) to give amide **1r** (340.1 mg, 1.32 mmol, 66%) as white crystals.

White crystals.

Rf = 0.36 (1/2 EtOAc/Hexane).

^{1}**H NMR** (400 MHz, $CDCl_3$) δ 7.32 – 7.27 (m, 2H), 7.22 – 7.15 (m, 3H), 5.57 (s, 1H), 5.43 (brs, 1H), 4.01 – 3.92 (m, 1H), 3.30 – 3.22 (m, 2H), 2.76 (tt, *J* = 12.3, 3.5 Hz, 1H), 2.36-2.30 (m, 2H), 2.10 – 1.96 (m, 3H), 1.69 – 1.50 (m, 4H), 0.95 (t, *J* = 7.4 Hz, 3H).

^{13}**C NMR** (101 MHz, $CDCl_3$) δ 167.1, 156.0, 146.4, 128.6, 126.9, 126.3, 116.6, 44.3, 41.1, 37.7, 35.7, 34.9, 29.4, 23.1, 11.6.

HRMS (ESI/QTOF) m/z: $[M + H]^+$ Calcd for $C_{17}H_{24}NO^+$ 258.1852; Found 258.1852.

IR (ν_{max}, cm^{-1}) 3235 (w), 3062 (w), 2965 (w), 2926 (m), 2872 (w), 2851 (w), 1740 (w), 1659 (m), 1621 (s), 1549 (s), 1490 (m), 1445 (m), 1372 (m), 1318 (w), 1271 (s), 1216 (s), 1155 (m), 973 (w), 858 (m), 752 (s), 695 (s).

Mp = 97 – 99 °C.

2-cyclohexylideneacetamide (1s)

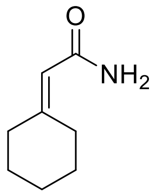

1s

To a solution of acid **S2** (140.2 mg, 1.0 mmol, 1.0 equiv), DIPEA (1.4 mL, 8.0 mmol, 8.0 equiv) and HATU (760.5 mg, 2.0 mmol, 2.0 equiv) in DMF (10 mL, 0.1 M) was added NH_4Cl (321 mg, 6.0 mmol, 6.0 equiv) and the mixture was stirred at rt for 1 h. The reaction mixture was diluted with aqueous solution of NH_4Cl and water, and it was extracted three times with EtOAc. The combined organic layers were washed with brine, dried over Na_2SO_4, filtered and concentrated under reduced pressure. The crude product was purified by FCC (4/1 EtOAc/Hexane) to give amide **1s** (121.5 mg, 0.87 mmol, 87%) as white crystals.

White crystals.

Rf = 0.38 (4/1 EtOAc/Hexane).

^{1}**H NMR** (400 MHz, $CDCl_3$) δ 5.55 (s, 1H), 5.38 (brs, 2H), 2.80 (t, *J* = 5.7 Hz, 2H), 2.16 (t, *J* = 5.9 Hz, 2H), 1.68 – 1.53 (m, 6H).

^{13}**C NMR** (101 MHz, $CDCl_3$) δ 169.2, 159.9, 114.6, 38.0, 29.8, 28.7, 27.9, 26.4.

HRMS (Sicrit plasma/LTQ-Orbitrap) m/z: $[M + H]^+$ Calcd for $C_8H_{14}NO^+$ 140.1070; Found 140.1069.

IR (ν_{max}, cm^{-1}) 3345 (m), 3175 (m), 2967 (w), 2927 (s), 2851 (m), 1665 (s), 1610 (s), 1443 (m), 1414 (s), 1336 (m), 1318 (m), 1260 (m), 1230 (m), 1193 (m), 1117 (w), 1079 (w), 997 (w), 937 (w), 894 (w), 854 (m), 776 (w), 674 (s), 654 (s).

Mp = 143 – 145 °C.

2-cyclohexylideneacetonitrile (1t)

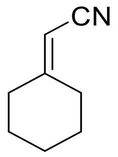

1t

Compound **1t** (22.7 mg, 187 μmol, 94%) was prepared from **1s** according to general procedure B.

The analytical data were in agreement with those reported in the literature.[12]

Colorless oil.

Rf = 0.60 (1/9 EtOAc/Hexane).

^{1}H NMR (400 MHz, $CDCl_3$) δ 5.03 (s, 1H), 2.48 (t, *J* = 5.8 Hz, 2H), 2.24 (t, *J* = 5.5 Hz, 2H), 1.71 – 1.55 (m, 6H).

^{13}C NMR (101 MHz, $CDCl_3$) δ 168.8, 117.1, 92.1, 36.1, 33.3, 28.1, 27.7, 25.7.

HRMS (APCI/QTOF) m/z: $[M + H]^+$ Calcd for $C_8H_{12}N^+$ 122.0964; Found 122.0959.

IR (ν_{max}, cm^{-1}) 2935 (s), 2858 (m), 2215 (s), 1631 (s), 1448 (s), 1443 (s), 1366 (w), 1344 (m), 1324 (w), 1307 (w), 1234 (w), 1134 (w), 1026 (w), 1007 (w), 969 (w), 931 (w), 896 (m), 859 (m), 808 (s), 775 (w), 652 (m).

methyl 2-(2-hexylcyclobutylidene)acetate (1u)

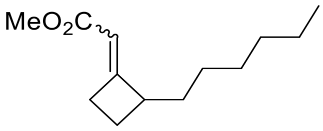

1u

Compound **1u** (4:1 mixture of isomers, 156 mg, 742 µmol, 74%) was prepared according to general procedure A2 from known corresponding ketone.

Colorless oil.

Rf = 0.30 (1/50 Et_2O/Hexane).

^{1}H NMR (400 MHz, $CDCl_3$) δ 5.61 (q, *J* = 2.4 Hz, 0.8H), 5.56 (q, *J* = 2.2 Hz, 0.2H), 3.67 (s, 2.4H), 3.66 (s, 0.6H), 3.39 – 3.28 (m, 0.2H), 3.12 – 2.90 (m, 2.4H), 2.89 – 2.79 (m, 0.2H), 2.69 – 2.60 (m, 0.2H), 2.25 – 2.11 (m, 0.8H), 1.97 – 1.86 (m, 0.2H), 1.75 – 1.20 (m, 11H), 0.89 – 0.85 (m, 3H).

^{13}C NMR (101 MHz, $CDCl_3$) δ 172.4 (major), 171.4 (minor), 167.3 (major), 166.6 (minor), 112.2 (minor), 110.7 (major), 51.0 (major), 50.9 (minor), 46.1 (minor), 45.2 (major), 33.7 (major), 32.9 (minor), 32.0 (minor), 31.9 (major), 31.0 (major), 30.1 (minor), 29.4 (major + minor), 27.0 (major), 26.9 (minor), 24.3 (major), 23.1 (minor), 22.8 (minor), 22.8 (major), 14.2 (minor), 14.2 (major).

HRMS (ESI/QTOF) m/z: $[M + H]^+$ Calcd for $C_{13}H_{23}O_2^+$ 211.1693; Found 211.1691.

IR (ν_{max}, cm^{-1}) 2926 (m), 2855 (m), 1716 (s), 1672 (m), 1460 (w), 1434 (m), 1340 (m), 1265 (m), 1236 (m), 1192 (s), 1174 (s), 1098 (w), 1028 (w), 913 (w), 854 (w), 731 (w).

2-(3-methoxypropyl)cyclobutan-1-one (S3)

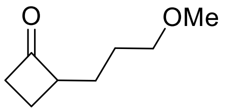

S3

A solution of cyclobutanone (1.5 mL, 20 mmol, 1.0 equiv), 1-aminopiperidine (3.2 mL, 30 mmol, 1.5 equiv) and TFA (77 µL, 1.0 mmol, 5 mol%) in dry toluene (20 mL) was refluxed for 3 h in the presence of $MgSO_4$. The reaction mixture was cooled to rt and an aqueous solution

of $NaHCO_3$ was added. The crude product was extracted three times with Et_2O. The combined organic layers were washed with brine, dried over Na_2SO_4, filtered and concentrated under reduced pressure. The crude hydrazone was used directly in the next step without further purification.

At -10 °C under argon, *n*-BuLi (2.5 M in hexanes, 8.3 mL, 20.7 mmol, 1.05 equiv) was added dropwise to a solution of crude hydrazone (3.0 g, 19.7 mmol, 1.0 equiv) in dry THF (39 mL). The resulting yellow solution was stirred at this temperature for 1 h before dropwise addition of 1-bromo-3-methoxypropane (2.3 mL, 20.1 mmol, 1.05 equiv) and the reaction mixture was stirred at rt for 16 h. 2 N HCl (39 mL, 78 mmol, 4.0 equiv) was added and the mixture was stirred for an additional 1 h. The crude product was extracted three times with Et_2O. The combined organic layers were washed with brine, dried over Na_2SO_4, filtered and concentrated under reduced pressure. Purification by FCC (1/2 Et_2O/Hexane) afforded ketone **S3** (1.7 g, 12 mmol, 60% over 2 steps) as a colorless oil.

Colorless oil.

Rf = 0.31 (3/7 Et_2O/Hexane).

^{1}H NMR (400 MHz, $CDCl_3$) δ 3.38-3.33 (m, 2H), 3.30 (s, 3H), 3.30-3.24 (m, 1H), 3.01 (dddd, *J* = 18.1, 10.5, 7.9, 2.8 Hz, 1H), 2.90 (dddd, *J* = 17.6, 9.6, 5.3, 2.7 Hz, 1H), 2.18 (ddt, *J* = 15.7, 10.4, 5.3 Hz, 1H), 1.78 – 1.53 (m, 5H).

^{13}C NMR (101 MHz, $CDCl_3$) δ 212.0, 72.4, 60.3, 58.7, 44.6, 27.3, 26.4, 17.0.

HRMS (ESI/QTOF) m/z: $[M + Na]^+$ Calcd for $C_8H_{14}NaO_2^+$ 165.0886; Found 165.0886.

IR (ν_{max}, cm^{-1}) 2927 (w), 2862 (w), 1775 (s), 1201 (w), 1117 (s), 1087 (m), 1059 (w), 915 (m), 730 (s).

methyl (E)-2-(2-(3-methoxypropyl)cyclobutylidene)acetate (1v)

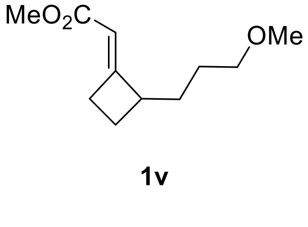

1v

Compound **1v** ((*E*)/(*Z*) = 9:2, 1.8 g, 9.1 mmol, 76%) was prepared according to general procedure A2.

Colorless oil.

Rf = 0.41 (1/2 Et_2O/Hexane).

^{1}H NMR (400 MHz, $CDCl_3$) δ 5.61 (q, *J* = 2.4 Hz, 1H major), 5.57 (q, *J* = 2.1 Hz, 1H minor), 3.67 (s, 3H major), 3.66 (s, 3H minor), 3.35 (t, *J* = 6.2 Hz, 2H major + 2H minor), 3.32 (s, 3H minor), 3.31 (s, 3H major), 3.13 – 2.91 (m, 3H major + 1H minor), 2.91 – 2.80 (m, 1H minor), 2.71 – 2.60 (m, 1H minor), 2.26 – 2.15 (m, 1H major + 1H minor), 2.01 – 1.89 (m, 1H minor), 1.77 – 1.44 (m, 5H major + 4H minor).

^{13}C NMR (101 MHz, $CDCl_3$) δ 171.7 (major), 170.7 (minor), 167.2 (major), 166.5 (minor), 112.5 (minor), 110.9 (major), 72.9 (minor), 72.6 (major), 58.7 (major), 58.7 (minor), 51.0 (major), 50.9 (minor), 45.7 (minor), 44.9 (major), 30.9 (major), 30.2 (major), 30.1 (minor), 29.5 (minor), 27.1 (minor), 27.1 (major), 24.1 (major), 23.1 (minor).

HRMS (ESI/QTOF) m/z: $[M + Na]^+$ Calcd for $C_{11}H_{18}NaO_3^+$ 221.1148; Found 221.1142.

IR (ν_{max}, cm^{-1}) 2978 (w), 2945 (w), 2859 (w), 1714 (s), 1672 (m), 1434 (m), 1341 (m), 1269 (m), 1239 (m), 1194 (s), 1174 (s), 1117 (s), 1093 (m), 1028 (m), 917 (m), 852 (m), 731 (s).

methyl (E)-2-(bicyclo[4.2.0]octan-7-ylidene)acetate (1w)

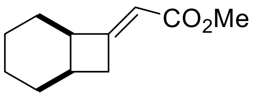

1w

Compound **1w** ((*E*)/(*Z*) 1.7:1, 765 mg, 4.24 mmol, 99%) was prepared according to general procedure A2.

Yellow oil.

Rf = 0.40 (1/9 Et_2O/Hexane).

^{1}H NMR (400 MHz, $CDCl_3$) δ 5.64 (q, *J* = 2.3 Hz, 1H, major), 5.54 (q, *J* = 1.9 Hz, 1H, minor), 3.68 (s, 3H, major), 3.66 (s, 3H, minor), 3.37 – 3.27 (m, 1H, minor), 3.08 (ddt, *J* = 9.3, 6.3, 2.8 Hz, 1H, major), 3.01 (ddd, *J* = 16.3, 8.2, 2.5 Hz, 1H, major), 2.77 – 2.68 (m, 1H, major + 1H

minor), 2.62 (dddd, *J* = 15.7, 8.1, 3.8, 1.5 Hz, 1H, minor), 2.51-2.44 (m, 1H, minor), 2.38 (pd, *J* = 8.3, 3.3 Hz, 1H, major), 2.10-2.02 (m, 1H, minor), 1.96 – 1.86 (m, 1H, major), 1.71 (dq, *J* = 13.4, 4.3 Hz, 1H, major), 1.64 – 1.04 (m, 6H major + 7H minor).

^{13}C NMR (101 MHz, $CDCl_3$) δ 170.1 (major), 169.4 (minor), 167.4 (major), 166.9 (minor), 110.7 (minor), 110.3 (major), 51.0 (major), 50.9 (minor), 43.5 (minor), 42.8 (major), 39.3 (major), 35.1 (minor), 29.6 (major), 29.0 (minor), 28.9 (major), 26.0 (minor), 25.6 (minor), 24.3 (major), 22.6 (major), 22.4 (minor), 21.7 (major), 21.3 (minor).

HRMS (ESI/QTOF) m/z: $[M + H]^+$ Calcd for $C_{11}H_{17}O_2^+$ 181.1223; Found 181.1227.

IR (ν_{max}, cm^{-1}) 2926 (m), 2852 (w), 1715 (s), 1674 (s), 1434 (m), 1338 (m), 1265 (m), 1192 (s), 1183 (s), 1138 (m), 1116 (m), 1091 (m), 1055 (w), 1036 (w), 1019 (m), 911 (w), 853 (m).

methyl 2-(1,4-dioxaspiro[4.5]decan-8-ylidene)acetate (S4)

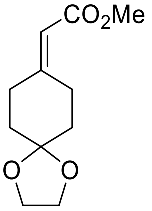

S4

Compound **S4** (6.9 g, 32.5 mmol, 81%) was prepared according to general procedure A1.

White crystals.

The analytical data were in agreement with those reported in the literature.[13]

Rf = 0.4 (1/4 EtOAc/Hexane).

^{1}H NMR (400 MHz, $CDCl_3$) δ 5.66 (t, *J* = 1.3 Hz, 1H), 3.99 – 3.95 (m, 4H), 3.68 (s, 3H), 3.02 – 2.97 (m, 2H), 2.40 – 2.35 (m, 2H), 1.80 – 1.72 (m, 4H).

^{13}C NMR (101 MHz, $CDCl_3$) δ 167.1, 160.7, 114.0, 108.1, 64.6, 51.1, 35.9, 35.1, 34.7, 26.2.

HRMS (APCI/QTOF) m/z: $[M + Na]^+$ Calcd for $C_{11}H_{16}NaO_4^+$ 235.0941; Found 235.0933.

IR (ν_{max}, cm^{-1}) 2950 (w), 2884 (w), 1714 (m), 1652 (m), 1434 (m), 1358 (w), 1337 (w), 1304 (w), 1274 (m), 1201 (m), 1165 (s), 1119 (s), 1086 (s), 1033 (s), 911 (s), 863 (m), 731 (m), 691 (m).

Mp = 40 – 41 °C.

4-(2-hydroxyethyl)cyclohexan-1-one (S5)

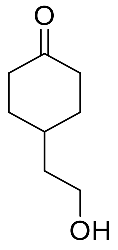

S5

Compound **S5** (3.3 g, 23.2 mmol, 72% over 3 steps) was prepared according to reported procedure from **S4** and the analytical data were in agreement with those reported in the literature.[14,15]

Colorless oil.

Rf = 0.20 (7/3 EtOAc/Hexane).

^{1}H NMR (400 MHz, $CDCl_3$) δ 3.74 (t, *J* = 6.6 Hz, 2H), 2.43 – 2.28 (m, 4H), 2.12 – 2.04 (m, 2H), 1.99 – 1.87 (m, 1H), 1.59 (q, *J* = 6.6 Hz, 2H), 1.54 (s, 1H), 1.49 – 1.36 (m, 2H).

^{13}C NMR (101 MHz, $CDCl_3$) δ 212.3, 60.8, 40.9, 38.4, 32.8, 32.7.

HRMS (Sicrit plasma/LTQ-Orbitrap) m/z: $[M + H]^+$ Calcd for $C_8H_{15}O_2^+$ 143.1067; Found 143.1066.

IR (ν_{max}, cm^{-1}) 3394 (w), 2927 (m), 2859 (w), 1705 (s), 1448 (w), 1421 (w), 1334 (w), 1249 (w), 1222 (w), 1166 (w), 1106 (w), 1092 (w), 1053 (m), 1017 (m), 961 (w), 930 (w), 868 (w), 841 (w), 785 (w), 748 (w), 683 (w).

methyl 2-(4-(2-hydroxyethyl)cyclohexylidene)acetate (1x)

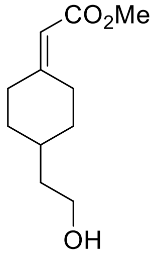

1x

Compound **1x** (3.0 g, 15.1 mmol, 73%) was prepared according to general procedure A1.

Colorless oil.

Rf = 0.45 (1/1 EtOAc/Hexane).

^{1}H NMR (400 MHz, $CDCl_3$) δ 5.61 (t, *J* = 1.6 Hz, 1H), 3.78 – 3.71 (m, 1H), 3.71 – 3.67 (m, 2H), 3.66 (s, 3H), 2.31 – 2.24 (m, 1H), 2.23 – 2.12 (m, 1H), 2.01 – 1.87 (m, 3H), 1.76 – 1.63 (m, 1H), 1.54 (s, 1H), 1.49 (q, *J* = 6.7 Hz, 2H), 1.20 – 1.03 (m, 2H).

^{13}C NMR (101 MHz, $CDCl_3$) δ 167.4, 163.4, 112.9, 60.8, 51.0, 39.2, 37.3, 34.6, 33.9, 33.8, 29.0.

HRMS (nanochip-ESI/LTQ-Orbitrap) m/z: $[M + H]^+$ Calcd for $C_{11}H_{19}O_3^+$ 199.1329; Found 199.1338.

IR (ν_{max}, cm^{-1}) 3379 (w), 2924 (m), 2850 (w), 1713 (s), 1647 (m), 1436 (m), 1381 (m), 1315 (w), 1263 (m), 1190 (m), 1168 (s), 1146 (s), 1086 (w), 1054 (m), 1025 (m), 965 (w), 938 (w), 898 (w), 859 (m), 726 (w), 674 (w).

methyl 2-(4-(2-(tosyloxy)ethyl)cyclohexylidene)acetate (1y)

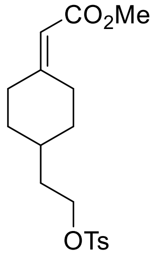

1y

At 0 °C, TsCl (158.6 mg, 0.83 mmol, 1.5 equiv) was added portionwise to a solution of alcohol **1x** (110.1 mg, 0.55 mmol, 1.0 equiv) and NEt_3 (523 µL, 3.0 mmol, 3.0 equiv) in DCM (2.8 mL), and the mixture was stirred at rt for 16 h. The reaction mixture was quenched by addition of water and it was extracted three times with DCM. The combined organic layers were washed with brine, dried over Na_2SO_4, filtered and concentrated under reduced pressure. The crude product was purified by FCC (1/4 EtOAc/Hexane) to give tosylated product **1y** (180.1 mg, 0.51 mmol, 93%) as a white crystalline solid.

White crystals.

Rf = 0.46 (1/4 EtOAc/Hexane).

^{1}H NMR (400 MHz, $CDCl_3$) δ 7.81 – 7.76 (m, 2H), 7.37 – 7.32 (m, 2H), 5.59 (t, *J* = 1.7 Hz, 1H), 4.06 (td, *J* = 6.3, 1.9 Hz, 2H), 3.74-3.68 (m, 1H), 3.66 (s, 3H), 2.45 (s, 3H), 2.23 (apparent dtd, *J* = 13.5, 3.9, 1.8 Hz, 1H), 2.11 (apparent tdd, *J* = 13.3, 4.6, 1.7 Hz, 1H), 1.91 – 1.71 (m, 3H), 1.68-1.59 (m, 1H), 1.59 – 1.52 (m, 2H), 1.11 – 0.94 (m, 2H).

^{13}C NMR (101 MHz, $CDCl_3$) δ 167.2, 162.5, 144.9, 133.2, 130.0, 128.0, 113.2, 68.6, 51.0, 37.0, 35.2, 34.1, 33.4, 33.3, 28.8, 21.8.

HRMS (nanochip-ESI/LTQ-Orbitrap) m/z: $[M + Na]^+$ Calcd for $C_{18}H_{24}NaO_5S^+$ 375.1237; Found 375.1253.

IR (ν_{max}, cm^{-1}) 2926 (w), 2851 (w), 1712 (m), 1649 (w), 1598 (w), 1434 (w), 1382 (w), 1357 (m), 1264 (w), 1223 (w), 1188 (m), 1173 (s), 1148 (s), 1097 (m), 1028 (w), 948 (m), 902 (m), 861 (m), 814 (m), 781 (w), 751 (m), 736 (m), 662 (s).

Mp = 48 – 50 °C.

methyl 2-(4-(2-chloroethyl)cyclohexylidene)acetate (1z)

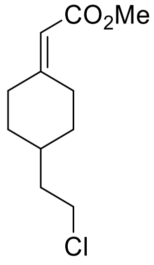

1z

To a solution of K_2CO_3 (69.0 mg, 0.5 mmol, 0.5 equiv) and PPh_3 (315 mg, 1.2 mmol, 1.2 equiv) in DCM (7 mL) at 0 °C was added NCS (160 mg, 1.2 mmol, 1.2 equiv). After 5 minutes at this temperature, a solution of alcohol **1x** (198.3 mg, 1.0 mmol, 1.0 equiv) in DCM (3 mL) was added dropwise. The reaction mixture was stirred at 0 °C for 15 minutes, then at rt for 5 h before addition of a saturated aqueous solution of $NaHCO_3$ to the reaction mixture. It was then extracted three times with DCM, the combined organic layers were washed with brine, dried over Na_2SO_4, filtered and concentrated under reduced pressure. The crude product was purified by FCC (1/20 EtOAc/Hexane) to give compound **1z** (211.9 mg, 0.98 mmol, 98%) as a colorless oil.

Colorless oil.

Rf = 0.50 (1/20 EtOAc/Hexane).

^{1}H NMR (400 MHz, $CDCl_3$) δ 5.62 (t, *J* = 1.6 Hz, 1H), 3.81 – 3.73 (m, 1H), 3.68 (s, 3H), 3.57 (t, *J* = 6.8 Hz, 2H), 2.33 – 2.26 (m, 1H), 2.24 – 2.15 (m, 1H), 2.01 – 1.87 (m, 3H), 1.84 – 1.73 (m, 1H), 1.73 – 1.66 (m, 2H), 1.19 – 1.03 (m, 2H).

^{13}C NMR (101 MHz, $CDCl_3$) δ 167.3, 162.8, 113.2, 51.0, 43.0, 39.0, 37.1, 34.5, 34.0, 33.3, 28.9.

HRMS (Sicrit plasma/LTQ-Orbitrap) m/z: $[M + H]^+$ Calcd for $C_{11}H_{18}ClO_2^+$ 217.0990; Found 217.0989.

IR (ν_{max}, cm^{-1}) 2925 (w), 2851 (w), 1713 (s), 1649 (m), 1434 (m), 1382 (w), 1316 (w), 1264 (m), 1230 (w), 1192 (m), 1144 (s), 1092 (w), 1028 (m), 1001 (w), 938 (w), 917 (w), 897 (w), 861 (m), 738 (m), 699 (m), 678 (w).

methyl 2-(4-(2-bromoethyl)cyclohexylidene)acetate (1aa)

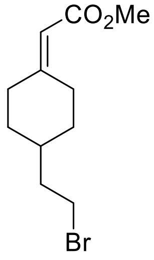

1aa

To a solution of CBr_4 (398 mg, 1.2 mmol, 1.2 equiv) and alcohol **1x** (198.3 mg, 1.0 mmol, 1.0 equiv) in THF (5 mL) at 0 °C was added PPh_3 (315 mg, 1.2 mmol, 1.2 equiv) portionwise and the reaction mixture was stirred at rt for 3 h. A saturated aqueous solution of $NaHCO_3$ was added to the reaction mixture and it was extracted three times with DCM, the combined organic layers were washed with brine, dried over Na_2SO_4, filtered and concentrated under reduced pressure. The crude product was purified by FCC (1/20 EtOAc/Hexane) to give compound **1aa** (245.0 mg, 0.94 mmol, 94%) as a colorless oil.

Colorless oil.

Rf = 0.46 (1/20 EtOAc/Hexane).

^{1}H NMR (400 MHz, $CDCl_3$) δ 5.62 (t, *J* = 1.7 Hz, 1H), 3.81 – 3.74 (m, 1H), 3.67 (s, 3H), 3.44 (t, *J* = 6.8 Hz, 2H), 2.29 (dtd, *J* = 13.4, 3.9, 1.8 Hz, 1H), 2.25 – 2.14 (m, 1H), 2.01 – 1.87 (m, 3H), 1.83 – 1.71 (m, 3H), 1.19 – 1.03 (m, 2H).

^{13}C NMR (101 MHz, $CDCl_3$) δ 167.3, 162.8, 113.2, 51.0, 39.2, 37.0, 35.7, 33.9, 33.2, 31.7, 28.8.

HRMS (Sicrit plasma/LTQ-Orbitrap) m/z: $[M + H]^+$ Calcd for $C_{11}H_{18}BrO_2^+$ 261.0485; Found 261.0485.

IR (ν_{max}, cm^{-1}) 2925 (w), 2851 (w), 1713 (s), 1649 (m), 1435 (m), 1382 (w), 1318 (w), 1263 (m), 1243 (w), 1194 (m), 1146 (s), 1027 (m), 937 (w), 902 (w), 860 (m), 749 (w), 728 (w), 678 (w).

methyl 2-(4-(2-cyanoethyl)cyclohexylidene)acetate (1ab)

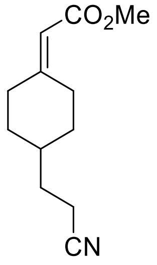

1ab

In a sealed tube, a solution of **1aa** (170 mg, 0.65 mmol, 1.0 equiv) and KCN (84 mg, 1.3 mmol, 2.0 equiv) in DMF (1.3 mL) was heated at 50 °C for 18 h. A saturated aqueous solution of K_2CO_3 was added to the reaction mixture and it was extracted three times with EtOAc, the combined organic layers were washed with 1 M NaOH, brine, dried over Na_2SO_4, filtered and concentrated under reduced pressure. The crude product was purified by FCC (1/2 EtOAc/Hexane) to give nitrile **1s** (102 mg, 0.49 mmol, 76%) as a colorless oil.

Colorless oil.

Rf = 0.57 (1/2 EtOAc/Hexane).

^{1}H NMR (400 MHz, $CDCl_3$) δ 5.63 (t, *J* = 1.7 Hz, 1H), 3.83 – 3.75 (m, 1H), 3.67 (s, 3H), 2.37 (t, *J* = 7.3 Hz, 2H), 2.34 – 2.26 (m, 1H), 2.25 – 2.15 (m, 1H), 2.01 – 1.88 (m, 3H), 1.77 – 1.63 (m, 1H), 1.62-1.56 (m, 2H), 1.19 – 1.03 (m, 2H).

^{13}C NMR (101 MHz, $CDCl_3$) δ 167.2, 162.1, 119.8, 113.5, 51.0, 36.8, 36.1, 33.8, 33.0, 31.7, 28.6, 15.0.

HRMS (Sicrit plasma/LTQ-Orbitrap) m/z: $[M + H]^+$ Calcd for $C_{12}H_{18}NO_2^+$ 208.1332; Found 208.1331.

IR (ν_{max}, cm^{-1}) 2930 (w), 2854 (w), 2245 (w), 1711 (s), 1649 (m), 1434 (m), 1383 (w), 1317 (w), 1263 (w), 1189 (m), 1164 (s), 1145 (s), 1098 (w), 1027 (m), 939 (w), 862 (m), 745 (w), 728 (w), 674 (w).

methyl 2-(4-(2-(1,3-dioxoisoindolin-2-yl)ethyl)cyclohexylidene)acetate (1ac)

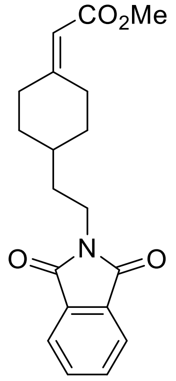

1ac

In a sealed tube, a solution of **1aa** (261.2 mg, 1.0 mmol, 1.0 equiv), K_2CO_3 (172.8 mg, 1.25 mmol, 1.25 equiv) and phthalimide (154.5 mg, 1.05 mmol, 1.05 equiv) in DMF (2 mL) was heated at 80 °C for 16 h. The reaction mixture was cooled at rt before addition of H_2O. It was then extracted three times with EtOAc, the combined organic layers were washed with brine, dried over Na_2SO_4, filtered and concentrated under reduced pressure. The crude product containing isomerized side-product was dissolved in dry DCM (10 mL) and a spatula tip of Sudan Red 7B dye was added before cooling the reaction mixture at -78 °C. At this temperature, a flow of O_3/O_2 (40%) was bubbled through the red/purple solution until a color change started to be observed. Argon was then bubbled into the mixture and PPh_3 (524.6 mg, 2.0 mmol, 2.0 equiv) was added. After warming up the mixture at rt, it was stirred for 2 h before removing volatiles under reduced pressure. The crude product was purified by FCC (1/4 EtOAc/Hexane) to give phthalimide **1ac** (183.2 mg, 0.56 mmol, 56%) as a white solid.

White solid.

Rf = 0.27 (1/4 EtOAc/Hexane).

^{1}H NMR (400 MHz, $CDCl_3$) δ 7.86 – 7.81 (m, 2H), 7.73 – 7.68 (m, 2H), 5.61 (t, *J* = 1.6 Hz, 1H), 3.79 – 3.68 (m, 3H), 3.67 (s, 3H), 2.29 (dtd, *J* = 13.5, 3.9, 1.7 Hz, 1H), 2.17 (tdd, *J* = 13.2, 4.7, 1.7 Hz, 1H), 2.06 – 1.89 (m, 3H), 1.69 – 1.49 (m, 3H), 1.29 – 1.07 (m, 2H).

^{13}C NMR (101 MHz, $CDCl_3$) δ 168.5, 167.3, 163.0, 134.0, 132.3, 123.3, 113.1, 51.0, 37.1, 36.1, 35.0, 34.9, 34.2, 33.7, 28.9.

HRMS (nanochip-ESI/LTQ-Orbitrap) m/z: $[M + Na]^+$ Calcd for $C_{19}H_{21}NNaO_4^+$ 350.1363; Found 350.1379.

IR (ν_{max}, cm^{-1}) 2928 (w), 2853 (w), 1772 (w), 1707 (s), 1649 (w), 1615 (w), 1436 (w), 1396 (m), 1371 (m), 1262 (w), 1234 (w), 1189 (m), 1150 (m), 1062 (w), 1028 (w), 995 (w), 945 (w), 905 (w), 867 (w), 794 (w), 719 (m).

Mp = 76 – 78 °C.

methyl 2-(4-(2-(4-methylbenzamido)ethyl)cyclohexylidene)acetate (1ad)

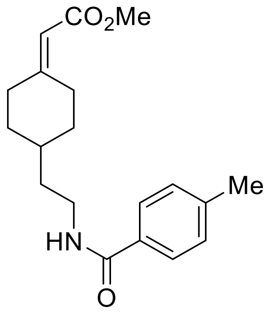

1ad

p-Toluamide (202.8 mg, 1.5 mmol, 1.5 equiv) was added at rt to a solution of NaH (60%wt, 44.0 mg, 1.1 mmol, 1.1 equiv) in DMF (4 mL) and the mixture was stirred at this temperature for 1 h before addition of bromide **1aa** (261.2 mg, 1.0 mmol, 1.0 equiv) in DMF (1 mL) to the reaction mixture. The solution was then heated at 50 °C for 16 h before being cooled to rt followed by addition of H_2O. It was then extracted three times with EtOAc, the combined organic layers were washed with brine, dried over Na_2SO_4, filtered and concentrated under reduced pressure. The crude product containing isomerized side-product was dissolved in dry DCM (10 mL) and a spatula tip of Sudan Red 7B dye was added before cooling the reaction mixture at -78 °C. At this temperature, a flow of O_3/O_2 (40%) was bubbled through the red/purple solution until a color change started to be observed. Argon was then bubbled into the mixture and PPh_3 (524.6 mg, 2.0 mmol, 2.0 equiv) was added. After warming up the mixture at rt, it was stirred for 2 h before removing volatiles under reduced pressure. The crude product was purified by FCC (1/2 EtOAc/Hexane) to give amide **1ad** (93.1 mg, 0.29 mmol, 29%) as a white solid.

White solid.

Rf = 0.35 (1/2 EtOAc/Hexane).

^{1}H NMR (400 MHz, $CDCl_3$) δ 7.65 (d, *J* = 8.2 Hz, 2H), 7.22 (d, *J* = 7.8 Hz, 2H), 6.08 (t, *J* = 4.6 Hz, 1H), 5.62 (s, 1H), 3.79 – 3.71 (m, 1H), 3.68 (s, 3H), 3.48 (td, *J* = 7.5, 5.8 Hz, 2H), 2.39

(s, 3H), 2.33 – 2.24 (m, 1H), 2.17 (td, J = 13.4, 4.5 Hz, 1H), 2.02 – 1.89 (m, 3H), 1.74 – 1.47 (m, 3H), 1.22-1.09 (m, 2H).

^{13}C NMR (101 MHz, $CDCl_3$) δ 167.6, 167.3, 163.0, 141.9, 132.0, 129.4, 127.0, 113.1, 51.0, 38.1, 37.2, 36.3, 35.1, 34.4, 33.8, 29.0, 21.6.

HRMS (ESI/QTOF) m/z: $[M + Na]^+$ Calcd for $C_{19}H_{25}NNaO_3^+$ 338.1727; Found 338.1729.

IR (ν_{max}, cm^{-1}) 3324 (w), 2921 (m), 2853 (w), 1717 (s), 1637 (s), 1543 (s), 1504 (m), 1436 (m), 1380 (w), 1301 (m), 1263 (m), 1189 (s), 1178 (s), 1148 (s), 1025 (w), 861 (w), 838 (w), 752 (m), 732 (w), 673 (w).

2-(4-(2-methoxy-2-oxoethylidene)cyclohexyl)ethyl 4-bromobenzoate (1ae)

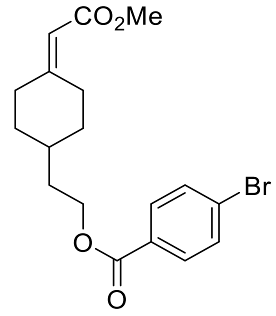

1ae

At 0 °C, EDC.HCl (230.1 mg, 1.2 mmol, 1.2 equiv) was added to a solution of alcohol **1x** (198.3 mg, 1.0 mmol, 1.0 equiv), 4-bromobenzoic acid (241.3 mg, 1.2 mmol, 1.2 equiv) and DMAP (24.4 mg, 0.2 mmol, 20 mol%) in DCM (5 mL) and the reaction mixture was stirred at rt for 14 h. A saturated aqueous solution of NH_4Cl was added to the reaction mixture and it was extracted three times with DCM. The combined organic layers were washed with brine, dried over Na_2SO_4, filtered and concentrated under reduced pressure. The crude product was purified by FCC (1/9 EtOAc/Hexane) to give ester **1ae** (378.4 mg, 0.99 mmol, 99%) as light-yellow crystals.

Light-yellow crystals.

Rf = 0.64 (1/4 EtOAc/Hexane).

^{1}H NMR (400 MHz, $CDCl_3$) δ 7.91 – 7.86 (m, 2H), 7.60 – 7.55 (m, 2H), 5.62 (s, 1H), 4.36 (t, *J* = 6.5 Hz, 2H), 3.81 – 3.74 (m, 1H), 3.68 (s, 3H), 2.34 – 2.26 (m, 1H), 2.19 (tdd, *J* = 13.0, 4.6, 1.6 Hz, 1H), 2.02 – 1.92 (m, 3H), 1.77 – 1.66 (m, 3H), 1.26 – 1.10 (m, 2H).

^{13}C NMR (101 MHz, $CDCl_3$) δ 167.3, 166.0, 162.8, 131.9, 131.2, 129.4, 128.1, 113.2, 63.6, 51.0, 37.1, 35.1, 34.4, 34.4, 33.8, 28.9.

HRMS (ESI/QTOF) m/z: $[M + Na]^+$ Calcd for $C_{18}H_{21}BrNaO_4^+$ 403.0515; Found 403.0515.

IR (ν_{max}, cm^{-1}) 2992 (w), 2927 (w), 2852 (w), 1713 (s), 1649 (m), 1590 (m), 1481 (w), 1435 (w), 1396 (w), 1383 (w), 1267 (s), 1175 (m), 1148 (s), 1103 (s), 1069 (m), 1028 (m), 1011 (m), 958 (w), 849 (m), 756 (s), 732 (m), 681 (w).

Mp = 77 – 79 °C.

4-(2-methoxy-2-oxoethylidene)cyclohexane-1-carboxylic acid (1af)

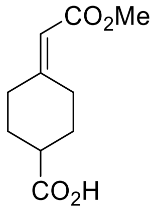

1af

Compound **1af** (184 mg, 0.93 mmol, 93%) was prepared according to general procedure A1 with 2.2 equiv of NaOMe.

White crystals.

Rf = 0.42 (3/7 EtOAc/Hexane + 2% AcOH).

^{1}H NMR (400 MHz, $CDCl_3$) δ 5.66 (s, 1H), 3.68 (s, 3H), 3.60 (dt, *J* = 14.3, 4.5 Hz, 1H), 2.60 (tt, *J* = 10.5, 3.9 Hz, 1H), 2.37 (dt, *J* = 13.9, 4.6 Hz, 1H), 2.28 – 2.15 (m, 2H), 2.14 – 2.04 (m, 2H), 1.78 – 1.62 (m, 2H).

^{13}C NMR (101 MHz, $CDCl_3$) δ 181.1, 167.1, 160.7, 114.1, 51.1, 42.1, 36.0, 29.9, 29.3, 27.9.

HRMS (nanochip-ESI/LTQ-Orbitrap) m/z: $[M + Na]^+$ Calcd for $C_{10}H_{14}NaO_4^+$ 221.0784; Found 221.0795.

IR (ν_{max}, cm^{-1}) 3044 (w), 3002 (w), 2950 (w), 2898 (w), 2865 (w), 2643 (w), 1710 (s), 1688 (s), 1653 (s), 1434 (m), 1385 (m), 1315 (m), 1265 (s), 1255 (m), 1230 (m), 1192 (s), 1177 (s), 1148 (s), 1120 (m), 1026 (m), 986 (m), 940 (m), 926 (m), 903 (m), 872 (s), 848 (m), 784 (w), 746 (m), 725 (w).

Mp = 49 – 51 °C.

diethyl 4-(2-methoxy-2-oxoethylidene)cyclohexane-1,1-dicarboxylate (1ag)

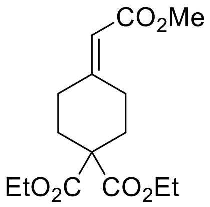

1ag

Compound **1ag** (295 mg, 0.99 mmol, 99%) was prepared according to general procedure A1.

Colorless oil.

Rf = 0.46 (1/4 EtOAc/Hexane).

^{1}H NMR (400 MHz, $CDCl_3$) δ 5.65 (t, *J* = 1.3 Hz, 1H), 4.20 (q, *J* = 7.1 Hz, 4H), 3.68 (s, 3H), 2.97 – 2.90 (m, 2H), 2.33 – 2.27 (m, 2H), 2.18-2.13 (m, 4H), 1.25 (t, *J* = 7.1 Hz, 6H).

^{13}C NMR (101 MHz, $CDCl_3$) δ 171.3, 167.0, 160.2, 114.1, 61.6, 54.7, 51.1, 33.7, 32.5, 31.9, 25.8, 14.2.

HRMS (APCI/QTOF) m/z: $[M + Na]^+$ Calcd for $C_{15}H_{22}NaO_6^+$ 321.1309; Found 321.1295.

IR (ν_{max}, cm^{-1}) 2980 (w), 2949 (w), 1725 (s), 1717 (s), 1654 (m), 1448 (w), 1436 (w), 1382 (w), 1367 (w), 1292 (m), 1242 (s), 1182 (s), 1156 (s), 1138 (s), 1099 (m), 1067 (m), 1026 (s), 861 (m), 657 (w).

methyl 2-((3aR,6aS)-5-oxohexahydropentalen-2(1H)-ylidene)acetate (1ah)

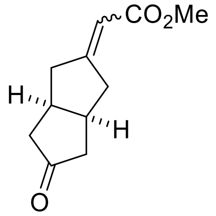

1ah

Compound **1ah** (301.3 mg, 1.86 mmol, 62%) was prepared according to general procedure A2.

White crystals.

Rf = 0.37 (1/2 EtOAc/Hexane).

^{1}H NMR (400 MHz, $CDCl_3$) δ 5.84 (t, *J* = 2.5 Hz, 1H), 3.69 (s, 3H), 3.16 (ddt, *J* = 19.8, 8.6, 2.5 Hz, 1H), 2.95 – 2.76 (m, 3H), 2.76 – 2.66 (m, 1H), 2.52 – 2.35 (m, 3H), 2.10 (dd, *J* = 19.1, 5.2 Hz, 1H), 2.00 (dd, *J* = 19.2, 5.6 Hz, 1H).

^{13}C NMR (101 MHz, $CDCl_3$) δ 219.4, 167.0, 166.7, 113.6, 51.1, 44.1, 43.2, 40.9, 40.3, 39.2, 38.1.

HRMS (ESI/QTOF) m/z: $[M + Na]^+$ Calcd for $C_{11}H_{14}NaO_3^+$ 217.0835; Found 217.0837.

IR (ν_{max}, cm^{-1}) 2949 (w), 2901 (w), 1737 (s), 1709 (s), 1655 (m), 1433 (m), 1407 (w), 1360 (m), 1282 (w), 1206 (s), 1171 (m), 1124 (s), 1021 (m), 932 (w), 912 (w), 876 (w), 847 (w), 813 (w), 727 (w).

Mp = 30.5 – 31.5 °C.

methyl 2-(tetrahydro-4H-pyran-4-ylidene)acetate (1ai)

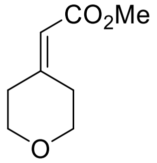

1ai

Compound **1ai** (142 mg, 0.91 mmol, 91%) was prepared according to general procedure A1.

The analytical data were in agreement with those reported in the literature.[1]

Colorless oil.

Rf = 0.37 (1/8 EtOAc/Hexane).

^{1}H NMR (400 MHz, $CDCl_3$) δ 5.68 (p, *J* = 1.3 Hz, 1H), 3.77 (t, *J* = 5.5 Hz, 2H), 3.73 (t, *J* = 5.6 Hz, 2H), 3.69 (s, 3H), 3.00 (td, *J* = 5.7, 1.3 Hz, 2H), 2.33 (td, *J* = 5.6, 1.2 Hz, 2H).

^{13}C NMR (101 MHz, $CDCl_3$) δ 167.0, 157.7, 114.2, 69.2, 68.6, 51.1, 37.7, 31.2.

HRMS (nanochip-ESI/LTQ-Orbitrap) m/z: $[M + H]^+$ Calcd for $C_8H_{13}O_3^+$ 157.0859; Found 157.0857.

IR (ν_{max}, cm^{-1}) 2951 (w), 2912 (w), 2846 (w), 1712 (s), 1651 (m), 1435 (m), 1387 (m), 1285 (w), 1250 (m), 1231 (m), 1201 (s), 1175 (m), 1146 (s), 1095 (m), 1029 (m), 1019 (m), 1005 (m), 988 (m), 862 (m), 852 (m), 683 (m).

methyl 2-((5S,8R,9S,10S,13S,14S)-10,13-dimethyl-17-oxohexadecahydro-3H-cyclopenta[a]phenanthren-3-ylidene)acetate (1aj)

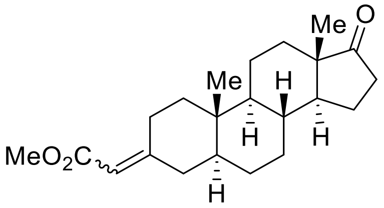

1aj

Compound **1aj** (1.18 g, 3.41 mmol, 74%) was prepared according to general procedure A1 and obtained as a 1:1 (*E*):(*Z*) mixture.

White solid.

Rf = 0.50 (1/3 EtOAc/Hexane).

^{1}H NMR (400 MHz, $CDCl_3$) (1:1 (*E*):(*Z*) mixture) δ 5.60 (t, *J* = 2.1 Hz, 0.5H), 5.58 (t, *J* = 2.4 Hz, 0.5H), 3.73 (brd, *J* = 15.4 Hz, 0.5H), 3.67 (s, 3H), 3.51 (dt, *J* = 14.2, 2.5 Hz, 0.5H), 2.43 (dd, *J* = 19.2, 8.8 Hz, 1H), 2.33 (td, *J* = 13.9, 4.9 Hz, 0.5H), 2.24 – 1.98 (m, 2H), 1.98 – 1.75 (m, 5H), 1.70 – 1.42 (m, 4H), 1.40 – 1.17 (m, 5.5H), 1.16 – 0.95 (m, 2H), 0.94 (s, 3H), 0.86 (s, 3H), 0.77 – 0.68 (m, 1H).

^{13}**C NMR** (101 MHz, $CDCl_3$) (1:1 (*E*):(*Z*) mixture) δ 221.4, 221.3, 167.4, 163.5, 163.4, 112.7, 112.5, 54.5, 54.4, 51.5, 51.0, 51.0, 48.3, 47.9, 47.6, 40.3, 40.1, 39.5, 36.4, 36.4, 36.0, 35.1, 33.6, 32.2, 31.7, 30.9, 30.9, 28.8, 28.6, 25.6, 21.9, 20.6, 20.6, 14.0, 12.1, 12.0.

HRMS (Sicrit plasma/LTQ-Orbitrap) m/z: $[M + H]^+$ Calcd for $C_{22}H_{33}O_3^+$ 345.2424; Found 345.2424.

IR (ν_{max}, cm^{-1}) 2970 (w), 2919 (m), 2851 (w), 1728 (s), 1715 (s), 1648 (m), 1438 (m), 1384 (w), 1246 (w), 1219 (m), 1175 (m), 1148 (s), 1056 (w), 1028 (m), 1011 (m), 951 (w), 923 (w), 888 (w), 863 (m), 829 (w), 753 (w), 732 (w), 681 (w).

Mp = 95 – 97 °C.

C) Dyotropic rearrangement: oxidative ring expansion to α-fluorinated β-dicarbonyl products

Conformation and relative stereochemistry:

X-Ray crystallographic analysis of compound **2q** reveals that the cycloheptanone ring adopts a chair conformation with the fluorine atom occupying a pseudo-axial position. The ^{19}F NMR spectrum (377 MHz, $CDCl_3$) shows a fluorine resonance at -161.97 ppm as a ddt (J = 42.0, 19.6, 4.7 Hz). Cycloheptanones **2a-2d**, **2n-2o, 2ag** and **2ai** exhibit similar ^{19}F NMR chemical shifts and coupling constants, consistent with a pseudo-axial F atom in a chair conformation.

For the C_5-monosubstituted cycloheptanones **2e-2f**, **2p**, **2r**, **2x-2ae**, **2aj-2aj'** a mixture of two diastereoisomers was obtained. The ^{19}F NMR spectra (377 MHz, $CDCl_3$) show fluorine resonances at δ -150 to -156 ppm and -161 to -166 ppm, corresponding to the major and minor isomers, respectively. Based on the F-H coupling constants, the fluorine atom adopts a pseudo-equatorial position in the major isomer and a pseudo-axial position in the minor isomer. For example, compound **2f-1** (major isomer) exhibits resonance at δ -150.72 ppm (t, J = 18.9 Hz), **2f-2** (minor isomer) resonances at δ -164.91 ppm (apparent dd, J = 36.3, 18.9 Hz,). The structure and relative stereochemistry of the minor diastereoisomer **2f-2**, in which the fluorine and phenyl groups are *cis* and the fluorine occupies a pseudo-axial position, were confirmed by X-ray crystallographic analysis.

For compounds **2k** and **2ah** (5,6-fused), the relative stereochemistry is determined by ^{1}H and ^{19}F NMR spectroscopic analysis (H-H and H-F coupling constants).

For compound **5**, F and CO_2Me are *trans*, and F is at pseudo-equatorial position, based on the H-H and H-F coupling constants.

methyl 1-fluoro-2-oxocycloheptane-1-carboxylate (2a)

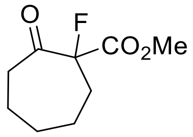

2a

Compound **2a** (23.1 mg, 122.7 µmol, 61%) was prepared according to general procedure B.

Compound **2a** (101.1 mg, 537.2 µmol, 54%) was prepared according to general procedure B.

Colorless oil.

Rf = 0.27 (1/4 Et_2O/Hexane).

^{1}H NMR (400 MHz, $CDCl_3$) δ 3.81 (s, 3H), 2.83 – 2.67 (m, 2H), 2.31 – 2.12 (m, 2H), 2.00 – 1.80 (m, 3H), 1.69 – 1.36 (m, 3H).

^{19}F NMR (376 MHz, $CDCl_3$) δ -163.50 (ddd, *J* = 35.5, 17.1, 3.2 Hz).

^{13}C NMR (101 MHz, $CDCl_3$) δ 204.4 (d, *J* = 20.8 Hz), 167.9 (d, *J* = 25.3 Hz), 99.2 (d, *J* = 198.4 Hz), 53.3, 40.9, 34.0 (d, *J* = 22.9 Hz), 29.1, 25.8, 23.9.

HRMS (APCI/QTOF) m/z: $[M + Na]^+$ Calcd for $C_9H_{13}FNaO_3^+$ 211.0741; Found 211.0735.

IR (ν_{max}, cm^{-1}) 2930 (w), 2859 (w), 1762 (s), 1725 (s), 1453 (m), 1437 (m), 1294 (m), 1276 (m), 1256 (m), 1240 (s), 1167 (m), 1150 (m), 1042 (s), 1009 (m), 939 (w), 888 (w), 842 (w), 804 (w), 782 (w), 742 (w), 669 (m).

ethyl 1-fluoro-2-oxocycloheptane-1-carboxylate (2b)

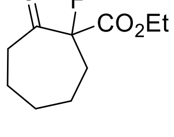

2b

Compound **2b** (20.3 mg, 100.3 µmol, 50%) was prepared according to general procedure B.

Colorless oil.

Rf = 0.50 (3/7 Et_2O/Hexane).

^{1}H NMR (400 MHz, $CDCl_3$) δ 4.26 (q, *J* = 7.1 Hz, 2H), 2.84 – 2.66 (m, 2H), 2.30 – 2.12 (m, 2H), 2.00 – 1.81 (m, 3H), 1.70 – 1.35 (m, 3H), 1.30 (t, *J* = 7.1 Hz, 3H).

^{19}F NMR (377 MHz, $CDCl_3$) δ -163.47 (ddd, *J* = 35.7, 17.3, 3.3 Hz).

^{13}C NMR (101 MHz, $CDCl_3$) δ 204.5 (d, *J* = 20.6 Hz), 167.4 (d, *J* = 25.3 Hz), 99.0 (d, *J* = 198.1 Hz), 62.6, 41.0, 34.0 (d, *J* = 23.0 Hz), 29.2, 25.9, 23.9, 14.1.

HRMS (ESI/QTOF) m/z: $[M + Na]^+$ Calcd for $C_{10}H_{15}FNaO_3^+$ 225.0897; Found 225.0900.

IR (ν_{max}, cm^{-1}) 2938 (w), 2863 (w), 1759 (s), 1724 (s), 1450 (m), 1369 (w), 1293 (m), 1271 (m), 1256 (m), 1237 (s), 1198 (w), 1168 (m), 1151 (m), 1094 (w), 1041 (s), 1020 (m), 943 (w), 890 (w), 857 (w), 788 (w), 768 (w), 742 (w).

benzyl 1-fluoro-2-oxocycloheptane-1-carboxylate (2c)

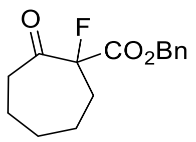

2c

Compound **2c** (19.6 mg, 96.9 µmol, 48%) was prepared according to general procedure B.

Colorless oil.

Rf = 0.30 (1/4 Et_2O/Hexane).

^{1}H NMR (400 MHz, $CDCl_3$) δ 7.40 – 7.31 (m, 5H), 5.24 (s, 2H), 2.80 – 2.67 (m, 2H), 2.33 – 2.12 (m, 2H), 1.99 – 1.80 (m, 3H), 1.70 – 1.49 (m, 2H), 1.47 – 1.35 (m, 1H).

^{19}F NMR (377 MHz, $CDCl_3$) δ -163.39 (dd, *J* = 35.5, 16.9 Hz).

^{13}C NMR (101 MHz, $CDCl_3$) δ 204.4 (d, *J* = 20.9 Hz), 167.3 (d, *J* = 25.8 Hz), 134.9, 128.8, 128.8, 128.3, 99.1 (d, *J* = 198.3 Hz), 68.0, 41.0, 34.0 (d, *J* = 22.9 Hz), 29.1, 25.8, 23.9 (d, *J* = 0.9 Hz).

HRMS (APCI/QTOF) m/z: $[M + Na]^+$ Calcd for $C_{15}H_{17}FNaO_3^+$ 287.1054; Found 287.1053.

IR (ν_{max}, cm^{-1}) 2933 (w), 2861 (w), 1760 (s), 1723 (s), 1454 (m), 1271 (s), 1234 (s), 1166 (s), 1149 (s), 1040 (s), 1002 (m), 945 (w), 908 (w), 888 (w), 841 (w), 742 (m), 697 (s).

methyl 1-fluoro-5,5-dimethyl-2-oxocycloheptane-1-carboxylate (2d)

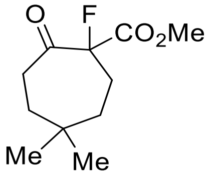

2d

Compound **2d** (26.9 mg, 124.4 µmol, 62%) was prepared according to general procedure B.

Light-yellow oil.

Rf = 0.35 (1/6 Et_2O/Hexane).

^{1}H NMR (400 MHz, $CDCl_3$) δ 3.82 (s, 3H), 2.89 (ddd, *J* = 13.4, 10.0, 5.6 Hz, 1H), 2.57 – 2.35 (m, 2H), 2.10 (tdd, *J* = 16.0, 7.5, 1.9 Hz, 1H), 1.65 – 1.54 (m, 3H), 1.48 (ddt, *J* = 15.2, 7.5, 1.8 Hz, 1H), 1.01 (s, 3H), 0.98 (s, 3H).

^{19}F NMR (377 MHz, $CDCl_3$) δ -163.40 (dd, *J* = 36.1, 16.0 Hz).

^{13}C NMR (101 MHz, $CDCl_3$) δ 204.1 (d, *J* = 19.9 Hz), 168.0 (d, *J* = 25.5 Hz), 98.9 (d, *J* = 198.4 Hz), 53.3, 38.5, 37.6, 36.2, 33.2, 32.3, 29.2 (d, *J* = 23.0 Hz), 25.1.

HRMS (nanochip-ESI/LTQ-Orbitrap) m/z: $[M + Na]^+$ Calcd for $C_{11}H_{17}FNaO_3^+$ 239.1054; Found 239.1049.

IR (ν_{max}, cm^{-1}) 2955 (m), 2924 (w), 2866 (w), 1766 (s), 1727 (s), 1459 (m), 1436 (m), 1368 (w), 1292 (m), 1270 (s), 1247 (m), 1211 (w), 1173 (m), 1131 (w), 1082 (m), 1028 (m), 1002 (m), 988 (w), 952 (w), 874 (w), 838 (w), 788 (w), 762 (w), 687 (w), 669 (w), 622 (w).

methyl 5-(tert-butyl)-1-fluoro-2-oxocycloheptane-1-carboxylate (2e)

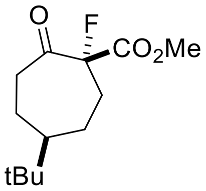

2e

Compound **2e** (dr 4.6:1) (22.0 mg, 90.0 µmol, 45%) was prepared according to general procedure B.

Semi solid. (**Mp** close to rt).

Rf = 0.42 (1/8 Et_2O/Hexane).

^{1}H NMR (600 MHz, $CDCl_3$) δ 3.82 (s, 3H, OMe, major), 3.80 (s, 3H, OMe, minor), 2.81 (td, *J* = 12.2, 3.8 Hz, 1H, CH_2, minor), 2.73 – 2.65 (m, 2H, CH_2, major + 1H, CH_2, minor), 2.63 – 2.54 (m, 1H, CH_2, major), 2.39-2.20 (m, 2H, CH_2, minor), 2.03 – 1.94 (m, 2H, major + 2H, CH_2, minor), 1.91-1.83 (m, 1H, CH_2, major), 1.45 – 1.36 (m, 1H, CH_2, major + 1H, CH_2, minor), 1.35 – 1.28 (m, 1H, CH_2, major + 1H, CH_2, minor), 1.24 – 1.19 (m, 1H major + 1H minor, CH), 0.86 (s, C_4H_9, minor, 9H), 0.86 (s, C_4H_9, major, 9H).

^{19}F NMR (377 MHz, $CDCl_3$) δ -151.66 (t, *J* = 18.2 Hz), -165.04 (ddd, *J* = 38.9, 18.3, 3.5 Hz).

^{13}C NMR (151 MHz, $CDCl_3$) δ 204.6 (d, *J* = 19.8 Hz, minor), 203.9 (d, *J* = 20.8 Hz, major), 168.0 (d, *J* = 25.3 Hz, major), 167.7 (d, *J* = 25.5 Hz, minor), 99.1 (d, *J* = 198.8 Hz, minor), 98.6 (d, *J* = 194.6 Hz, major), 53.3 (OMe, minor), 53.2 (OMe, major), 51.6 (CH, minor), 48.7 (CH, major), 39.7 (CH2, minor), 38.5 (CH2, major), 34.3 (d, *J* = 22.8 Hz, CH2, minor), 33.8 (Cq, major), 33.7 (Cq, minor), 33.2 (d, *J* = 22.3 Hz, CH2, major), 27.8 (minor), 27.7 (C4H9, minor), 27.4 (C4H9, major), 25.3 (major), 25.1 (d, *J* = 6.5 Hz, major), 24.8 (minor).

HRMS (Sicrit plasma/LTQ-Orbitrap) m/z: $[M + H]^+$ Calcd for $C_{13}H_{22}FO_3^+$ 245.1547; Found 245.1546.

IR (ν_{max}, cm^{-1}) 2957 (m), 2870 (w), 1758 (s), 1727 (s), 1469 (w), 1439 (m), 1397 (w), 1368 (m), 1284 (m), 1274 (m), 1235 (m), 1195 (m), 1169 (m), 1076 (w), 1049 (m), 1015 (m), 971 (w), 958 (w), 926 (w), 891 (w), 762 (w), 736 (w).

methyl 1-fluoro-2-oxo-5-phenylcycloheptane-1-carboxylate (2f)

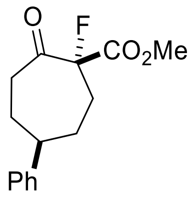

2f

Compound **2f** (dr 5.7:1) (25.5 mg, 96.5 µmol, 48%) was prepared according to general procedure B.

The two diastereoisomers could be separated by prep-TLC purification (1/6 EtOAc/Hexane).

Major dia: **methyl (1S,5R)-1-fluoro-2-oxo-5-phenylcycloheptane-1-carboxylate (2f-1)**

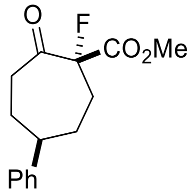

2f-1

Colorless oil.

Rf = 0.40 (1/6 EtOAc/Hexane).

^{1}H NMR (400 MHz, $CDCl_3$) δ 7.34 – 7.28 (m, 2H), 7.25 – 7.21 (m, 1H), 7.20 – 7.16 (m, 2H), 3.86 (s, 3H), 2.99 – 2.83 (m, 2H), 2.83 – 2.75 (m, 1H), 2.67 – 2.54 (m, 1H), 2.17 – 1.88 (m, 5H).

^{19}F NMR (377 MHz, $CDCl_3$) δ -150.72 (t, *J* = 18.9 Hz).

^{13}C NMR (101 MHz, $CDCl_3$) δ 203.7 (d, *J* = 21.4 Hz), 168.0 (d, *J* = 25.2 Hz), 146.0, 128.8, 126.7, 98.6 (d, *J* = 194.3 Hz), 53.3, 45.2, 38.9, 32.4 (d, *J* = 22.2 Hz), 32.3, 31.5 (d, *J* = 6.0 Hz).

HRMS (nanochip-ESI/LTQ-Orbitrap) m/z: $[M + Na]^+$ Calcd for $C_{15}H_{17}FNaO_3^+$ 287.1054; Found 287.1047.

IR (ν_{max}, cm^{-1}) 3029 (w), 2947 (w), 2865 (w), 1753 (m), 1724 (s), 1602 (w), 1493 (w), 1450 (m), 1440 (m), 1338 (w), 1309 (w), 1284 (m), 1226 (m), 1198 (m), 1169 (m), 1130 (w), 1111 (w), 1079 (w), 1055 (w), 1027 (m), 976 (w), 929 (w), 907 (w), 887 (w), 848 (w), 830 (w), 800 (w), 759 (m), 700 (s).

Minor dia: **methyl (1R,5R)-1-fluoro-2-oxo-5-phenylcycloheptane-1-carboxylate (2f-2)**

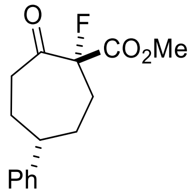

2f-2

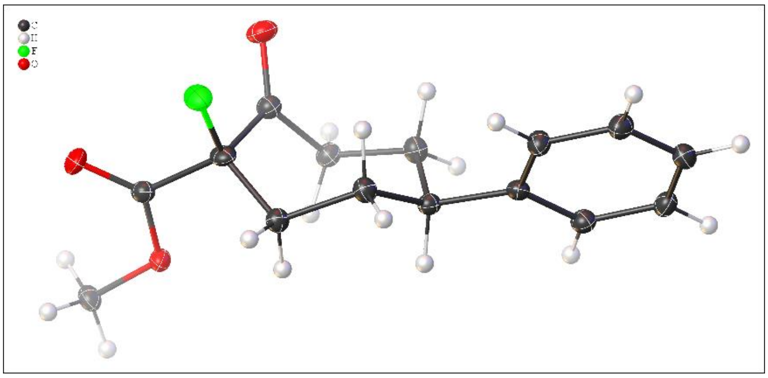

X-Ray structure of 2f-2 (50% ellipsoid probability level)

White crystals.

Rf = 0.30 (1/6 EtOAc/Hexane).

^{1}H NMR (400 MHz, $CDCl_3$) δ 7.33 – 7.27 (m, 2H), 7.25 – 7.18 (m, 1H), 7.18 – 7.13 (m, 2H), 3.85 (s, 3H), 3.04 (td, *J* = 12.6, 3.6 Hz, 1H), 2.80 – 2.70 (m, 2H), 2.60 – 2.41 (m, 2H), 2.20 – 2.10 (m, 1H), 2.04 – 1.96 (m, 1H), 1.94 – 1.80 (m, 2H).

^{19}F NMR (377 MHz, $CDCl_3$) δ -164.80 – -165.01 (m).

^{13}C NMR (101 MHz, $CDCl_3$) δ 204.0 (d, *J* = 19.6 Hz), 167.7 (d, *J* = 25.2 Hz), 146.3, 128.8, 126.8, 126.7, 99.0 (d, *J* = 199.8 Hz), 53.5, 48.2, 40.3, 34.4, 34.2 (d, *J* = 23.0 Hz), 31.2.

HRMS (nanochip-ESI/LTQ-Orbitrap) m/z: $[M + Na]^+$ Calcd for $C_{15}H_{17}FNaO_3^+$ 287.1054; Found 287.1047.

IR (ν_{max}, cm^{-1}) 3026 (w), 2935 (w), 2861 (w), 1765 (s), 1727 (s), 1493 (w), 1450 (m), 1439 (m), 1288 (m), 1267 (m), 1227 (m), 1193 (w), 1169 (m), 1136 (w), 1118 (w), 1063 (w), 1038 (m), 1028 (m), 966 (w), 924 (w), 846 (w), 756 (m), 701 (m), 672 (w).

Mp = 92 – 94 °C.

ethyl 1-fluoro-2-oxocyclopentane-1-carboxylate (2g)

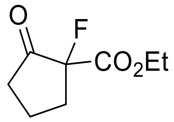

2g

Compound **2g** (22.0 mg, 126.4 µmol, 63%) was prepared according to general procedure B.

The analytical data were in agreement with those reported in the literature.[16]

Light-yellow oil.

Rf = 0.25 (1/4 Et_2O/Hexane)

^{1}H NMR (400 MHz, $CDCl_3$) δ 4.28 (qd, *J* = 7.1, 0.9 Hz, 2H), 2.60 – 2.45 (m, 3H), 2.31 (dddd, *J* = 21.9, 14.3, 7.7, 6.6 Hz, 1H), 2.20 – 2.05 (m, 2H), 1.30 (t, *J* = 7.1 Hz, 3H).

^{19}F NMR (376 MHz, $CDCl_3$) δ -164.08 (dd, *J* = 22.0, 19.5 Hz).

^{13}C NMR (101 MHz, $CDCl_3$) δ 207.7 (d, *J* = 17.1 Hz), 167.6 (d, *J* = 27.1 Hz), 94.8 (d, *J* = 200.2 Hz), 62.5, 35.8, 34.0 (d, *J* = 20.9 Hz), 18.2 (d, *J* = 3.4 Hz), 14.2.

HRMS (APCI/QTOF) m/z: $[M + Na]^+$ Calcd for $C_8H_{11}FNaO_3^+$ 197.0584; Found 197.0589.

IR (ν_{max}, cm^{-1}) 2982 (w), 2941 (w), 2915 (w), 1767 (s), 1752 (s), 1728 (s), 1467 (w), 1448 (w), 1402 (w), 1371 (w), 1313 (m), 1291 (m), 1269 (m), 1162 (s), 1126 (m), 1043 (m), 1020 (s), 964 (w), 914 (w), 857 (w), 819 (w), 770 (w), 650 (w), 617 (w).

methyl 6-fluoro-7-oxospiro[3.4]octane-6-carboxylate (2h)

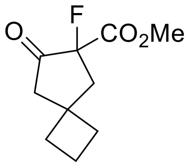

2h

Compound **2h** (20.8 mg, 103.9 µmol, 52%) was prepared according to general procedure B.

Light-yellow oil.

Rf = 0.32 (1/4 Et_2O/Hexane).

^{1}H NMR (400 MHz, $CDCl_3$) δ 3.81 (s, 3H), 2.71 (dd, *J* = 18.6, 1.8 Hz, 1H), 2.63 – 2.42 (m, 3H), 2.19 – 2.07 (m, 3H), 2.07 – 1.89 (m, 3H).

^{19}F NMR (377 MHz, $CDCl_3$) δ -161.06 (ddd, *J* = 26.5, 22.8, 2.9 Hz).

^{13}C NMR (101 MHz, $CDCl_3$) δ 206.6 (d, *J* = 17.0 Hz), 168.1 (d, *J* = 26.9 Hz), 95.8 (d, *J* = 199.9 Hz), 53.2, 50.3, 46.3 (d, *J* = 20.0 Hz), 40.3, 34.6, 32.9, 16.1.

HRMS (ESI/QTOF) m/z: $[M + Na]^+$ Calcd for $C_{10}H_{13}FNaO_3^+$ 223.0741; Found 223.0740.

IR (ν_{max}, cm^{-1}) 2957 (w), 2935 (w), 2854 (w), 1767 (s), 1734 (s), 1437 (m), 1398 (w), 1310 (m), 1270 (m), 1234 (m), 1196 (m), 1160 (m), 1125 (m), 1091 (m), 1073 (m), 1020 (m), 992 (w), 916 (w), 876 (w), 796 (w), 741 (w), 668 (w).

methyl 4-benzyl-1-fluoro-2-oxocyclopentane-1-carboxylate (2i)

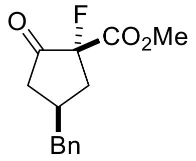

2i

Compound **2i** (dr 2.6:1) (22.0 mg, 87.9 µmol, 44%) was prepared according to general procedure B.

Colorless oil.

Rf = 0.42 (1/3 Et_2O/Hexane).

^{1}H NMR (400 MHz, $CDCl_3$) δ 7.31 (tt, *J* = 8.2, 1.7 Hz, 2H), 7.26 – 7.21 (m, 1H), 7.19 – 7.15 (m, 2H), 3.83 (s, 2.17H), 3.81 (s, 0.83H), 2.92 – 2.59 (m, 4.44H), 2.46 (dddd, *J* = 20.9, 14.8, 6.0, 2.6 Hz, 0.56H), 2.29 – 2.10 (m, 1.73H), 2.09 – 1.96 (m, 0.27H).

^{19}F NMR (377 MHz, $CDCl_3$) δ -159.10 (dd, *J* = 23.3, 9.0 Hz, minor), -162.72 (ddd, *J* = 35.7, 20.9, 3.9 Hz, major).

^{13}C NMR (101 MHz, $CDCl_3$) δ 206.9 (d, *J* = 17.2 Hz, minor), 205.7 (d, *J* = 16.7 Hz, major), 168.1 (d, *J* = 26.2 Hz, major), 167.9 (d, *J* = 27.6 Hz, minor), 138.9, 129.0, 128.8, 126.8 (major), 126.8 (minor), 95.7 (d, *J* = 204.2 Hz, minor), 95.4 (d, *J* = 197.8 Hz, major), 53.3 (minor), 53.2 (major), 43.4 (major), 42.3 (minor), 41.7 (minor), 41.3 (major), 40.3 (d, *J* = 21.8 Hz, major), 39.6 (d, *J* = 19.2 Hz, minor), 35.1 (major), 33.3 (d, *J* = 4.2 Hz, minor).

HRMS (Sicrit plasma/LTQ-Orbitrap) m/z: $[M + H]^+$ Calcd for $C_{14}H_{16}FO_3^+$ 251.1078; Found 251.1078.

IR (ν_{max}, cm^{-1}) 3029 (w), 2956 (w), 2920 (w), 2853 (w), 1769 (s), 1733 (s), 1496 (w), 1439 (m), 1401 (w), 1281 (m), 1243 (w), 1193 (m), 1157 (m), 1130 (m), 1091 (m), 1033 (m), 1001 (m), 914 (w), 854 (w), 794 (w), 752 (m), 702 (s), 670 (w).

ethyl 1-fluoro-2-oxocyclohexane-1-carboxylate (2j)

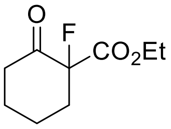

2j

Compound **2j** (26.7 mg, 141.9 µmol, 71%) was prepared according to general procedure B.

Compound **2j** (106.8 mg, 567.5 µmol, 57%) was prepared according to general procedure B.

The analytical data were in agreement with those reported in the literature.[16]

Light-yellow oil.

Rf = 0.25 (1/4 Et_2O/Hexane).

^{1}H NMR (400 MHz, $CDCl_3$) δ 4.29 (q, *J* = 7.1 Hz, 2H), 2.77 – 2.67 (m, 1H), 2.64 – 2.55 (m, 1H), 2.53 – 2.38 (m, 1H), 2.21 – 2.07 (m, 1H), 1.98 – 1.79 (m, 4H), 1.32 (t, *J* = 7.1 Hz, 3H).

^{19}F NMR (377 MHz, $CDCl_3$) δ -160.82 (ddd, *J* = 20.3, 13.6, 5.2 Hz).

^{13}C NMR (101 MHz, $CDCl_3$) δ 202.0 (d, *J* = 19.9 Hz), 167.1 (d, *J* = 24.7 Hz), 96.5 (d, *J* = 196.7 Hz), 62.5, 39.7, 36.1 (d, *J* = 21.6 Hz), 26.7, 21.1 (d, *J* = 5.9 Hz), 14.2.

HRMS (ESI/QTOF) m/z: $[M + Na]^+$ Calcd for $C_9H_{13}FNaO_3^+$ 211.0741; Found 211.0743.
IR (ν_{max}, cm^{-1}) 2944 (w), 2871 (w), 1753 (m), 1729 (s), 1451 (w), 1370 (w), 1342 (w), 1285 (m), 1218 (m), 1152 (m), 1095 (m), 1070 (m), 1054 (m), 1016 (m), 994 (w), 949 (w), 908 (w), 859 (m), 829 (w), 810 (w), 773 (w), 718 (w), 673 (w).

methyl (3aR,7aS)-5-fluoro-6-oxooctahydro-1H-indene-5-carboxylate (2k)

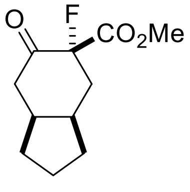

2k

Compound **2k** (dr 2.4:1) (20.4 mg, 95.2 µmol, 48%) was prepared according to general procedure B.

The two diastereoisomers could be separated by prep-TLC purification (1/4 Et_2O/Hexane).

Major dia: **methyl (3aR,5R,7aS)-5-fluoro-6-oxooctahydro-1H-indene-5-carboxylate (2k-1)**

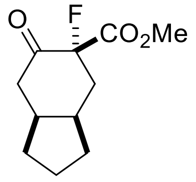

2k-1

Colorless oil.

Rf = 0.40 (1/4 Et_2O/Hexane).

^{1}H NMR (400 MHz, $CDCl_3$) δ 3.82 (s, 3H), 2.93 (tdd, *J* = 8.4, 5.0, 2.9 Hz, 1H), 2.55 – 2.38 (m, 3H), 2.36 – 2.15 (m, 2H), 1.97 – 1.84 (m, 2H), 1.84 – 1.73 (m, 1H), 1.70 – 1.59 (m, 1H), 1.46 – 1.35 (m, 2H).

^{19}F NMR (377 MHz, $CDCl_3$) δ -165.41 – -165.63 (m).

^{13}C NMR (101 MHz, $CDCl_3$) δ 203.2 (d, *J* = 20.7 Hz), 167.9 (d, *J* = 25.2 Hz), 94.9 (d, *J* = 194.5 Hz), 53.1, 40.6, 40.3, 37.0 (d, *J* = 22.3 Hz), 33.0 (d, *J* = 1.5 Hz), 31.6, 31.0, 23.6.

HRMS (nanochip-ESI/LTQ-Orbitrap) m/z: $[M + Na]^+$ Calcd for $C_{11}H_{15}FNaO_3^+$ 237.0897; Found 237.0902.

IR (ν_{max}, cm^{-1}) 2954 (m), 2871 (w), 1766 (s), 1730 (s), 1452 (w), 1437 (w), 1336 (w), 1283 (m), 1243 (m), 1193 (w), 1163 (w), 1127 (w), 1075 (m), 1050 (w), 996 (w), 965 (w), 944 (w), 798 (w), 671 (w).

Minor dia: **methyl (3aR,5S,7aS)-5-fluoro-6-oxooctahydro-1H-indene-5-carboxylate (2k-2)**

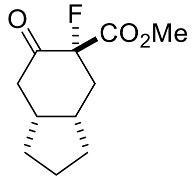

2k-2

Colorless oil.

Rf = 0.30 (1/4 Et_2O/Hexane).

^{1}H NMR (400 MHz, $CDCl_3$) δ 3.84 (s, 3H), 2.92 (dd, *J* = 14.8, 6.7 Hz, 1H), 2.61 – 2.40 (m, 4H), 1.96 (td, *J* = 14.1, 10.9 Hz, 1H), 1.91 – 1.73 (m, 3H), 1.65 (tdd, *J* = 12.9, 5.7, 3.0 Hz, 1H), 1.61 – 1.47 (m, 1H), 1.42 – 1.32 (m, 1H).

^{19}F NMR (377 MHz, $CDCl_3$) δ -158.26 (ddd, *J* = 14.0, 9.5, 3.8 Hz).

^{13}C NMR (101 MHz, $CDCl_3$) δ 202.3 (d, *J* = 16.8 Hz), 167.5 (d, *J* = 25.5 Hz), 95.5 (d, *J* = 200.1 Hz), 53.4, 41.3, 40.9, 37.3 (d, *J* = 19.3 Hz), 36.2 (d, *J* = 7.0 Hz), 31.2, 30.1, 22.6.

HRMS (nanochip-ESI/LTQ-Orbitrap) m/z: $[M + Na]^+$ Calcd for $C_{11}H_{15}FNaO_3^+$ 237.0897; Found 237.0902.

IR (ν_{max}, cm^{-1}) 2954 (w), 2872 (w), 1758 (m), 1734 (s), 1452 (w), 1437 (w), 1363 (w), 1303 (w), 1288 (w), 1245 (w), 1219 (w), 1155 (w), 1102 (w), 1085 (w), 1054 (w), 1029 (w), 994 (w), 965 (w), 886 (w), 809 (w), 672 (w).

Compound 2k-2

^{19}F NMR (377 MHz, $CDCl_3$)
δ -158.26 (ddd, *J* = 13.9, 9.5, 3.9 Hz).
^{3}J(Ha-Fe) and ^{3}J(He-Fe): 13.9 and 9.5 Hz
^{4}J(H-Fe) = 3.9 Hz

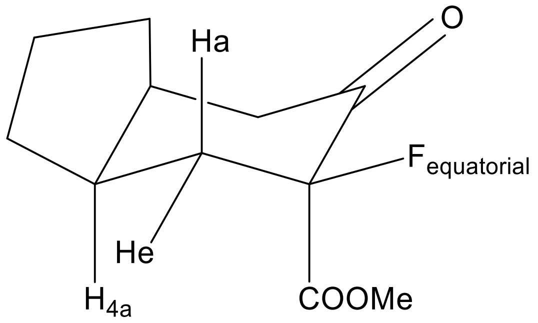

37.27 (d, *J* = 19.3 Hz)
He: 2.53 – 2.41 (m, 1H)
Ha: 1.96 (td, *J* = 14.1, 10.9 Hz, 1H)
^{3}J(Ha-Fe), ^{2}J(Ha-He), ^{3}J(Ha-H_{4a}) = 14.1, 14.1, 10.9 Hz
F is equatorial, H_{4a} is axial

methyl 1-fluoro-2-oxocyclooctane-1-carboxylate (2l)

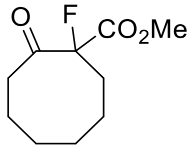

2l

Compound **2l** (19.6 mg, 96.9 µmol, 48%) was prepared according to general procedure B.

Colorless oil.

Rf = 0.31 (1/4 Et_2O/Hexane).

^{1}H NMR (400 MHz, $CDCl_3$) δ 3.80 (s, 3H), 2.77 – 2.47 (m, 3H), 2.31 – 2.20 (m, 1H), 2.08 – 1.95 (m, 1H), 1.92 – 1.82 (m, 1H), 1.81 – 1.34 (m, 6H).

^{19}F NMR (377 MHz, $CDCl_3$) δ -171.42 (dd, *J* = 38.6, 4.1 Hz).

^{13}C NMR (101 MHz, $CDCl_3$) δ 208.9 (d, *J* = 22.0 Hz), 167.7 (d, *J* = 25.0 Hz), 99.4 (d, *J* = 200.7 Hz), 53.4, 39.0, 33.7 (d, *J* = 22.1 Hz), 27.5 (d, *J* = 2.6 Hz), 26.7, 24.5, 21.4 (d, *J* = 2.7 Hz).

HRMS (ESI/QTOF) m/z: $[M + Na]^+$ Calcd for $C_{10}H_{15}FNaO_3^+$ 225.0897; Found 225.0895.

IR (ν_{max}, cm^{-1}) 2930 (m), 2860 (w), 1758 (s), 1721 (s), 1631 (w), 1437 (m), 1363 (w), 1282 (s), 1265 (s), 1224 (s), 1196 (s), 1171 (s), 1057 (m), 1016 (m), 942 (m), 898 (w), 852 (w), 804 (w), 723 (w), 664 (w).

1-fluoro-2-oxo-N-propylcyclooctane-1-carboxamide (2m)

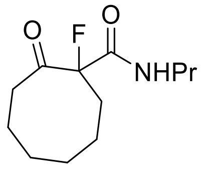

2m

Compound **2m** (14.7 mg, 0.64 mmol, 32%) was prepared according to general procedure B.

Colorless oil.

Rf = 0.65 (1/2 EtOAc/Hexane).

^{1}**H NMR** (400 MHz, $CDCl_3$) δ 6.44 (s, 1H), 3.27 – 3.12 (m, 3H), 2.98 (dddd, *J* = 42.2, 15.8, 12.9, 4.5 Hz, 1H), 2.35 (ddt, *J* = 12.6, 6.1, 3.5 Hz, 1H), 2.15 – 2.06 (m, 1H), 1.99 – 1.90 (m, 1H), 1.85 – 1.67 (m, 4H), 1.58 – 1.46 (m, 3H), 1.46 – 1.35 (m, 1H), 1.15 – 1.03 (m, 1H), 0.89 (t, *J* = 7.4 Hz, 3H).

^{19}**F NMR** (377 MHz, $CDCl_3$) δ -173.81 (dt, *J* = 42.4, 6.9 Hz).

^{13}**C NMR** (101 MHz, $CDCl_3$) δ 209.0 (d, *J* = 17.4 Hz), 166.9 (d, *J* = 20.8 Hz), 101.3 (d, *J* = 201.9 Hz), 41.2, 38.5, 31.5 (d, *J* = 22.0 Hz), 29.8, 25.8, 24.3, 22.7, 22.2 (d, *J* = 3.0 Hz), 11.3.

HRMS (ESI/QTOF) m/z: $[M + Na]^+$ Calcd for $C_{12}H_{20}FNNaO_2^+$ 252.1370; Found 252.1374.

IR (ν_{max}, cm^{-1}) 3351 (w), 2930 (m), 2871 (w), 1718 (s), 1662 (s), 1528 (s), 1465 (m), 1447 (m), 1267 (m), 1226 (w), 1202 (w), 1151 (w), 1120 (m), 1079 (w), 1041 (w), 1024 (w), 1009 (w), 993 (w), 927 (w), 864 (w), 661 (m).

Mp = 39 – 41 °C.

2-acetyl-2-fluorocycloheptan-1-one (2n)

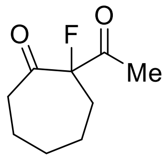

2n

Compound **2n** (20.1 mg, 116.7 µmol, 58%) (volatile product) was prepared according to general procedure B.

Colorless oil.

Rf = 0.50 (1/4 Et_2O/Hexane).

^{1}**H NMR** (400 MHz, $CDCl_3$) δ 2.80 (td, *J* = 12.1, 3.4 Hz, 1H), 2.56 – 2.48 (m, 1H), 2.39 – 2.21 (m, 1H), 2.28 (d, *J* = 5.4 Hz, 3H), 2.18 – 2.06 (m, 1H), 2.04 – 1.81 (m, 3H), 1.54 – 1.40 (m, 3H).

^{19}**F NMR** (377 MHz, $CDCl_3$) δ -160.80 – -161.03 (m).

^{13}**C NMR** (101 MHz, $CDCl_3$) δ 204.7 (d, *J* = 30.3 Hz), 204.5 (d, *J* = 18.6 Hz), 106.1 (d, *J* = 196.5 Hz), 40.7, 33.4 (d, *J* = 22.6 Hz), 29.8, 26.8, 25.9, 23.8.

HRMS (Sicrit plasma/LTQ-Orbitrap) m/z: $[M + H]^+$ Calcd for $C_9H_{14}FO_2^+$ 173.0972; Found 173.0972.

IR (ν_{max}, cm^{-1}) 2932 (m), 2861 (w), 1730 (m), 1714 (s), 1454 (w), 1420 (w), 1358 (m), 1322 (w), 1218 (w), 1195 (w), 1171 (w), 1148 (w), 1065 (w), 1032 (w), 1019 (w), 949 (w), 913 (w), 882 (w), 840 (w), 822 (w), 792 (w), 745 (w), 678 (w), 669 (w), 648 (w).

2-fluoro-2-pentanoylcycloheptan-1-one (2o)

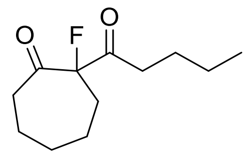

2o

Compound **2o** (21.5 mg, 100.3 µmol, 50%) was prepared according to general procedure B.

Colorless oil.

Rf = 0.30 (1/8 EtOAc/Hexane).

^{1}H NMR (400 MHz, $CDCl_3$) δ 2.83 (td, *J* = 12.0, 3.3 Hz, 1H), 2.64 (tt, *J* = 7.2, 3.6 Hz, 2H), 2.50 (ddq, *J* = 12.0, 6.5, 2.8 Hz, 1H), 2.42 – 2.21 (m, 1H), 2.18 – 2.05 (m, 1H), 2.04 – 1.82 (m, 2H), 1.70 – 1.39 (m, 6H), 1.29 (sext, *J* = 7.3 Hz, 2H), 0.89 (t, *J* = 7.3 Hz, 3H).

^{19}F NMR (377 MHz, $CDCl_3$) δ -163.27 (ddq, *J* = 40.2, 18.0, 3.7 Hz).

^{13}C NMR (101 MHz, $CDCl_3$) δ 206.8 (d, *J* = 28.7 Hz), 204.7 (d, *J* = 18.2 Hz), 106.3 (d, *J* = 197.0 Hz), 40.7, 37.7, 33.8 (d, *J* = 22.7 Hz), 29.9, 26.9, 24.9 (d, *J* = 2.3 Hz), 23.9, 22.3, 13.9.

HRMS (ESI/QTOF) m/z: $[M + Na]^+$ Calcd for $C_{12}H_{19}FNaO_2^+$ 237.1261; Found 237.1262.

IR (ν_{max}, cm^{-1}) 2956 (m), 2933 (m), 2864 (w), 1731 (m), 1712 (s), 1452 (m), 1402 (w), 1368 (w), 1322 (w), 1235 (w), 1175 (w), 1147 (w), 1059 (w), 1020 (w), 950 (w), 882 (w), 837 (w), 794 (w), 753 (w), 672 (w).

2-acetyl-5-(tert-butyl)-2-fluorocycloheptan-1-one (2p)

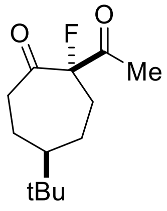

2p

Compound **2p** (dr 3.7:1) (23.0 mg, 100.7 µmol, 50%) was prepared according to general procedure B.

Colorless oil.

Rf = 0.54 (1/6 EtOAc/Hexane).

^{1}H NMR (400 MHz, $CDCl_3$) δ 2.86 – 2.72 (m, 1H), 2.67-2.28 (m, 2H) (0.8+0.8+0.2+02), 2.27 (d, *J* = 3.8 Hz, 0.6H), 2.26 (d, *J* = 5.2 Hz, 2.4H), 2.24 – 2.02 (m, 0.4H), 1.99 – 1.87 (m, 1.6 + 0.2H), 1.79 (dddd, *J* = 17.8, 15.0, 8.5, 3.8 Hz, 0.8H), 1.56 – 1.44 (m, 0.8H), 1.40 – 1.08 (m, 1 + 0.8 + 0.4H), 0.86 (s, 2H), 0.85 (s, 7H).

^{19}F NMR (377 MHz, $CDCl_3$) δ -153.15 (ddt, *J* = 23.3, 17.7, 5.0 Hz, major), -161.73 (ddt, *J* = 41.0, 18.8, 5.1 Hz, minor).

^{13}C NMR (101 MHz, $CDCl_3$) δ 204.9 (d, *J* = 30.4 Hz) (major), 204.8 (d, *J* = 19.8 Hz) (major), 204.8 (d, *J* = 18.0 Hz) (minor), 204.4 (d, *J* = 30.5 Hz) (minor), 106.5 (d, *J* = 196.9 Hz) (minor), 104.0 (d, *J* = 194.3 Hz) (major), 52.1 (minor), 47.3 (major), 39.4 (minor), 38.1 (major), 33.8 (major), 33.6 (minor), 33.4 (d, *J* = 22.4 Hz) (minor), 32.2 (d, *J* = 22.1 Hz) (major), 28.5 (minor), 27.8 (minor), 27.3 (major), 26.1 (major), 25.9 (d, *J* = 1.4 Hz) (minor), 24.8 (minor), 24.5 (d, *J* = 4.6 Hz) (major), 24.2 (major).

HRMS (Sicrit plasma/LTQ-Orbitrap) m/z: $[M + H]^+$ Calcd for $C_{13}H_{22}FO_2^+$ 229.1598; Found 229.1597.

IR (ν_{max}, cm^{-1}) 2961 (m), 2870 (w), 1730 (m), 1713 (s), 1472 (w), 1445 (w), 1420 (w), 1397 (w), 1366 (m), 1357 (m), 1215 (m), 1183 (w), 1146 (w), 1116 (w), 1073 (w), 1046 (m), 1008 (w), 964 (w), 953 (w), 924 (w), 824 (w), 801 (w), 730 (w).

1-fluoro-2-oxo-N-propylcycloheptane-1-carboxamide (2q)

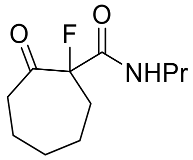

2q

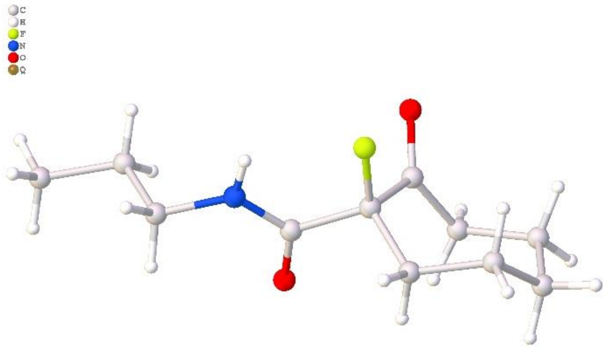

X-Ray structure of 2q (50% ellipsoid probability level)

Compound **2q** (21.8 mg, 101.2 µmol, 51%) was prepared according to general procedure B. **2q** was dissolved in hot hexane, then recrystallized by keeping the sample in freezer (-35 °C) for 48 h before removing hexane directly at low temperature to afford white crystals.

White crystals.

Rf = 0.62 (1/1 EtOAc/Hexane).

^{1}H NMR (400 MHz, $CDCl_3$) δ 6.47 (brs, 1H), 3.30 – 3.13 (m, 2H), 3.12 – 3.03 (m, 1H), 2.63 – 2.43 (m, 2H), 2.23 – 2.09 (m, 1H), 2.07 – 1.97 (m, 1H), 1.97 – 1.84 (m, 2H), 1.73 – 1.31 (m, 5H), 0.90 (t, *J* = 7.4 Hz, 3H).

^{19}F NMR (377 MHz, $CDCl_3$) δ -161.97 (ddt, *J* = 42.0, 19.6, 4.7 Hz).

^{13}C NMR (101 MHz, $CDCl_3$) δ 205.2 (d, *J* = 17.2 Hz), 166.9 (d, *J* = 20.9 Hz), 101.6 (d, *J* = 199.3 Hz), 41.3, 41.3, 34.1 (d, *J* = 22.5 Hz), 30.2, 27.5, 23.9, 22.7, 11.3.

HRMS (ESI/QTOF) m/z: $[M + H]^+$ Calcd for $C_{11}H_{19}FNO_2^+$ 216.1394; Found 216.1403.

IR (ν_{max}, cm^{-1}) 3359 (w), 2932 (m), 2861 (w), 1727 (s), 1662 (s), 1532 (s), 1450 (m), 1368 (m), 1320 (w), 1290 (w), 1264 (w), 1234 (m), 1205 (m), 1169 (m), 1149 (m), 1083 (w), 1060 (w), 1023 (m), 983 (w), 944 (w), 888 (w), 845 (w), 784 (w).

Mp = 45 – 47 °C.

1-fluoro-2-oxo-5-phenyl-N-propylcycloheptane-1-carboxamide (2r)

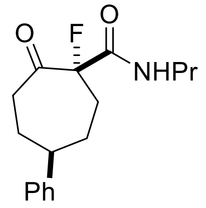

2r

Compound **2r** (dr 5.3:1) (28.6 mg, 98.2 μmol, 49%) was prepared according to general procedure B at 30 °C for 12 h with MeCN/H_2O (8/1, 0.5 M).

Colorless oil.

Rf = 0.56 (1/3 EtOAc/Hexane).

^{1}H NMR (400 MHz, $CDCl_3$) δ 7.34 – 7.26 (m, 2H), 7.25 – 7.14 (m, 3H), 6.55 – 6.46 (m, 0.16H), 6.45 (d, *J* = 6.5 Hz, 0.84H), 3.30 (ddd, *J* = 14.5, 11.9, 2.8 Hz, 0.16H), 3.27 – 3.20 (m, 2H), 3.17 (td, *J* = 8.3, 4.2 Hz, 0.84H), 2.95 (tt, *J* = 8.5, 3.8 Hz, 0.84H), 2.90 – 2.76 (m, 0.32H), 2.76 – 2.54 (m, 1.68+0.16H), 2.39 – 2.13 (m, 2H), 2.08 – 1.94 (m, 2.68H), 1.85 – 1.66 (m, 0.32H), 1.55 (h, *J* = 7.4 Hz, 2H), 0.92 (t, *J* = 7.4 Hz, 3H).

^{19}F NMR (377 MHz, $CDCl_3$) δ -155.10 (dd, *J* = 28.8, 17.0 Hz, major), -162.48 (ddt, *J* = 42.4, 19.6, 4.6 Hz, minor).

^{13}C NMR (101 MHz, $CDCl_3$) δ 205.1 (d, *J* = 17.1 Hz, minor), 204.6 (d, *J* = 18.3 Hz, major), 167.3 (d, *J* = 21.3 Hz, major), 166.6 (d, *J* = 21.0 Hz, minor), 146.2 (minor), 145.4 (major), 128.8 (major), 128.8 (minor), 127.0 (major), 126.7 (minor), 126.7 (minor), 126.5 (major), 101.8 (d, *J* = 200.0 Hz, minor), 100.2 (d, *J* = 197.3 Hz, major), 48.8 (minor), 43.2 (major), 41.3 (minor), 41.2 (major), 40.4 (minor), 39.0 (major), 35.6 (minor), 34.0 (d, *J* = 22.5 Hz, minor), 31.6 (d, *J* = 22.2 Hz, major), 31.2 (minor), 30.4 (d, *J* = 4.1 Hz, major), 30.2 (major), 22.8 (major), 22.7 (minor), 11.3 (major + minor).

HRMS (ESI/QTOF) m/z: $[M + Na]^+$ Calcd for $C_{17}H_{22}FNNaO_2^+$ 314.1527; Found 314.1532.

IR (ν_{max}, cm^{-1}) 3357 (w), 2964 (w), 2933 (w), 2872 (w), 1725 (s), 1666 (s), 1530 (s), 1495 (w), 1452 (m), 1380 (w), 1344 (w), 1274 (w), 1228 (w), 1179 (w), 1150 (w), 1120 (w), 1080 (w), 1031 (m), 951 (w), 922 (w), 759 (m), 736 (w), 701 (s), 672 (m).

methyl 1-fluoro-2-hexyl-5-oxocyclopentane-1-carboxylate (2u)

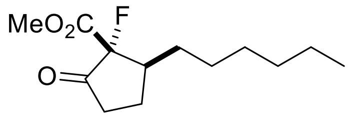

2u

Compound **2u** (dr 2.6:1, rr > 20:1) (14.6 mg, 87.9 μmol, 30%) was prepared according to general procedure B at 30 °C for 48 h.

Colorless oil.

Rf = 0.37 (1/4 Et_2O/Hexane).

^{1}H NMR (400 MHz, $CDCl_3$) δ 3.83 (s, 0.84H), 3.81 (s, 2.16H), 2.66 – 2.52 (m, 1.28H), 2.47 – 2.31 (m, 1.72H), 2.25 – 2.14 (m, 1H), 1.88 – 1.72 (m, 1H), 1.66 – 1.57 (m, 1H), 1.42 – 1.21 (m, 9H), 0.90 – 0.85 (m, 3H).

^{19}F NMR (377 MHz, $CDCl_3$) δ -166.84 (d, *J* = 22.2 Hz, major), -180.84 (dd, *J* = 28.5, 3.7 Hz, minor).

^{13}C NMR (101 MHz, $CDCl_3$) δ 208.4 (d, *J* = 17.1 Hz, major), 207.3 (d, *J* = 17.0 Hz, minor), 168.5 (d, *J* = 26.3 Hz, minor), 166.7 (d, *J* = 28.9 Hz, major), 99.1 (d, *J* = 206.6 Hz, major), 96.0 (d, *J* = 199.7 Hz, minor), 53.1 (minor), 52.8 (major), 46.4 (d, *J* = 18.4 Hz, major), 45.5 (d, *J* = 20.6 Hz, minor), 36.5 (minor), 35.3 (d, *J* = 1.5 Hz, major), 31.7 (minor), 31.7 (major), 30.1 (major), 29.4 (minor), 29.3 (major), 27.8 (d, *J* = 6.4 Hz, minor), 27.1 (minor), 27.1 (major), 25.4 (minor), 23.2 (d, *J* = 9.0 Hz, major), 22.7 (major + minor), 14.2 (minor), 14.2 (major).

HRMS (APCI/QTOF) m/z: $[M + Na]^+$ Calcd for $C_{13}H_{21}FNaO_3^+$ 267.1367; Found 267.1373.

IR (ν_{max}, cm^{-1}) 2954 (m), 2928 (m), 2858 (w), 1771 (s), 1759 (s), 1730 (m), 1460 (w), 1438 (w), 1321 (w), 1279 (m), 1244 (m), 1193 (m), 1160 (m), 1141 (m), 1096 (w), 1046 (m), 1003 (w), 966 (w), 917 (w), 823 (w), 782 (w), 725 (w).

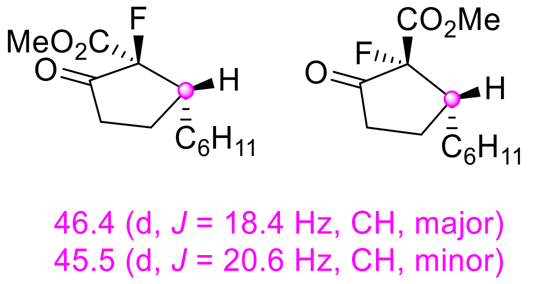

methyl 1-fluoro-2-(3-methoxypropyl)-5-oxocyclopentane-1-carboxylate (2v)

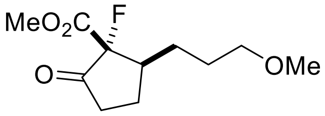

2v

Compounds **2v** (dr 2:1, rr > 10:1) (23.2 mg, 49.9 µmol, 50%) was prepared according to general procedure B.

Colorless oil.

Rf = 0.33 (1/1 Et_2O/Hexane).

^{1}H NMR (500 MHz, $CDCl_3$) δ 3.82 (s, 1H), 3.81 (s, 2H), 3.41 – 3.33 (m, 2H), 3.31 (s, 1H), 3.31 (s, 2H), 2.66 – 2.35 (m, 3H), 2.26 – 2.15 (m, 1H), 1.90 – 1.41 (m, 5H).

^{19}F NMR (377 MHz, $CDCl_3$) δ -166.58 (d, *J* = 21.7 Hz, major), -180.73 (dd, *J* = 28.1, 3.6 Hz, minor).

^{13}C NMR (126 MHz, $CDCl_3$) δ 208.2 (d, *J* = 17.0 Hz, major), 207.1 (d, *J* = 16.9 Hz, minor), 168.4 (d, *J* = 26.3 Hz, minor), 166.5 (d, *J* = 28.8 Hz, major), 99.0 (d, *J* = 206.5 Hz, major), 95.8 (d, *J* = 199.9 Hz, minor), 72.5 (minor), 72.3 (major), 58.8 (minor), 58.7 (major), 53.1 (minor), 52.8 (major), 46.2 (d, *J* = 18.5 Hz, major), 45.3 (d, *J* = 20.3 Hz, minor), 36.4 (minor), 35.2 (d, *J* = 1.6 Hz, major), 27.3 (minor), 27.2 (major), 27.0 (d, *J* = 0.9 Hz, major), 25.4 (minor), 24.7 (d, *J* = 6.9 Hz, minor), 23.2 (d, *J* = 9.0 Hz, major).

HRMS (ESI/QTOF) m/z: $[M + Na]^+$ Calcd for $C_{11}H_{17}FNaO_4^+$ 255.1003; Found 255.0994.

IR (ν_{max}, cm^{-1}) 2939 (w), 2870 (w), 1769 (s), 1755 (s), 1729 (s), 1455 (w), 1438 (m), 1325 (w), 1279 (m), 1191 (m), 1162 (m), 1116 (s), 1040 (m), 955 (w), 917 (w), 865 (w), 782 (w).

methyl 1-fluoro-2-oxooctahydro-1H-indene-1-carboxylate (2w)

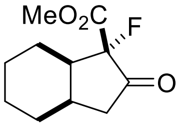

2w

Compounds **2w** (dr 9.5:1, rr > 10:1) (13.7 mg, 63.9 µmol, 32%) was prepared according to general procedure B.

Colorless oil.

Rf = 0.29 (1/4 Et_2O/Hexane).

^{1}H NMR (400 MHz, $CDCl_3$) δ 3.81 (s, 3H), 2.80 – 2.70 (m, 1H), 2.55 – 2.37 (m, 3H), 1.82 – 1.74 (m, 1H), 1.72 – 1.62 (m, 2H), 1.59 – 1.16 (m, 5H).

^{19}F NMR (377 MHz, $CDCl_3$) δ -157.93 (dd, *J* = 21.9, 3.5 Hz, major), -159.68 (dd, *J* = 31.5, 24.6 Hz, minor).

^{13}C NMR (101 MHz, $CDCl_3$) δ 207.0 (d, *J* = 16.3 Hz), 167.1 (d, *J* = 26.5 Hz), 98.2 (d, *J* = 194.0 Hz), 52.7, 45.1 (d, *J* = 20.8 Hz), 38.4, 31.5, 26.0, 24.1, 22.4 (d, *J* = 6.0 Hz), 20.1.

HRMS (ESI/QTOF) m/z: $[M + Na]^+$ Calcd for $C_{11}H_{15}FNaO_3^+$ 237.0897; Found 237.0893.

IR (ν_{max}, cm^{-1}) 2934 (m), 2861 (w), 1764 (s), 1735 (s), 1451 (w), 1439 (m), 1301 (m), 1281 (m), 1201 (m), 1130 (w), 1075 (m), 1053 (m), 1037 (m), 1020 (m), 669 (w).

methyl 1-fluoro-5-(2-hydroxyethyl)-2-oxocycloheptane-1-carboxylate (2x)

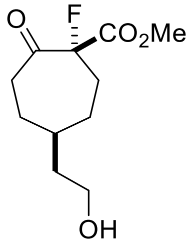

2x

Compound **2x** (dr 3:1) (23.2 mg, 99.9 µmol, 50%) was prepared according to general procedure B.

Light-yellow oil.

Rf = 0.43 (7/3 EtOAc/Hexane).

^{1}H NMR (400 MHz, $CDCl_3$) δ 3.82 (s, 2.25H), 3.81 (s, 0.75H), 3.69 (t, *J* = 6.5 Hz, 2H), 2.91 (td, *J* = 12.6, 3.4 Hz, 0.25H), 2.81 – 2.61 (m, 2H), 2.54 – 2.35 (m, 1H), 2.34 – 2.28 (m, 0.25H), 2.10 – 1.80 (m, 4H), 1.69 – 1.47 (m, 4H), 1.45 – 1.27 (m, 0.5H).

^{19}**F NMR** (377 MHz, $CDCl_3$) δ -157.33 (ddd, *J* = 27.8, 16.4, 2.8 Hz, major), -164.61 – -164.81 (m, minor).

^{13}**C NMR** (151 MHz, $CDCl_3$) δ 204.1 (d, *J* = 19.3 Hz, minor), 204.1 (d, *J* = 21.3 Hz, major), 168.0 (d, *J* = 25.5 Hz, major), 167.7 (d, *J* = 25.5 Hz, minor), 98.8 (d, *J* = 199.2 Hz, minor), 98.7 (d, *J* = 196.0 Hz, major), 60.8 (major), 60.4 (minor), 53.4 (minor), 53.3 (major), 40.0 (minor), 39.9 (minor), 38.0 (major), 37.8 (minor), 36.7 (major), 33.7 (major), 33.7 (d, *J* = 23.0 Hz, minor), 32.7 (minor), 30.2 (major), 30.1 (d, *J* = 23.0 Hz, major), 29.9 (minor), 28.9 (d, *J* = 3.9 Hz, major).

HRMS (Sicrit plasma/LTQ-Orbitrap) m/z: $[M + H]^+$ Calcd for $C_{11}H_{18}FO_4^+$ 233.1184; Found 233.1182.

IR (ν_{max}, cm^{-1}) 3412 (w), 2929 (m), 2869 (w), 1756 (s), 1723 (s), 1438 (m), 1288 (m), 1259 (m), 1196 (m), 1171 (m), 1134 (m), 1052 (s), 1018 (s), 958 (w), 935 (w), 892 (w), 846 (w), 790 (w), 734 (w).

methyl 1-fluoro-5-(2-hydroxyethyl)-2-oxocycloheptane-1-carboxylate (2y)

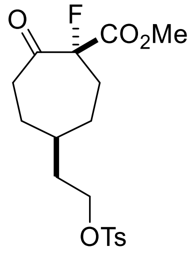

2y

Compound **2y** (dr 3.5:1) (45.5 mg, 117.7 µmol, 59%) was prepared according to general procedure B.

Colorless oil.

Rf = 0.30 (1/2 EtOAc/Hexane).

^{1}**H NMR** (400 MHz, $CDCl_3$) δ 7.80 – 7.76 (m, 2H), 7.38 – 7.33 (m, 2H), 4.05 (t, *J* = 6.3 Hz, 2H), 3.81 (s, 3H), 2.84 (td, *J* = 12.7, 3.4 Hz, 0.22H), 2.66 (td, *J* = 6.6, 2.1 Hz, 1.56H), 2.63 – 2.55 (m, 0.22H), 2.45 (s, 3H), 2.44 – 2.21 (m, 1.22H), 2.03 – 1.41 (m, 7.34H), 1.37 – 1.19 (m, 0.44H).

^{19}**F NMR** (377 MHz, $CDCl_3$) δ -156.75 (dd, *J* = 26.2, 16.1 Hz, major), -164.71 – -164.91 (m, minor).

^{13}**C NMR** (101 MHz, $CDCl_3$) δ 203.6 (d, *J* = 18.9 Hz, minor), 203.6 (d, *J* = 21.5 Hz, major), 167.8 (d, *J* = 25.2 Hz, major), 167.5 (d, *J* = 25.4 Hz, minor), 145.1, 133.0, 130.1, 128.0, 98.7 (d, *J* = 199.4 Hz, minor), 98.5 (d, *J* = 196.4 Hz, major), 68.2 (major), 68.0 (minor), 53.5 (minor), 53.3 (major), 39.7 (minor), 37.8 (major), 37.2 (minor), 36.0 (minor), 33.6 (major), 33.4 (d, *J* = 23.1 Hz, minor), 33.2 (major), 32.2 (minor), 30.1 (d, *J* = 23.2 Hz, major), 29.8 (major), 29.4 (minor), 28.6 (d, *J* = 4.3 Hz, major), 21.8.

HRMS (Sicrit plasma/LTQ-Orbitrap) m/z: $[M + H]^+$ Calcd for $C_{18}H_{24}FO_6S^+$ 387.1272; Found 387.1272.

IR (ν_{max}, cm^{-1}) 2930 (w), 2869 (w), 1761 (w), 1725 (m), 1597 (w), 1448 (w), 1355 (m), 1291 (w), 1259 (w), 1209 (w), 1188 (m), 1173 (s), 1096 (m), 1050 (w), 1019 (w), 957 (m), 917 (m), 839 (w), 816 (m), 764 (m), 734 (m), 663 (s).

methyl 5-(2-chloroethyl)-1-fluoro-2-oxocycloheptane-1-carboxylate (2z)

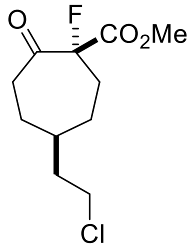

2z

Compound **2z** (dr 3.1:1) (25.9 mg, 103.3 µmol, 52%) was prepared according to general procedure B.

Colorless oil.

Rf = 0.43 (1/4 EtOAc/Hexane).

^{1}**H NMR** (400 MHz, $CDCl_3$) δ 3.83 (s, 2.27H), 3.82 (s, 0.73H), 3.59 – 3.53 (m, 2H), 2.93 (td, *J* = 12.6, 3.5 Hz, 0.24H), 2.77 – 2.70 (m, 1.52H), 2.66 (dtt, *J* = 12.6, 6.4, 2.9 Hz, 0.24H), 2.51 – 2.30 (m, 1.24H), 2.12 – 1.69 (m, 5.76H), 1.66 – 1.52 (m, 1.52H), 1.42 – 1.29 (m, 0.48H).

^{19}**F NMR** (377 MHz, $CDCl_3$) δ -157.00 (dd, *J* = 26.4, 16.1 Hz, major), -164.63 – -164.88 (m, minor).

^{13}**C NMR** (101 MHz, $CDCl_3$) δ 203.8 (d, *J* = 19.4 Hz, minor), 203.7 (d, *J* = 21.4 Hz, major), 167.9 (d, *J* = 25.2 Hz, major), 167.6 (d, *J* = 25.6 Hz, minor), 98.8 (d, *J* = 199.4 Hz, minor), 98.6 (d, *J* = 196.4 Hz, major), 53.5 (minor), 53.3 (major), 42.8 (major), 42.6 (minor), 39.8 (minor), 39.5 (minor), 38.1 (minor), 37.9 (major), 36.7 (major), 34.5 (major), 33.5 (d, *J* = 23.2 Hz, minor), 32.1 (minor), 30.2 (d, *J* = 23.2 Hz, major), 29.7 (major), 29.4 (minor), 28.4 (d, *J* = 4.2 Hz, major).

HRMS (Sicrit plasma/LTQ-Orbitrap) m/z: $[M + H]^+$ Calcd for $C_{11}H_{17}ClFO_3^+$ 251.0845; Found 251.0844.

IR ($ν_{max}$, cm^{-1}) 2932 (w), 2865 (w), 1755 (s), 1726 (s), 1644 (w), 1441 (m), 1351 (w), 1286 (m), 1252 (m), 1203 (m), 1168 (m), 1136 (w), 1112 (w), 1050 (m), 1020 (m), 956 (w), 933 (w), 885 (w), 847 (w), 787 (w), 753 (w), 719 (w), 653 (m).

methyl 5-(2-bromoethyl)-1-fluoro-2-oxocycloheptane-1-carboxylate (2aa)

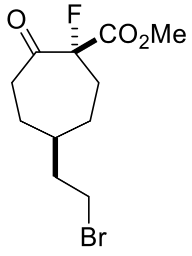

2aa

Compound **2aa** (dr 3.4:1) (27.2 mg, 92.2 µmol, 46%) was prepared according to general procedure B.

Colorless oil.

Rf = 0.47 (1/4 EtOAc/Hexane).

^{1}**H NMR** (400 MHz, $CDCl_3$) δ 3.83 (s, 2.32H), 3.82 (s, 0.68H), 3.41 (t, *J* = 6.9 Hz, 2H), 2.93 (td, *J* = 12.6, 3.4 Hz, 0.23H), 2.73 (ddd, *J* = 8.1, 6.2, 2.1 Hz, 1.54H), 2.66 (dtt, *J* = 12.6, 6.4, 2.9 Hz, 0.23H), 2.51 – 2.30 (m, 1.23H), 2.13 – 1.75 (m, 5.77H), 1.67 – 1.50 (m, 1.54H), 1.43 – 1.29 (m, 0.46H).

^{19}**F NMR** (377 MHz, $CDCl_3$) δ -156.96 (dd, *J* = 26.2, 16.3 Hz, major), -164.66 – -164.87 (m, minor).

^{13}**C NMR** (101 MHz, $CDCl_3$) δ 203.7 (d, *J* = 19.3 Hz, minor), 203.7 (d, *J* = 21.4 Hz, major), 167.9 (d, *J* = 25.2 Hz, major), 167.6 (d, *J* = 25.6 Hz, minor), 98.7 (d, *J* = 199.5 Hz, minor), 98.6 (d, *J* = 196.3 Hz, major), 53.5 (minor), 53.3 (major), 39.8 (minor), 39.6 (minor), 39.4 (minor), 37.9 (major), 36.9 (major), 35.8 (major), 33.5 (d, *J* = 23.0 Hz, minor), 31.9 (minor), 31.3 (minor), 31.3 (major), 30.2 (d, *J* = 23.2 Hz, major), 29.6 (major), 29.2 (minor), 28.3 (d, *J* = 4.2 Hz, major).

HRMS (ESI/QTOF) m/z: $[M + Na]^+$ Calcd for $C_{11}H_{16}BrFNaO_3^+$ 317.0159; Found 317.0165.

IR (ν_{max}, cm^{-1}) 2925 (w), 2855 (w), 1757 (s), 1725 (s), 1642 (w), 1616 (w), 1439 (m), 1346 (w), 1285 (m), 1262 (s), 1240 (s), 1200 (m), 1162 (m), 1130 (w), 1050 (m), 1021 (m), 932 (w), 882 (w), 847 (w), 787 (w), 747 (w).

methyl 5-(2-cyanoethyl)-1-fluoro-2-oxocycloheptane-1-carboxylate (2ab)

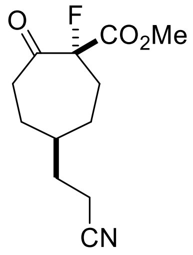

2ab

Compound **2ab** (dr 3:1) (30.9 mg, 128.1 µmol, 64%) was prepared according to general procedure B.

Colorless oil.

Rf = 0.33 (2/3 EtOAc/Hexane).

^{1}**H NMR** (400 MHz, $CDCl_3$) δ 3.82 (s, 2.25H), 3.81 (s, 0.75H), 2.93 (tdd, *J* = 12.7, 3.4, 1.5 Hz, 0.25H), 2.80 – 2.70 (m, 1.5H), 2.70 – 2.62 (m, 0.25H), 2.51 – 2.31 (m, 3.5H), 2.13 – 1.51 (m, 7H), 1.42 – 1.29 (m, 0.5H).

^{19}**F NMR** (377 MHz, $CDCl_3$) δ -156.76 (dd, *J* = 25.6, 16.2 Hz, major), -164.71 – -164.91 (m, minor).

^{13}C NMR (101 MHz, $CDCl_3$) δ 203.4 (d, *J* = 18.8 Hz, minor), 203.4 (d, *J* = 21.7 Hz, major), 167.7 (d, *J* = 25.2 Hz, major), 167.4 (d, *J* = 25.5 Hz, minor), 119.3 (minor), 119.3 (major), 98.6 (d, *J* = 199.5 Hz, minor), 98.4 (d, *J* = 196.5 Hz, major), 53.5 (minor), 53.3 (major), 39.9 (minor), 39.5 (minor), 37.7 (major), 36.4 (major), 33.3 (d, *J* = 23.3 Hz, minor), 32.3 (minor), 31.9 (minor), 30.1 (d, *J* = 23.3 Hz, major), 29.8 (major), 29.5 (major), 29.2 (minor), 28.3 (d, *J* = 4.5 Hz, major), 15.3 (major), 15.1 (minor).

HRMS (ESI/QTOF) m/z: $[M + Na]^+$ Calcd for $C_{12}H_{16}FNNaO_3^+$ 264.1006; Found 264.1016.

IR (ν_{max}, cm^{-1}) 3011 (w), 2935 (w), 2865 (w), 2245 (w), 1757 (s), 1725 (s), 1643 (w), 1438 (m), 1352 (w), 1287 (m), 1258 (m), 1204 (m), 1172 (m), 1123 (m), 1051 (m), 1014 (m), 956 (w), 933 (w), 886 (w), 848 (w), 787 (w), 754 (w).

methyl 5-(2-(1,3-dioxoisoindolin-2-yl)ethyl)-1-fluoro-2-oxocycloheptane-1-carboxylate (2ac)

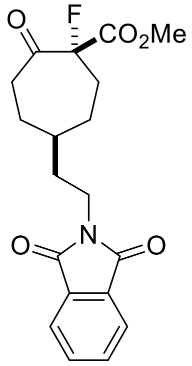

2ac

Compound **2ac** (dr 4:1) (41.2 mg, 114.0 µmol, 57%) was prepared according to general procedure B at 30 °C for 18 h with MeCN/H_2O (8/1, 0.5 M).

Colorless oil.

Rf = 0.44 (1/2 EtOAc/Hexane).

^{1}H NMR (400 MHz, $CDCl_3$) δ 7.86 – 7.81 (m, 2H), 7.75 – 7.69 (m, 2H), 3.82 (s, 2.4H), 3.80 (s, 0.6H), 3.74 – 3.67 (m, 2H), 2.87 (td, *J* = 12.4, 3.6 Hz, 0.2H), 2.80 – 2.62 (m, 1.6H), 2.55 – 2.16 (m, 1.2H), 2.14 – 1.86 (m, 2.6H), 1.81 – 1.55 (m, 5H), 1.52 – 1.30 (m, 0.4H).

^{19}F NMR (377 MHz, $CDCl_3$) δ -156.57 (dd, *J* = 26.8, 16.0 Hz, major), -164.79 – -165.00 (m, minor).

^{13}C NMR (101 MHz, $CDCl_3$) δ 203.9 (d, *J* = 19.5 Hz, minor), 203.8 (d, *J* = 21.3 Hz, major), 168.5 (minor), 168.5 (major), 167.9 (d, *J* = 25.4 Hz, major), 167.7 (d, *J* = 25.6 Hz, minor), 134.2 (major + minor), 132.2 (major + minor), 123.4 (major + minor), 98.9 (d, *J* = 199.2 Hz, minor), 98.7 (d, *J* = 195.9 Hz, major), 53.4 (minor), 53.3 (major), 39.9 (minor), 38.6 (minor), 37.9 (major), 36.0 (major), 35.9 (minor), 35.8 (minor), 34.8 (major), 33.6 (d, *J* = 23.1 Hz, minor), 33.0 (major), 32.3 (minor), 30.2 (d, *J* = 23.2 Hz, major), 30.0 (major), 29.6 (minor), 28.7 (d, *J* = 4.2 Hz, major).

HRMS (ESI/QTOF) m/z: $[M + Na]^+$ Calcd for $C_{19}H_{20}FNNaO_5^+$ 384.1218; Found 384.1219.

IR (ν_{max}, cm^{-1}) 2941 (w), 2864 (w), 1766 (w), 1706 (s), 1614 (w), 1437 (w), 1397 (m), 1372 (m), 1266 (w), 1189 (w), 1172 (w), 1050 (w), 1013 (w), 961 (w), 895 (w), 868 (w), 795 (w), 718 (s).

methyl 1-fluoro-5-(2-(4-methylbenzamido)ethyl)-2-oxocycloheptane-1-carboxylate (2ad)

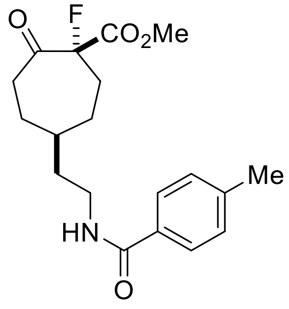

2ad

Compound **2ad** (dr 3.3:1) (33.5 mg, 95.9 µmol, 48%) was prepared according to general procedure B at 30 °C for 24 h with MeCN/H_2O (8/1, 0.5 M).

Colorless oil.

Rf = 0.50 (3/2 EtOAc/Hexane).

^{1}H NMR (400 MHz, $CDCl_3$) δ 7.67 – 7.62 (m, 2H), 7.23 (d, *J* = 7.9 Hz, 2H), 6.23 – 6.03 (m, 1H), 3.82 (s, 2.3H), 3.81 (s, 0.7H), 3.51 – 3.43 (m, 2H), 2.89 (td, *J* = 12.6, 3.6 Hz, 0.23H), 2.81

– 2.61 (m, 1.54H), 2.55 – 2.26 (m, 1.23H), 2.39 (s, 3H), 2.11-1.52 (m, 7.54H), 1.47 – 1.31 (m, 0.46H).

^{19}F NMR (377 MHz, $CDCl_3$) δ -157.54 (dd, *J* = 27.6, 16.0 Hz, major), -164.64 – -164.85 (m, minor).

^{13}C NMR (101 MHz, $CDCl_3$) δ 204.0 (d, *J* = 21.2 Hz), 167.9 (d, *J* = 25.5 Hz, major), 167.7, 167.6 (d, *J* = 25.3 Hz, minor), 142.1, 131.8 (minor), 131.7 (major), 129.4, 126.9, 98.8 (d, *J* = 199.4 Hz, minor), 98.7 (d, *J* = 196.2 Hz, major), 53.4 (minor), 53.3 (major), 39.9 (minor), 39.0 (minor), 38.1 (major), 37.9 (major), 37.9 (minor), 37.4 (minor), 34.8 (major), 34.1 (major), 33.6 (d, *J* = 23.3 Hz, minor), 32.6 (minor), 30.0 (d, *J* = 23.1 Hz, major), 30.0 (major), 29.8 (minor), 28.7 (d, *J* = 3.8 Hz, major), 21.6.

HRMS (APCI/QTOF) m/z: $[M + Na]^+$ Calcd for $C_{19}H_{24}FNNaO_4^+$ 372.1582; Found 372.1578.

IR (ν_{max}, cm^{-1}) 3327 (w), 2929 (w), 2866 (w), 1726 (s), 1635 (m), 1542 (m), 1504 (m), 1438 (m), 1369 (w), 1286 (m), 1251 (m), 1203 (m), 1170 (m), 1139 (w), 1050 (m), 1018 (m), 912 (m), 838 (w), 730 (s), 672 (m).

methyl 5-(2-((4-bromobenzoyl)oxy)ethyl)-1-fluoro-2-oxocycloheptane-1-carboxylate (2ae)

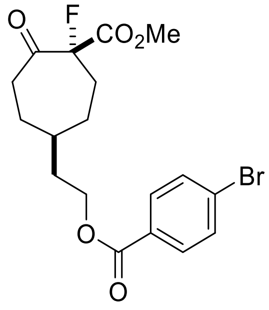

2ae

Compound **2ae** (dr 3.7:1) (34.8 mg, 83.8 µmol, 42%) was prepared according to general procedure B at 30 °C for 36 h with MeCN/H_2O (8/1, 0.5 M).

The two diastereoisomers could be separated by prep-TLC purification (1/4 EtOAc/Hexane).

Major Dia: **methyl (1S,5R)-5-(2-((4-bromobenzoyl)oxy)ethyl)-1-fluoro-2-oxocycloheptane-1-carboxylate (2ae-1)**

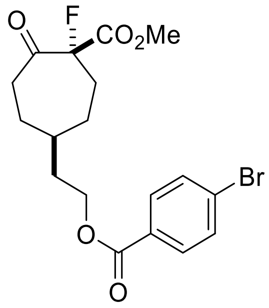

2ae-1

Colorless oil.

Rf = 0.52 (1/4 EtOAc/Hexane).

^{1}H NMR (400 MHz, $CDCl_3$) δ 7.90 – 7.84 (m, 2H), 7.61 – 7.56 (m, 2H), 4.36 (t, *J* = 6.7 Hz, 2H), 3.83 (s, 3H), 2.82-2.69 (m, 2H), 2.48 (dddd, *J* = 27.5, 15.4, 9.6, 2.7 Hz, 1H), 2.12 – 2.02 (m, 1H), 2.02 – 1.86 (m, 3H), 1.80 (q, *J* = 6.7 Hz, 2H), 1.76 – 1.58 (m, 2H).

^{19}F NMR (376 MHz, $CDCl_3$) δ -157.50 (ddd, *J* = 27.2, 15.9, 2.7 Hz).

^{13}C NMR (101 MHz, $CDCl_3$) δ 203.8 (d, *J* = 21.3 Hz), 167.9 (d, *J* = 25.3 Hz), 166.0, 131.9, 131.2, 129.1, 128.4, 98.7 (d, *J* = 196.6 Hz), 63.3, 53.3, 37.9, 34.2, 32.9, 30.1 (d, *J* = 23.0 Hz), 30.1, 28.8 (d, *J* = 3.9 Hz).

HRMS (ESI/QTOF) m/z: $[M + Na]^+$ Calcd for $C_{18}H_{20}BrFNaO_5^+$ 437.0370; Found 437.0365.

IR (ν_{max}, cm^{-1}) 2952 (w), 2932 (w), 2861 (w), 1748 (m), 1718 (s), 1590 (w), 1480 (w), 1440 (w), 1397 (w), 1364 (w), 1271 (s), 1205 (w), 1173 (m), 1106 (m), 1067 (w), 1011 (m), 963 (w), 849 (w), 757 (m), 683 (w).

Minor Dia: **methyl (1R,5R)-5-(2-((4-bromobenzoyl)oxy)ethyl)-1-fluoro-2-oxocycloheptane-1-carboxylate (2ae-2)**

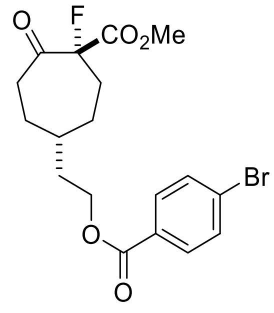

2ae-2

Colorless oil.

Rf = 0.50 (1/4 EtOAc/Hexane).

^{1}H NMR (600 MHz, $CDCl_3$) δ 7.89 – 7.86 (m, 2H), 7.61 – 7.56 (m, 2H), 4.36 (t, *J* = 6.5 Hz, 2H), 3.82 (s, 3H), 2.91 (td, *J* = 12.6, 3.5 Hz, 1H), 2.68 (ddt, *J* = 12.7, 6.5, 3.2 Hz, 1H), 2.40 – 2.28 (m, 2H), 2.12 – 2.05 (m, 1H), 1.93 – 1.87 (m, 1H), 1.75 (q, *J* = 6.4 Hz, 2H), 1.73 – 1.67 (m, 1H), 1.50 – 1.37 (m, 2H).

^{19}F NMR (565 MHz, $CDCl_3$) δ -164.80 – -164.97 (m).

^{13}C NMR (151 MHz, $CDCl_3$) δ 203.8 (d, *J* = 19.5 Hz), 167.7 (d, *J* = 25.4 Hz), 166.0, 132.0, 131.2, 129.2, 128.4, 98.8 (d, *J* = 199.5 Hz), 63.1, 53.5, 39.9, 38.3, 36.1, 33.6 (d, *J* = 23.0 Hz), 32.5, 29.8.

HRMS (ESI/QTOF) m/z: $[M + Na]^+$ Calcd for $C_{18}H_{20}BrFNaO_5^+$ 437.0370; Found 437.0365.

IR (ν_{max}, cm^{-1}) 2953 (w), 2927 (w), 2859 (w), 1764 (m), 1720 (s), 1590 (w), 1455 (w), 1437 (w), 1397 (w), 1271 (s), 1173 (w), 1113 (m), 1106 (m), 1068 (w), 1012 (m), 849 (w), 757 (m), 682 (w), 673 (w).

4-fluoro-4-(methoxycarbonyl)-5-oxocycloheptane-1-carboxylic acid (2af)

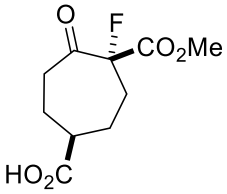

2af

Compound **2af** (dr 3.4:1) (24.6 mg, 105.9 µmol, 53%) was prepared according to general procedure B.

Colorless oil.

Rf = 0.40 (2/3 EtOAc/Hexane + 1% AcOH)

^{1}H NMR (400 MHz, $CDCl_3$) δ 3.82 (s, 3H), 3.00 (ddd, *J* = 13.2, 11.2, 3.5 Hz, 0.77H), 2.93 – 2.82 (m, 1H), 2.77 (ddt, *J* = 13.1, 6.6, 3.6 Hz, 0.23H), 2.67 (ddt, *J* = 13.6, 7.1, 3.4 Hz, 0.77H), 2.58 – 1.99 (m, 4.23H), 1.97 – 1.85 (m, 1.54H), 1.84 – 1.74 (m, 0.46H).

^{19}F NMR (377 MHz, $CDCl_3$) δ -161.67 (dd, *J* = 34.5, 15.0 Hz, major), -164.58 (dd, *J* = 36.5, 16.3 Hz, minor).

^{13}C NMR (101 MHz, $CDCl_3$) δ 203.2 (d, *J* = 19.7 Hz, major), 203.1 (d, *J* = 21.1 Hz, minor), 179.8 (major), 179.8 (minor), 167.5 (d, *J* = 25.4 Hz, major), 167.4 (d, *J* = 25.1 Hz, minor), 98.6 (d, *J* = 199.9 Hz, minor), 98.3 (d, *J* = 198.2 Hz, major), 53.5 (minor), 53.5 (major), 45.9 (minor), 42.1 (major), 39.0 (minor), 38.2 (major), 32.7 (d, *J* = 23.2 Hz, minor), 30.2 (d, *J* = 23.2 Hz, major), 27.9 (minor), 26.8 (major), 26.1 (minor), 25.0 (d, *J* = 1.8 Hz, major).

HRMS (nanochip-ESI/LTQ-Orbitrap) m/z: $[M + Na]^+$ Calcd for $C_{10}H_{13}FNaO_5^+$ 255.0639; Found 255.0634.

IR (ν_{max}, cm^{-1}) 3503 (w), 3232 (w), 2955 (w), 2870 (w), 1720 (s), 1704 (s), 1644 (w), 1454 (m), 1439 (m), 1284 (m), 1257 (m), 1213 (s), 1176 (m), 1126 (m), 1058 (m), 1032 (m), 1015 (m), 961 (m), 934 (m), 910 (m), 849 (m), 826 (m), 787 (m), 734 (m).

1,1-diethyl 4-methyl 4-fluoro-5-oxocycloheptane-1,1,4-tricarboxylate (2ag)

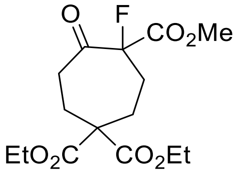

2ag

Compound **2ag** (27.3 mg, 82.1 µmol, 41%) was prepared according to general procedure B at 30 °C for 48 h.

Colorless oil.

^{1}H NMR (400 MHz, $CDCl_3$) δ 4.26 (qd, *J* = 7.1, 3.8 Hz, 2H), 4.17 (q, *J* = 7.1 Hz, 2H), 3.81 (s, 3H), 3.05 (td, *J* = 12.6, 2.4 Hz, 1H), 2.72 – 2.56 (m, 2H), 2.52 – 2.20 (m, 3H), 2.12 – 1.96 (m, 2H), 1.28 (t, *J* = 7.1 Hz, 3H), 1.24 (t, *J* = 7.1 Hz, 3H).

^{19}F NMR (377 MHz, $CDCl_3$) δ -163.17 (ddd, *J* = 35.0, 16.5, 3.0 Hz).

^{13}C NMR (101 MHz, $CDCl_3$) δ 202.6 (d, *J* = 19.3 Hz), 170.9, 170.3, 167.3 (d, *J* = 25.5 Hz), 97.9 (d, *J* = 199.1 Hz), 62.1, 62.0, 57.3, 53.5, 37.4, 30.9, 29.5 (d, *J* = 23.1 Hz), 28.2, 14.2, 14.1.

HRMS (nanochip-ESI/LTQ-Orbitrap) m/z: $[M + Na]^+$ Calcd for $C_{15}H_{21}FNaO_7^+$ 355.1164; Found 355.1155.

IR (ν_{max}, cm^{-1}) 2955 (w), 2923 (w), 2853 (w), 1768 (w), 1726 (s), 1443 (w), 1367 (w), 1295 (m), 1232 (s), 1209 (s), 1191 (s), 1145 (m), 1113 (w), 1094 (w), 1070 (m), 1024 (m), 952 (w), 859 (w), 788 (w), 764 (w), 732 (w), 690 (w).

methyl (3aR, 7aS)-5-fluoro-2,6-dioxooctahydro-1H-indene-5-carboxylate (2ah)

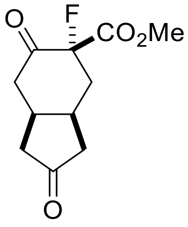

2ah

Compound **2ah** (dr 2.2:1) (20.9 mg, 91.6 µmol, 46%) was prepared according to general procedure B.

The two diastereoisomers could be separated by prep-TLC purification (3/2 EtOAc/Hexane).

Major dia: **methyl (3aR,5R,7aS)-5-fluoro-2,6-dioxooctahydro-1H-indene-5-carboxylate (2ah-1)**

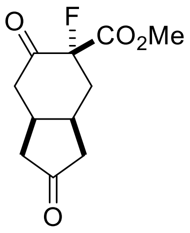

2ah-1

Colorless oil.

Rf = 0.55 (3/2 EtOAc/Hexane).

^{1}H NMR (400 MHz, $CDCl_3$) δ 3.84 (s, 3H), 3.22 (dt, *J* = 14.1, 6.2 Hz, 1H), 3.04 – 2.92 (m, 2H), 2.57 – 2.37 (m, 4H), 2.23 (ddd, *J* = 39.1, 13.4, 11.9 Hz, 1H), 2.17 (d, *J* = 18.0 Hz, 1H), 2.05 (dd, *J* = 18.9, 11.5 Hz, 1H).

^{19}F NMR (377 MHz, $CDCl_3$) δ -164.97 (ddd, *J* = 39.3, 10.9, 5.9 Hz).

^{13}C NMR (101 MHz, $CDCl_3$) δ 215.4, 201.3 (d, *J* = 23.4 Hz), 166.8 (d, *J* = 24.5 Hz), 95.2 (d, *J* = 194.2 Hz), 53.3, 45.2, 40.2, 39.2 (d, *J* = 2.1 Hz), 38.1, 36.7 (d, *J* = 22.8 Hz), 30.8 (d, *J* = 2.3 Hz).

HRMS (nanochip-ESI/LTQ-Orbitrap) m/z: $[M + Na]^+$ Calcd for $C_{11}H_{13}FNaO_4^+$ 251.0690; Found 251.0694.

IR (ν_{max}, cm^{-1}) 2958 (w), 2922 (w), 1737 (s), 1439 (w), 1407 (w), 1337 (w), 1300 (m), 1278 (m), 1232 (w), 1201 (w), 1160 (w), 1128 (w), 1067 (m), 1037 (w), 998 (w), 972 (w), 944 (w), 892 (w), 804 (w), 763 (w), 665 (w).

Compound 2ah-1

^{19}F NMR (377 MHz, $CDCl_3$) δ
-164.97 (ddd, J = 39.1, 10.9, 5.9 Hz)
^{3}J(Ha-Fa) = 39.1,
^{3}J(He-Fa) = 10.9 Hz
^{4}J(H-Fa) = 5.9 Hz

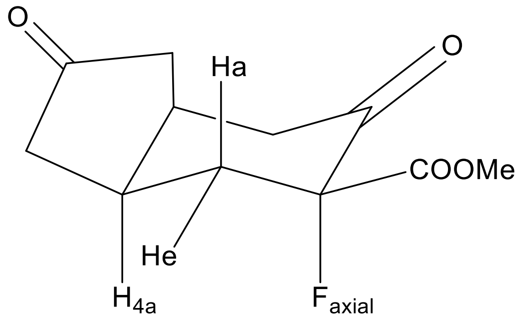

He: 2.47-2.38 (m, 1H)
Ha: 2.23 (ddd, J = 39.1, 13.4, 11.9 Hz)
^{3}J(Ha-Fa) = 39.1 Hz
^{2}J(Ha-He) and ^{3}J(Ha-H_{4a}) = 13.4 or 11.9 Hz
F is axial, H_{4a} is axial

Minor dia: **methyl (3aR,5S,7aS)-5-fluoro-2,6-dioxooctahydro-1H-indene-5-carboxylate (2ah-2)**

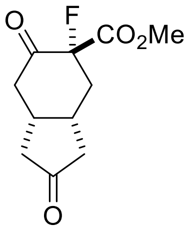

2ah-2

Colorless oil.

Rf = 0.45 (3/2 EtOAc/Hexane).

^{1}H NMR (400 MHz, $CDCl_3$) δ 3.88 (s, 3H), 3.10 – 2.87 (m, 3H), 2.75 – 2.59 (m, 2H), 2.54 – 2.38 (m, 2H), 2.31 (dd, J = 18.6, 3.6 Hz, 1H), 2.13 – 1.95 (m, 2H).

^{19}F NMR (377 MHz, $CDCl_3$) δ -159.61 (td, J = 12.9, 4.6 Hz).

^{13}C NMR (101 MHz, $CDCl_3$) δ 215.5, 200.1 (d, J = 18.2 Hz), 167.0 (d, J = 24.7 Hz), 95.3 (d, J = 201.3 Hz), 53.6, 44.6, 41.5, 40.5, 38.2, 37.3 (d, J = 20.2 Hz), 33.1 (d, J = 6.7 Hz).

HRMS (nanochip-ESI/LTQ-Orbitrap) m/z: $[M + Na]^+$ Calcd for $C_{11}H_{13}FNaO_4^+$ 251.0690; Found 251.0694.

IR (ν_{max}, cm^{-1}) 2960 (w), 2920 (w), 2854 (w), 1734 (s), 1439 (w), 1408 (w), 1365 (w), 1300 (w), 1266 (w), 1202 (w), 1146 (m), 1106 (w), 1044 (w), 1021 (w), 997 (w), 971 (w), 952 (w), 835 (w), 805 (w), 663 (w).

Compound 2ah-2

^{19}F NMR (400 MHz, $CDCl_3$)
δ -159.61 (td, *J* = 12.9, 4.6 Hz)
^3J(Ha-Fe) = ^3J(He-Fe) = 12.9 Hz
^4J(H-Fe) = 4.6 Hz

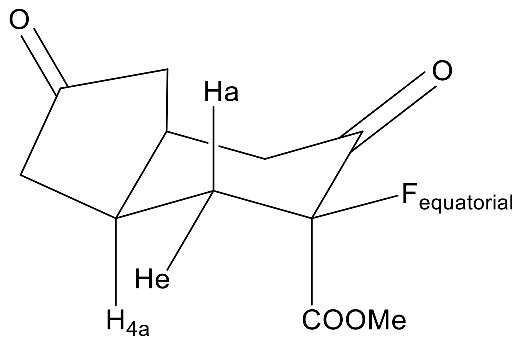

Ha: 2.01 (td, *J* = 14.3, 10.4 Hz, 1H)
^3J(Ha-Fe) = 14.3 Hz
^2J(Ha-He) = 14.3 Hz
^3J(Ha-H_{4a}) = 10.4 Hz
He: 2.64 (ddd, *J* = 14.1, 12.1, 5.5 Hz, 1H)
^3J(He-Fe) = 12.1 Hz
^2J(He-Ha) = 14.1 Hz
^3J(He-H_{4a}) = 5.5 Hz
F is equatorial, H_{4a} is axial

methyl 4-fluoro-5-oxooxepane-4-carboxylate (2ai)

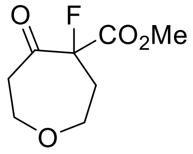

2ai

Compound **2ai** (22.1 mg, 116.2 µmol, 58%) was prepared according to general procedure B.

Colorless oil.

Rf = 0.25 (1/4 EtOAc/Hexane).

^{1}H NMR (400 MHz, $CDCl_3$) δ 4.08-4.01 (m, 2H), 3.84 (s, 3H), 3.80 – 3.73 (m, 1H), 3.69 (ddd, *J* = 13.0, 10.5, 2.1 Hz, 1H), 3.02 (dddd, *J* = 14.2, 9.6, 5.7, 1.1 Hz, 1H), 2.92 (dtd, *J* = 14.4, 4.3, 2.8 Hz, 1H), 2.47 (dddd, *J* = 35.8, 15.9, 10.6, 3.7 Hz, 1H), 2.20 (dddd, *J* = 15.9, 12.7, 4.4, 2.0 Hz, 1H).

^{19}F NMR (377 MHz, $CDCl_3$) δ -164.48 (dd, *J* = 35.5, 12.7 Hz).

^{13}C NMR (101 MHz, $CDCl_3$) δ 201.5 (d, *J* = 23.3 Hz), 167.2 (d, *J* = 25.1 Hz), 98.7 (d, *J* = 197.9 Hz), 67.3 (d, *J* = 1.8 Hz), 66.8 (d, *J* = 1.6 Hz), 53.4, 43.8, 35.2 (d, *J* = 22.1 Hz).

HRMS (ESI/QTOF) m/z: $[M + Na]^+$ Calcd for $C_8H_{11}FNaO_4^+$ 213.0534; Found 213.0531.

IR (ν_{max}, cm^{-1}) 2958 (w), 2920 (w), 2852 (w), 1756 (s), 1725 (s), 1438 (m), 1280 (s), 1250 (m), 1213 (m), 1131 (s), 1119 (s), 1098 (s), 1054 (m), 1020 (s), 984 (m), 937 (m), 887 (w), 850 (w), 818 (m), 792 (w).

methyl (3aS,3bR,5aS,7S,10aS,10bS,12aS)-7-fluoro-10a,12a-dimethyl-1,8-dioxooctadecahydrocyclohepta[a]cyclopenta[f]naphthalene-7-carboxylate (2aj) & methyl (3aS,3bR,5aS,8S,10aS,10bS,12aS)-8-fluoro-10a,12a-dimethyl-1,7-dioxooctadecahydrocyclohepta[a]cyclopenta[f]naphthalene-8-carboxylate (2aj’)

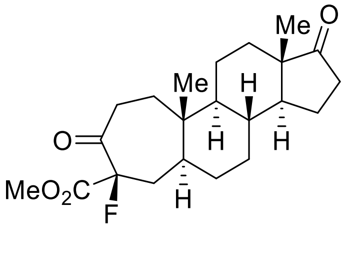

2aj

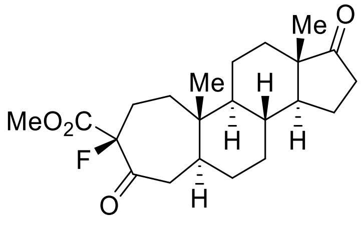

2aj'

Compound **2aj + 2aj’** (rr 1:1; dr 7:1 & 5.2:1) (45.6 mg, 120.5 µmol, 60%) was prepared according to general procedure B at 30 °C for 18 h with MeCN/H_2O (8/1, 0.5 M).

White solid.

Rf = 0.28 (1/3 EtOAc/Hexane).

^{1}**H NMR** (400 MHz, $CDCl_3$) (As the four isomers could not be isolated, the obtained analytical data refer to a complex mixture. We therefore only report the observed shifts for the two major isomers) δ 3.84, 3.84, 3.80 (three s, 3H, OMe), 2.83 (ddd, *J* = 14.7, 12.0, 2.3 Hz, 0.5H, CH_2), 2.78 – 2.68 (m, 0.5H, CH_2), 2.55 – 2.33 (m, 2H, CH_2), 2.28 – 2.22 (m, 0.5H), 2.12 – 1.88 (m, 5H CH_2), 1.88 – 1.68 (m, 3H (CH_2), + 0.5H (CH)), 1.60 – 1.35 (m, 1.5H (CH) + 3.5H (CH_2)), 1.35 – 1.16 (m, 1H (CH) + 2H (CH_2)), 1.08-0.96 (m, 1H, CH_2), 0.88, 0.85, 0.85 (three s, 6H, 2CH_3), 0.90 – 0.76 (m, 1H, CH).

^{19}**F NMR** (377 MHz, $CDCl_3$) δ -146.16 (dd, *J* = 16.0, 9.4 Hz, major diastereoisomer), -147.07 (apparent t, *J* = 18.7 Hz, major diastereoisomer), -163.50 (ddd, *J* = 38.9, 16.1, 3.1 Hz, minor diastereoisomer), -165.51 (ddd, *J* = 40.3, 16.9, 3.3 Hz, minor diastereoisomer).

^{13}**C NMR** (101 MHz, $CDCl_3$) (As the four isomers could not be isolated, the obtained analytical data refer to a complex mixture. We therefore only report the observed shifts for the two major isomers) δ 220.87, 220.85, 203.25 (d, *J* = 19.6 Hz), 202.33 (d, *J* = 20.3 Hz), 168.3 (d, *J* = 25.5 Hz), 168.0 (d, *J* = 25.2 Hz), 97.7 (d, *J* = 192.9 Hz), 97.0 (d, *J* = 190.2 Hz), 53.2 (CH), 53.14 (CH_3), 53.07 (CH_3), 52.4 (CH), 51.50 (CH), 51.49 (CH), 47.7 (Cq), 47.5 (Cq), 44.5 (CH_2), 44.1 (CH), 44.0 (d, *J* = 8.6 Hz, CH), 39.0 (Cq), 38.7 (Cq), 36.8 (d, *J* = 21.8 Hz, CH_2), 36.5 (CH_2), 36.1 (CH_2), 36.0 (2 CH_2), 34.9 (CH), 34.8 (CH), 34.5 (d, *J* = 6.6 Hz, CH_2), 31.72 (CH_2), 31.70 (CH_2), 30.9 (CH_2), 30.8 (CH_2), 30.7 (CH_2), 30.1 (CH_2), 28.7 (d, *J* = 23.8 Hz, CH_2), 21.85 (CH_2), 21.83 (CH_2), 21.3 (CH_2), 21.2 (CH_2), 13.93 (CH_3), 13.86 (CH_3), 13.2 (CH_3), 12.1 (CH_3).

HRMS (APCI/QTOF) m/z: $[M + Na]^+$ Calcd for $C_{22}H_{31}FNaO_4^+$ 401.2099; Found 401.2092.

IR (ν_{max}, cm^{-1}) 2932 (w), 2858 (w), 1760 (m), 1733 (s), 1450 (w), 1373 (w), 1341 (w), 1290 (w), 1270 (w), 1236 (w), 1203 (w), 1174 (w), 1083 (w), 1057 (w), 1010 (w), 971 (w), 912 (m), 830 (w), 729 (s).

methyl 2-oxocycloheptane-1-carboxylate & methyl 2-hydroxycyclohept-1-ene-1-carboxylate (3a)

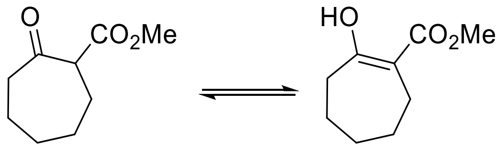

3a

Procedure 1: Compound **3a** (806 mg, 4.74 mmol, 95%), obtained as a 3:1 mixture of ketone/enol isomers, was prepared according to reported procedure using toluene instead of benzene.[17]

Procedure 2: To fluorinated intermediate **5** (4.0 mg, 13.6 µmol, 1.0 equiv) in dry THF (270 µL) was slowly added NaOMe (5.4 M in MeOH, 26 µL, 136 µmol, 10.0 equiv) at rt and it was further stirred for 30 minutes before quenching with saturated aqueous solution of NH_4Cl. It was then extracted three times with EtOAc. The combined organic layers were washed with brine, dried over Na_2SO_4, filtered and concentrated under reduced pressure. The crude product was purified by prep-TLC (1/4 EtOAc/Hexane) to give **3a** (2.1 mg, 12.3 µmol, 91%) as a colorless oil.

The analytical data were in agreement with those reported in the literature.[17]

Colorless oil.

Rf = 0.45 (1/4 EtOAc/Hexane).

^{1}H NMR (400 MHz, $CDCl_3$) δ (the ratio of ketone and enol is around 9:1) 12.64 (s, 0.1H, enol OH), 3.73 (s, 0.3H, enol Me), 3.71 (s, 2.7H, ketone Me), 3.54 (dd, *J* = 10.3, 4.0 Hz, 0.9H), 2.66 – 2.52 (m, 1.8H), 2.45 – 2.36 (m, 0.4H), 2.13 – 2.04 (m, 0.9H), 1.97-1.52 (m, 4.9H), 1.50 – 1.37 (m, 2H).

^{13}C NMR (101 MHz, $CDCl_3$) δ 209.1 (ketone), 179.8 (enol), 173.5 (enol), 171.1 (ketone), 101.6 (enol), 59.0 (ketone), 52.3 (ketone), 51.6 (enol), 43.2 (ketone), 35.5 (enol), 32.1 (enol), 29.8 (ketone), 28.1 (ketone), 27.7 (ketone), 27.5 (enol), 24.8 (enol), 24.5 (ketone), 24.5 (enol).

HRMS (Sicrit plasma/LTQ-Orbitrap) m/z: $[M + H]^+$ Calcd for $C_9H_{15}O_3^+$ 171.1016; Found 171.1015.

IR (ν_{max}, cm^{-1}) 2925 (m), 2853 (w), 1743 (m), 1706 (m), 1641 (m), 1612 (m), 1440 (m), 1381 (w), 1356 (m), 1311 (m), 1271 (m), 1240 (s), 1214 (s), 1196 (s), 1143 (m), 1049 (m), 1017 (w), 961 (w), 940 (w), 920 (w), 861 (m), 830 (m), 786 (w), 723 (w), 673 (w).

methyl 1-fluoro-2-(oxo-18O)cycloheptane-1-carboxylate (^{18}O-2a)

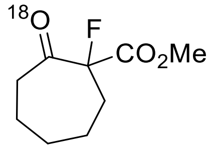

^{18}O-2a

Compound **^{18}O-2a** (22.2 mg, 116.8 µmol, 58%, 64% ^{18}O incorporation) was prepared according to general procedure B using ^{18}O labelled water $H_2{}^{18}O$ (97% ^{18}O).

Colorless oil.

Rf = 0.45 (1/4 EtOAc/Hexane).

^{1}H NMR (600 MHz, $CDCl_3$) δ 3.80 (s, 3H), 2.80 – 2.74 (m, 1H), 2.72 – 2.67 (m, 1H), 2.31 – 2.15 (m, 2H), 1.98 – 1.80 (m, 3H), 1.66 – 1.58 (m, 1H), 1.57 – 1.49 (m, 1H), 1.46 – 1.38 (m, 1H).

^{19}F {^{1}H} NMR (376 MHz, $CDCl_3$) δ -163.51 (0.36 F, minor), -163.52 (0.64 F, major).

^{13}C NMR (151 MHz, $CDCl_3$) δ 204.35 (d, J = 20.5 Hz, 0.36 C, minor), 204.30 (d, J = 21.0 Hz, 0.64 C, major), 167.9 (d, J = 25.4 Hz), 99.1 (d, J = 198.4 Hz), 53.3, 40.9, 34.0 (d, J = 22.9 Hz), 29.1, 25.8, 23.9.

^{13}C {^{1}H, ^{19}F} NMR (101 MHz, $CDCl_3$) δ 204.35 (0.36 C, minor), 204.30 (0.64 C, major), 167.9, 99.1, 53.3, 40.9, 34.0, 29.1, 25.8, 23.9.

HRMS (ESI/QTOF) m/z: $[M + Na]^+$ Calcd for $C_9H_{13}FNaO_2[^{18}O]^+$ 213.0783; Found 213.0792. $[M + Na]^+$ Calcd for $C_9H_{13}FNaO_2[^{16}O]^+$ 211.0741; Found 211.0752.

IR (ν_{max}, cm^{-1}) 2938 (m), 2861 (w), 1760 (s), 1731 (s), 1693 (s), 1439 (m), 1352 (w), 1276 (s), 1257 (s), 1239 (s), 1199 (m), 1165 (s), 1151 (s), 1064 (m), 1041 (s), 1005 (m), 942 (m), 889 (w), 843 (w), 803 (w), 780 (w), 743 (w).

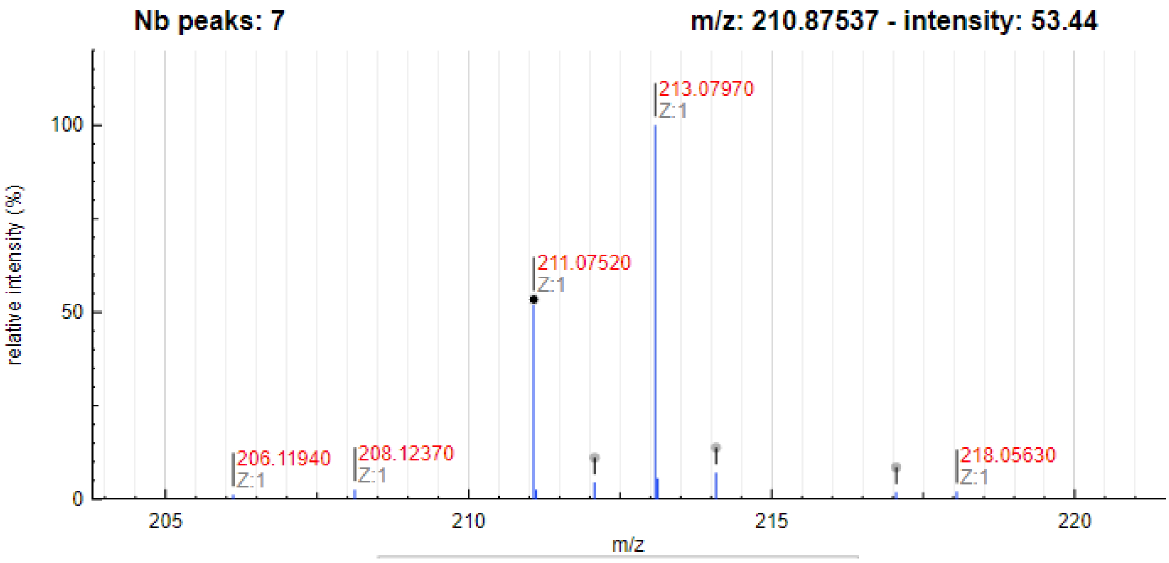

HMRS analysis of **2a - ^{18}O/^{16}O** was in accordance with the 64% ^{18}O incorporation observed by NMR.

methyl 2-(benzoyloxy)-2-fluorocycloheptane-1-carboxylate (5)

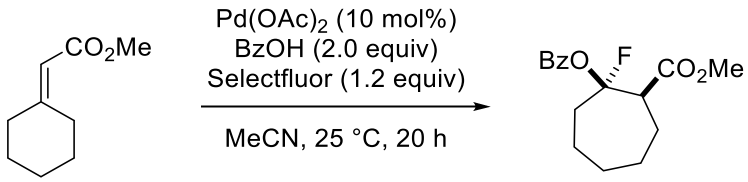

1a **5**

Dry MeCN (400 µL, 0.5 M) was added to a test tube containing unsaturated ester **1a** (30.8 mg, 0.2 mmol, 1.0 equiv), $Pd(OAc)_2$ (4.5 mg, 20 µmol, 10 mol%), benzoic acid (48.9 mg, 0.4 mmol, 2.0 equiv) and Selectfluor (85.0 mg, 0.24 mmol, 1.2 equiv). The tube was capped under Ar atmosphere and it was stirred at 25 °C for 20 h. Filtration on a pad of silica gel eluted with Et_2O followed by prep-TLC purification (1/8 Et_2O/Hexane) afforded **5** (5.1 mg, 17.3 µmol, 9% yield) as a colorless oil.

N.B.: *Compound **5** is probably not very stable on silica gel purification.*

Colorless oil.

Rf = 0.45 (1/8 Et_2O/Hexane).

^{1}H NMR (400 MHz, $CDCl_3$) δ 8.04 – 8.01 (m, 2H), 7.61 – 7.56 (m, 1H), 7.48 – 7.43 (m, 2H), 3.64 (s, 3H), 3.55 (ddd, *J* = 21.2, 9.8, 3.3 Hz, 1H), 2.73 – 2.60 (m, 1H), 2.58 – 2.48 (m, 1H), 2.05 – 1.97 (m, 1H), 1.95 – 1.52 (m, 7H).

^{19}F NMR (376 MHz, $CDCl_3$) δ -108.82 (td, *J* = 21.8, 10.1 Hz).

^{13}C NMR (101 MHz, $CDCl_3$) δ 171.8, 163.6 (d, *J* = 3.9 Hz), 133.6, 129.9, 128.7, 128.6, 118.0 (d, *J* = 234.0 Hz), 52.3 (d, *J* = 22.4 Hz), 52.1, 36.5 (d, *J* = 24.6 Hz), 27.4, 25.7, 25.5 (d, *J* = 4.0 Hz), 21.3 (d, *J* = 6.5 Hz).

HRMS (APCI/QTOF) m/z: $[M + Na]^+$ Calcd for $C_{16}H_{19}FNaO_4^+$ 317.1160; Found 317.1145.

IR (ν_{max}, cm^{-1}) 2939 (w), 2867 (w), 1737 (s), 1451 (w), 1369 (w), 1314 (w), 1265 (s), 1205 (m), 1177 (m), 1147 (m), 1099 (m), 1068 (m), 1021 (m), 993 (m), 919 (w), 709 (s).

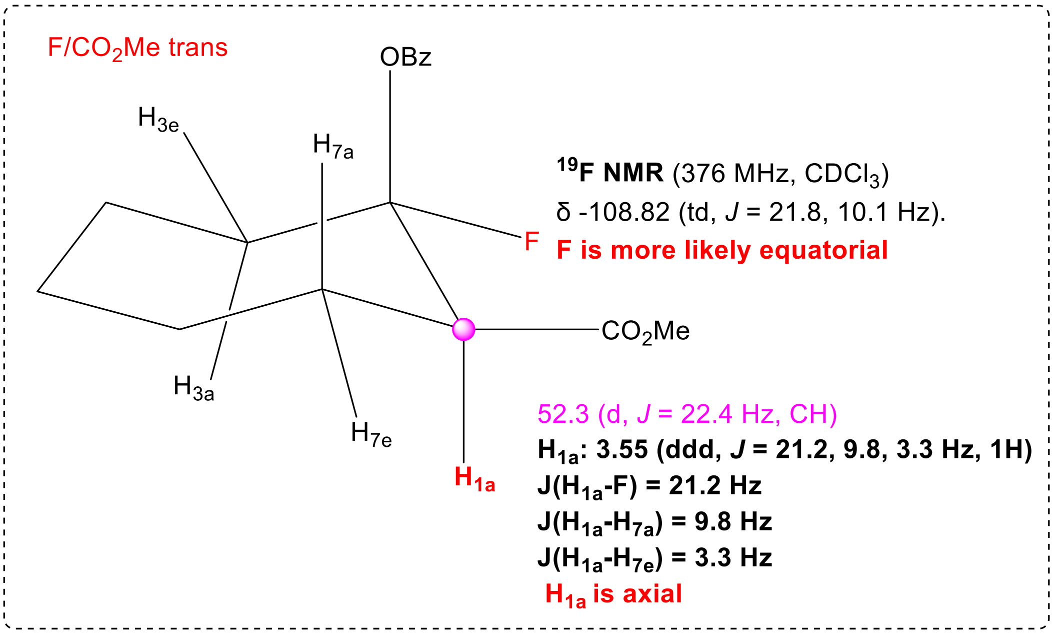

ethyl 2-hydroxy-2-(1-hydroxycyclopentyl)acetate (4j)

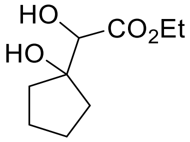

4j

Compound **4j** (67.8 mg, 360 μmol, 18%) was obtained from **1j** using general procedure B.

Colorless oil.

Rf = 0.43 (1/1 EtOAc/Hexane).

^{1}H NMR (400 MHz, $CDCl_3$) δ 4.35 – 4.20 (m, 3H), 4.04 (s, 1H), 3.32 (s, 1H), 2.31 (s, 1H), 1.86 – 1.58 (m, 8H), 1.31 (t, J = 7.2 Hz, 3H).

^{13}C NMR (101 MHz, $CDCl_3$) δ 173.6, 83.6, 76.4, 62.1, 37.9, 36.2, 24.3, 23.7, 14.3.

HRMS (ESI/QTOF) m/z: $[M + Na]^+$ Calcd for $C_9H_{16}NaO_4^+$ 211.0941; Found 211.0943.

IR (ν_{max}, cm^{-1}) 3459 (w), 2961 (w), 2874 (w), 1728 (s), 1446 (w), 1371 (m), 1266 (m), 1202 (s), 1092 (s), 1063 (m), 1020 (s), 951 (w), 862 (w), 841 (w).

D) Starting materials of failed examples

Alkene modifications:

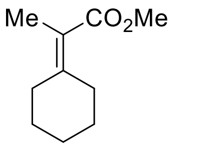

1ak

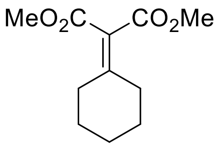

1al

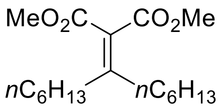

1am

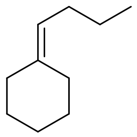

1an

Allylic substituted:

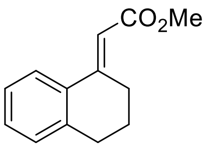

S6

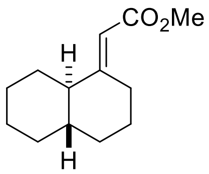

S7

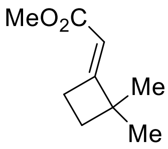

S8

Heterocycles:

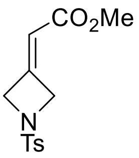

S9

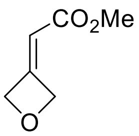

S10

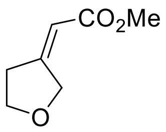

S11

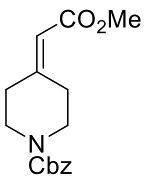

S12

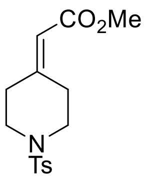

S13

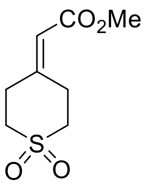

S14

1ak – **1an**, **S6** and **S10** – **S12** are literature known compounds.

methyl 2-octahydronaphthalen-1(2H)-ylidene)acetate (S7)

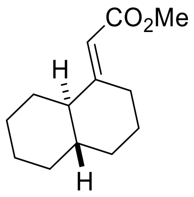

S7

Compound **S7** (124 mg, 0.68 mmol, 95%) was prepared according to general procedure A1.

Colorless oil.

Rf = 0.38 (1/15 Et_2O/Hexane).

^{1}H NMR (400 MHz, $CDCl_3$) δ 5.52 (t, *J* = 1.6 Hz, 1H), 3.94 (dtd, *J* = 12.9, 3.3, 1.7 Hz, 1H), 3.68 (s, 3H), 1.95 – 1.63 (m, 8H), 1.48 – 1.03 (m, 7H).

^{13}C NMR (101 MHz, $CDCl_3$) δ 167.9, 166.9, 110.0, 51.0, 49.5, 45.6, 35.1, 34.5, 30.9, 28.7, 27.9, 26.3, 26.1.

HRMS (ESI/QTOF) m/z: $[M + H]^+$ Calcd for $C_{13}H_{21}O_2^+$ 209.1536; Found 209.1537.

IR (ν_{max}, cm^{-1}) 2922 (m), 2851 (w), 1718 (s), 1638 (m), 1446 (w), 1433 (m), 1255 (w), 1203 (m), 1160 (s), 1133 (s), 1033 (w), 925 (w), 858 (w), 733 (w).

methyl 2-(2,2-dimethylcyclobutylidene)acetate (S8)

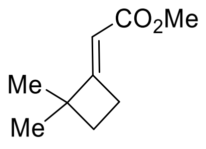

S8

Compound **S8** ((*E*)/(*Z*) = 5:1, 43 mg, 0.28 mmol, 47%) was prepared according to general procedure A2.

Colorless oil.

Rf = 0.44 (1/9 EtOAc/Hexane).

^{1}H NMR (400 MHz, $CDCl_3$) δ 5.57 (t, *J* = 2.5 Hz, 0.8H), 5.52 (t, *J* = 2.2 Hz, 0.2H), 3.67 (s, 2.5H), 3.65 (s, 0.5H), 3.05 (td, *J* = 8.1, 2.5 Hz, 1.67H), 2.67 (td, *J* = 8.3, 2.1 Hz, 0.33H), 1.89 – 1.81 (m, 2H), 1.34 (s, 1H), 1.18 (s, 5H).

^{13}C NMR (101 MHz, $CDCl_3$) δ 177.1 (major), 175.2 (minor), 167.5 (major), 165.9 (minor), 112.4 (minor), 109.7 (major), 51.0 (major), 50.9 (minor), 46.5 (minor), 45.0 (major), 32.4 (major), 32.1 (minor), 28.6 (major), 27.0 (major), 26.3 (minor), 25.9 (minor).

HRMS (ESI/QTOF) m/z: $[M + H]^+$ Calcd for $C_9H_{15}O_2^+$ 155.1067; Found 155.1061.

IR (ν_{max}, cm^{-1}) 2952 (m), 2861 (w), 1717 (s), 1673 (m), 1436 (m), 1339 (s), 1271 (m), 1172 (s), 1056 (m), 1021 (m), 853 (m).

methyl 2-(1-tosylazetidin-3-ylidene)acetate (S9)

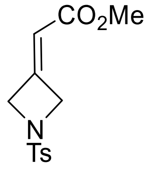

S9

Compound **S9** (220 mg, 0.78 mmol, 78%) was prepared according to general procedure A1.

White solid.

Rf = 0.35 (1/4 EtOAc/Hexane).

^{1}H NMR (400 MHz, $CDCl_3$) δ 7.77 – 7.72 (m, 2H), 7.37 (d, *J* = 8.0 Hz, 2H), 5.69 (p, *J* = 2.4 Hz, 1H), 4.73 – 4.70 (m, 2H), 4.51 – 4.47 (m, 2H), 3.67 (s, 3H), 2.44 (s, 3H).

^{13}C NMR (101 MHz, $CDCl_3$) δ 165.4, 149.8, 144.7, 131.6, 130.1, 128.5, 114.3, 61.6, 59.1, 51.7, 21.7.

HRMS (ESI/QTOF) m/z: $[M + H]^+$ Calcd for $C_{13}H_{16}NO_4S^+$ 282.0795; Found 282.0802.

IR (ν_{max}, cm^{-1}) 2978 (w), 2949 (w), 2915 (w), 2855 (w), 1722 (m), 1701 (m), 1431 (m), 1341 (s), 1253 (m), 1215 (m), 1162 (s), 1105 (s), 1092 (m), 1075 (m), 1026 (s), 918 (w), 908 (w), 817 (m), 778 (w), 723 (m), 711 (m), 672 (s).

Mp = 127 – 129 °C.

methyl 2-(1-tosylpiperidin-4-ylidene)acetate (S13)

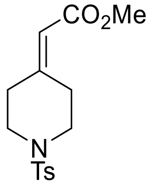

S13

Compound **S13** (142 mg, 0.46 mmol, 46%) was prepared according to general procedure A1.

Rf = 0.35 (1/2 EtOAc/Hexane).

^{1}**H NMR** (400 MHz, $CDCl_3$) δ 7.68 – 7.64 (m, 2H), 7.31 (d, *J* = 8.0 Hz, 2H), 5.49 (tt, *J* = 3.3, 1.6 Hz, 1H), 3.64 (s, 3H), 3.60 – 3.56 (m, 2H), 3.18 (t, *J* = 5.7 Hz, 2H), 2.99 – 2.96 (m, 2H), 2.42 (s, 3H), 2.26 – 2.20 (m, 2H).

^{13}**C NMR** (101 MHz, $CDCl_3$) δ 171.4, 143.7, 133.2, 130.0, 129.8, 127.9, 121.2, 52.0, 44.9, 42.9, 42.1, 28.5, 21.6.

HRMS (ESI/QTOF) m/z: $[M + H]^+$ Calcd for $C_{15}H_{20}NO_4S^+$ 310.1108; Found 310.1111.

IR (ν_{max}, cm^{-1}) 2935 (w), 2870 (w), 2841 (w), 1725 (m), 1438 (w), 1332 (m), 1290 (w), 1211 (m), 1174 (m), 1161 (s), 1146 (s), 1093 (m), 985 (m), 971 (m), 942 (s), 919 (m), 816 (m), 802 (w), 771 (m), 725 (m), 711 (m), 680 (s).

Mp = 73 – 75 °C.

methyl 2-(1,1-dioxidotetrahydro-4H-thiopyran-4-ylidene)acetate (S14)

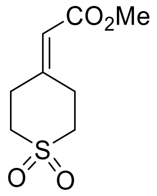

S14

At 0 °C, mCPBA (77%, 661 mg, 3.83 mmol, 2.0 equiv) was added portionwise to a solution of the known corresponding thioether[18] (330 mg, 1.91 mmol, 1.0 equiv) in dry DCM (20 mL). The reaction mixture was stirred at rt for 16 h and the mixture was then filtered (washed with DCM). A saturated aqueous solution of $NaHCO_3$ and $Na_2S_2O_3$ (v/v 1/1) was added and the crude product was extracted three times with DCM. The combined organic layers were washed with brine, dried over Na_2SO_4, filtered and concentrated under reduced pressure. The crude product was purified by FCC (4/1 EtOAc/Hexane) to give sulfone **S14** (380 mg, 1.86 mmol, 97%) as pale-yellow crystals.

Pale-yellow crystals.

Rf = 0.29 (2/1 EtOAc/Hexane).

^{1}H NMR (400 MHz, $CDCl_3$) δ 5.86 (t, *J* = 1.1 Hz, 1H), 3.73 (s, 3H), 3.50 – 3.44 (m, 2H), 3.16 – 3.08 (m, 4H), 2.85 – 2.78 (m, 2H).

^{13}C NMR (101 MHz, $CDCl_3$) δ 166.0, 151.8, 119.1, 52.2, 51.7, 51.6, 34.6, 26.0.

HRMS (ESI/QTOF) m/z: $[M + Na]^+$ Calcd for $C_8H_{12}NaO_4S^+$ 227.0349; Found 227.0347.

IR (ν_{max}, cm^{-1}) 2957 (w), 2923 (m), 2850 (w), 1713 (s), 1652 (m), 1429 (m), 1381 (m), 1319 (m), 1306 (m), 1277 (s), 1245 (s), 1161 (s), 1115 (s), 1030 (s), 933 (s), 903 (m), 877 (s), 856 (m), 774 (w), 744 (m), 724 (m), 701 (m), 684 (m).

Mp = 128 – 130 °C.

E) References

[1] A. M. Sheta, A. Alkayal, M. A. Mashaly, S. B. Said, S. S. Elmorsy, A. V. Malkov, B. R. Buckley, *Angew. Chem. Int. Ed.* **2021**, *60*, 21832–21837.

[2] Y. Chen, L. Huang, X. P. Zhang, *Org. Lett.* **2003**, *5*, 2493–2496.

[3] M. E. Weiss, L. M. Kreis, A. Lauber, E. M. Carreira, *Angew. Chem. Int. Ed.* **2011**, *50*, 11125–11128.

[4] S. E. Denmark, I. Rivera, *J. Org. Chem.* **1994**, *59*, 6887–6889.

[5] G. Kang, D. Romo, *ACS Catal.* **2021**, *11*, 1309–1315.

[6] P. Szcześniak, M. Pieczykolan, S. Stecko, *J. Org. Chem.* **2016**, *81*, 1057–1074.

[7] M. Konda, R. G. Jadhav, S. Maiti, S. M. Mobin, B. Kauffmann, A. K. Das, *Org. Biomol. Chem.* **2018**, *16*, 1728–1735.

[8] K. Bodenschatz, J. Stöckl, M. Winterer, R. Schobert, *Tetrahedron* **2022**, *104*, 132113.

[9] T. Rigotti, D. P. Schwinger, R. Graßl, C. Jandl, T. Bach, *Chem. Sci.* **2022**, *13*, 2378–2384.

[10] M. Yu, G. Li, S. Wang, L. Zhang, *Adv. Synth. Catal.* **2007**, *349*, 871–875.

[11] P. Van der Veken, K. Senten, I. Kertèsz, I. De Meester, A.-M. Lambeir, M.-B. Maes, S. Scharpé, A. Haemers, K. Augustyns, *J. Med. Chem.* **2005**, *48*, 1768–1780.

[12] Q. Wu, N. Chen, J. Xu, *ChemistrySelect* **2022**, *7*, e202103943.

[13] K. L. Perlman, R. R. Sicinski, H. M. Darwish, H. F. DeLuca, *Bioorg. Med. Chem. Lett.* **1995**, *5*, 2695–2700.

[14] T. C. Nugent, F. Goswami, S. Debnath, I. Hussain, H. Ali El Damrany Hussein, A. Karn, S. Nakka, *Adv. Synth. Catal.* **2021**, *363*, 3539–3545.

[15] N. Itagaki, M. Kimura, T. Sugahara, Y. Iwabuchi, *Org. Lett.* **2005**, *7*, 4185–4188.

[16] K. Balaraman, R. Vasanthan, V. Kesavan, *Tetrahedron: Asymmetry* **2013**, *24*, 919–924.

[17] S. Ikeda, M. Shibuya, N. Kanoh, Y. Iwabuchi, *Org. Lett.* **2009**, *11*, 1833–1836.

[18] P. Pasman, J. W. Verhoeven, Th. J. deBoer, *Tetrahedron* **1976**, *32*, 2827–2830.

3) Copies of NMR Spectra

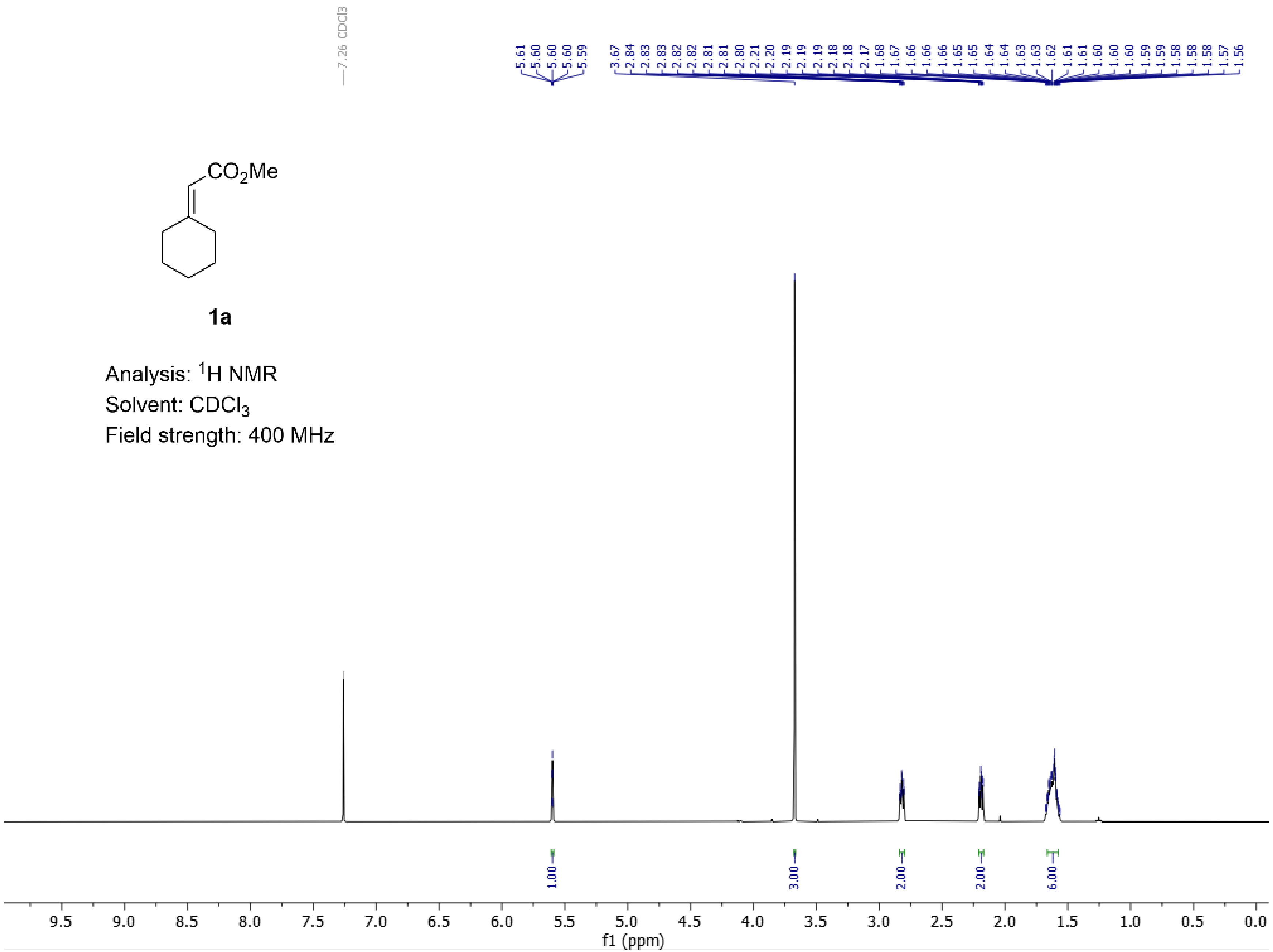

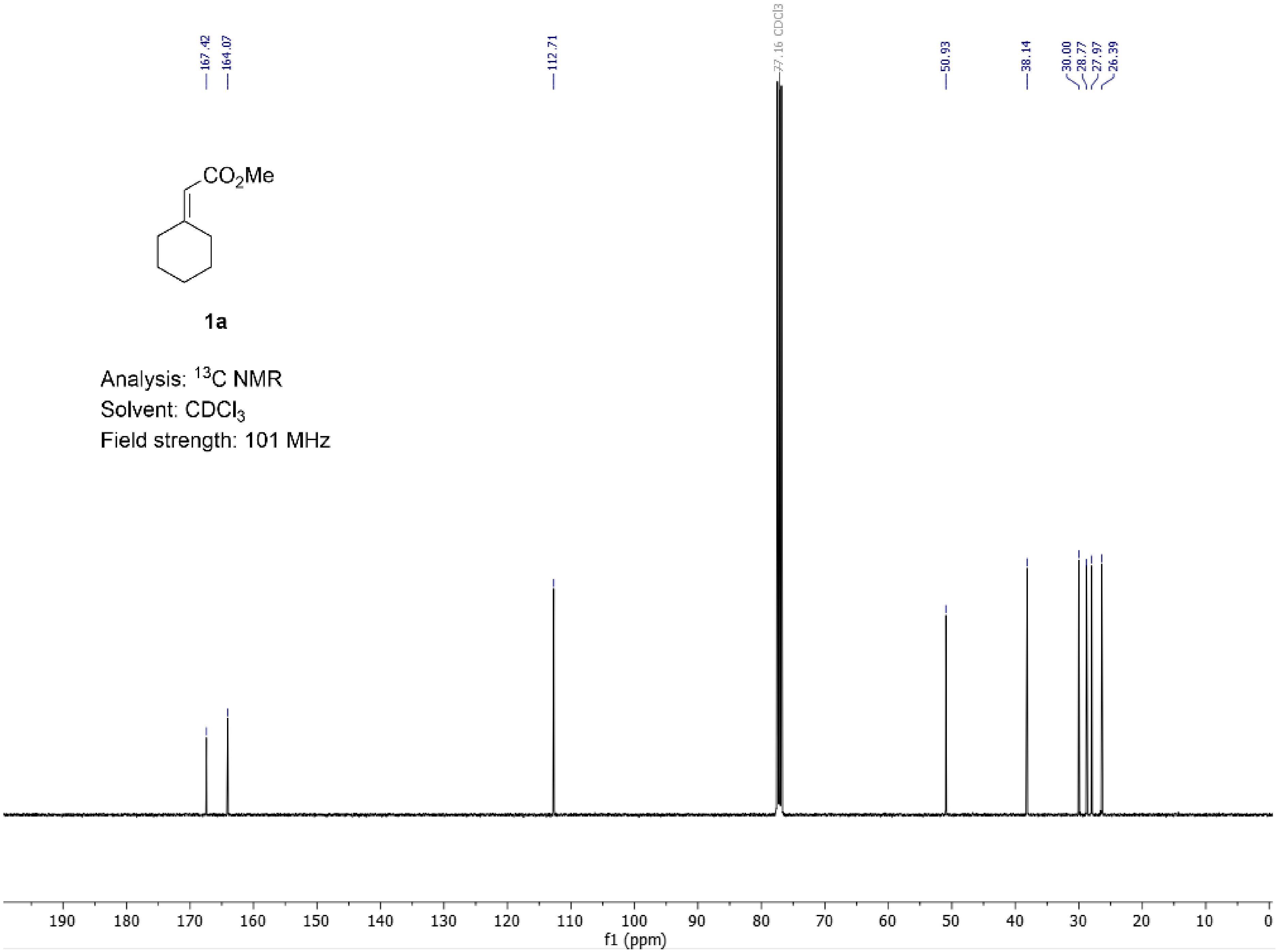
167.42
164.07
112.71
77.16 CDCl3
50.93
38.14
30.00
28.77
27.97
26.39
CO2Me
1a
Analysis: 13C NMR
Solvent: CDCl3
Field strength: 101 MHz
190 180 170 160 150 140 130 120 110 100 90 80 70 60 50 40 30 20 10 0
f1 (ppm)

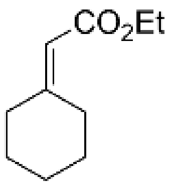

1b

Analysis: ^{1}H NMR
Solvent: $CDCl_3$
Field strength: 400 MHz

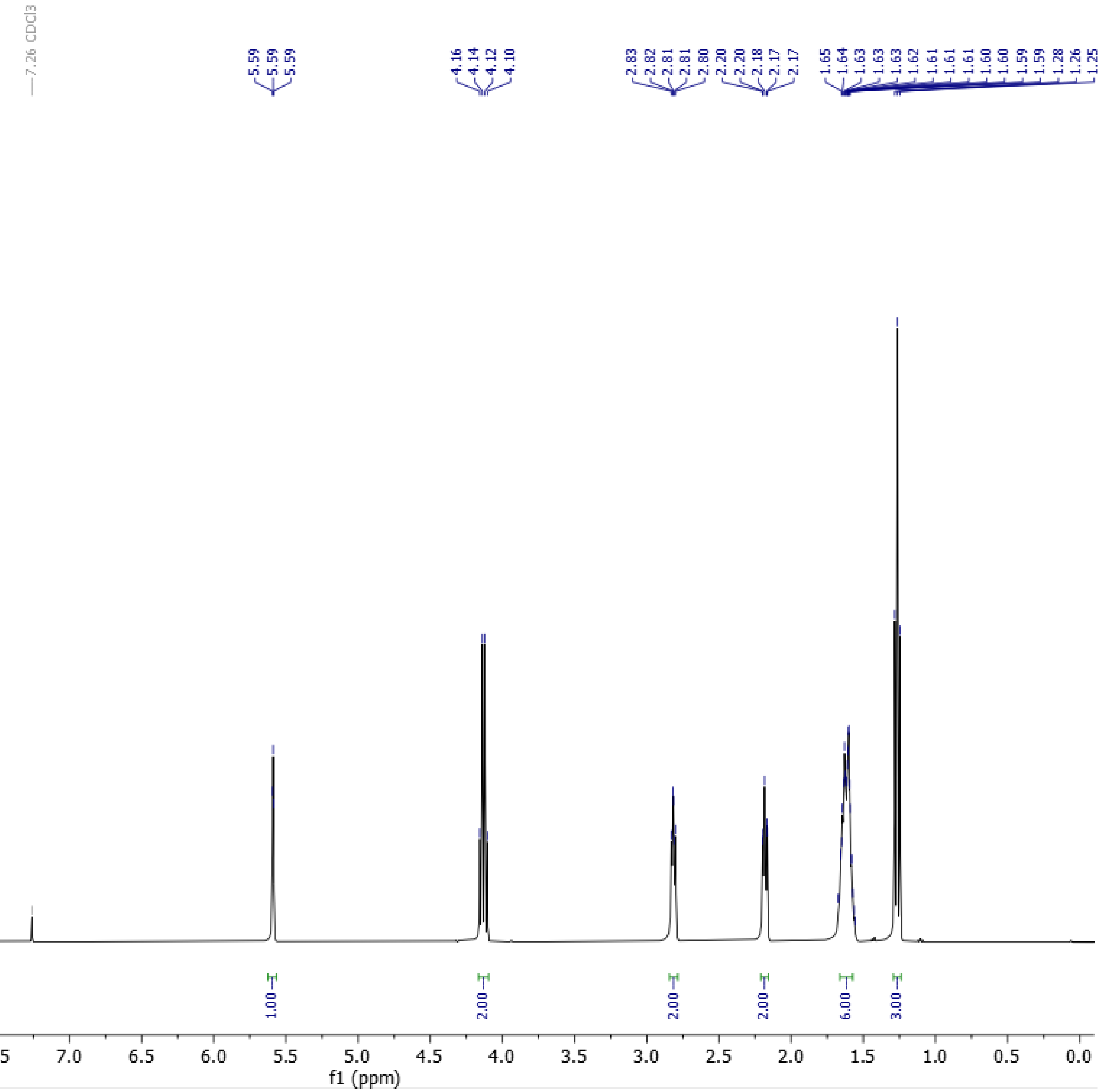

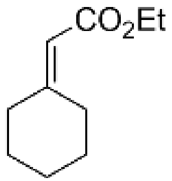

1b

Analysis: ^{13}C NMR
Solvent: $CDCl_3$
Field strength: 101 MHz

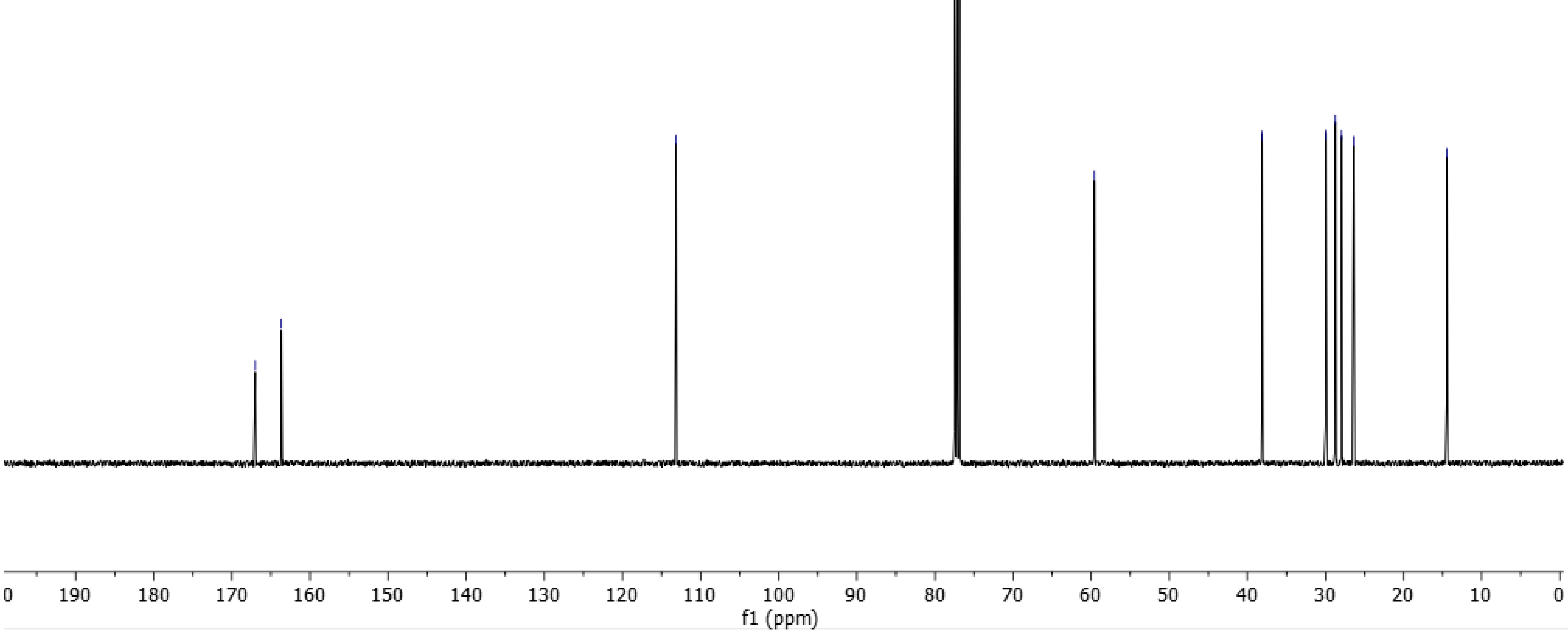

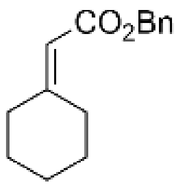

1c

Analysis: ^{1}H NMR
Solvent: $CDCl_3$
Field strength: 400 MHz

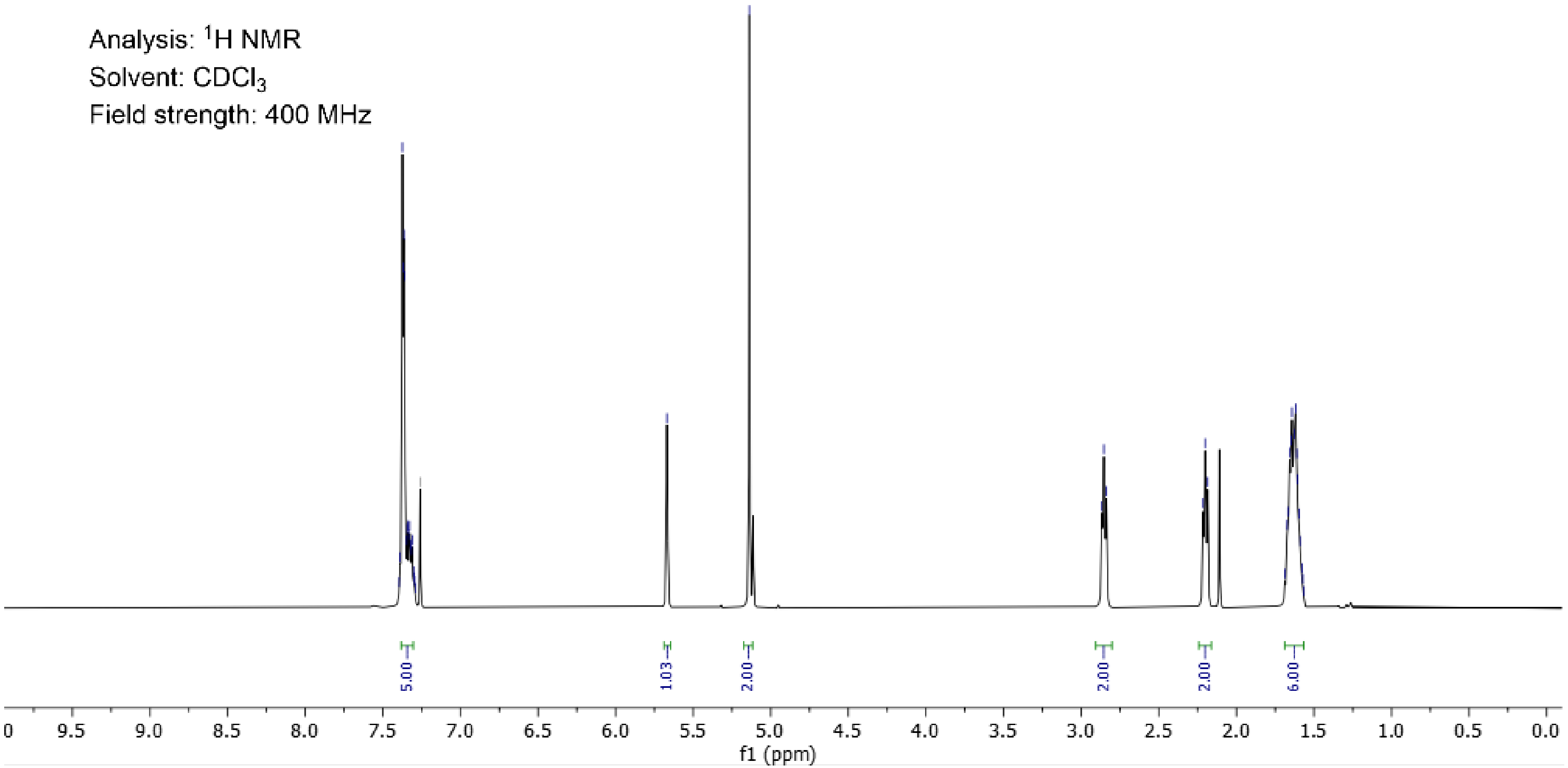

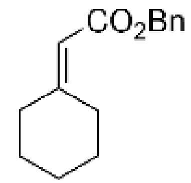

1c

Analysis: ^{13}C NMR
Solvent: $CDCl_3$
Field strength: 101 MHz

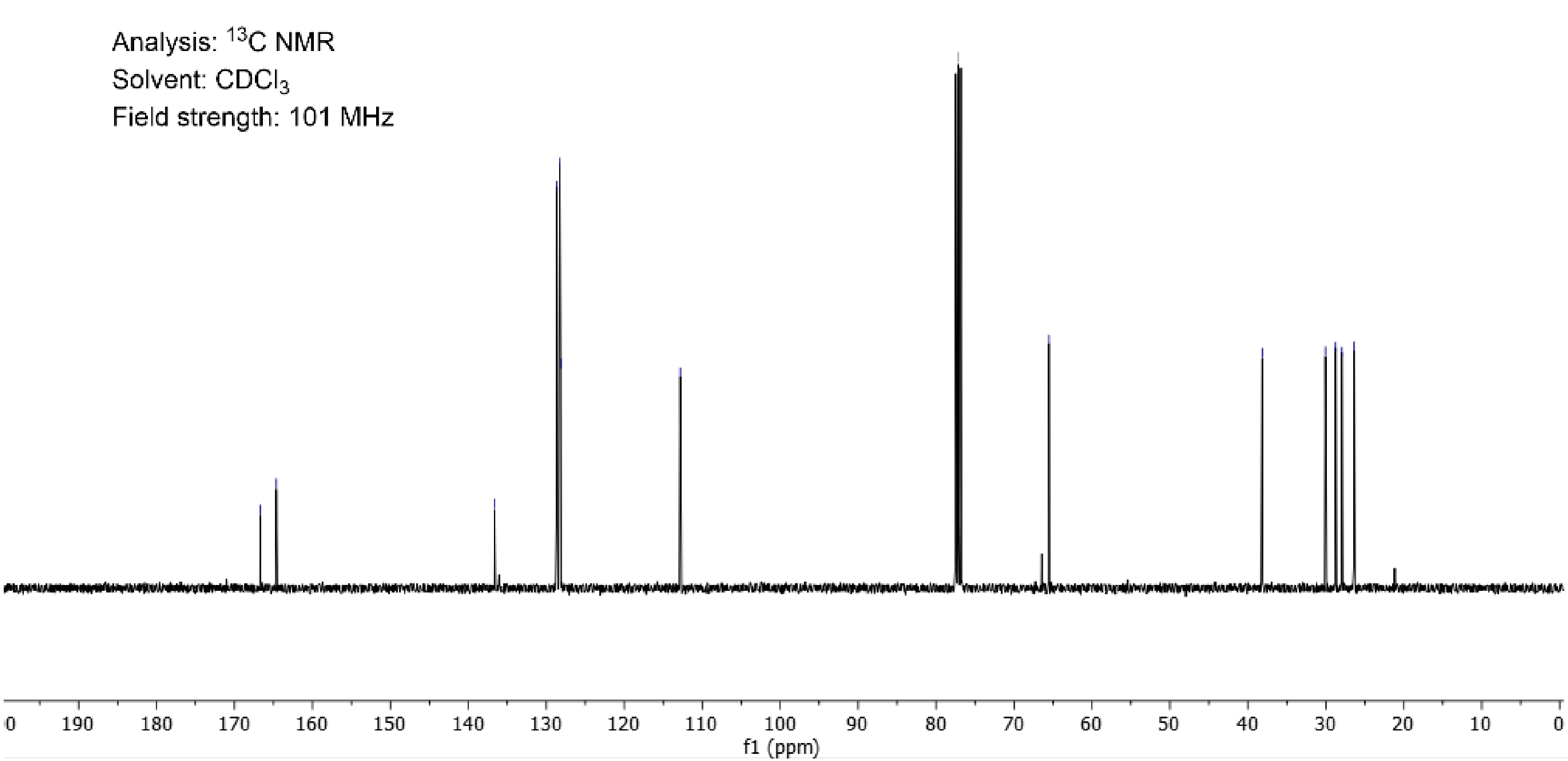

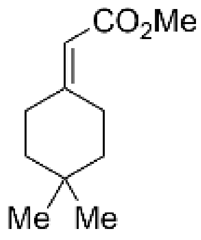

1d

Analysis: ^{1}H NMR
Solvent: $CDCl_3$
Field strength: 400 MHz

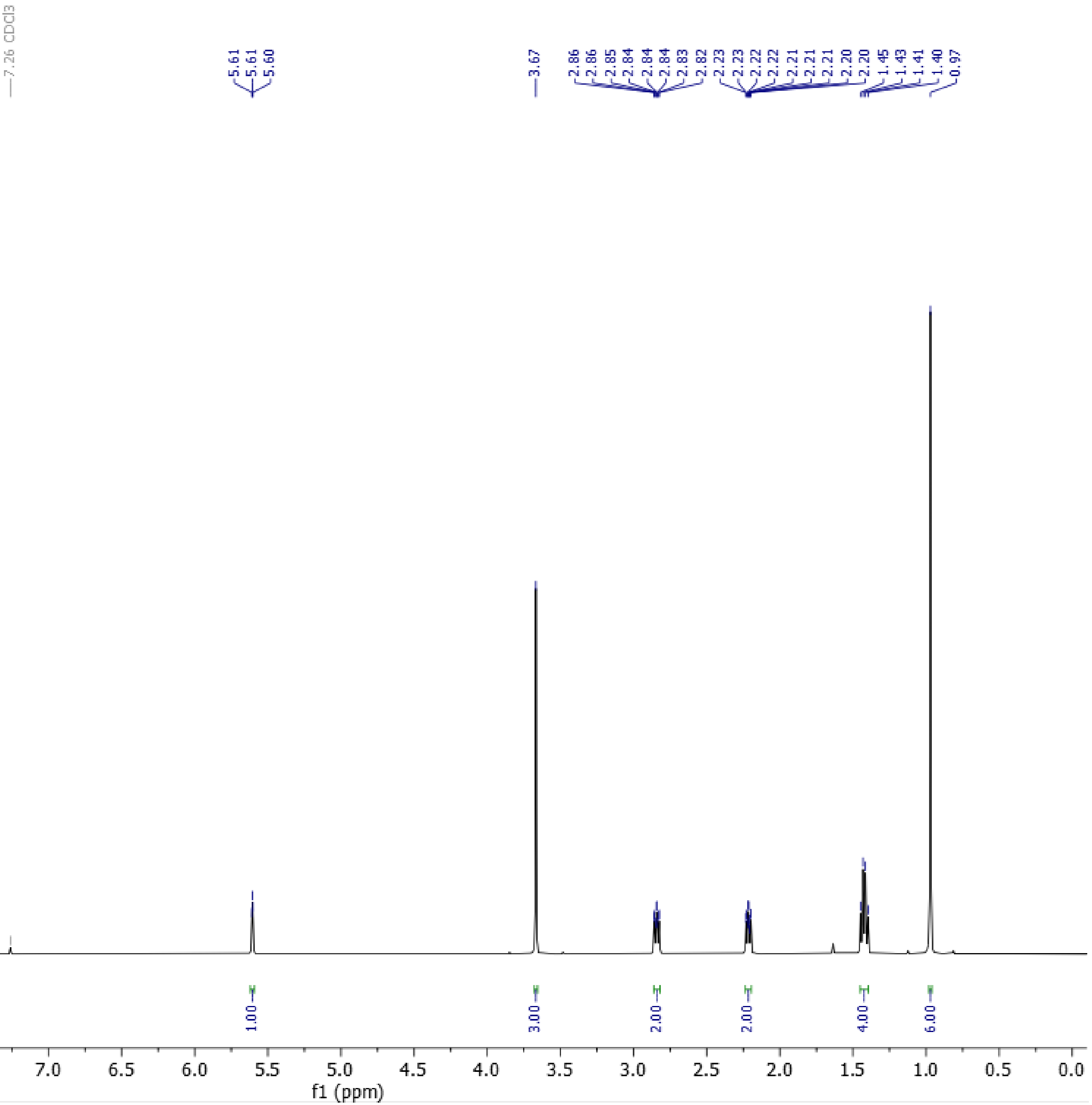

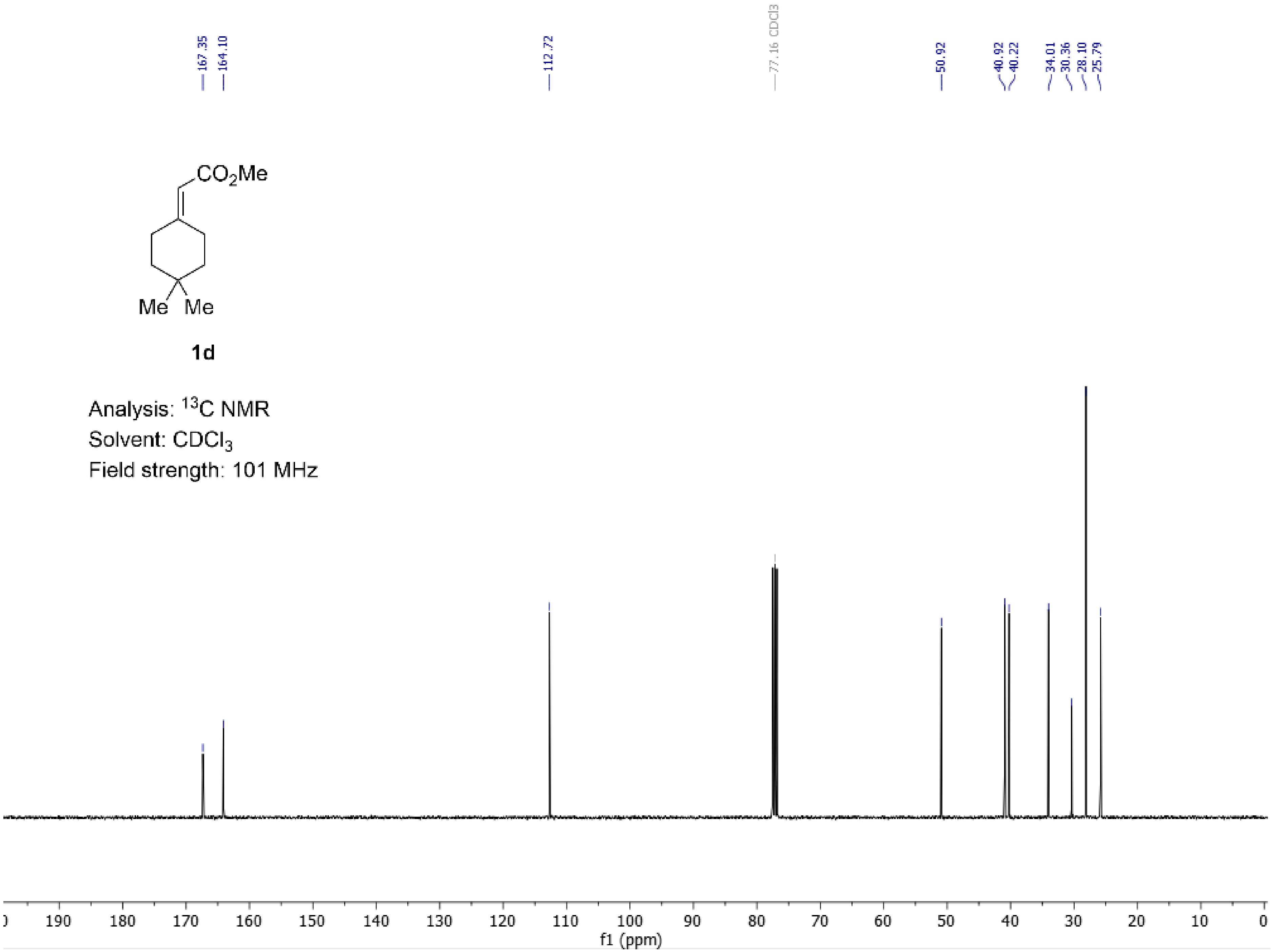
167.35
164.10
112.72
77.16 CDCl3
50.92
40.92
40.22
34.01
30.36
28.10
25.79
CO2Me
Me
Me
1d
Analysis: 13C NMR
Solvent: CDCl3
Field strength: 101 MHz
190
180
170
160
150
140
130
120
110
100
90
80
70
60
50
40
30
20
10
0
f1 (ppm)

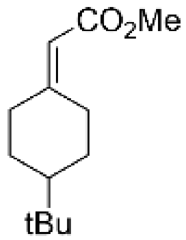

1e

Analysis: ^{1}H NMR
Solvent: $CDCl_3$
Field strength: 400 MHz

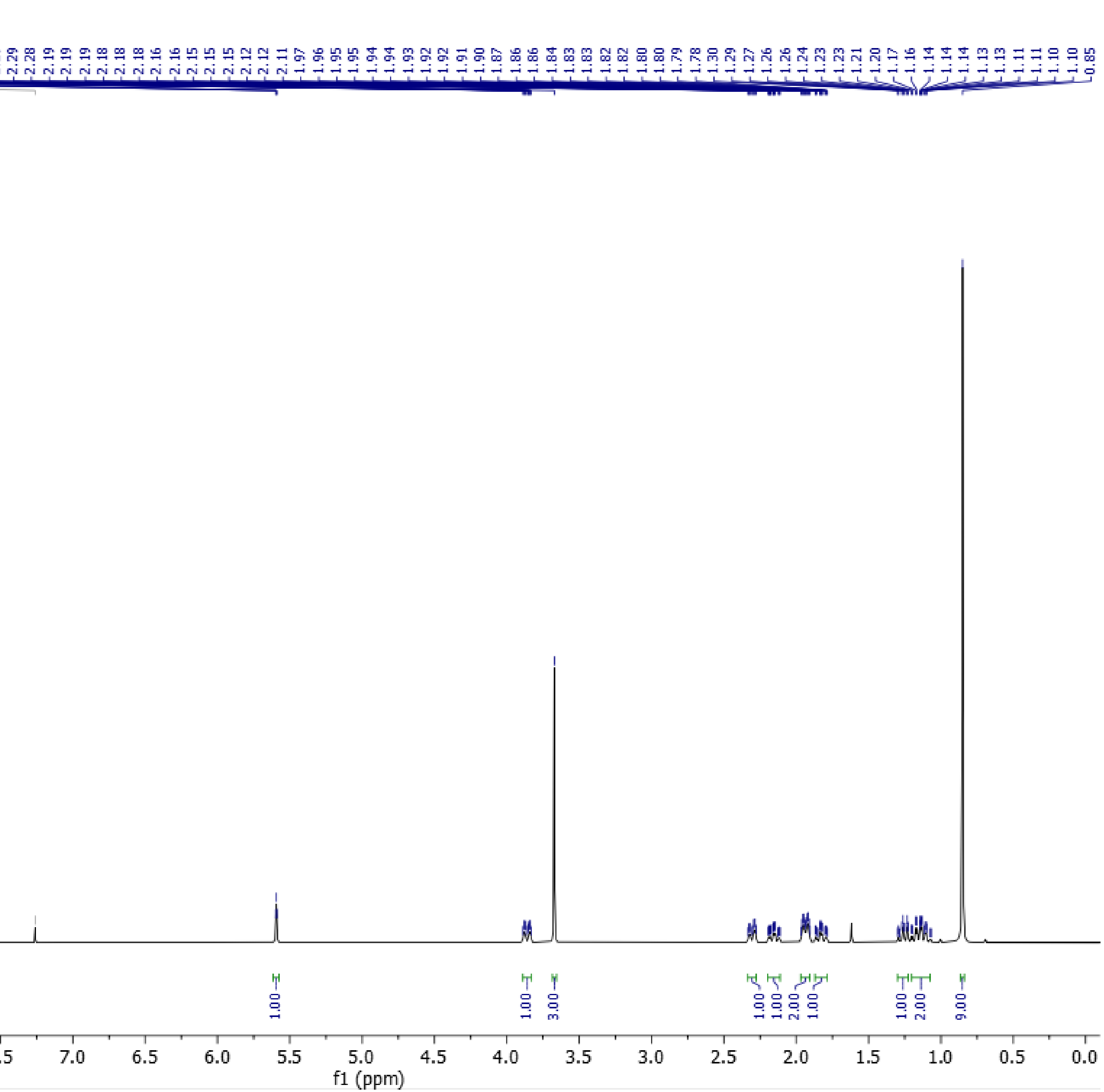

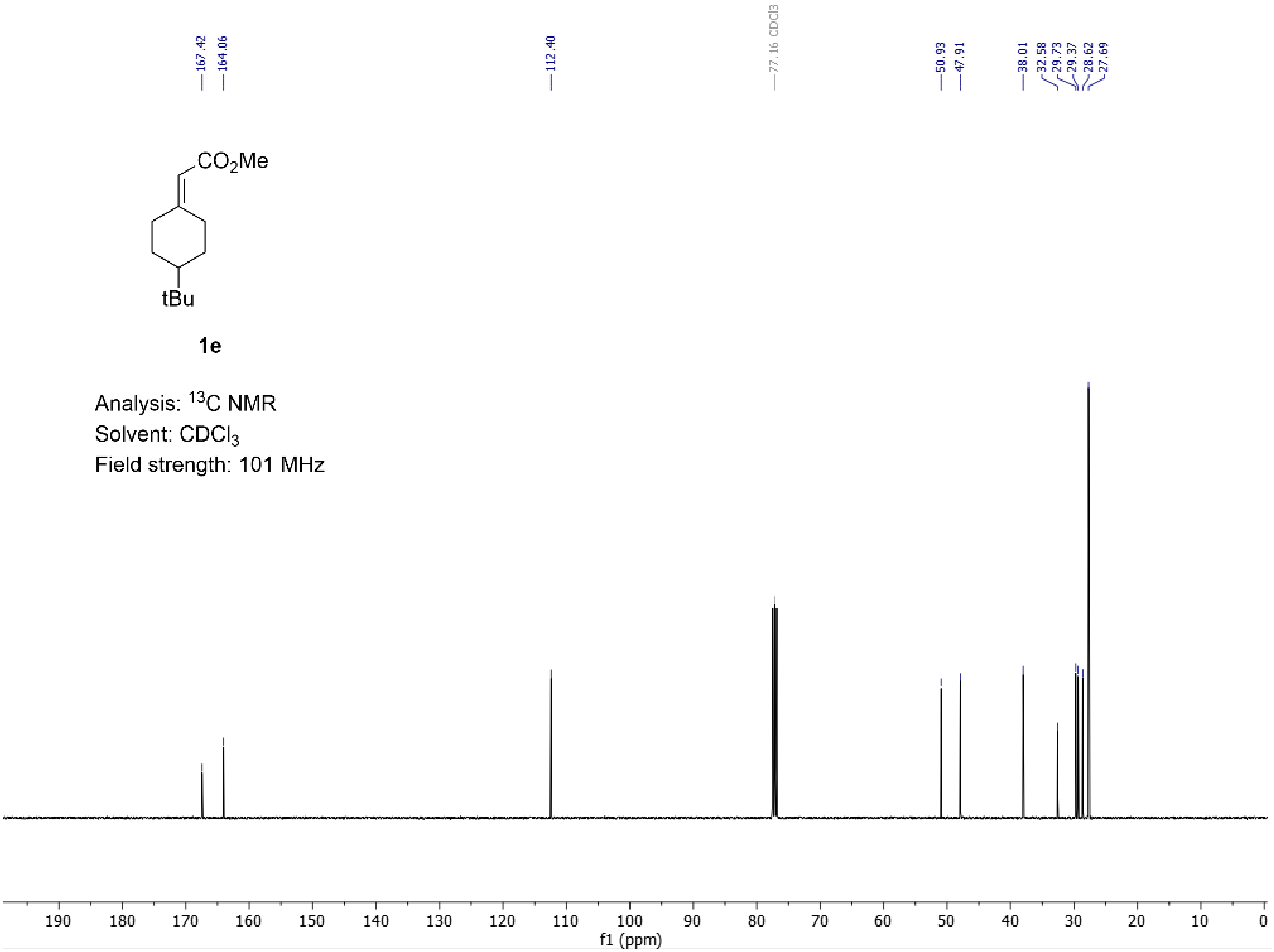

167.42
164.06
112.40
77.16 CDCl3
50.93
47.91
38.01
32.58
29.73
29.37
28.62
27.69
CO2Me
tBu
1e
Analysis: 13C NMR
Solvent: CDCl3
Field strength: 101 MHz
190 180 170 160 150 140 130 120 110 100 90 80 70 60 50 40 30 20 10 0
f1 (ppm)

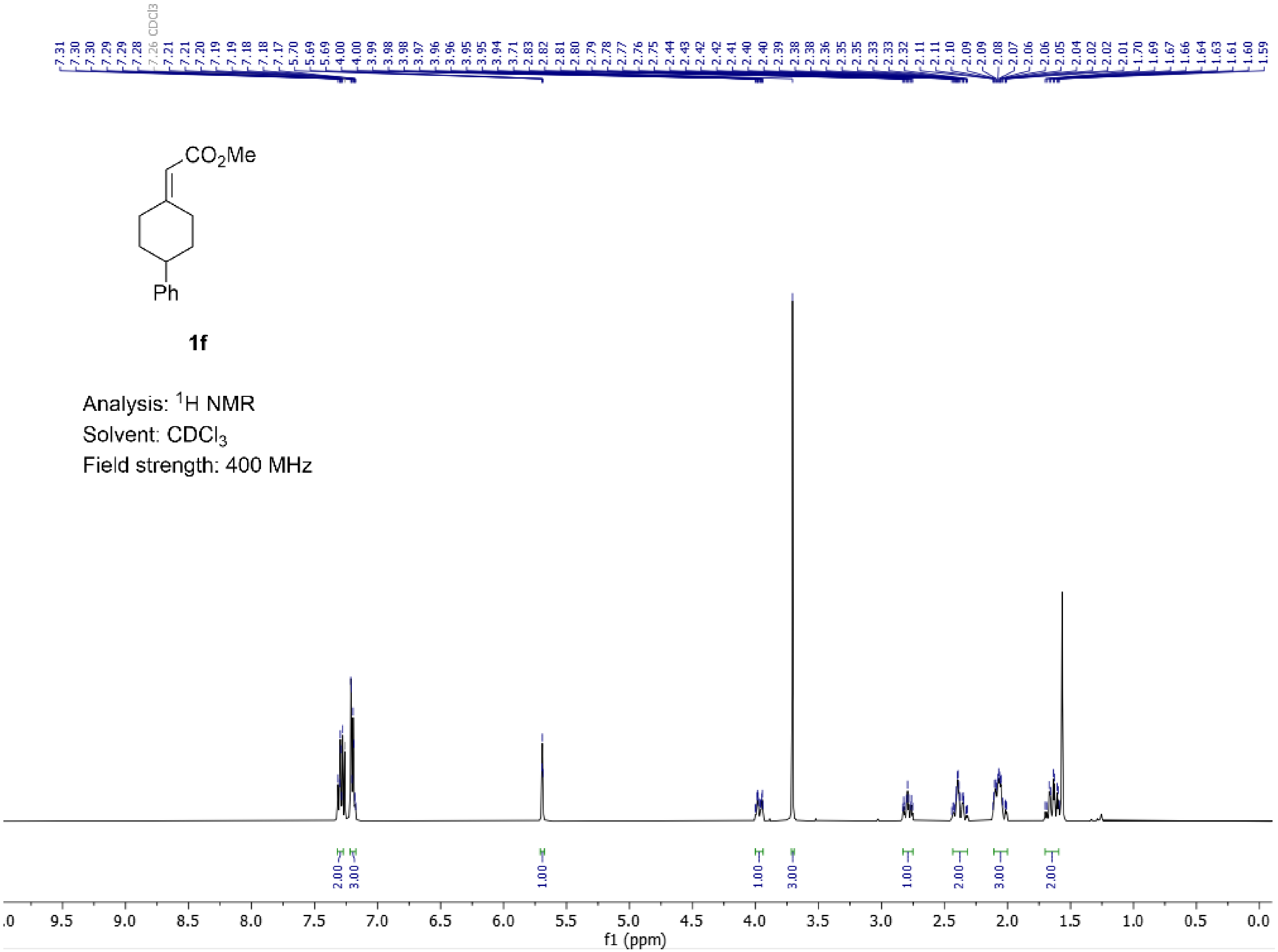
7.31 7.30 7.30 7.29 7.29 7.28 7.26 CDCl3 7.21 7.21 7.20 7.19 7.19 7.18 7.18 7.17 5.70 5.69 5.69 4.00 4.00 3.99 3.98 3.98 3.97 3.96 3.96 3.95 3.95 3.94 3.71 2.83 2.82 2.81 2.80 2.79 2.78 2.77 2.76 2.75 2.44 2.43 2.42 2.42 2.41 2.40 2.40 2.39 2.38 2.38 2.36 2.35 2.35 2.33 2.33 2.32 2.11 2.11 2.10 2.09 2.09 2.08 2.07 2.06 2.06 2.05 2.04 2.02 2.02 2.01 1.70 1.69 1.67 1.66 1.64 1.63 1.61 1.60 1.59
CO2Me
Ph
1f
Analysis: 1H NMR
Solvent: CDCl3
Field strength: 400 MHz
2.00 3.00 1.00 1.00 3.00 1.00 2.00 3.00 2.00
f1 (ppm)

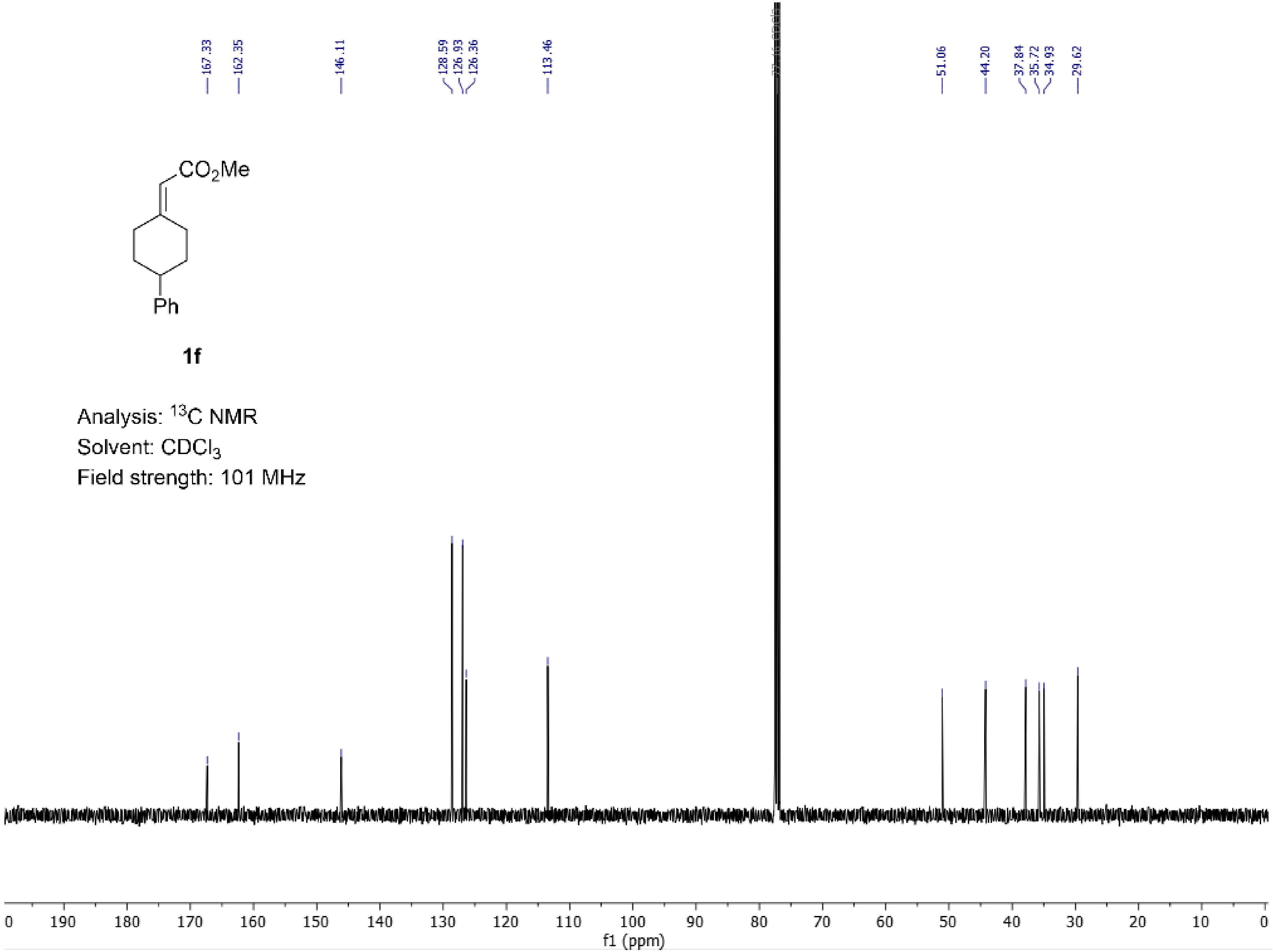

1f

Analysis: ^{13}C NMR
Solvent: $CDCl_3$
Field strength: 101 MHz

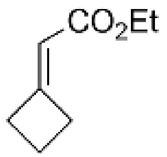

1g

Analysis: ^{1}H NMR
Solvent: $CDCl_3$
Field strength: 400 MHz

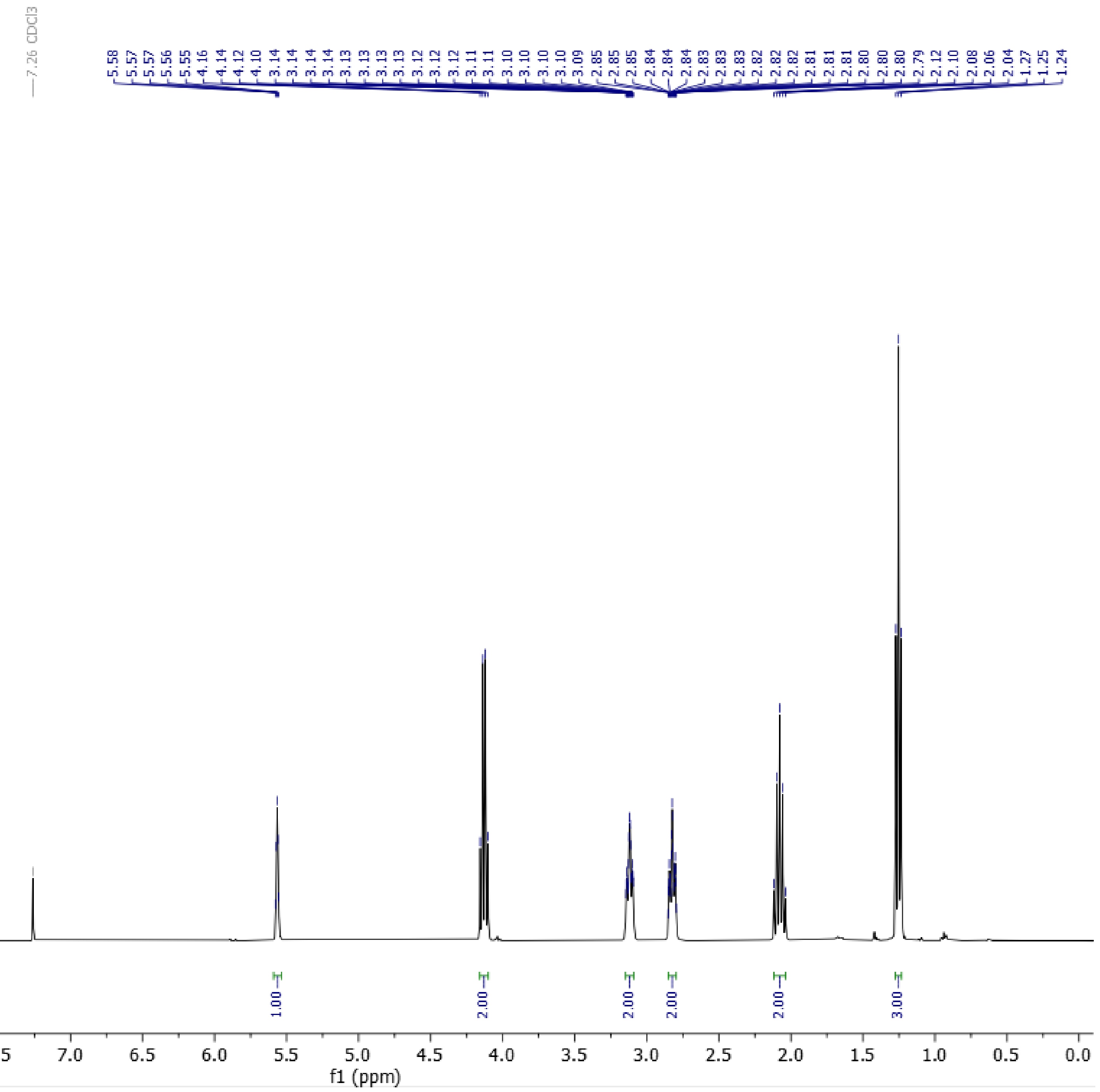

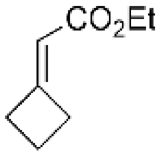

1g

Analysis: ^{13}C NMR
Solvent: $CDCl_3$
Field strength: 101 MHz

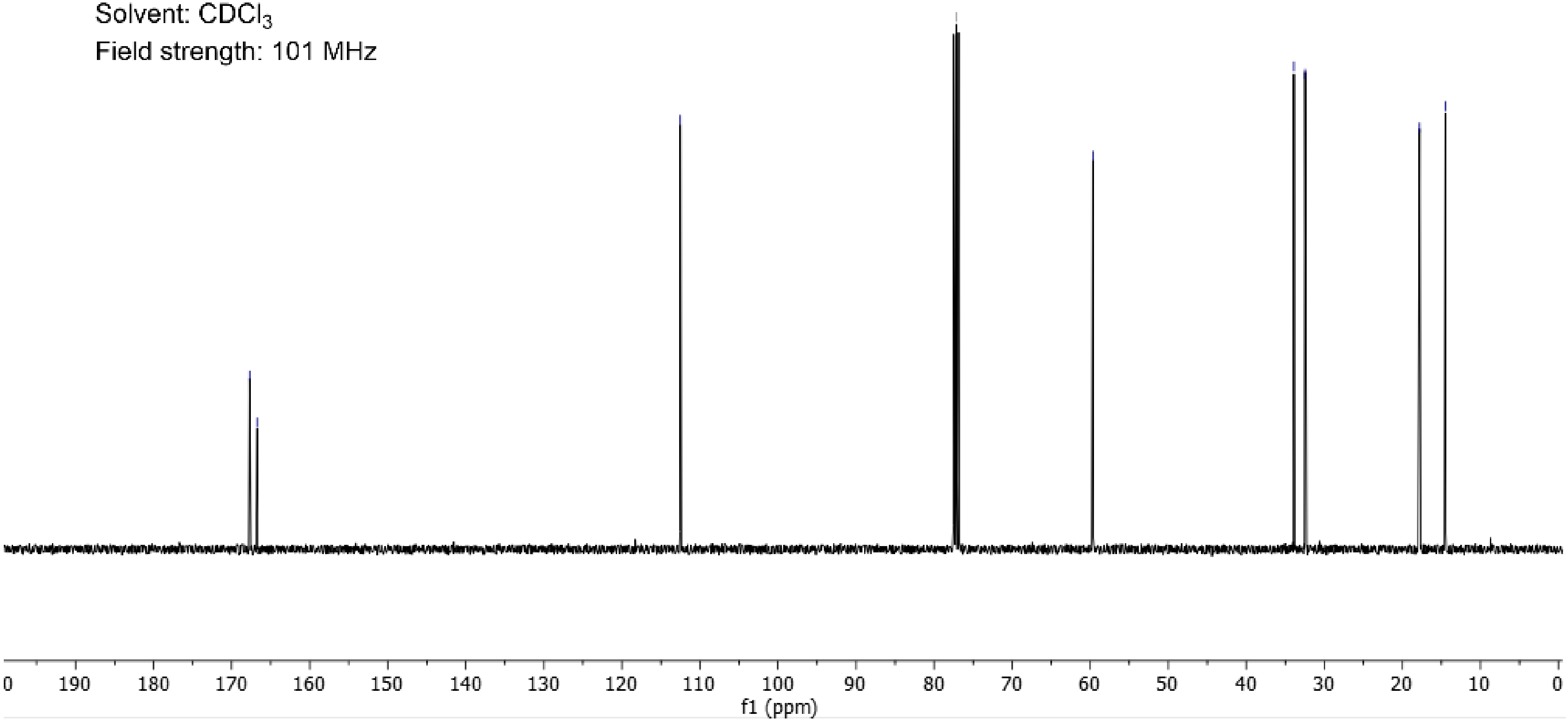

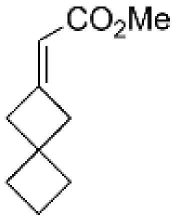

1h

Analysis: ^{1}H NMR
Solvent: $CDCl_3$
Field strength: 400 MHz

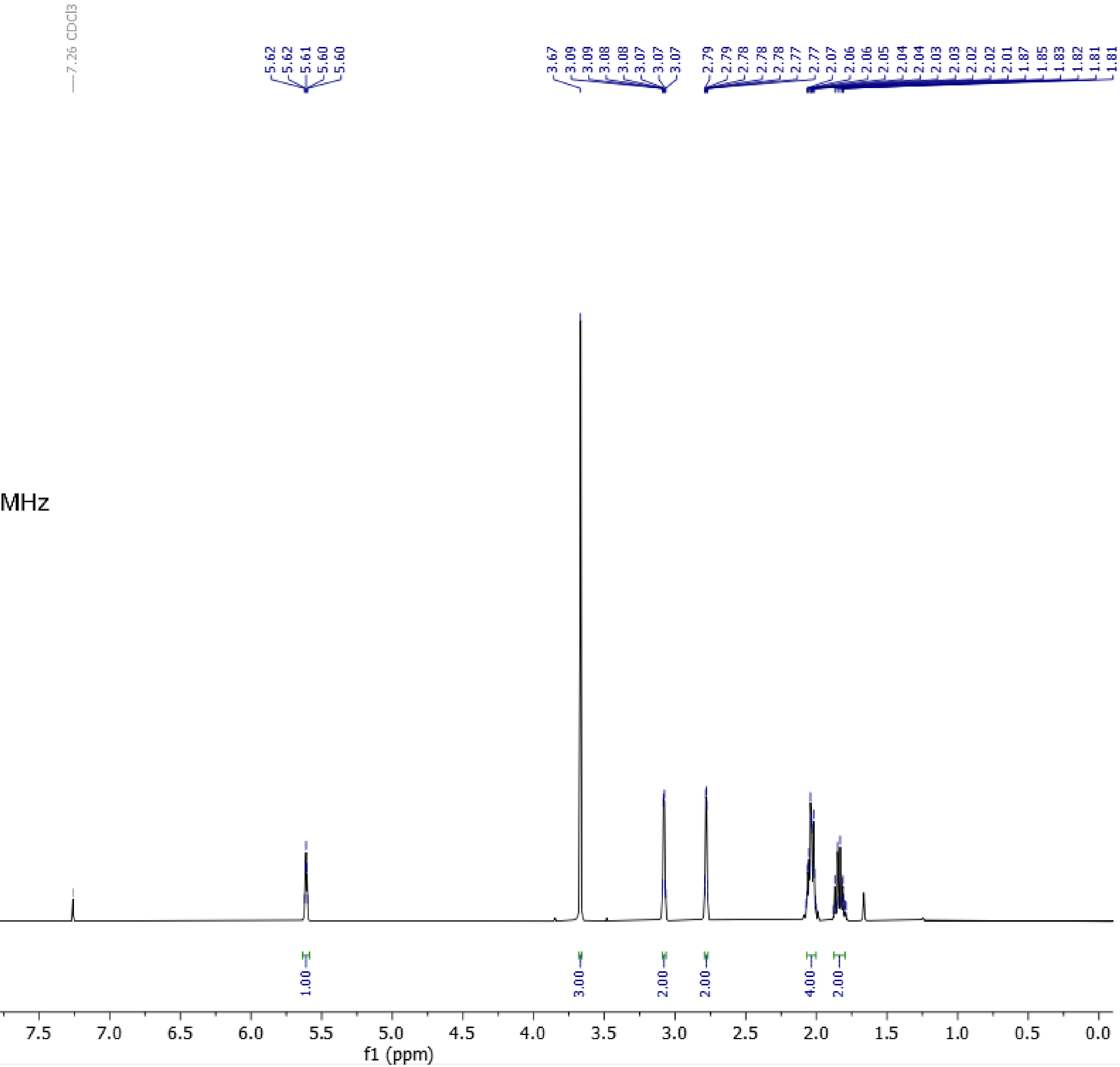

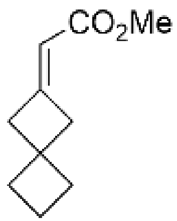

1h

Analysis: ^{13}C NMR
Solvent: $CDCl_3$
Field strength: 101 MHz

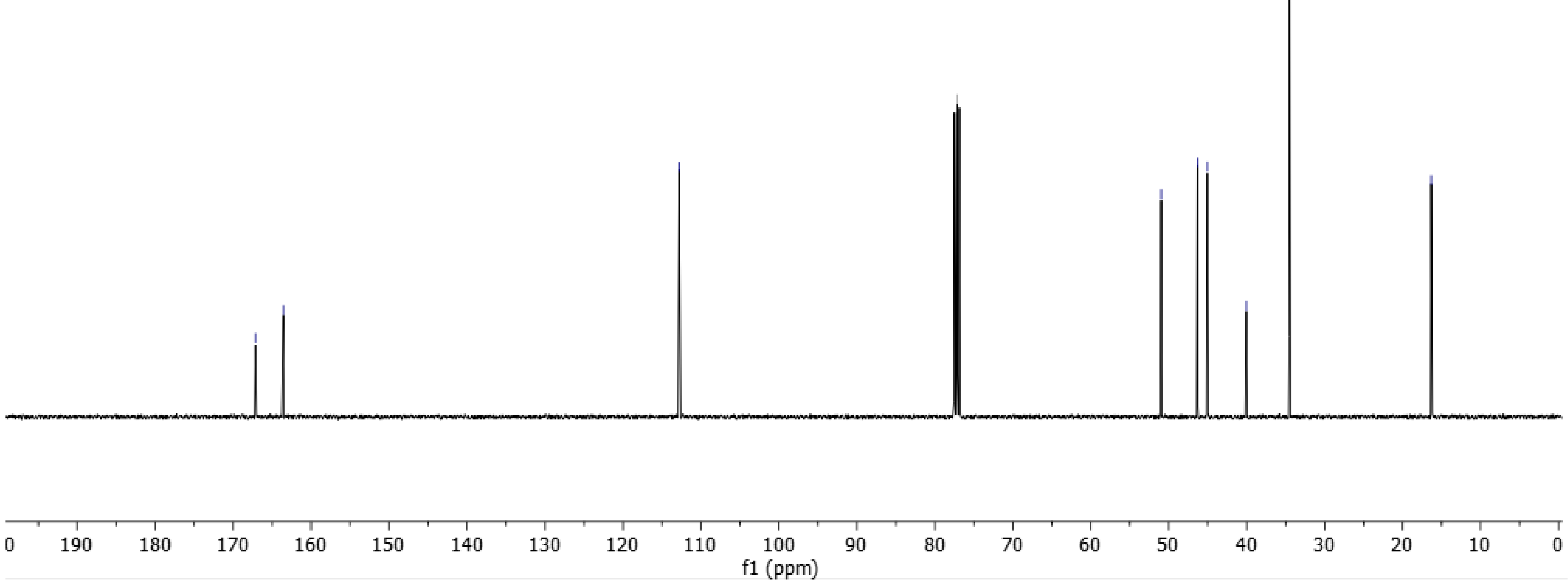

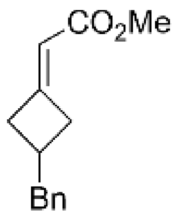

1i

Analysis: ^{1}H NMR
Solvent: $CDCl_3$
Field strength: 600 MHz

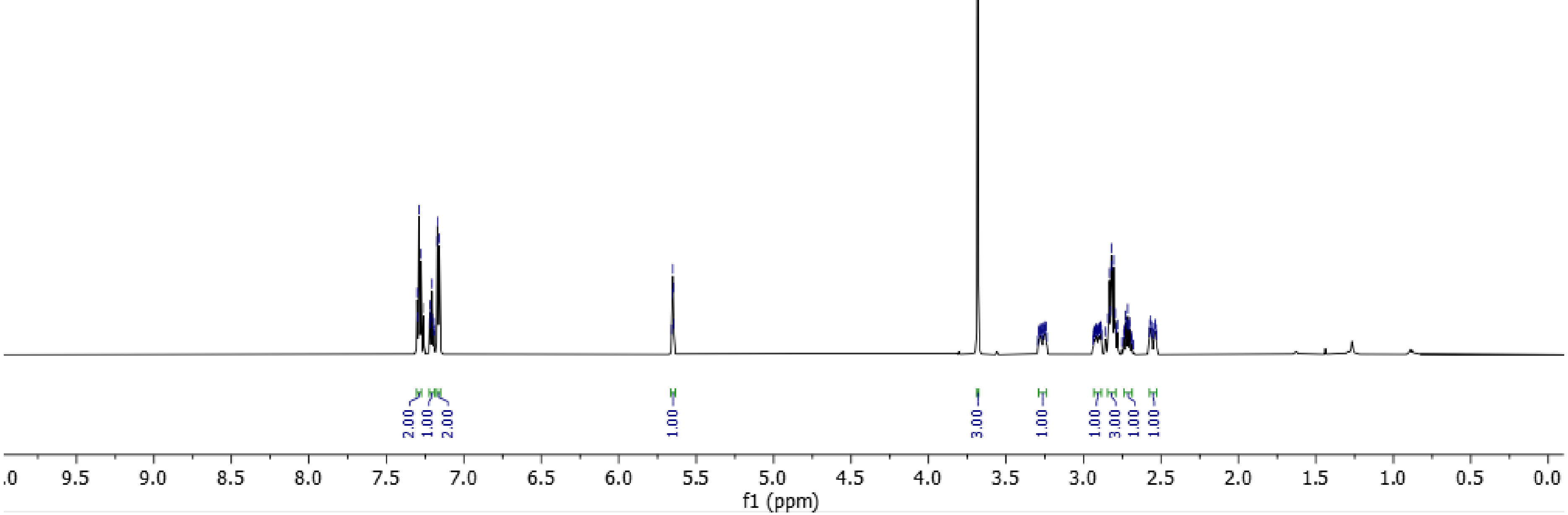

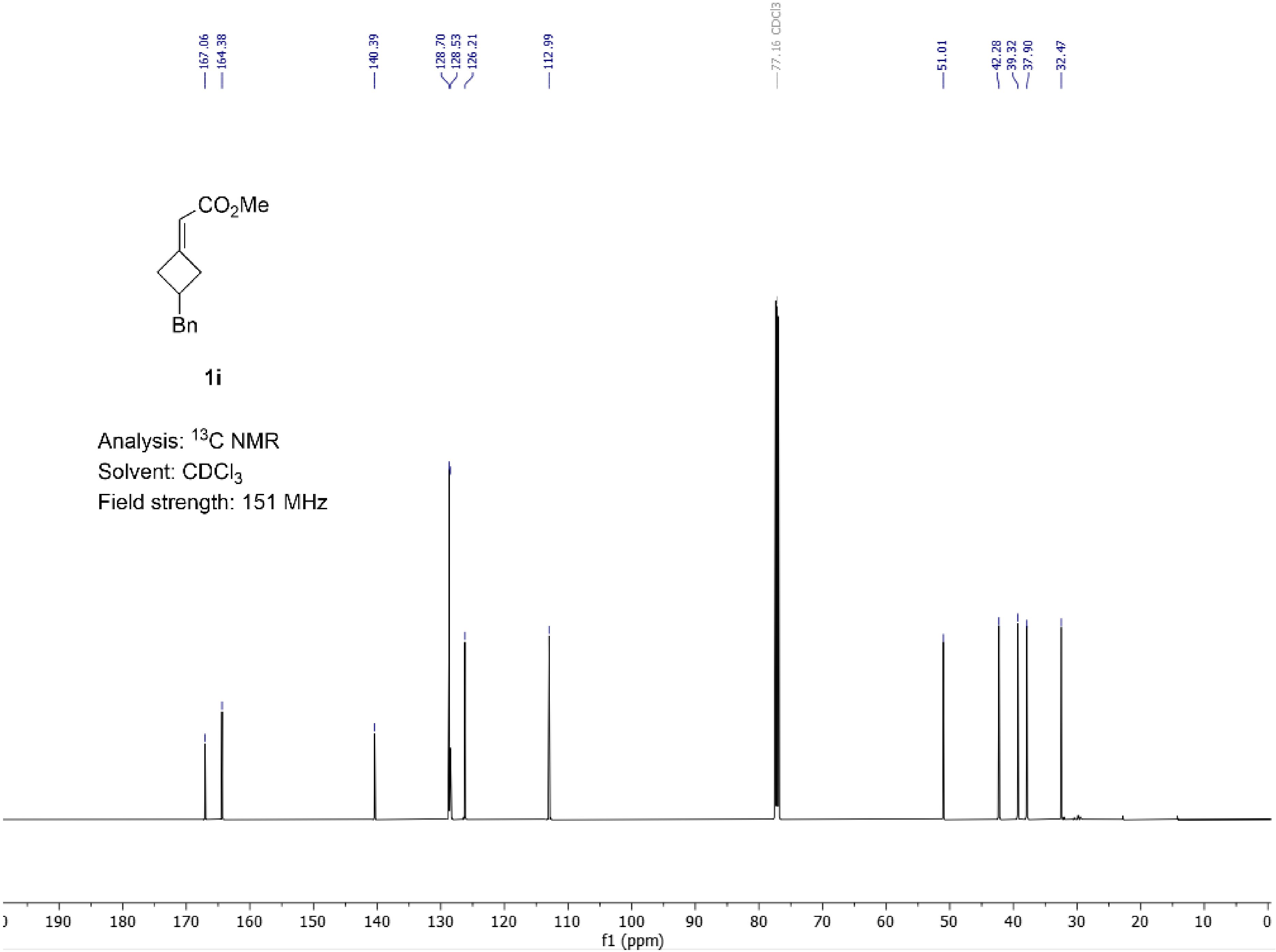
167.06
164.38
140.39
128.70
128.53
126.21
112.99
77.16 CDCl3
51.01
42.28
39.32
37.90
32.47
CO2Me
Bn
1i
Analysis: 13C NMR
Solvent: CDCl3
Field strength: 151 MHz
190 180 170 160 150 140 130 120 110 100 90 80 70 60 50 40 30 20 10 0
f1 (ppm)

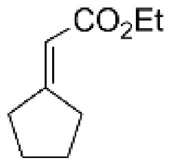

1j

Analysis: ^{1}H NMR
Solvent: $CDCl_3$
Field strength: 400 MHz

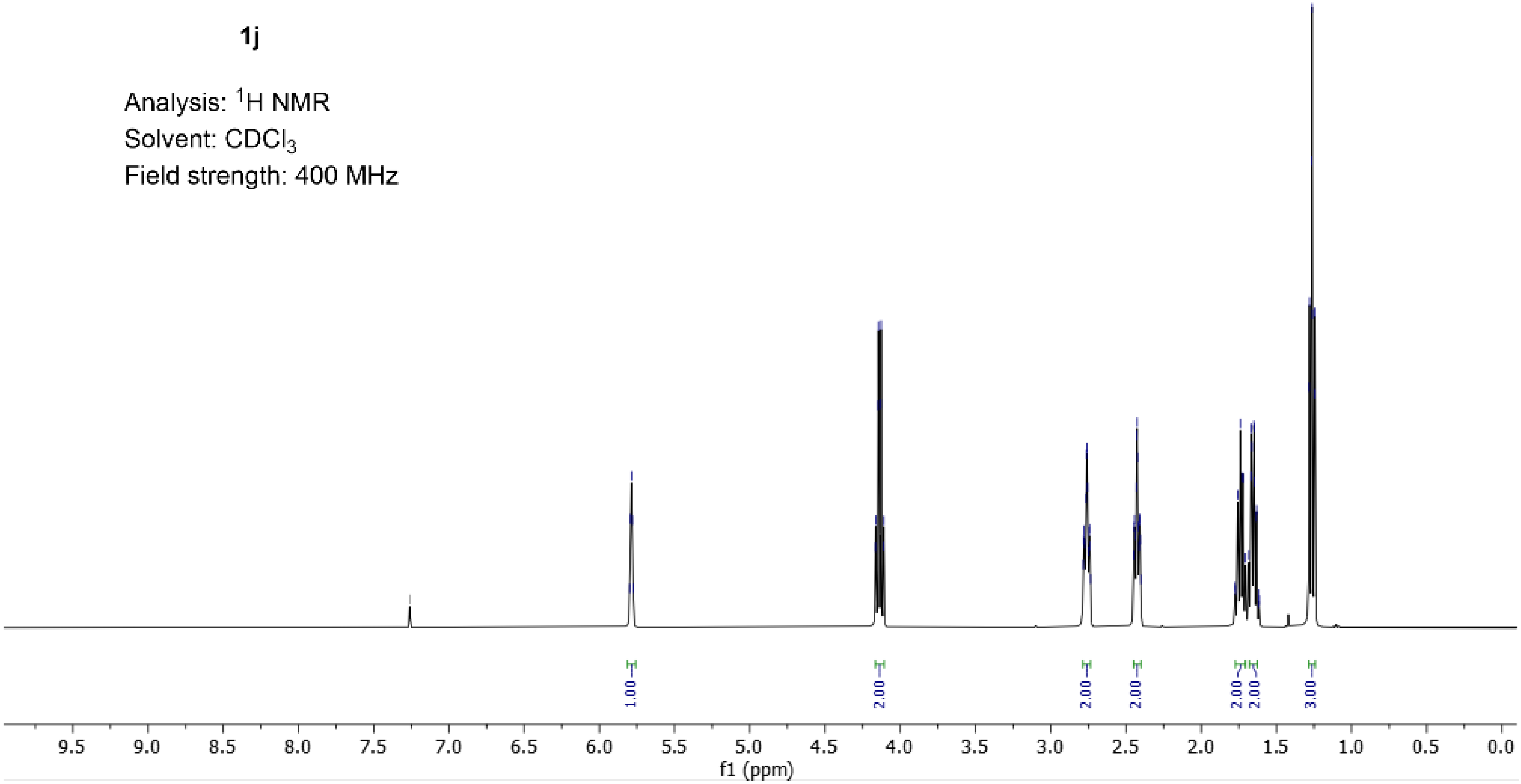

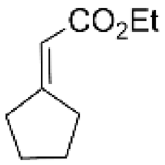

1j

Analysis: ^{13}C NMR
Solvent: $CDCl_3$
Field strength: 101 MHz

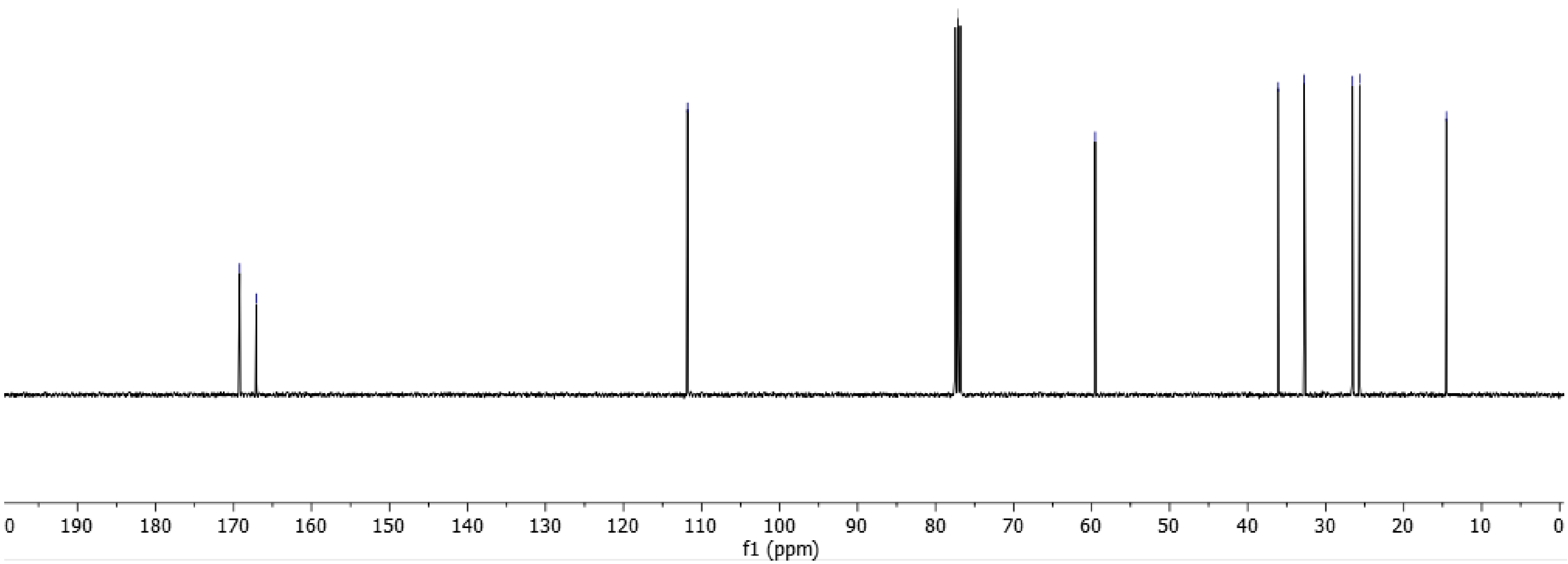

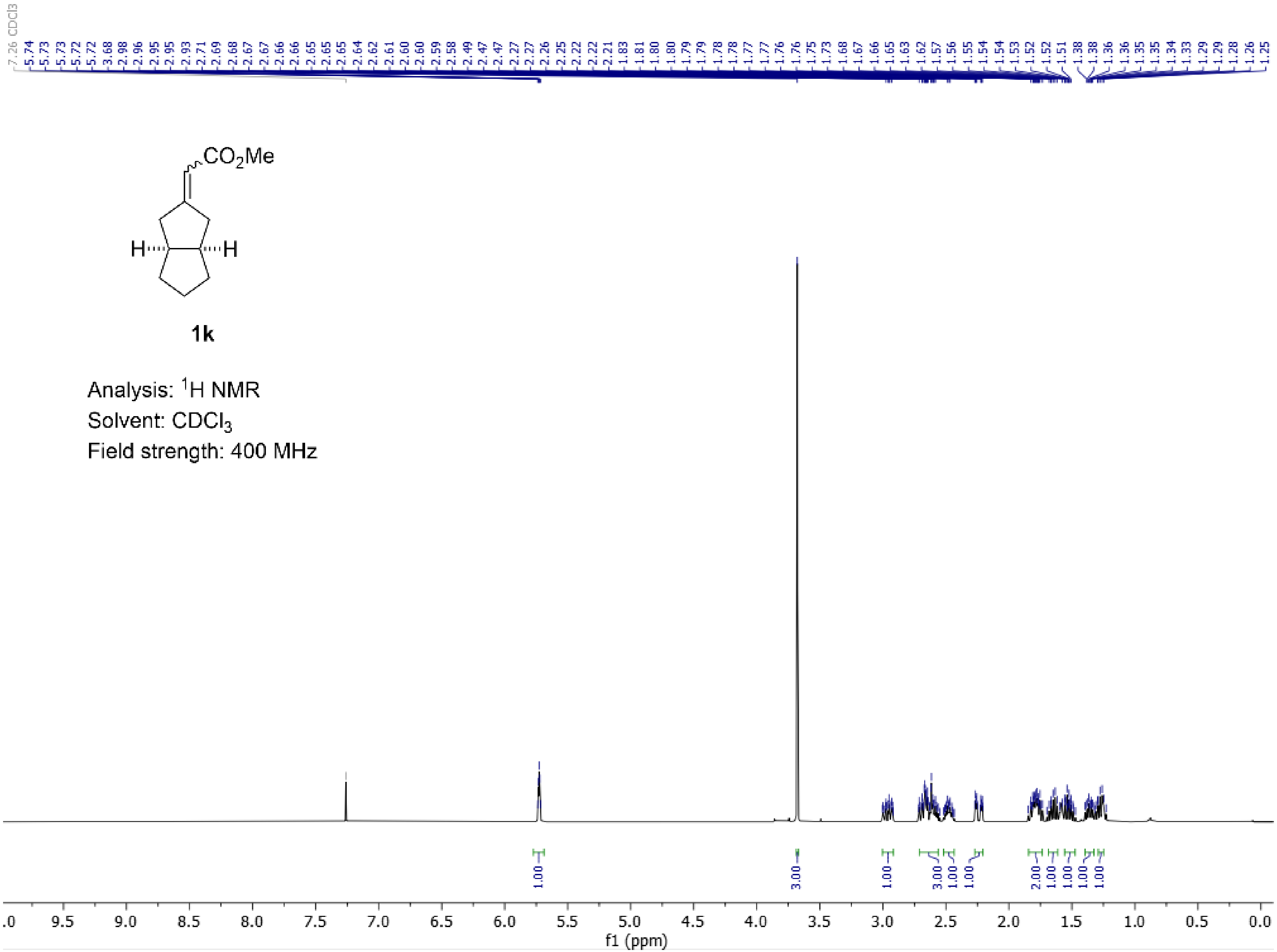
CO2Me
H
H
1k
Analysis: 1H NMR
Solvent: CDCl3
Field strength: 400 MHz
f1 (ppm)

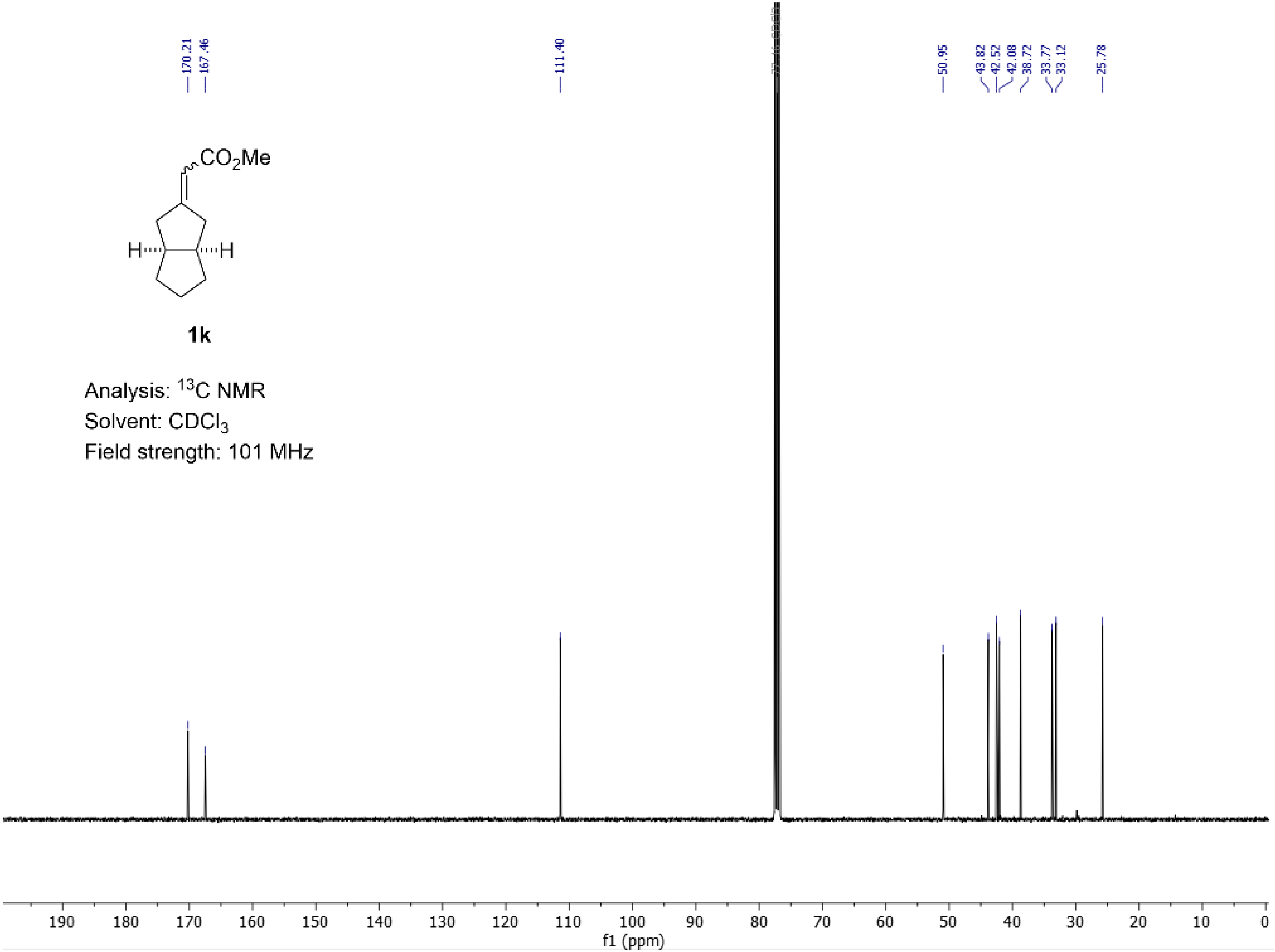

1k

Analysis: ^{13}C NMR
Solvent: $CDCl_3$
Field strength: 101 MHz

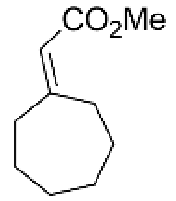

1l

Analysis: ^{1}H NMR
Solvent: $CDCl_3$
Field strength: 400 MHz

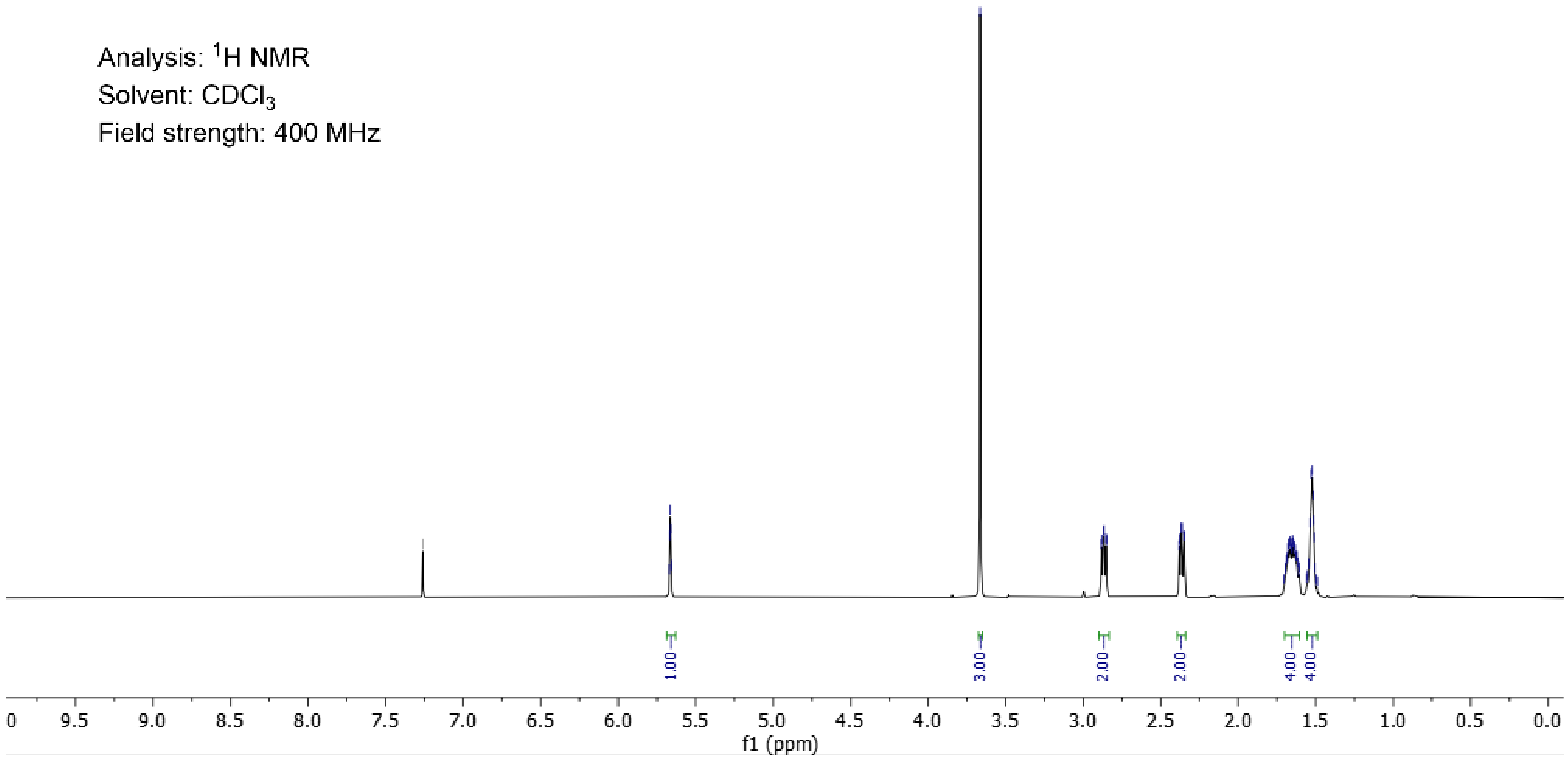

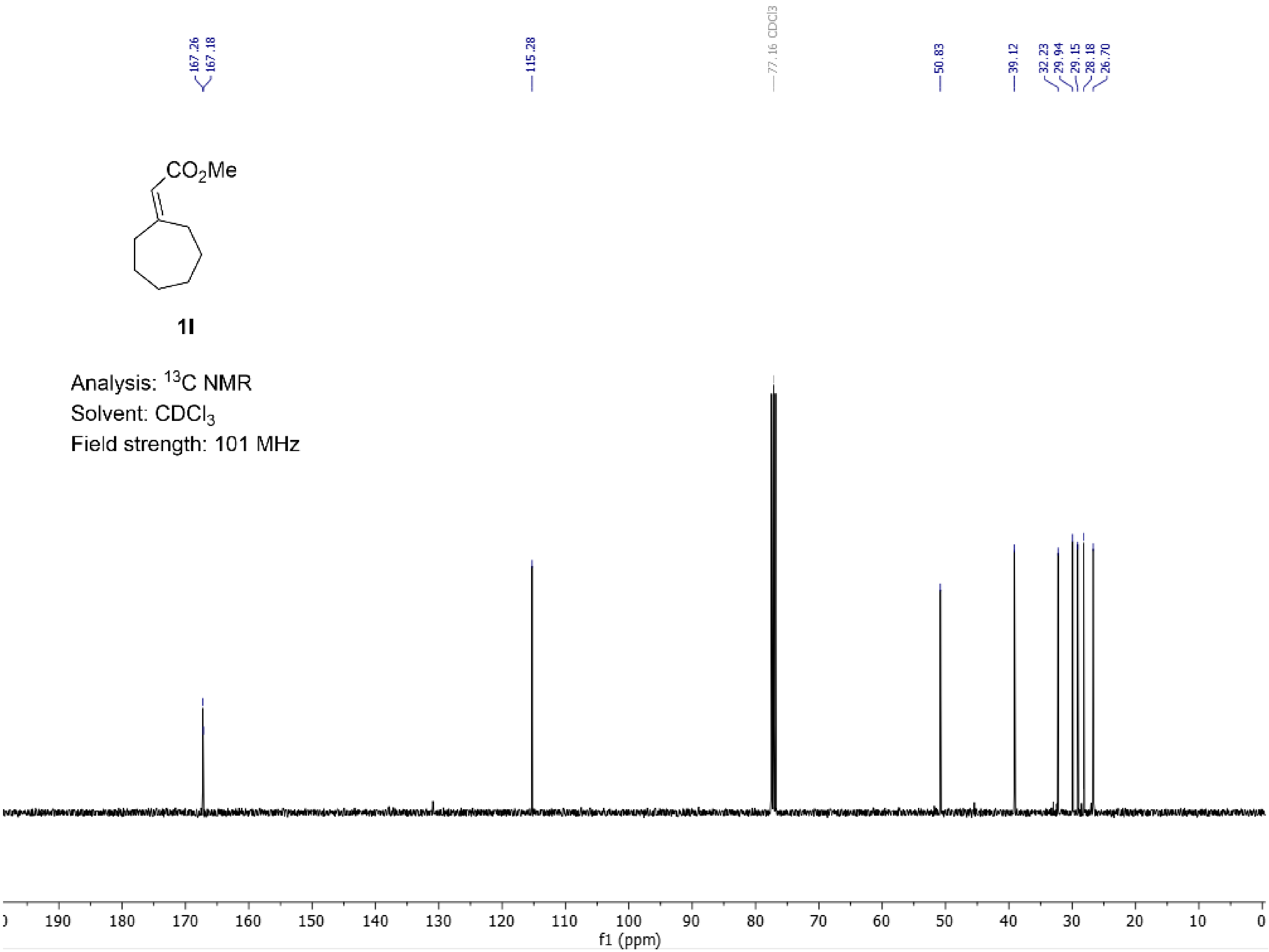
167.26
167.18
115.28
77.16 CDCl3
50.83
39.12
32.23
29.94
29.15
28.18
26.70
CO2Me
1l
Analysis: 13C NMR
Solvent: CDCl3
Field strength: 101 MHz
190
180
170
160
150
140
130
120
110
100
90
80
70
60
50
40
30
20
10
0
f1 (ppm)

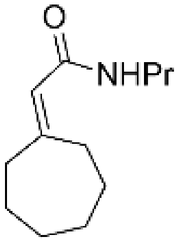

1m

Analysis: ^{1}H NMR
Solvent: $CDCl_3$
Field strength: 400 MHz

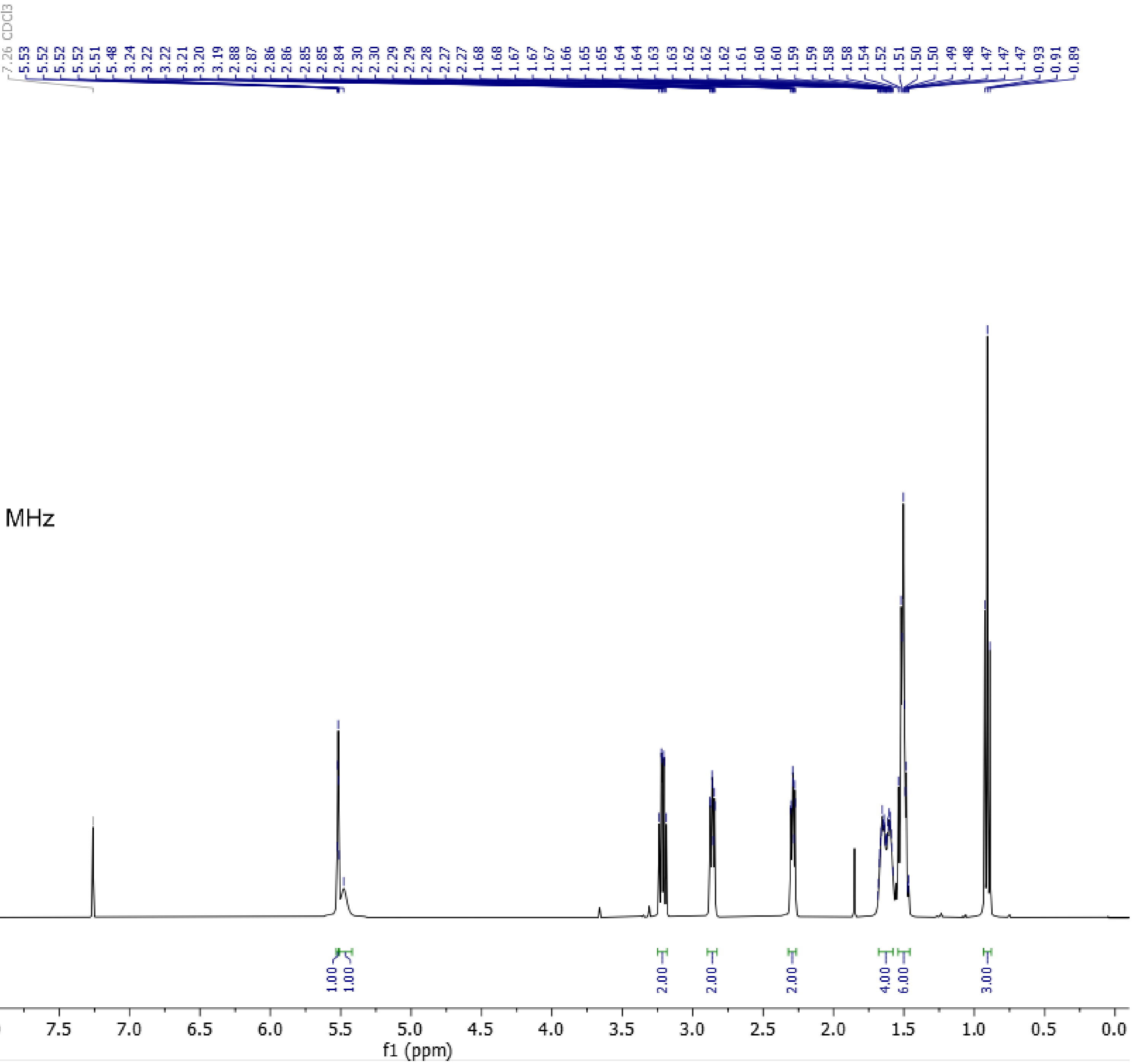

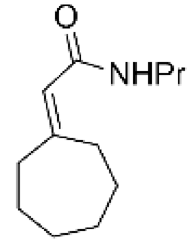

1m

Analysis: ^{13}C NMR
Solvent: $CDCl_3$
Field strength: 101 MHz

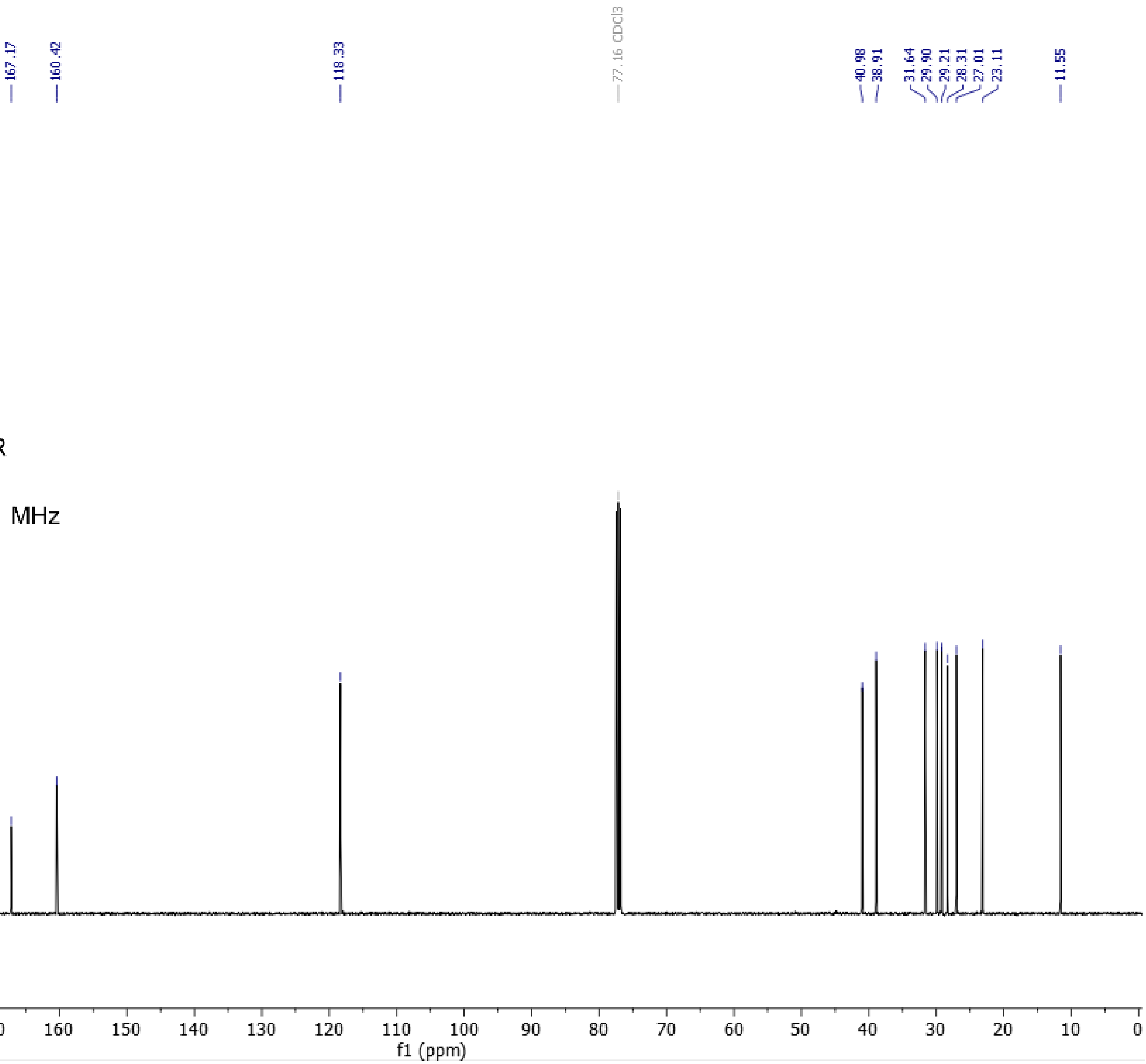

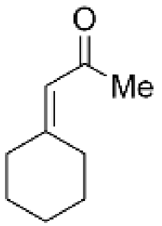

1n

Analysis: ^{1}H NMR
Solvent: $CDCl_3$
Field strength: 400 MHz

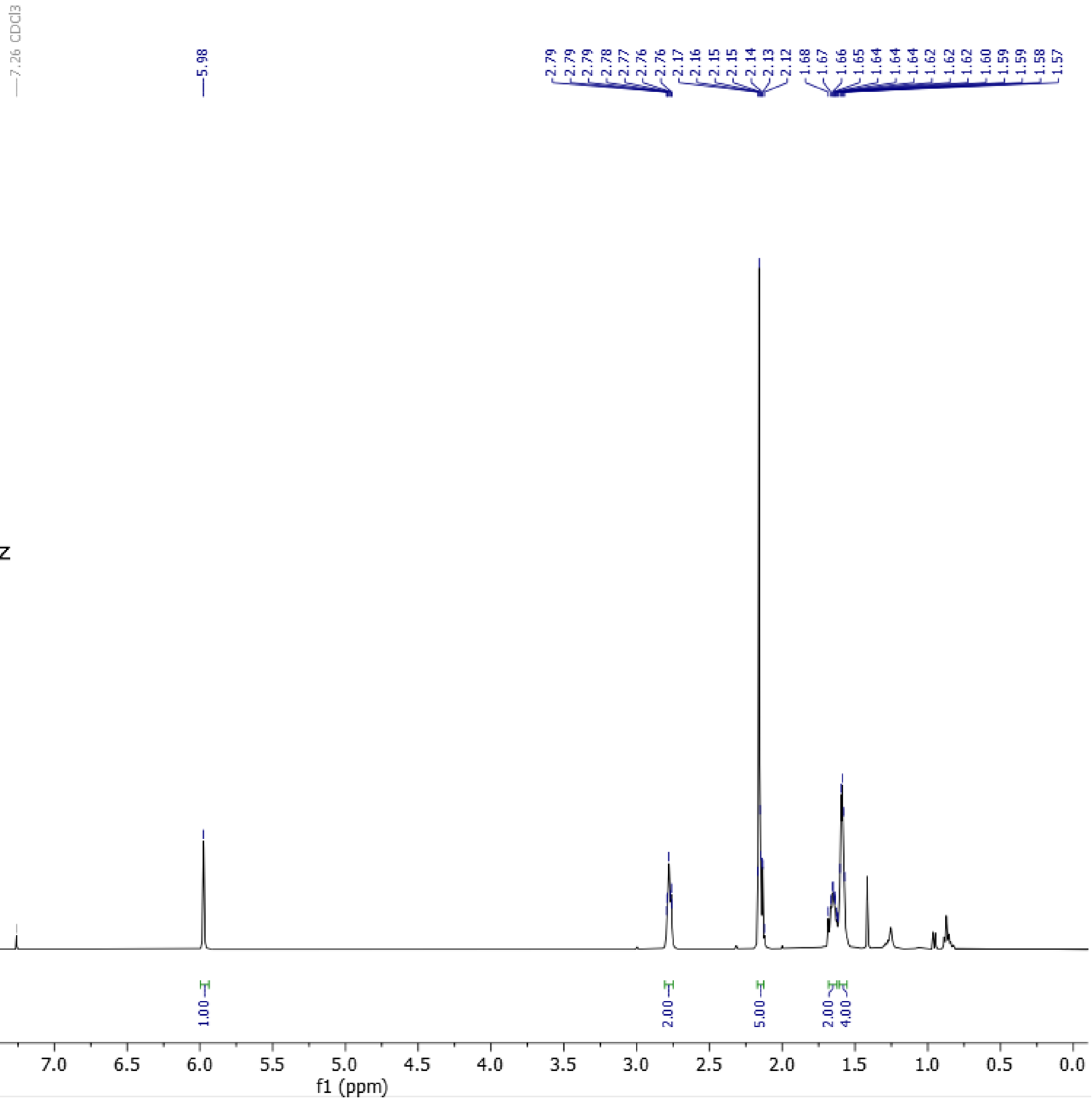

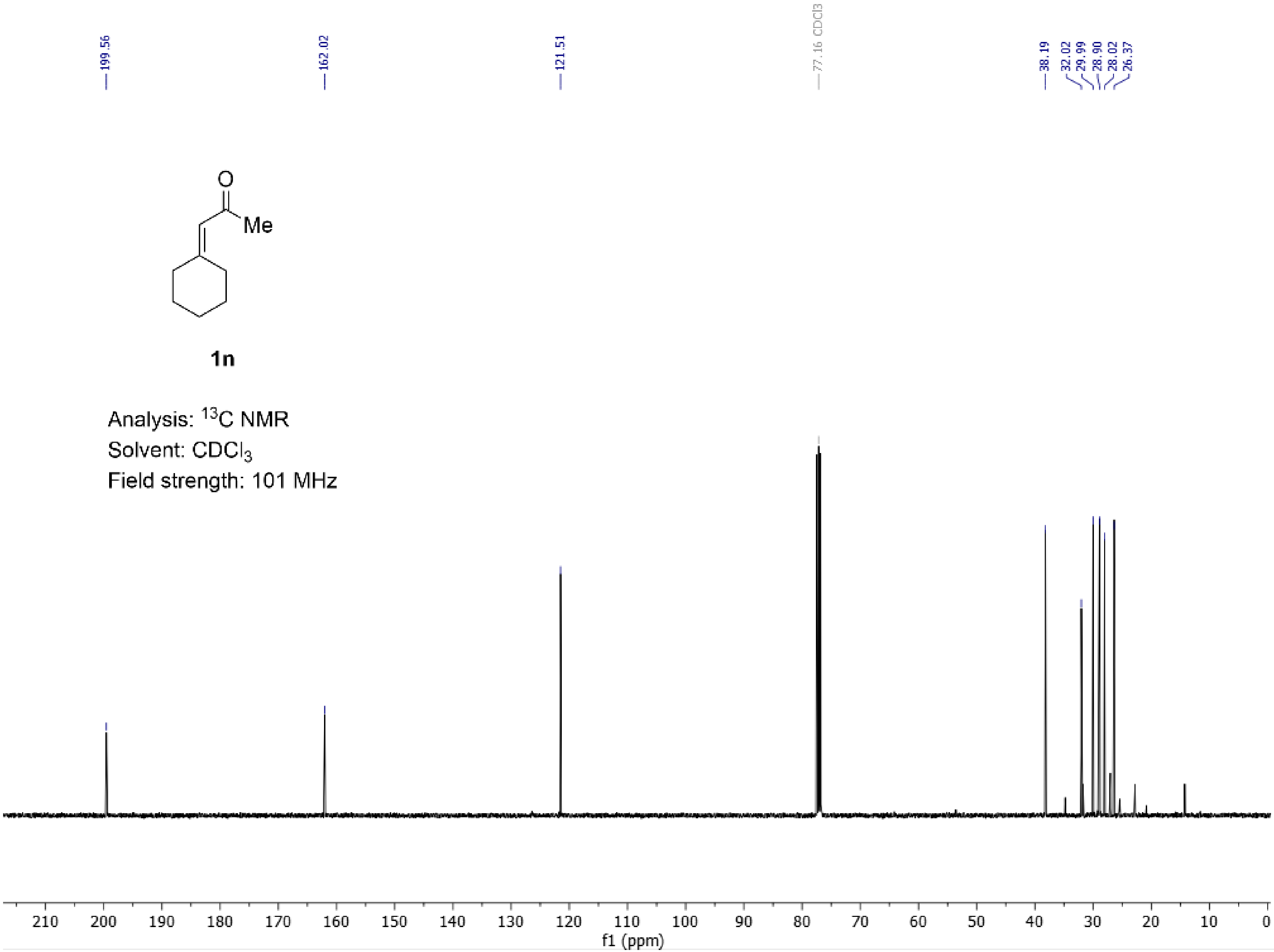
199.56
162.02
121.51
77.16 CDCl3
38.19
32.02
29.99
28.90
28.02
26.37
O
Me
1n
Analysis: ^{13}C NMR
Solvent: $CDCl_3$
Field strength: 101 MHz
210 200 190 180 170 160 150 140 130 120 110 100 90 80 70 60 50 40 30 20 10 0
f1 (ppm)

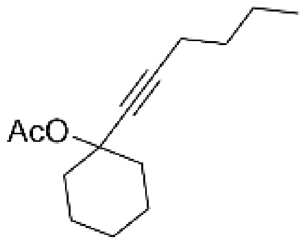

S1

Analysis: ^{1}H NMR
Solvent: $CDCl_3$
Field strength: 400 MHz

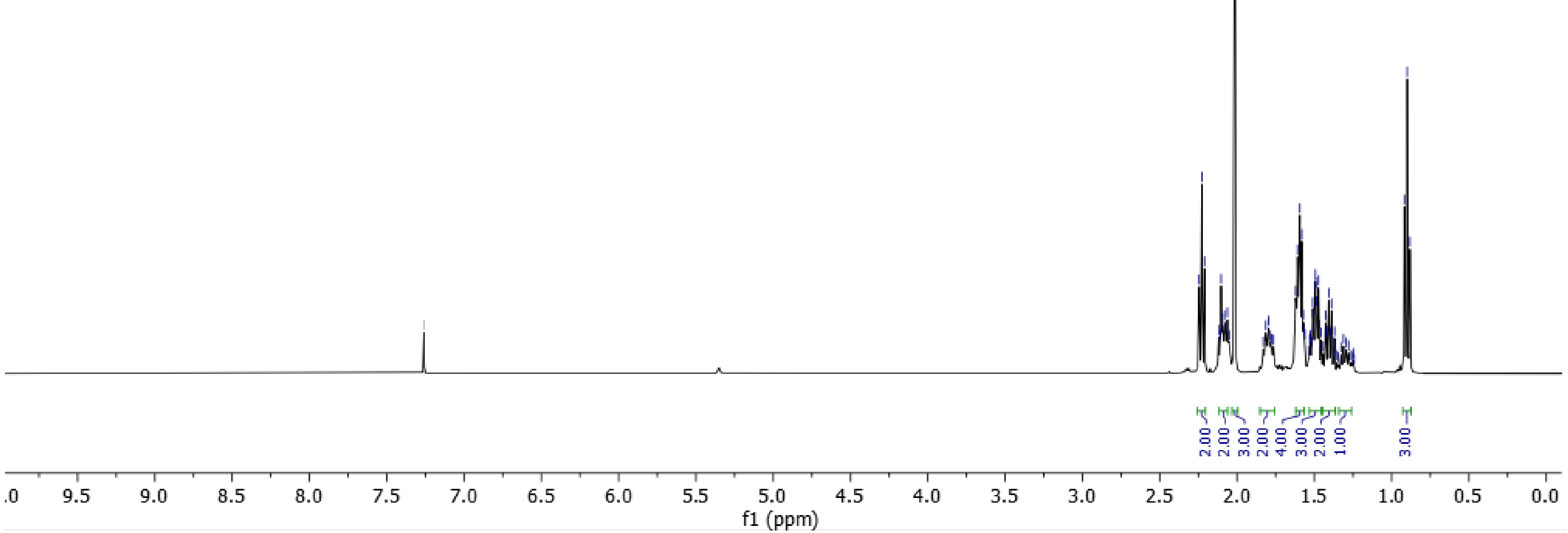

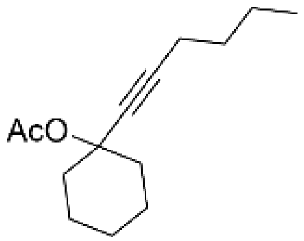

S1

Analysis: ^{13}C NMR
Solvent: $CDCl_3$
Field strength: 101 MHz

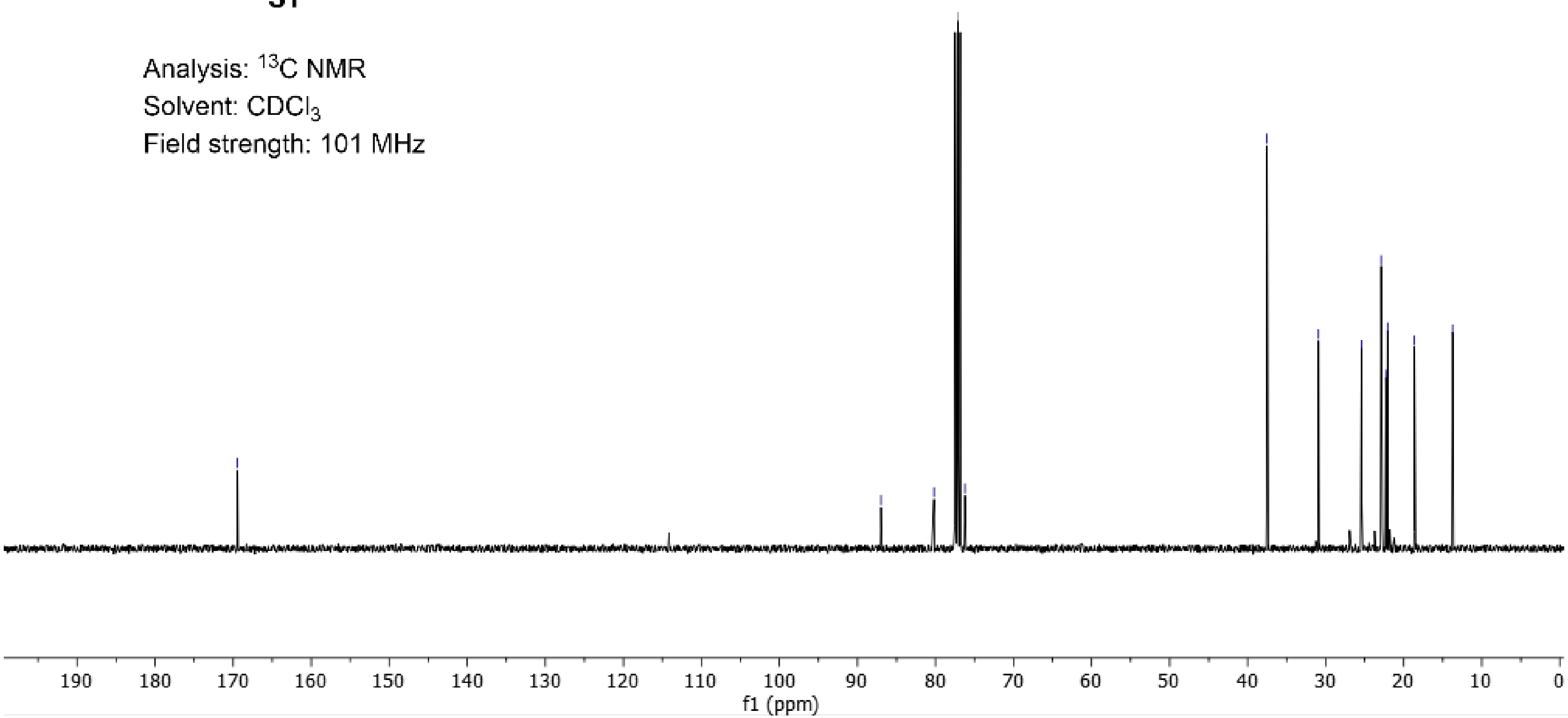

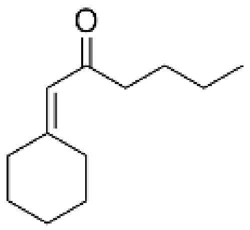

1o

Analysis: ^{1}H NMR
Solvent: $CDCl_3$
Field strength: 400 MHz

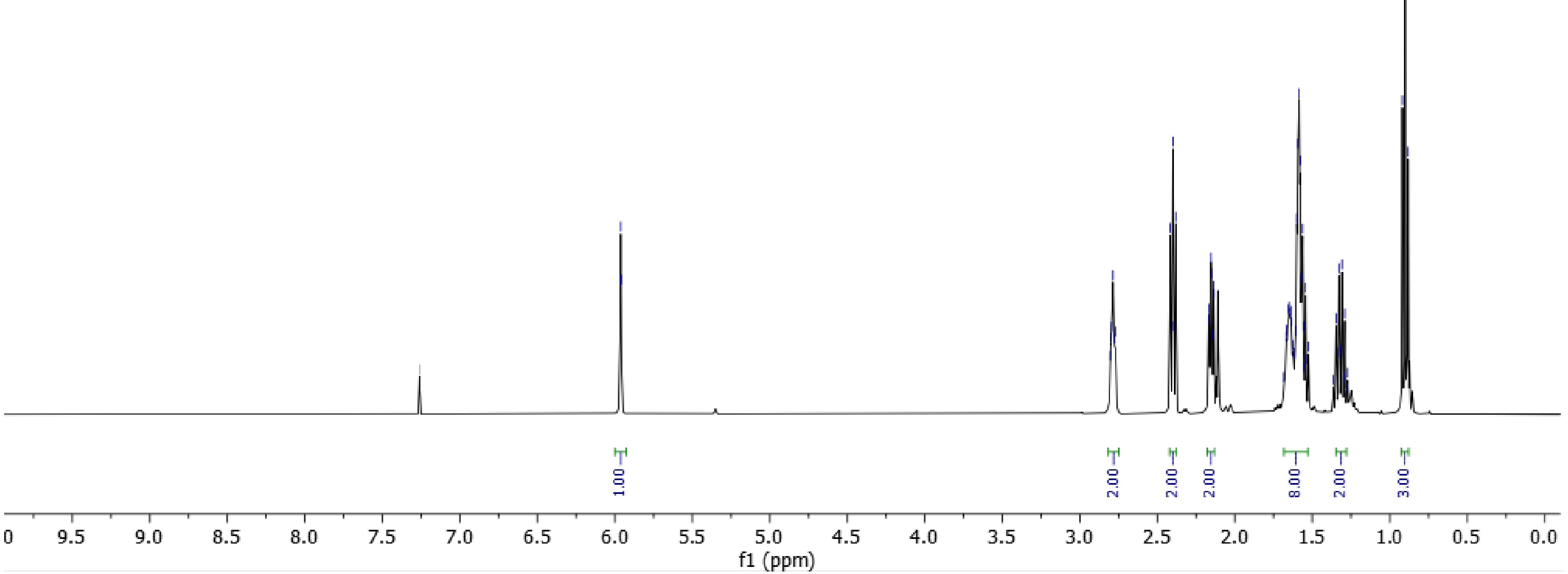

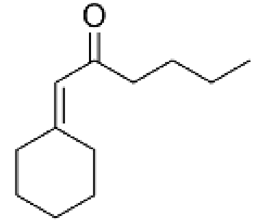

1o

Analysis: ^{13}C NMR
Solvent: $CDCl_3$
Field strength: 101 MHz

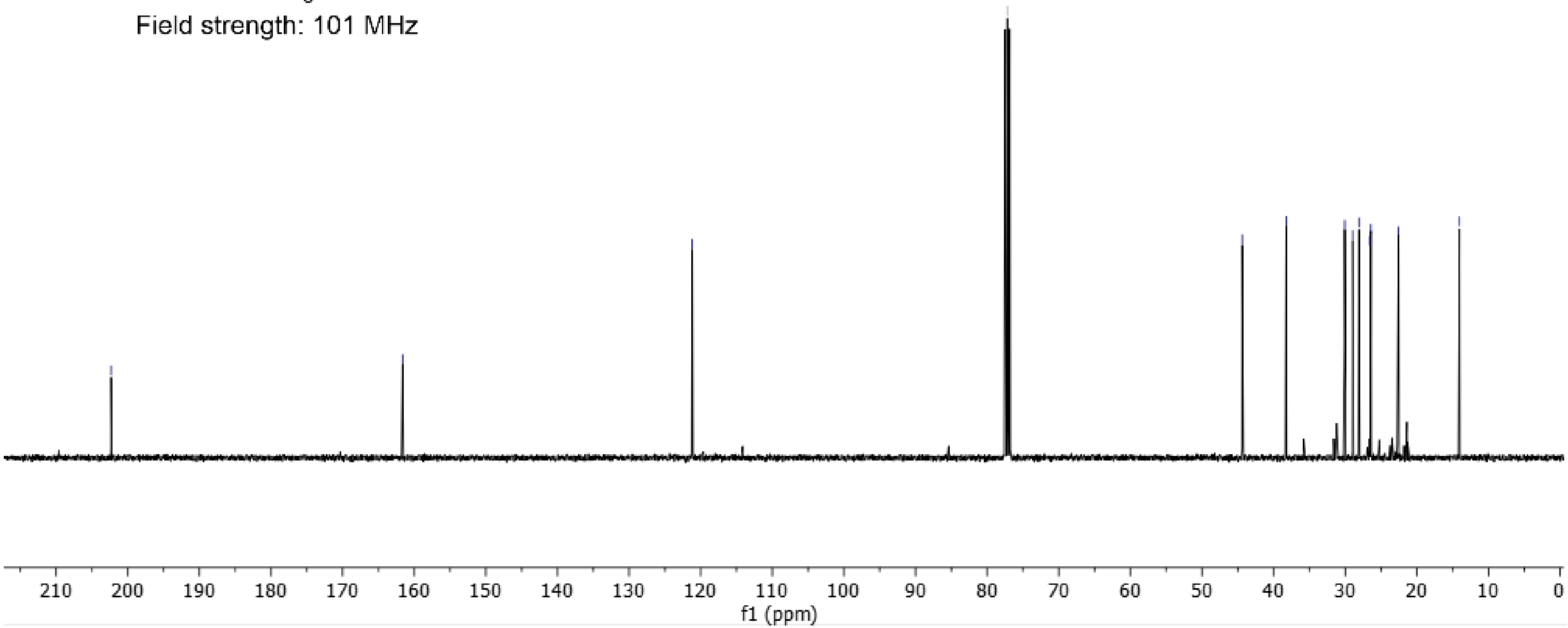

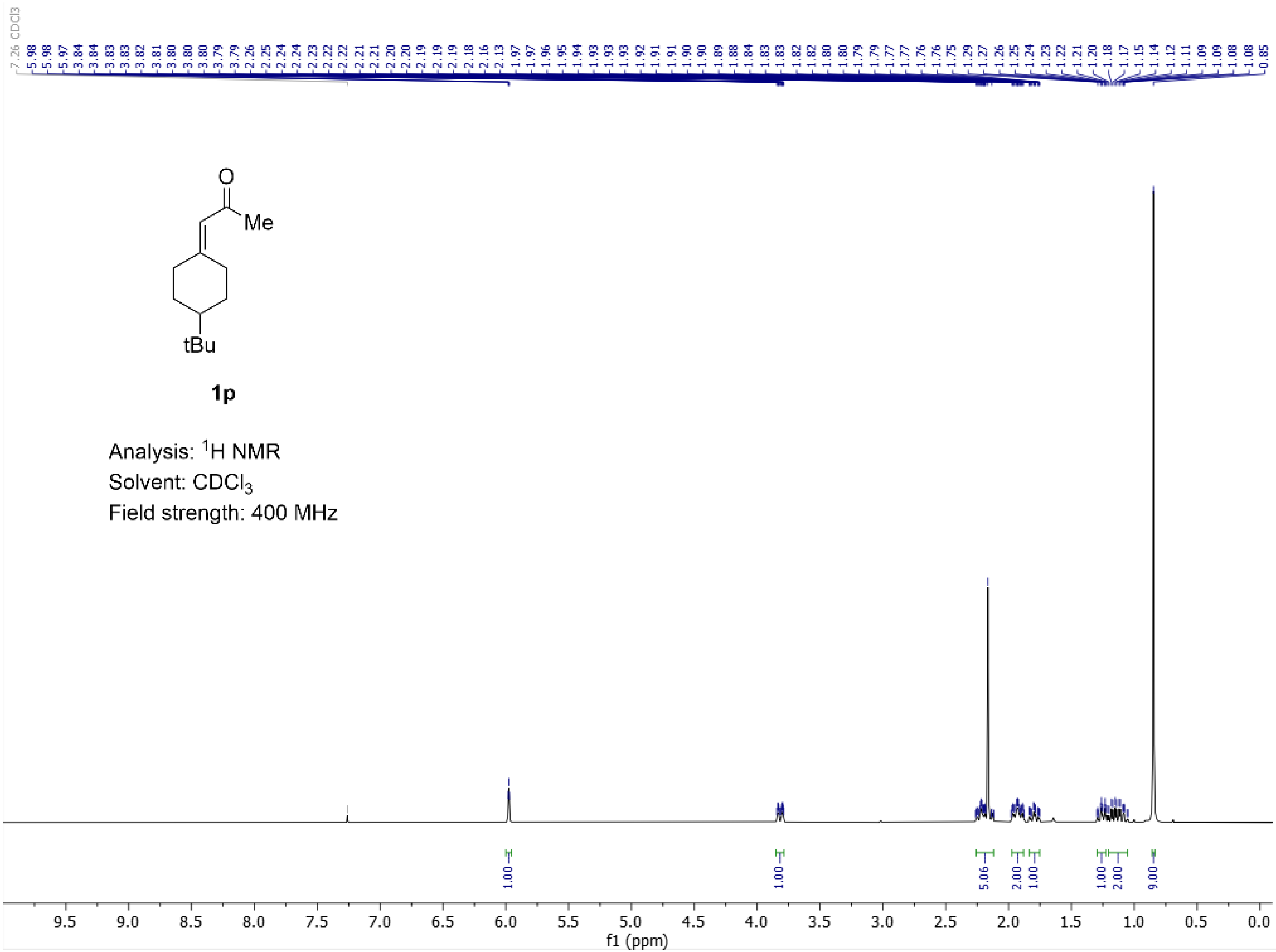
O
Me
tBu
1p
Analysis: ^{1}H NMR
Solvent: $CDCl_3$
Field strength: 400 MHz
f1 (ppm)

1p

Analysis: ^{13}C NMR
Solvent: $CDCl_3$
Field strength: 101 MHz

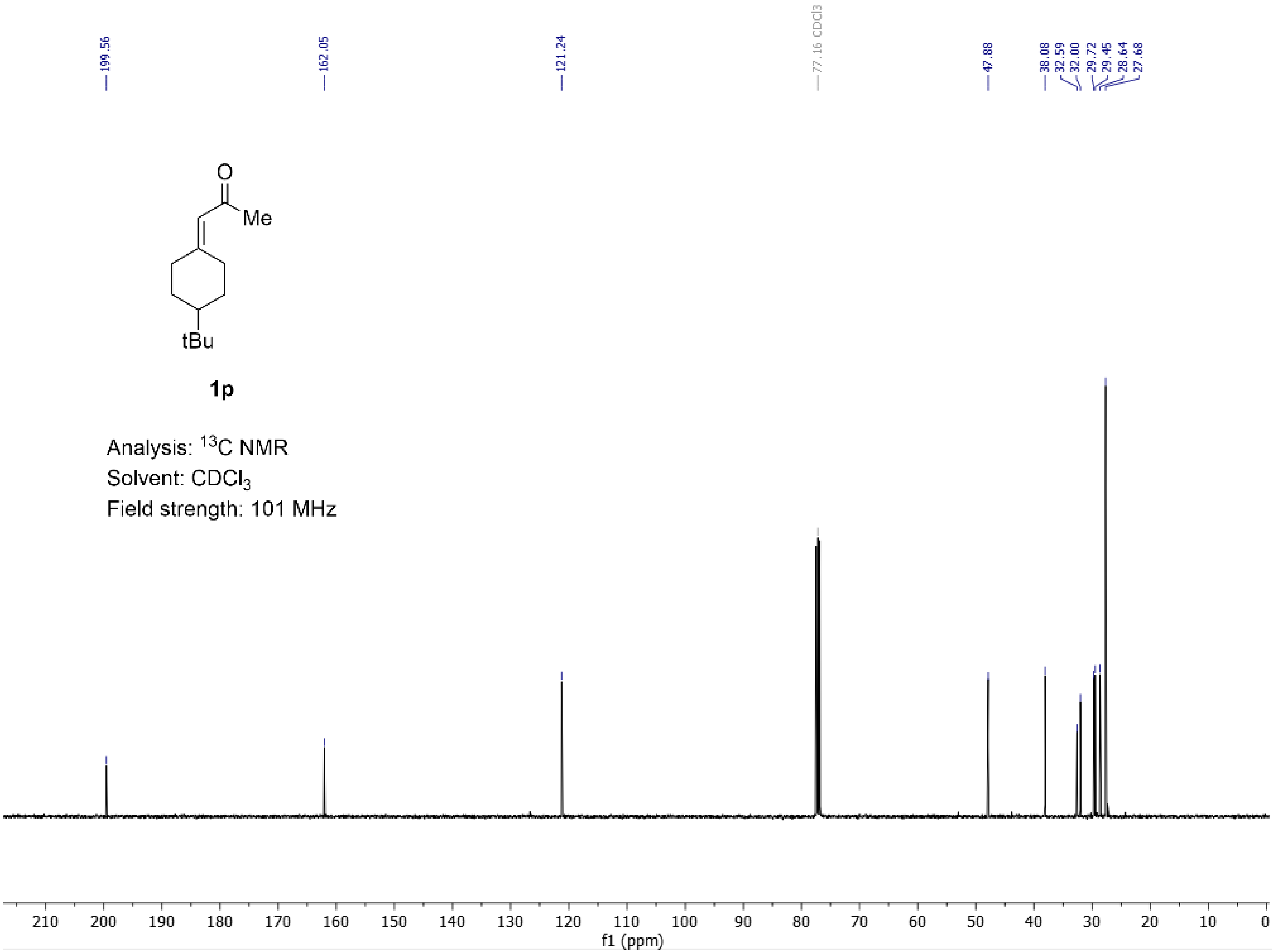

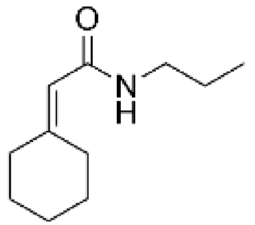

1q

Analysis: ^{1}H NMR
Solvent: $CDCl_3$
Field strength: 400 MHz

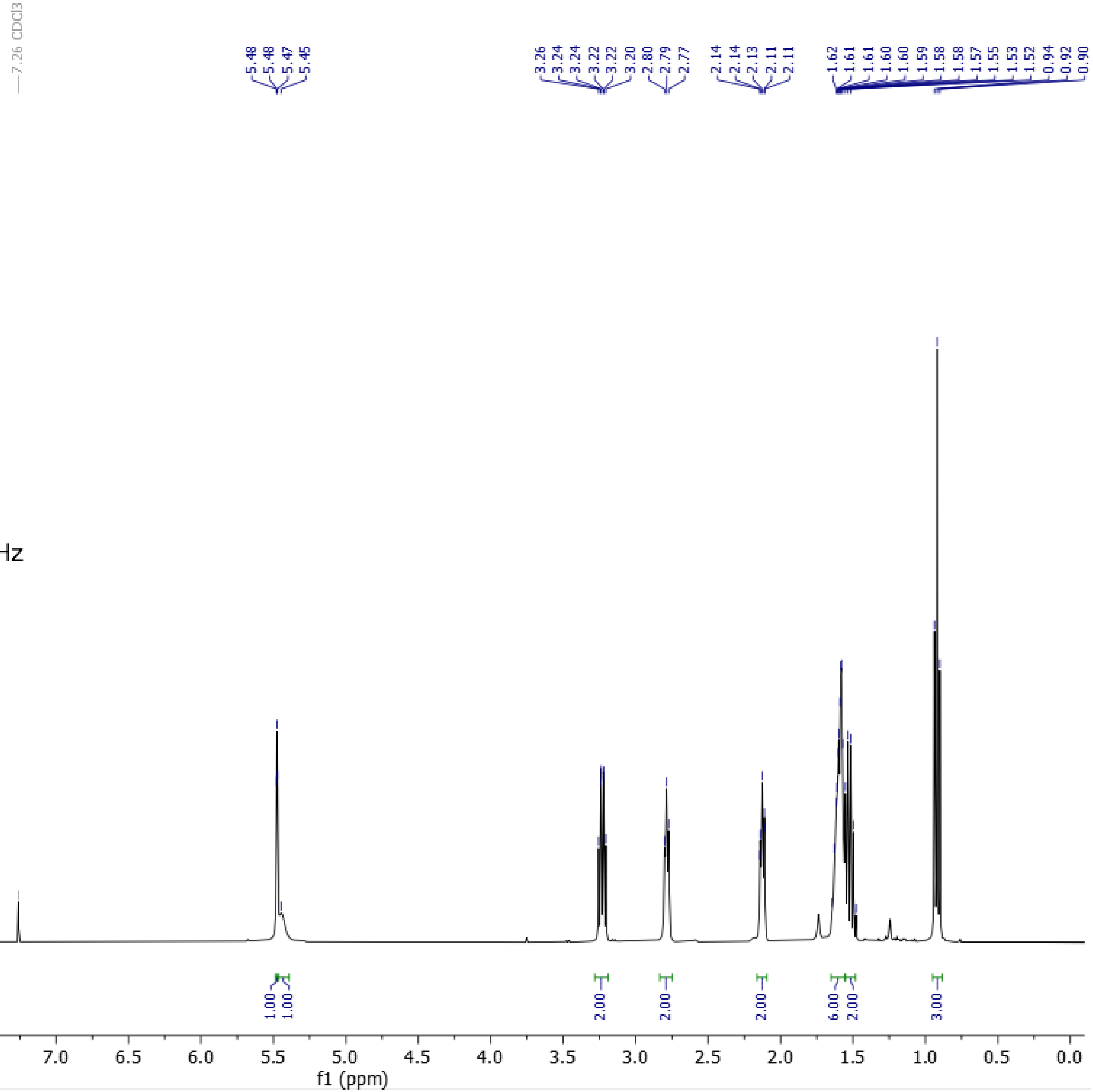

1q

Analysis: ^{13}C NMR
Solvent: $CDCl_3$
Field strength: 101 MHz

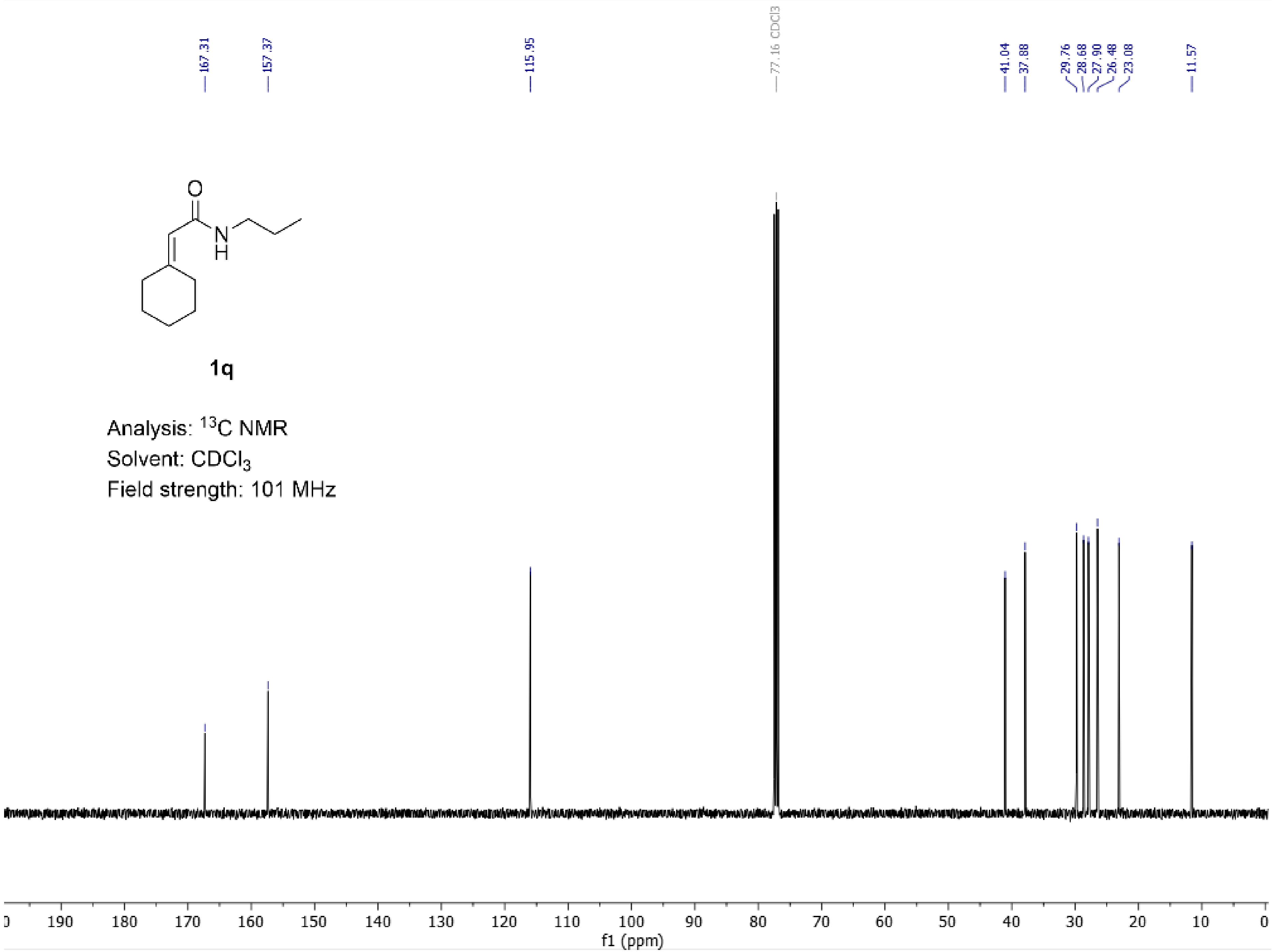

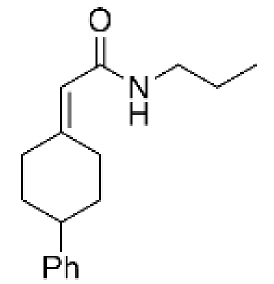

1r

Analysis: ^{1}H NMR
Solvent: $CDCl_3$
Field strength: 400 MHz

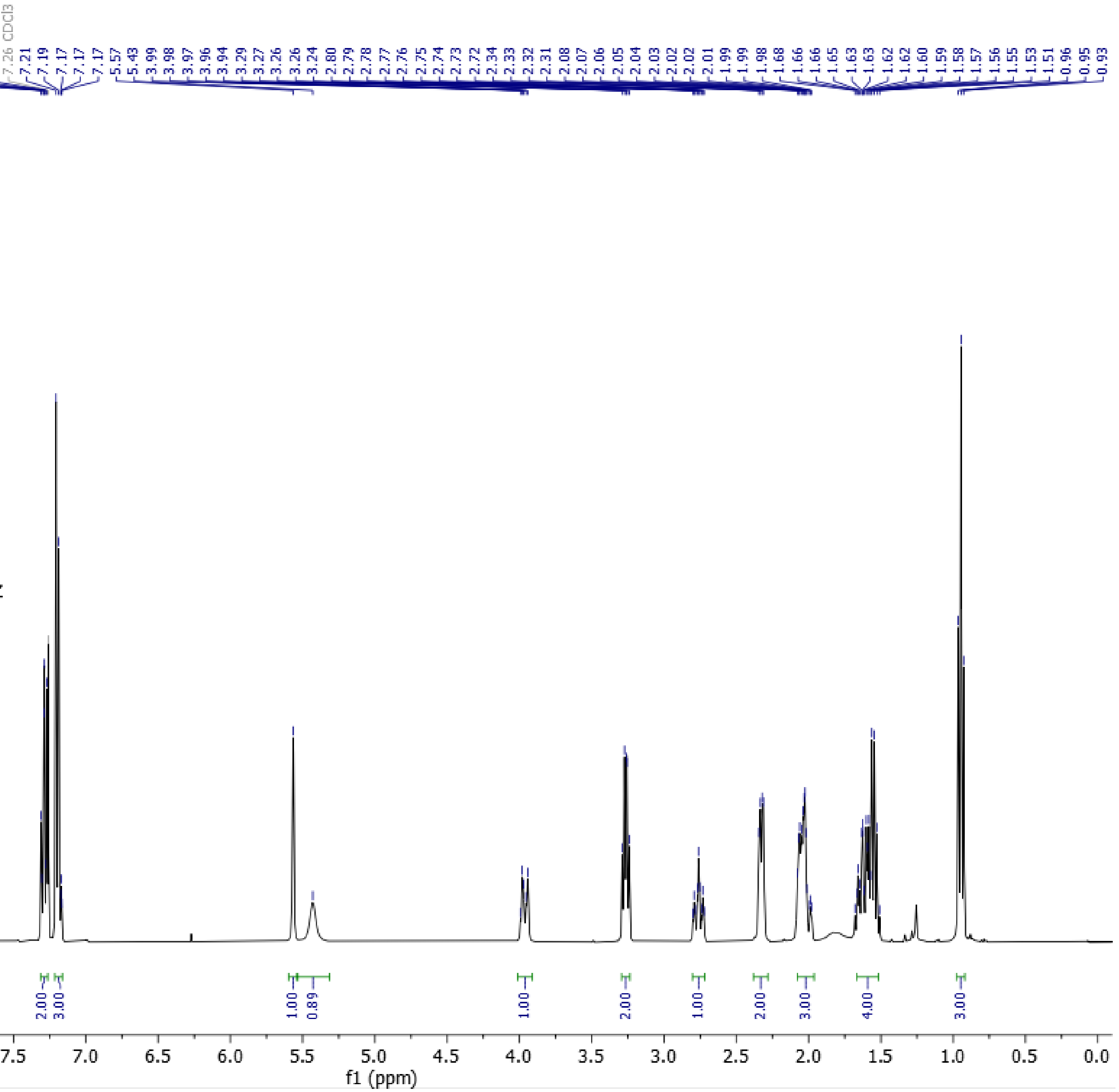

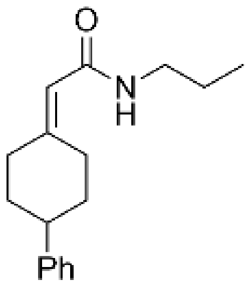

1r

Analysis: ^{13}C NMR
Solvent: $CDCl_3$
Field strength: 101 MHz

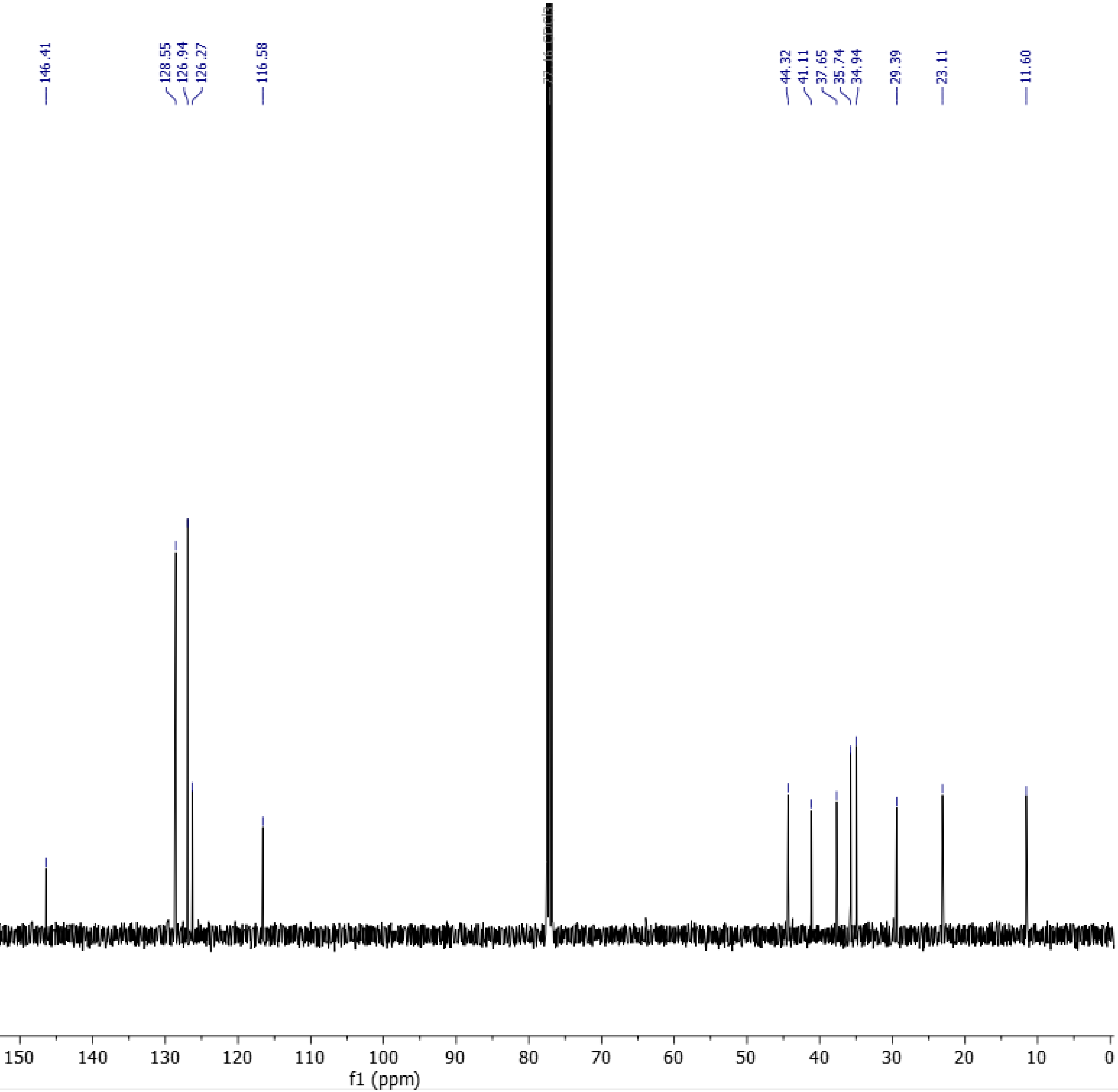

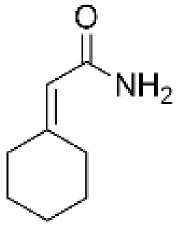

1s

Analysis: ^{1}H NMR
Solvent: $CDCl_3$
Field strength: 400 MHz

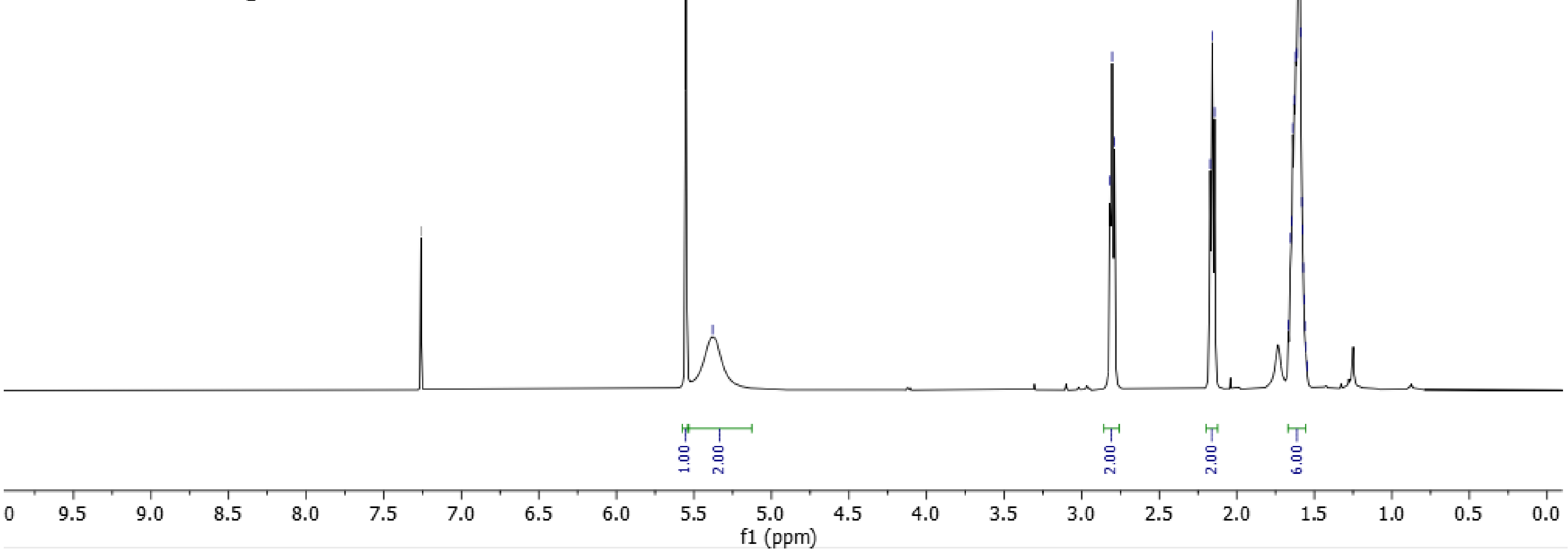

1s

Analysis: ^{13}C NMR
Solvent: $CDCl_3$
Field strength: 101 MHz

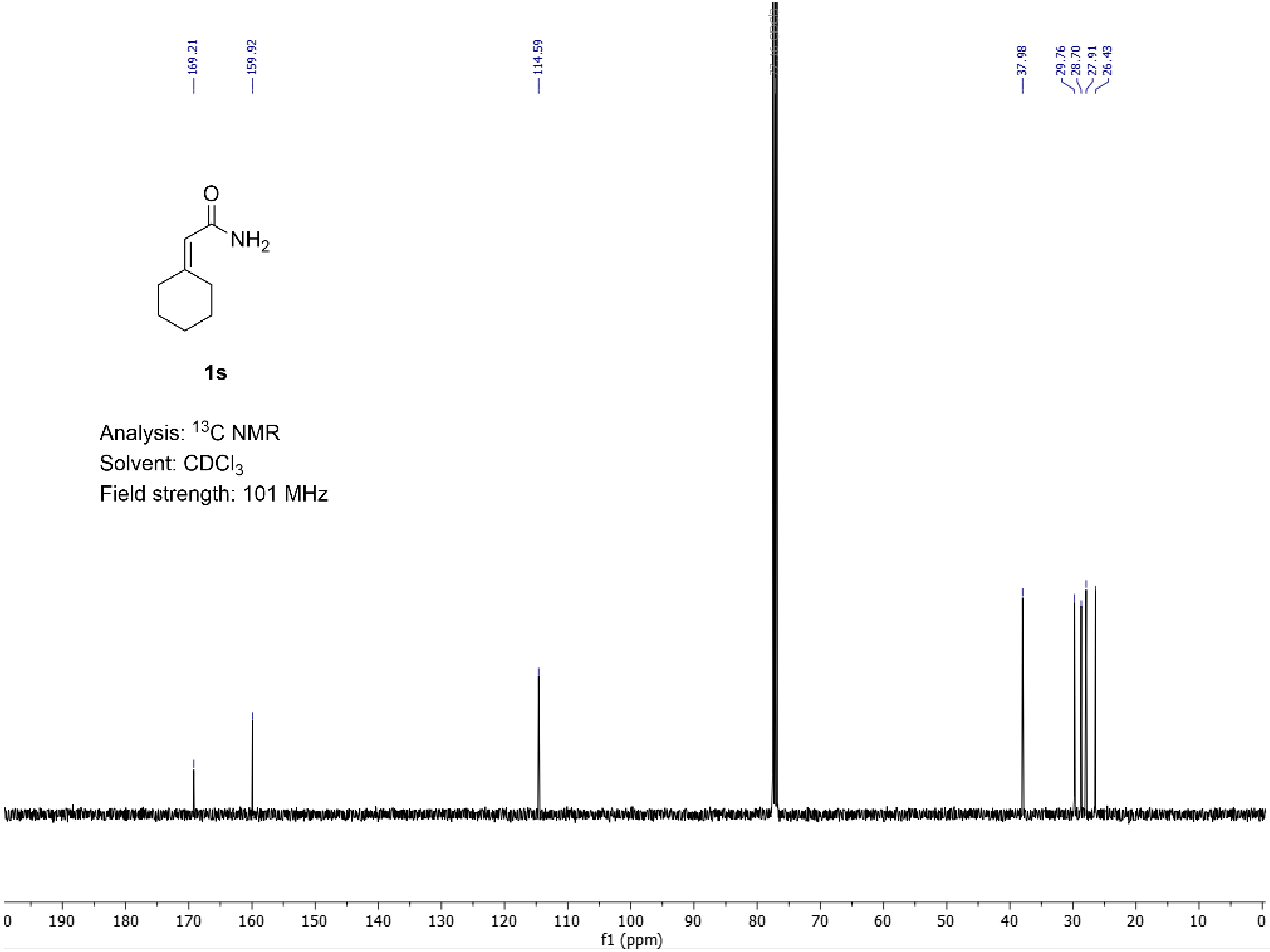

1t

Analysis: ^{1}H NMR
Solvent: $CDCl_3$
Field strength: 400 MHz

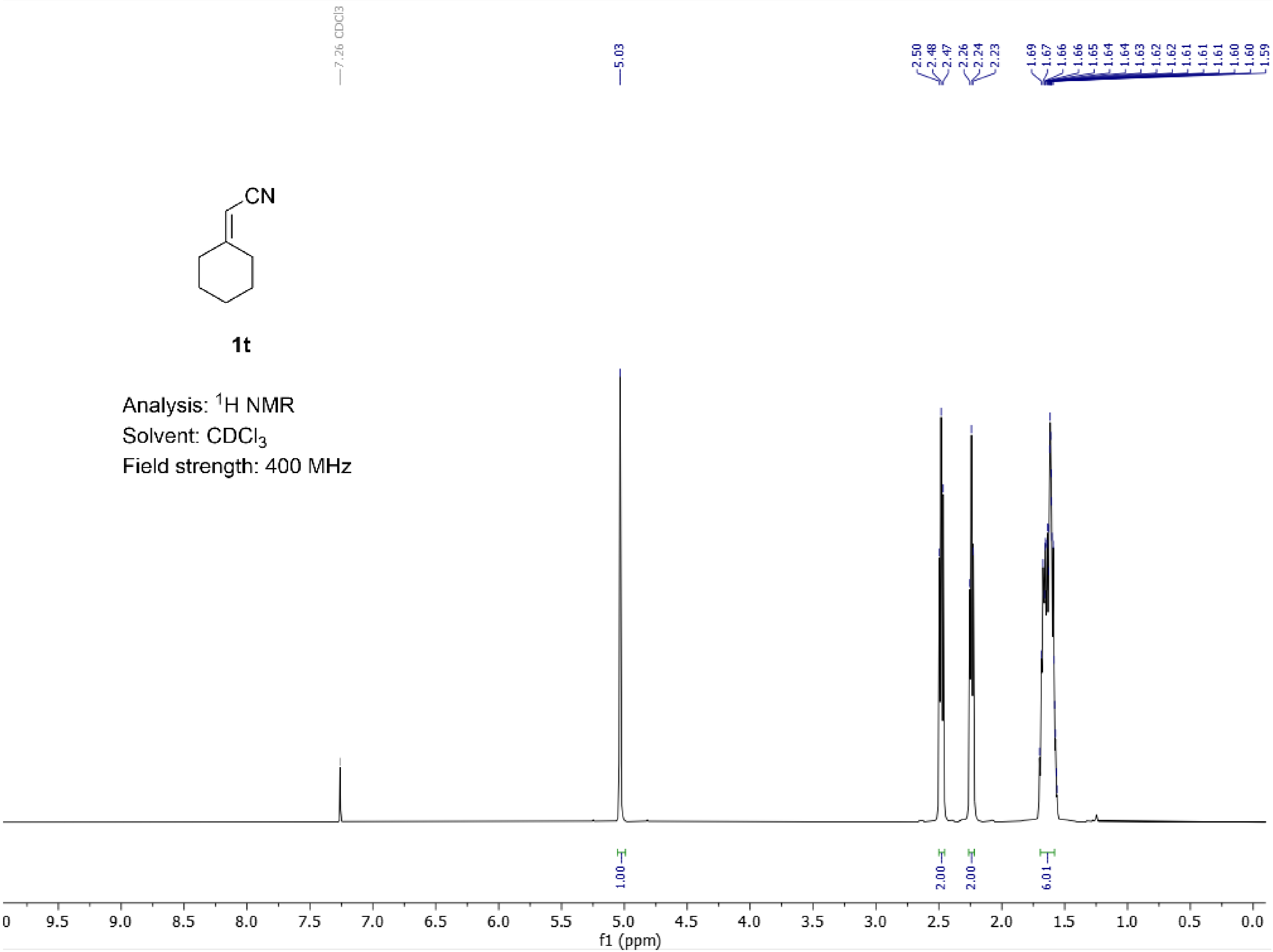

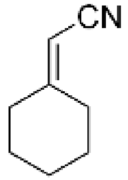

1t

Analysis: ^{13}C NMR
Solvent: $CDCl_3$
Field strength: 101 MHz

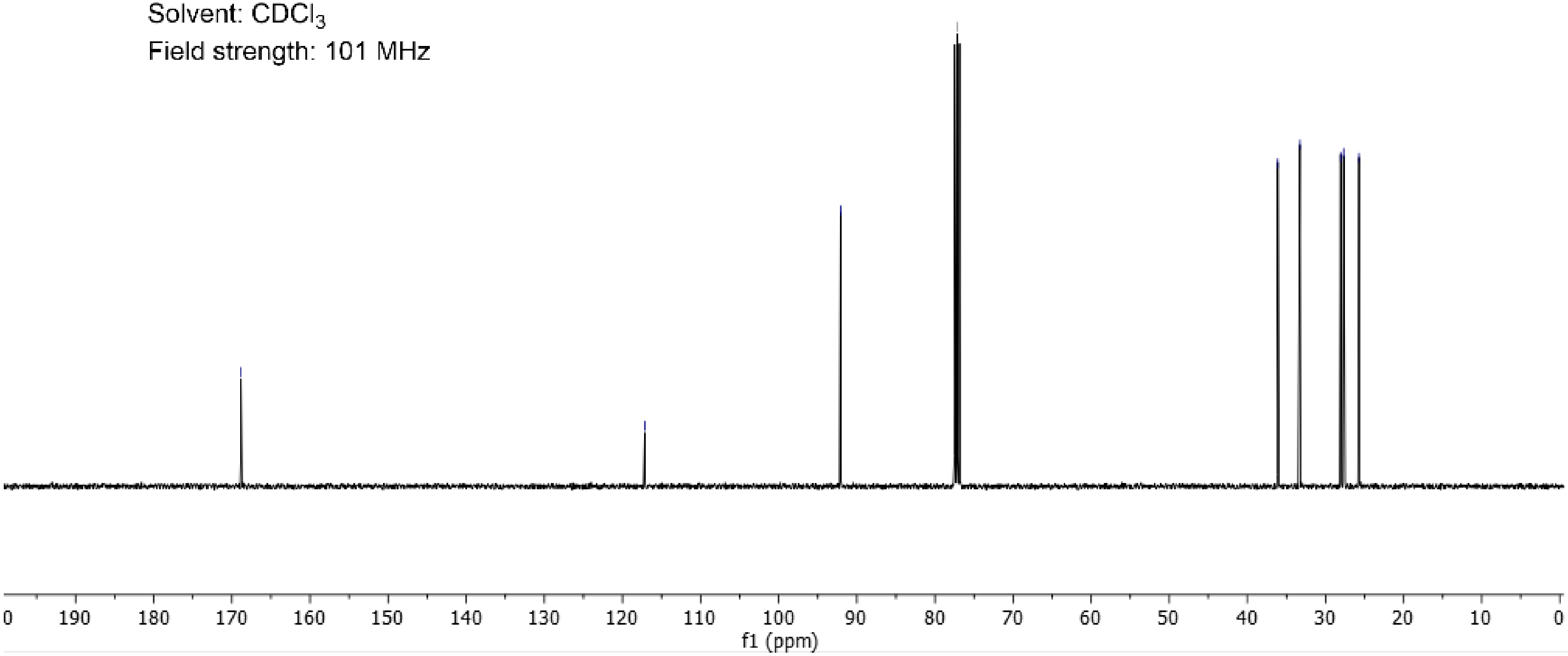

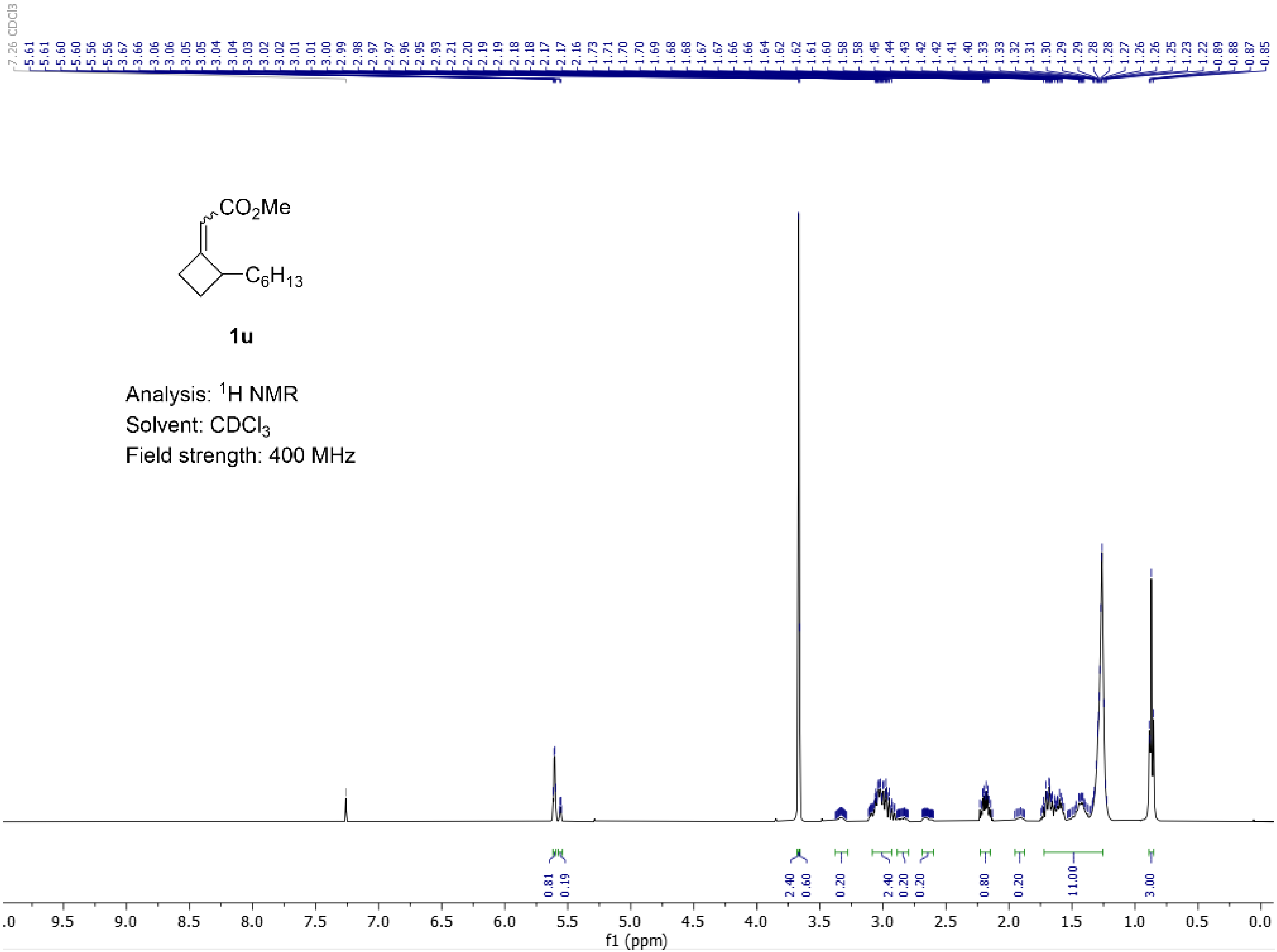
7.26 CDCl3
5.61 5.61 5.60 5.60 5.56 5.56 3.67 3.66 3.06 3.06 3.05 3.05 3.04 3.04 3.03 3.02 3.02 3.01 3.01 3.00 2.99 2.98 2.97 2.97 2.96 2.95 2.93 2.21 2.20 2.19 2.19 2.18 2.18 2.17 2.17 2.16 1.73 1.71 1.70 1.70 1.69 1.68 1.68 1.67 1.67 1.66 1.66 1.64 1.62 1.62 1.61 1.60 1.58 1.58 1.45 1.44 1.43 1.42 1.42 1.41 1.40 1.33 1.33 1.32 1.31 1.30 1.29 1.29 1.28 1.28 1.27 1.26 1.26 1.25 1.23 1.22 0.89 0.88 0.87 0.85
CO2Me
C6H13
1u
Analysis: 1H NMR
Solvent: CDCl3
Field strength: 400 MHz
0.81 0.19 2.40 0.60 0.20 2.40 0.20 0.20 0.80 0.20 11.00 3.00
f1 (ppm)

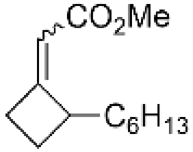

1u

Analysis: ^{13}C NMR
Solvent: $CDCl_3$
Field strength: 101 MHz

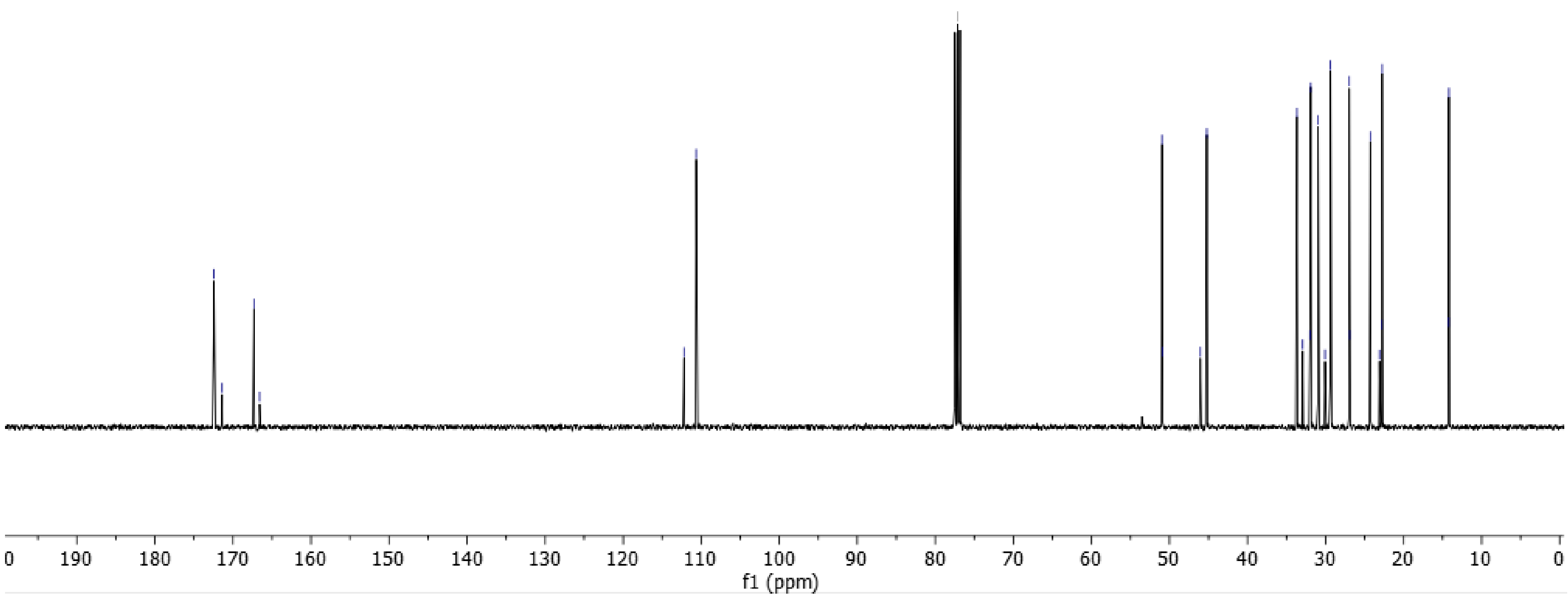

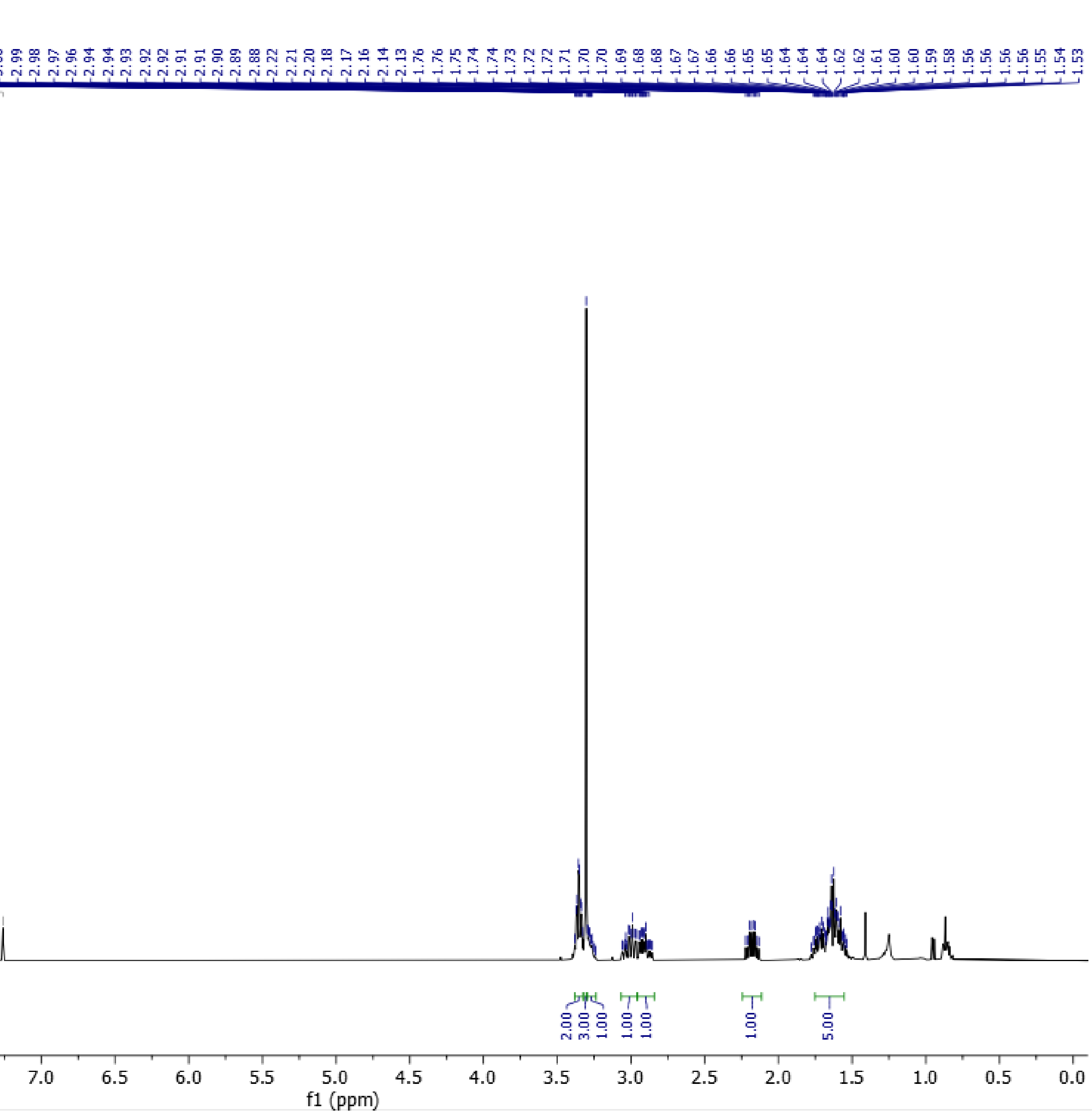

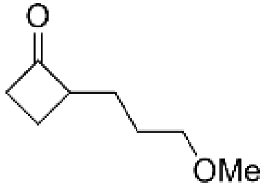

S3

Analysis: ^{1}H NMR
Solvent: $CDCl_3$
Field strength: 400 MHz

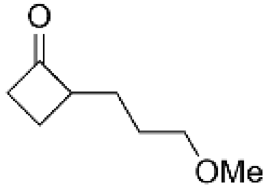

S3

Analysis: ^{13}C NMR
Solvent: $CDCl_3$
Field strength: 101 MHz

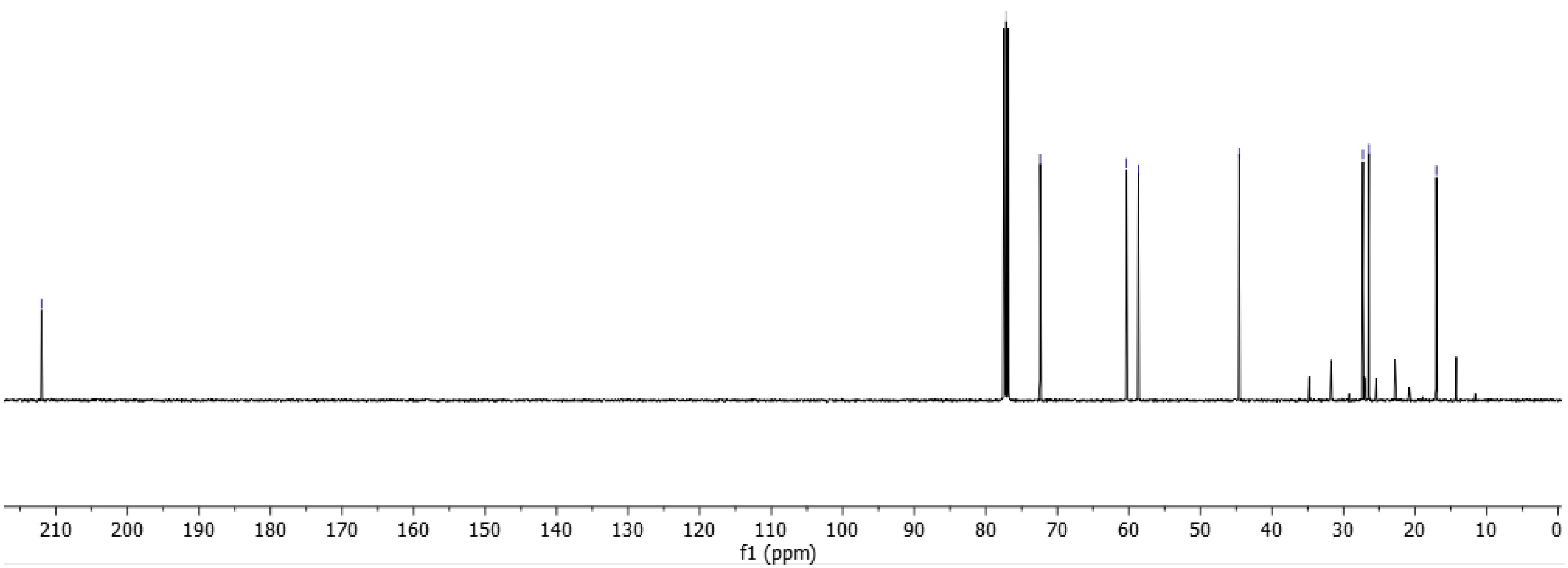

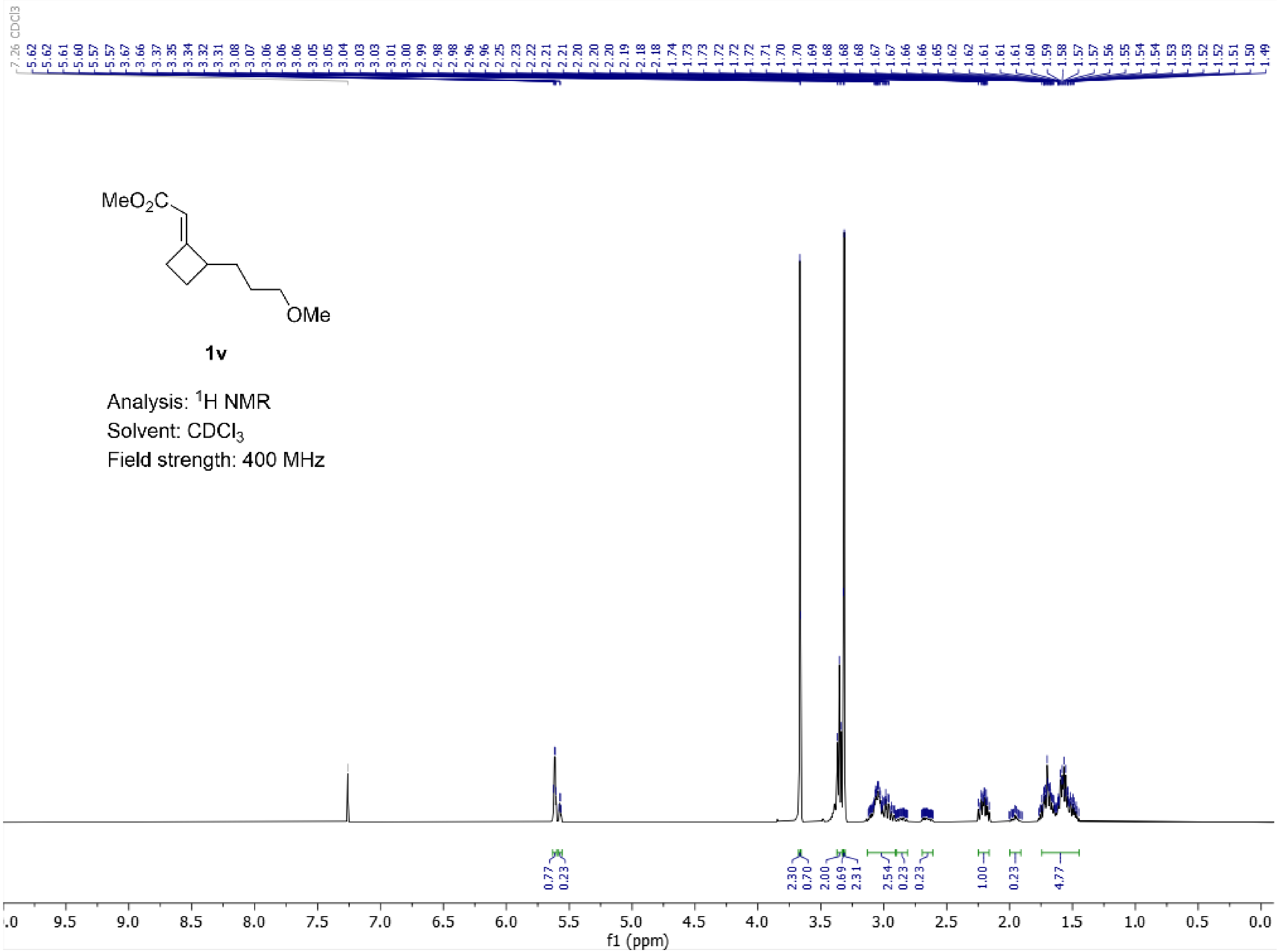

MeO2C
OMe
1v
Analysis: 1H NMR
Solvent: CDCl3
Field strength: 400 MHz
f1 (ppm)

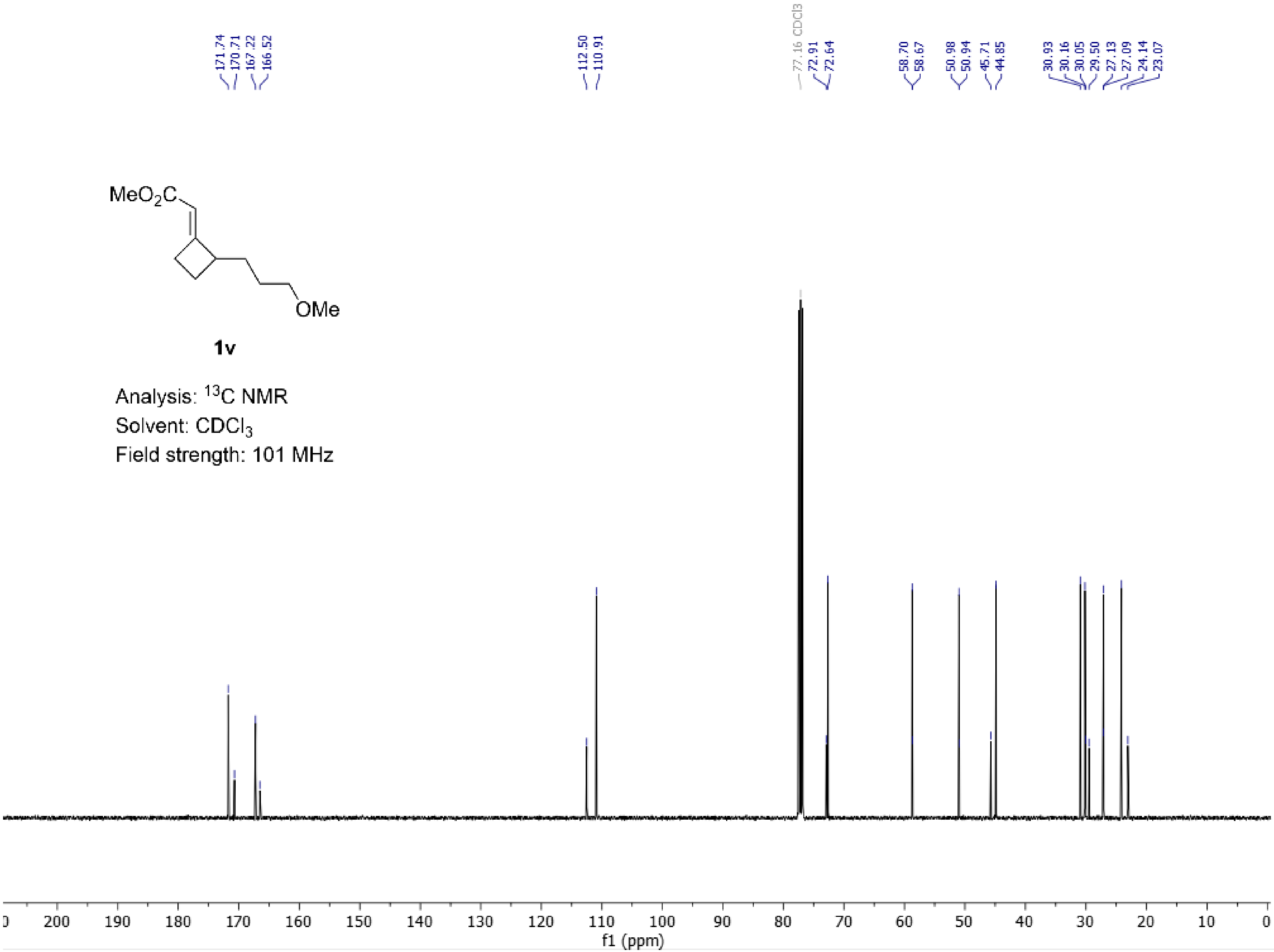

1v

Analysis: ^{13}C NMR
Solvent: $CDCl_3$
Field strength: 101 MHz

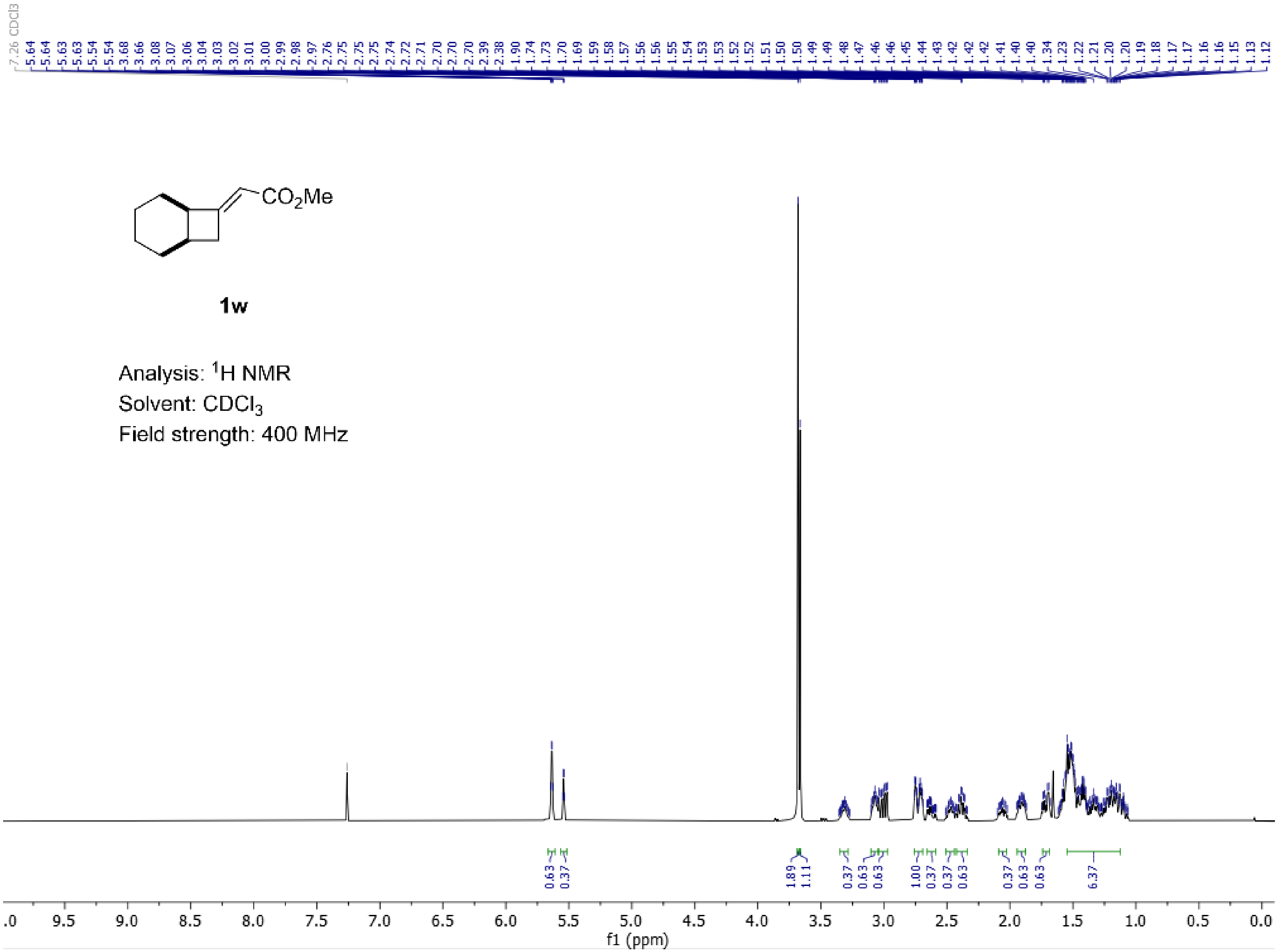

1w

Analysis: ^{1}H NMR
Solvent: $CDCl_3$
Field strength: 400 MHz

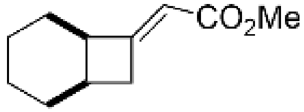

1w

Analysis: ^{13}C NMR

Solvent: $CDCl_3$

Field strength: 101 MHz

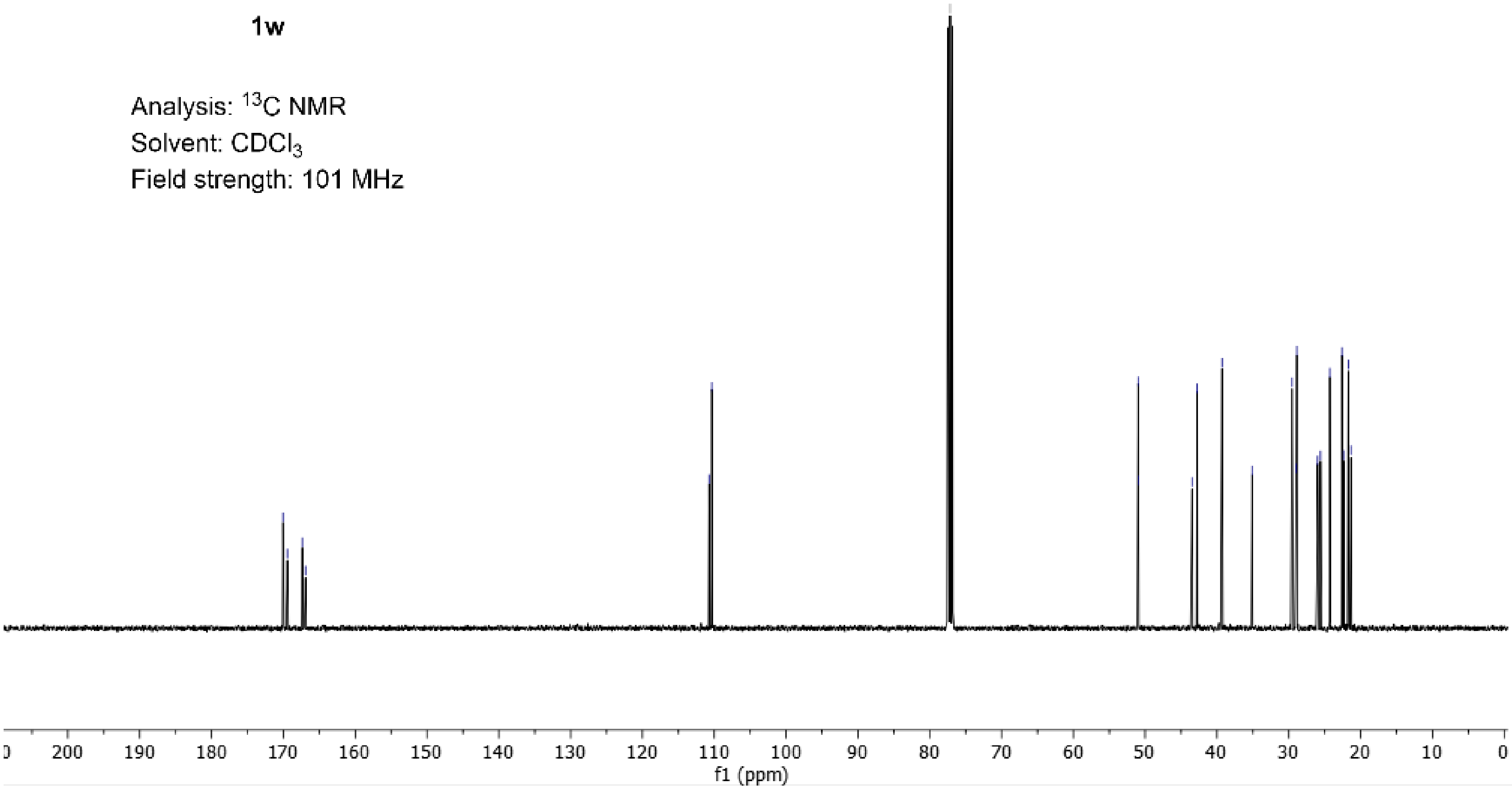

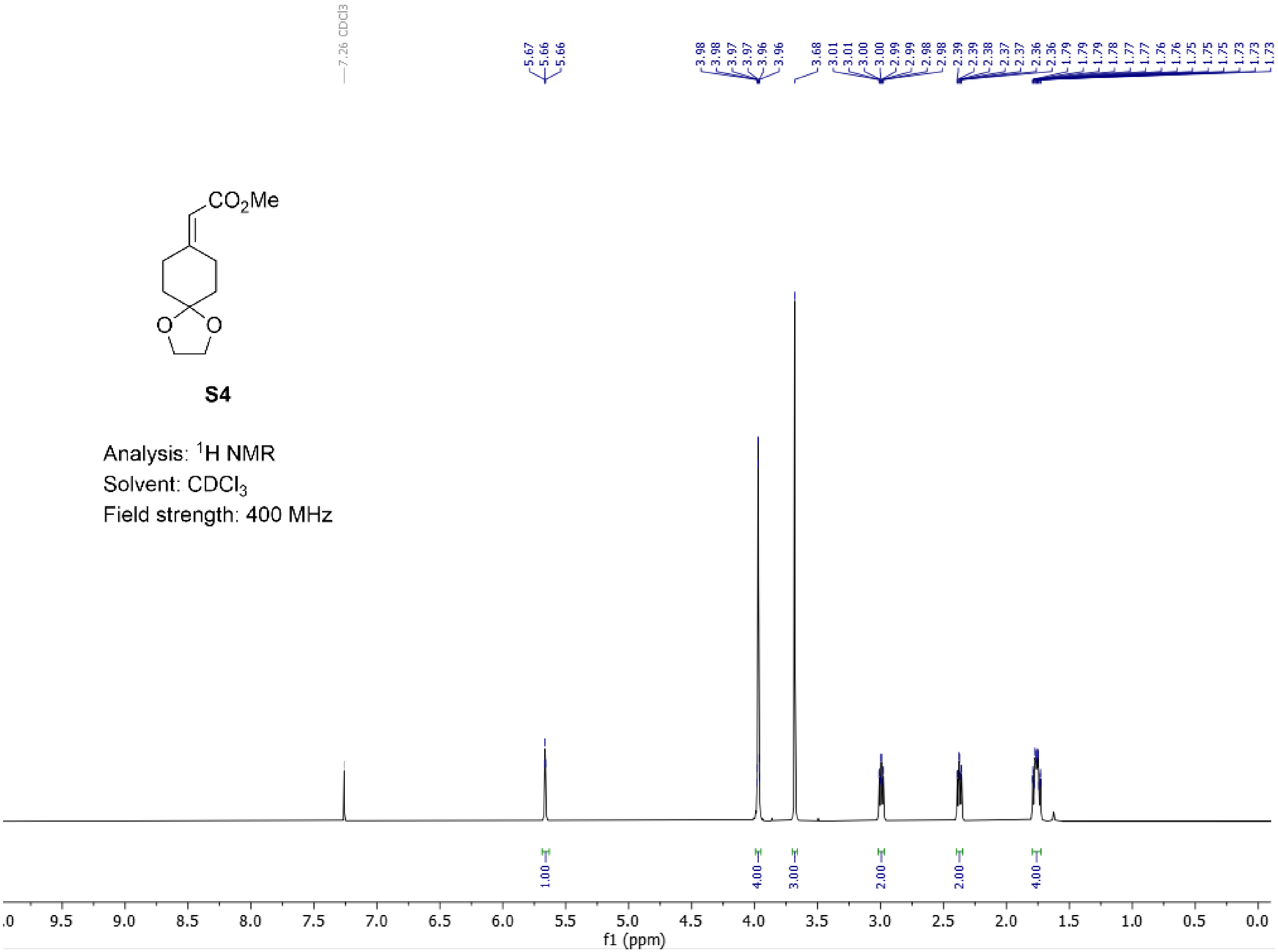

CO2Me
O
O
S4
Analysis: 1H NMR
Solvent: CDCl3
Field strength: 400 MHz
7.26 CDCl3
5.67 5.66 5.66
3.98 3.98 3.97 3.97 3.96 3.96
3.68
3.01 3.01 3.00 3.00 2.99 2.99 2.98 2.98
2.39 2.39 2.38 2.37 2.37 2.36 2.36
1.79 1.79 1.79 1.78 1.77 1.77 1.76 1.76 1.75 1.75 1.75 1.73 1.73 1.73
1.00
4.00
3.00
2.00
2.00
4.00
f1 (ppm)

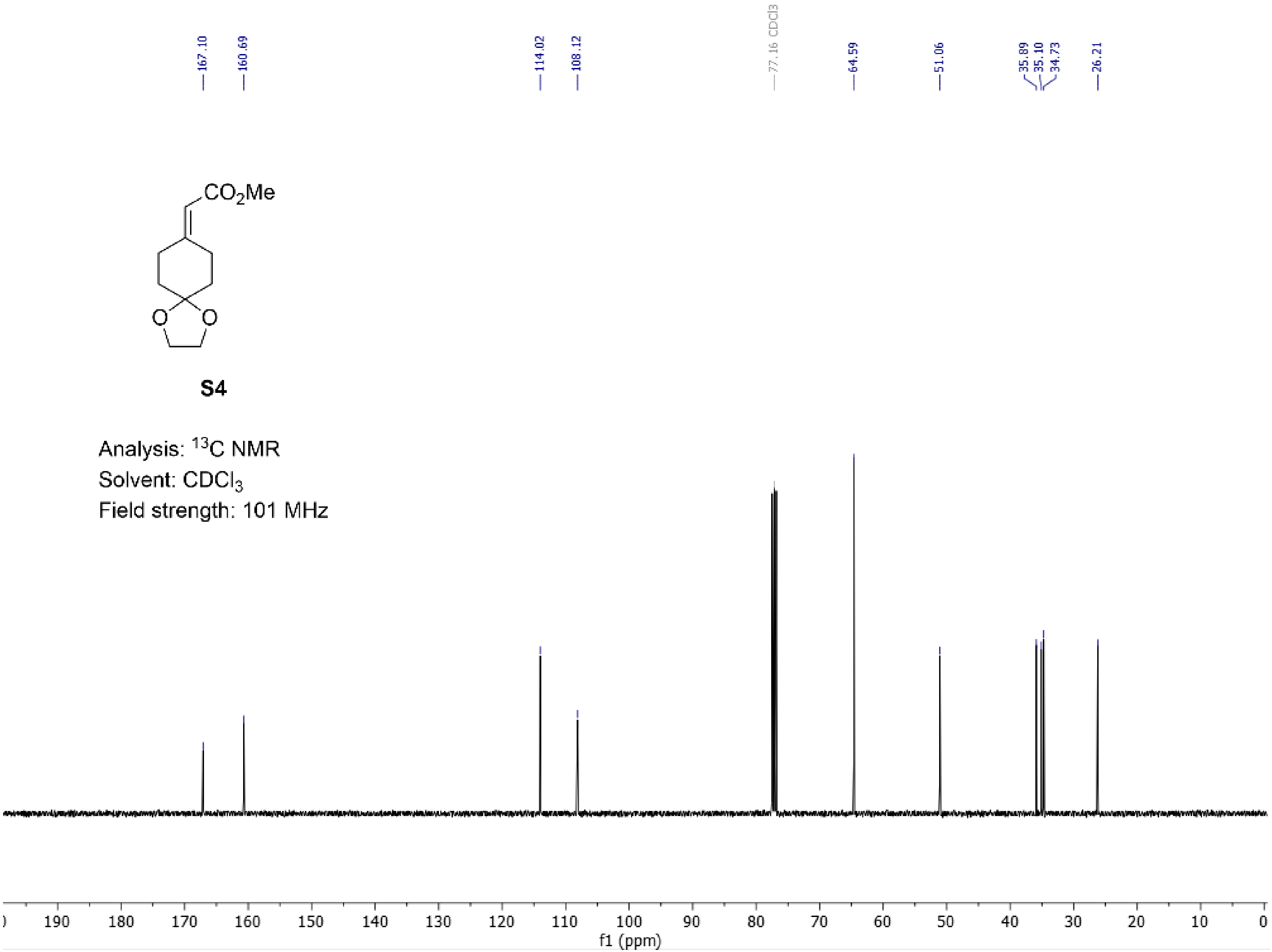
167.10
160.69
114.02
108.12
77.16 CDCl3
64.59
51.06
35.89
35.10
34.73
26.21
CO2Me
O
O
S4
Analysis: 13C NMR
Solvent: CDCl3
Field strength: 101 MHz
190
180
170
160
150
140
130
120
110
100
90
80
70
60
50
40
30
20
10
0
f1 (ppm)

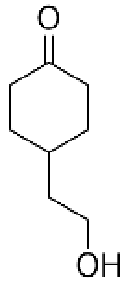

S5

Analysis: ^{1}H NMR
Solvent: $CDCl_3$
Field strength: 400 MHz

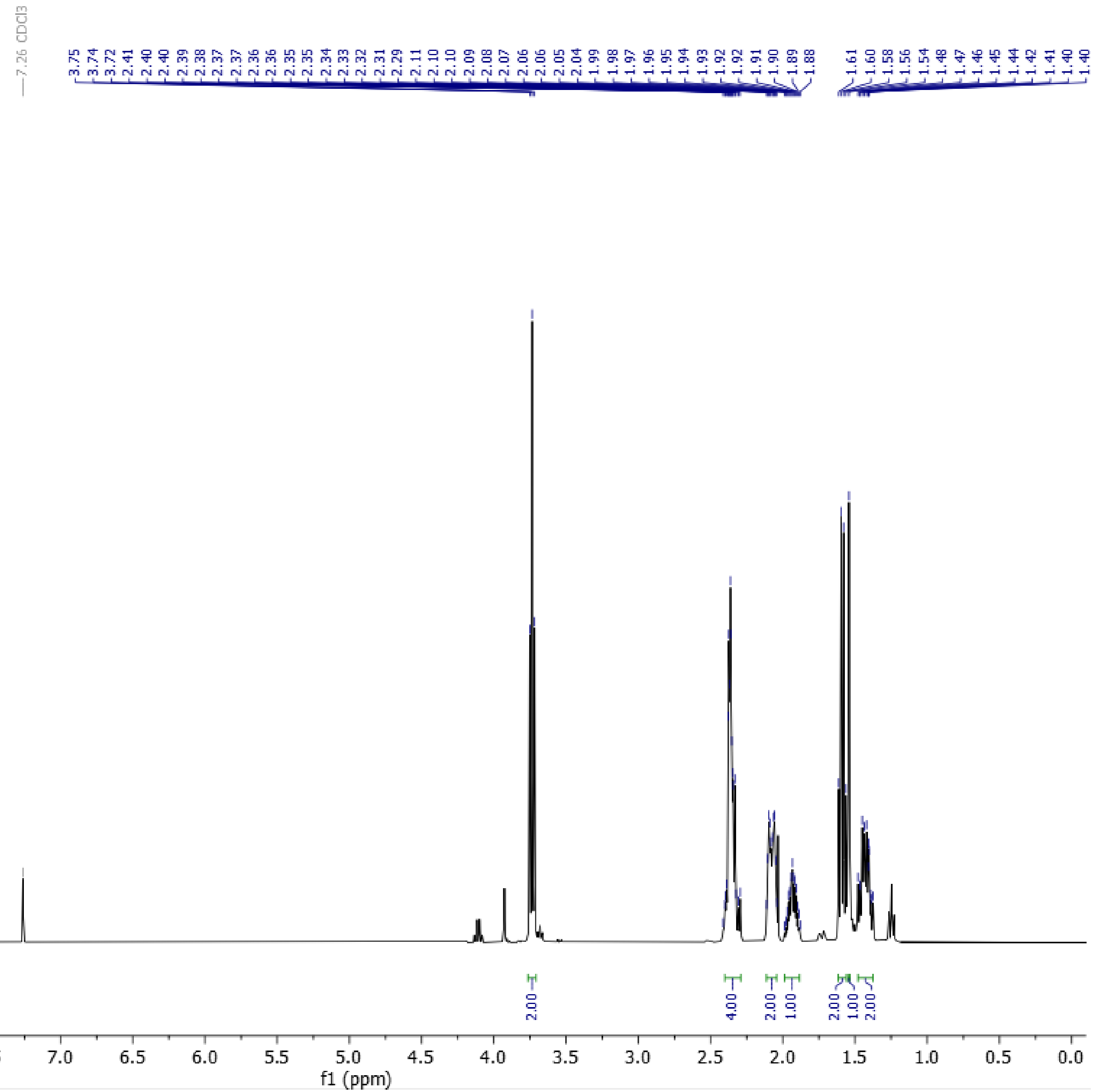

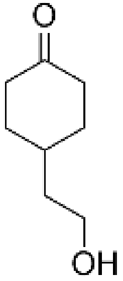

S5

Analysis: ^{13}C NMR
Solvent: $CDCl_3$
Field strength: 101 MHz

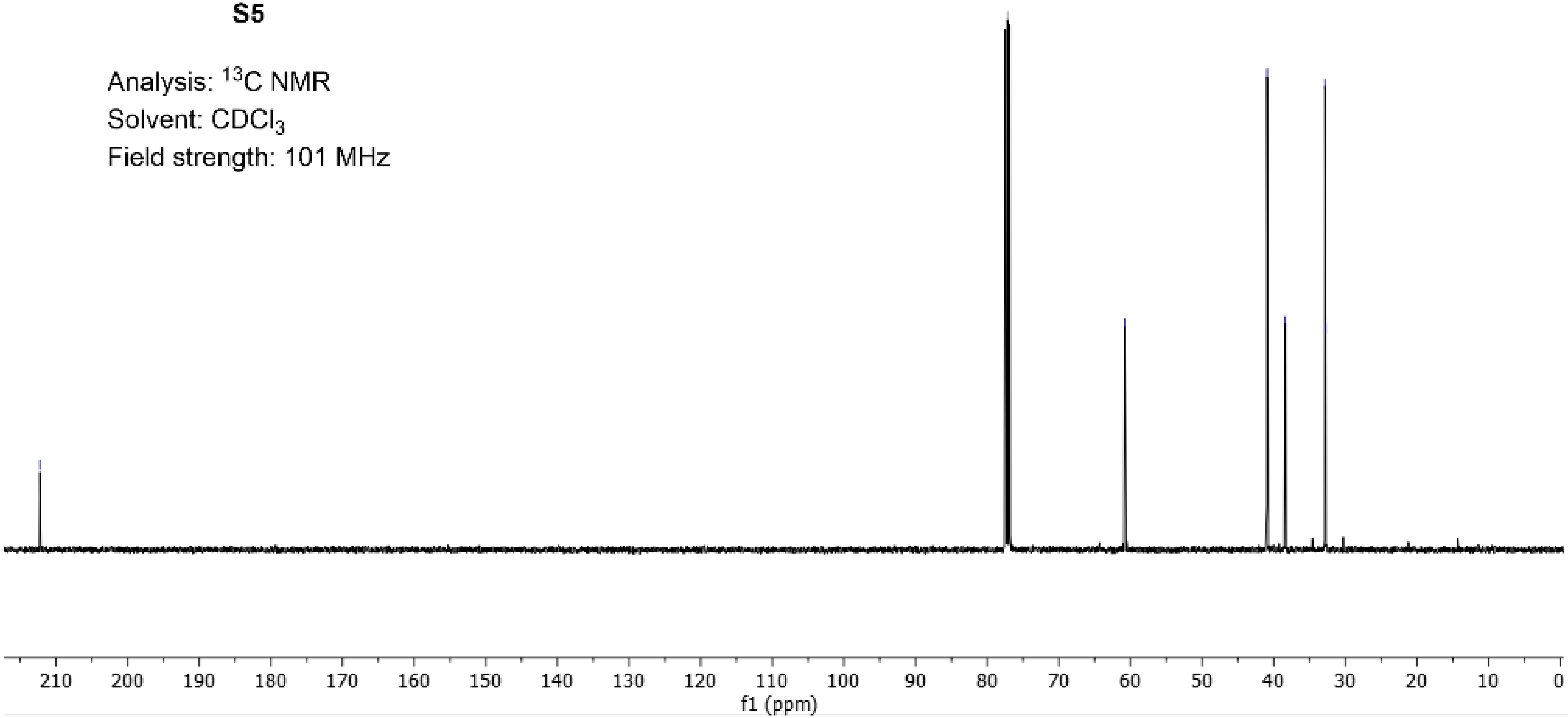

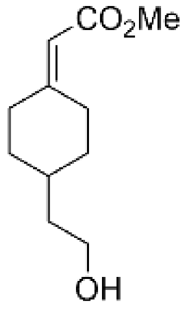

1x

Analysis: ^{1}H NMR
Solvent: $CDCl_3$
Field strength: 400 MHz

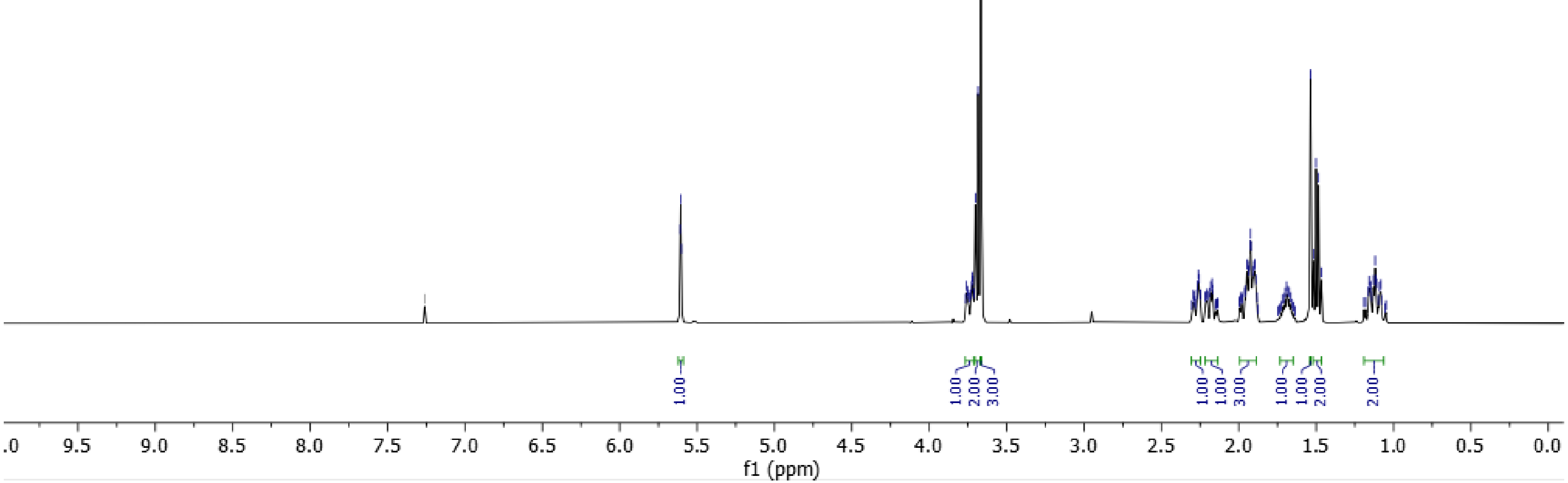

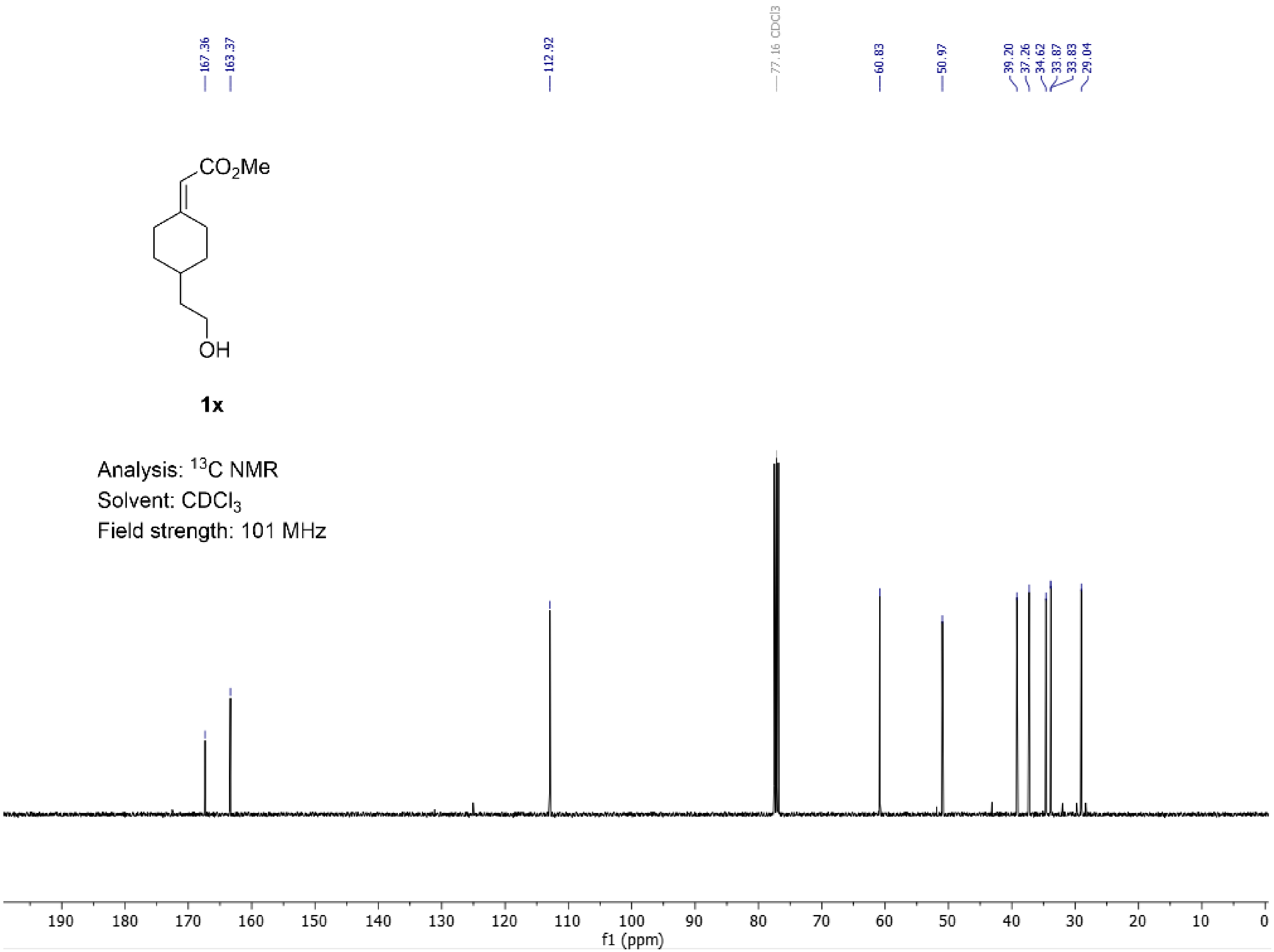
167.36
163.37
112.92
77.16 CDCl3
60.83
50.97
39.20
37.26
34.62
33.87
33.83
29.04
CO2Me
OH
1x
Analysis: 13C NMR
Solvent: CDCl3
Field strength: 101 MHz
190 180 170 160 150 140 130 120 110 100 90 80 70 60 50 40 30 20 10 0
f1 (ppm)

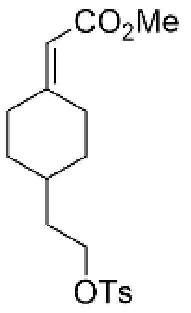

1y

Analysis: ^{1}H NMR
Solvent: $CDCl_3$
Field strength: 400 MHz

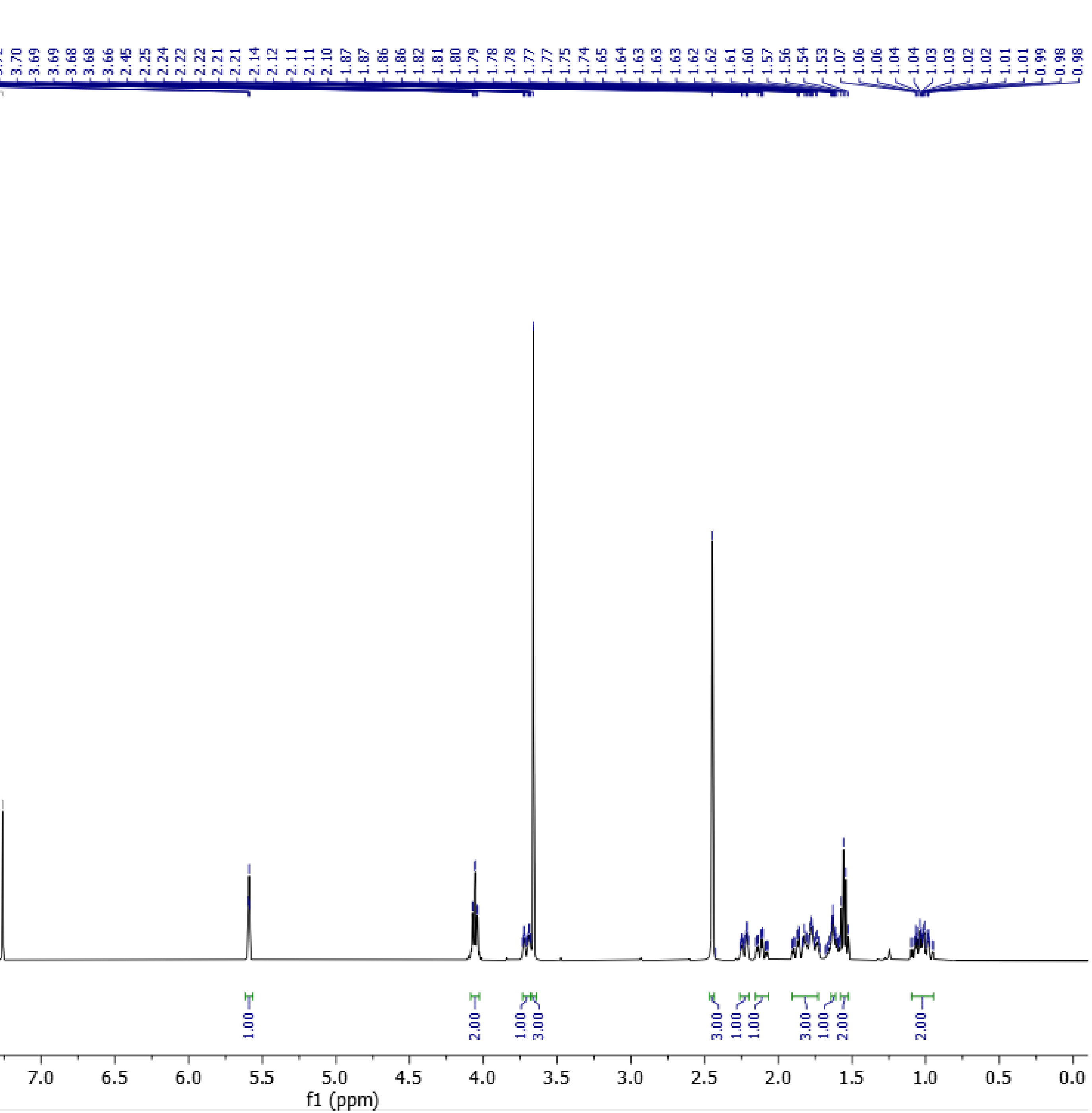

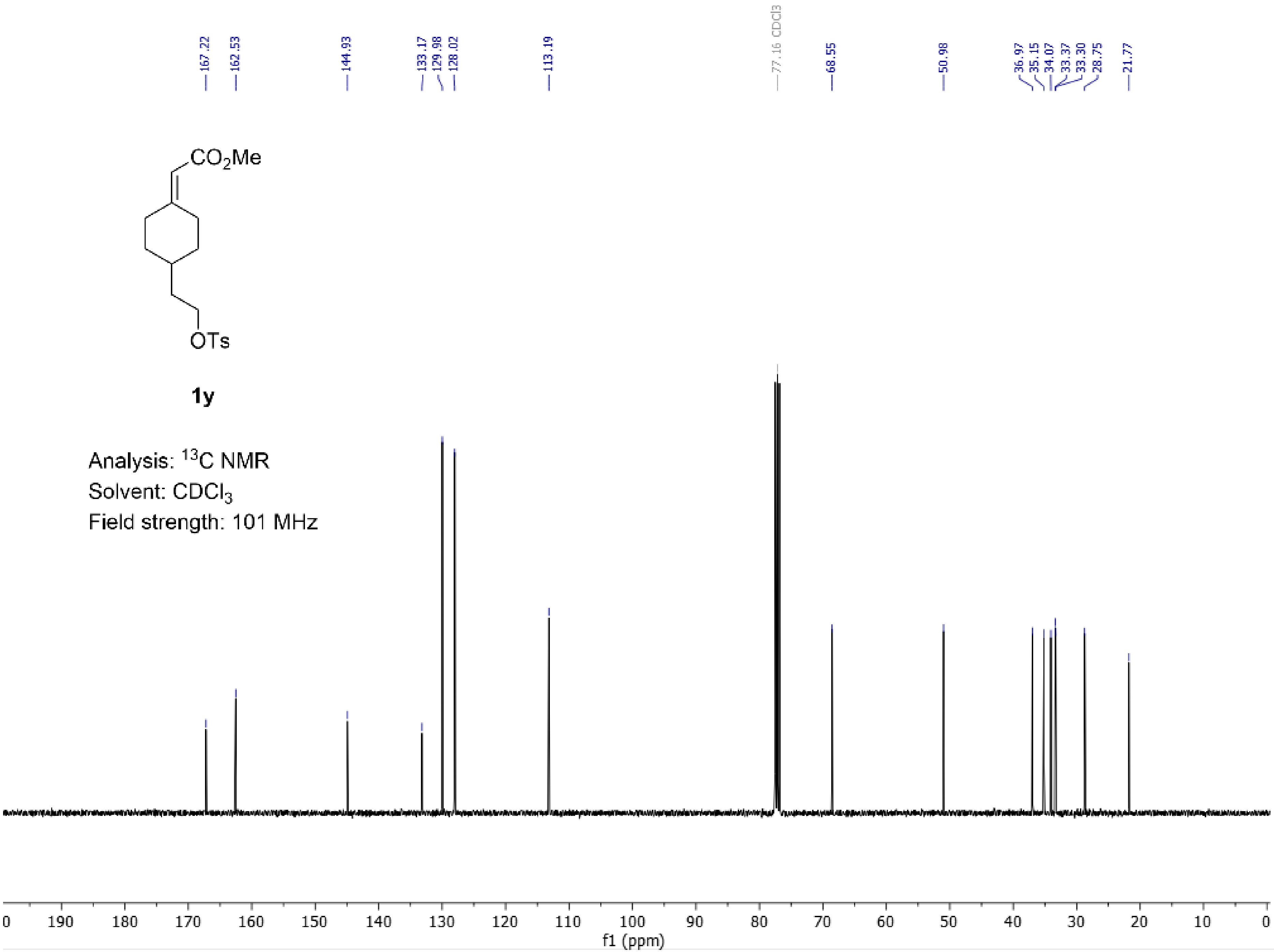
167.22
162.53
144.93
133.17
129.98
128.02
113.19
77.16 CDCl3
68.55
50.98
36.97
35.15
34.07
33.37
33.30
28.75
21.77
CO2Me
OTs
1y
Analysis: 13C NMR
Solvent: CDCl3
Field strength: 101 MHz
0
190
180
170
160
150
140
130
120
110
100
90
80
70
60
50
40
30
20
10
0
f1 (ppm)

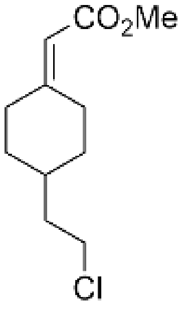

1z

Analysis: ^{1}H NMR
Solvent: $CDCl_3$
Field strength: 400 MHz

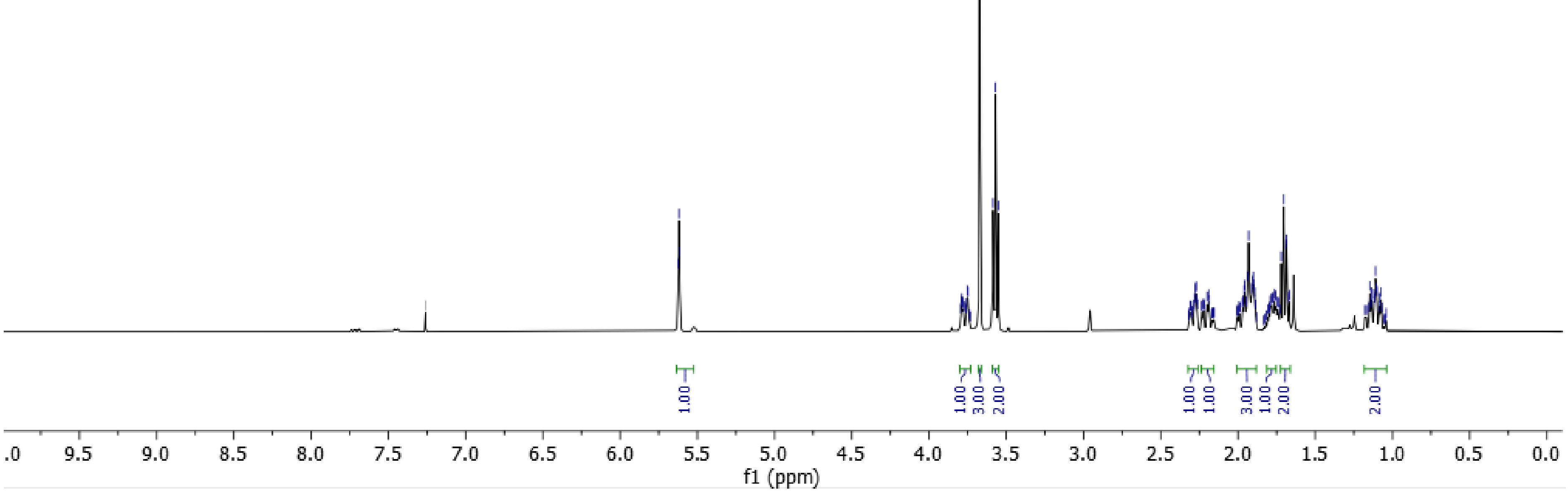

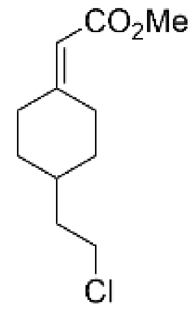

1z

Analysis: ^{13}C NMR
Solvent: $CDCl_3$
Field strength: 101 MHz

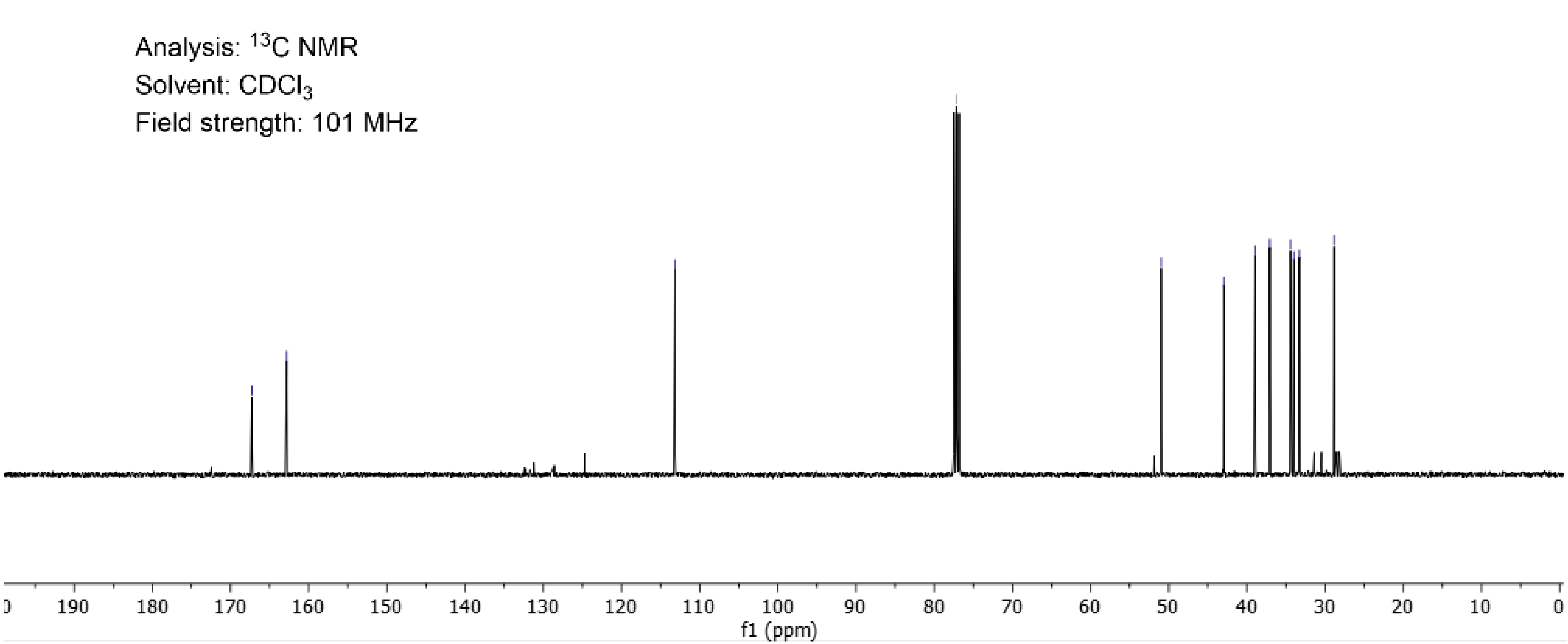

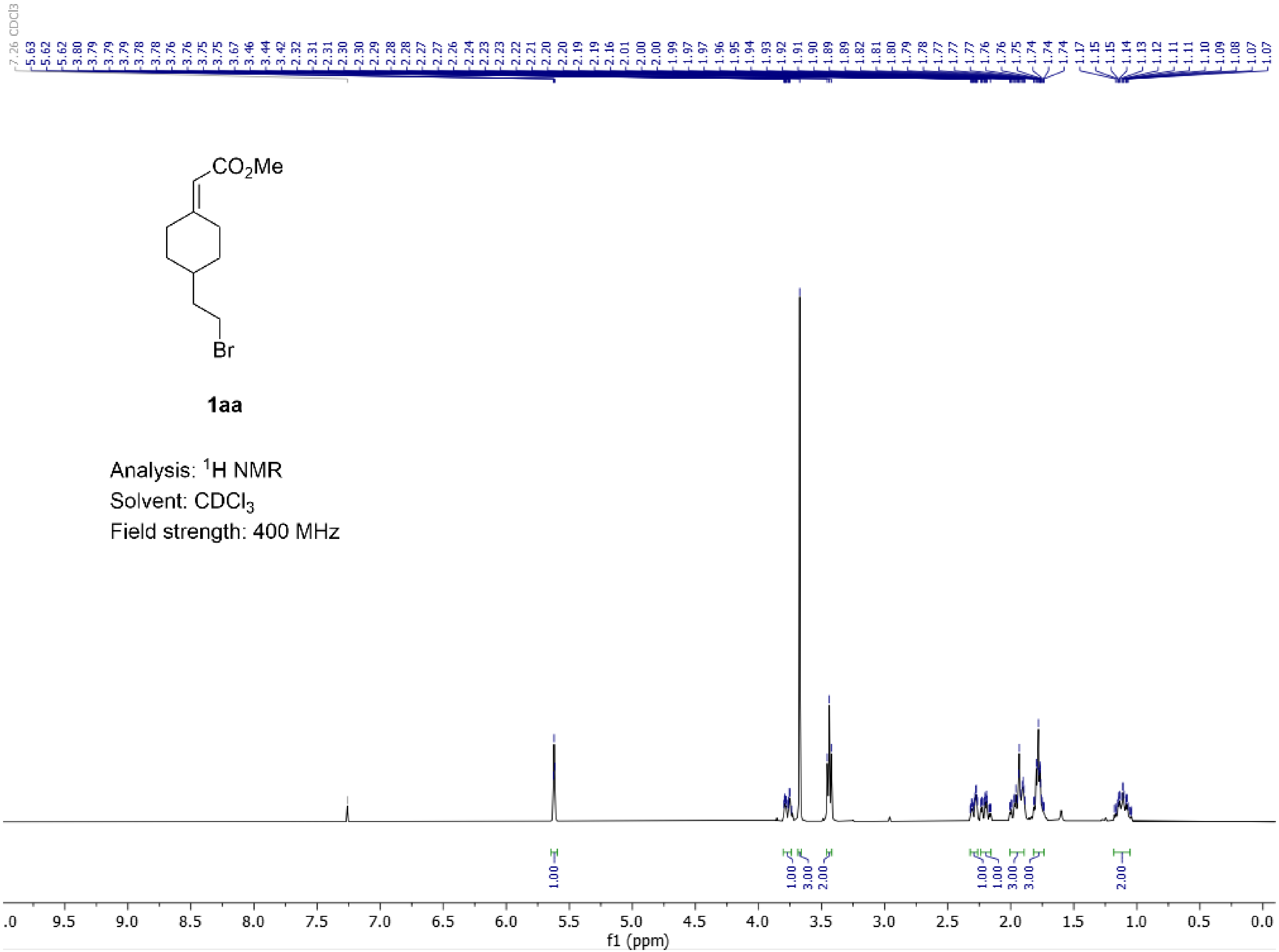
7.26 CDCl3
5.63 5.62 3.80 3.79 3.79 3.79 3.78 3.78 3.76 3.76 3.75 3.75 3.67 3.46 3.44 3.42 2.32 2.31 2.31 2.30 2.30 2.29 2.28 2.28 2.27 2.27 2.26 2.24 2.23 2.23 2.22 2.21 2.20 2.20 2.19 2.19 2.16 2.01 2.00 2.00 1.99 1.97 1.97 1.96 1.95 1.94 1.93 1.92 1.91 1.90 1.89 1.89 1.82 1.81 1.80 1.79 1.78 1.77 1.77 1.77 1.76 1.76 1.75 1.74 1.74 1.74 1.17 1.15 1.15 1.14 1.13 1.12 1.11 1.11 1.10 1.09 1.08 1.07 1.07
CO2Me
Br
1aa
Analysis: 1H NMR
Solvent: CDCl3
Field strength: 400 MHz
1.00
1.00
3.00
2.00
1.00
1.00
3.00
3.00
2.00
.0 9.5 9.0 8.5 8.0 7.5 7.0 6.5 6.0 5.5 5.0 4.5 4.0 3.5 3.0 2.5 2.0 1.5 1.0 0.5 0.0
f1 (ppm)

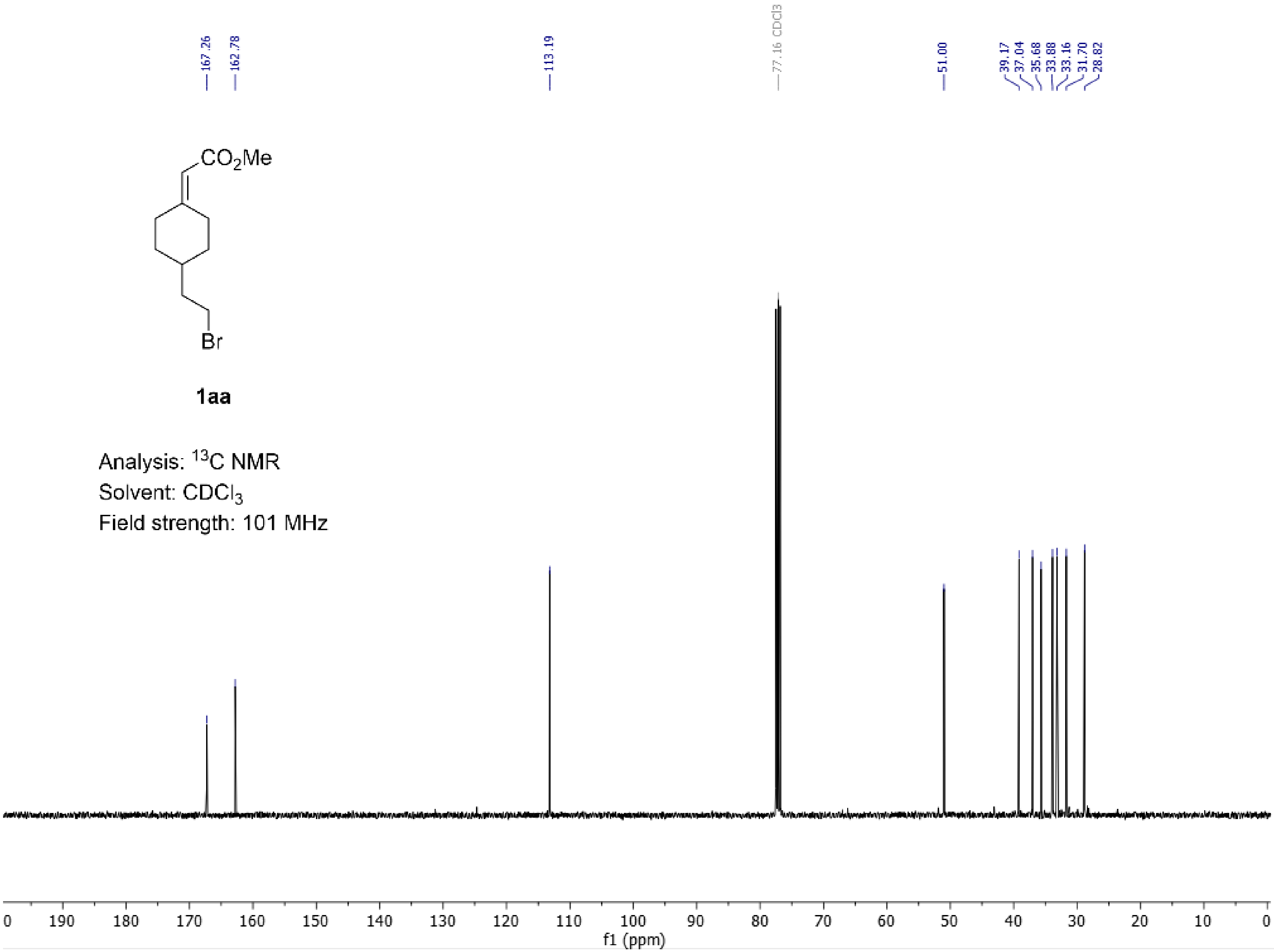

167.26
162.78
113.19
77.16 CDCl3
51.00
39.17
37.04
35.68
33.88
33.16
31.70
28.82
CO2Me
Br
1aa
Analysis: 13C NMR
Solvent: CDCl3
Field strength: 101 MHz
f1 (ppm)

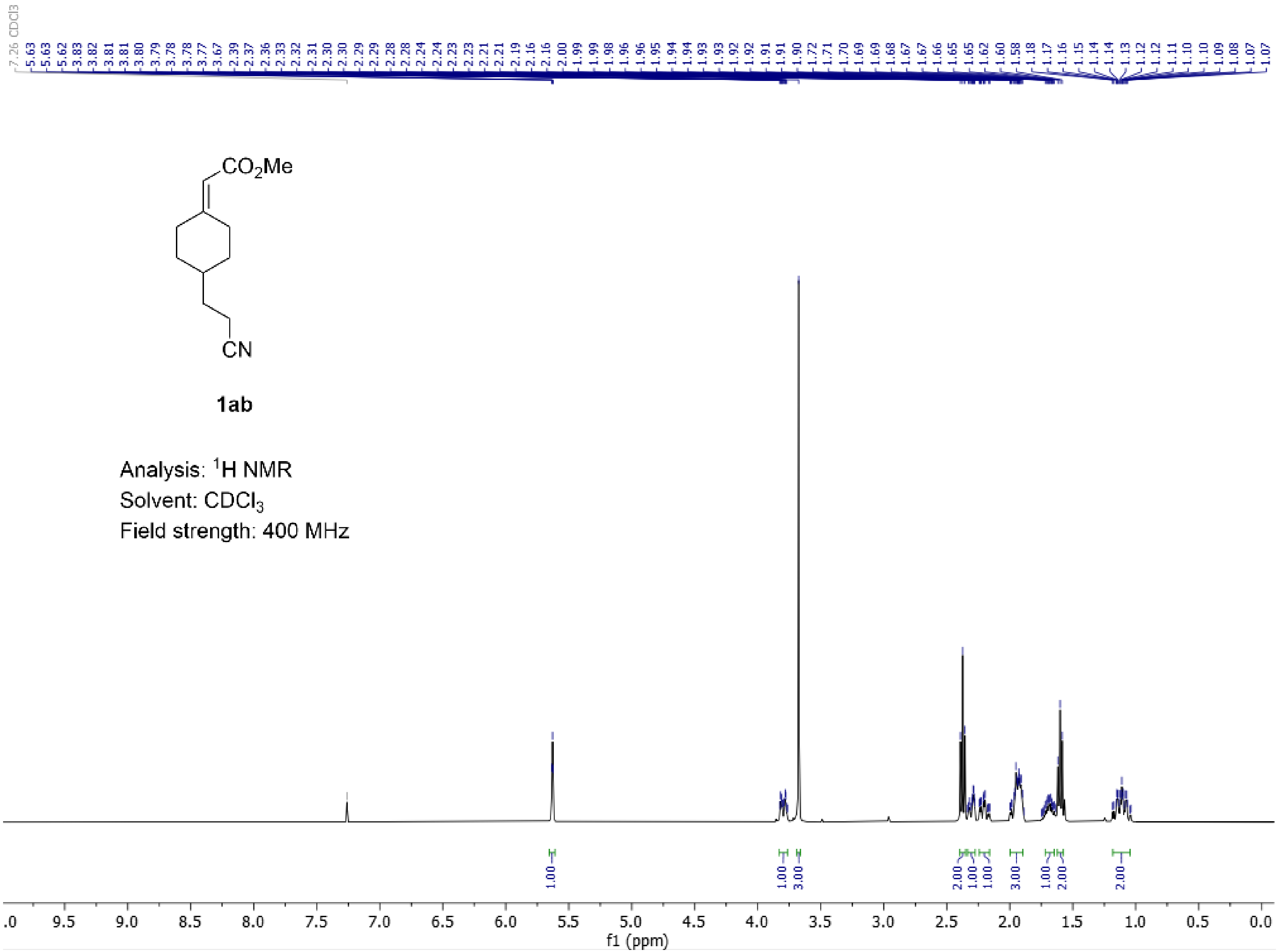
7.26 CDCl3
5.63 5.63 5.62 3.83 3.82 3.81 3.81 3.80 3.79 3.78 3.78 3.77 3.67 2.39 2.37 2.36 2.33 2.32 2.31 2.30 2.30 2.29 2.29 2.28 2.28 2.24 2.24 2.23 2.23 2.21 2.21 2.19 2.16 2.16 2.00 1.99 1.99 1.98 1.96 1.96 1.95 1.94 1.94 1.93 1.93 1.92 1.92 1.91 1.91 1.90 1.72 1.71 1.70 1.69 1.69 1.68 1.67 1.67 1.66 1.65 1.65 1.62 1.60 1.58 1.18 1.17 1.16 1.15 1.14 1.14 1.13 1.12 1.12 1.11 1.10 1.09 1.08 1.07 1.07
CO2Me
CN
1ab
Analysis: 1H NMR
Solvent: CDCl3
Field strength: 400 MHz
1.00 1.00 3.00 2.00 1.00 1.00 3.00 1.00 2.00 2.00
.0 9.5 9.0 8.5 8.0 7.5 7.0 6.5 6.0 5.5 5.0 4.5 4.0 3.5 3.0 2.5 2.0 1.5 1.0 0.5 0.0
f1 (ppm)

1ab

Analysis: ^{13}C NMR
Solvent: $CDCl_3$
Field strength: 101 MHz

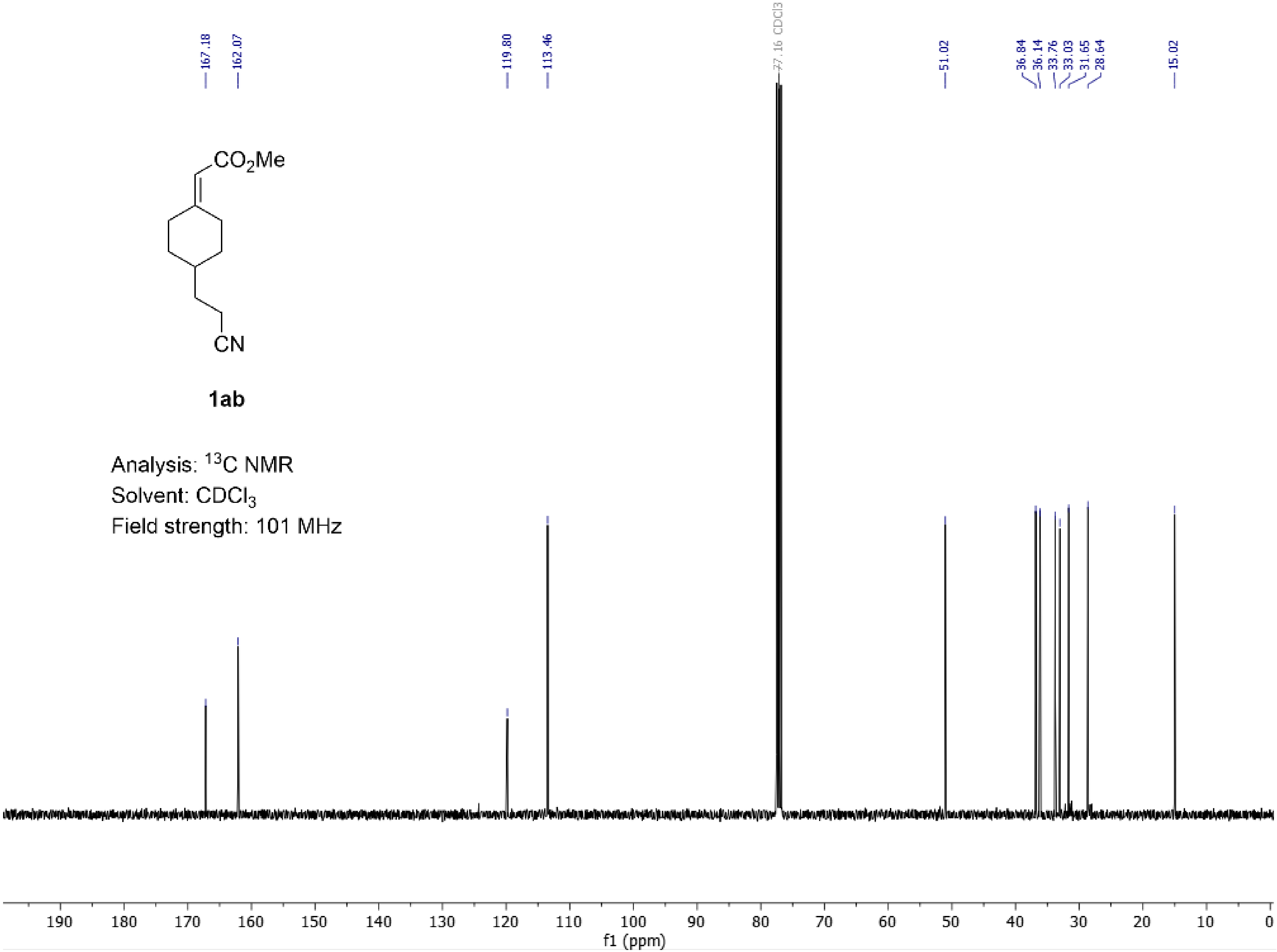

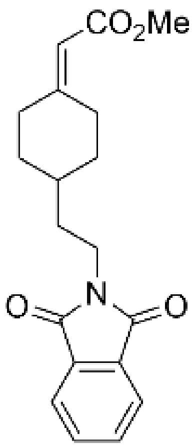

1ac

Analysis: ^{1}H NMR
Solvent: $CDCl_3$
Field strength: 400 MHz

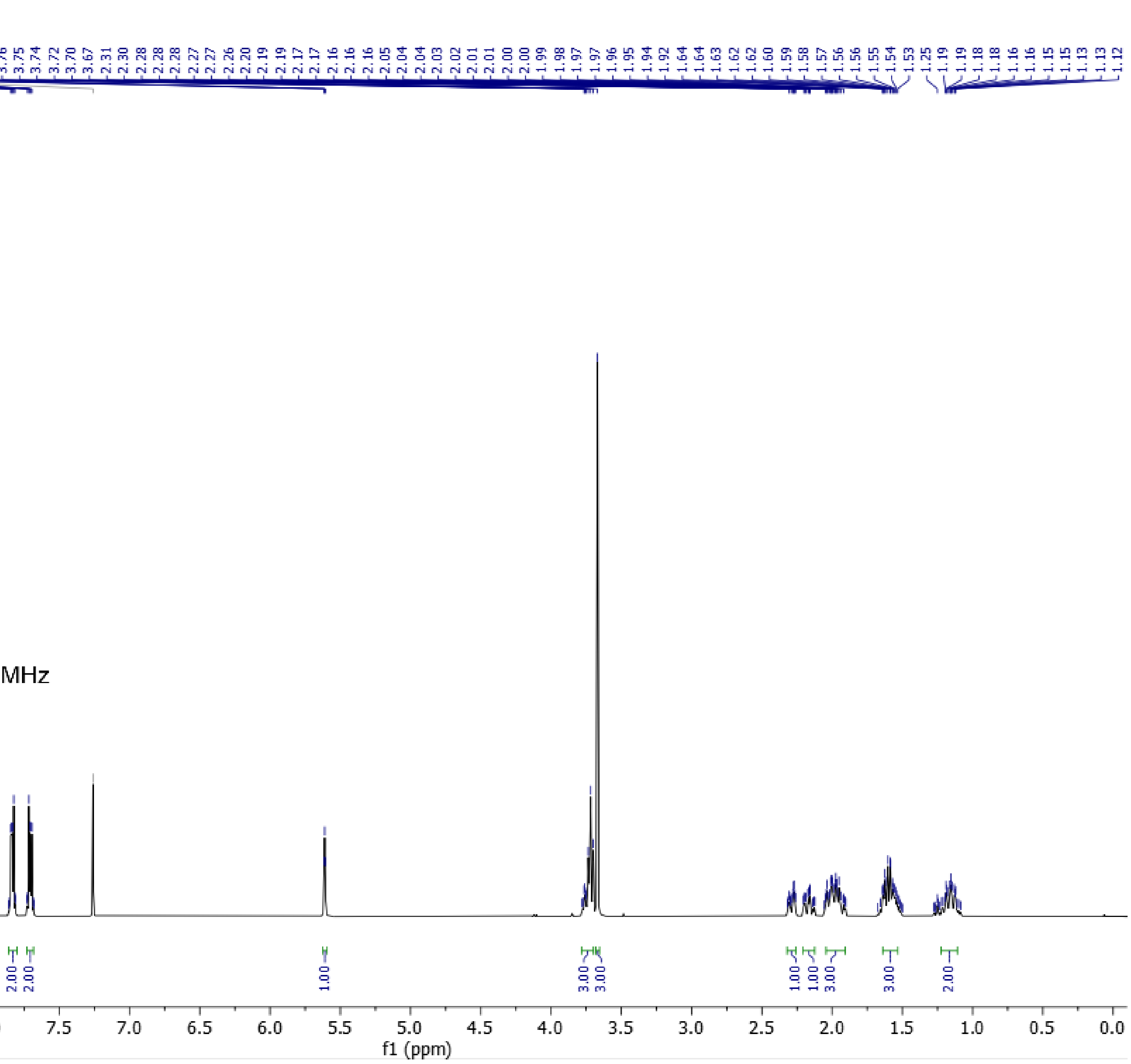

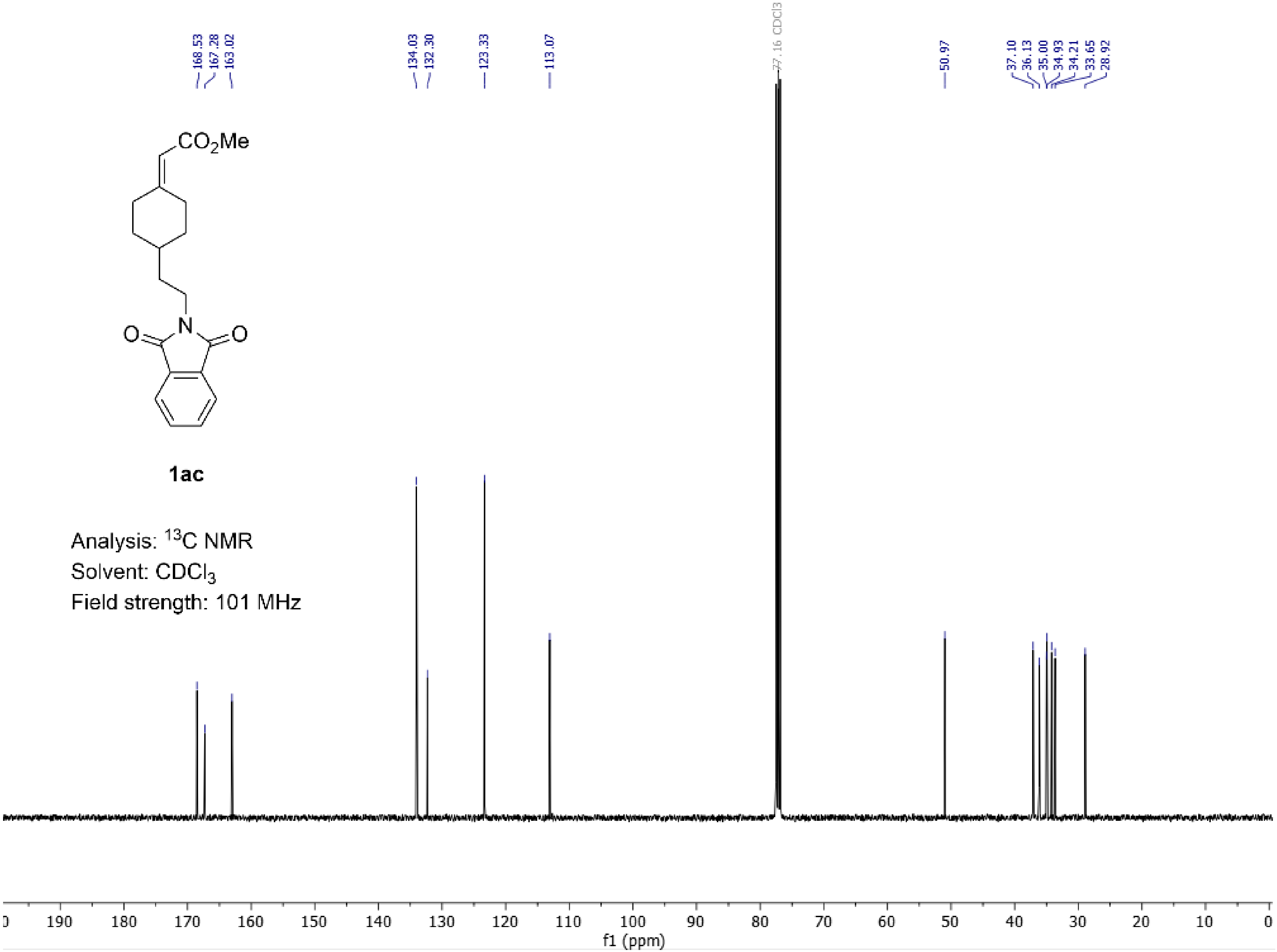
168.53
167.28
163.02
134.03
132.30
123.33
113.07
77.16 CDCl3
50.97
37.10
36.13
35.00
34.93
34.21
33.65
28.92
CO2Me
N
O
O
1ac
Analysis: 13C NMR
Solvent: CDCl3
Field strength: 101 MHz
190
180
170
160
150
140
130
120
110
100
90
80
70
60
50
40
30
20
10
0
f1 (ppm)

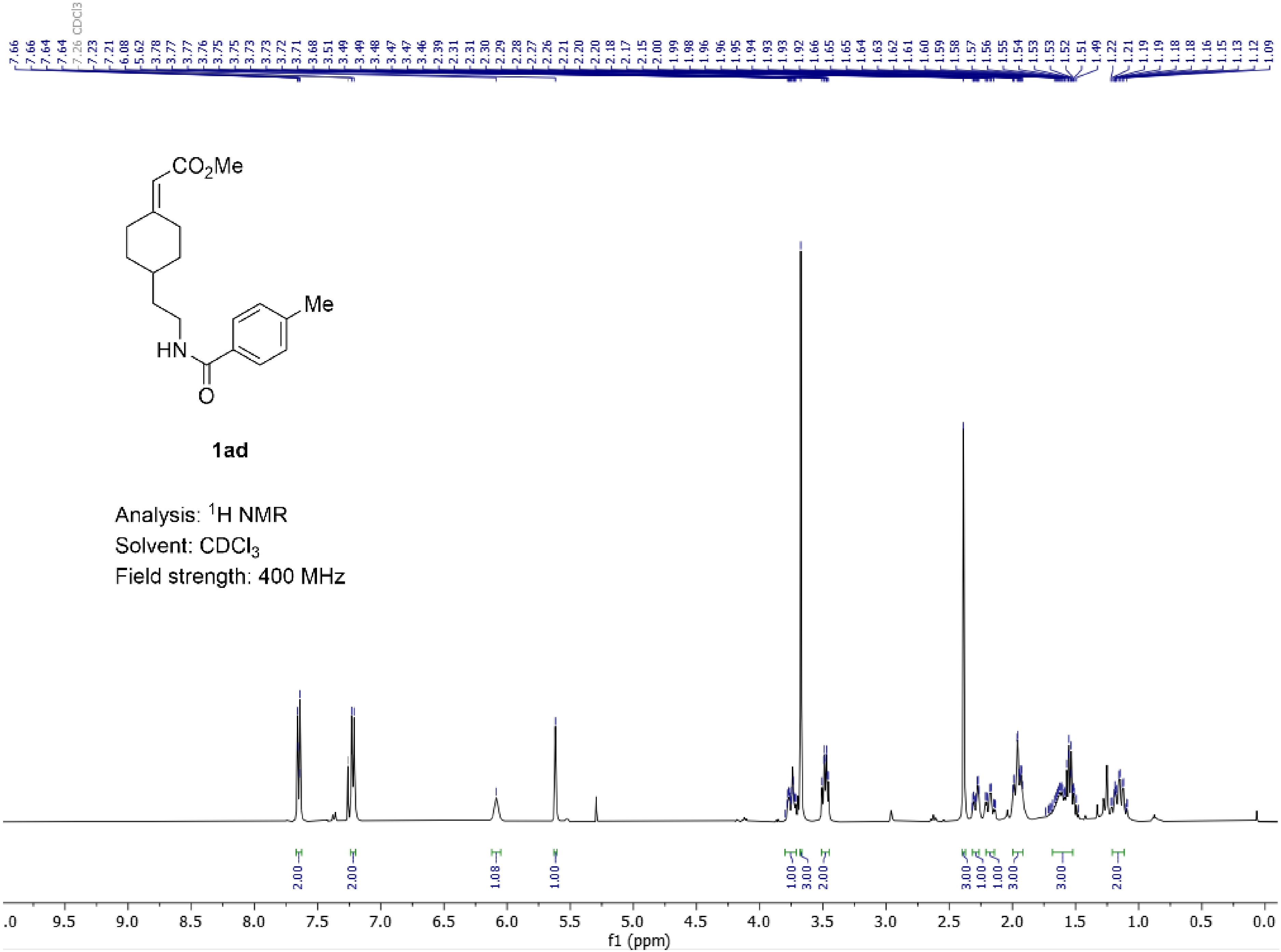

1ad

Analysis: ^{1}H NMR
Solvent: $CDCl_3$
Field strength: 400 MHz

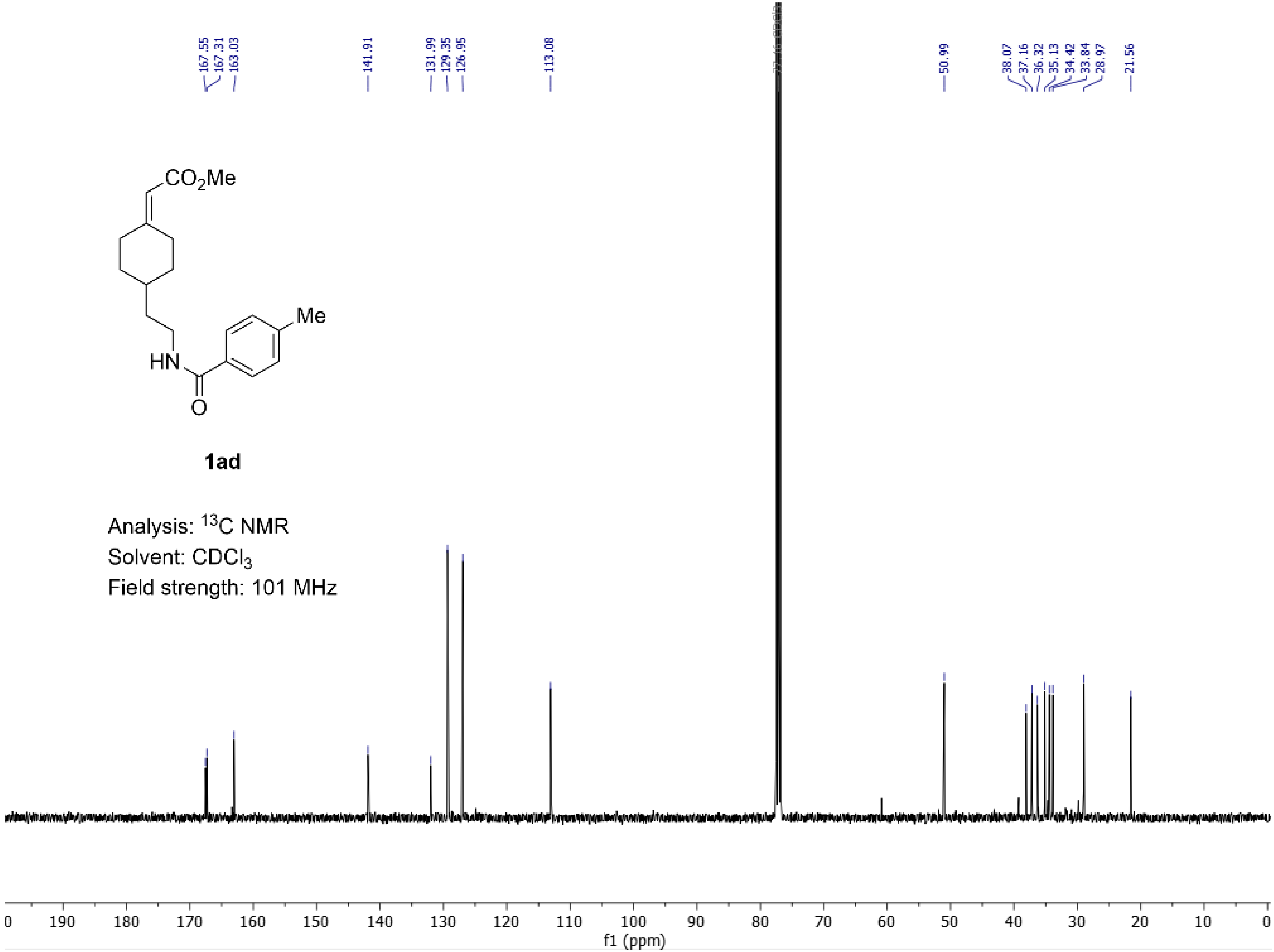
167.55
167.31
163.03
141.91
131.99
129.35
126.95
113.08
50.99
38.07
37.16
36.32
35.13
34.42
33.84
28.97
21.56
CO2Me
Me
HN
O
1ad
Analysis: 13C NMR
Solvent: CDCl3
Field strength: 101 MHz
0 190 180 170 160 150 140 130 120 110 100 90 80 70 60 50 40 30 20 10 0
f1 (ppm)

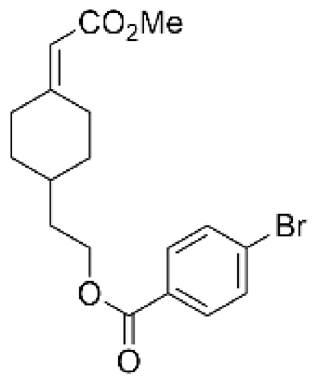

1ae

Analysis: ^{1}H NMR

Solvent: $CDCl_3$

Field strength: 400 MHz

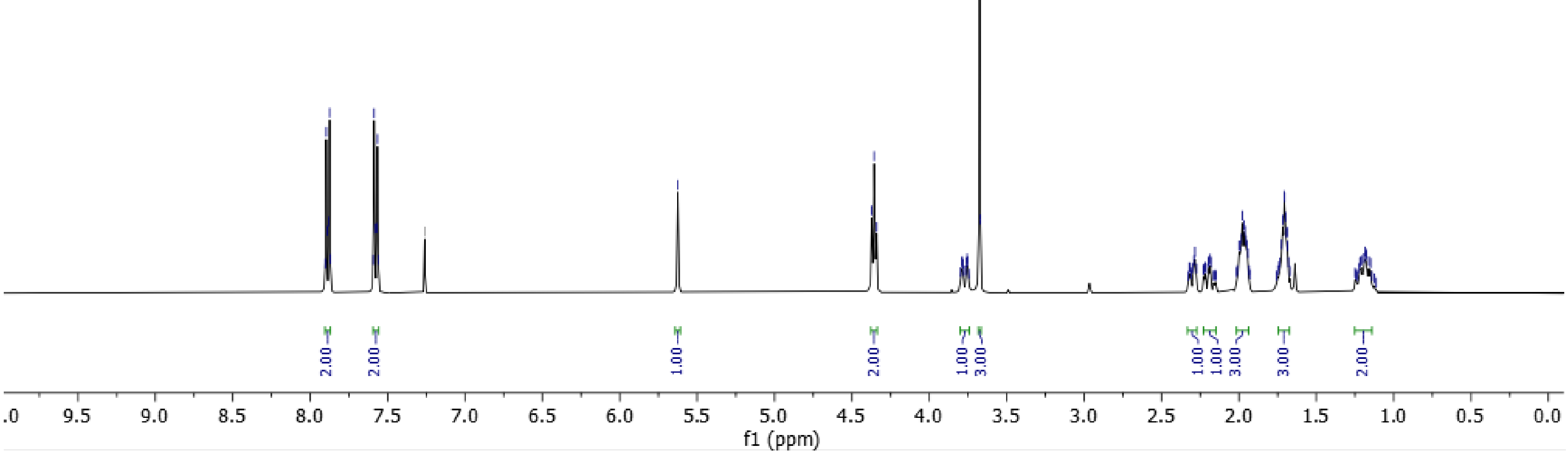

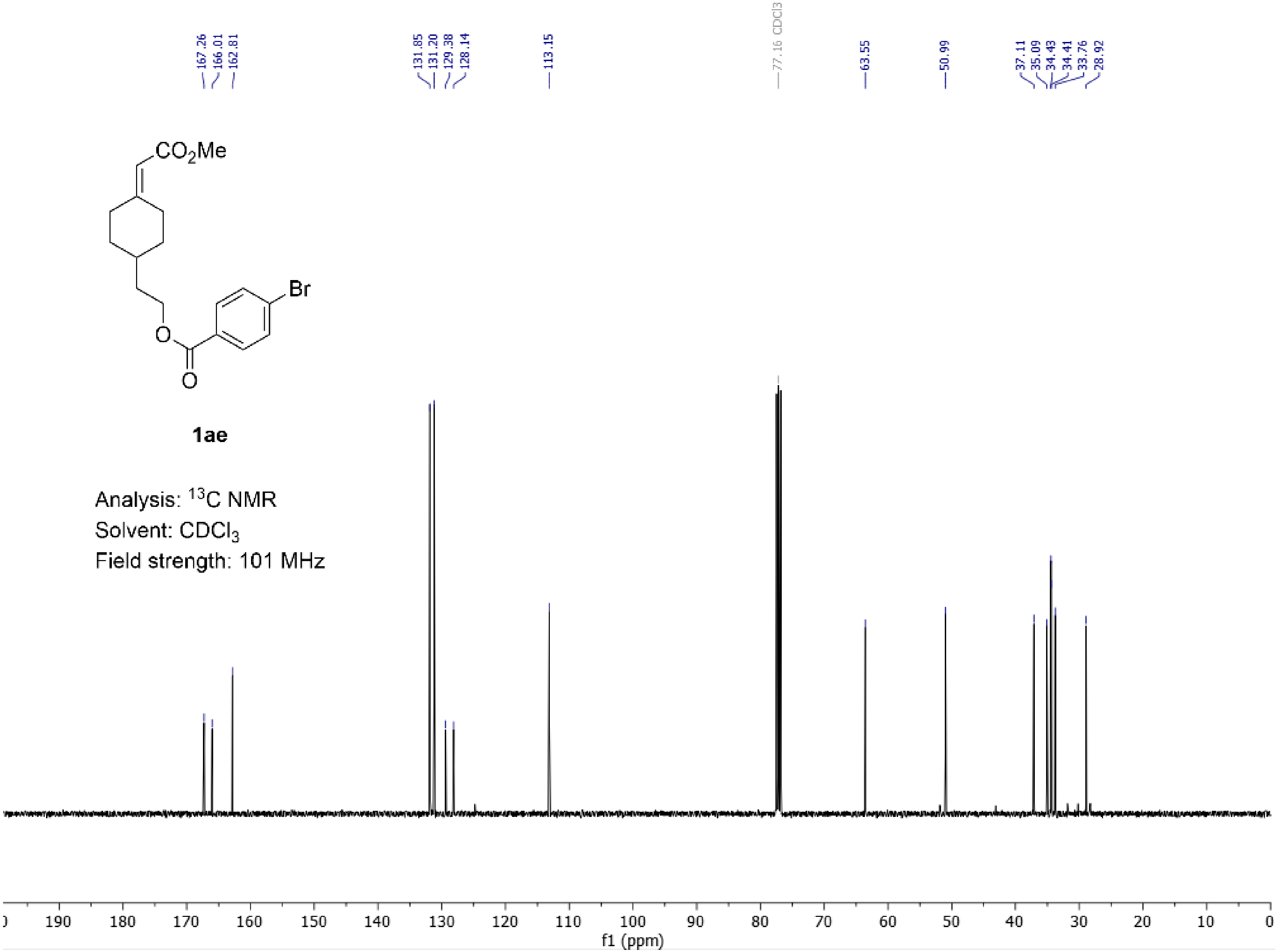
167.26
166.01
162.81
131.85
131.20
129.38
128.14
113.15
77.16 CDCl3
63.55
50.99
37.11
35.09
34.43
34.41
33.76
28.92
CO2Me
Br
O
O
1ae
Analysis: 13C NMR
Solvent: CDCl3
Field strength: 101 MHz
190
180
170
160
150
140
130
120
110
100
90
80
70
60
50
40
30
20
10
0
f1 (ppm)

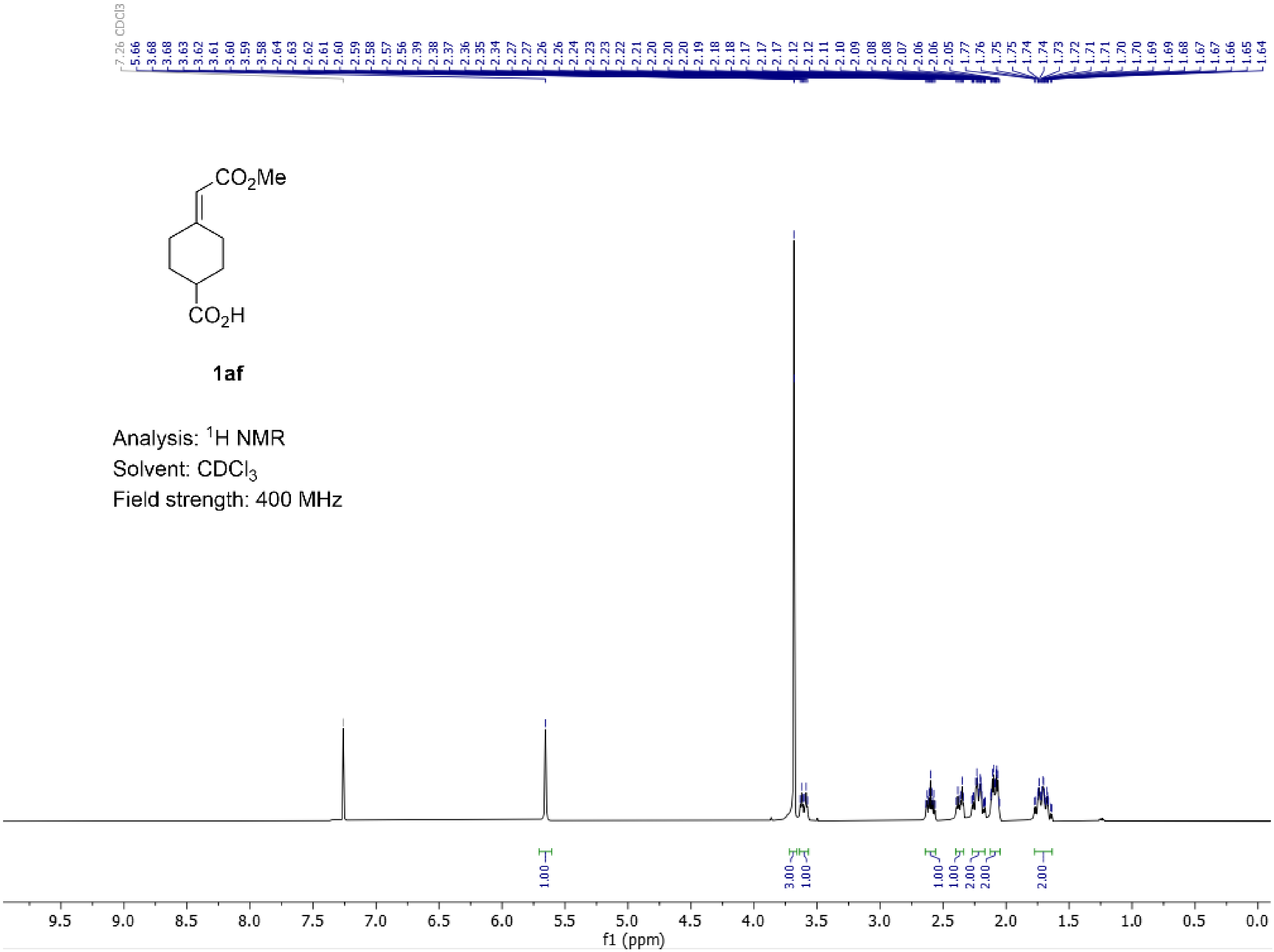
CO2Me
CO2H
1af
Analysis: 1H NMR
Solvent: CDCl3
Field strength: 400 MHz
f1 (ppm)

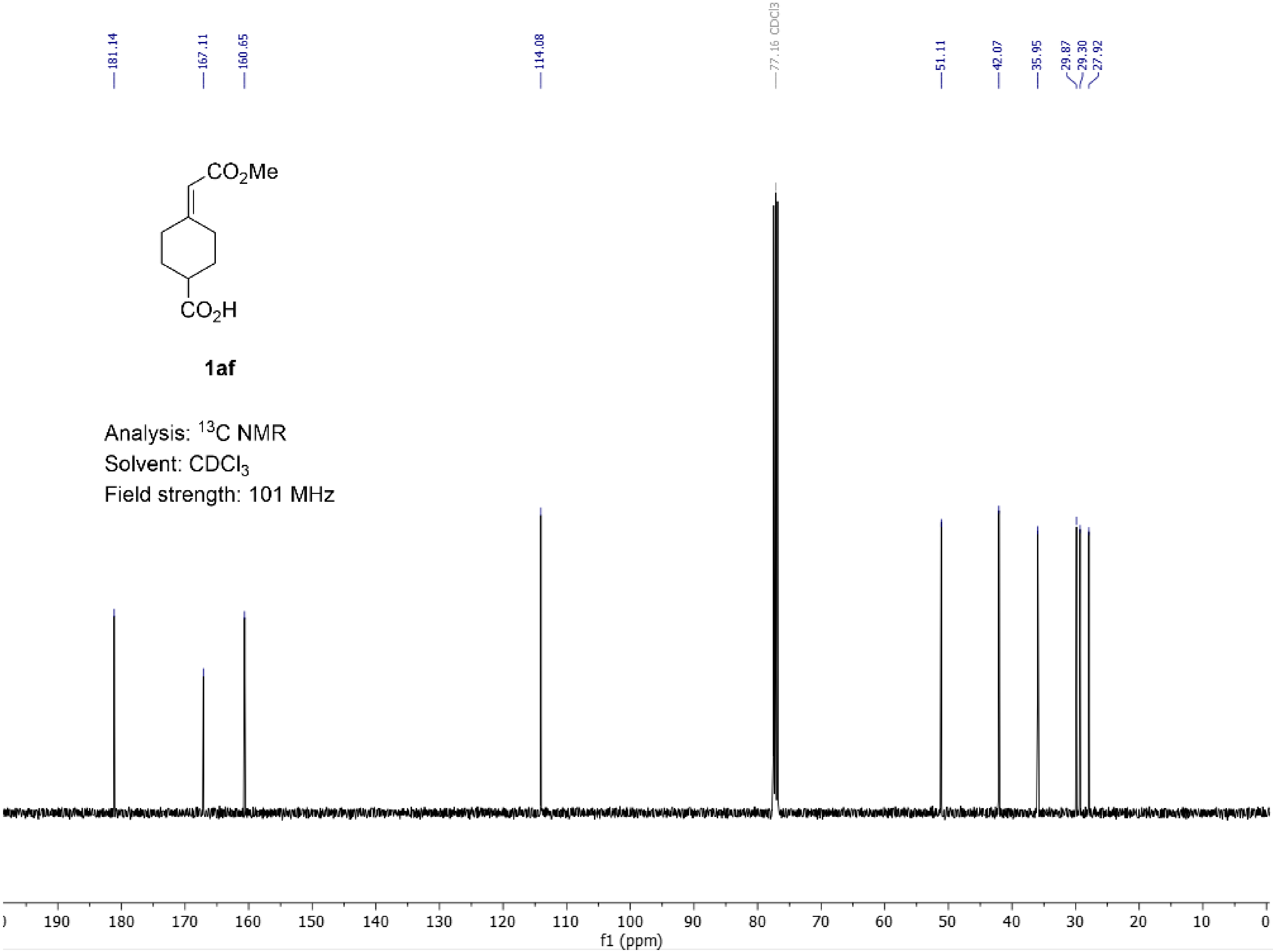
181.14
167.11
160.65
114.08
77.16 CDCl3
51.11
42.07
35.95
29.87
29.30
27.92
CO2Me
CO2H
1af
Analysis: 13C NMR
Solvent: CDCl3
Field strength: 101 MHz
190 180 170 160 150 140 130 120 110 100 90 80 70 60 50 40 30 20 10 0
f1 (ppm)

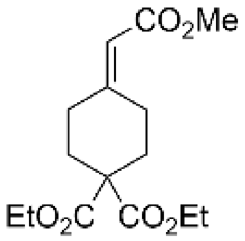

1ag

Analysis: ^{1}H NMR
Solvent: $CDCl_3$
Field strength: 400 MHz

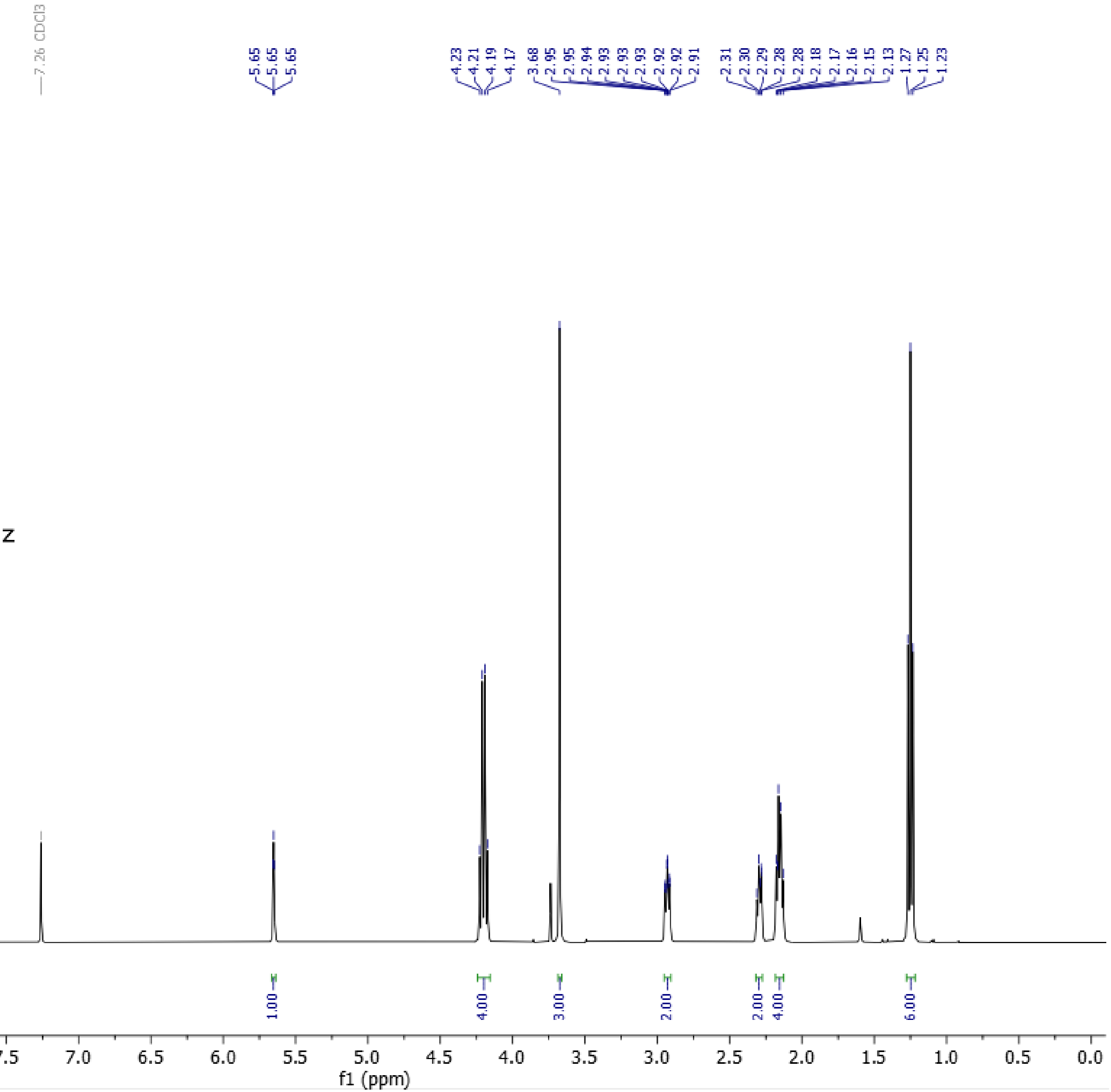

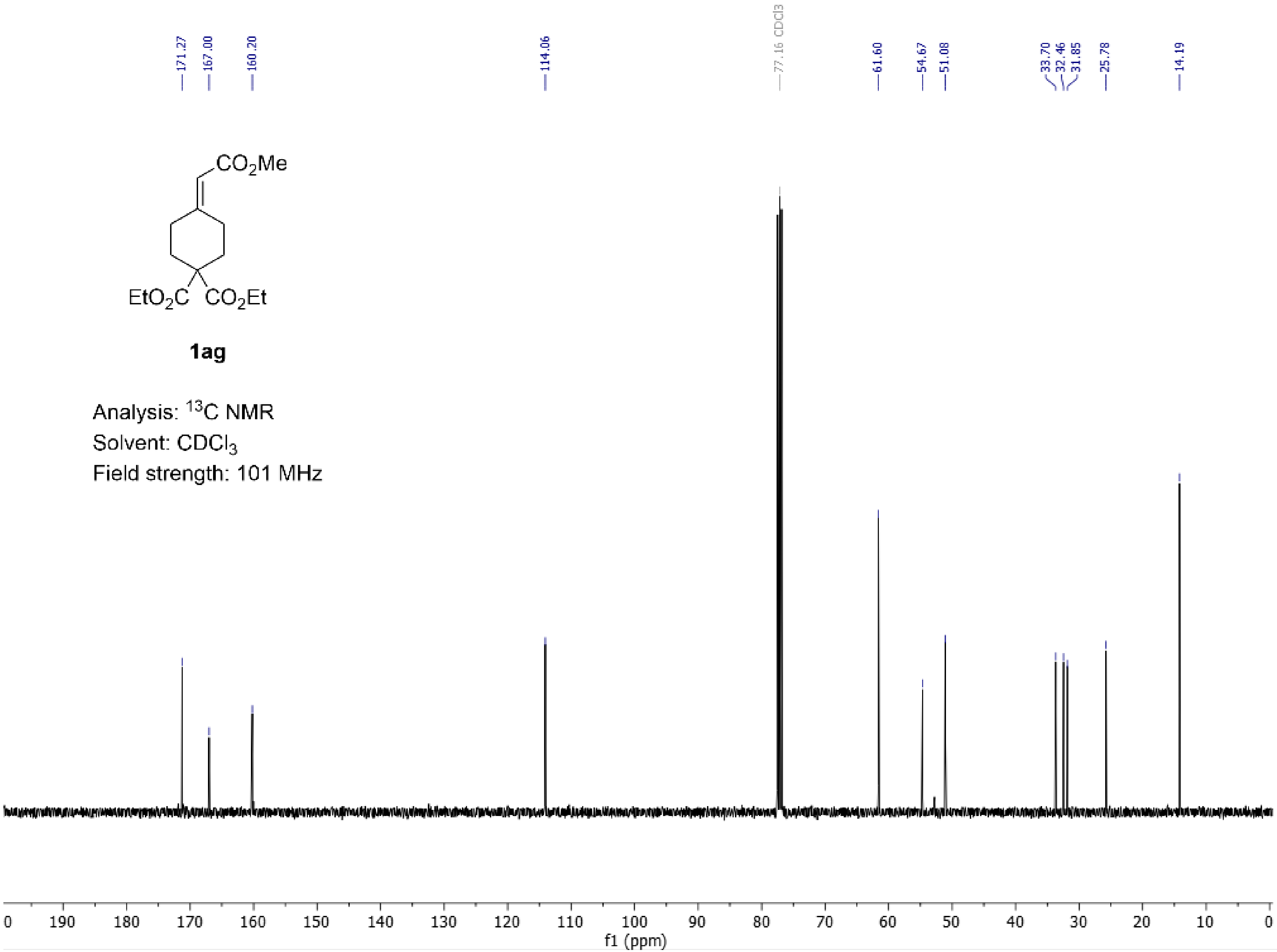
171.27
167.00
160.20
114.06
77.16 CDCl3
61.60
54.67
51.08
33.70
32.46
31.85
25.78
14.19
CO_2Me
EtO_2C CO_2Et
1ag
Analysis: ^{13}C NMR
Solvent: $CDCl_3$
Field strength: 101 MHz
0 190 180 170 160 150 140 130 120 110 100 90 80 70 60 50 40 30 20 10 0
f1 (ppm)

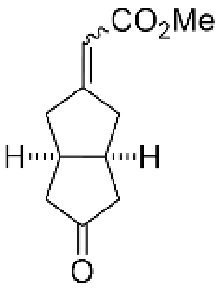

1ah

Analysis: ^{1}H NMR
Solvent: $CDCl_3$
Field strength: 400 MHz

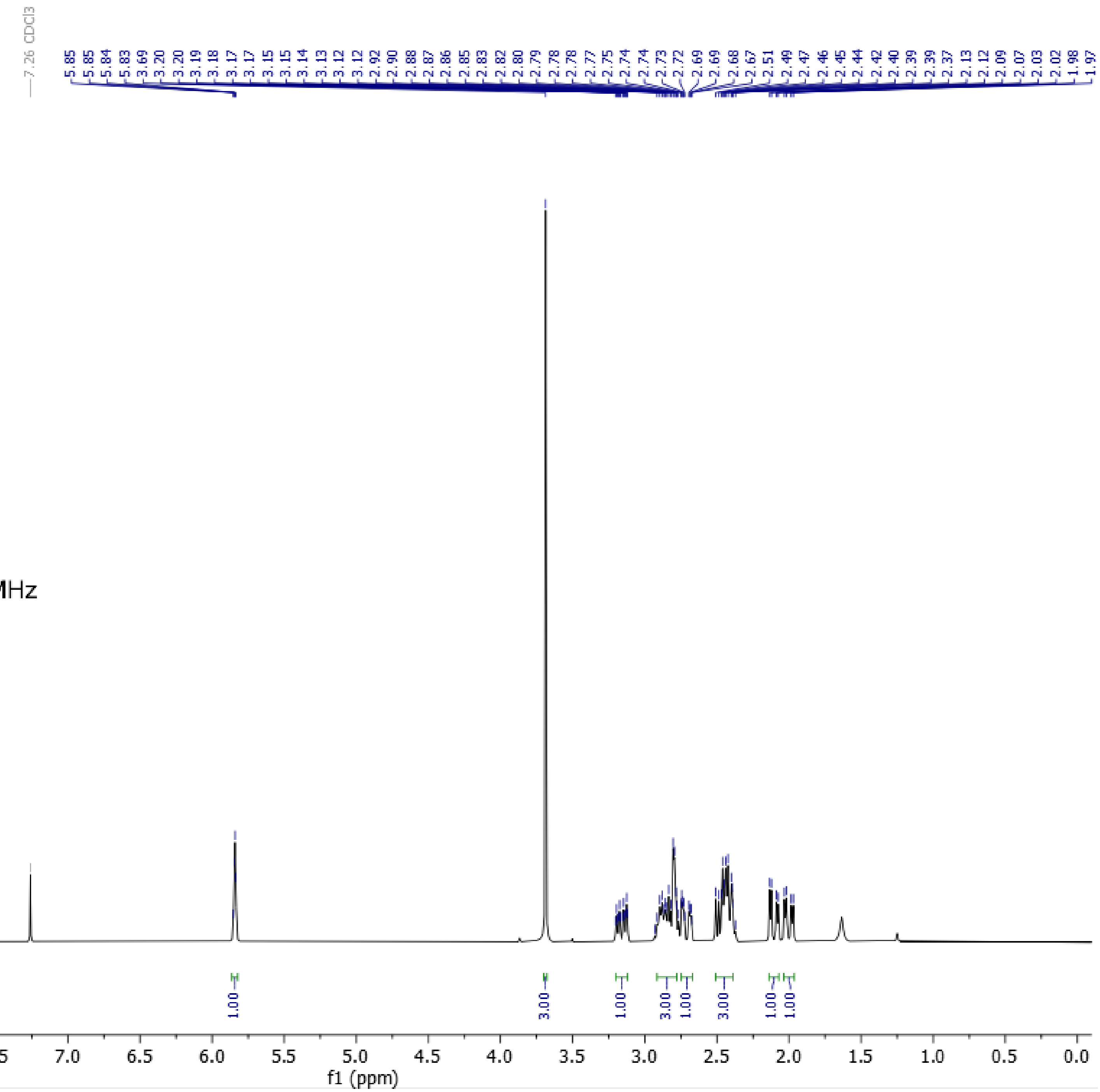

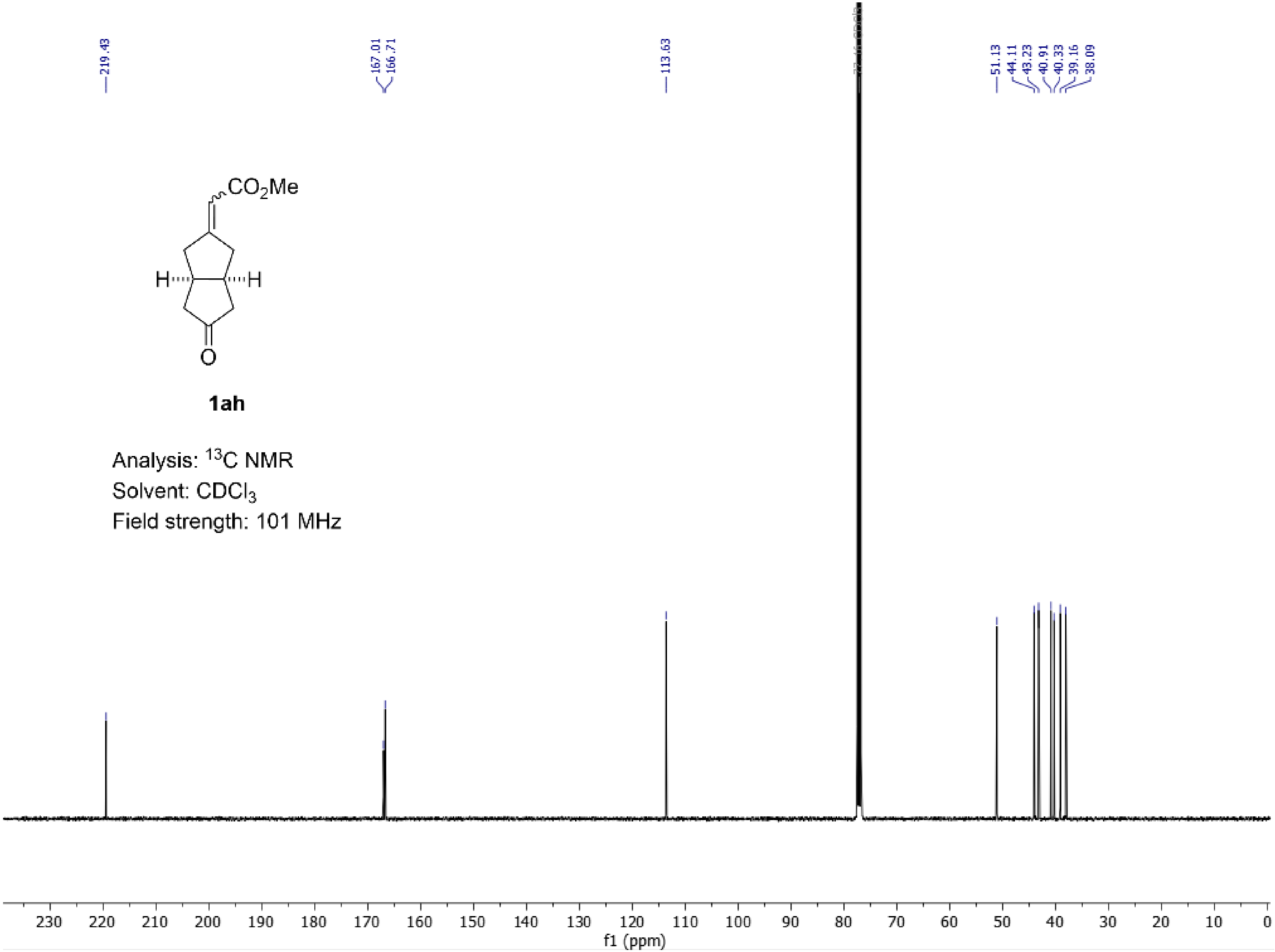

1ah

Analysis: ^{13}C NMR
Solvent: $CDCl_3$
Field strength: 101 MHz

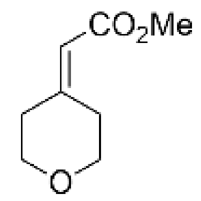

1ai

Analysis: ^{1}H NMR
Solvent: $CDCl_3$
Field strength: 400 MHz

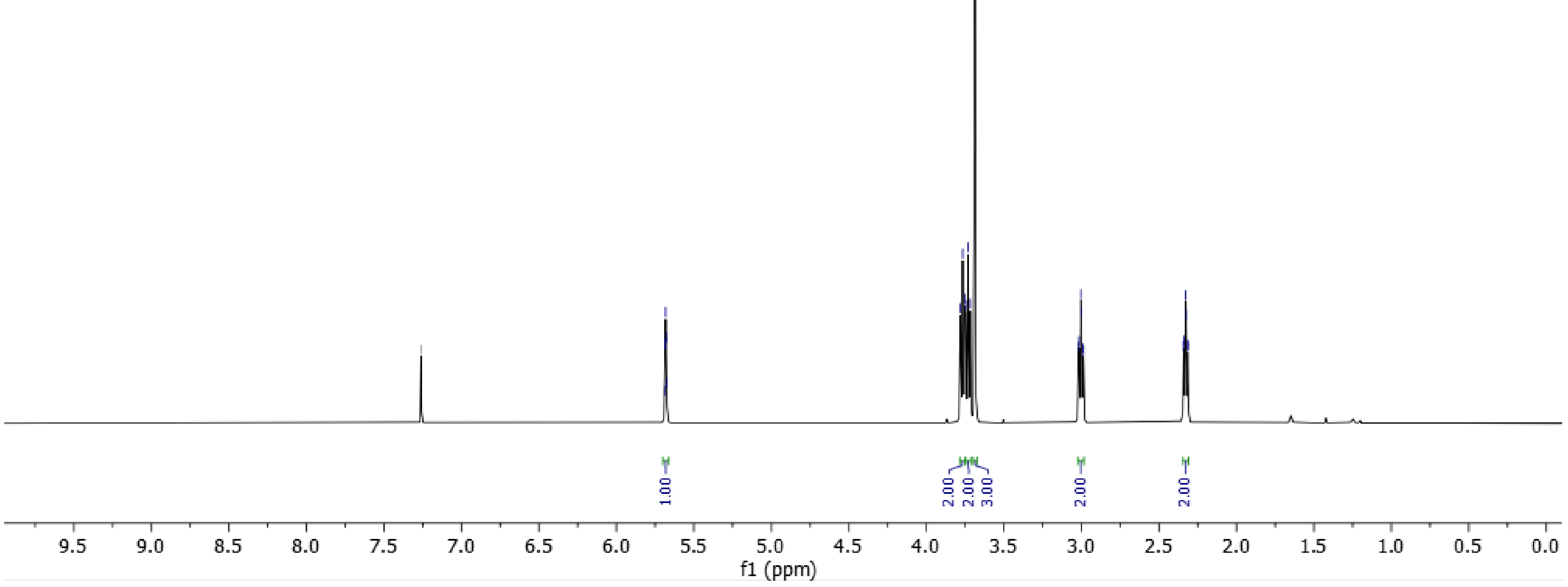

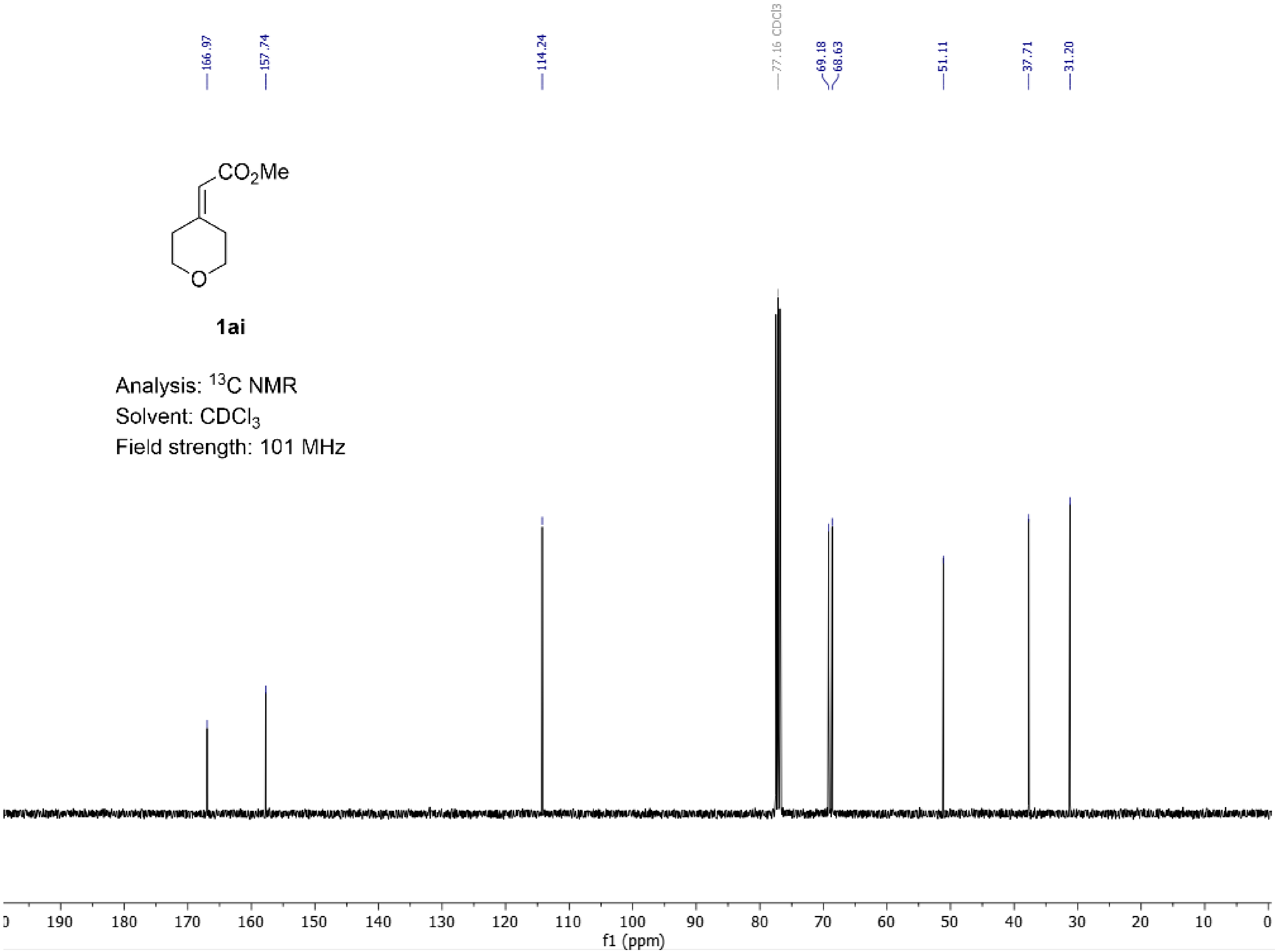
166.97
157.74
114.24
77.16 CDCl3
69.18
68.63
51.11
37.71
31.20
CO2Me
O
1ai
Analysis: 13C NMR
Solvent: CDCl3
Field strength: 101 MHz
190 180 170 160 150 140 130 120 110 100 90 80 70 60 50 40 30 20 10 0
f1 (ppm)

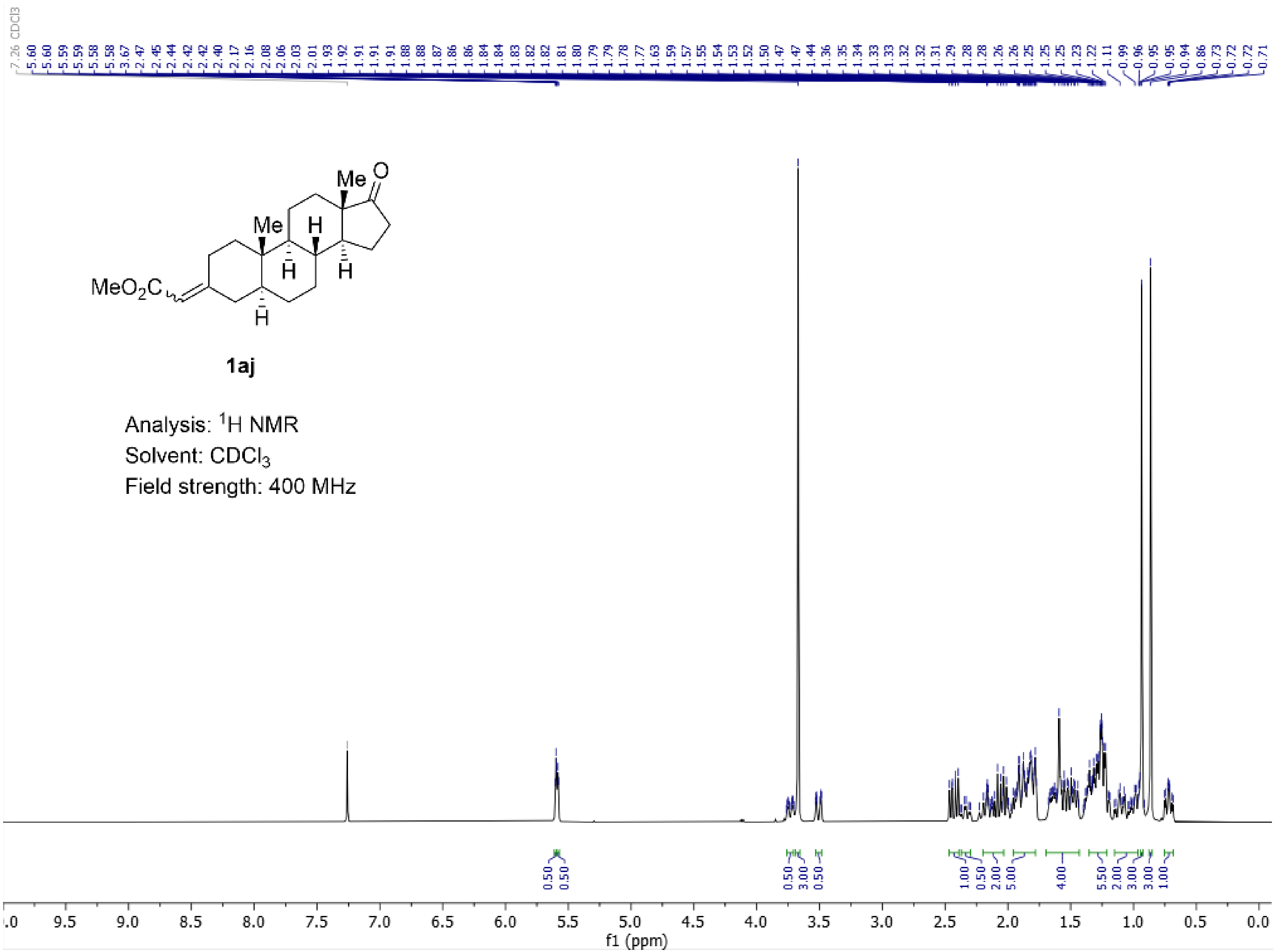

1aj

Analysis: ^{1}H NMR
Solvent: $CDCl_3$
Field strength: 400 MHz

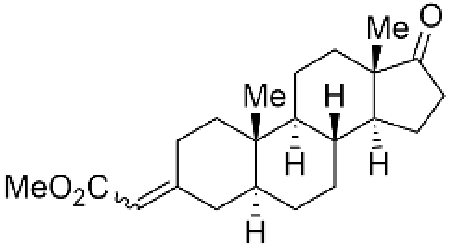

1aj

Analysis: ^{13}C NMR
Solvent: $CDCl_3$
Field strength: 101 MHz

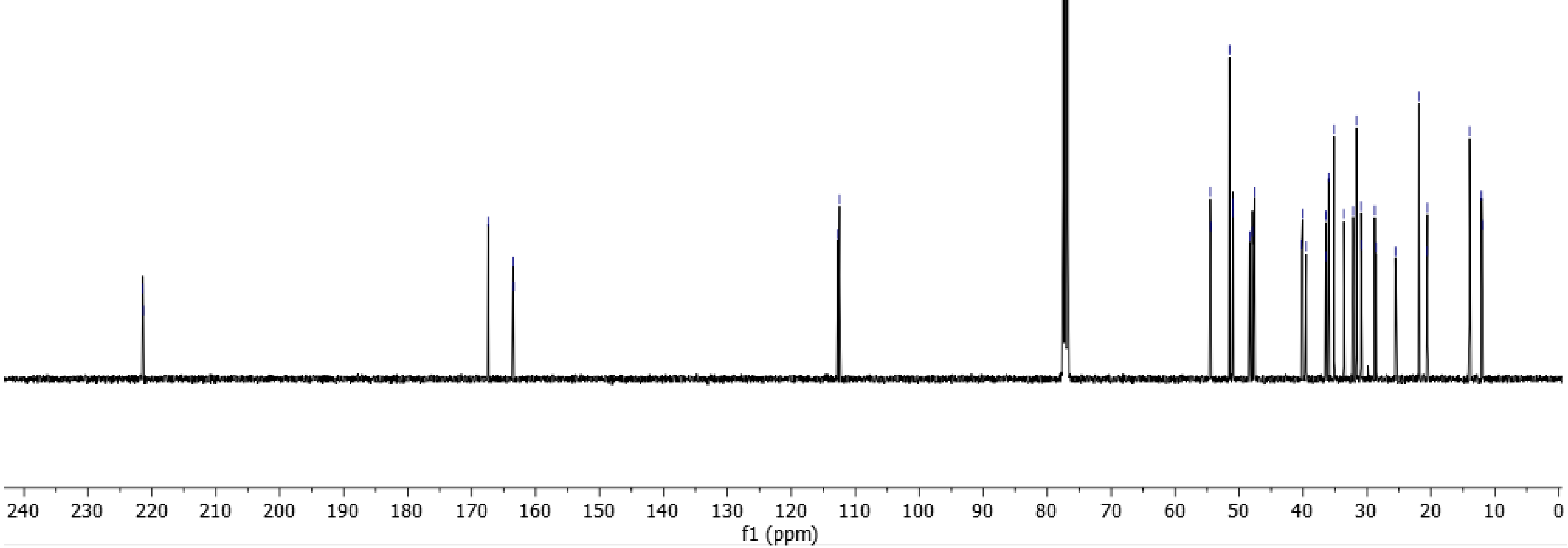

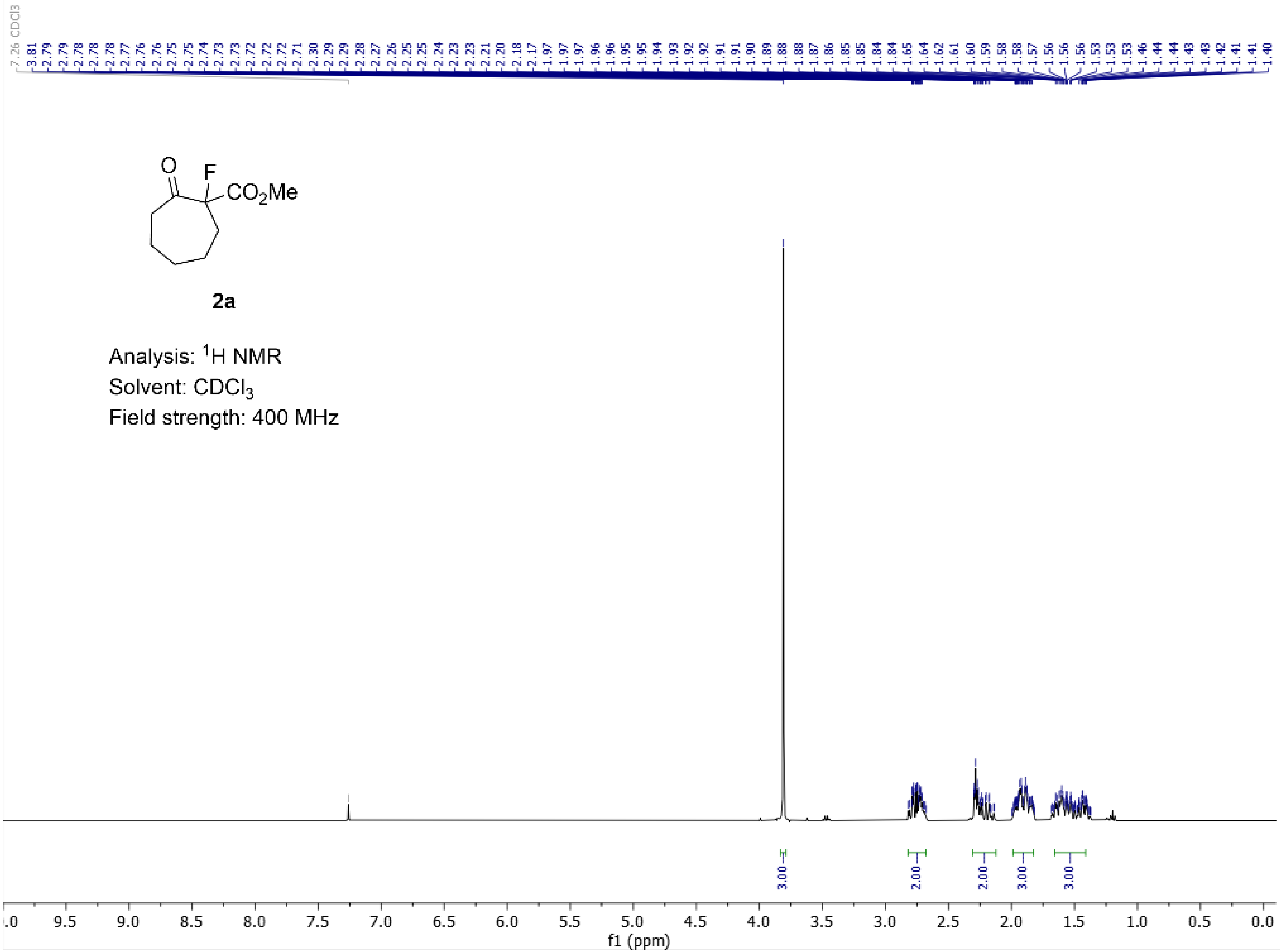
7.26 CDCl3
3.81
2.79 2.78 2.78 2.78 2.77 2.76 2.76 2.75 2.75 2.74 2.73 2.73 2.72 2.72 2.72 2.71
2.30 2.29 2.29 2.28 2.27 2.26 2.25 2.25 2.24 2.23 2.23 2.21 2.20 2.18 2.17
1.97 1.97 1.97 1.96 1.96 1.95 1.95 1.94 1.93 1.92 1.92 1.91 1.91 1.90 1.89 1.88 1.88 1.87 1.86 1.85 1.85 1.84 1.84
1.65 1.64 1.62 1.61 1.60 1.59 1.58 1.58 1.57 1.56 1.56 1.56 1.53 1.53 1.46 1.44 1.44 1.43 1.43 1.42 1.41 1.41 1.40
O
F
CO2Me
2a
Analysis: 1H NMR
Solvent: CDCl3
Field strength: 400 MHz
3.00
2.00
2.00
3.00
3.00
.0 9.5 9.0 8.5 8.0 7.5 7.0 6.5 6.0 5.5 5.0 4.5 4.0 3.5 3.0 2.5 2.0 1.5 1.0 0.5 0.0
f1 (ppm)

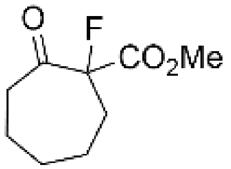

2a

Analysis: ^{19}F NMR
Solvent: $CDCl_3$
Field strength: 376 MHz

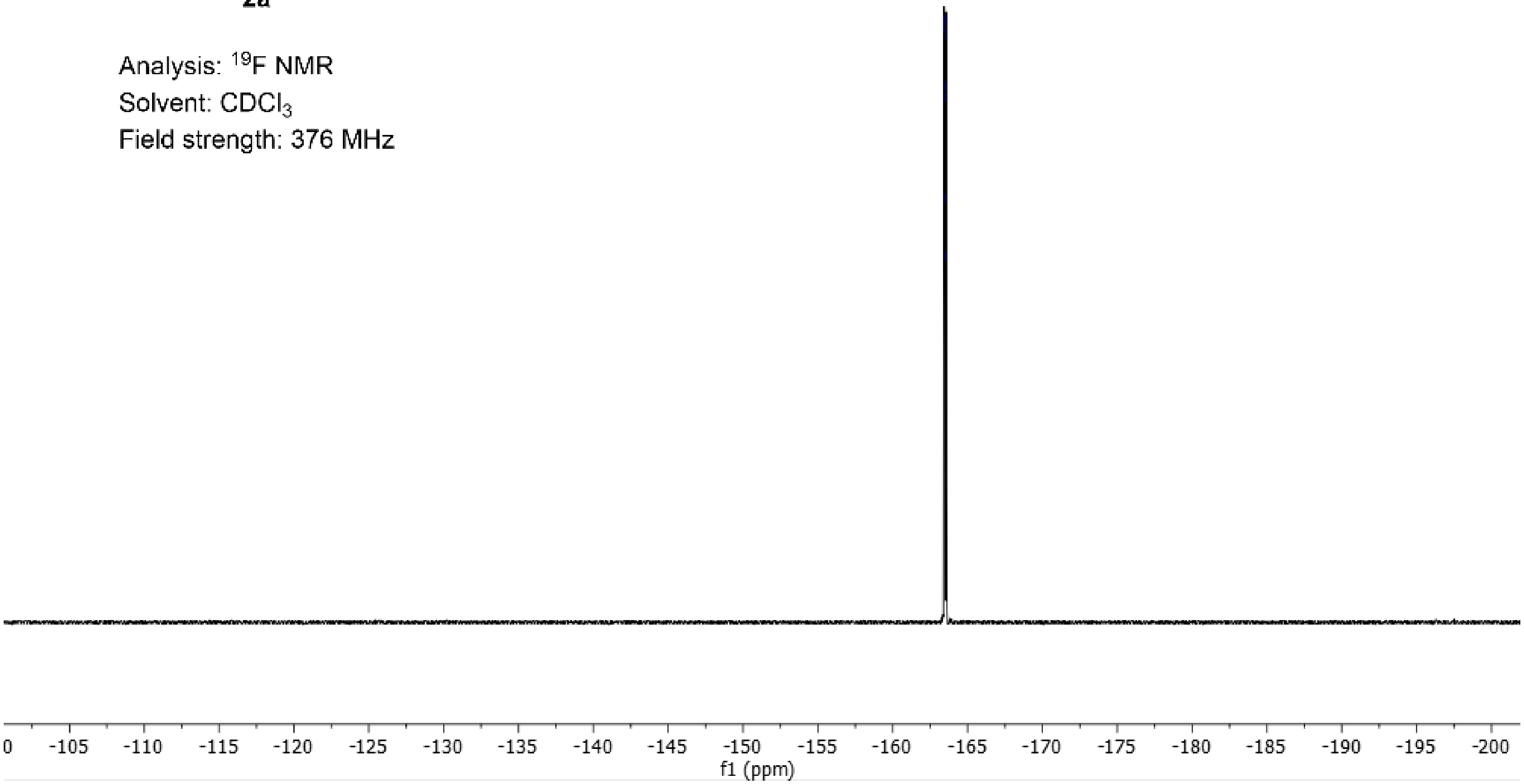

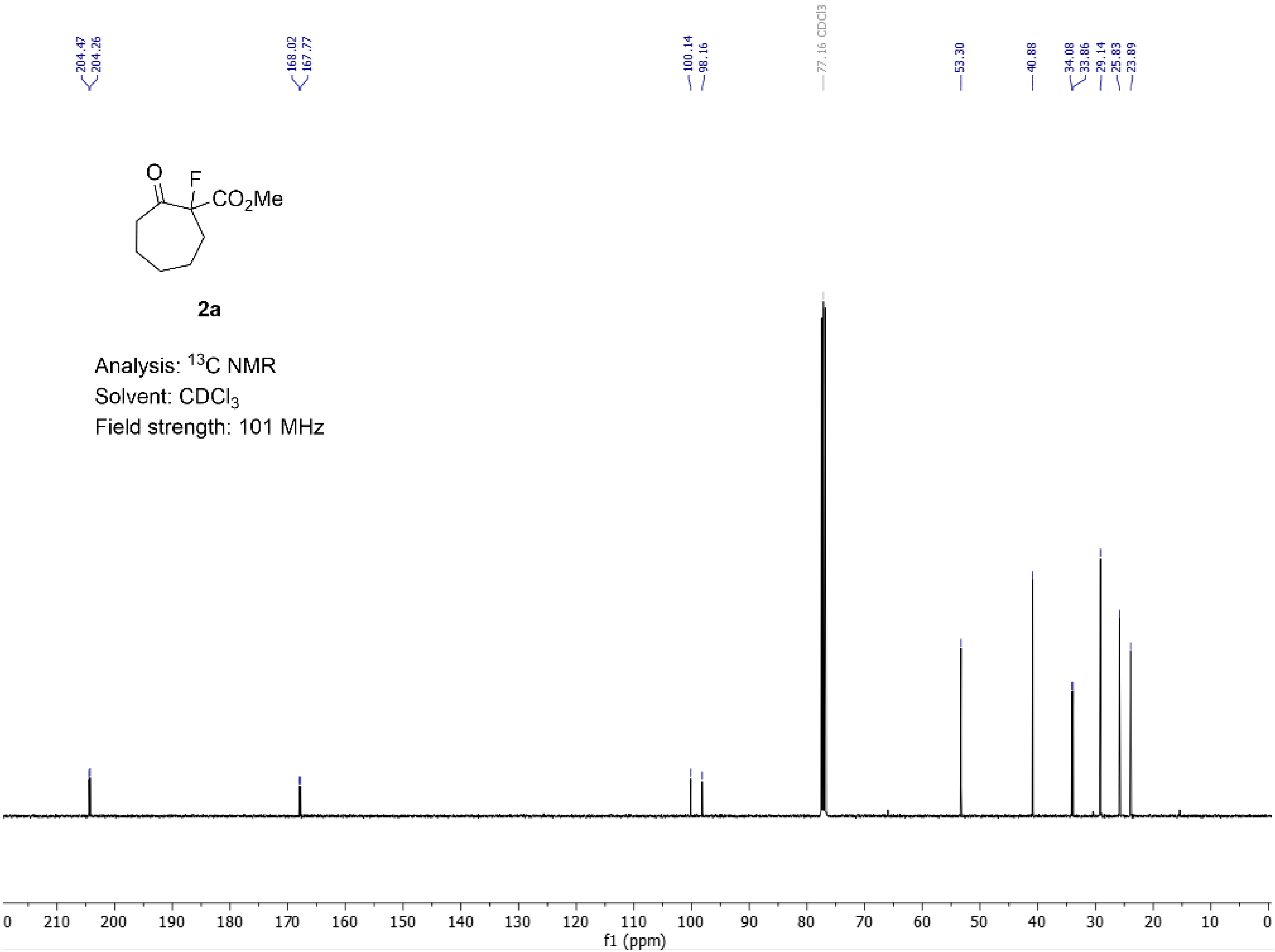
204.47
204.26
168.02
167.77
100.14
98.16
77.16 CDCl3
53.30
40.88
34.08
33.86
29.14
25.83
23.89
O
F
CO2Me
2a
Analysis: 13C NMR
Solvent: CDCl3
Field strength: 101 MHz
0 210 200 190 180 170 160 150 140 130 120 110 100 90 80 70 60 50 40 30 20 10 0
f1 (ppm)

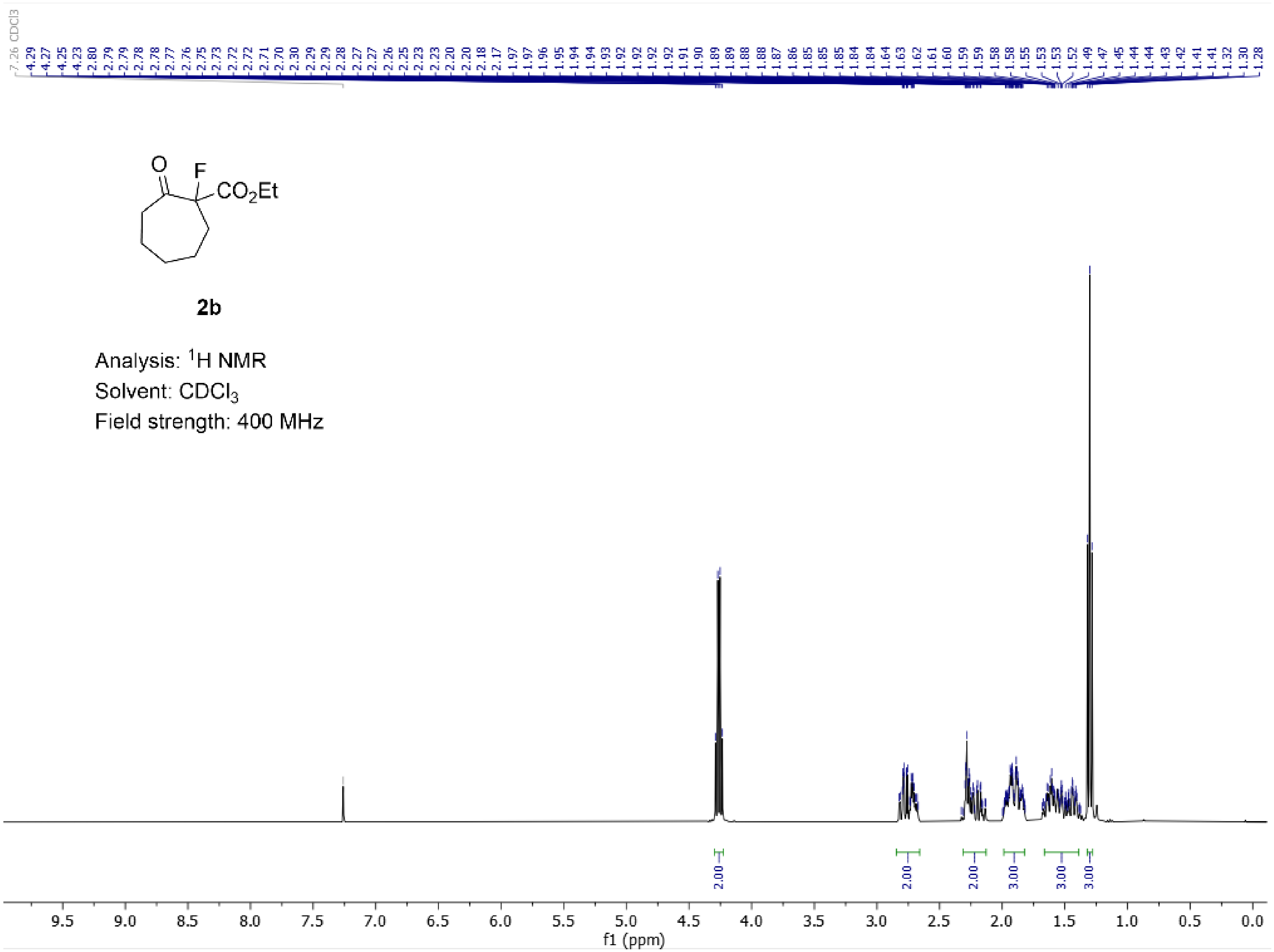
7.26 CDCl3
4.29 4.27 4.25 4.23 2.80 2.79 2.79 2.78 2.78 2.77 2.76 2.75 2.73 2.72 2.72 2.71 2.70 2.30 2.29 2.29 2.28 2.27 2.27 2.27 2.26 2.25 2.23 2.23 2.20 2.20 2.18 2.17 1.97 1.97 1.96 1.95 1.94 1.94 1.93 1.92 1.92 1.92 1.92 1.91 1.90 1.89 1.89 1.88 1.88 1.87 1.86 1.85 1.85 1.85 1.84 1.84 1.64 1.63 1.62 1.61 1.60 1.59 1.59 1.58 1.58 1.55 1.53 1.53 1.52 1.49 1.47 1.45 1.44 1.44 1.43 1.42 1.41 1.41 1.32 1.30 1.28
O
F
CO2Et
2b
Analysis: 1H NMR
Solvent: CDCl3
Field strength: 400 MHz
2.00
2.00
2.00
3.00
3.00
3.00
9.5 9.0 8.5 8.0 7.5 7.0 6.5 6.0 5.5 5.0 4.5 4.0 3.5 3.0 2.5 2.0 1.5 1.0 0.5 0.0
f1 (ppm)

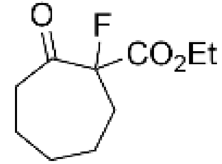

2b

Analysis: ^{19}F NMR
Solvent: $CDCl_3$
Field strength: 377 MHz

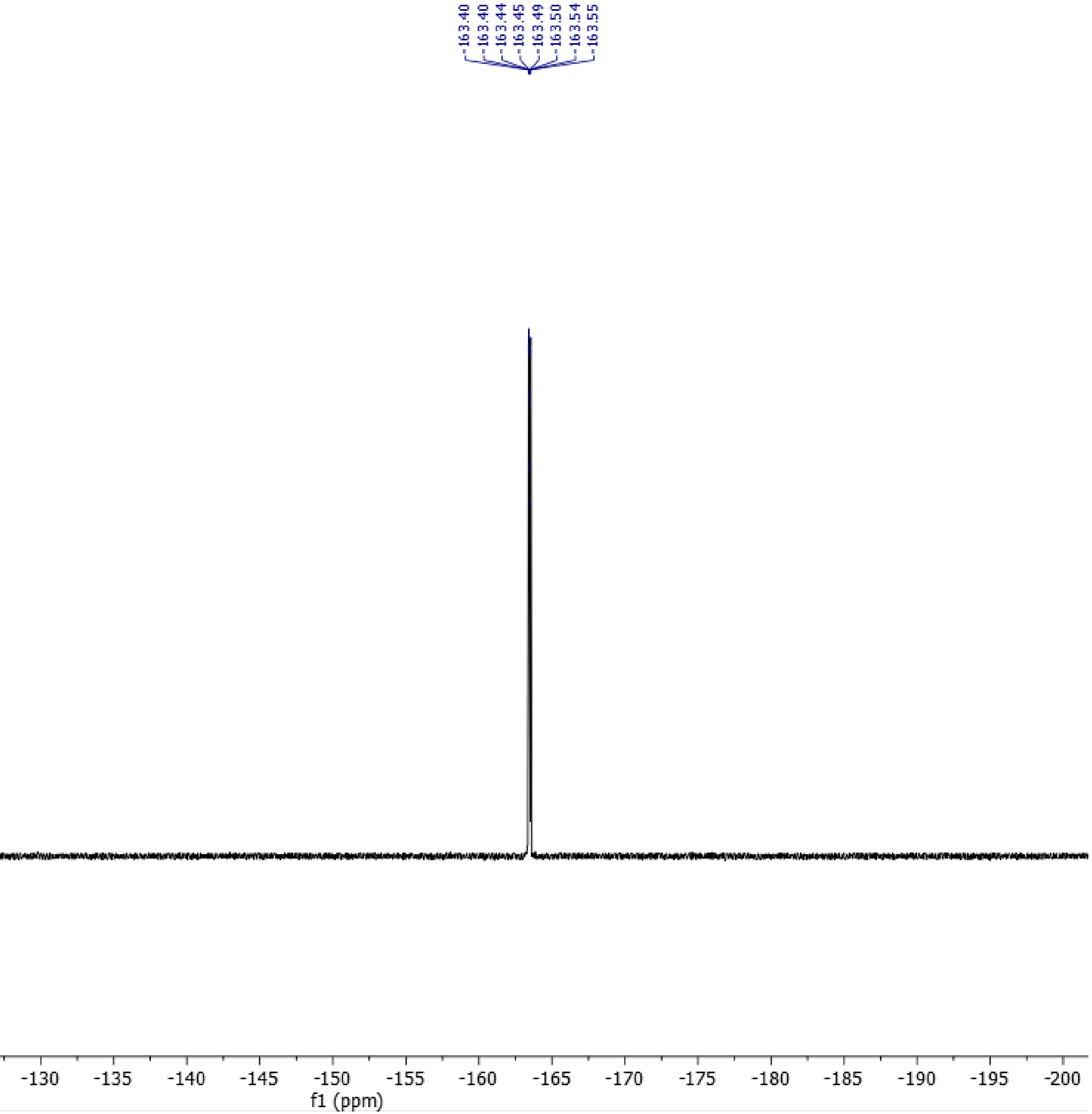

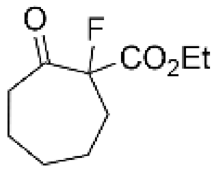

2b

Analysis: ^{13}C NMR
Solvent: $CDCl_3$
Field strength: 101 MHz

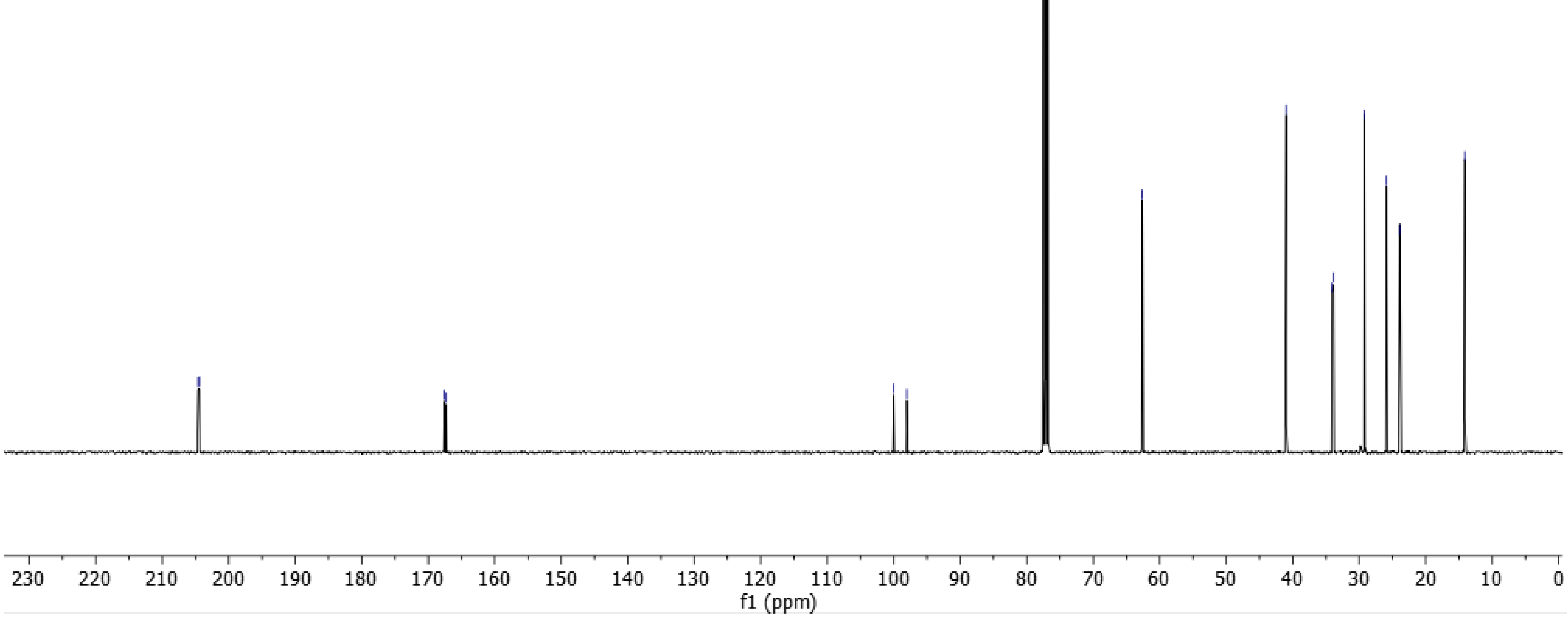

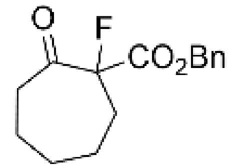

2c

Analysis: ^{1}H NMR
Solvent: $CDCl_3$
Field strength: 400 MHz

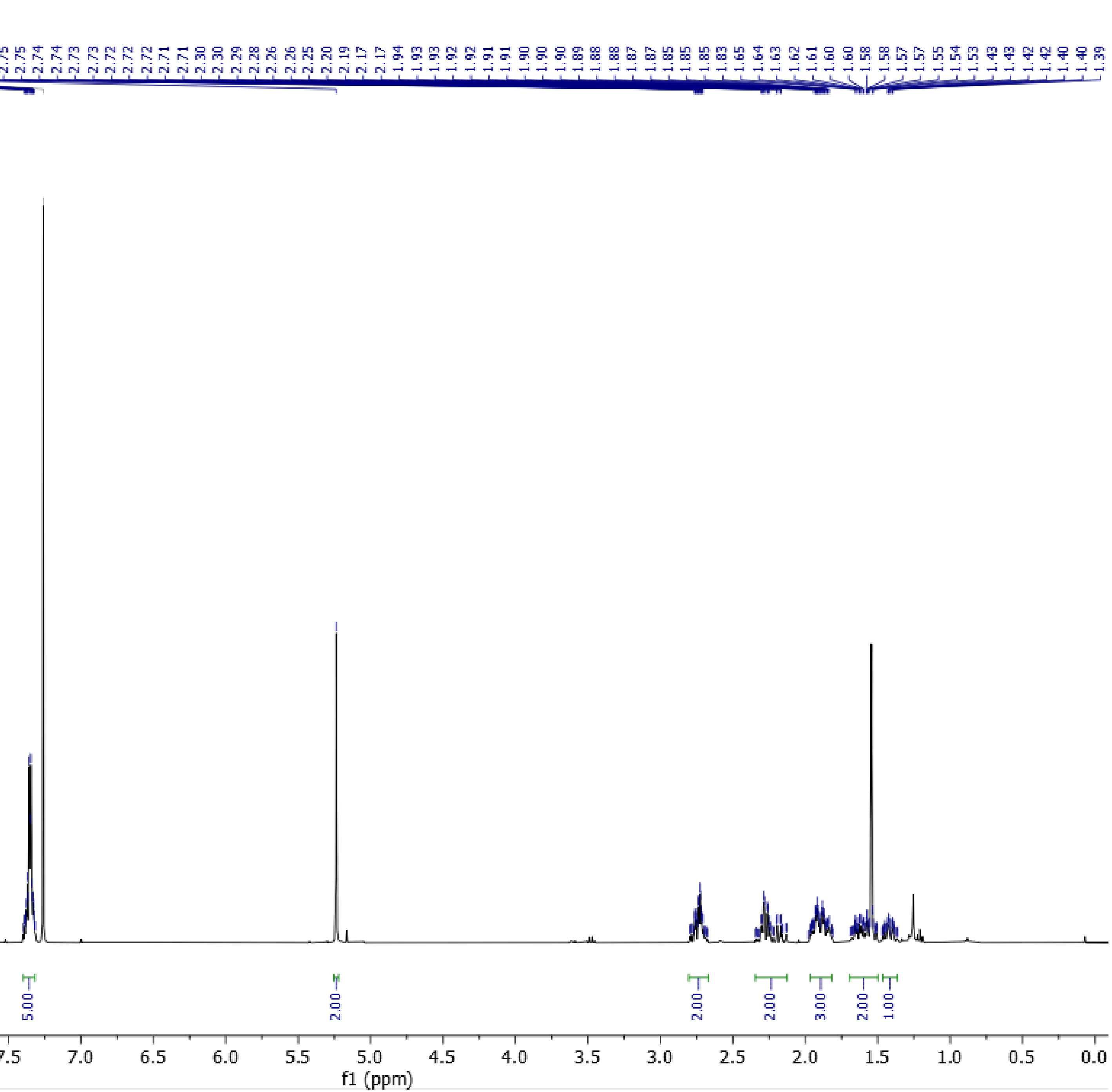

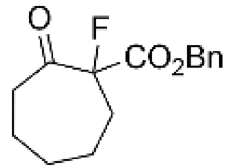

2c

Analysis: ^{19}F NMR
Solvent: $CDCl_3$
Field strength: 377 MHz

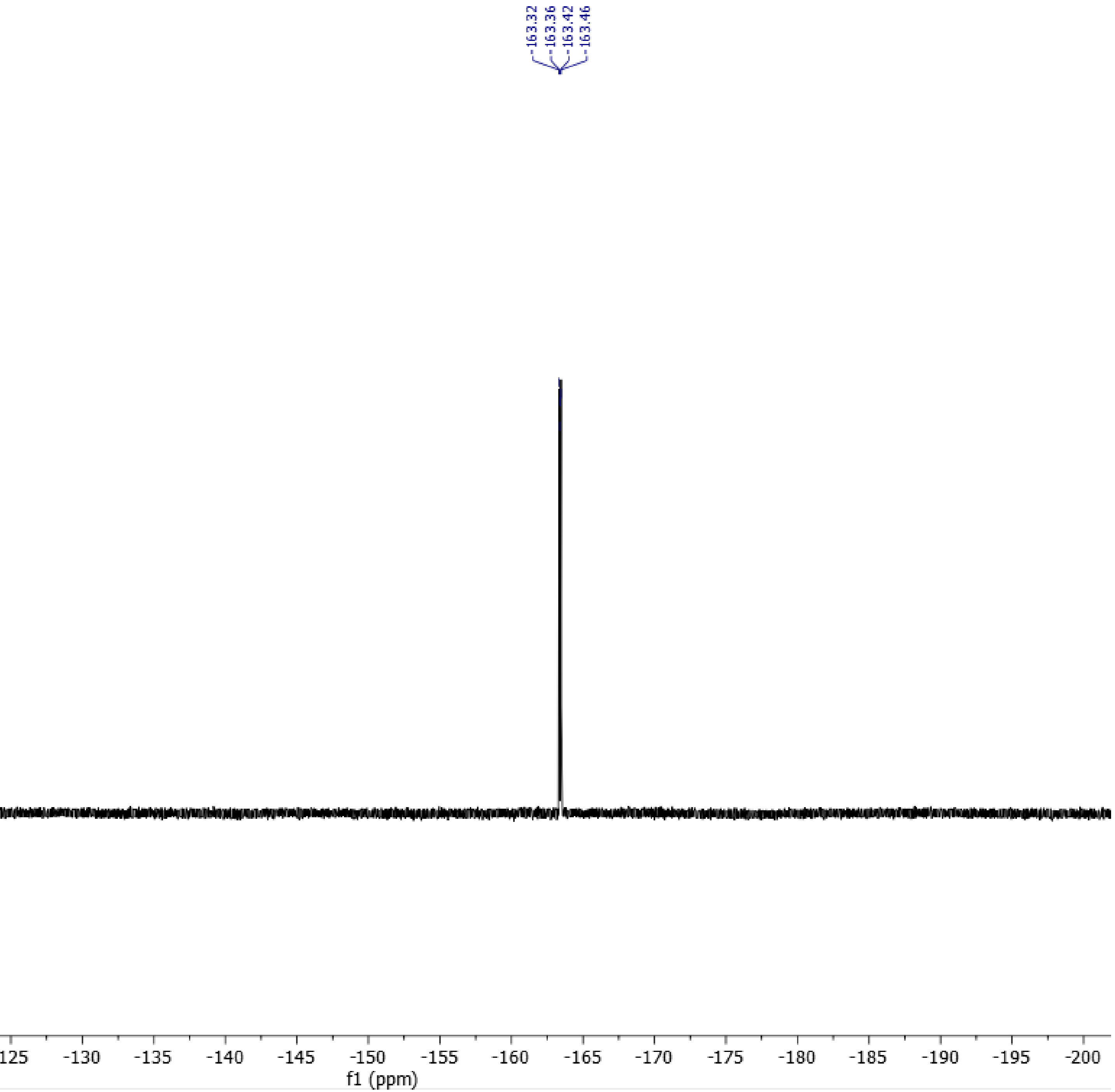

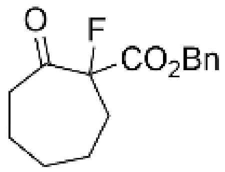

2c

Analysis: ^{13}C NMR
Solvent: $CDCl_3$
Field strength: 101 MHz

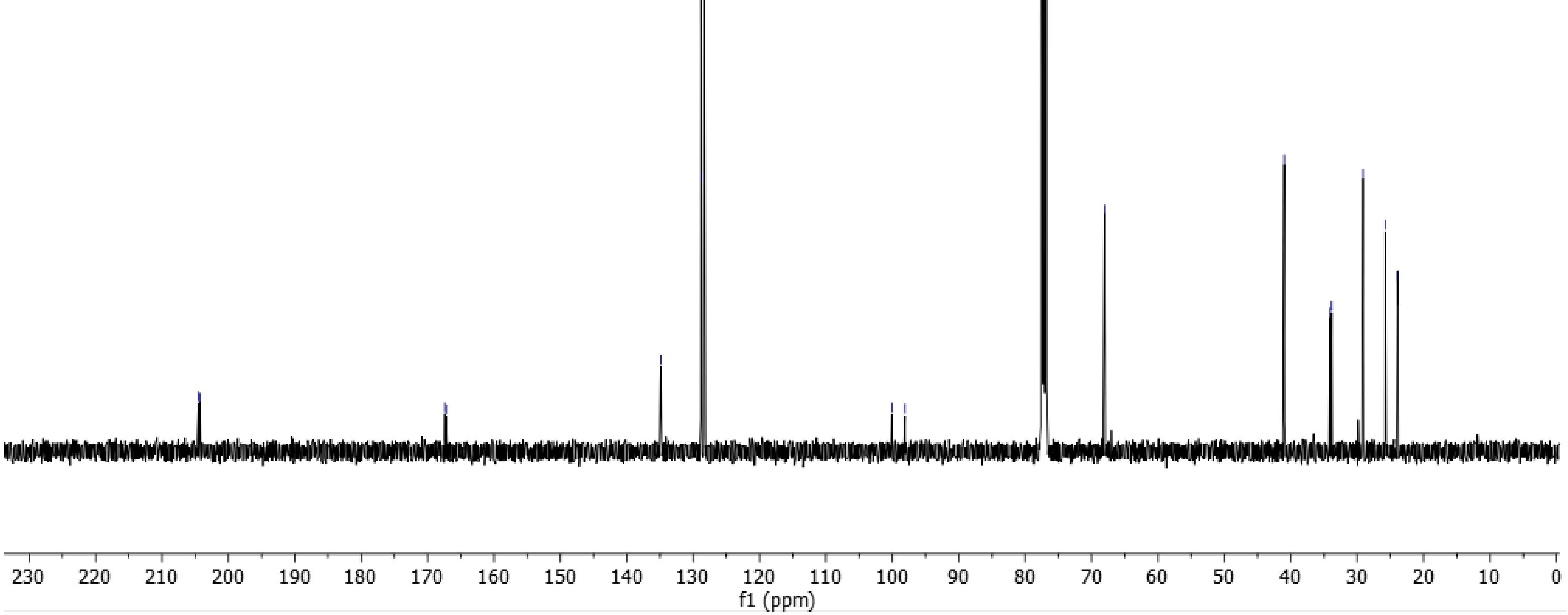

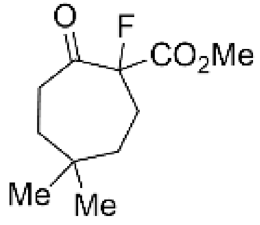

2d

Analysis: ^{1}H NMR
Solvent: $CDCl_3$
Field strength: 400 MHz

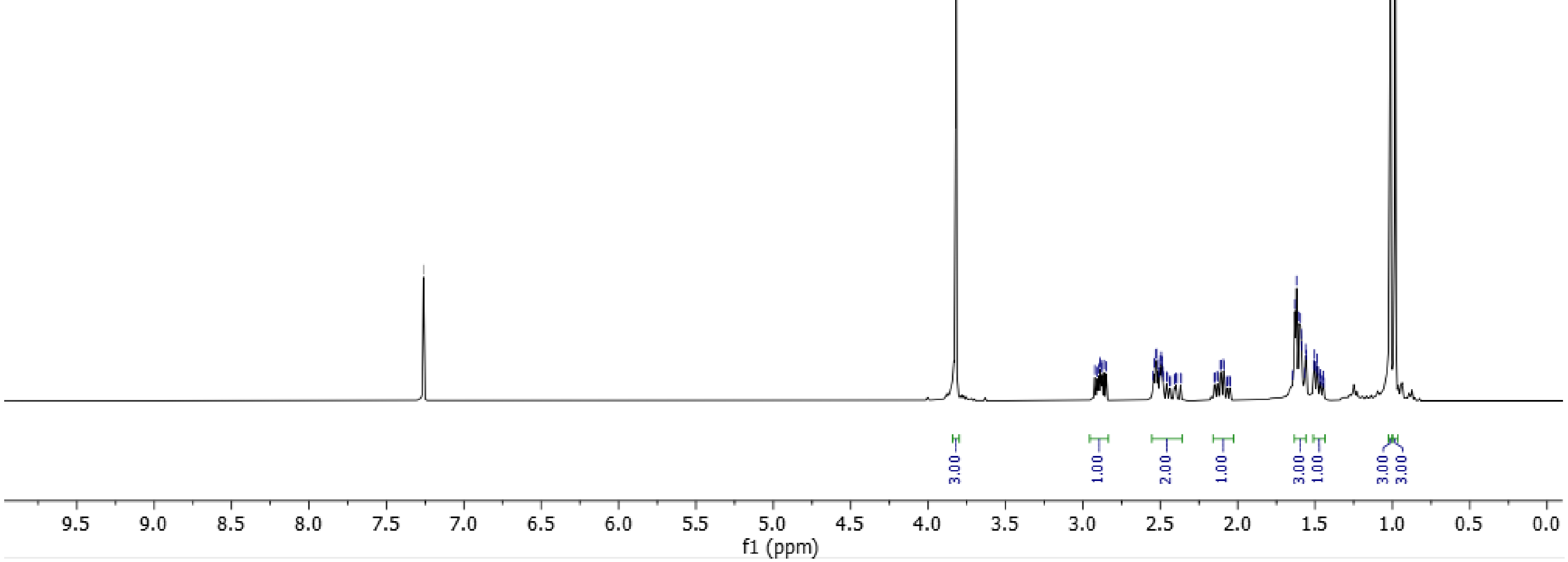

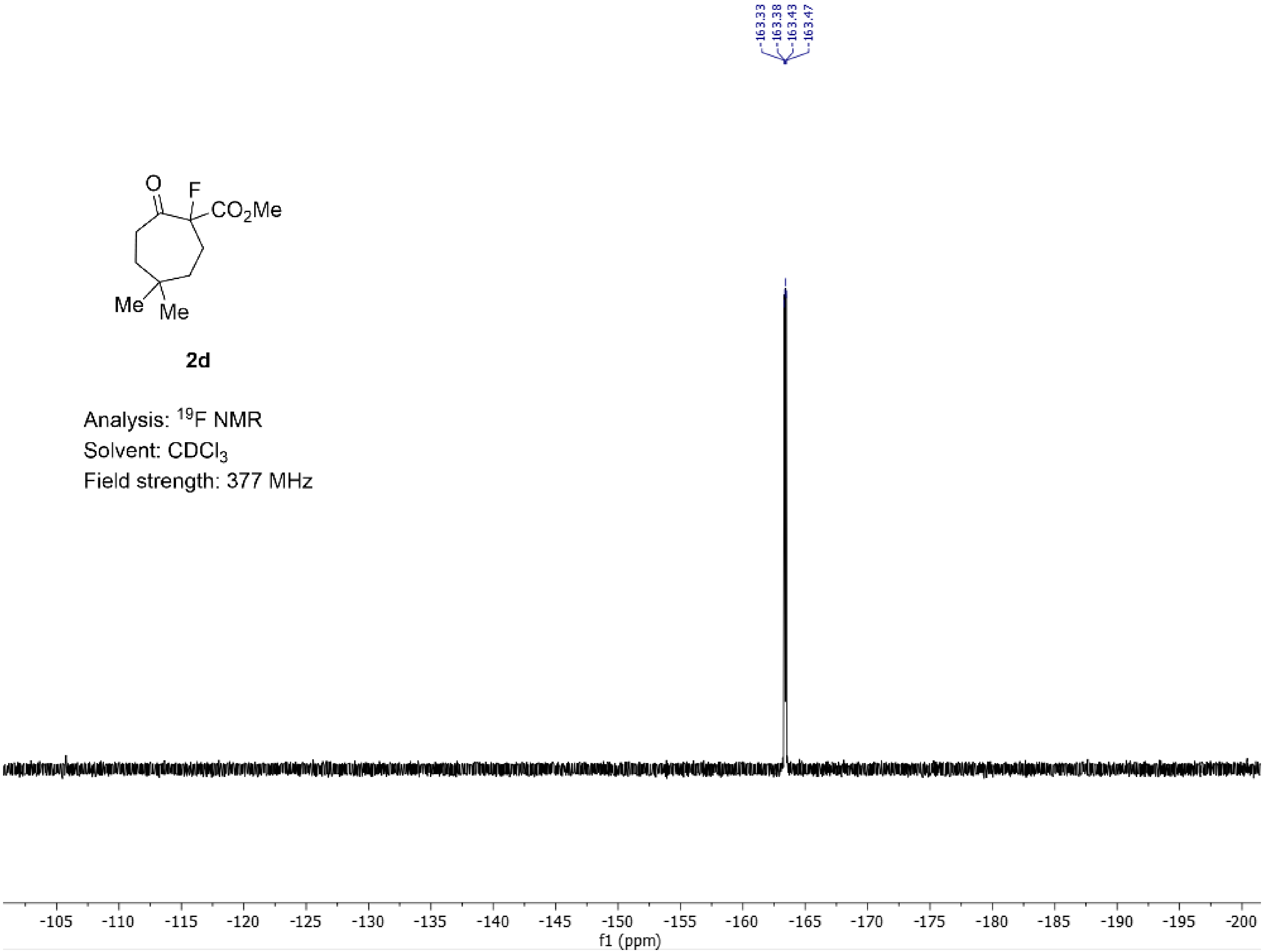

2d

Analysis: ^{19}F NMR
Solvent: $CDCl_3$
Field strength: 377 MHz

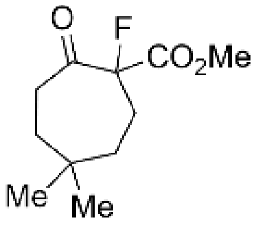

2d

Analysis: ^{13}C NMR
Solvent: $CDCl_3$
Field strength: 101 MHz

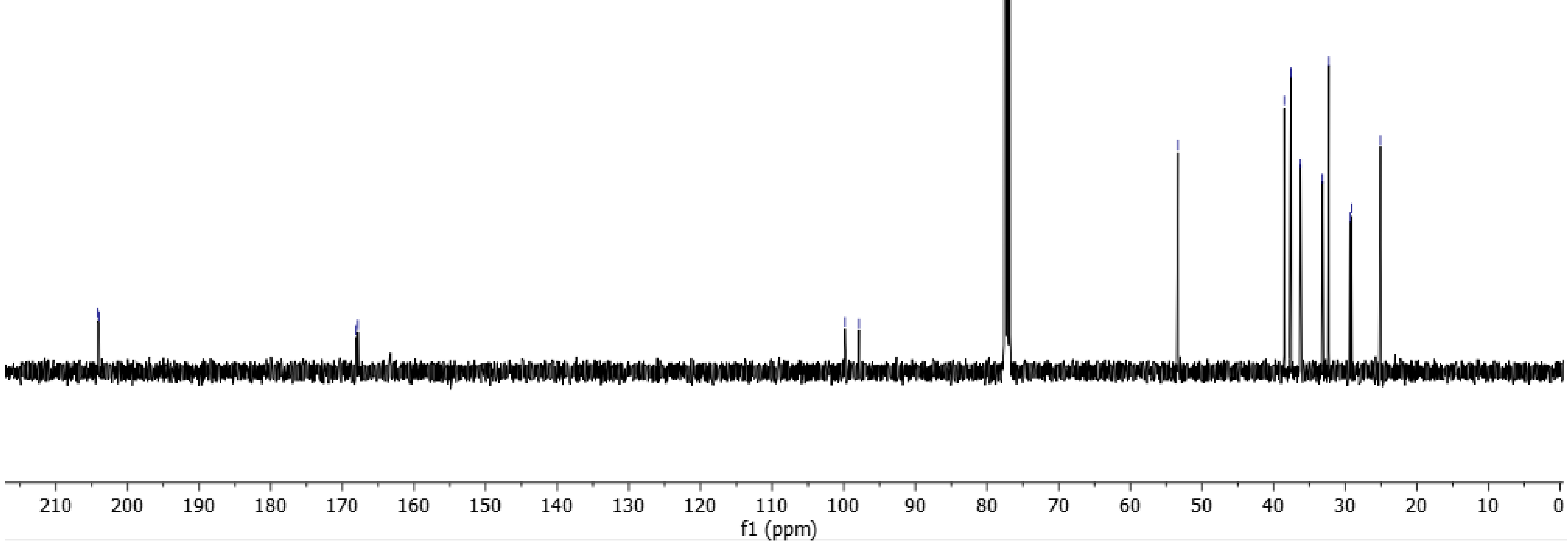

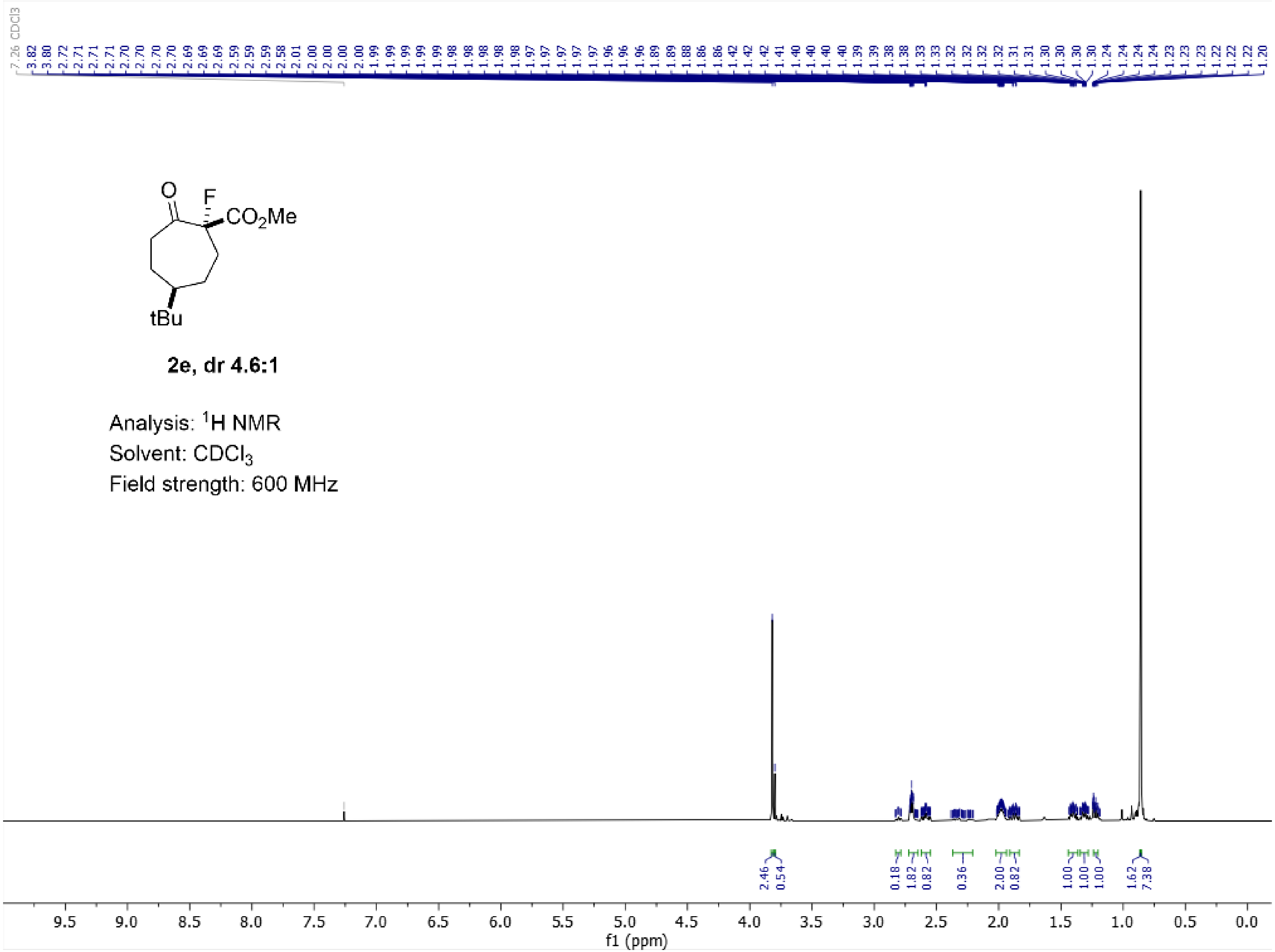
7.26 CDCl3
3.82 3.80 2.72 2.71 2.71 2.71 2.70 2.70 2.70 2.69 2.69 2.69 2.59 2.59 2.59 2.58 2.01 2.00 2.00 2.00 2.00 1.99 1.99 1.99 1.99 1.98 1.98 1.98 1.98 1.98 1.97 1.97 1.97 1.97 1.96 1.96 1.96 1.89 1.89 1.88 1.86 1.86 1.42 1.42 1.42 1.41 1.40 1.40 1.40 1.40 1.39 1.39 1.38 1.38 1.33 1.33 1.32 1.32 1.32 1.32 1.31 1.31 1.30 1.30 1.30 1.30 1.24 1.24 1.24 1.24 1.23 1.23 1.23 1.22 1.22 1.22 1.20
O
F
CO_2Me
tBu
2e, dr 4.6:1
Analysis: ^{1}H NMR
Solvent: $CDCl_3$
Field strength: 600 MHz
2.46 0.54 0.18 1.82 0.82 0.36 2.00 0.82 1.00 1.00 1.00 1.62 7.38
9.5 9.0 8.5 8.0 7.5 7.0 6.5 6.0 5.5 5.0 4.5 4.0 3.5 3.0 2.5 2.0 1.5 1.0 0.5 0.0
f1 (ppm)

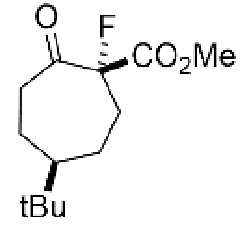

2e, dr 4.6:1

Analysis: ^{19}F NMR
Solvent: $CDCl_3$
Field strength: 377 MHz

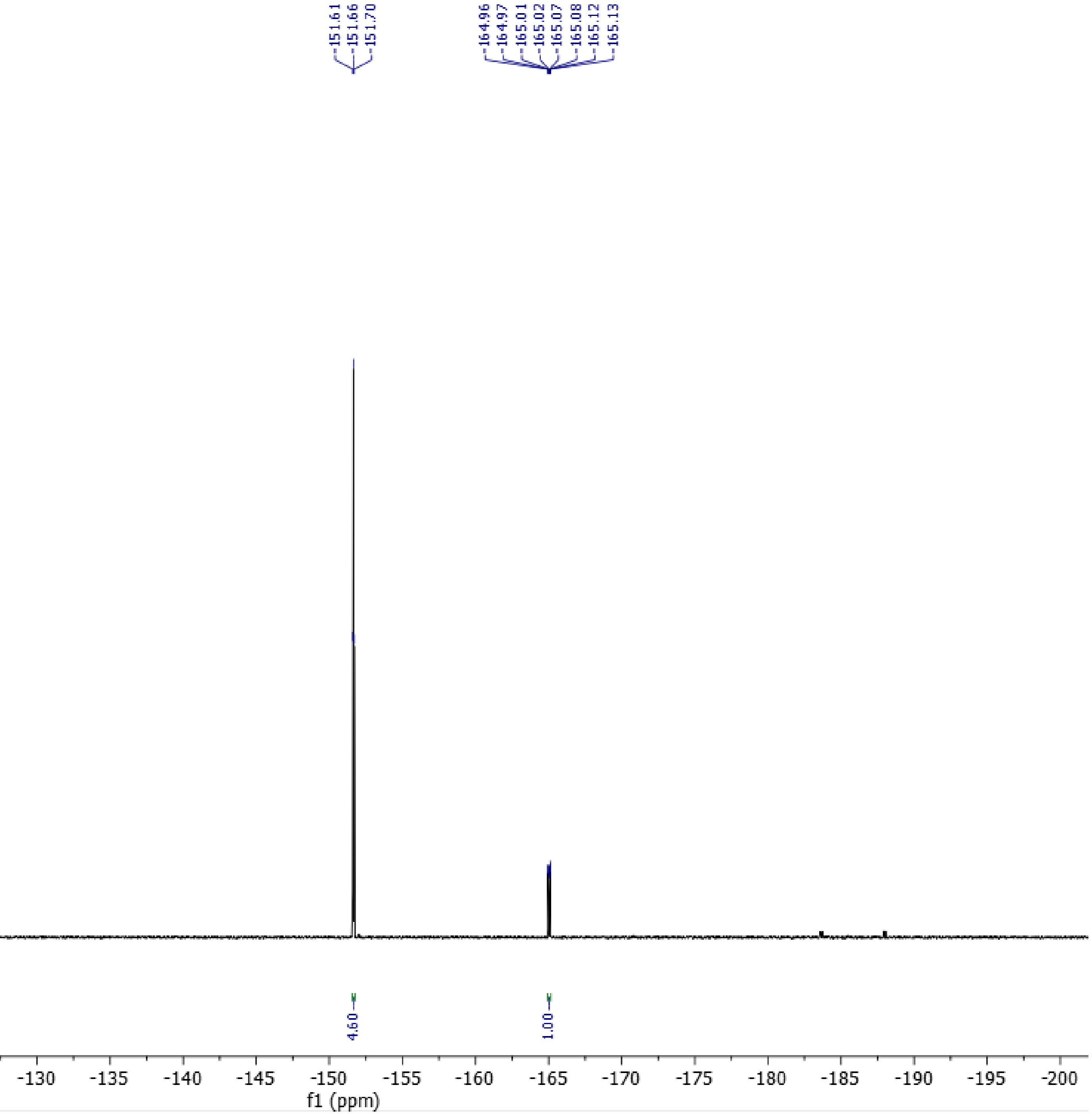

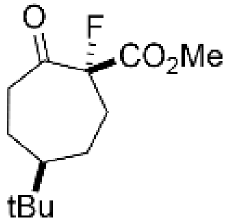

2e, dr 4.6:1

Analysis: ^{13}C NMR
Solvent: $CDCl_3$
Field strength: 151 MHz

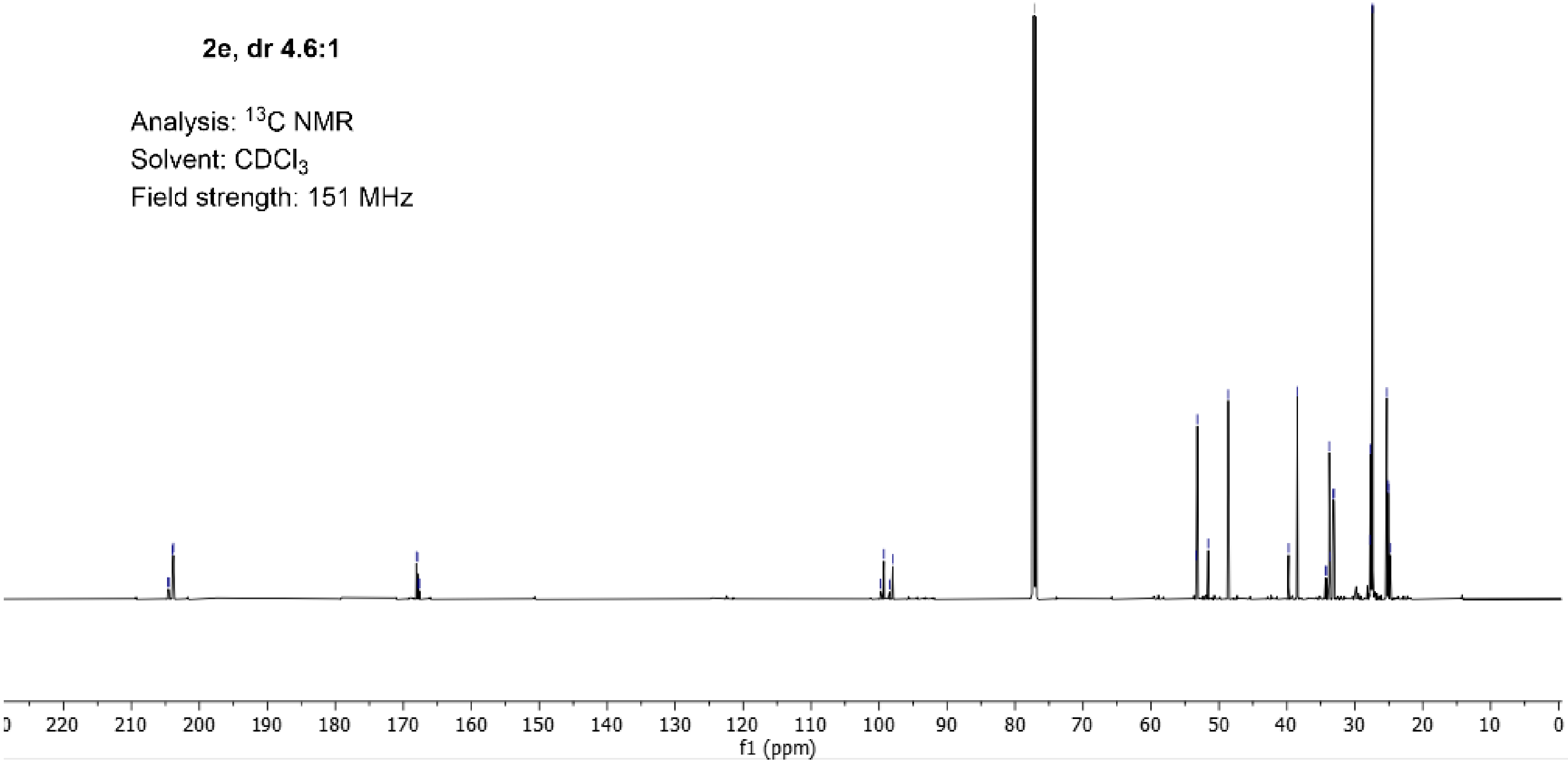

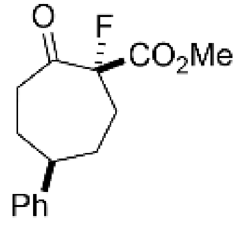

2f-1

Analysis: ^{1}H NMR
Solvent: $CDCl_3$
Field strength: 400 MHz

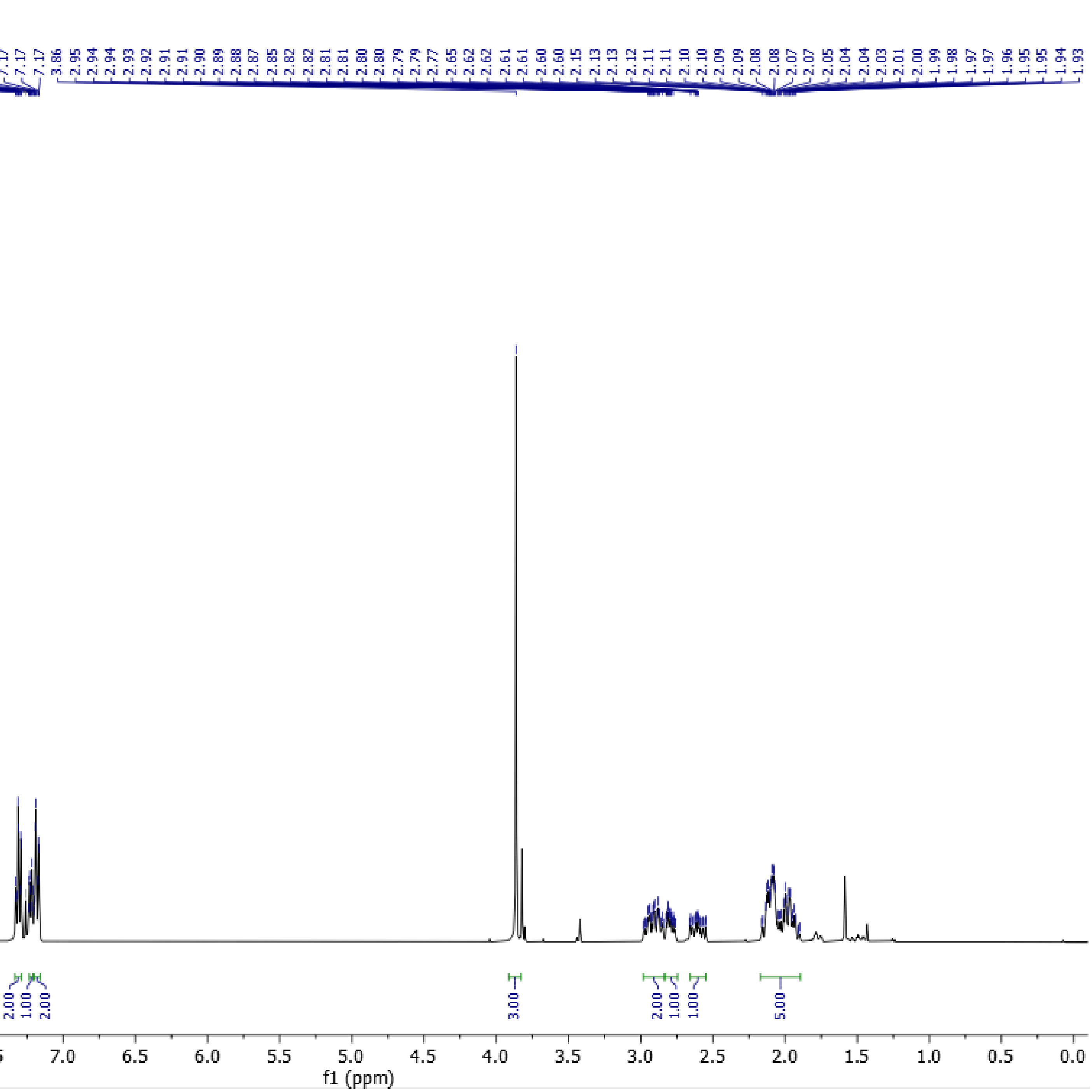

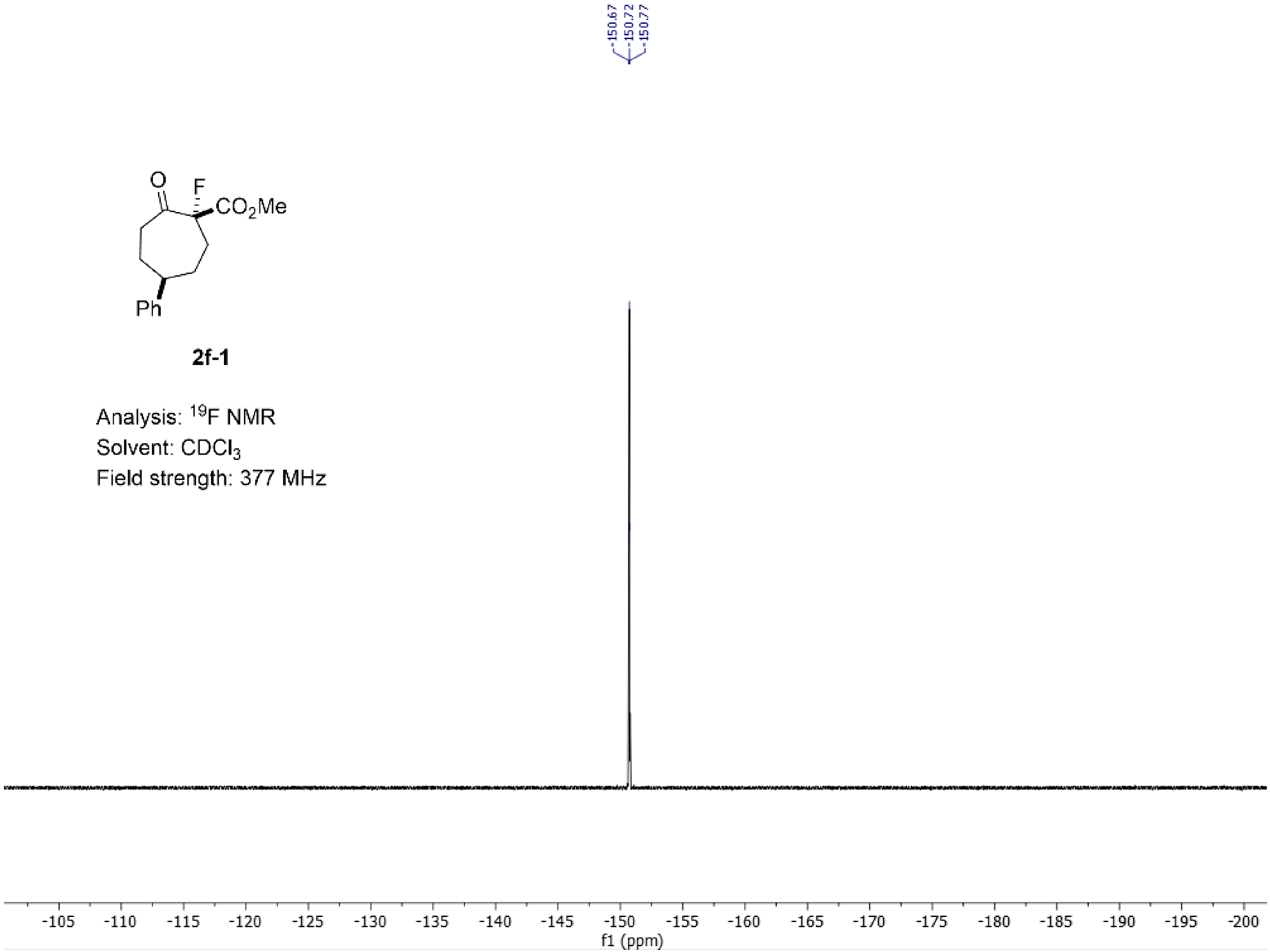

2f-1

Analysis: ^{19}F NMR
Solvent: $CDCl_3$
Field strength: 377 MHz

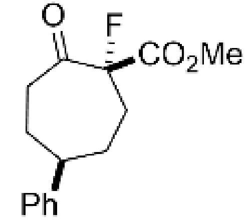

2f-1

Analysis: ^{13}C NMR
Solvent: $CDCl_3$
Field strength: 101 MHz

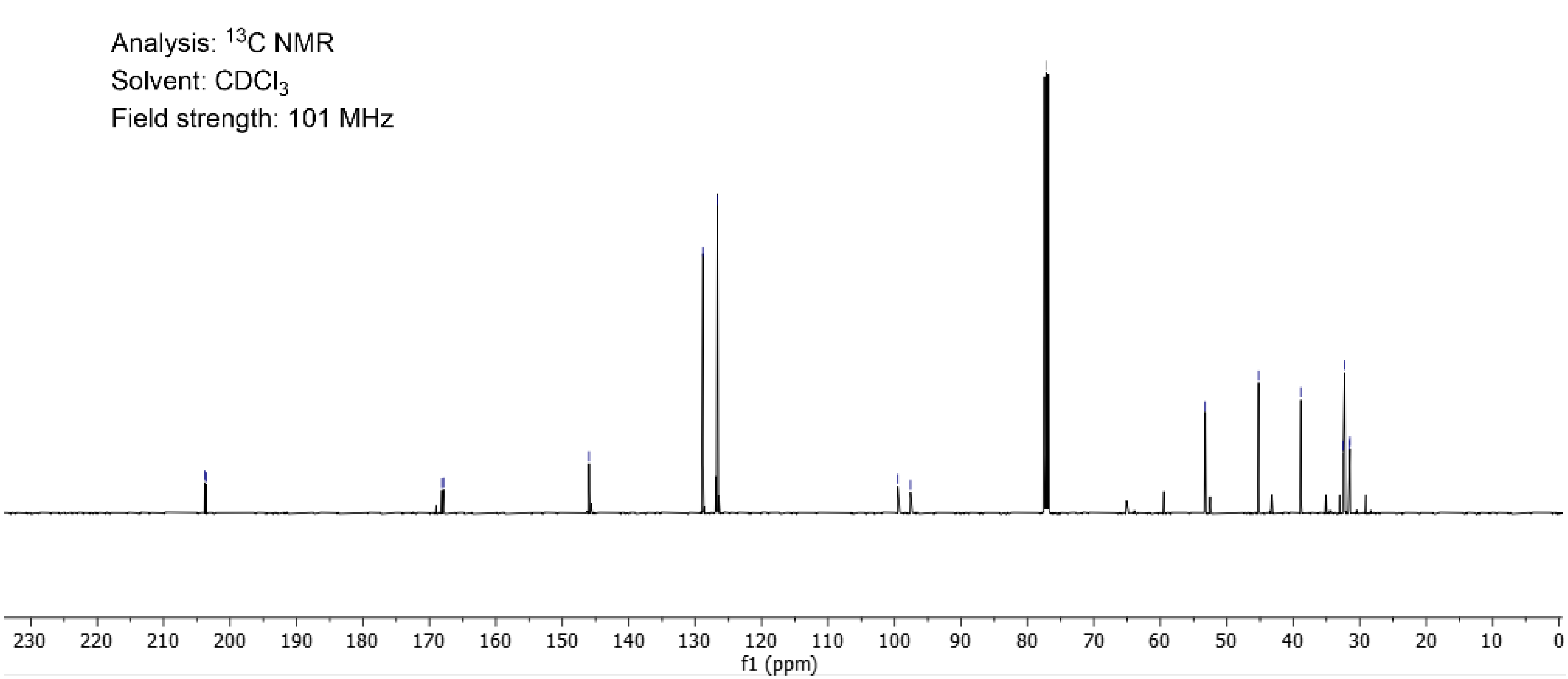

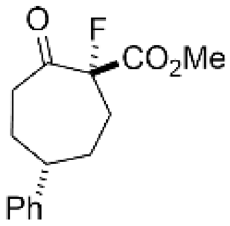

2f-2

Analysis: ^{1}H NMR
Solvent: $CDCl_3$
Field strength: 400 MHz

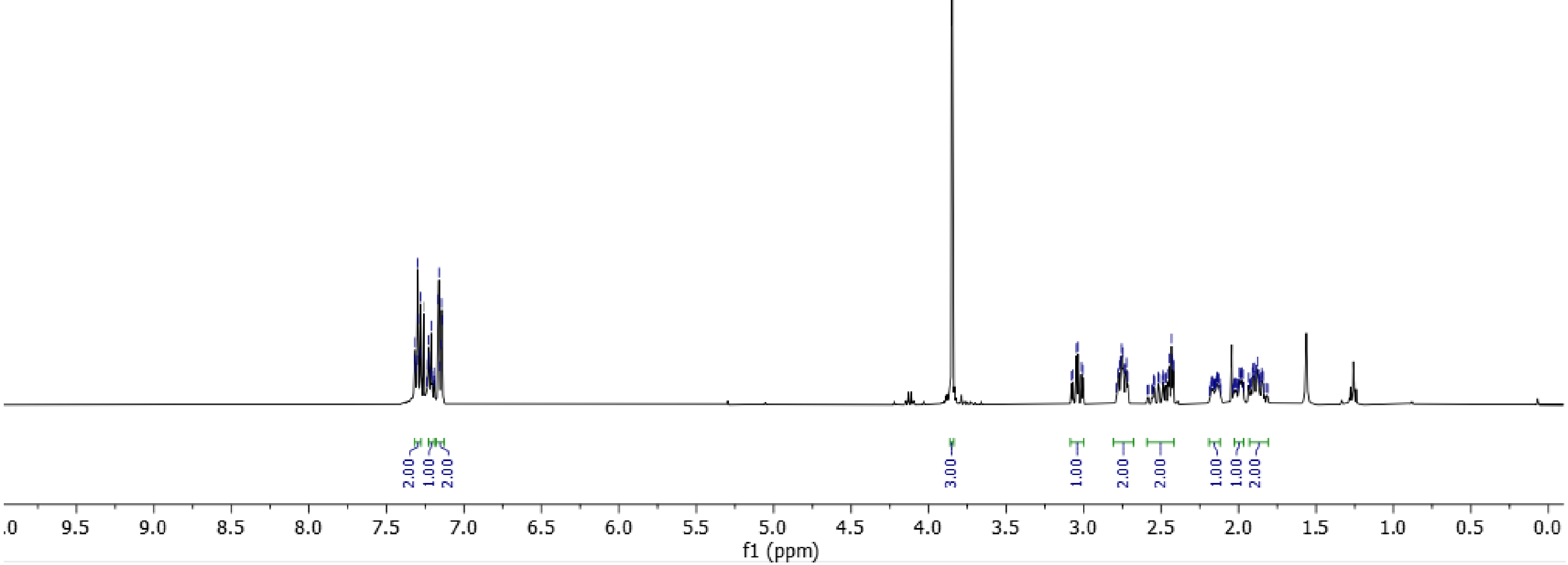

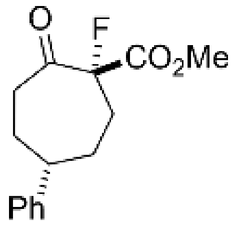

2f-2

Analysis: ^{19}F NMR
Solvent: $CDCl_3$
Field strength: 377 MHz

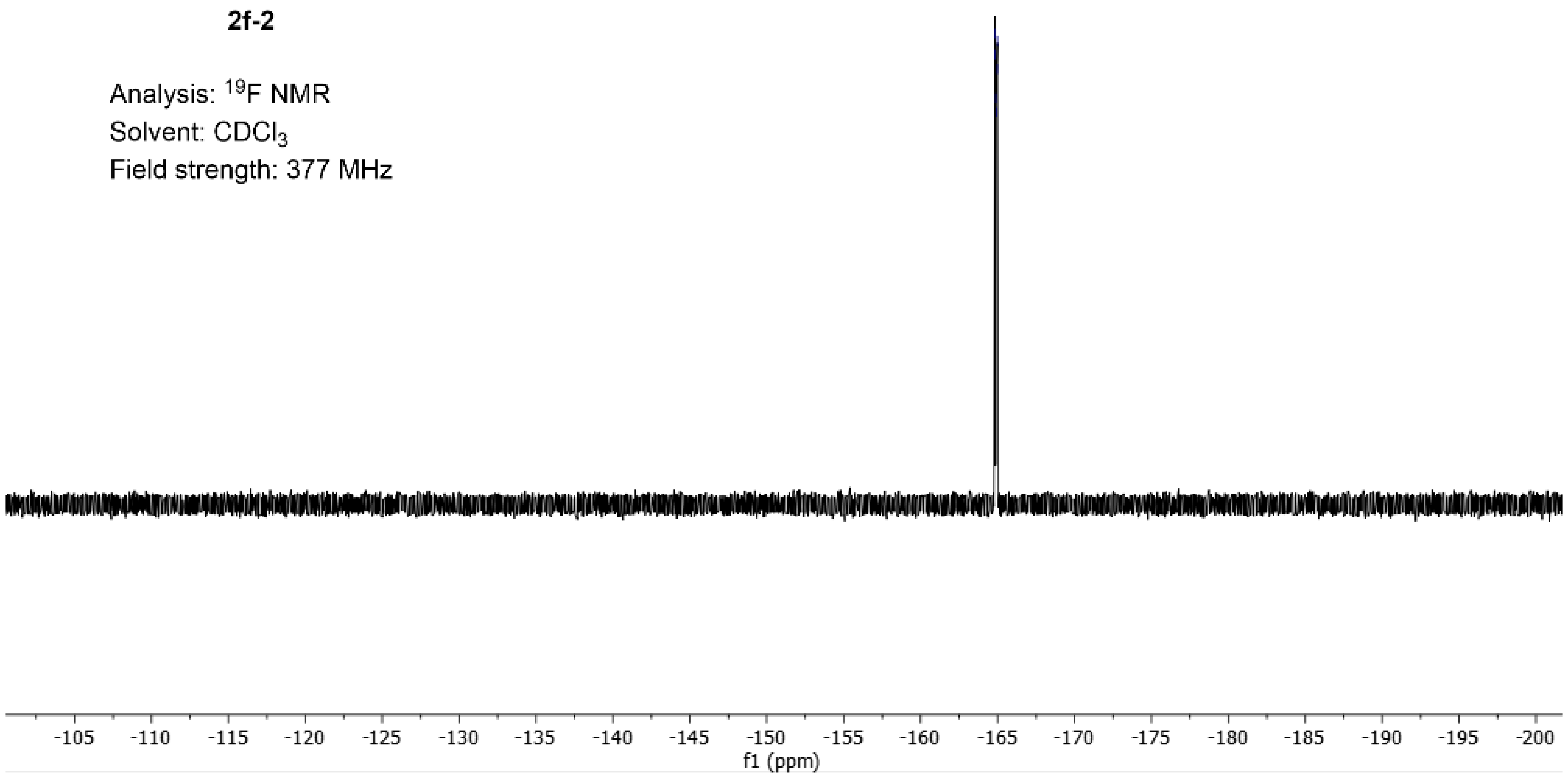

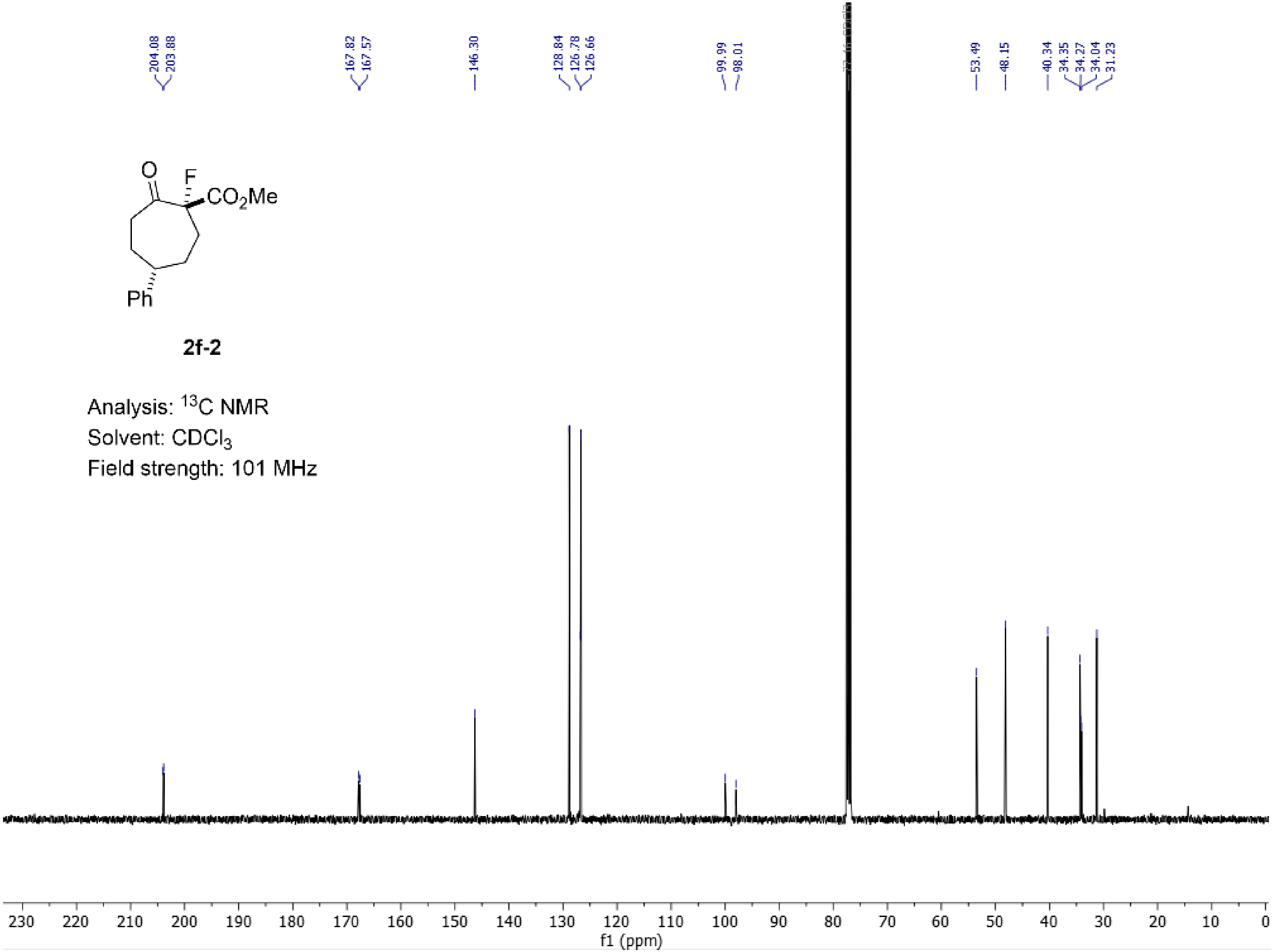
204.08
203.88
167.82
167.57
146.30
128.84
126.78
126.66
99.99
98.01
53.49
48.15
40.34
34.35
34.27
34.04
31.23
O
F
CO_2Me
Ph
2f-2
Analysis: ^{13}C NMR
Solvent: $CDCl_3$
Field strength: 101 MHz
230 220 210 200 190 180 170 160 150 140 130 120 110 100 90 80 70 60 50 40 30 20 10 0
f1 (ppm)

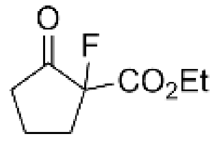

2g

Analysis: ^{1}H NMR
Solvent: $CDCl_3$
Field strength: 400 MHz

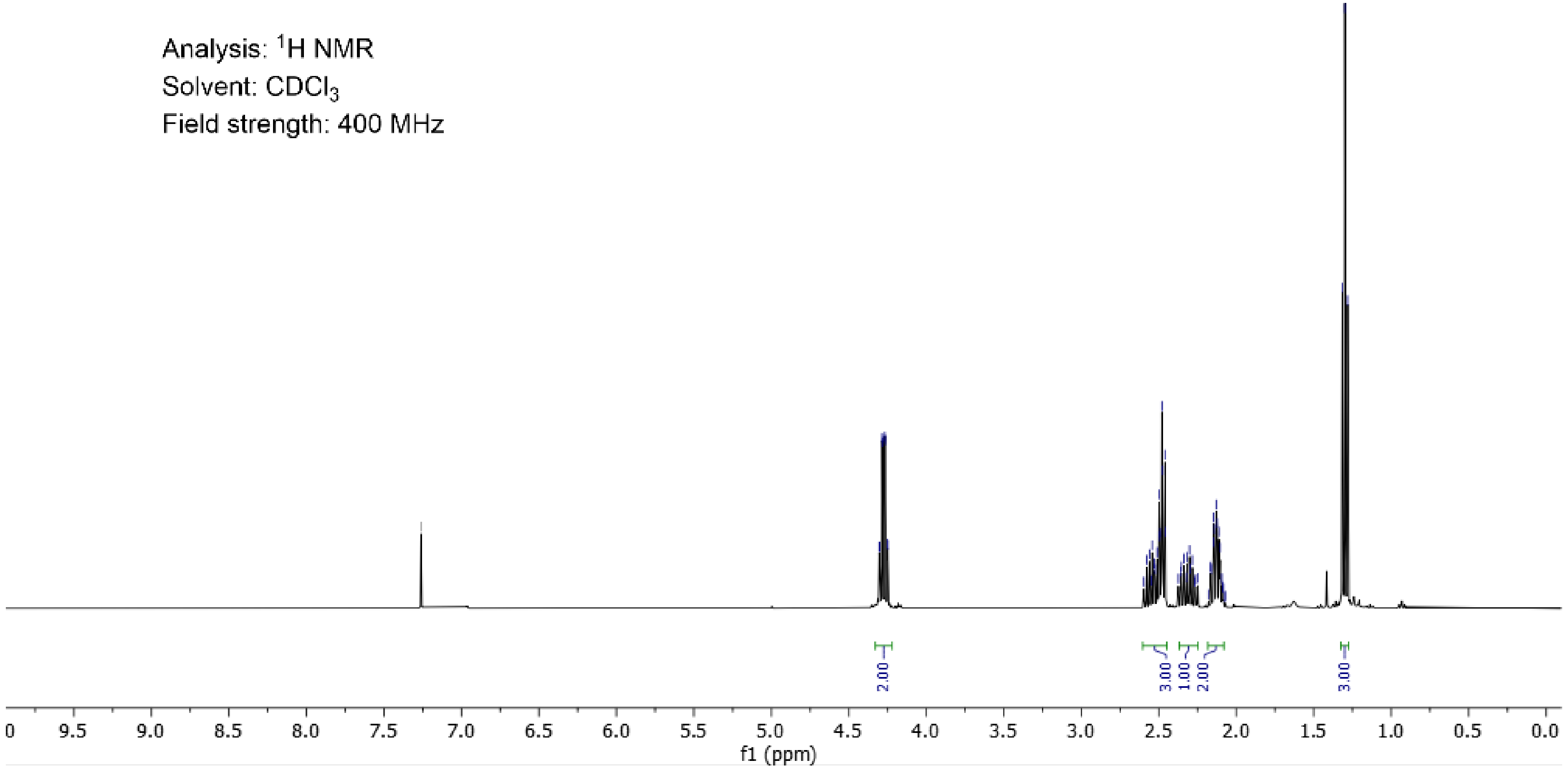

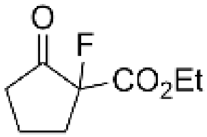

2g

Analysis: ^{19}F NMR
Solvent: $CDCl_3$
Field strength: 376 MHz

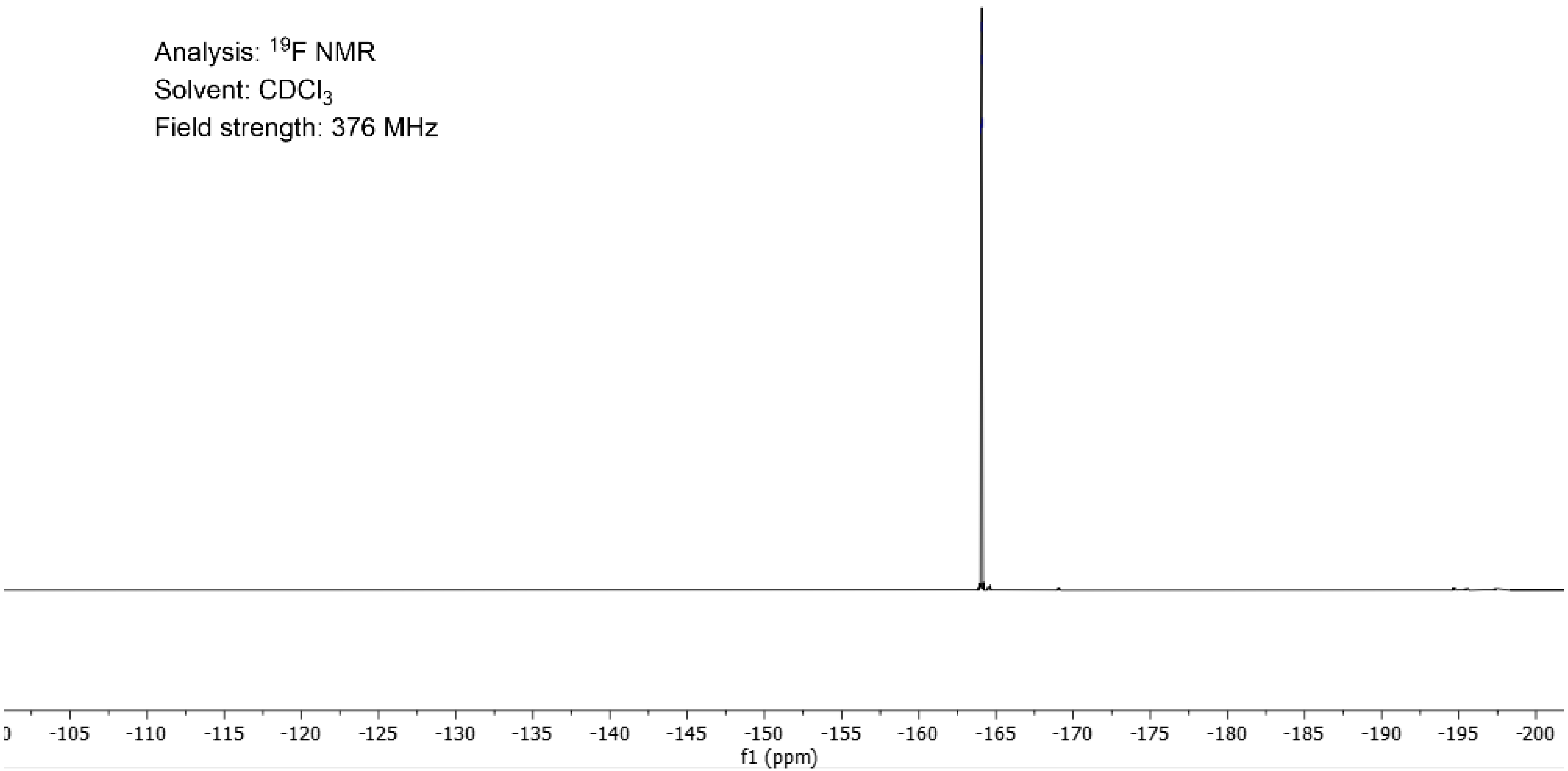

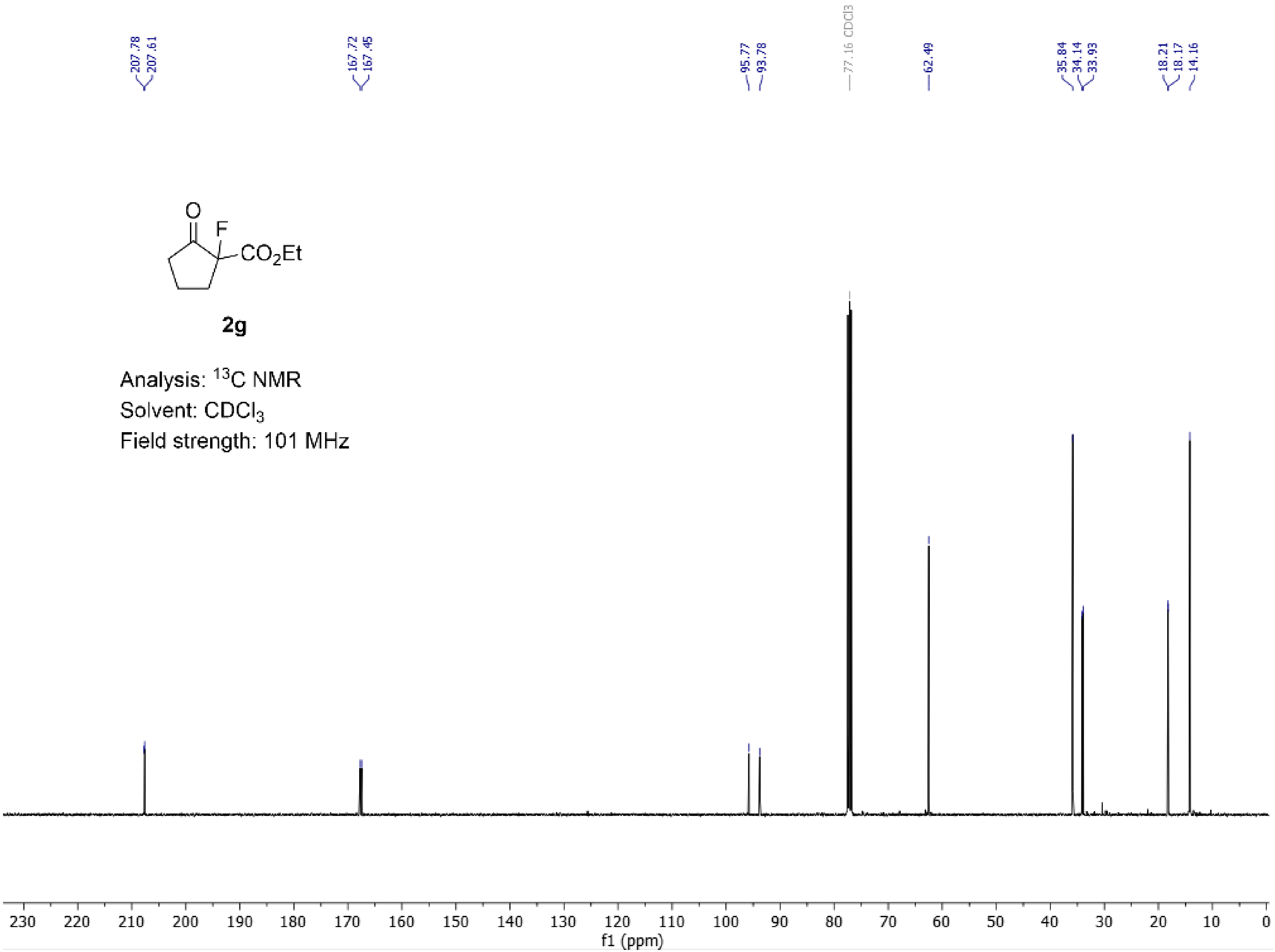
207.78
207.61
167.72
167.45
95.77
93.78
77.16 CDCl3
62.49
35.84
34.14
33.93
18.21
18.17
14.16
O
F
CO_2Et
2g
Analysis: ^{13}C NMR
Solvent: $CDCl_3$
Field strength: 101 MHz
230 220 210 200 190 180 170 160 150 140 130 120 110 100 90 80 70 60 50 40 30 20 10 0
f1 (ppm)

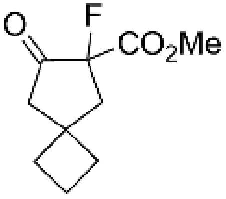

2h

Analysis: ^{1}H NMR

Solvent: $CDCl_3$

Field strength: 400 MHz

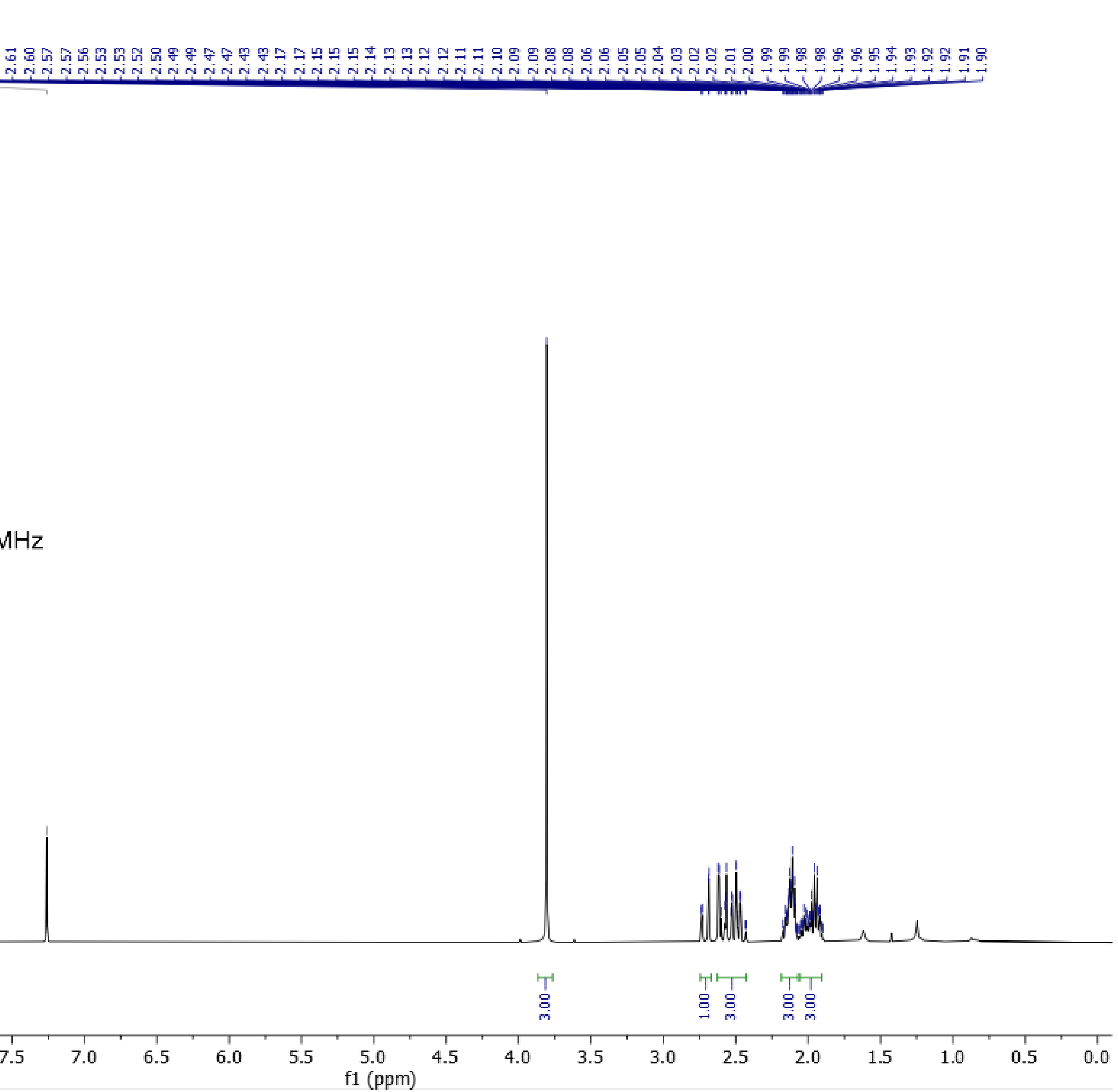

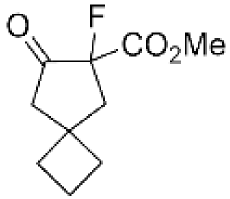

2h

Analysis: ^{19}F NMR
Solvent: $CDCl_3$
Field strength: 377 MHz

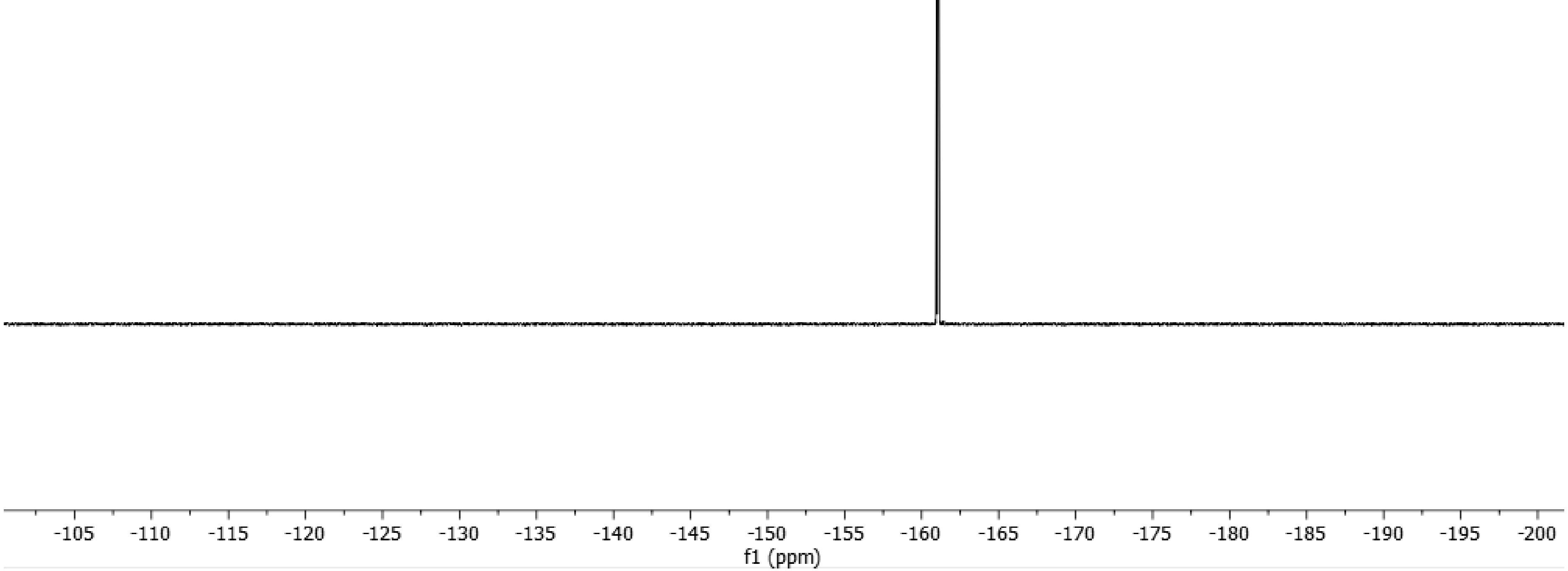

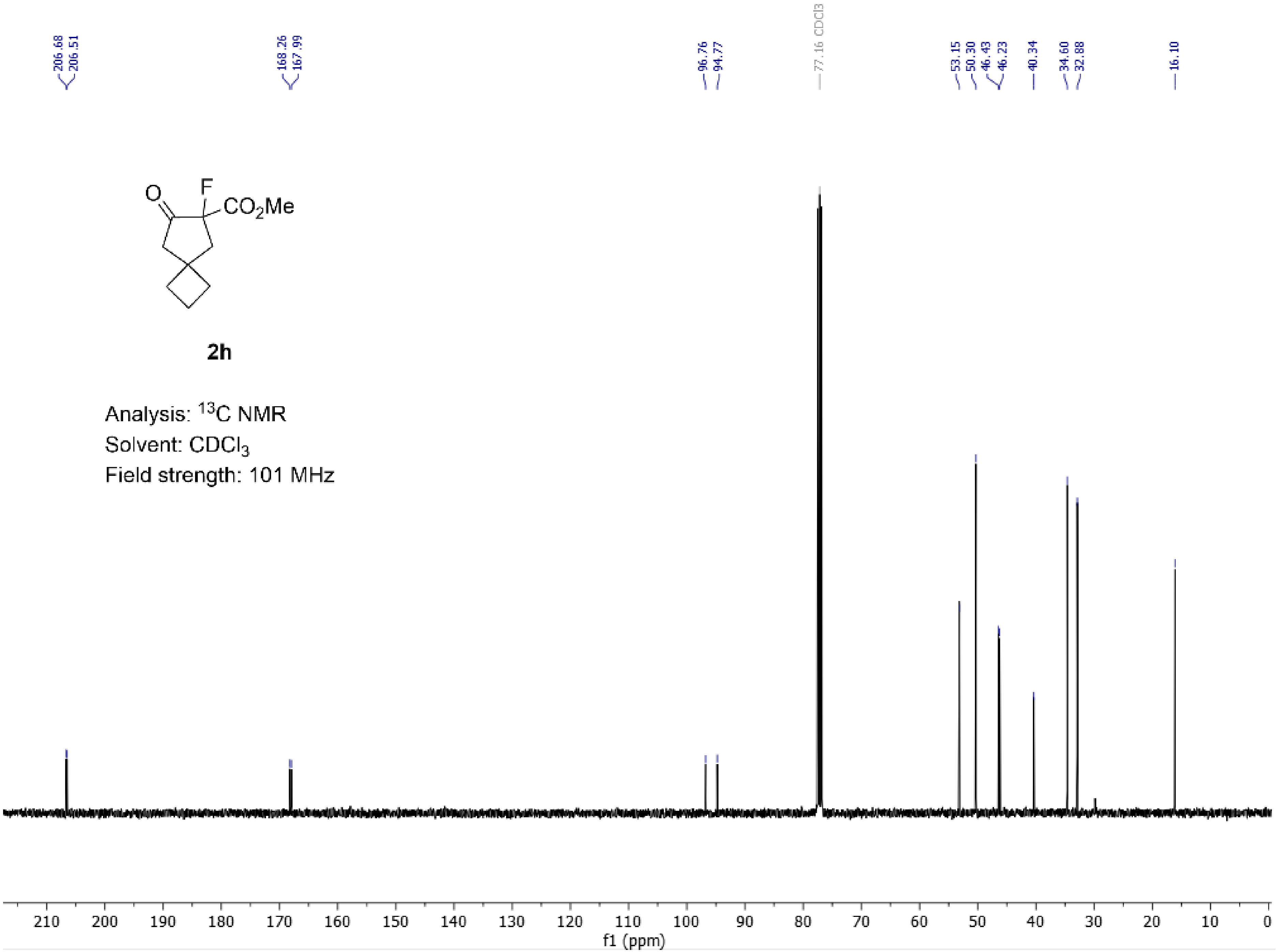

2h

Analysis: ^{13}C NMR
Solvent: $CDCl_3$
Field strength: 101 MHz

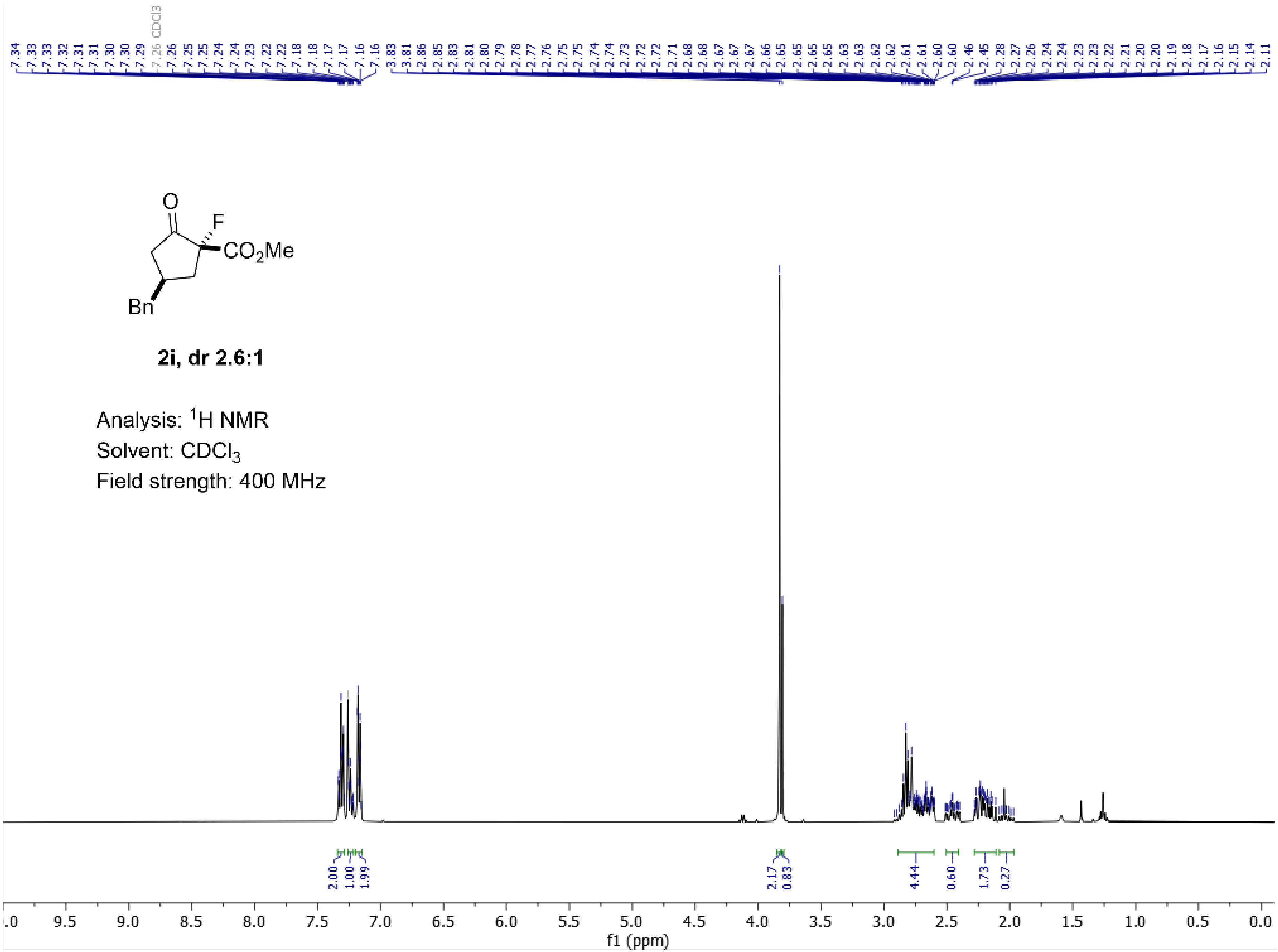

2i, dr 2.6:1
Analysis: 1H NMR
Solvent: CDCl3
Field strength: 400 MHz
f1 (ppm)

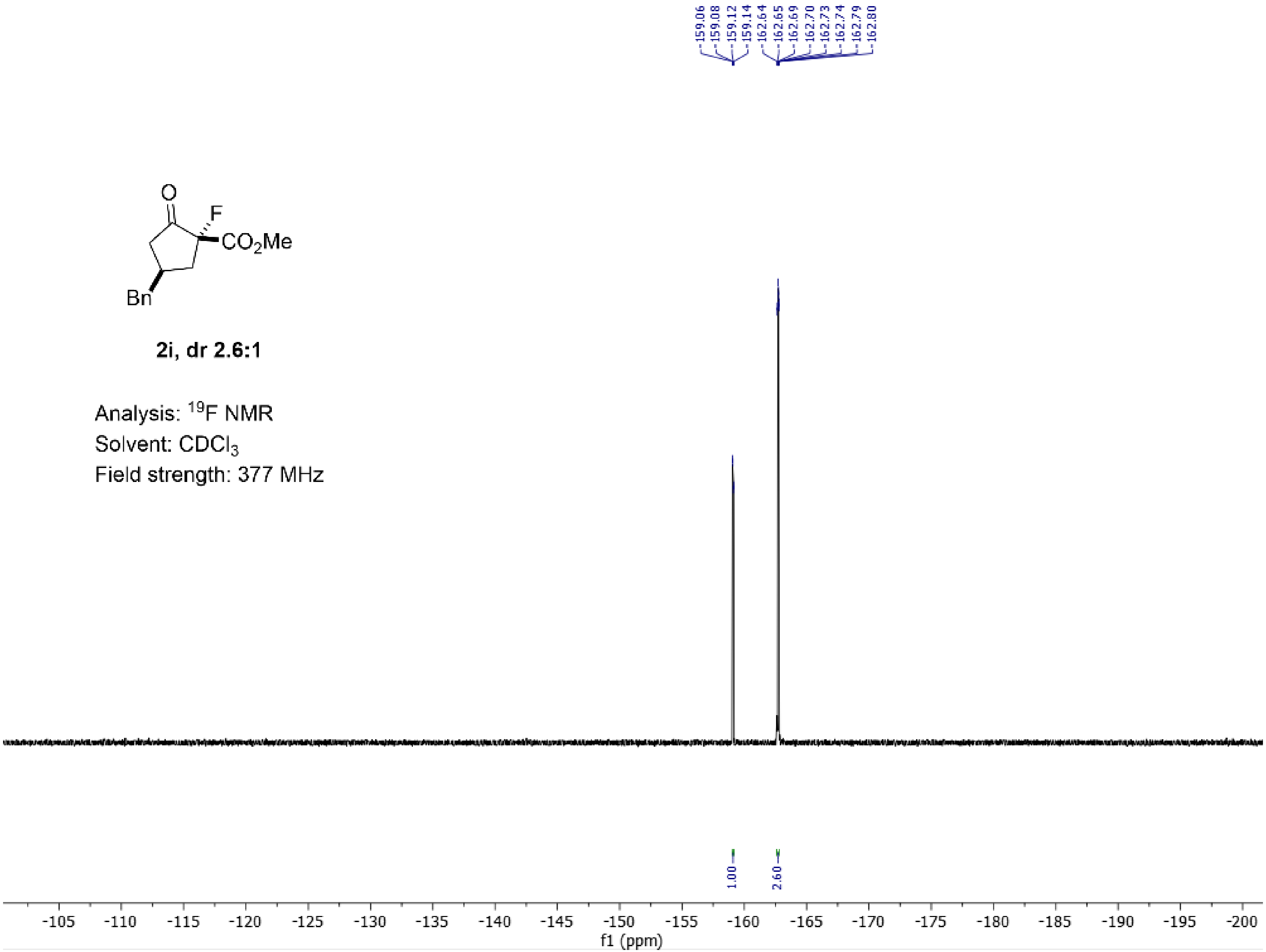

159.06
159.08
159.12
159.14
162.64
162.65
162.69
162.70
162.73
162.74
162.79
162.80
O
F
CO2Me
Bn
2i, dr 2.6:1
Analysis: 19F NMR
Solvent: CDCl3
Field strength: 377 MHz
1.00
2.60
-105 -110 -115 -120 -125 -130 -135 -140 -145 -150 -155 -160 -165 -170 -175 -180 -185 -190 -195 -200
f1 (ppm)

2i, dr 2.6:1

Analysis: ^{13}C NMR
Solvent: $CDCl_3$
Field strength: 101 MHz

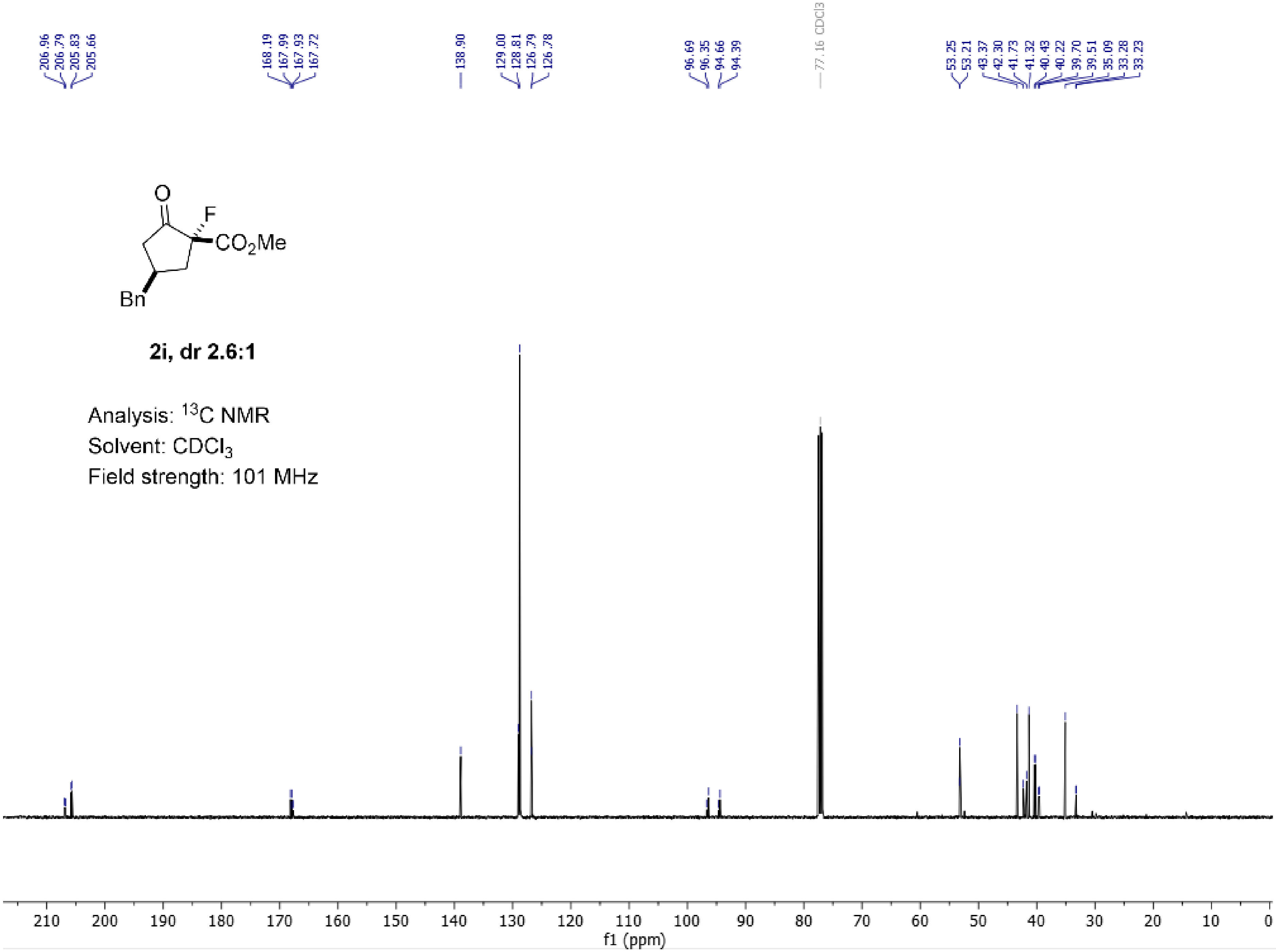

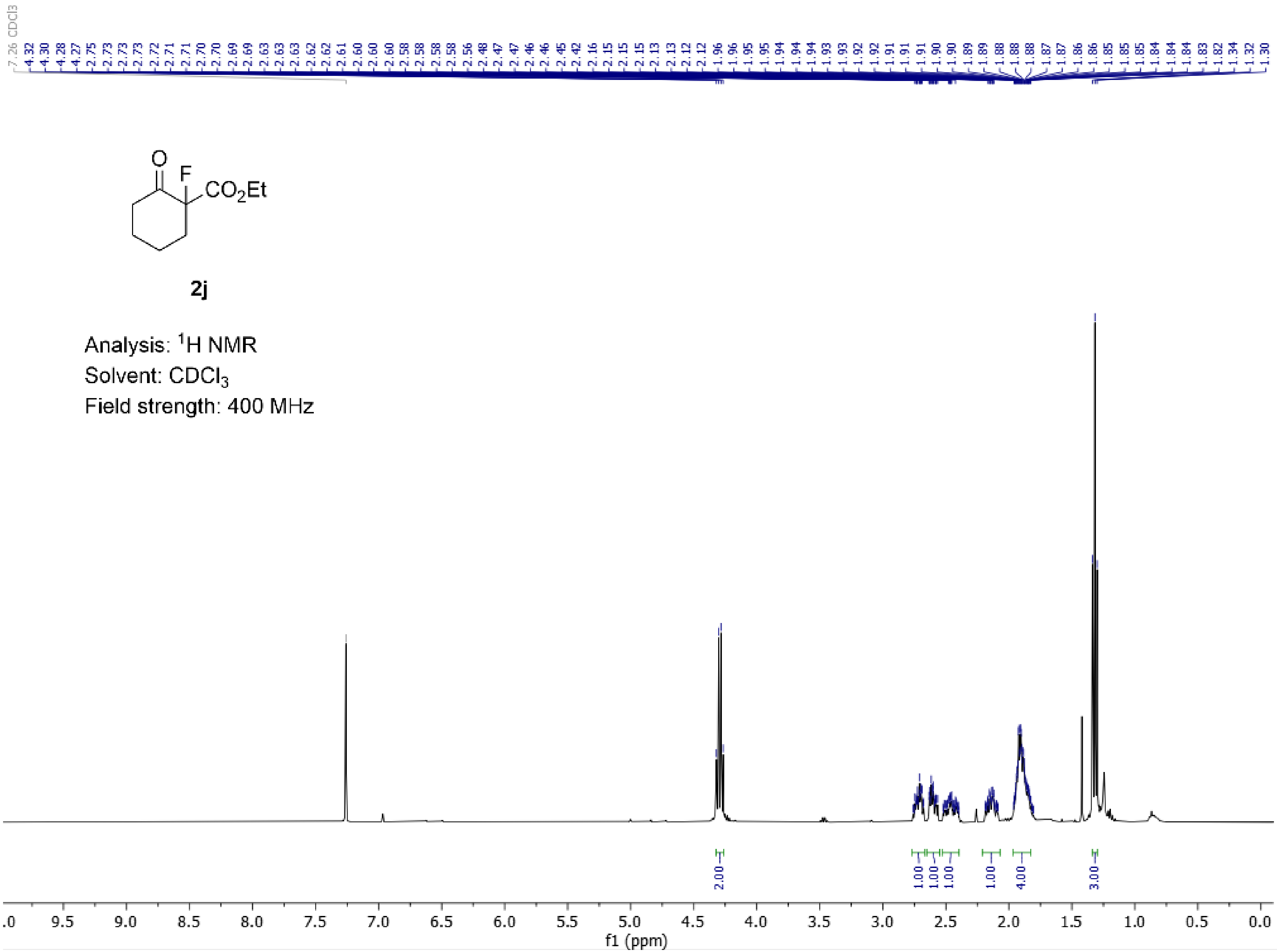

2j
Analysis: ¹H NMR
Solvent: $CDCl_3$
Field strength: 400 MHz
f1 (ppm)

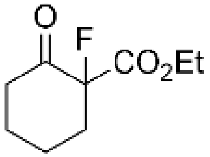

2j

Analysis: ^{19}F NMR
Solvent: $CDCl_3$
Field strength: 377 MHz

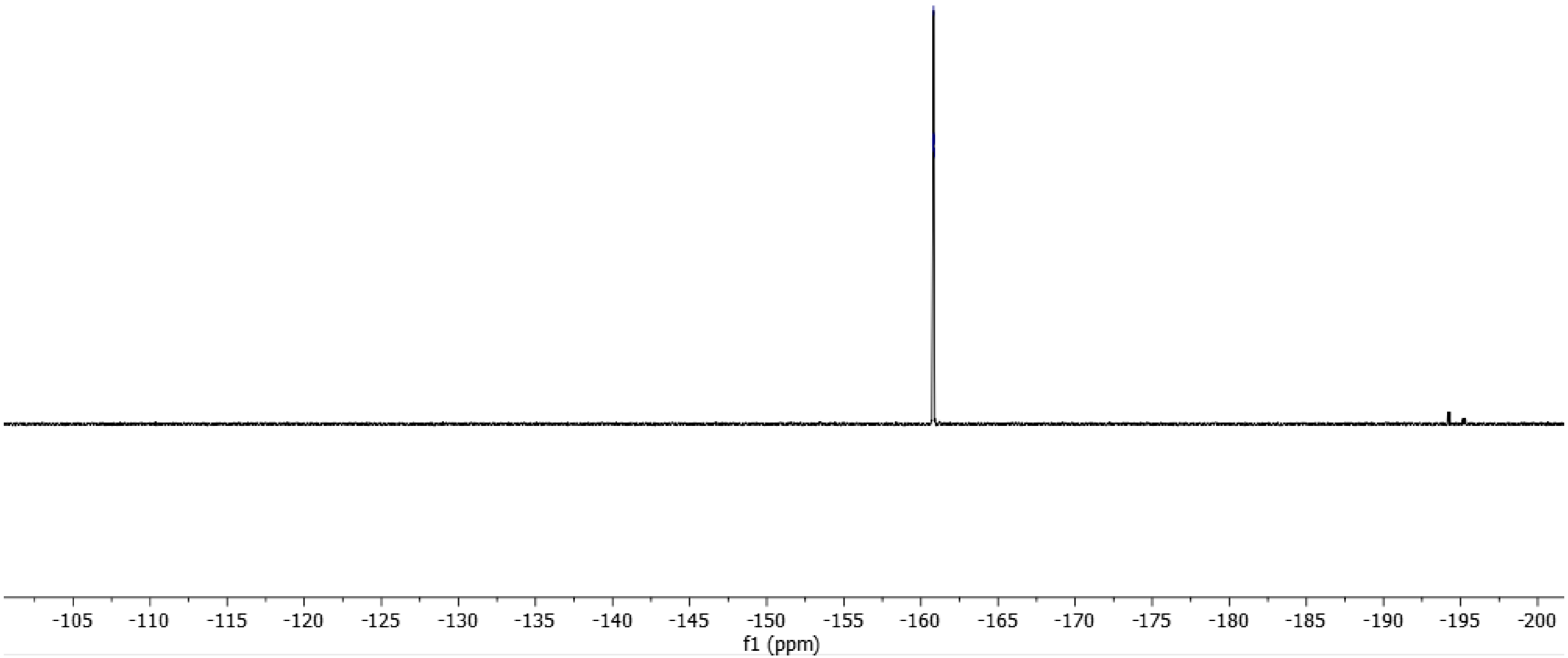

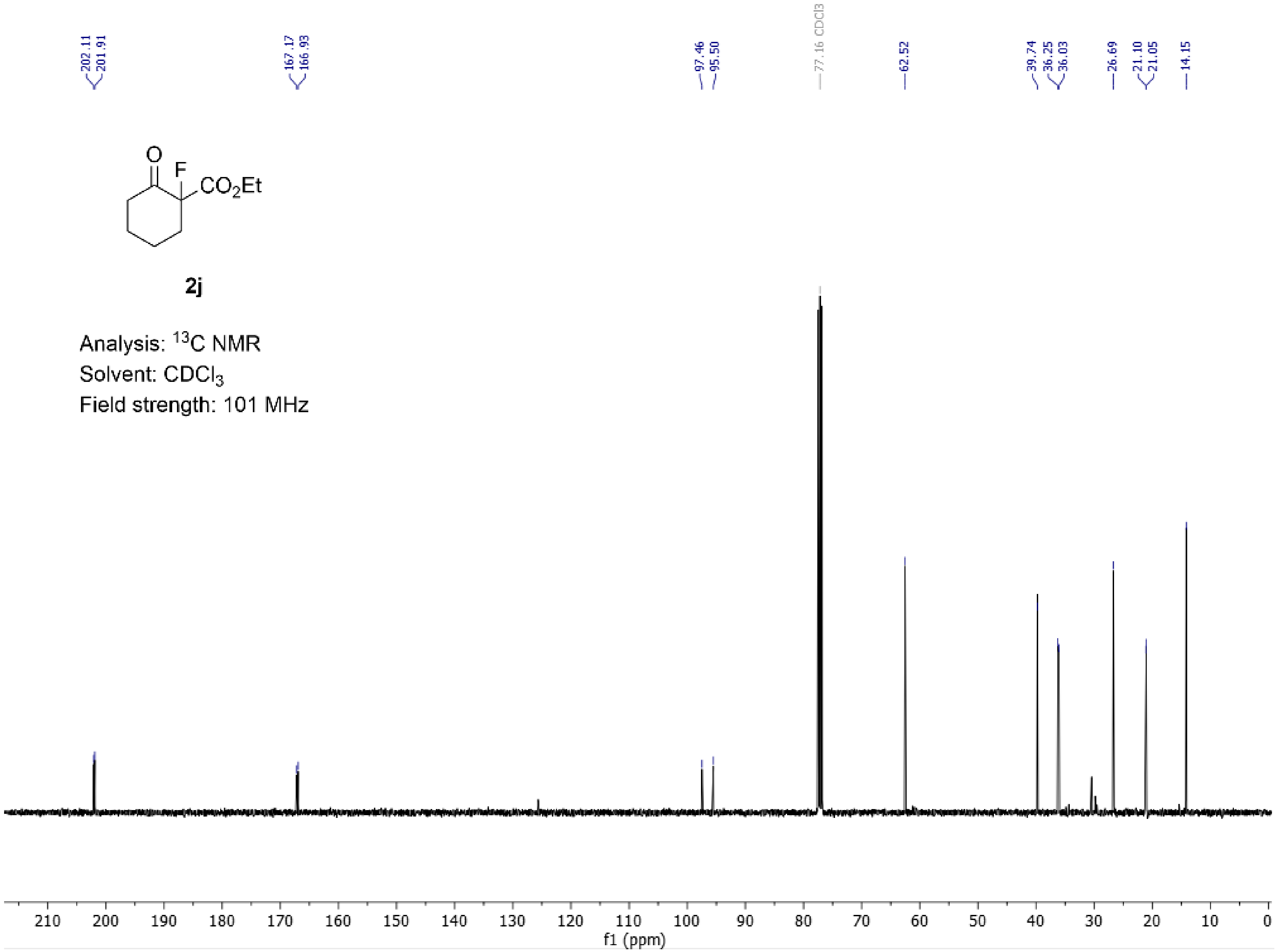
202.11
201.91
167.17
166.93
97.46
95.50
77.16 CDCl3
62.52
39.74
36.25
36.03
26.69
21.10
21.05
14.15
O
F
CO_2Et
2j
Analysis: ^{13}C NMR
Solvent: $CDCl_3$
Field strength: 101 MHz
210 200 190 180 170 160 150 140 130 120 110 100 90 80 70 60 50 40 30 20 10 0
f1 (ppm)

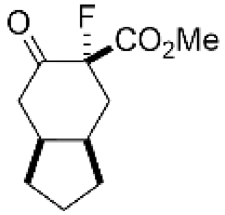

2k-1

Analysis: ^{1}H NMR
Solvent: $CDCl_3$
Field strength: 400 MHz

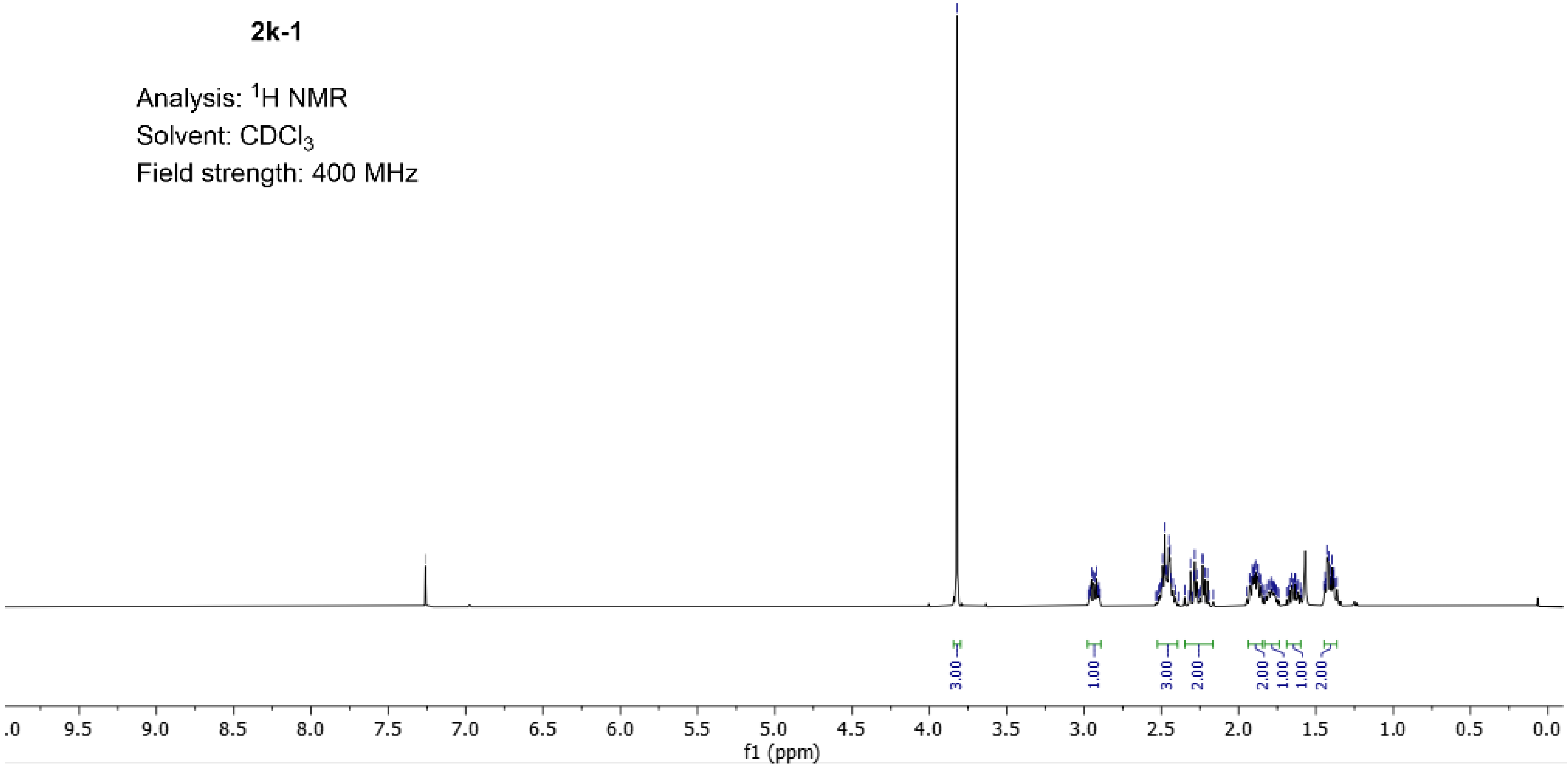

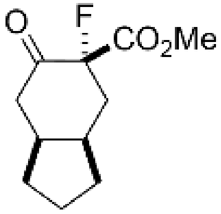

2k-1

Analysis: ^{19}F NMR
Solvent: $CDCl_3$
Field strength: 377 MHz

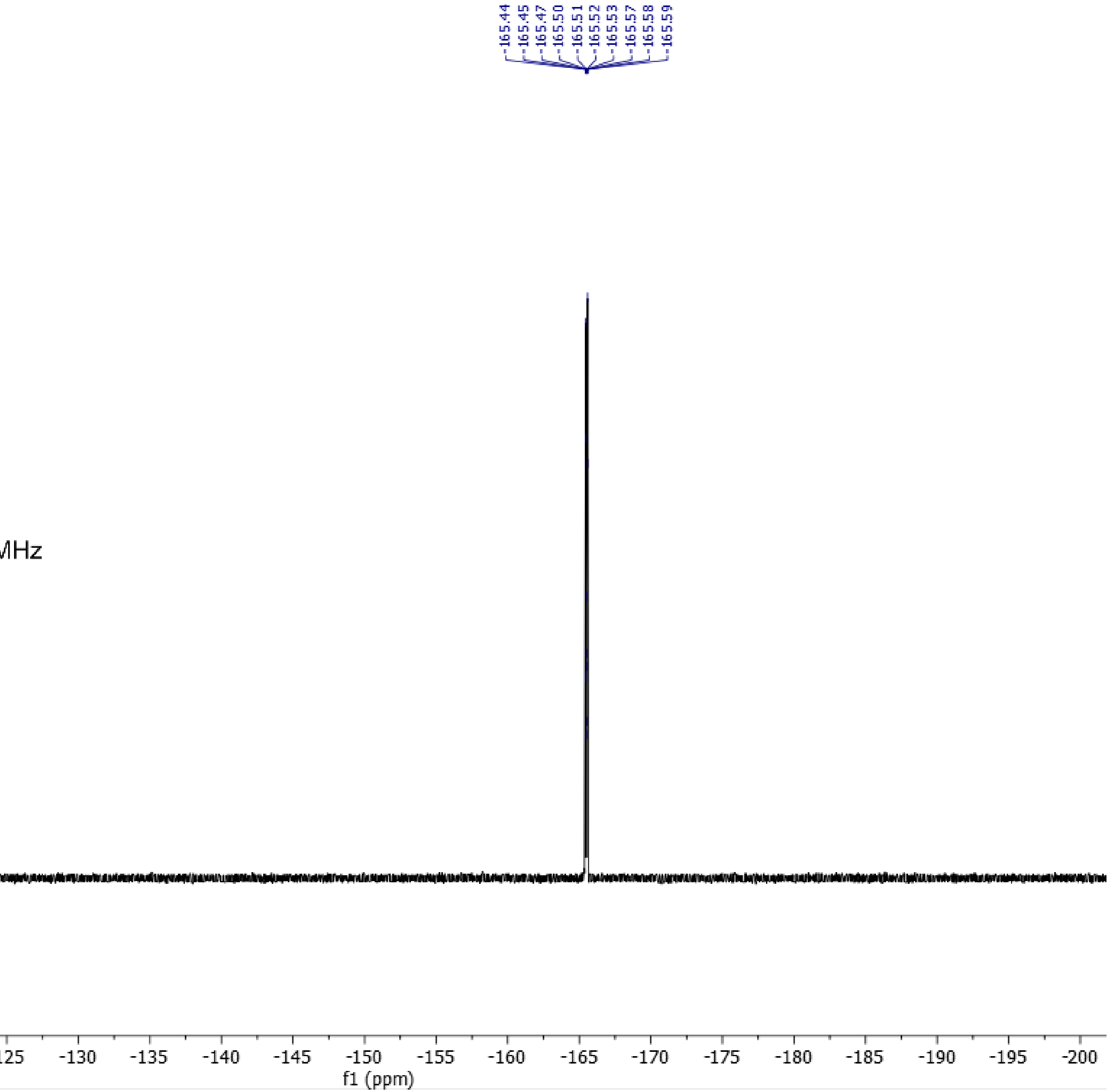

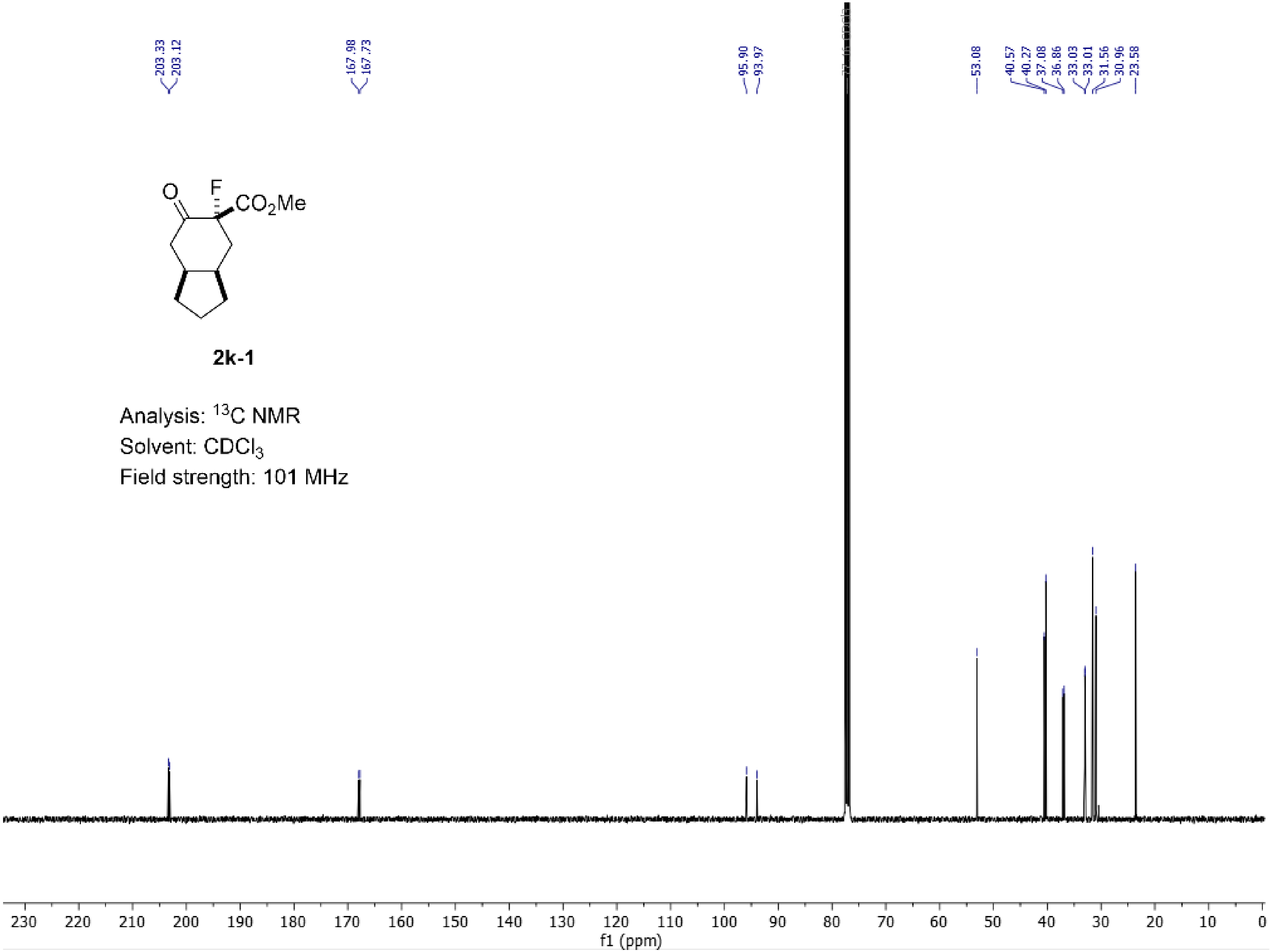

2k-1

Analysis: ^{13}C NMR

Solvent: $CDCl_3$

Field strength: 101 MHz

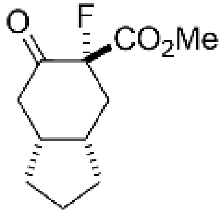

2k-2

Analysis: ^{1}H NMR
Solvent: $CDCl_3$
Field strength: 400 MHz

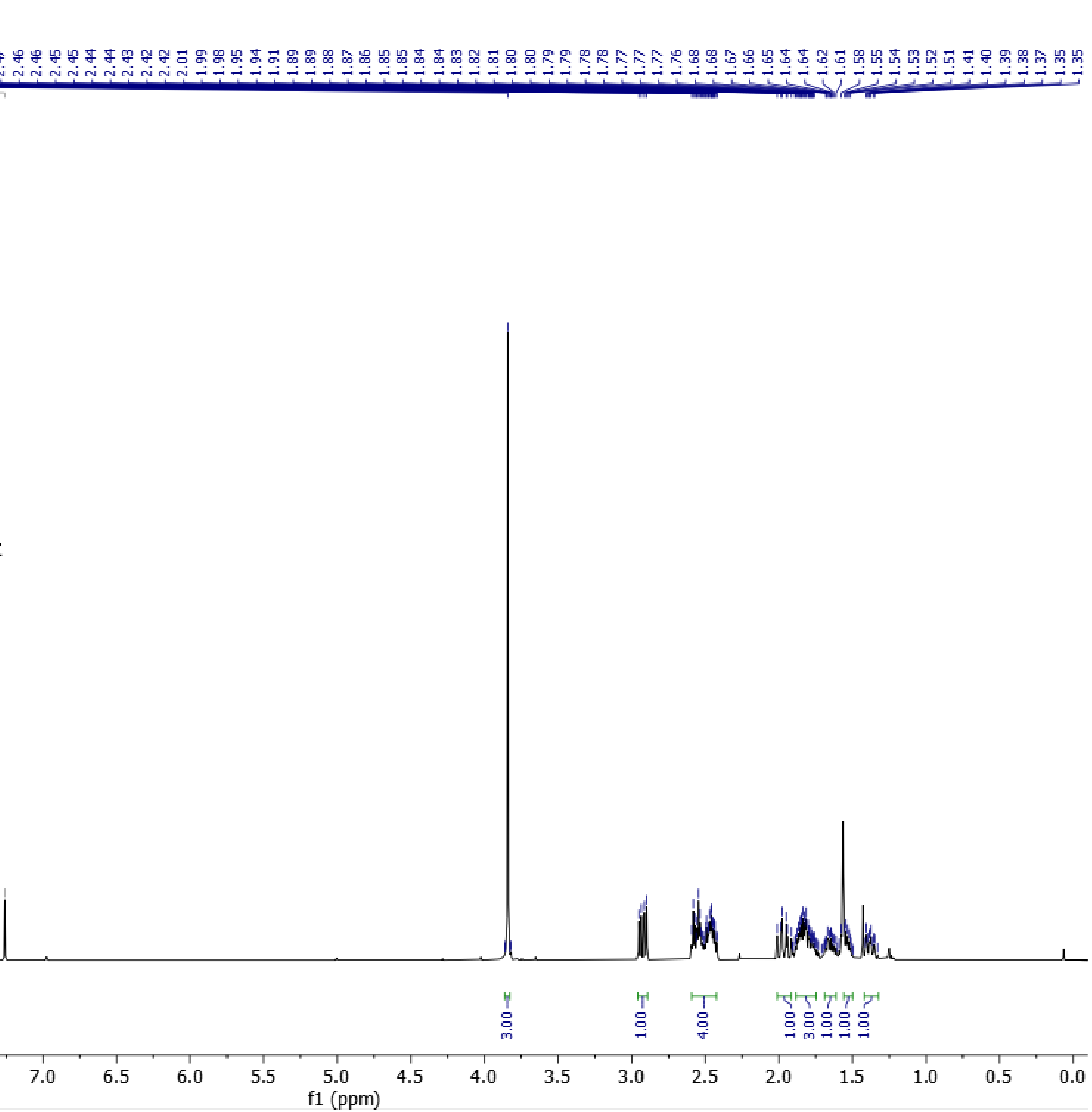

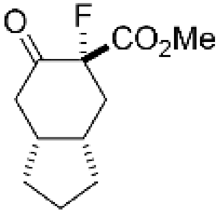

2k-2

Analysis: ^{19}F NMR
Solvent: $CDCl_3$
Field strength: 377 MHz

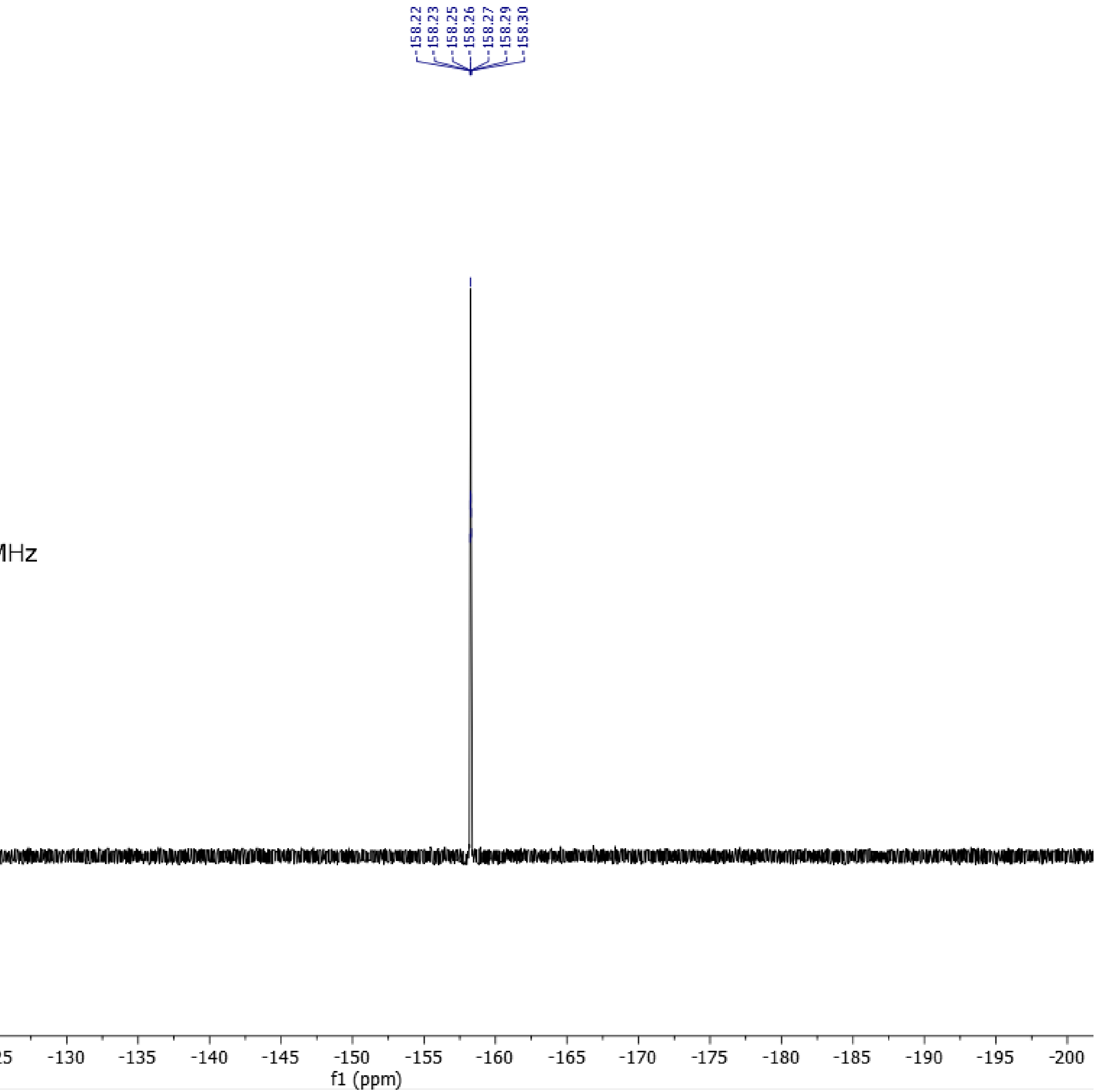

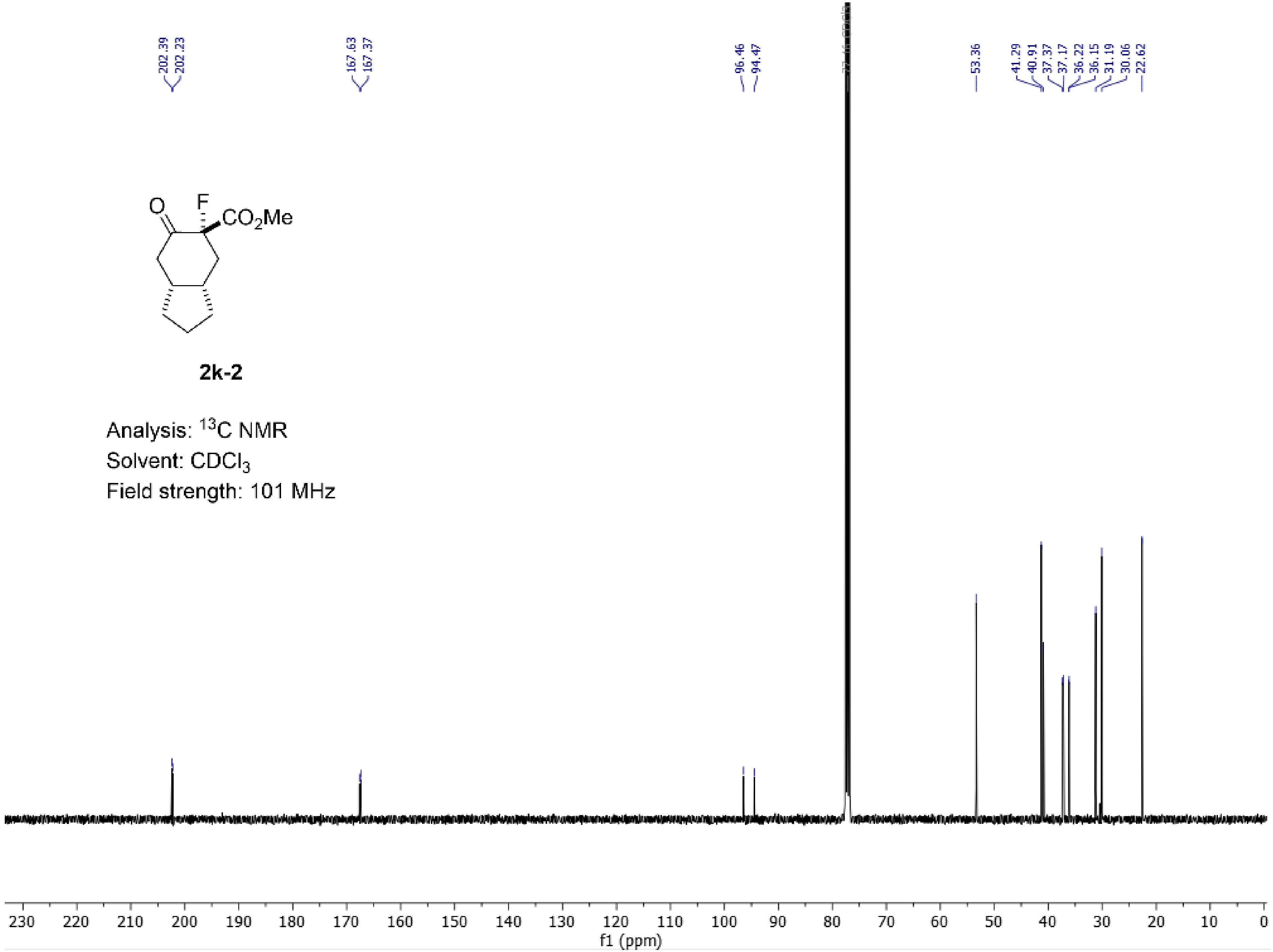
202.39
202.23
167.63
167.37
96.46
94.47
53.36
41.29
40.91
37.37
37.17
36.22
36.15
31.19
30.06
22.62
O
F
CO2Me
2k-2
Analysis: 13C NMR
Solvent: CDCl3
Field strength: 101 MHz
230 220 210 200 190 180 170 160 150 140 130 120 110 100 90 80 70 60 50 40 30 20 10 0
f1 (ppm)

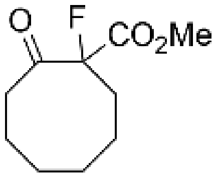

2l

Analysis: ^{1}H NMR
Solvent: $CDCl_3$
Field strength: 400 MHz

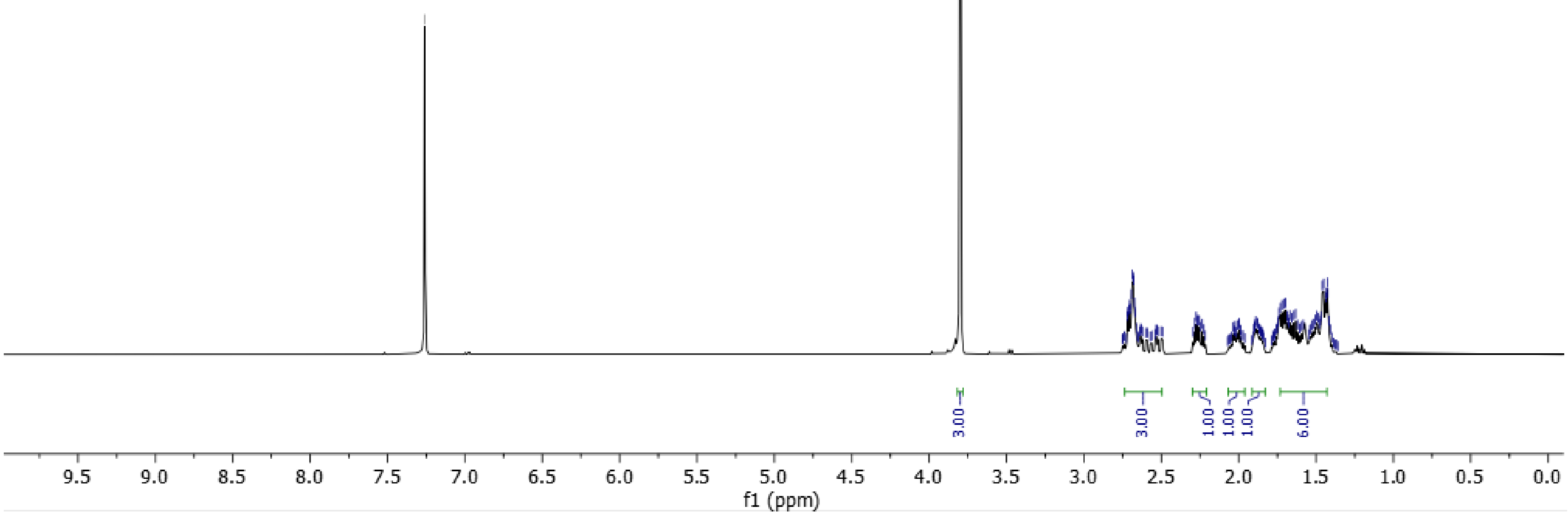

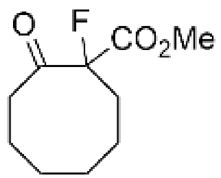

2l

Analysis: ^{19}F NMR
Solvent: $CDCl_3$
Field strength: 377 MHz

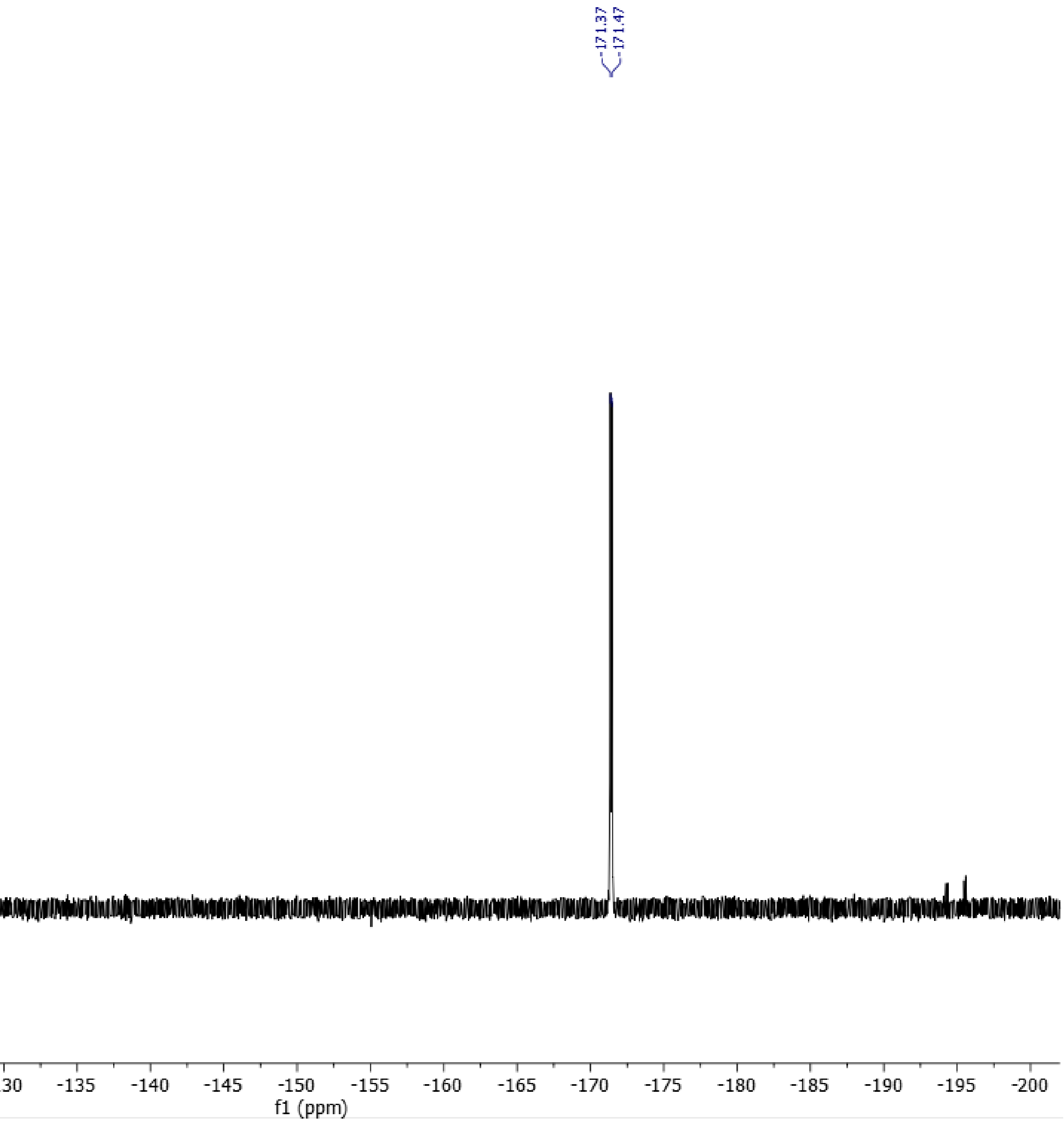

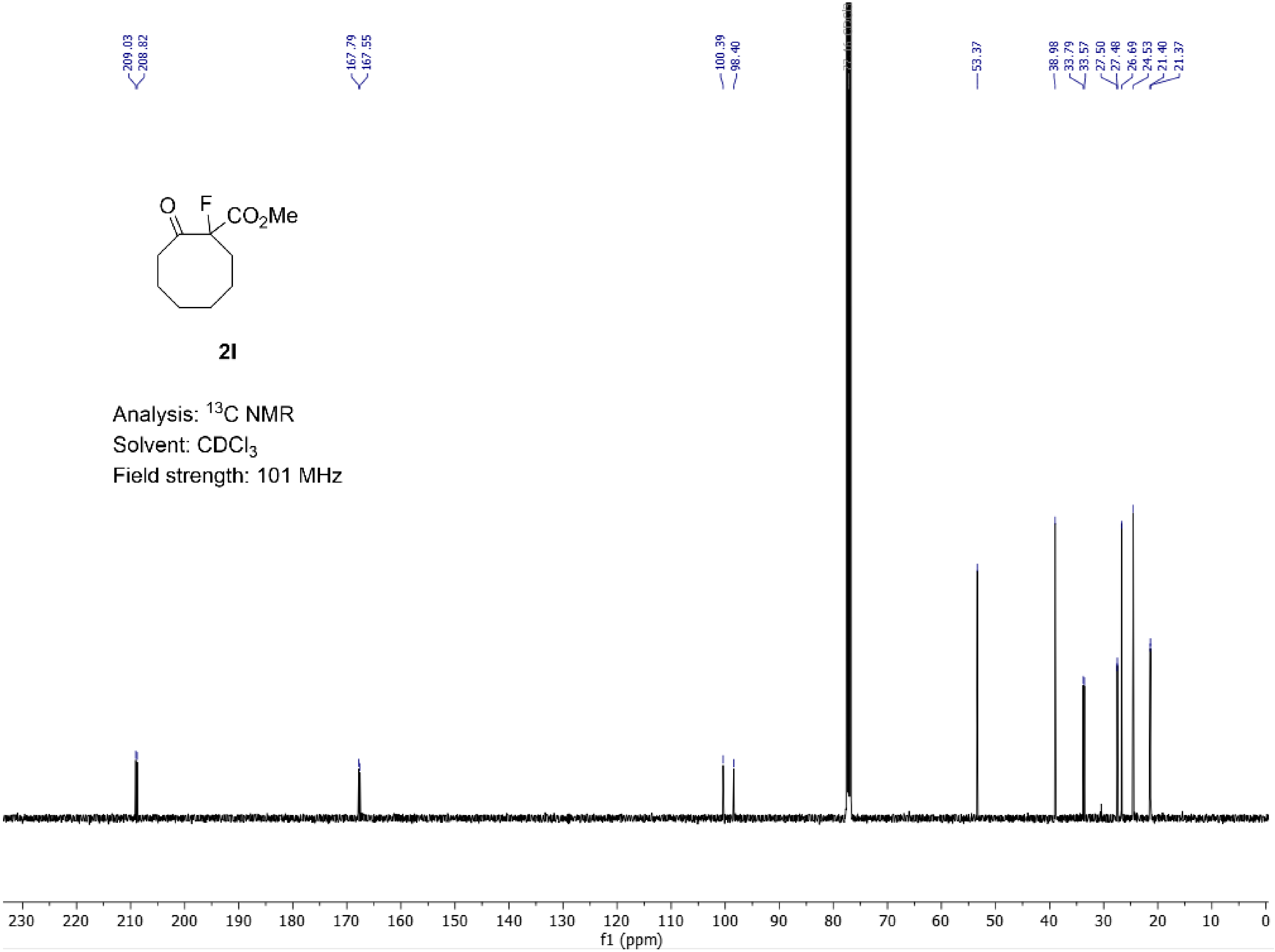
209.03
208.82
167.79
167.55
100.39
98.40
53.37
38.98
33.79
33.57
27.50
27.48
26.69
24.53
21.40
21.37
O
F
CO_2Me
2l
Analysis: ^{13}C NMR
Solvent: $CDCl_3$
Field strength: 101 MHz
230 220 210 200 190 180 170 160 150 140 130 120 110 100 90 80 70 60 50 40 30 20 10 0
f1 (ppm)

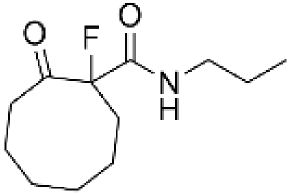

2m

Analysis: ^{1}H NMR
Solvent: $CDCl_3$
Field strength: 400 MHz

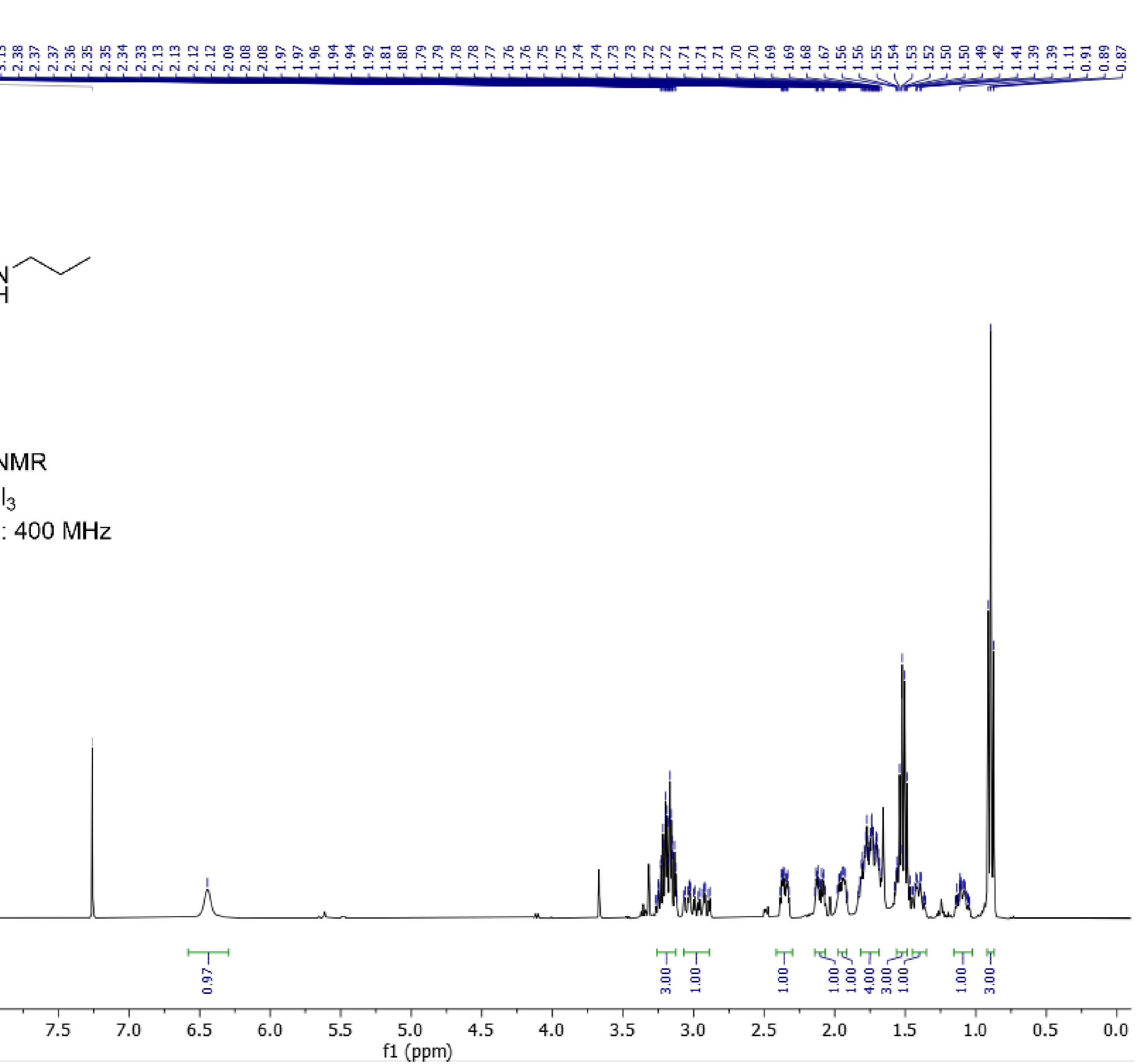

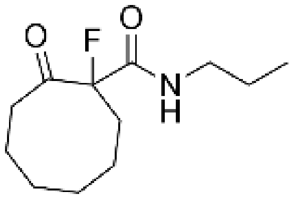

2m

Analysis: ^{19}F NMR
Solvent: $CDCl_3$
Field strength: 377 MHz

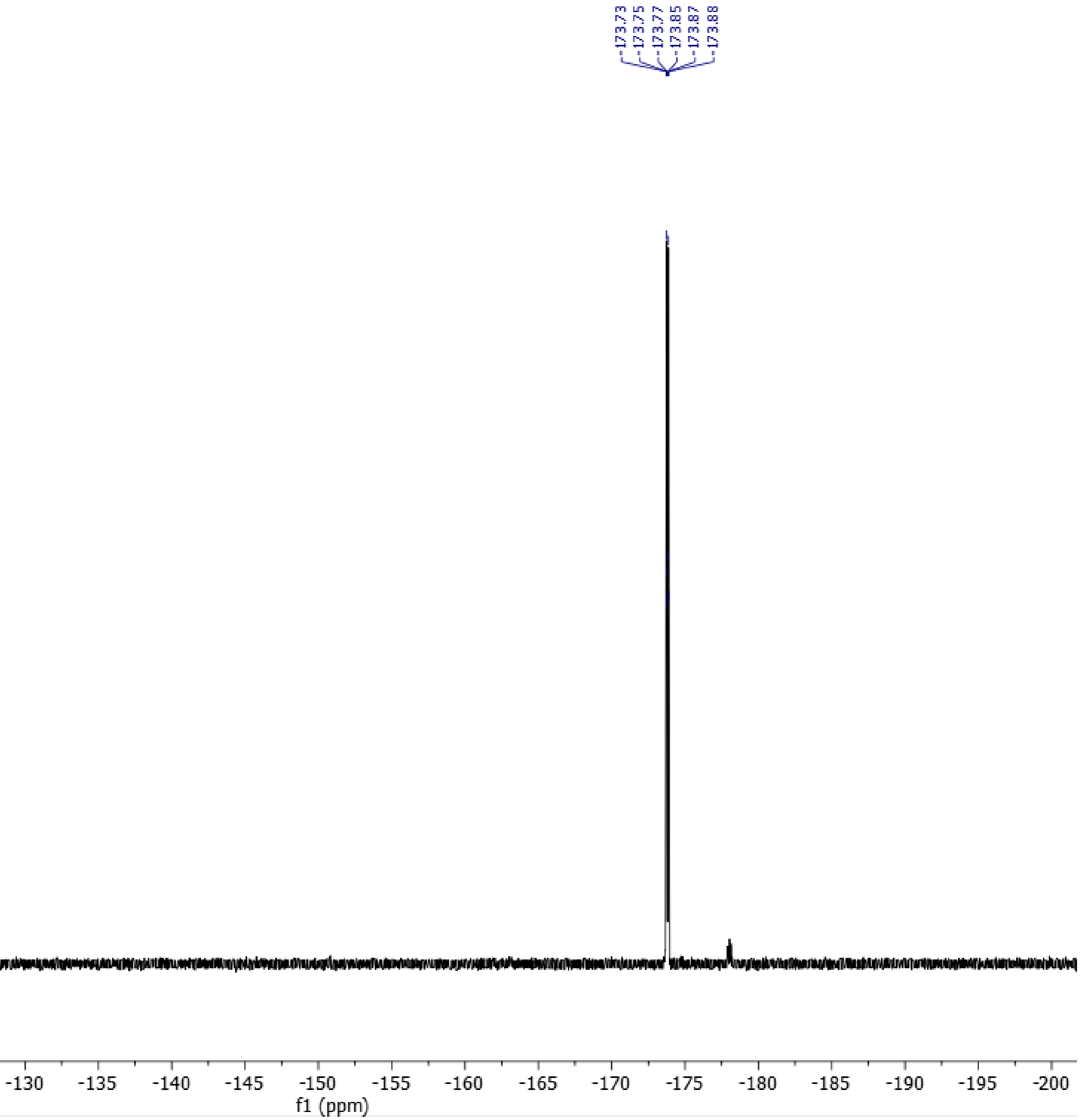

2m

Analysis: ^{13}C NMR

Solvent: $CDCl_3$

Field strength: 101 MHz

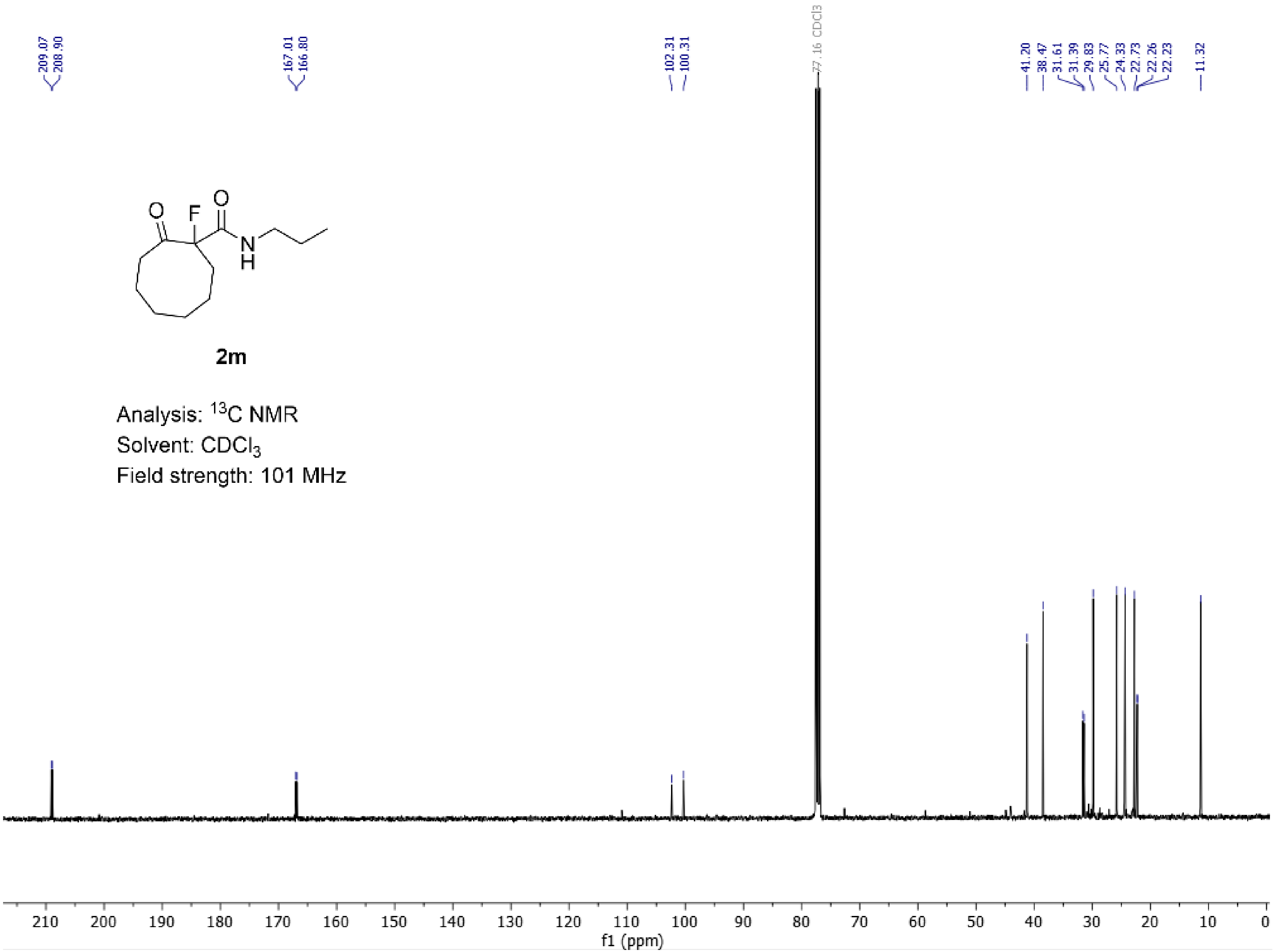

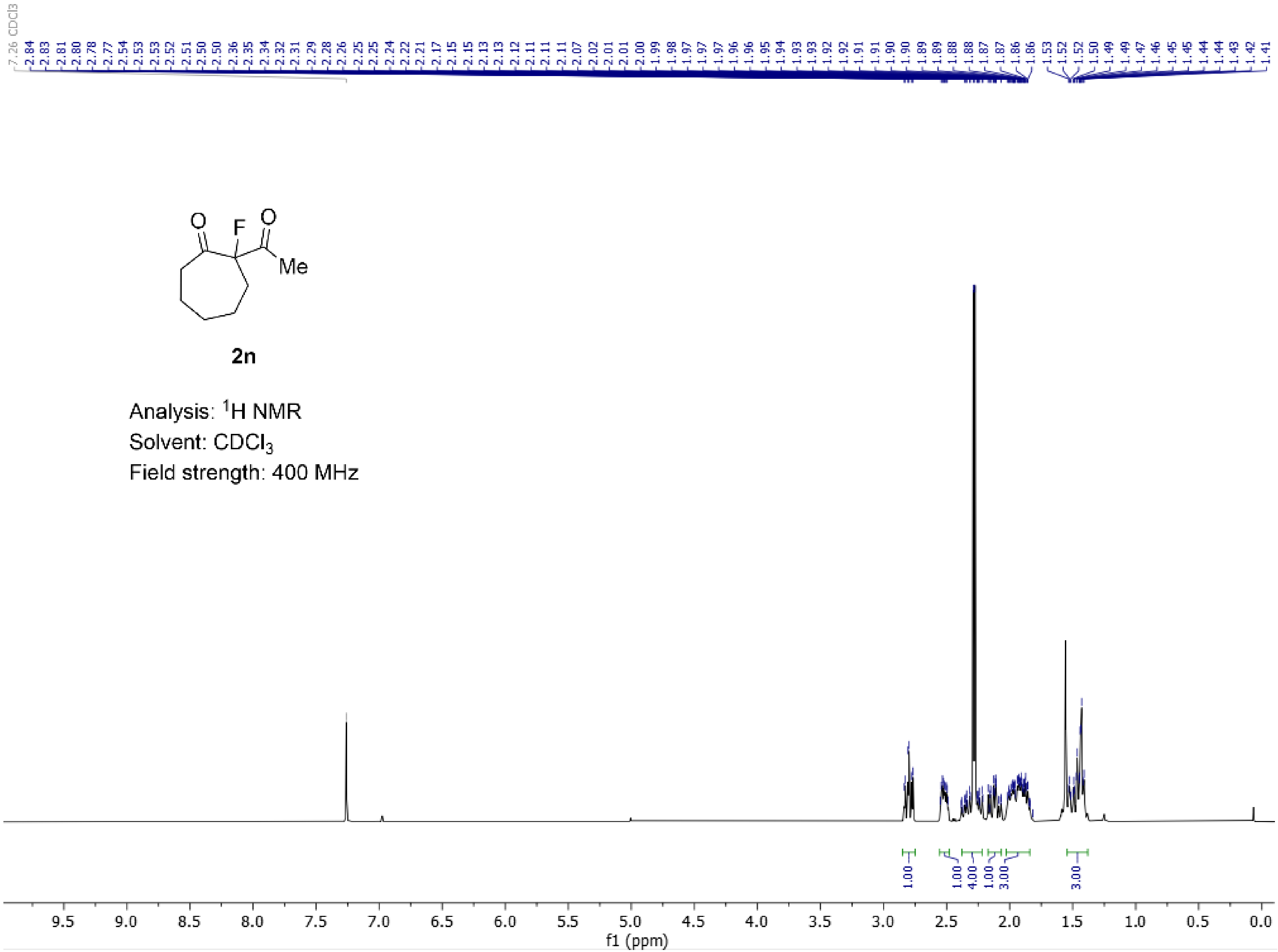
7.26 CDCl3
2.84 2.83 2.81 2.80 2.78 2.77 2.54 2.53 2.53 2.52 2.51 2.50 2.50 2.36 2.35 2.34 2.32 2.31 2.29 2.28 2.26 2.25 2.25 2.24 2.22 2.21 2.17 2.15 2.15 2.13 2.13 2.12 2.11 2.11 2.11 2.07 2.02 2.01 2.01 2.00 1.99 1.98 1.97 1.97 1.97 1.96 1.96 1.95 1.94 1.93 1.93 1.92 1.92 1.91 1.91 1.90 1.90 1.89 1.89 1.88 1.88 1.87 1.87 1.86 1.86
1.53 1.52 1.52 1.50 1.49 1.49 1.47 1.46 1.45 1.45 1.44 1.44 1.43 1.42 1.41
O
F
O
Me
2n
Analysis: 1H NMR
Solvent: CDCl3
Field strength: 400 MHz
1.00
1.00
4.00
1.00
3.00
3.00
9.5 9.0 8.5 8.0 7.5 7.0 6.5 6.0 5.5 5.0 4.5 4.0 3.5 3.0 2.5 2.0 1.5 1.0 0.5 0.0
f1 (ppm)

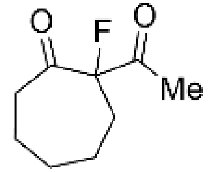

2n

Analysis: ^{19}F NMR
Solvent: $CDCl_3$
Field strength: 377 MHz

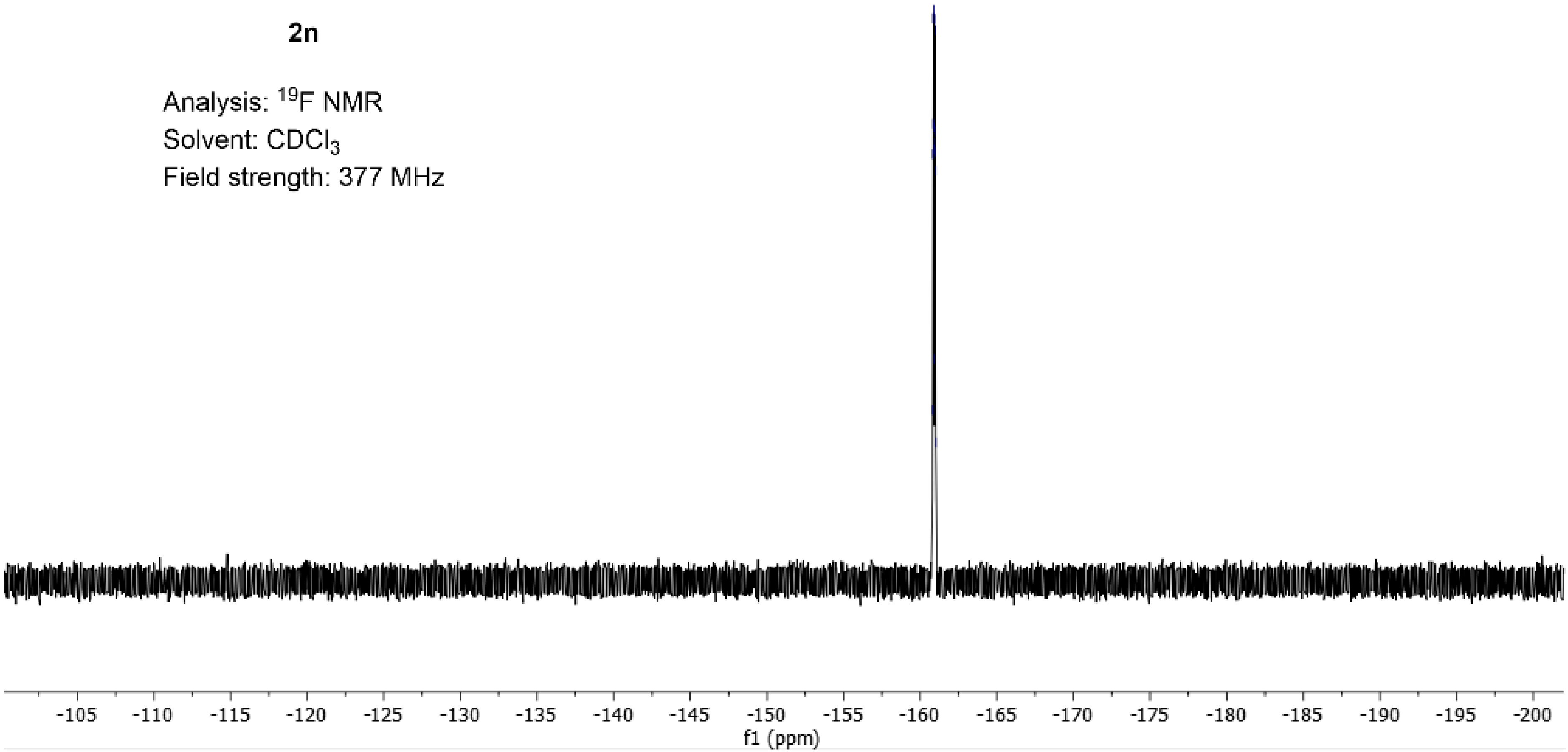

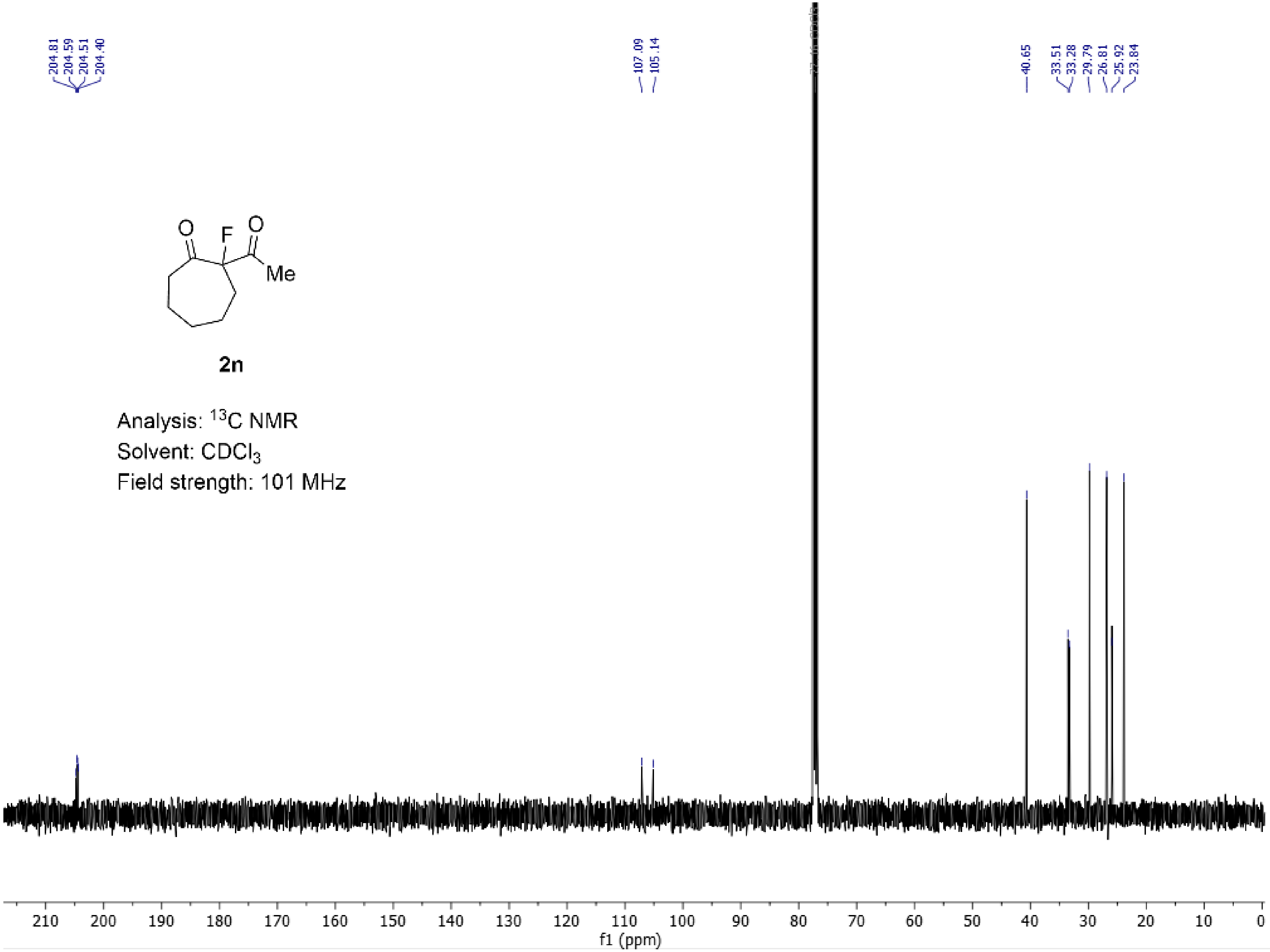
204.81
204.59
204.51
204.40
107.09
105.14
40.65
33.51
33.28
29.79
26.81
25.92
23.84
O
F
O
Me
2n
Analysis: 13C NMR
Solvent: CDCl3
Field strength: 101 MHz
210 200 190 180 170 160 150 140 130 120 110 100 90 80 70 60 50 40 30 20 10 0
f1 (ppm)

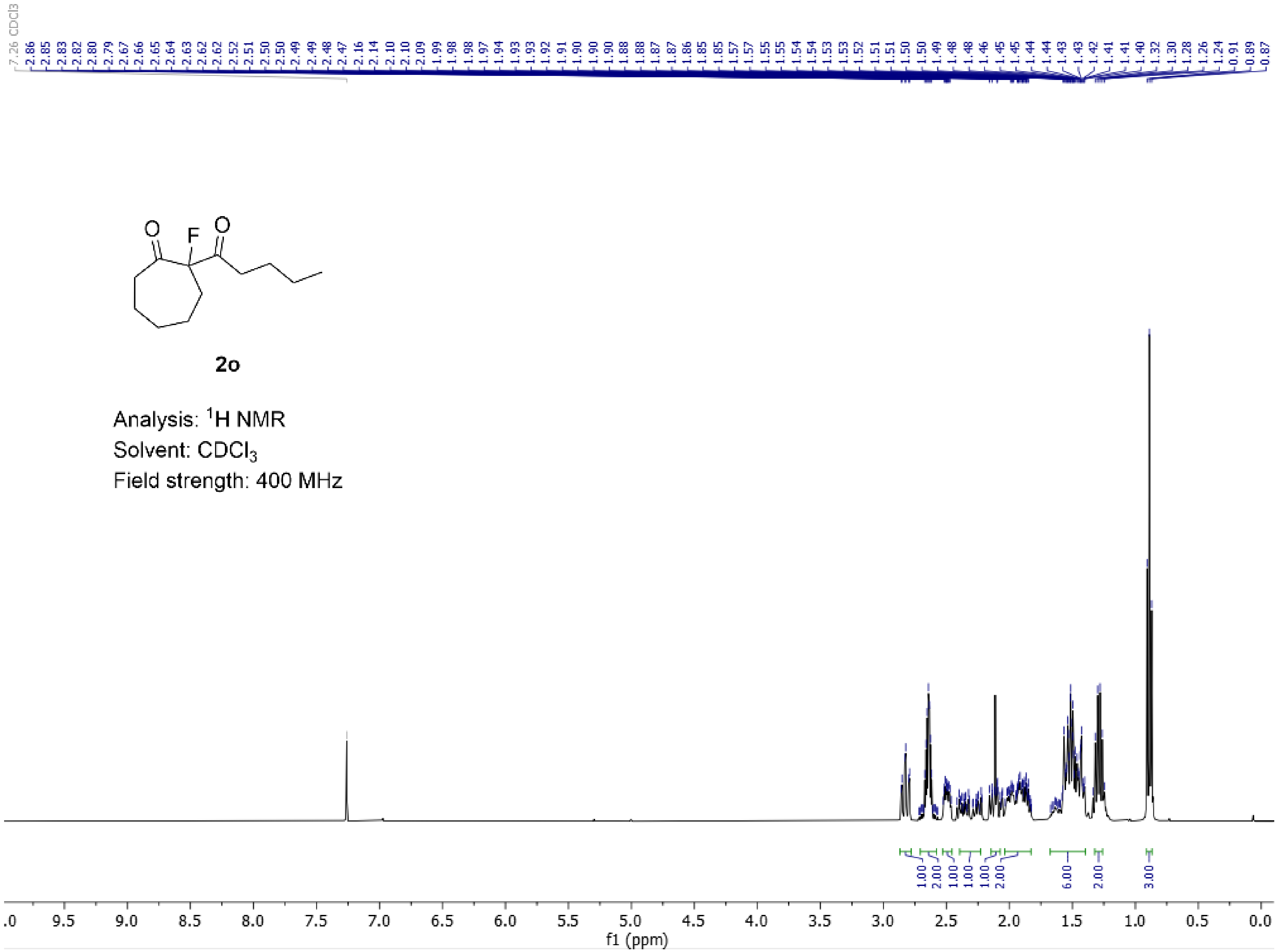

2o
Analysis: 1H NMR
Solvent: CDCl3
Field strength: 400 MHz
f1 (ppm)

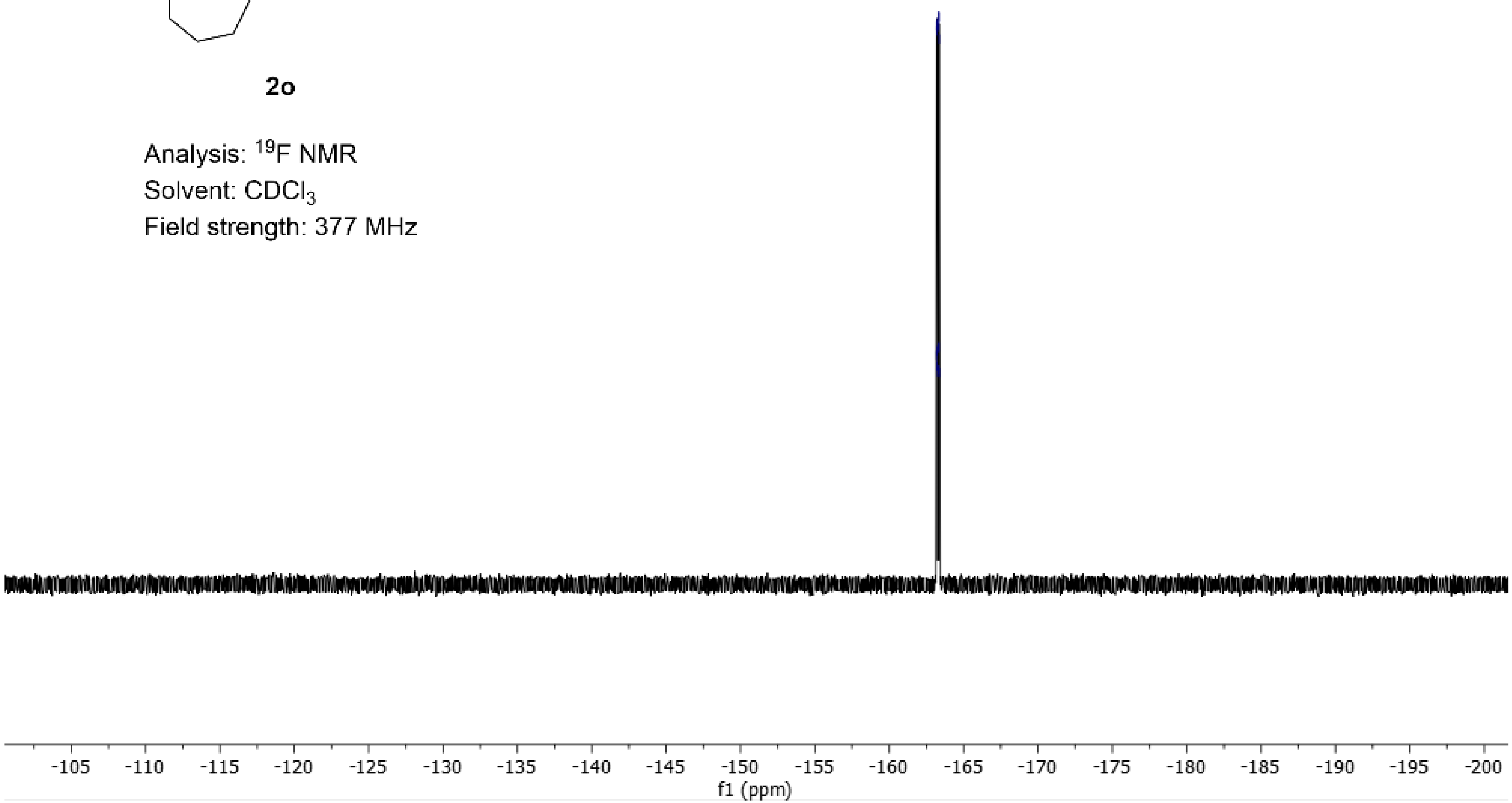

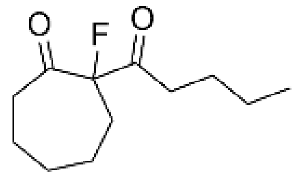

2o

Analysis: ^{19}F NMR
Solvent: $CDCl_3$
Field strength: 377 MHz

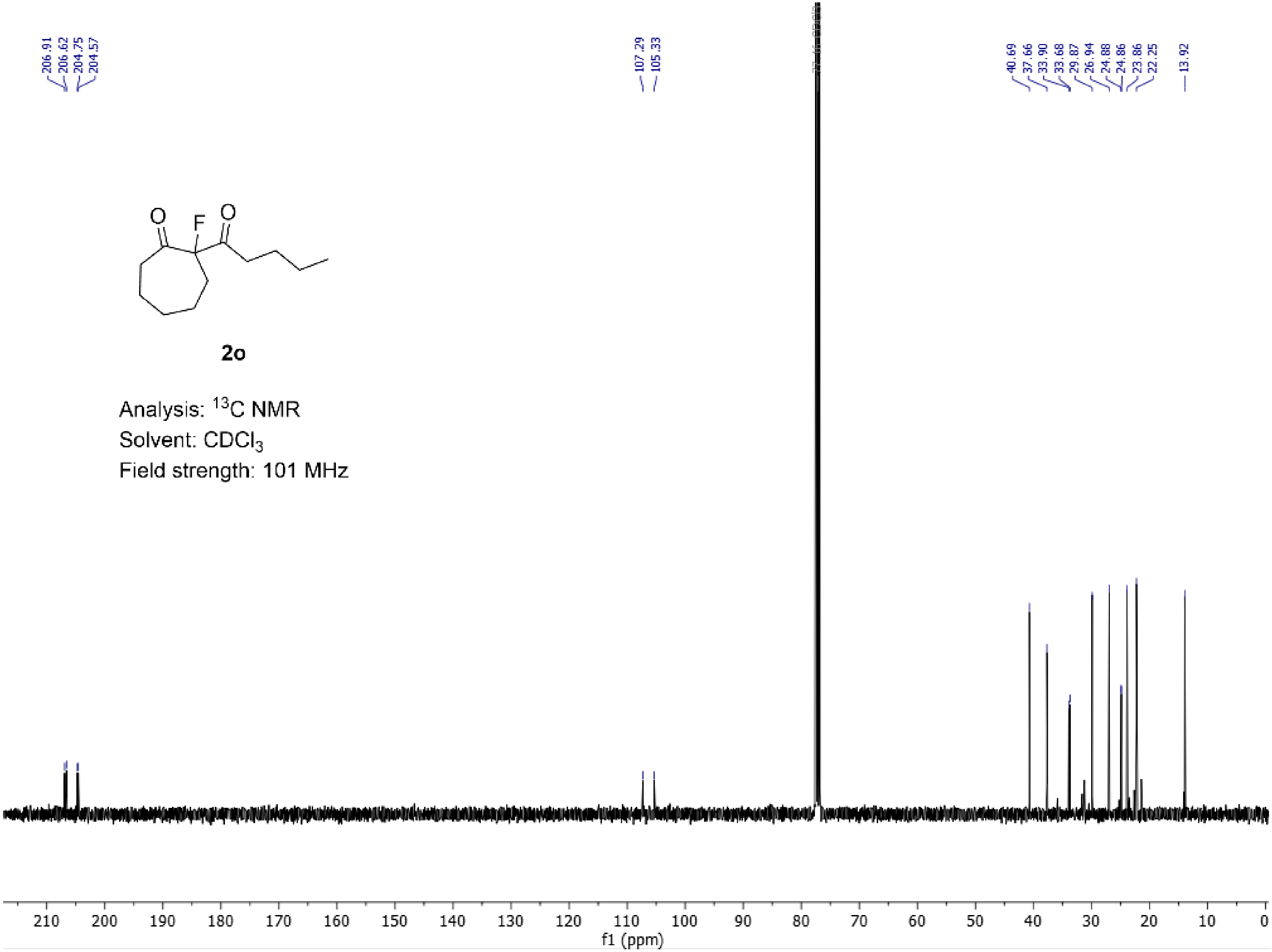
206.91
206.62
204.75
204.57
107.29
105.33
40.69
37.66
33.90
33.68
29.87
26.94
24.88
24.86
23.86
22.25
13.92
O
F
O
2o
Analysis: ^{13}C NMR
Solvent: $CDCl_3$
Field strength: 101 MHz
210 200 190 180 170 160 150 140 130 120 110 100 90 80 70 60 50 40 30 20 10 0
f1 (ppm)

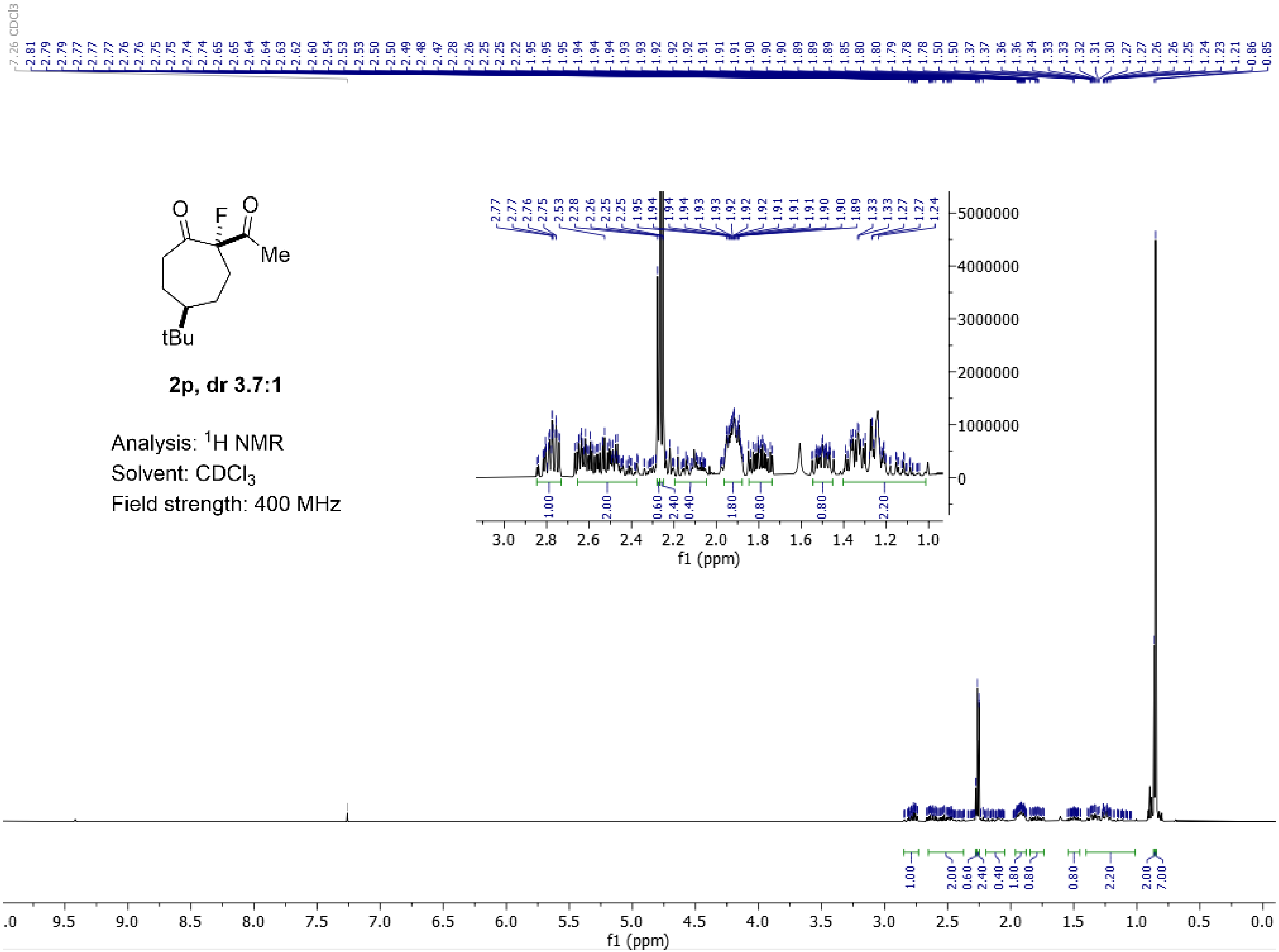

2p, dr 3.7:1
Analysis: 1H NMR
Solvent: CDCl3
Field strength: 400 MHz
f1 (ppm)

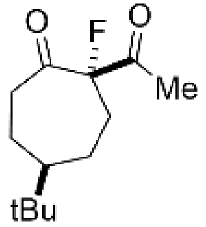

2p, dr 3.7:1

Analysis: ^{19}F NMR
Solvent: $CDCl_3$
Field strength: 377 MHz

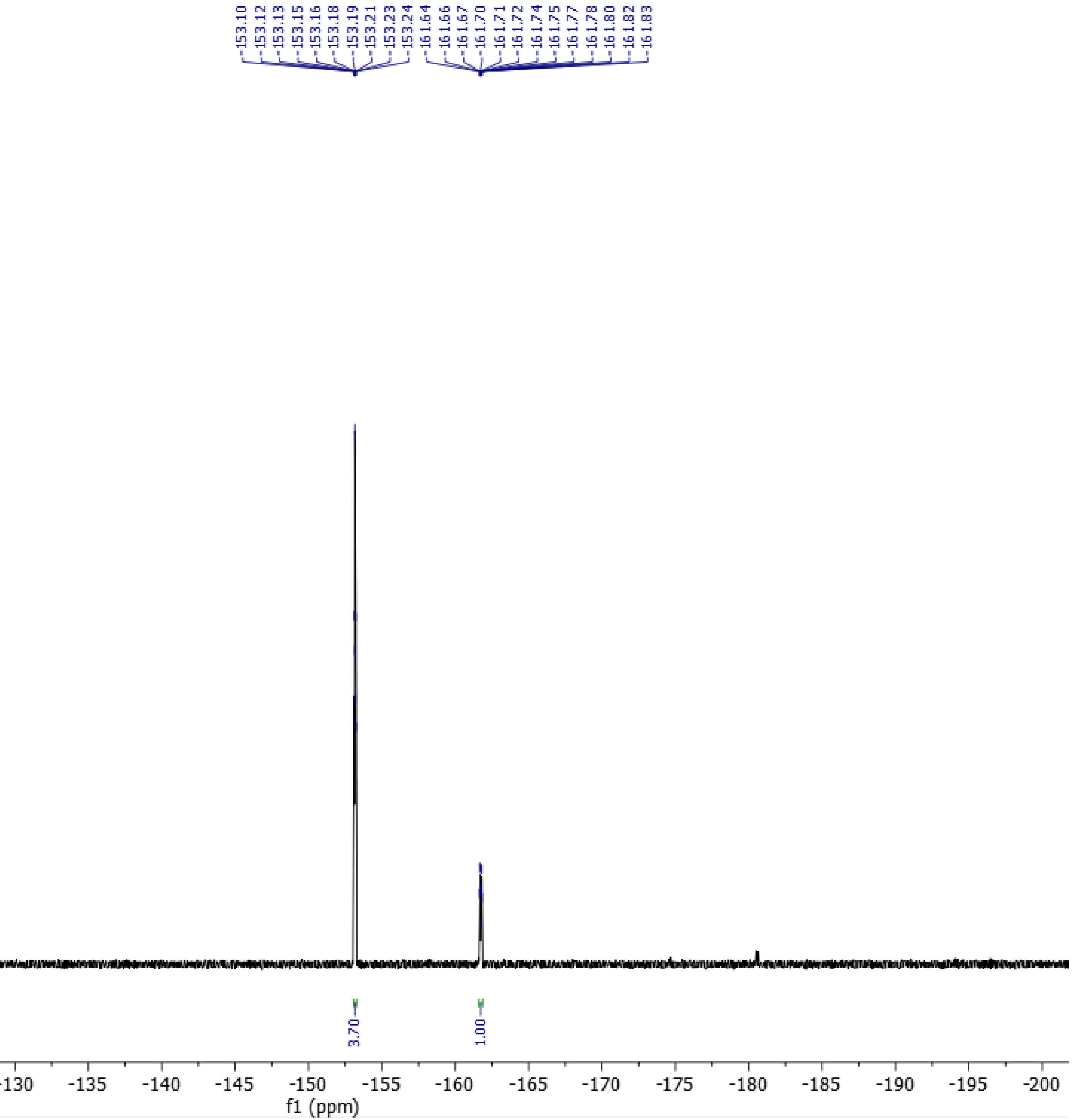

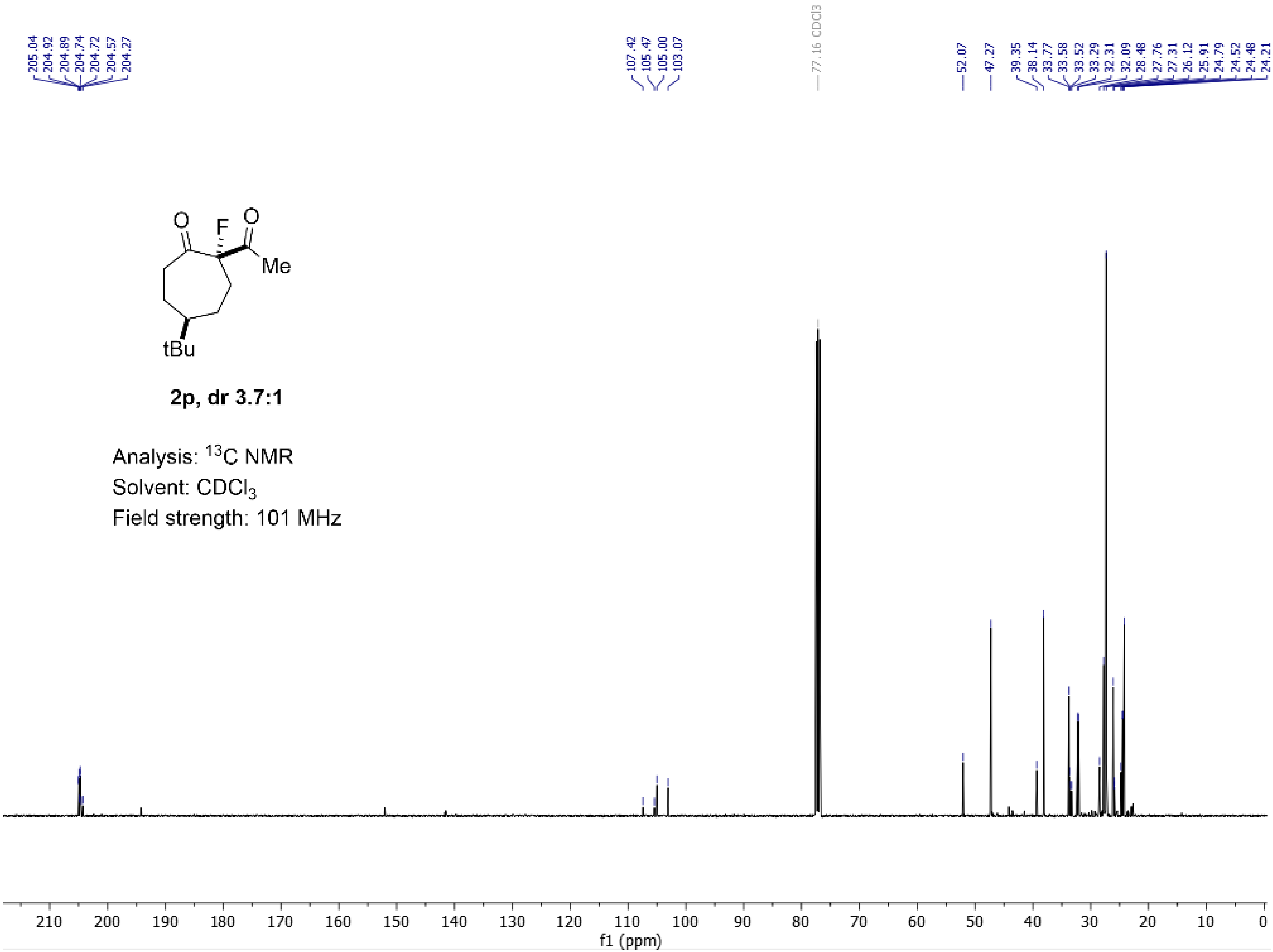

2p, dr 3.7:1

Analysis: ^{13}C NMR
Solvent: $CDCl_3$
Field strength: 101 MHz

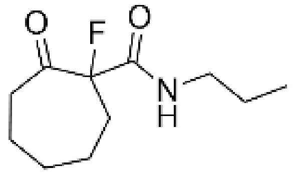

2q

Analysis: ^{1}H NMR
Solvent: $CDCl_3$
Field strength: 400 MHz

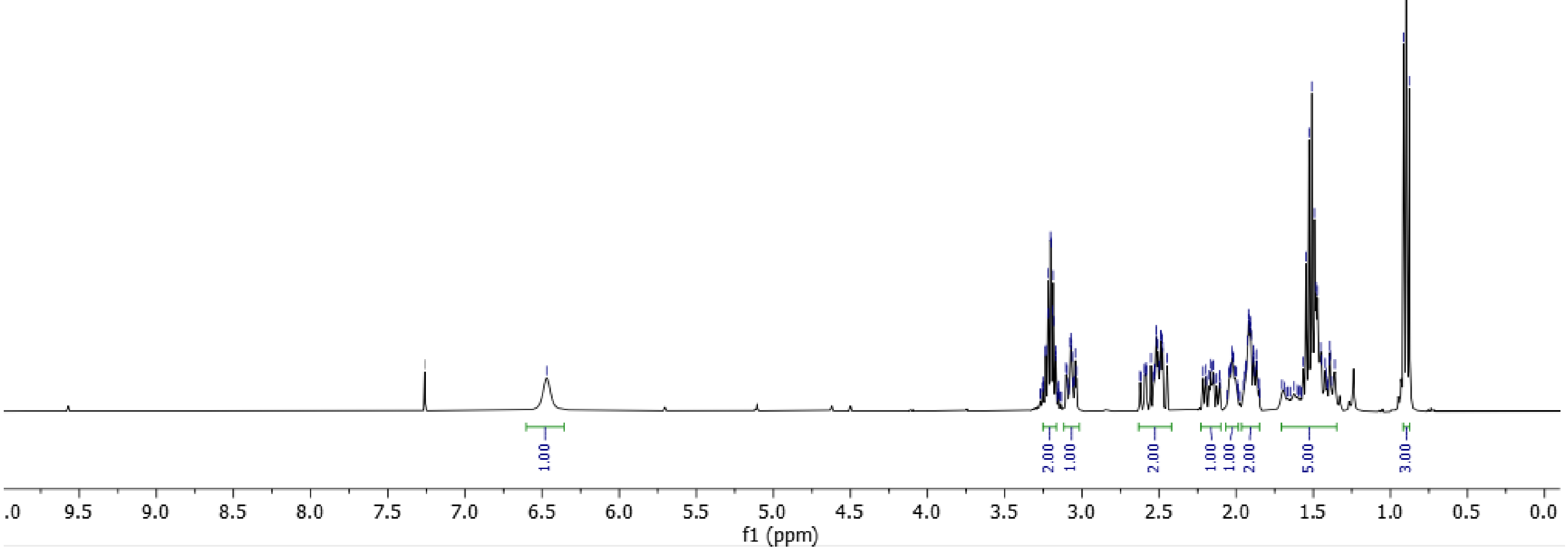

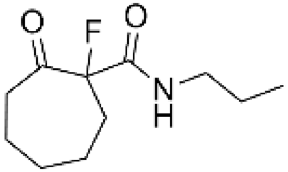

2q

Analysis: ^{19}F NMR
Solvent: $CDCl_3$
Field strength: 377 MHz

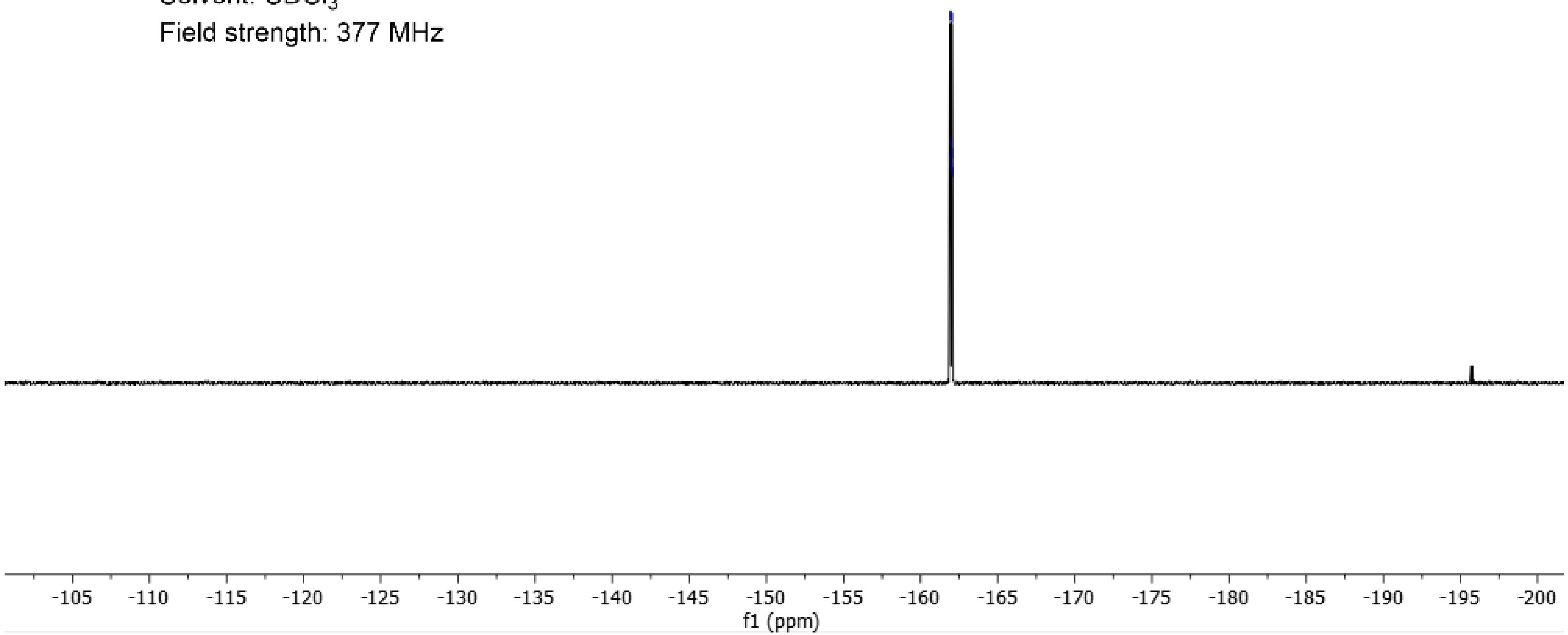

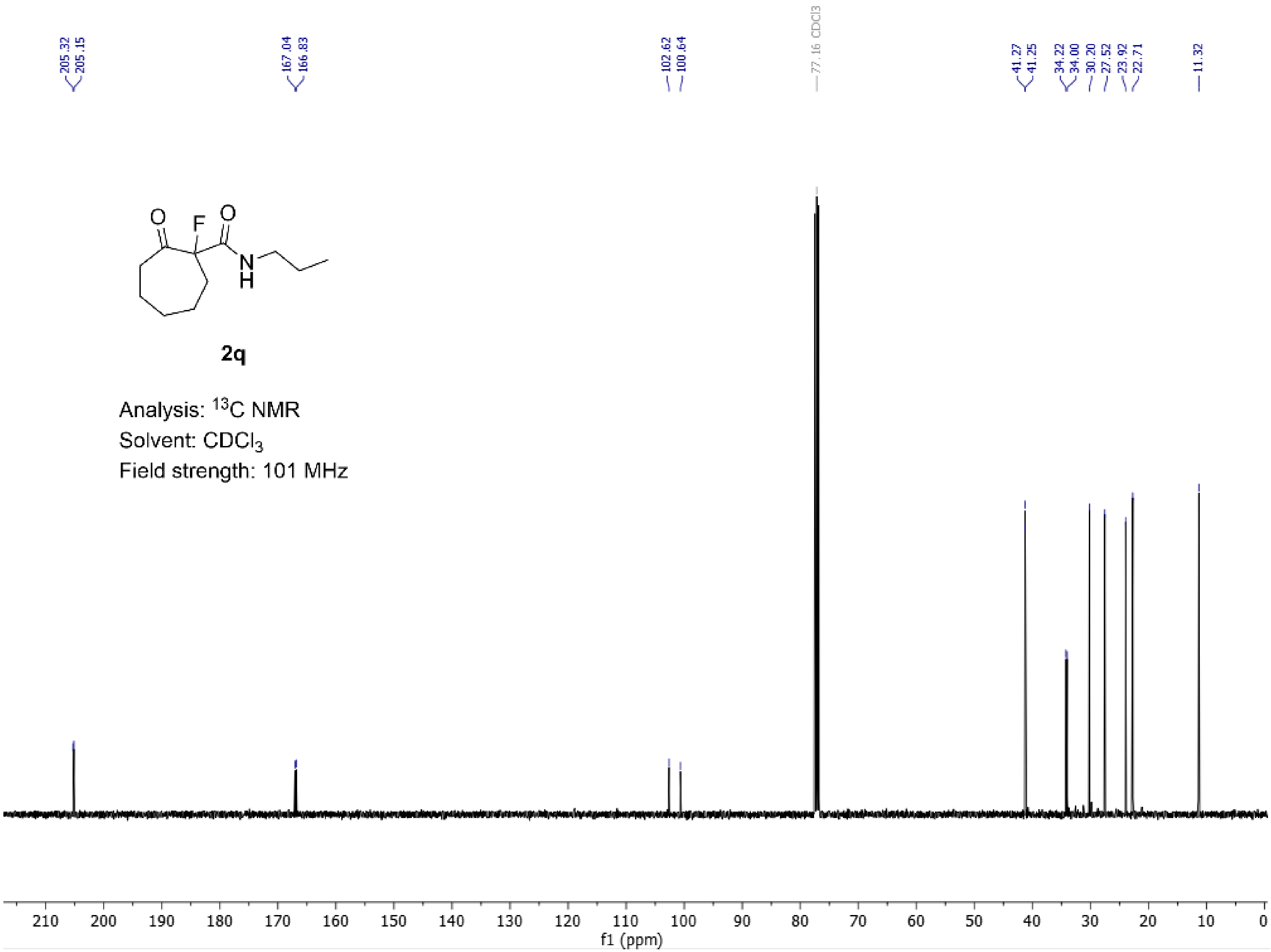

205.32
205.15
167.04
166.83
102.62
100.64
77.16 CDCl3
41.27
41.25
34.22
34.00
30.20
27.52
23.92
22.71
11.32
2q
Analysis: 13C NMR
Solvent: CDCl3
Field strength: 101 MHz
210 200 190 180 170 160 150 140 130 120 110 100 90 80 70 60 50 40 30 20 10 0
f1 (ppm)

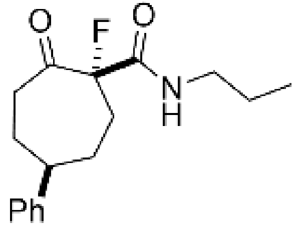

2r, dr 5.3:1

Analysis: ^{1}H NMR
Solvent: $CDCl_3$
Field strength: 400 MHz

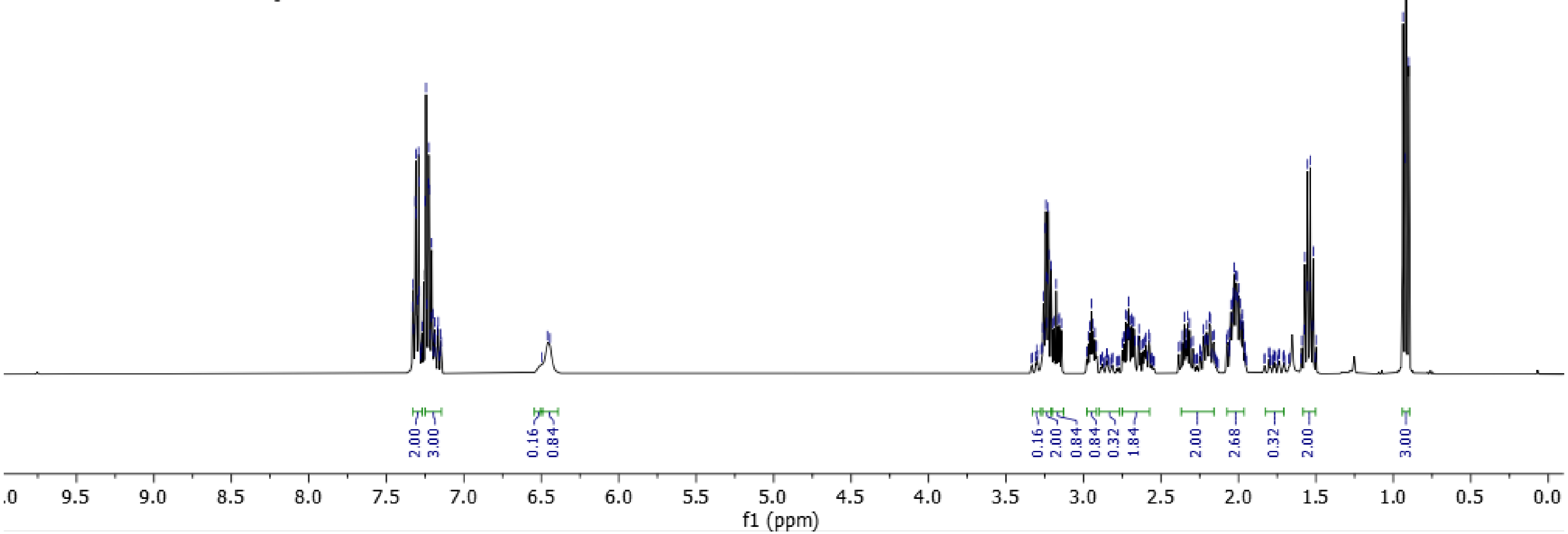

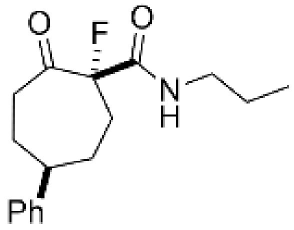

2r, dr 5.3:1

Analysis: ^{19}F NMR
Solvent: $CDCl_3$
Field strength: 377 MHz

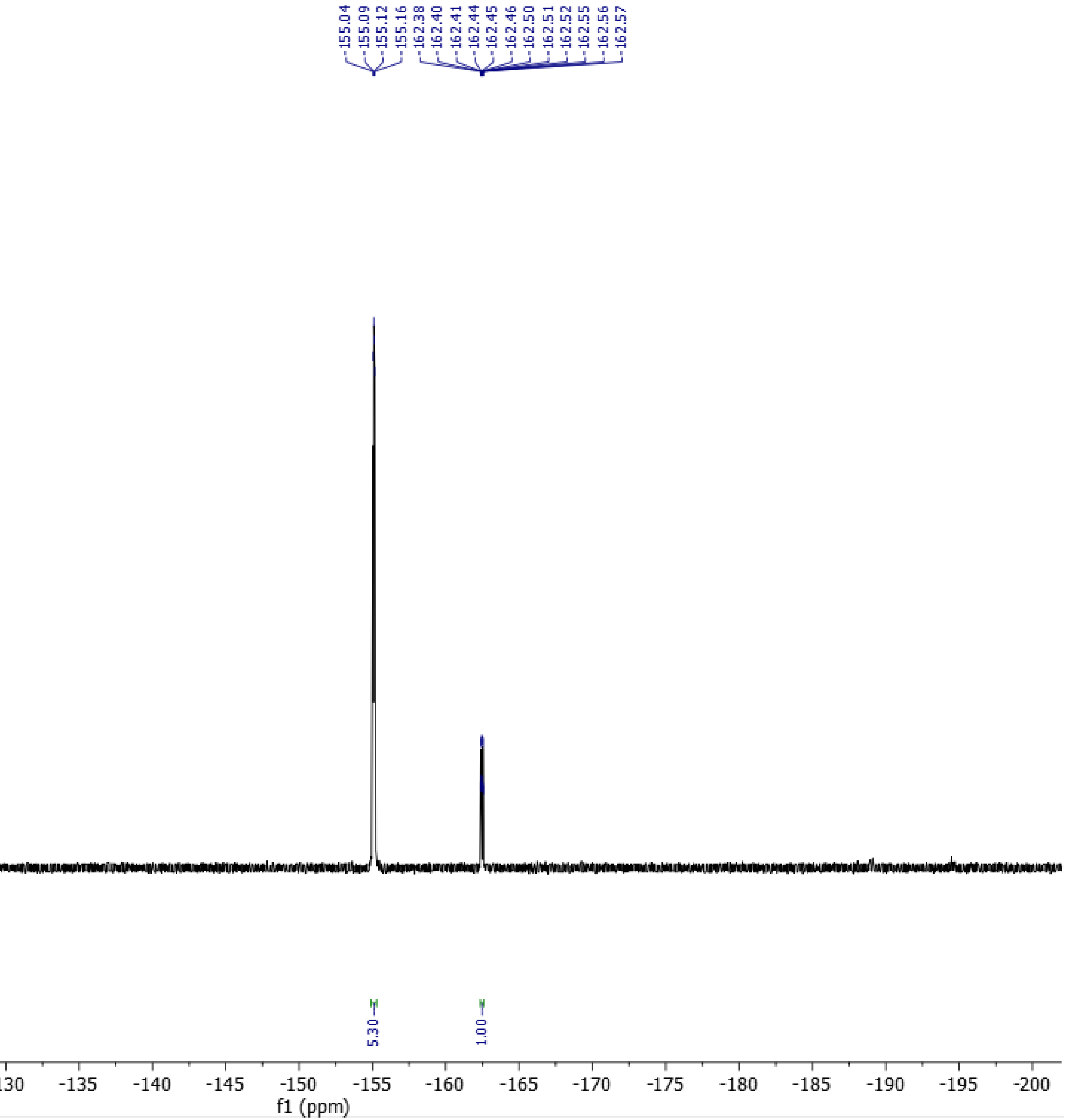

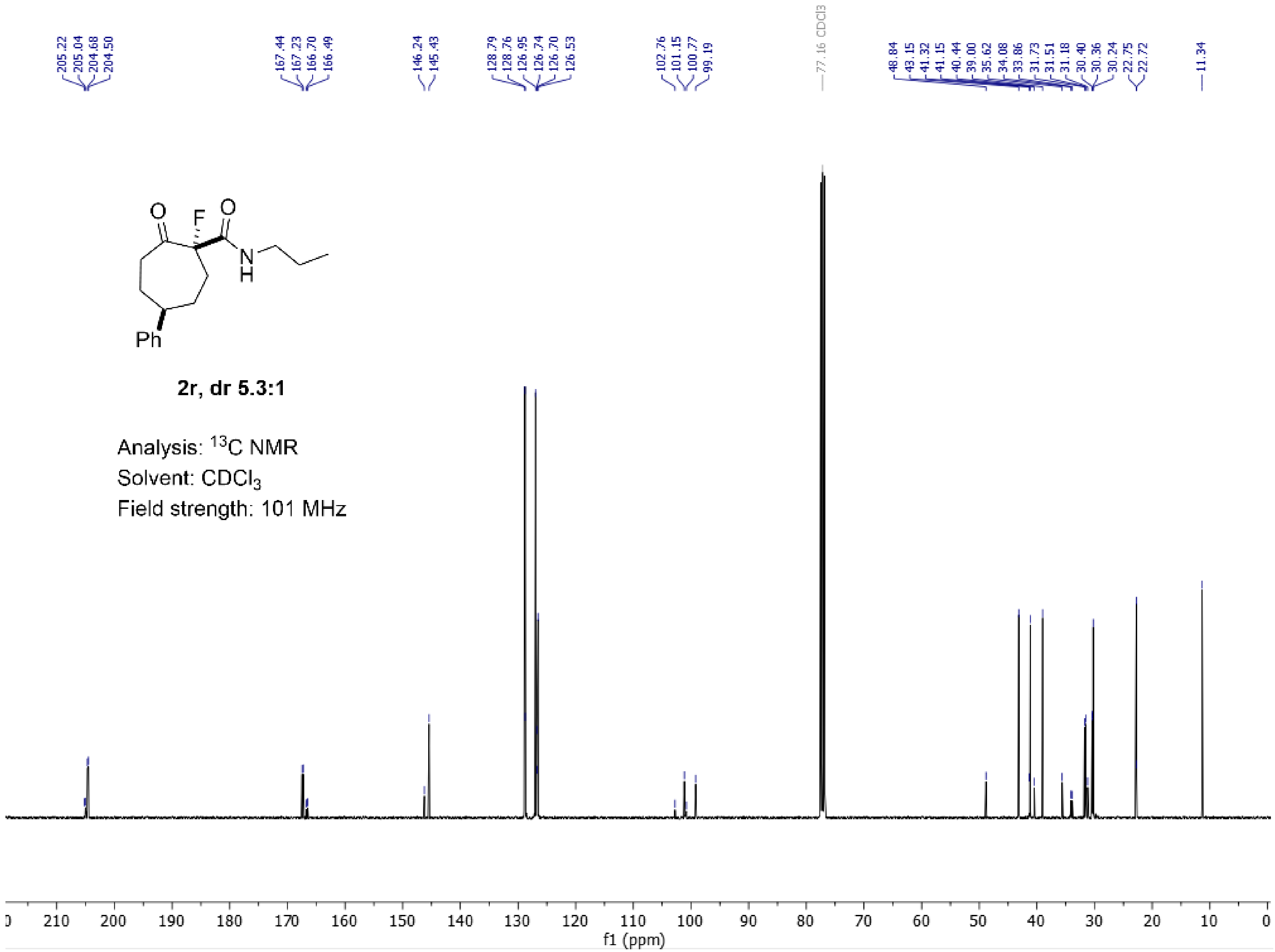
205.22
205.04
204.68
204.50
167.44
167.23
166.70
166.49
146.24
145.43
128.79
128.76
126.95
126.74
126.70
126.53
102.76
101.15
100.77
99.19
77.16 CDCl3
48.84
43.15
41.32
41.15
40.44
39.00
35.62
34.08
33.86
31.73
31.51
31.18
30.40
30.36
30.24
22.75
22.72
11.34
O
F
O
N
H
Ph
2r, dr 5.3:1
Analysis: 13C NMR
Solvent: CDCl3
Field strength: 101 MHz
210
200
190
180
170
160
150
140
130
120
110
100
90
80
70
60
50
40
30
20
10
0
f1 (ppm)

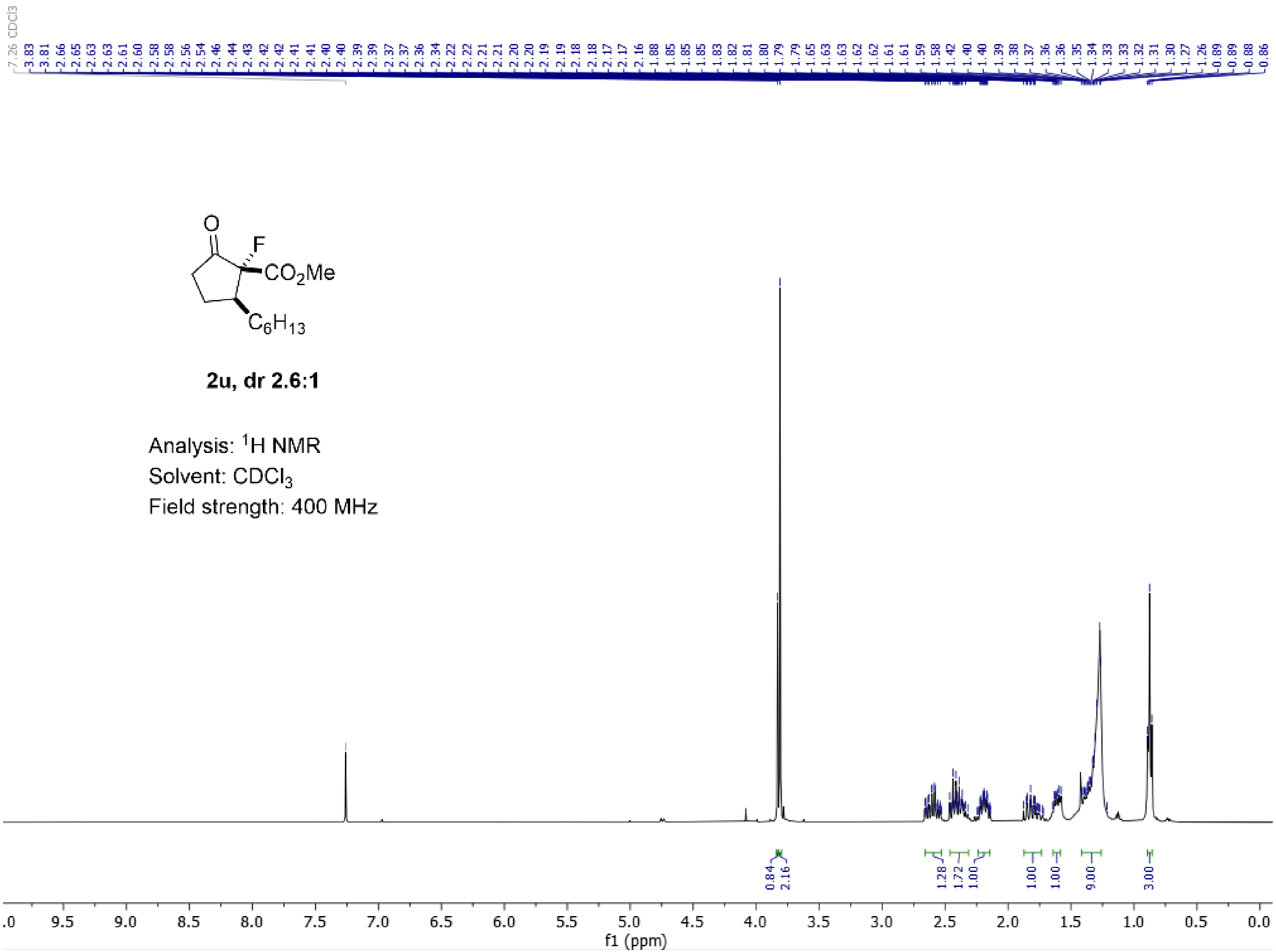
7.26 CDCl3
3.83
3.81
2.66
2.65
2.63
2.63
2.61
2.60
2.58
2.58
2.56
2.54
2.46
2.44
2.43
2.42
2.42
2.41
2.41
2.40
2.40
2.39
2.39
2.37
2.37
2.36
2.34
2.22
2.22
2.21
2.21
2.20
2.20
2.19
2.19
2.18
2.18
2.17
2.17
2.16
1.88
1.85
1.85
1.85
1.83
1.82
1.81
1.80
1.79
1.79
1.65
1.63
1.63
1.62
1.62
1.61
1.61
1.59
1.58
1.42
1.40
1.40
1.39
1.38
1.37
1.36
1.36
1.35
1.34
1.33
1.33
1.32
1.31
1.30
1.27
1.26
0.89
0.89
0.88
0.86
O
F
CO2Me
C6H13
2u, dr 2.6:1
Analysis: 1H NMR
Solvent: CDCl3
Field strength: 400 MHz
0.84
2.16
1.28
1.72
1.00
1.00
1.00
9.00
3.00
.0 9.5 9.0 8.5 8.0 7.5 7.0 6.5 6.0 5.5 5.0 4.5 4.0 3.5 3.0 2.5 2.0 1.5 1.0 0.5 0.0
f1 (ppm)

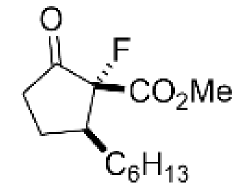

2u, dr 2.6:1

Analysis: ^{19}F NMR
Solvent: $CDCl_3$
Field strength: 377 MHz

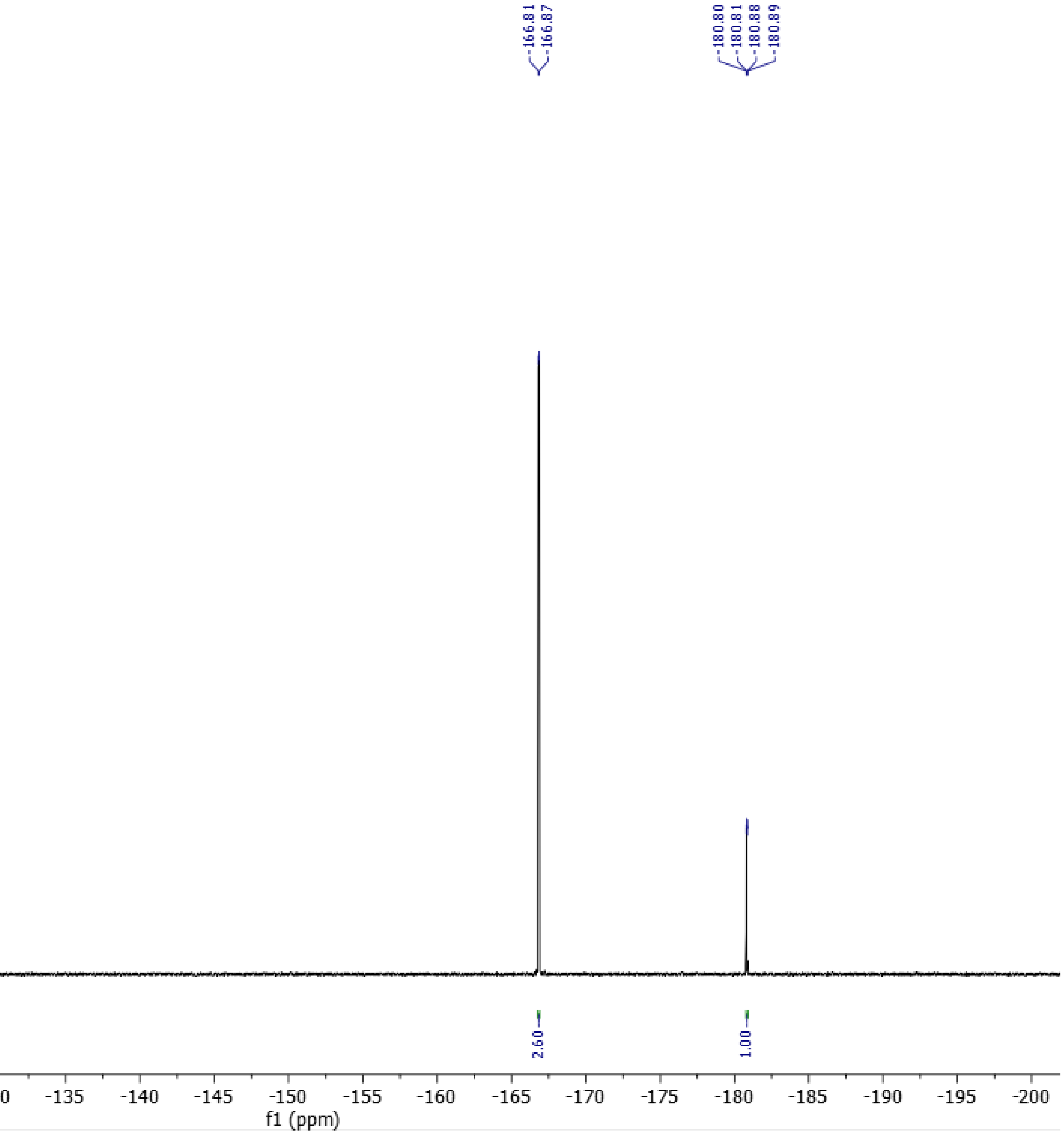

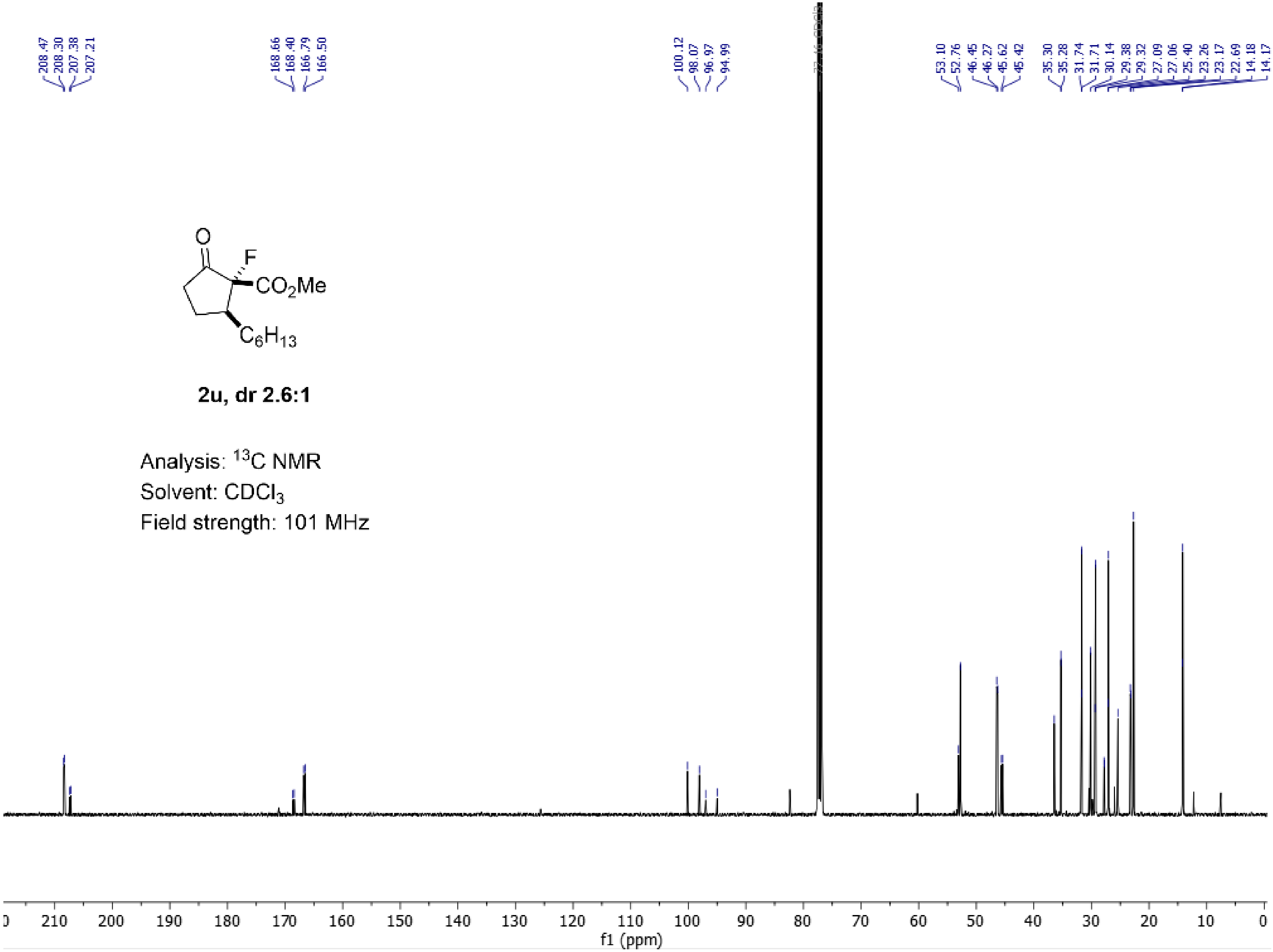

2u, dr 2.6:1

Analysis: ^{13}C NMR
Solvent: $CDCl_3$
Field strength: 101 MHz

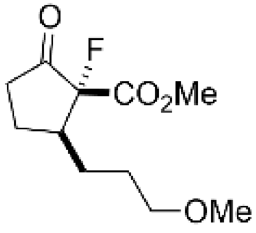

2v, dr 2:1

Analysis: ^{1}H NMR
Solvent: $CDCl_3$
Field strength: 500 MHz

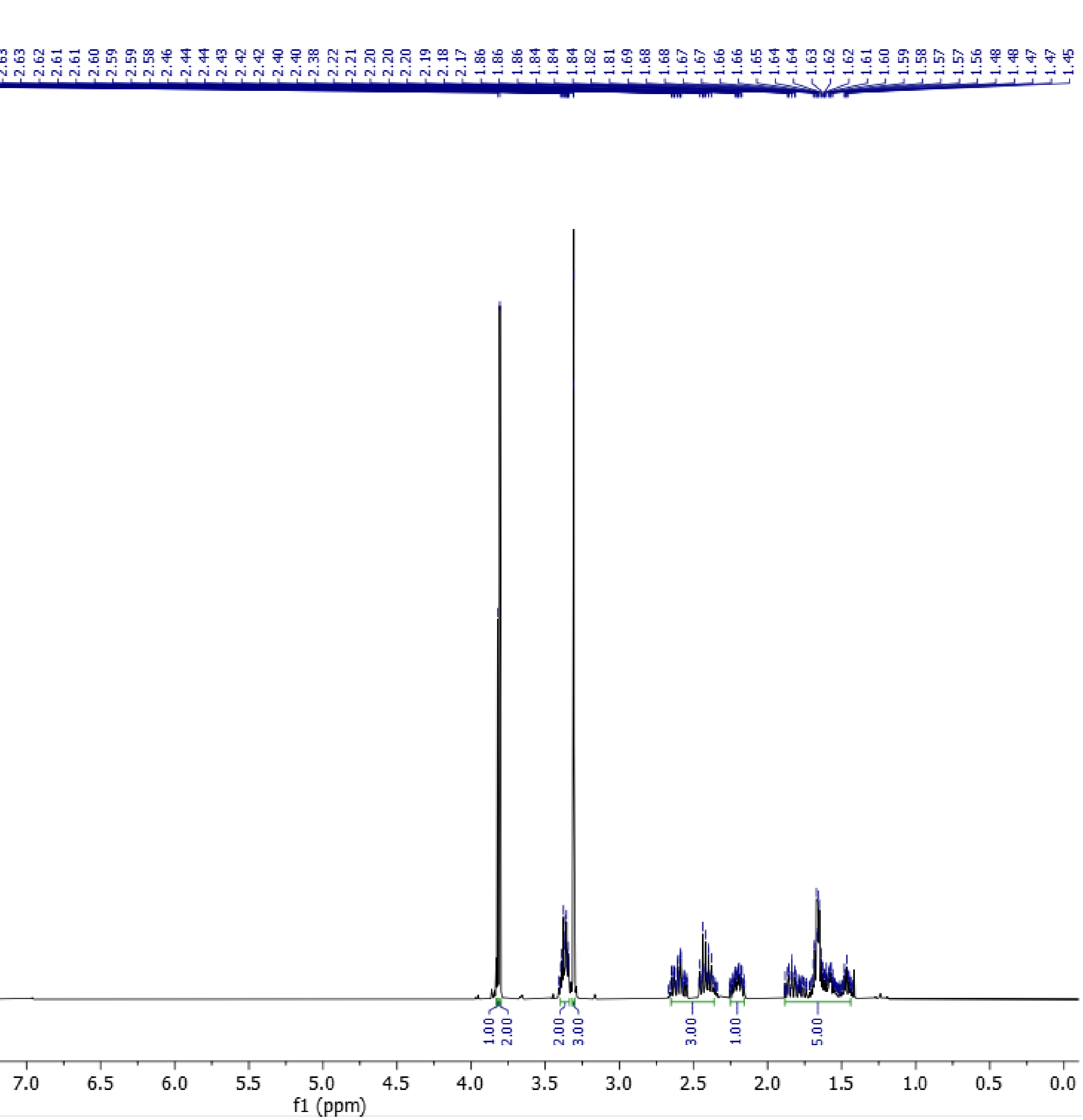

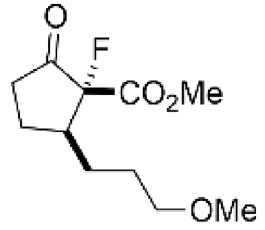

2v, dr 2:1

Analysis: ^{19}F NMR
Solvent: $CDCl_3$
Field strength: 377 MHz

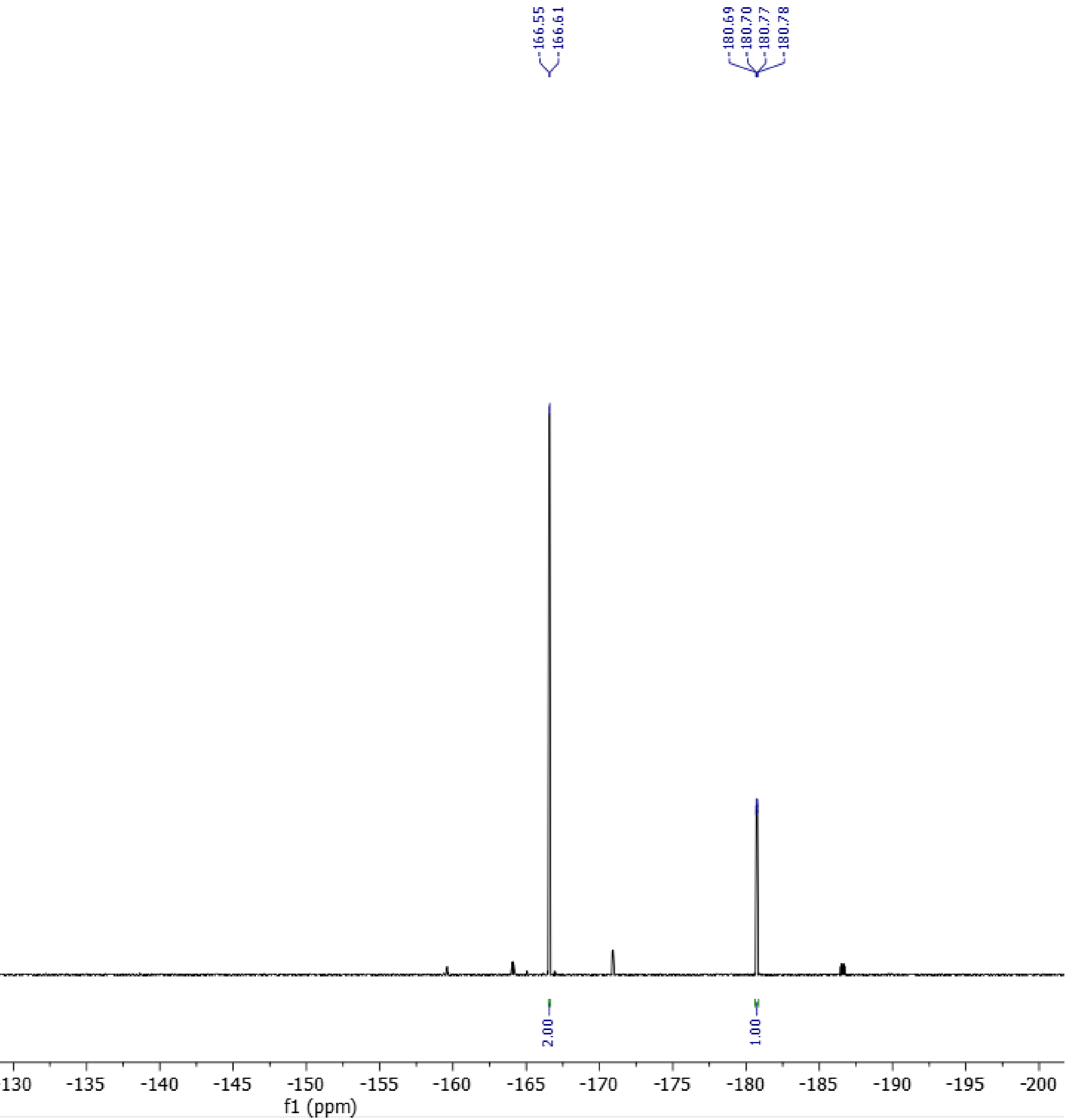

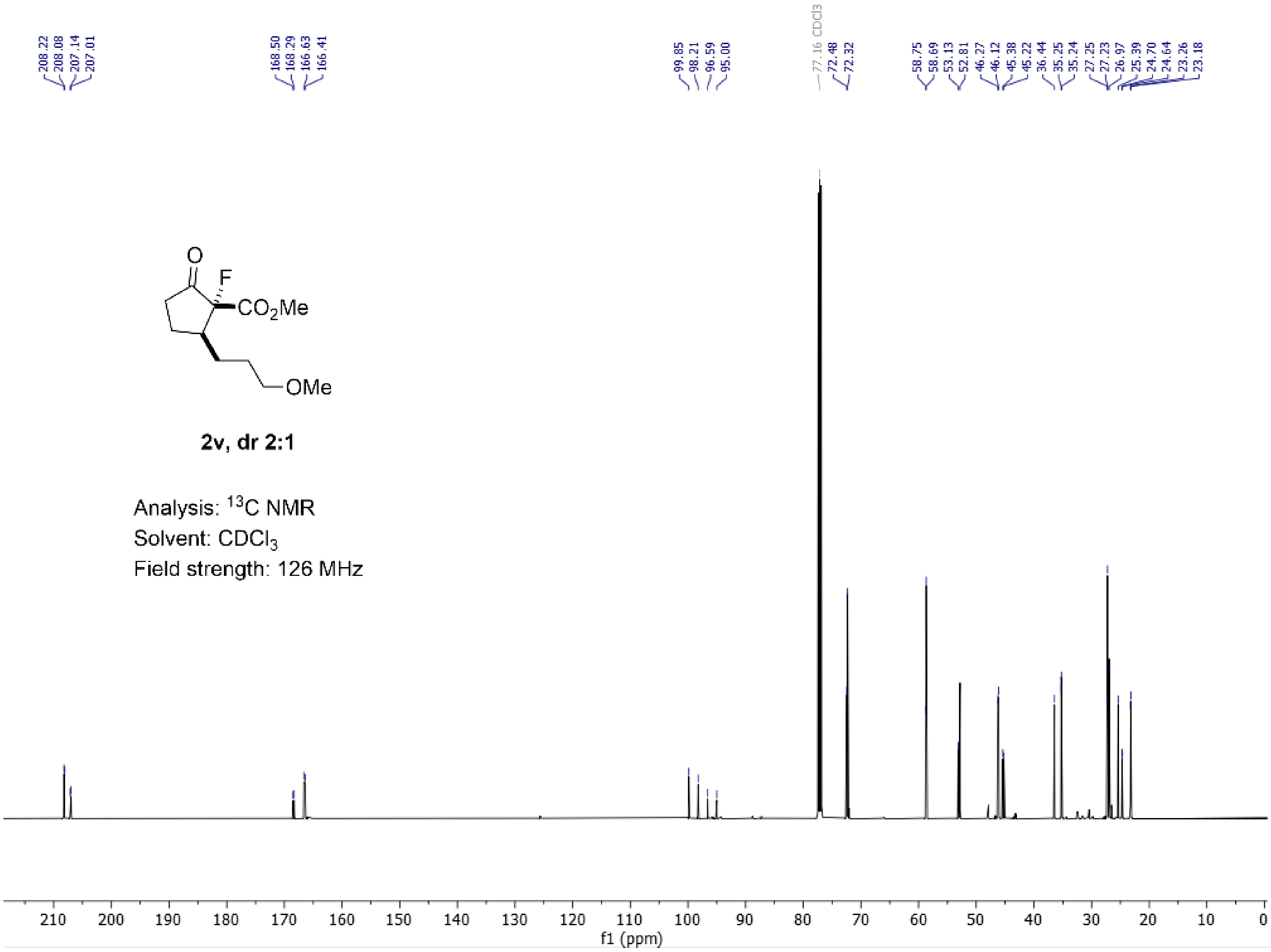

2v, dr 2:1

Analysis: ^{13}C NMR
Solvent: $CDCl_3$
Field strength: 126 MHz

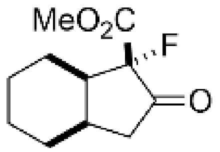

2w, dr 9.5:1

Analysis: ^{1}H NMR
Solvent: $CDCl_3$
Field strength: 400 MHz

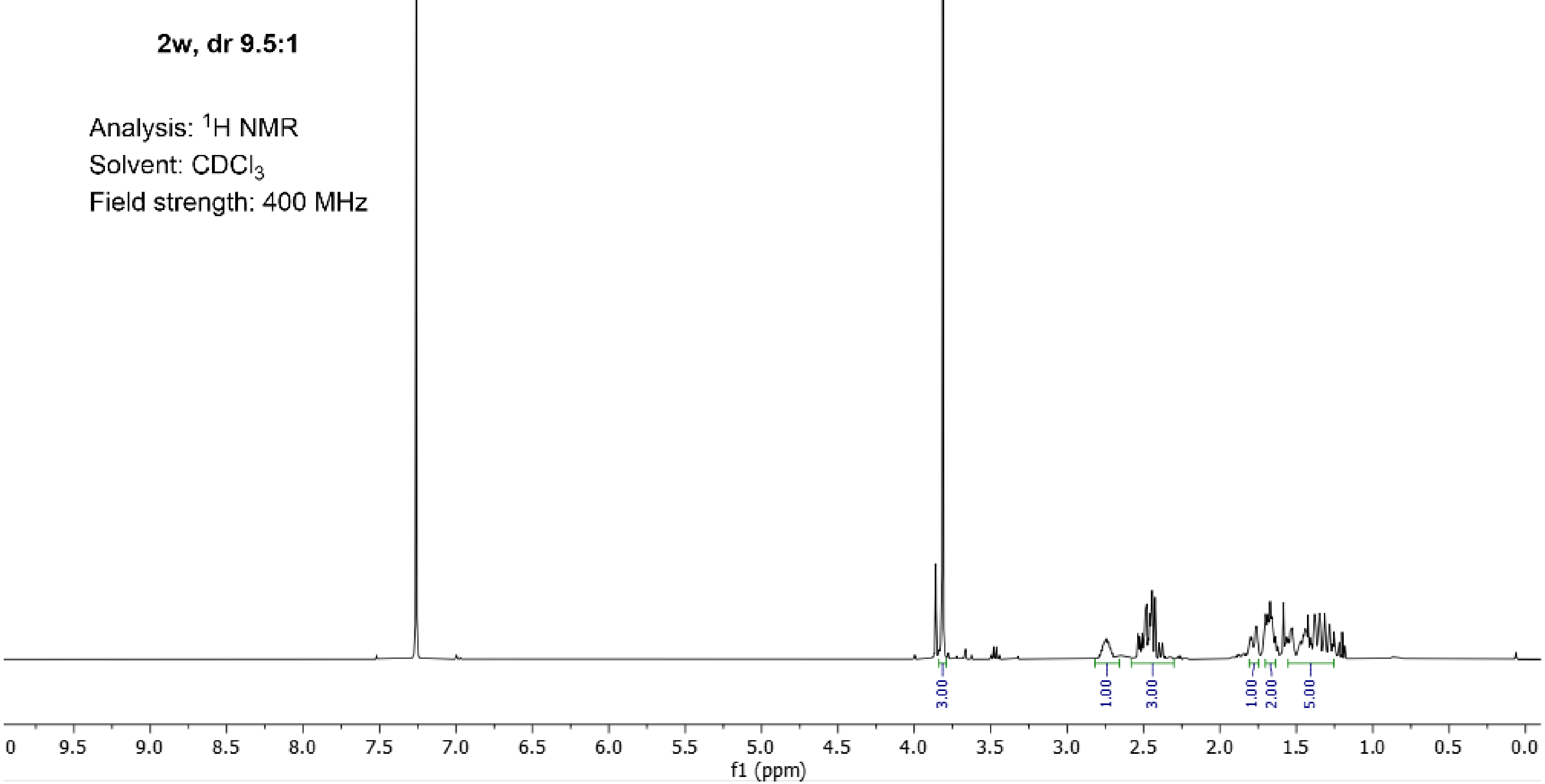

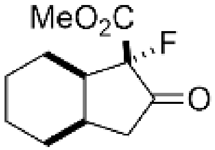

2w, dr 9.5:1

Analysis: ^{19}F NMR
Solvent: $CDCl_3$
Field strength: 377 MHz

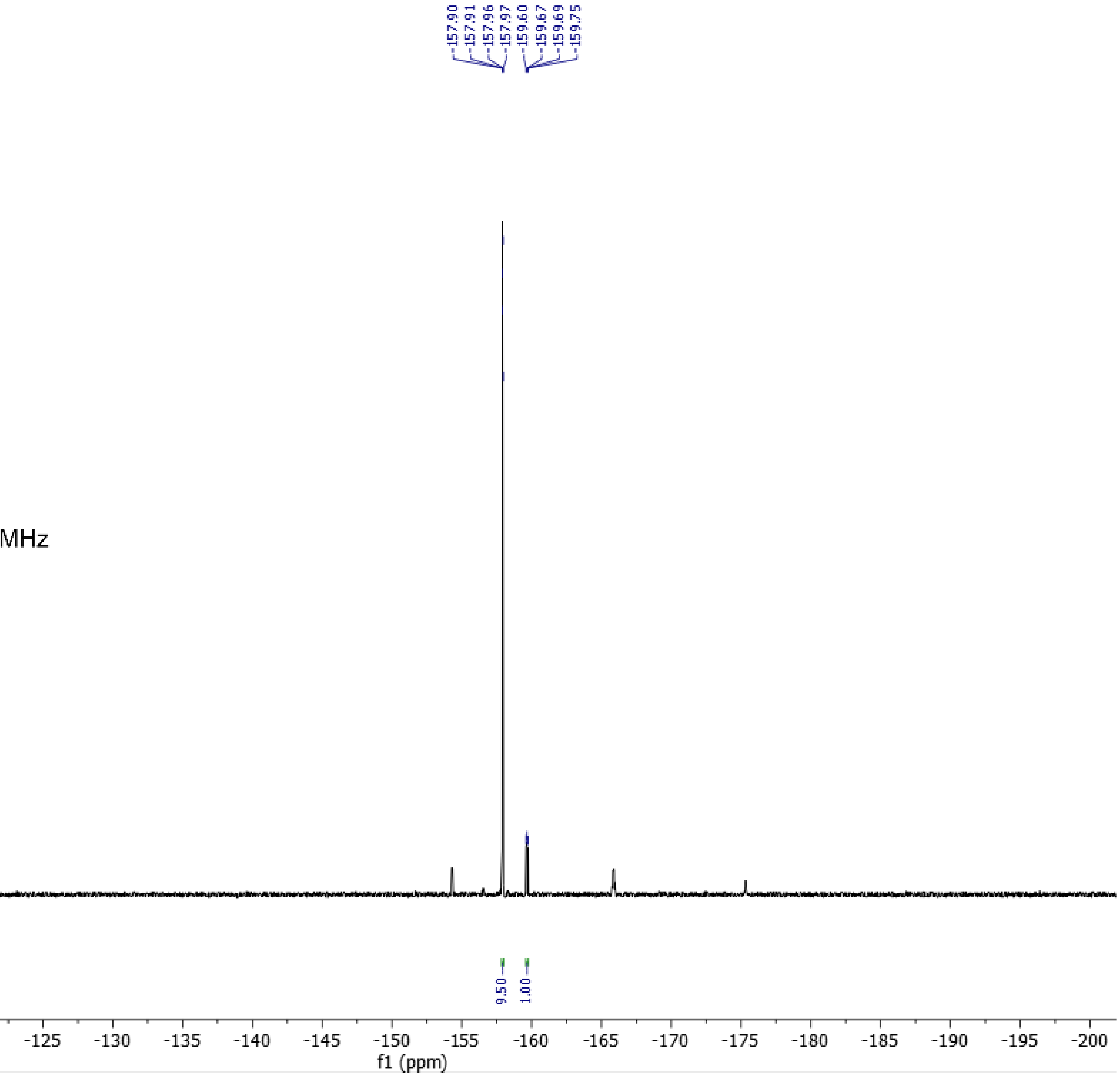

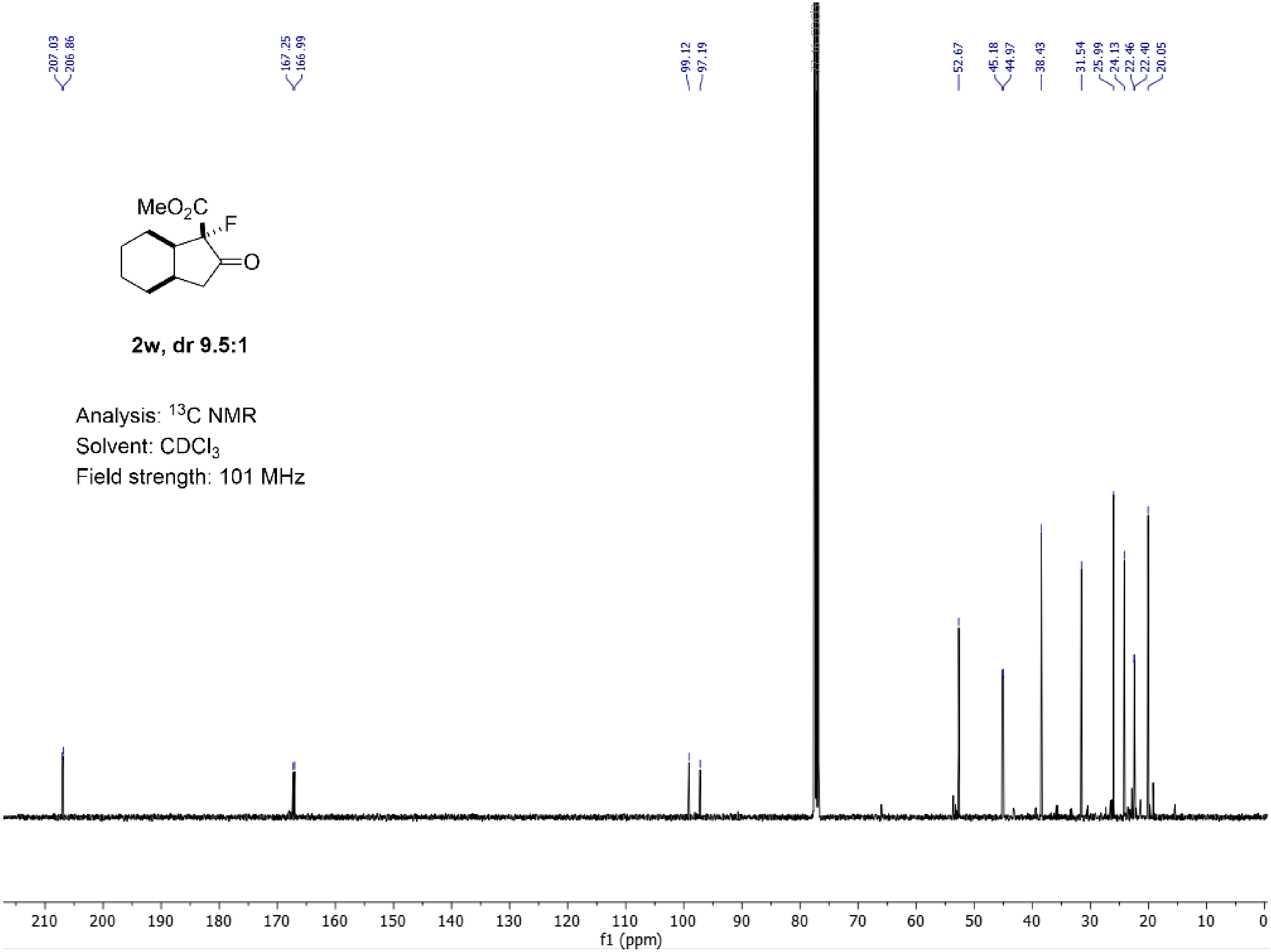
207.03
206.86
167.25
166.99
99.12
97.19
52.67
45.18
44.97
38.43
31.54
25.99
24.13
22.46
22.40
20.05
MeO_2C
F
O
2w, dr 9.5:1
Analysis: ^{13}C NMR
Solvent: $CDCl_3$
Field strength: 101 MHz
210 200 190 180 170 160 150 140 130 120 110 100 90 80 70 60 50 40 30 20 10 0
f1 (ppm)

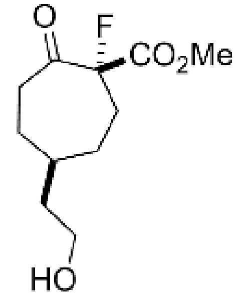

2x, dr 3:1

Analysis: ^{1}H NMR
Solvent: $CDCl_3$
Field strength: 400 MHz

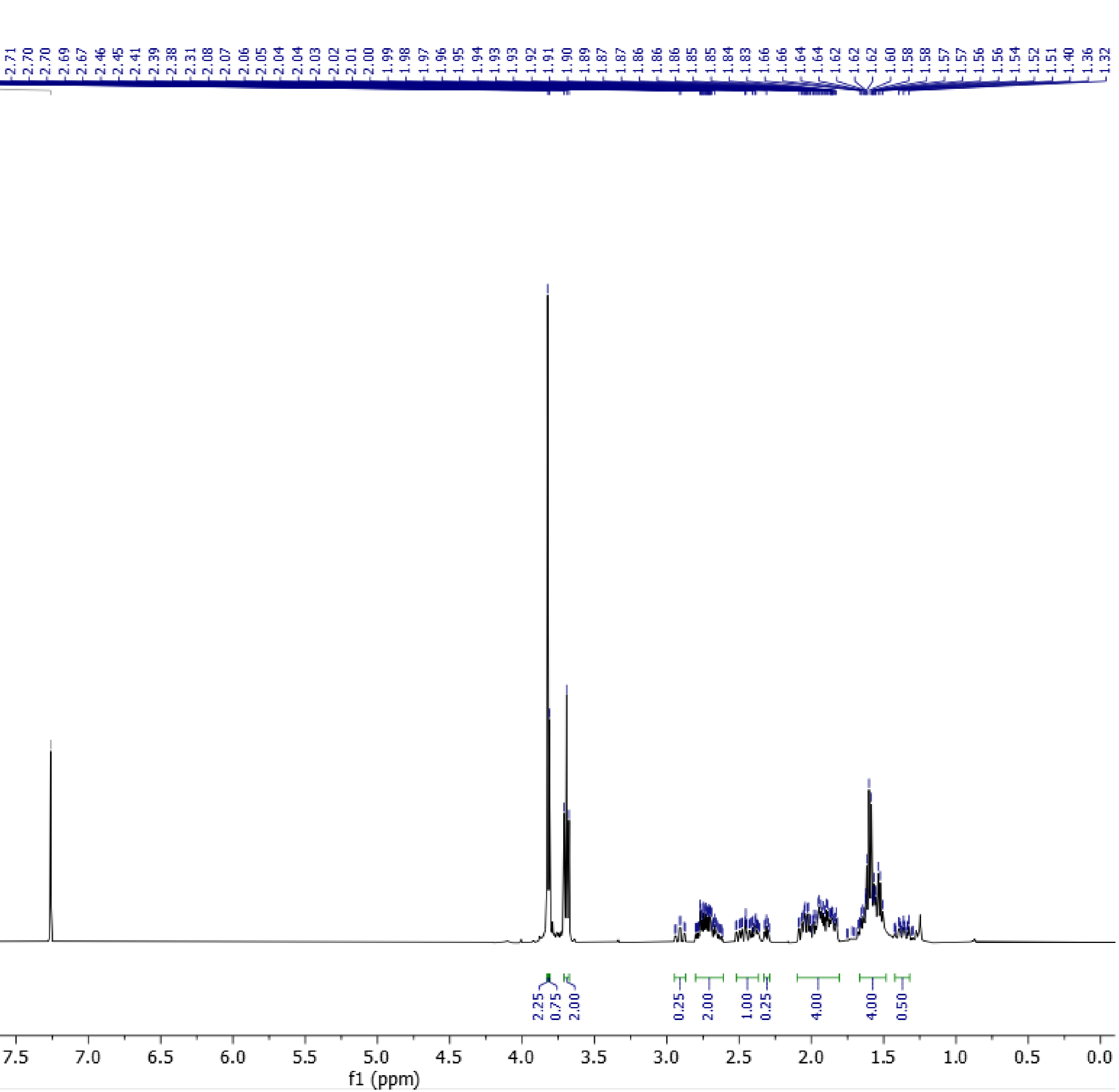

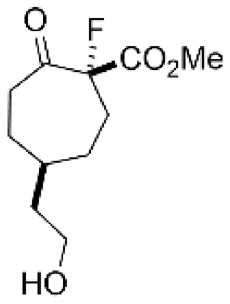

2x, dr 3:1

Analysis: ^{19}F NMR
Solvent: $CDCl_3$
Field strength: 377 MHz

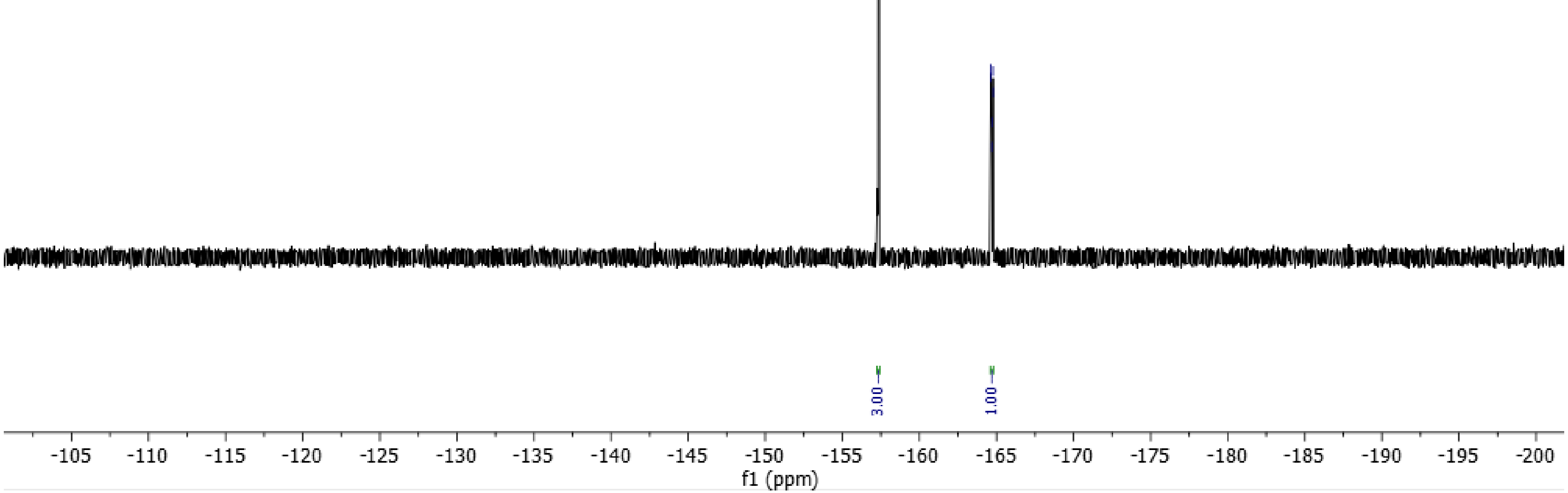

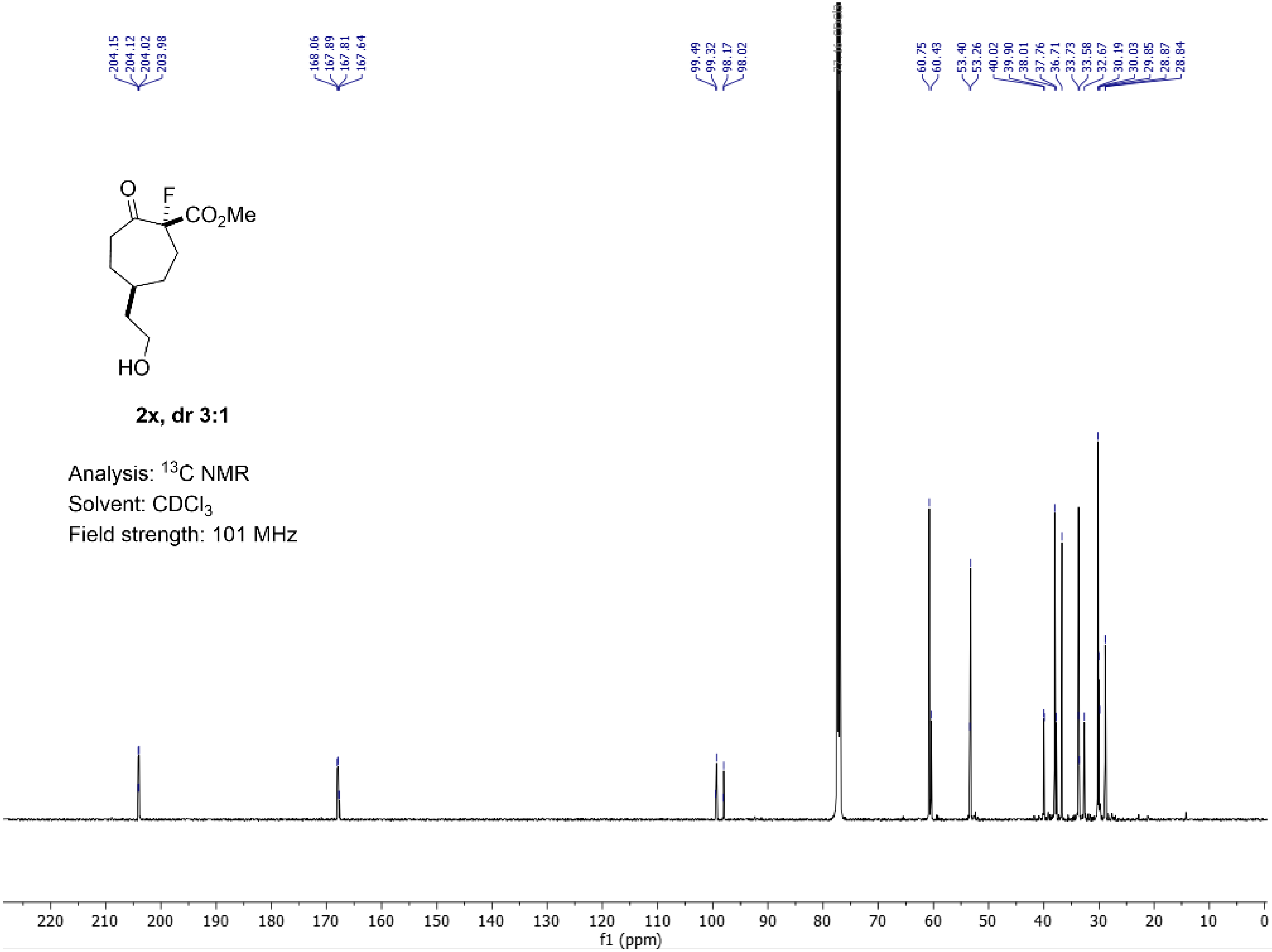

2x, dr 3:1

Analysis: ^{13}C NMR
Solvent: $CDCl_3$
Field strength: 101 MHz

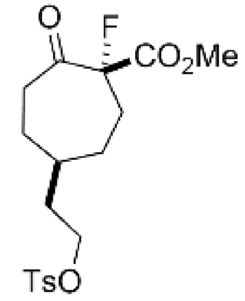

2y, dr 3.5:1

Analysis: ^{1}H NMR
Solvent: $CDCl_3$
Field strength: 400 MHz

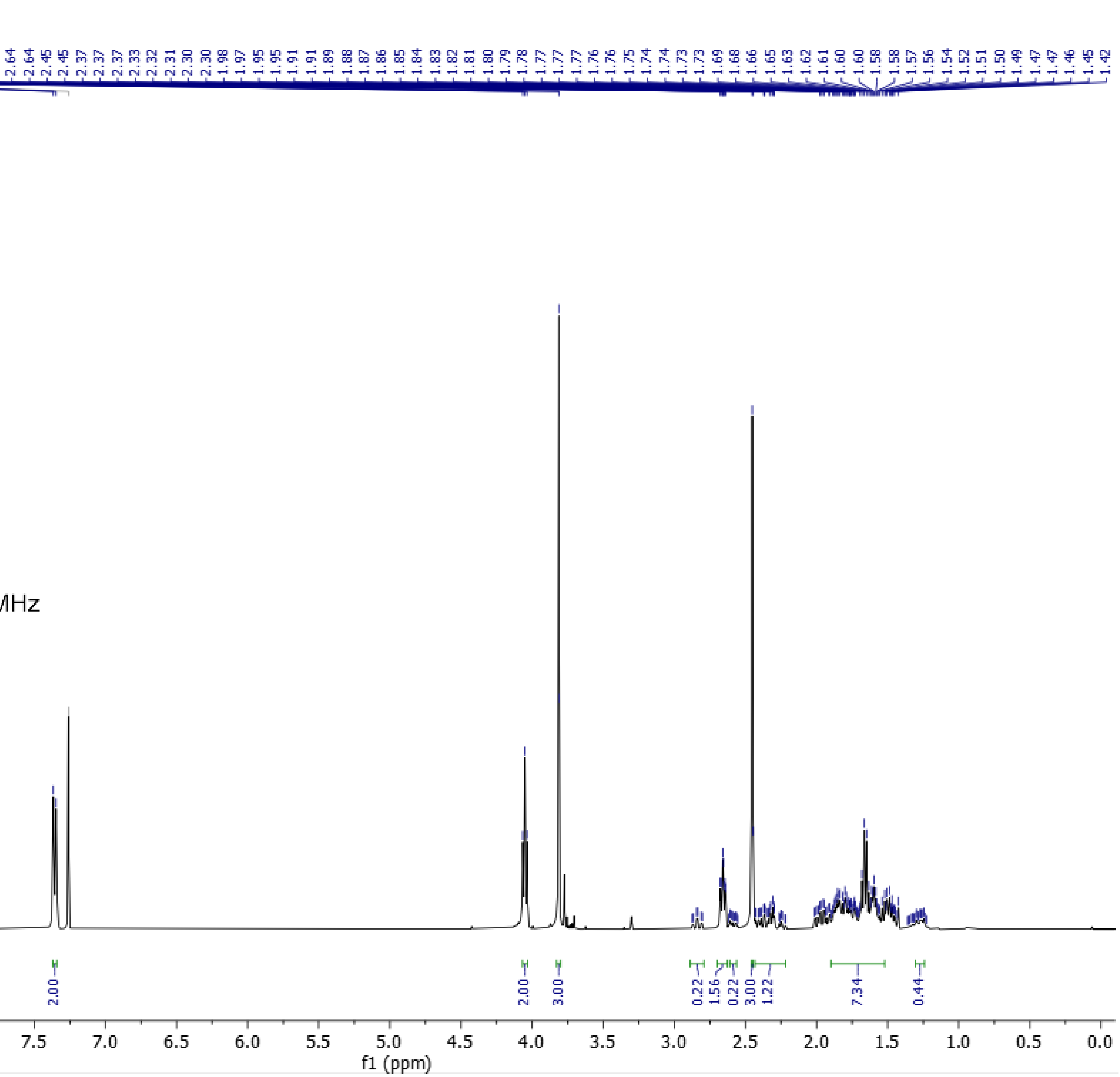

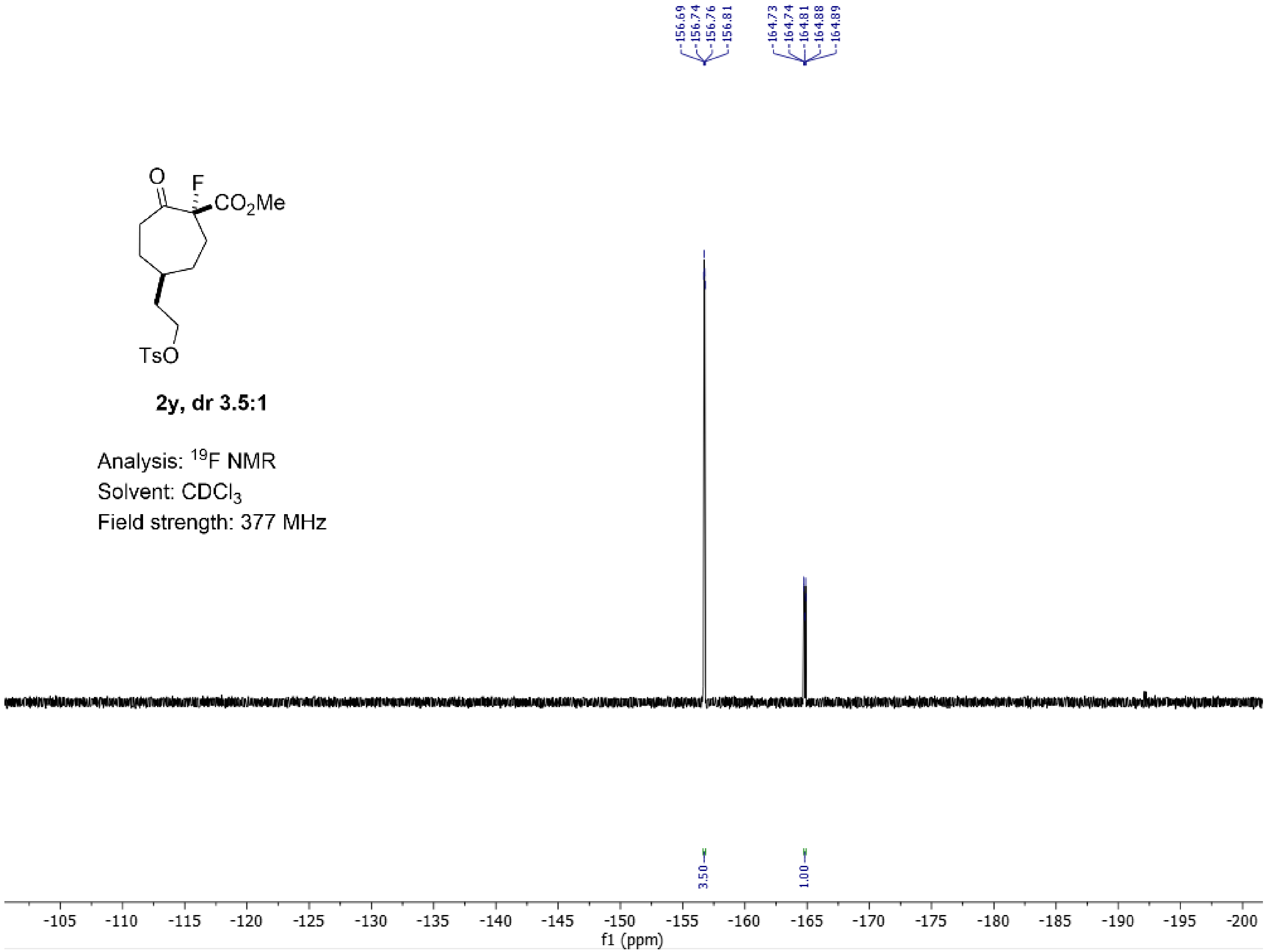

2y, dr 3.5:1

Analysis: ^{19}F NMR
Solvent: $CDCl_3$
Field strength: 377 MHz

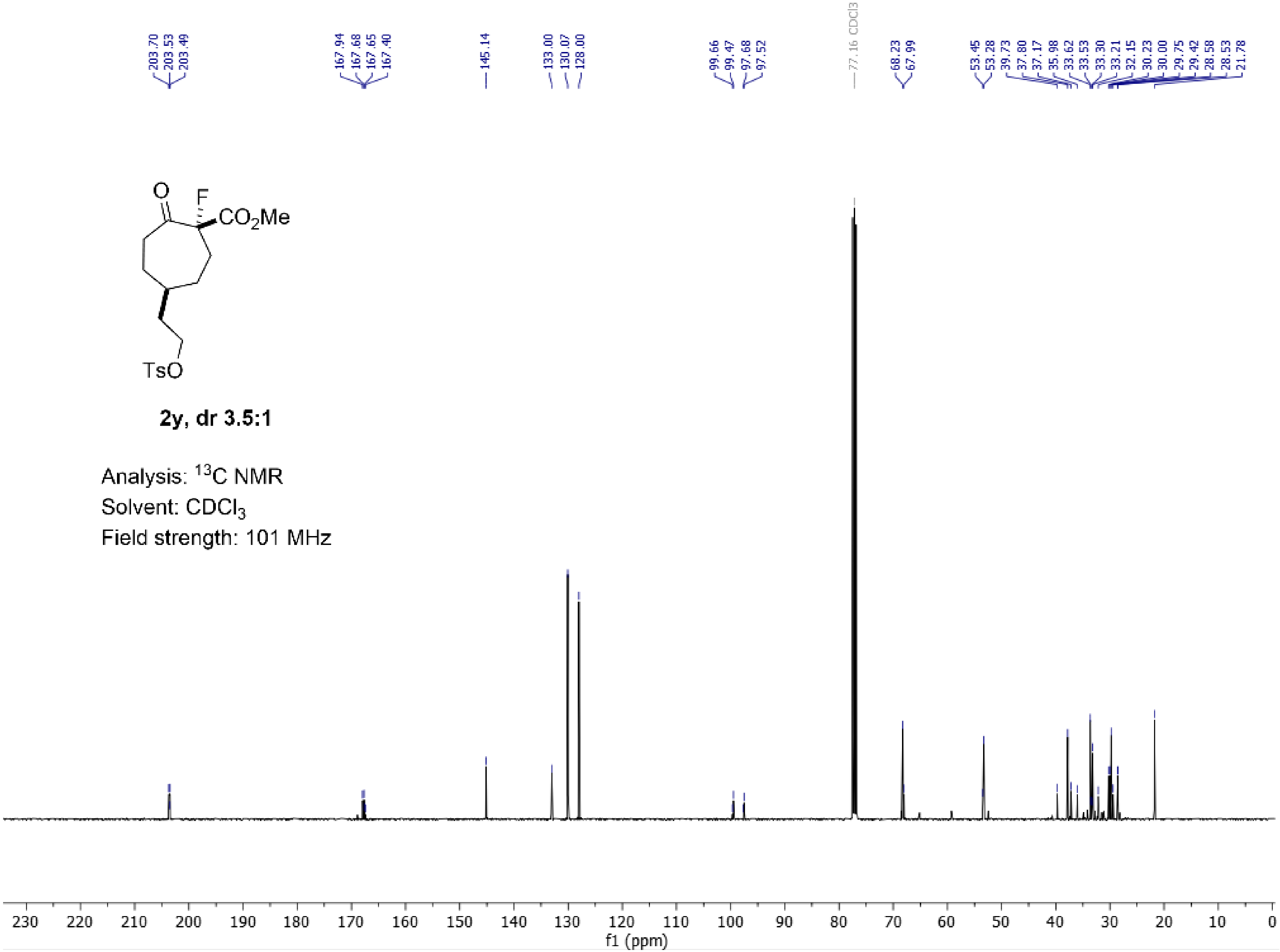

2y, dr 3.5:1

Analysis: ^{13}C NMR
Solvent: $CDCl_3$
Field strength: 101 MHz

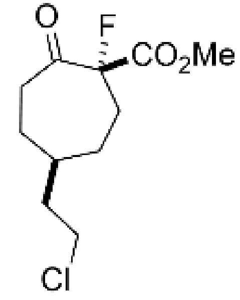

2z, dr 3.1:1

Analysis: ^{1}H NMR
Solvent: $CDCl_3$
Field strength: 400 MHz

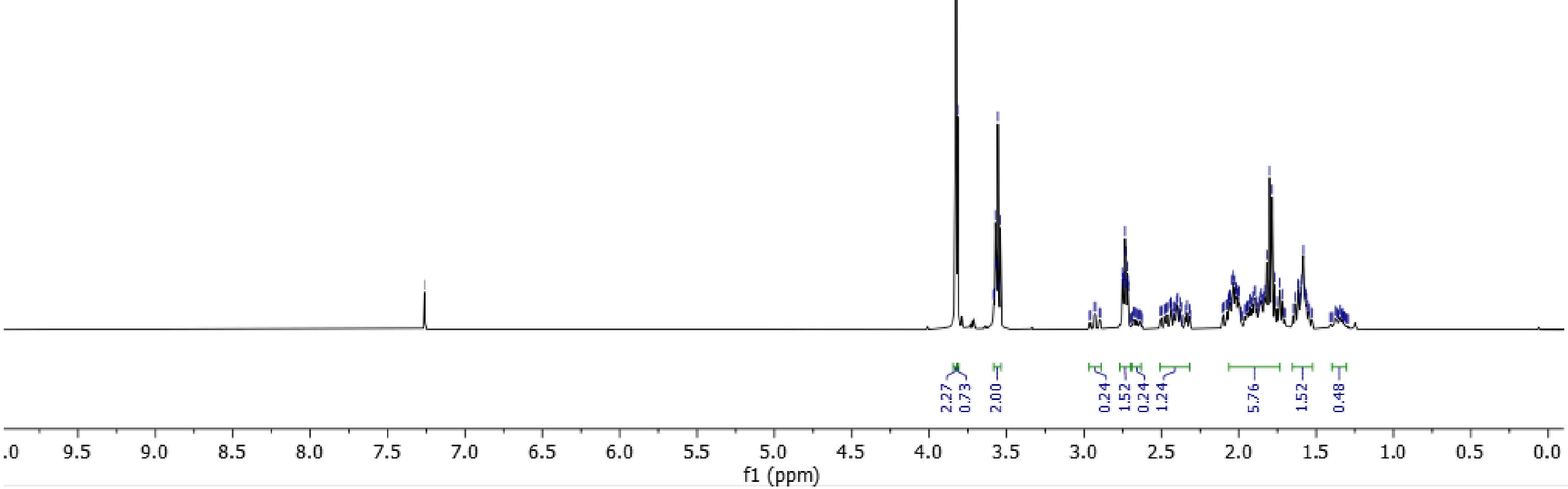

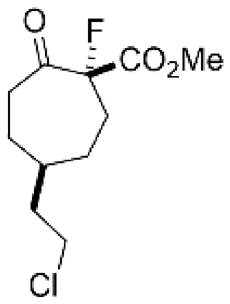

2z, dr 3.1:1

Analysis: ^{19}F NMR
Solvent: $CDCl_3$
Field strength: 377 MHz

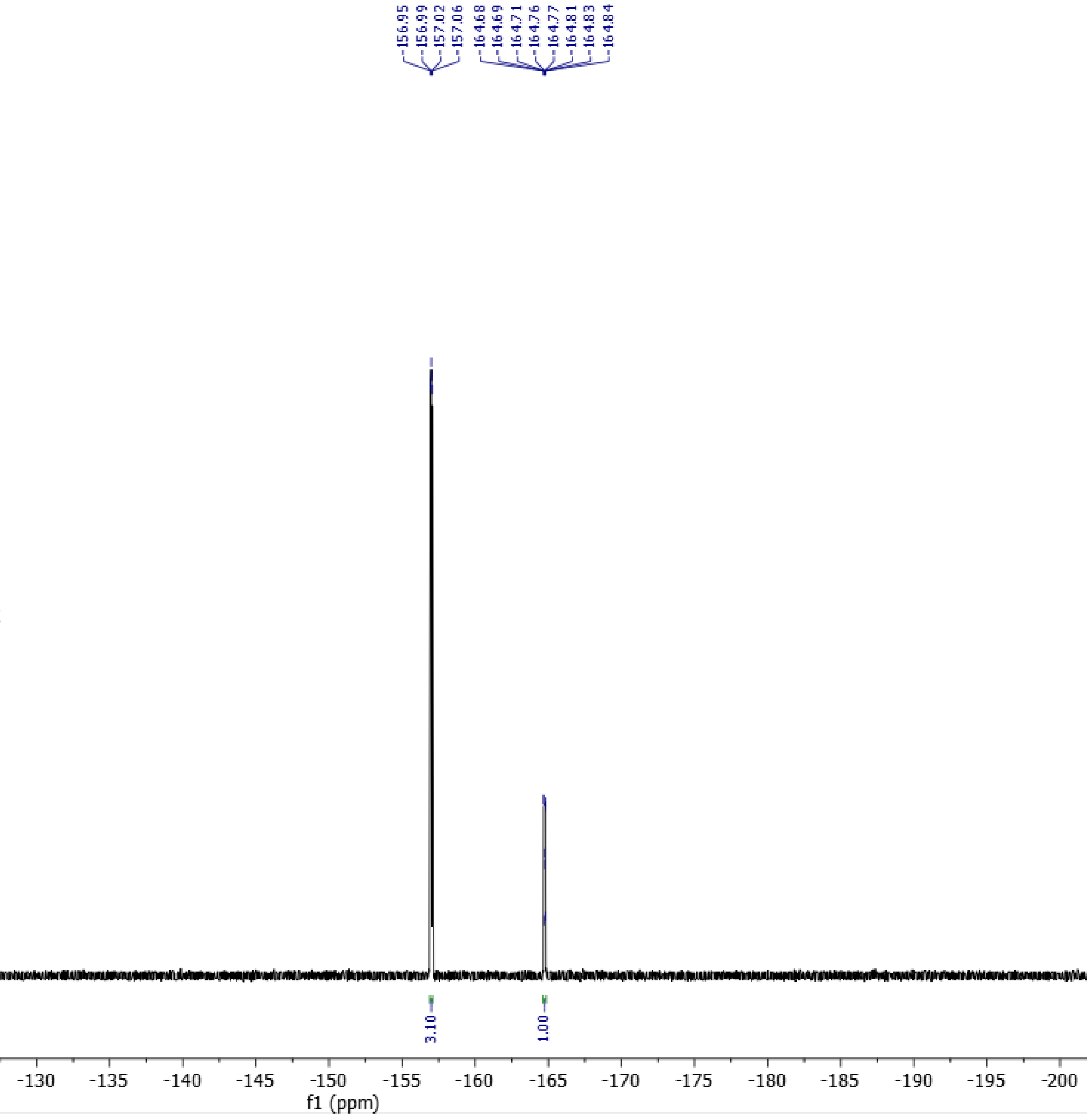

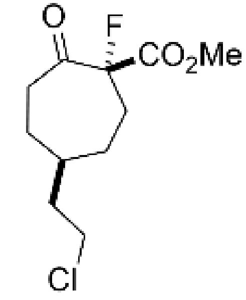

2z, dr 3.1:1

Analysis: ^{13}C NMR
Solvent: $CDCl_3$
Field strength: 101 MHz

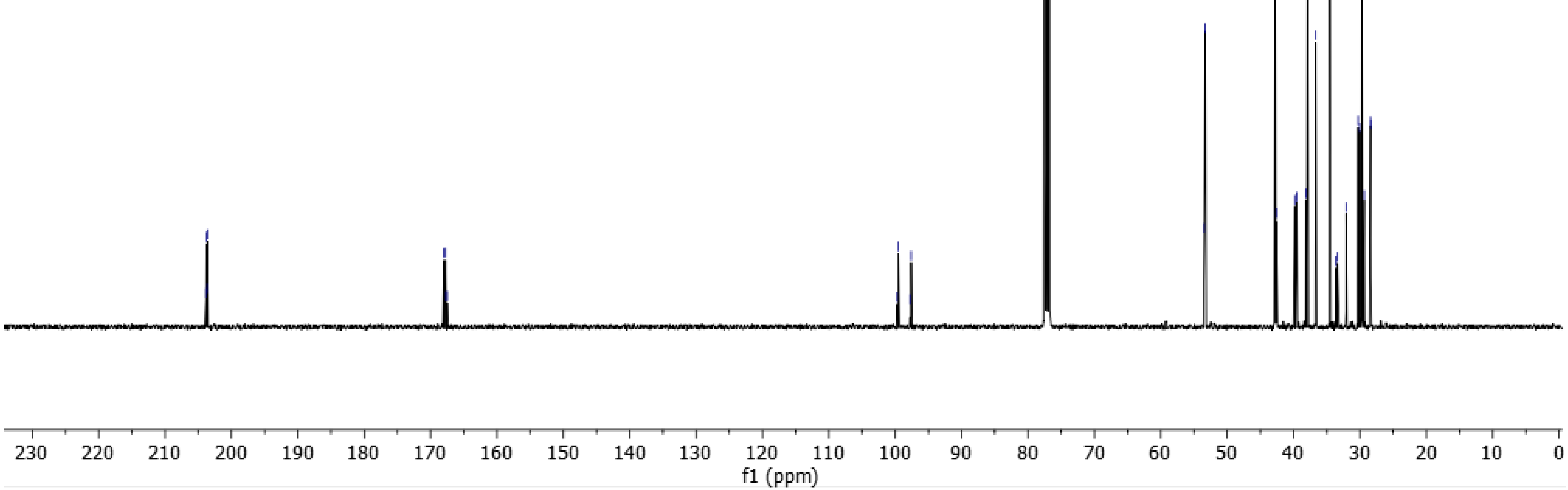

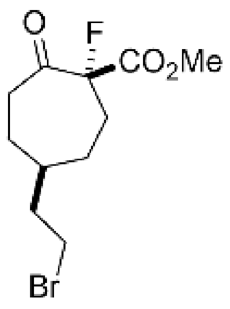

2aa, dr 3.4:1

Analysis: ^{1}H NMR
Solvent: $CDCl_3$
Field strength: 400 MHz

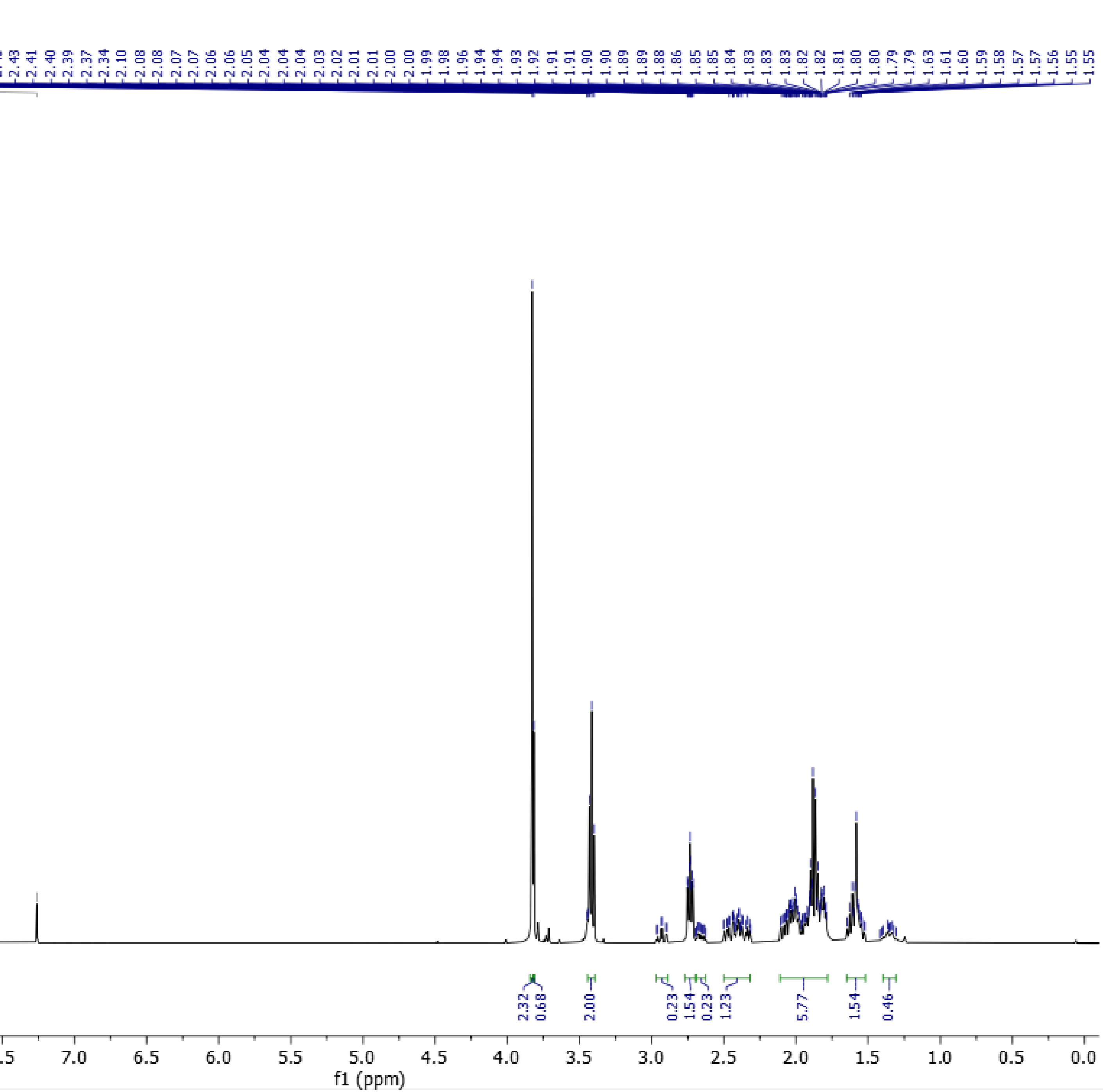

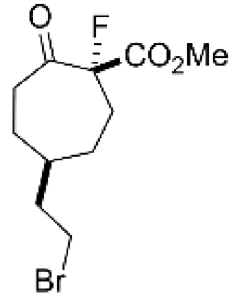

2aa, dr 3.4:1

Analysis: ^{19}F NMR
Solvent: $CDCl_3$
Field strength: 377 MHz

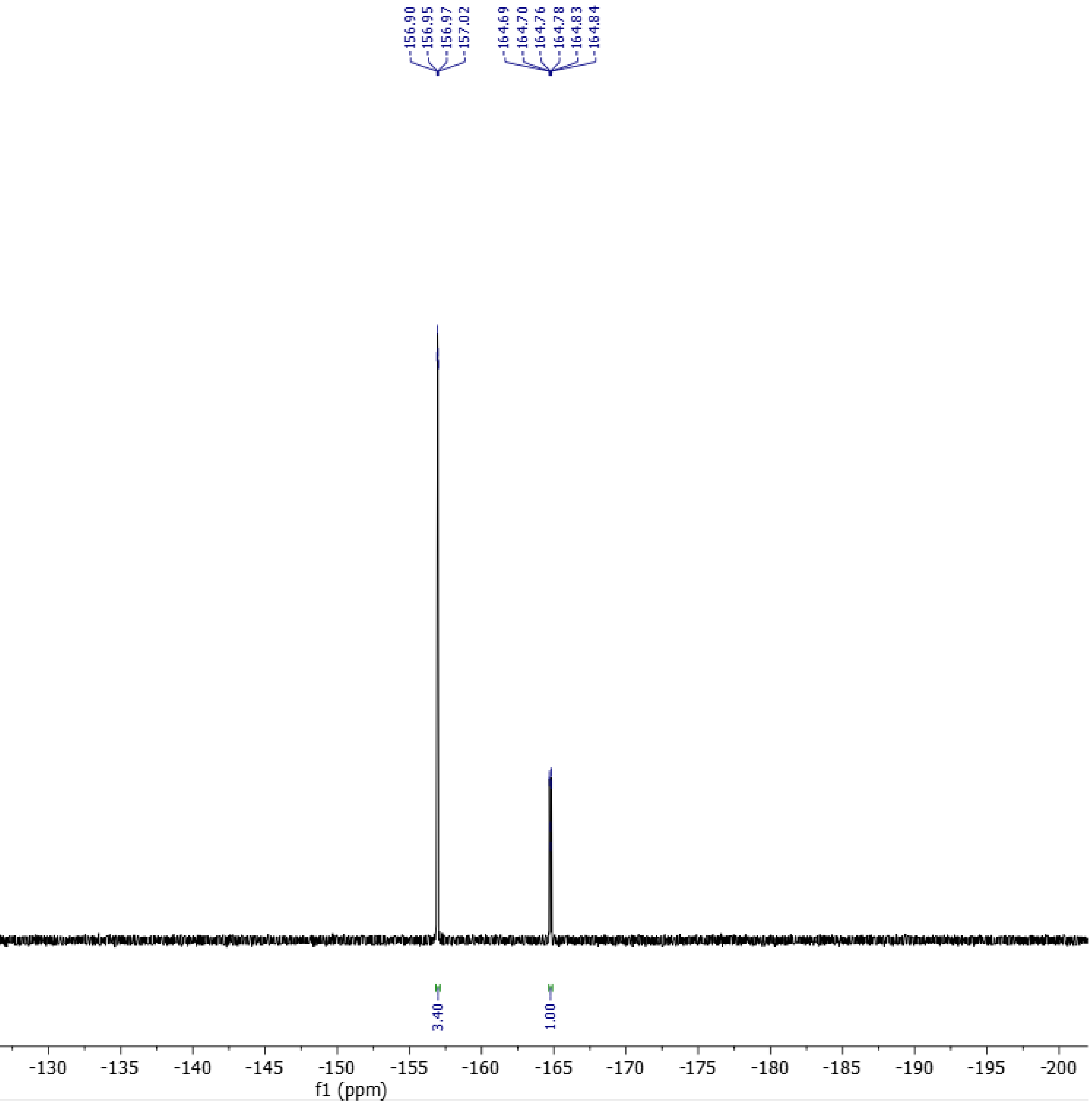

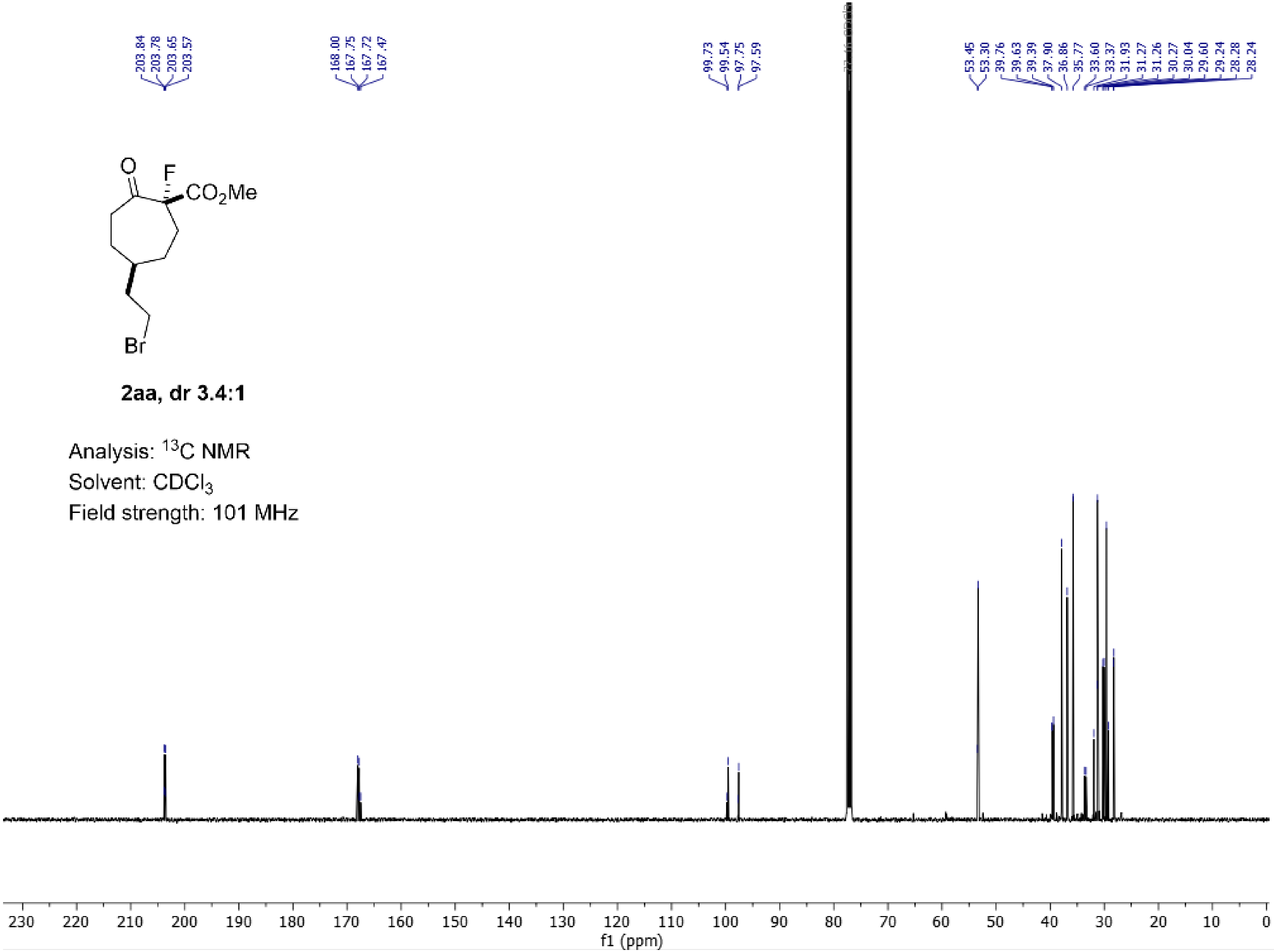

2aa, dr 3.4:1

Analysis: ^{13}C NMR
Solvent: $CDCl_3$
Field strength: 101 MHz

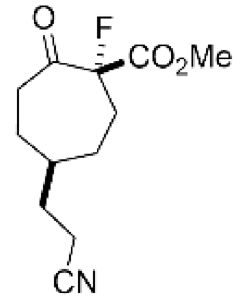

2ab, dr 3:1

Analysis: ^{1}H NMR
Solvent: $CDCl_3$
Field strength: 400 MHz

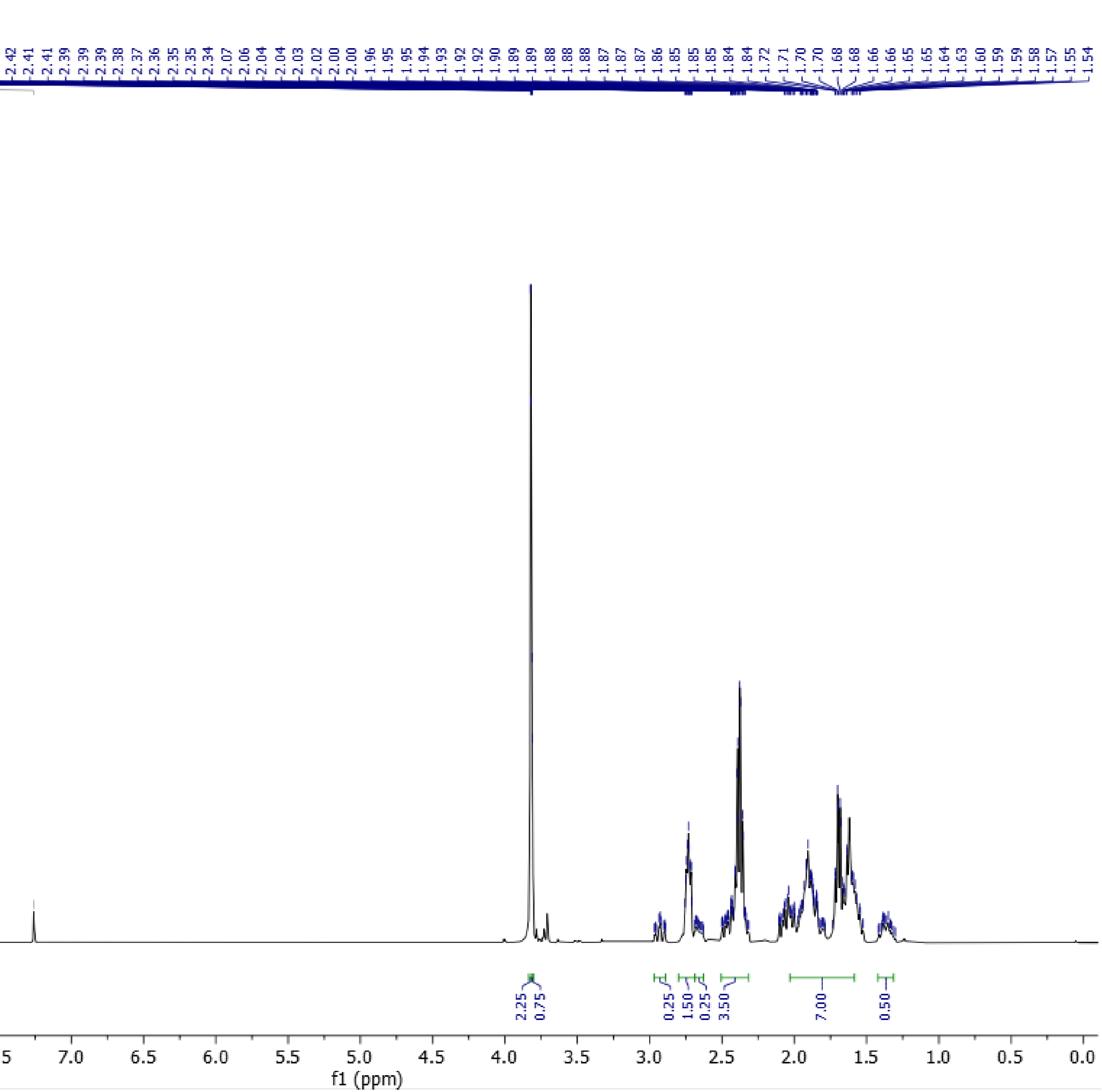

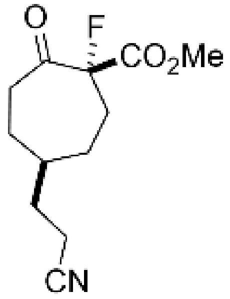

2ab, dr 3:1

Analysis: ^{19}F NMR
Solvent: $CDCl_3$
Field strength: 377 MHz

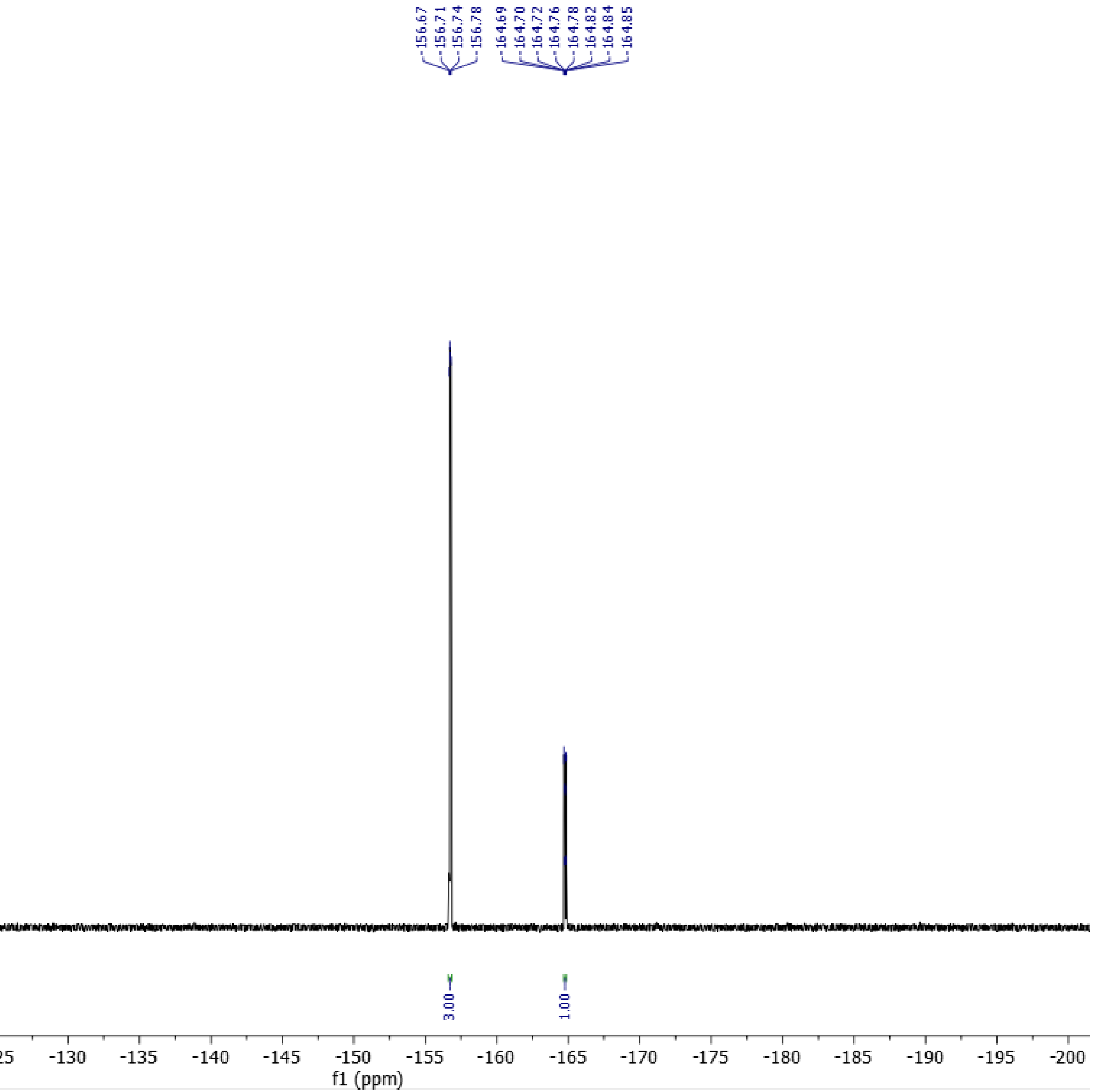

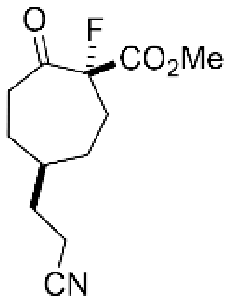

2ab, dr 3:1

Analysis: ^{13}C NMR
Solvent: $CDCl_3$
Field strength: 101 MHz

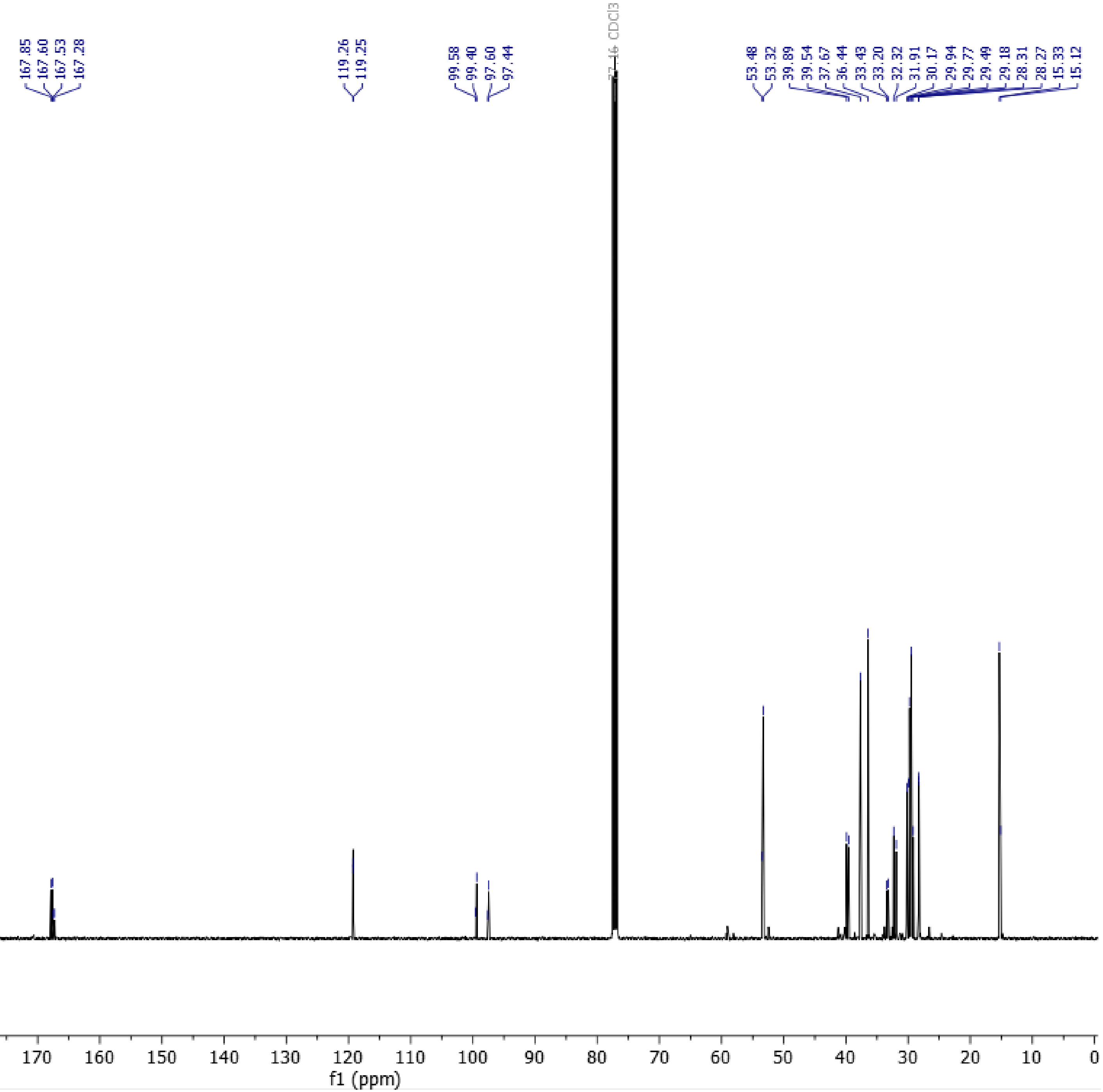

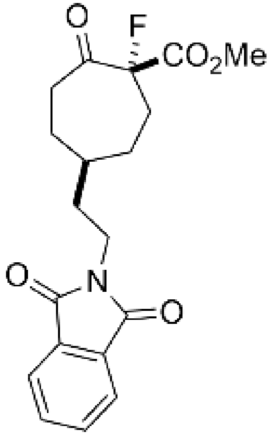

2ac, dr 4:1

Analysis: ^{1}H NMR
Solvent: $CDCl_3$
Field strength: 400 MHz

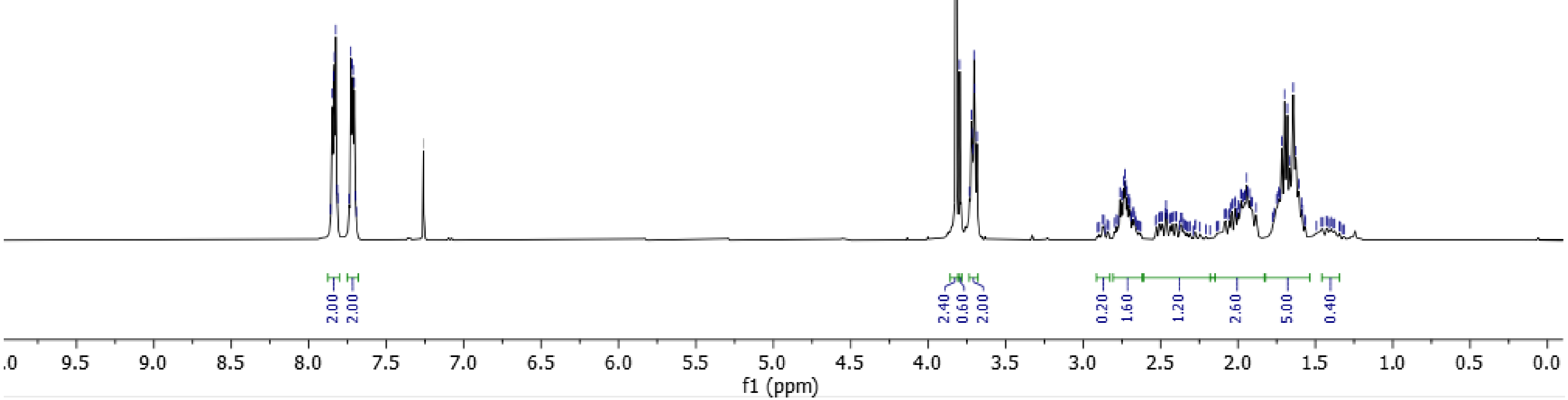

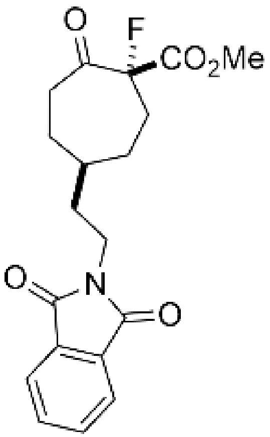

2ac, dr 4:1

Analysis: ^{19}F NMR
Solvent: $CDCl_3$
Field strength: 377 MHz

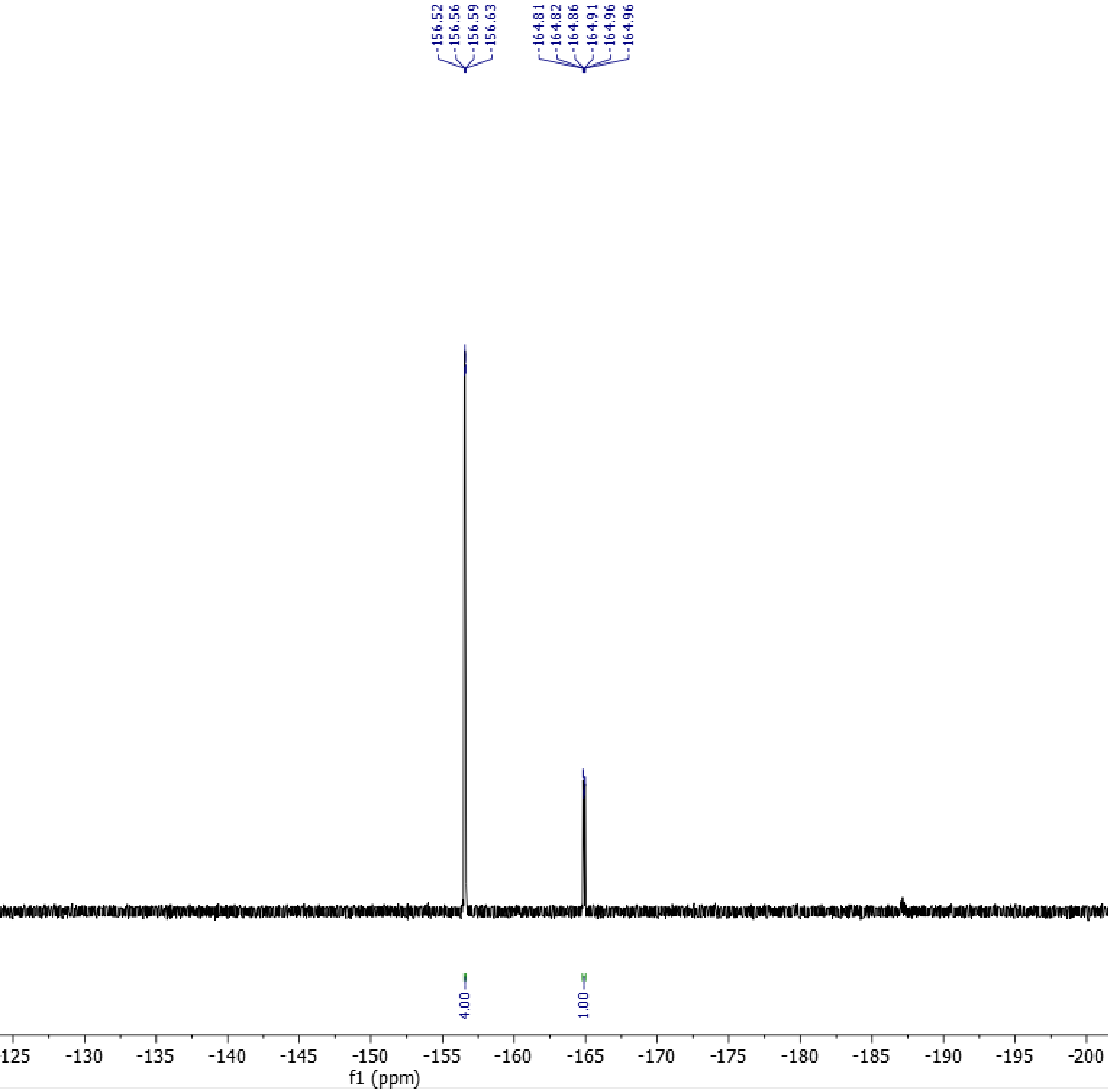

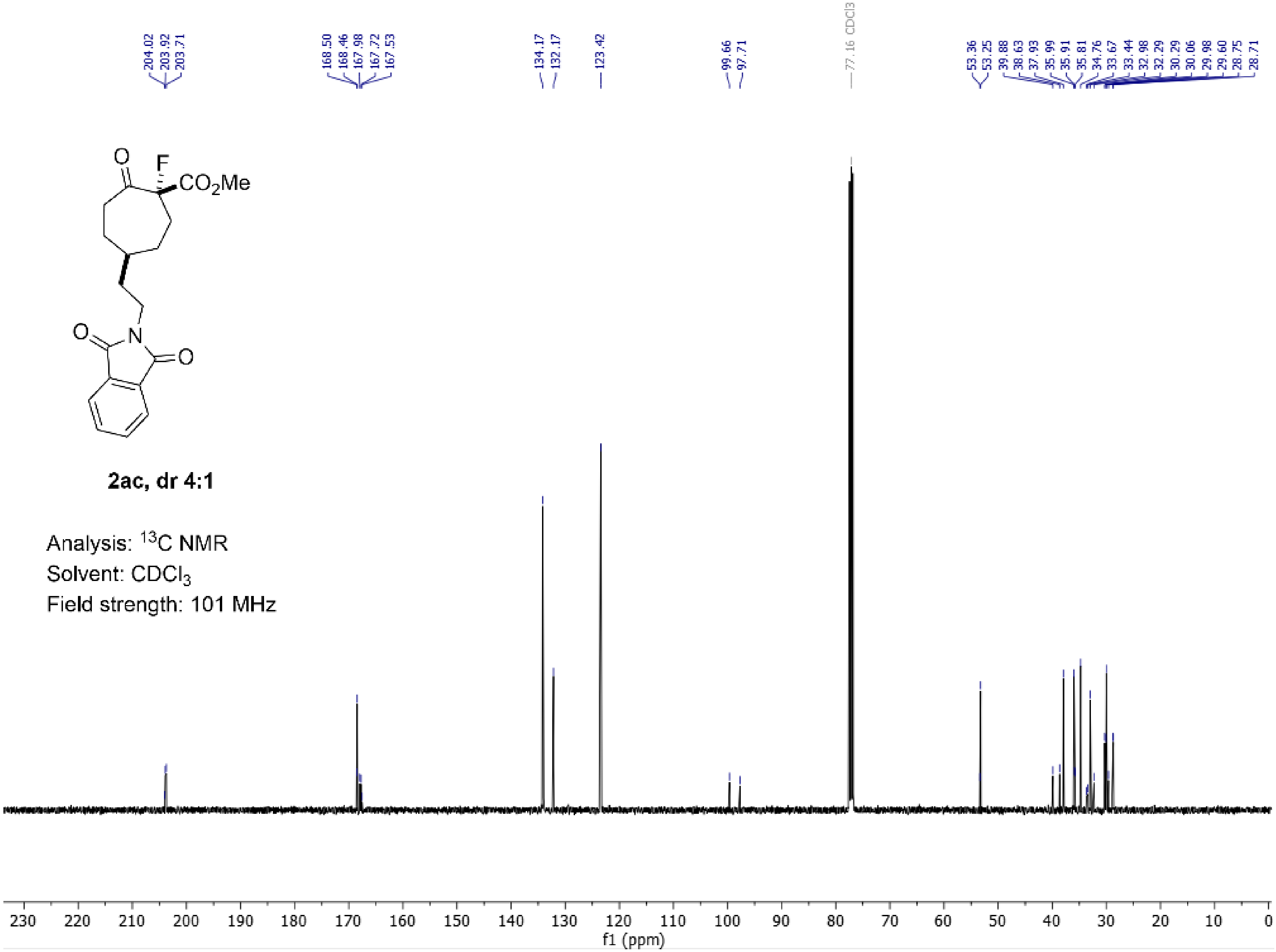
204.02
203.92
203.71
168.50
168.46
167.98
167.72
167.53
134.17
132.17
123.42
99.66
97.71
77.16 CDCl3
53.36
53.25
39.88
38.63
37.93
35.99
35.91
35.81
34.76
33.67
33.44
32.98
32.29
30.29
30.06
29.98
29.60
28.75
28.71
2ac, dr 4:1
Analysis: 13C NMR
Solvent: CDCl3
Field strength: 101 MHz
230 220 210 200 190 180 170 160 150 140 130 120 110 100 90 80 70 60 50 40 30 20 10 0
f1 (ppm)

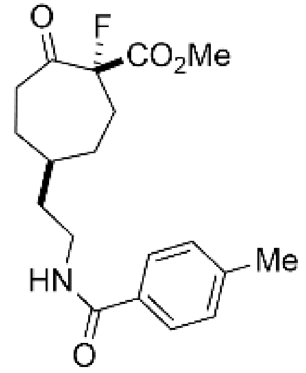

2ad, dr 3.3:1

Analysis: ^{1}H NMR
Solvent: $CDCl_3$
Field strength: 400 MHz

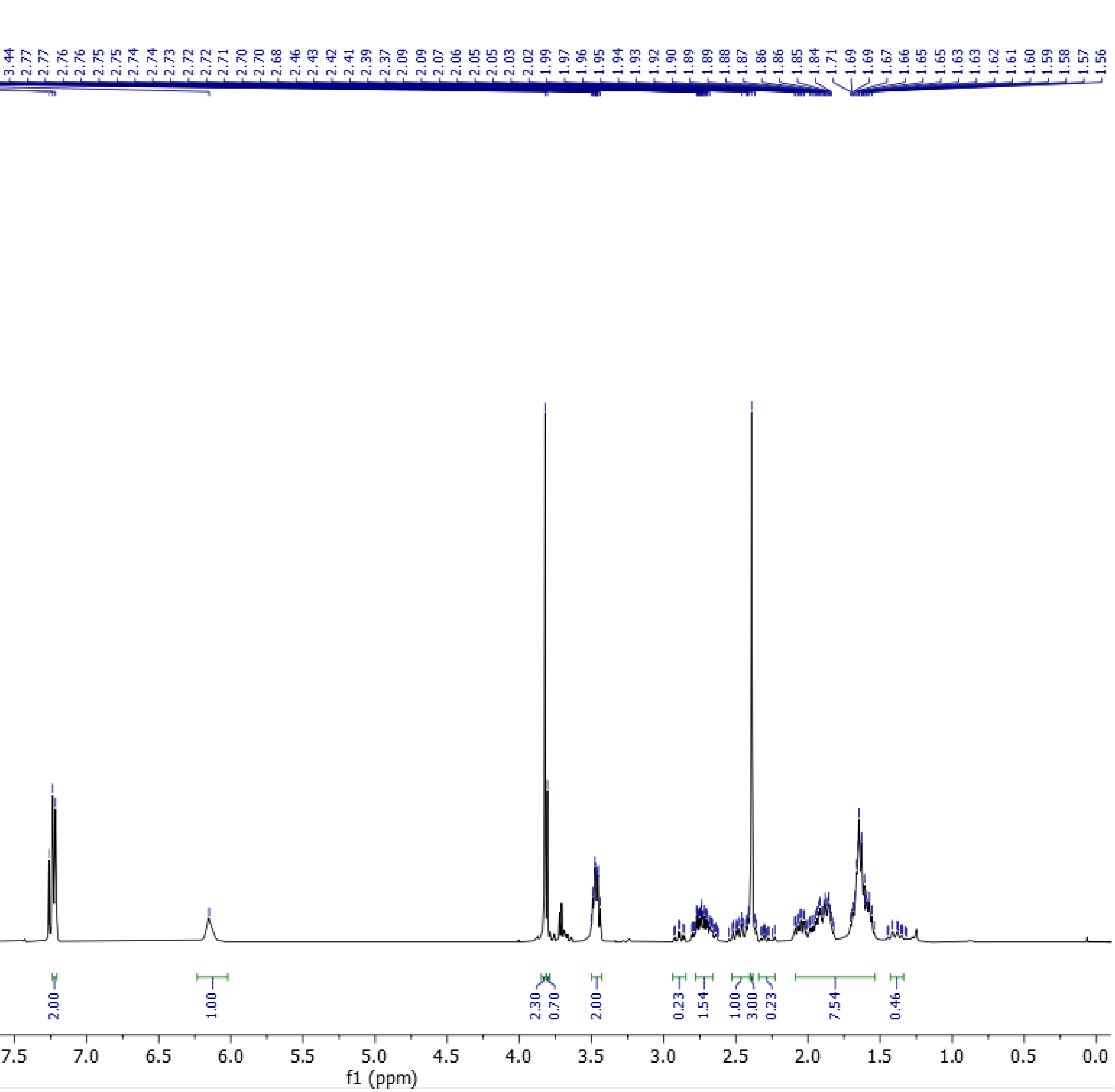

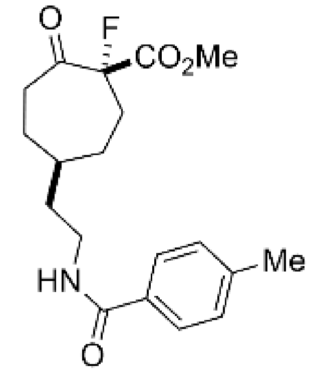

2ad, dr 3.3:1

Analysis: ^{19}F NMR
Solvent: $CDCl_3$
Field strength: 377 MHz

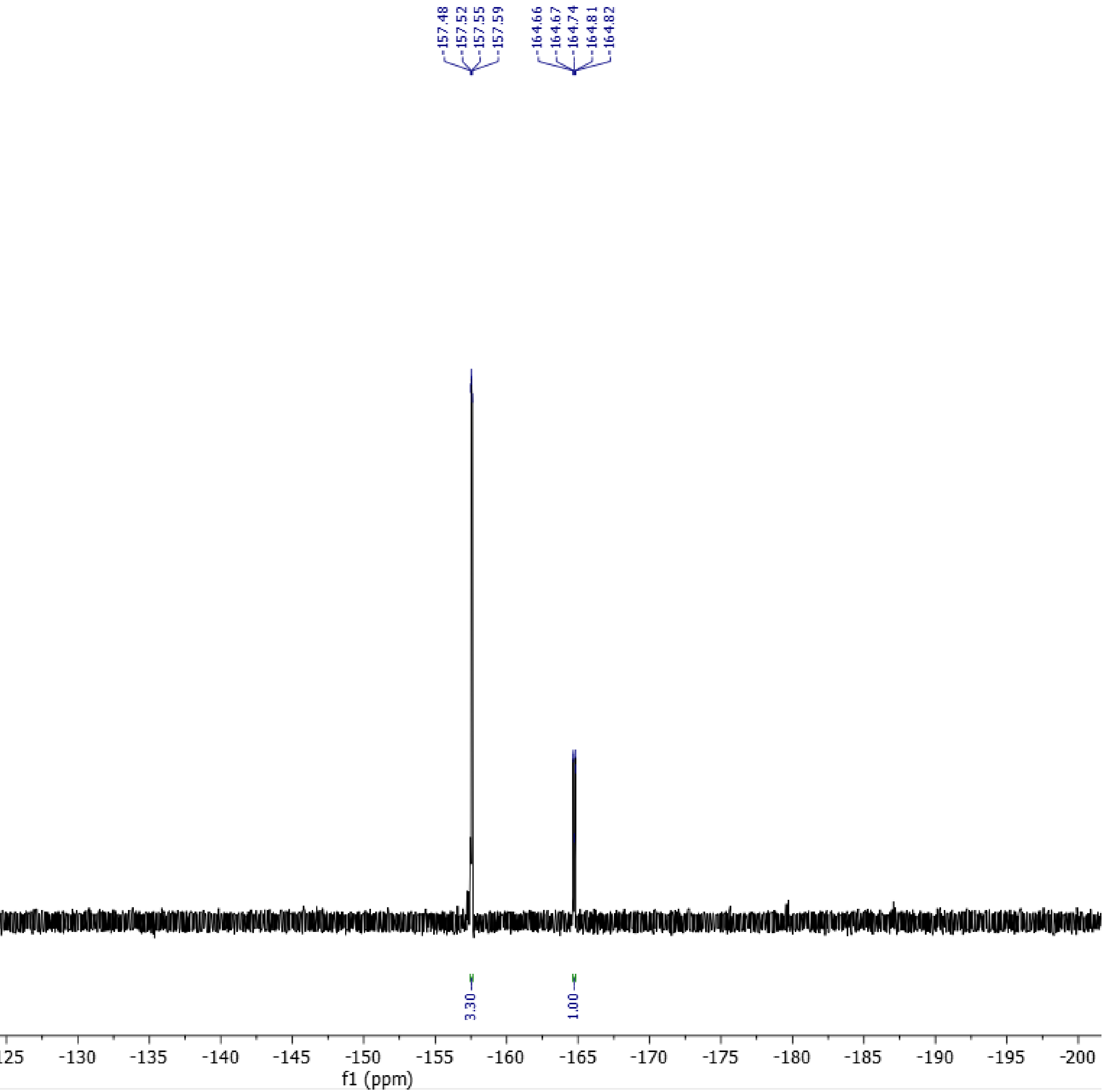

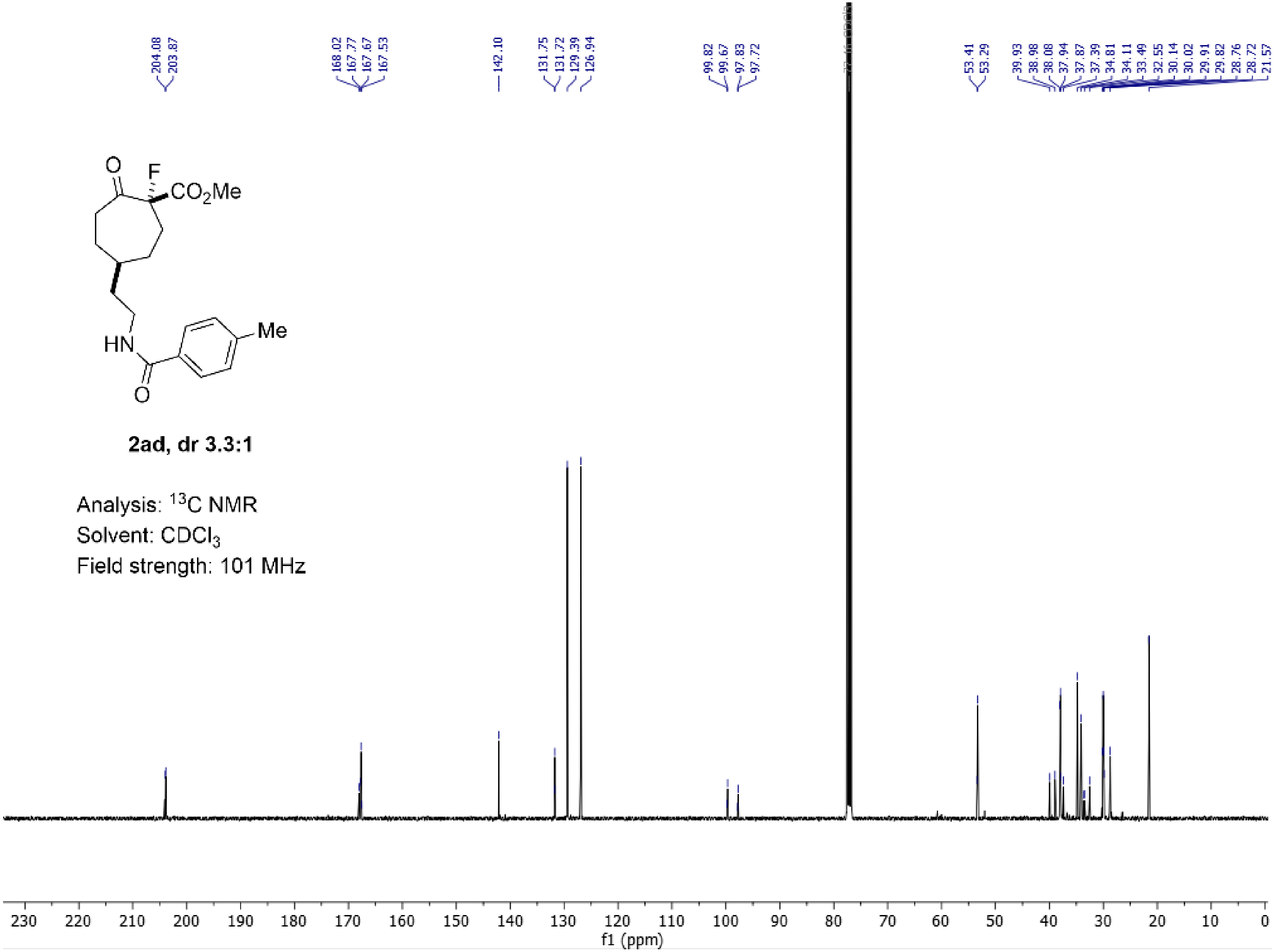
204.08
203.87
168.02
167.77
167.67
167.53
142.10
131.75
131.72
129.39
126.94
99.82
99.67
97.83
97.72
53.41
53.29
39.93
38.98
38.08
37.94
37.87
37.39
34.81
34.11
33.49
32.55
30.14
30.02
29.91
29.82
28.76
28.72
21.57
O
F
CO_2Me
HN
Me
O
2ad, dr 3.3:1
Analysis: ^{13}C NMR
Solvent: $CDCl_3$
Field strength: 101 MHz
230 220 210 200 190 180 170 160 150 140 130 120 110 100 90 80 70 60 50 40 30 20 10 0
f1 (ppm)

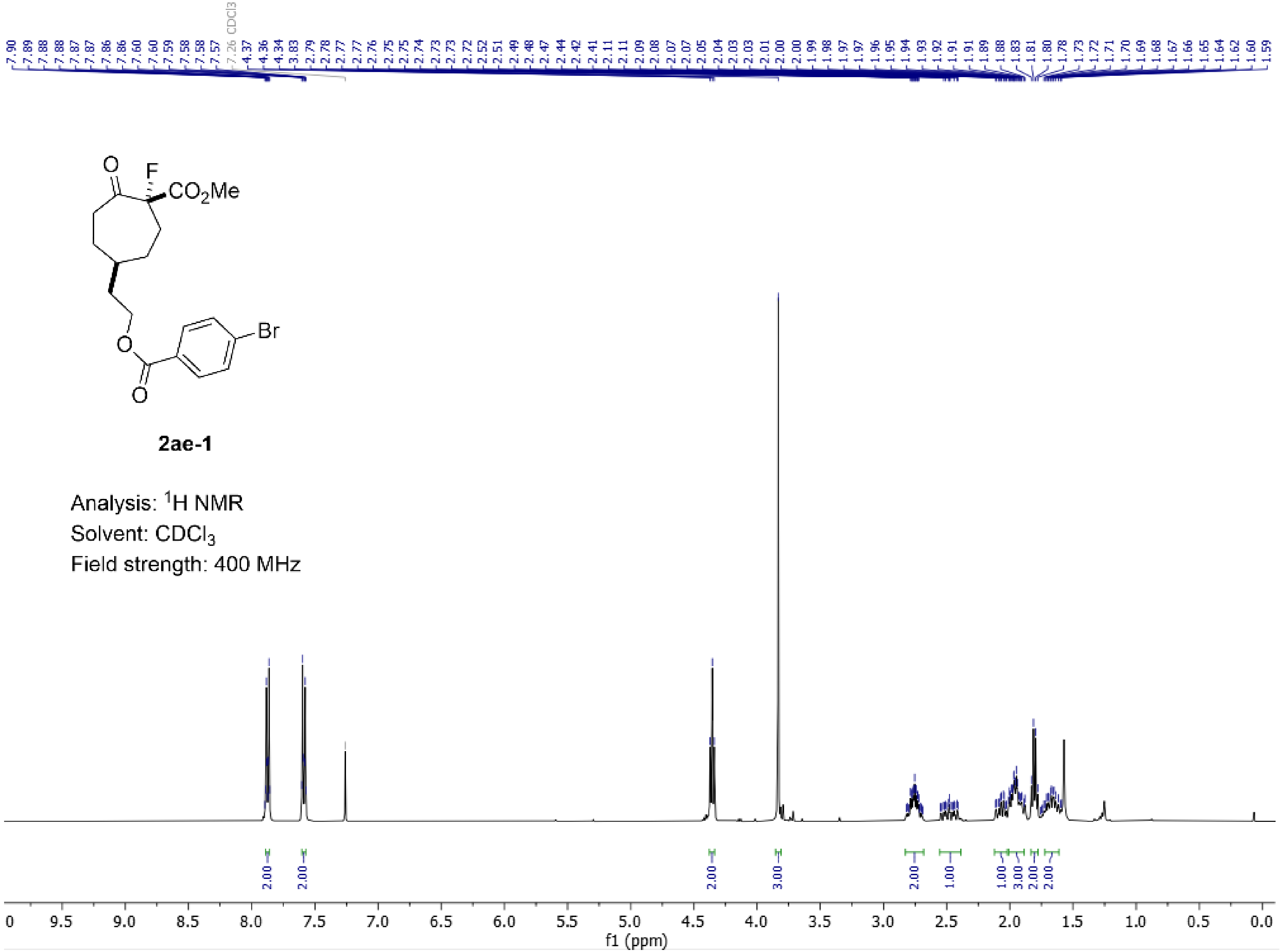
O
F
CO_2Me
O
O
Br
2ae-1
Analysis: ^1H NMR
Solvent: $CDCl_3$
Field strength: 400 MHz
f1 (ppm)

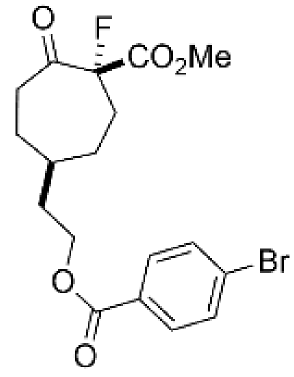

2ae-1

Analysis: ^{19}F NMR
Solvent: $CDCl_3$
Field strength: 376 MHz

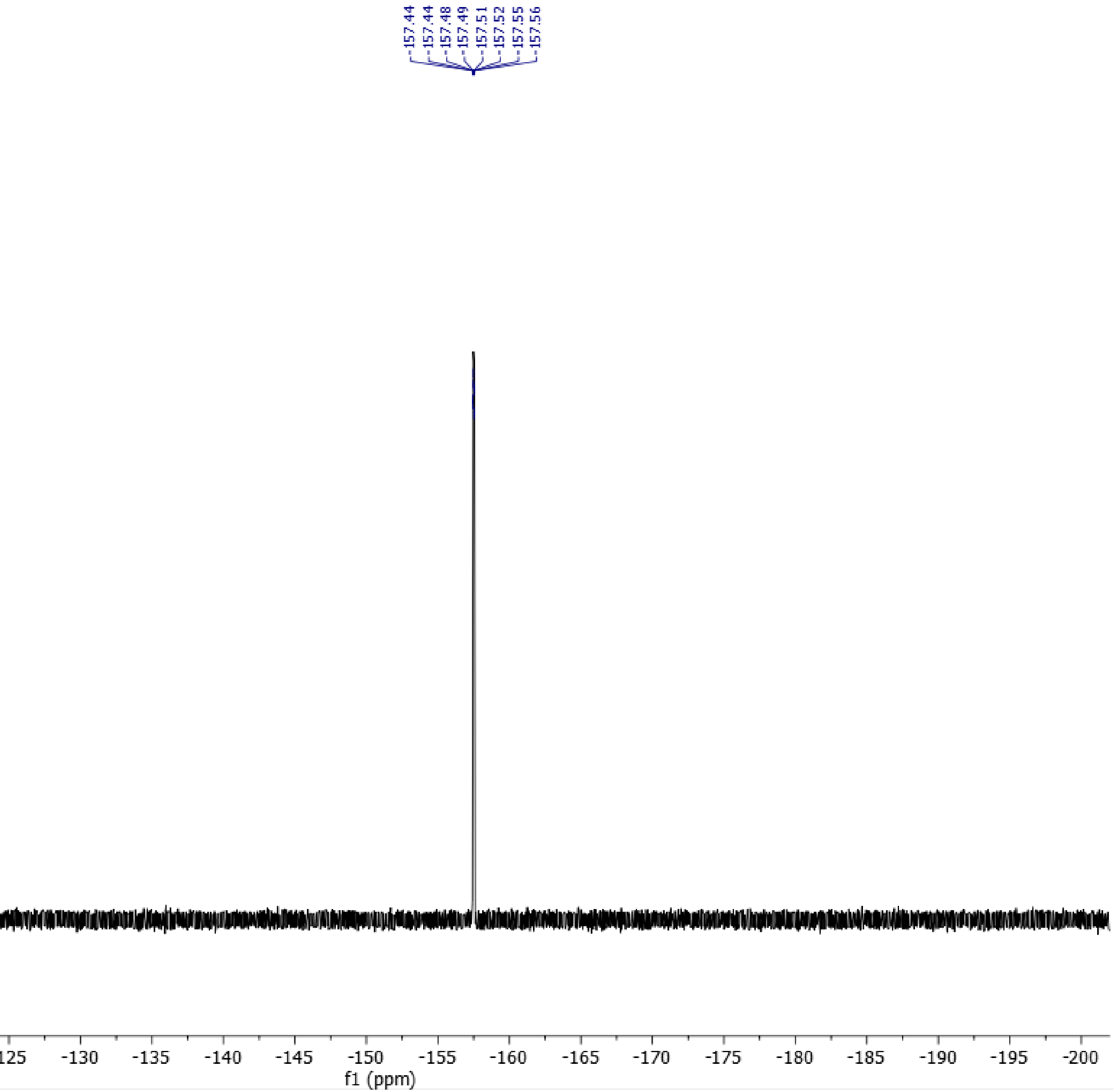

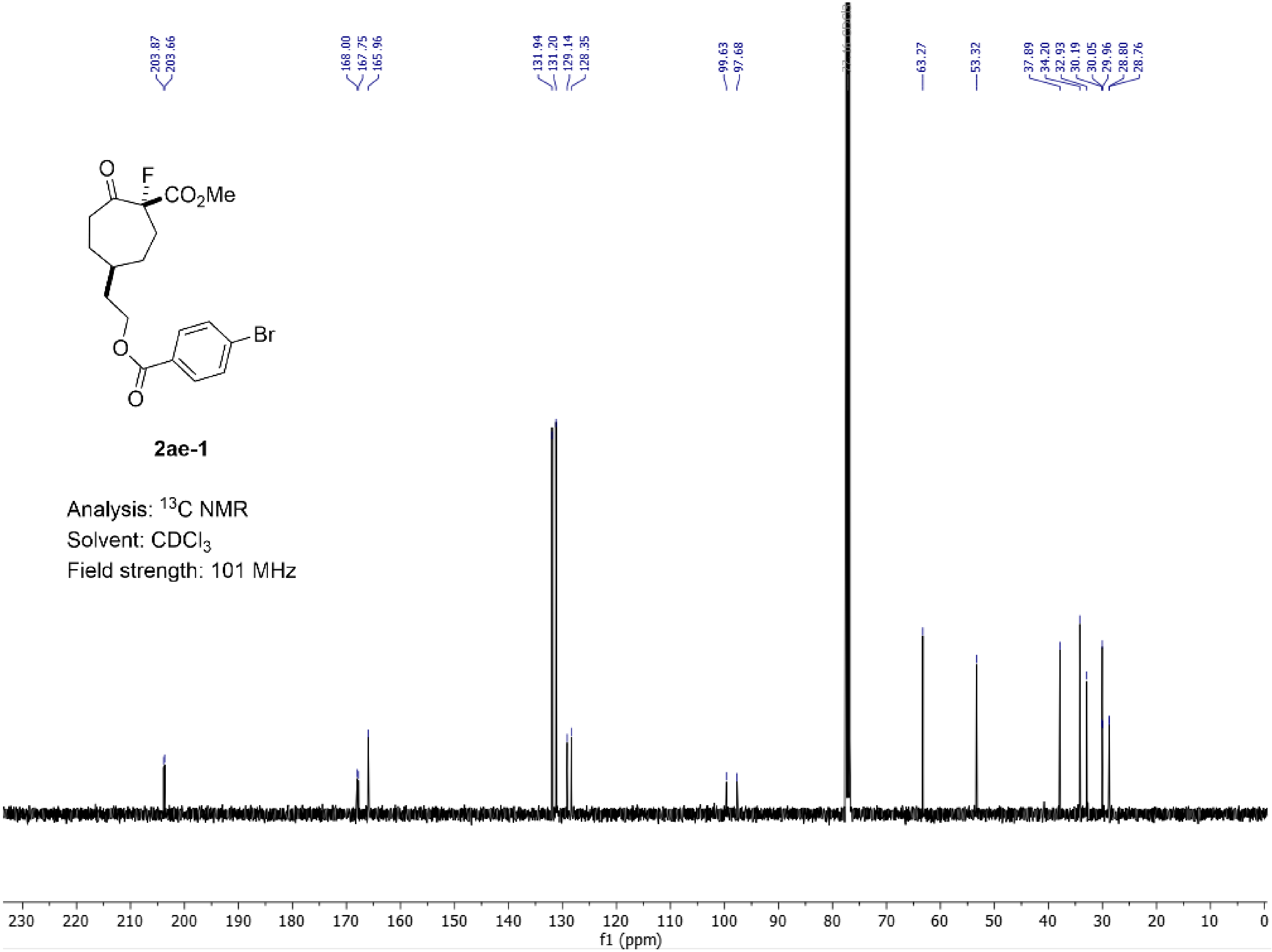
203.87
203.66
168.00
167.75
165.96
131.94
131.20
129.14
128.35
99.63
97.68
63.27
53.32
37.89
34.20
32.93
30.19
30.05
29.96
28.80
28.76
O
F
CO2Me
Br
O
O
2ae-1
Analysis: 13C NMR
Solvent: CDCl3
Field strength: 101 MHz
230 220 210 200 190 180 170 160 150 140 130 120 110 100 90 80 70 60 50 40 30 20 10 0
f1 (ppm)

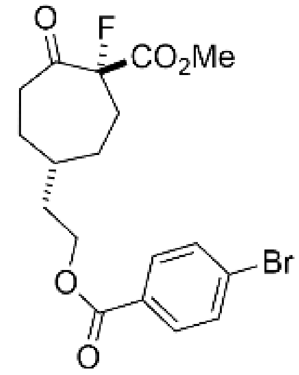

2ae-2

Analysis: ^{1}H NMR
Solvent: $CDCl_3$
Field strength: 600 MHz

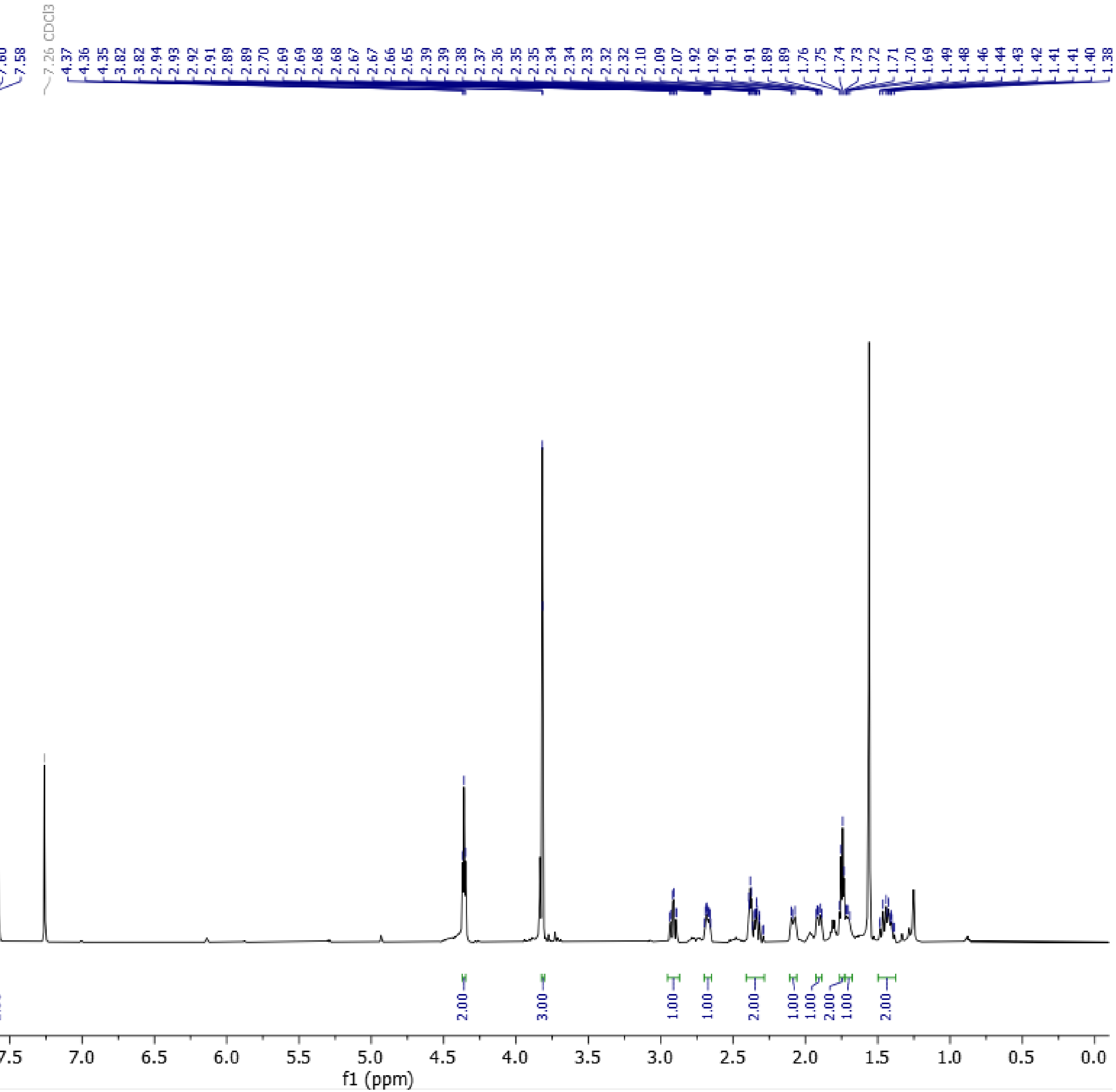

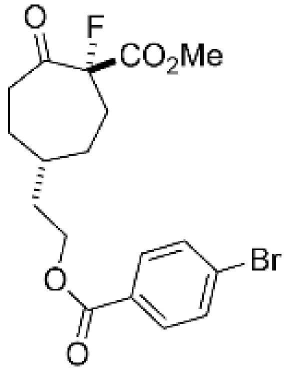

2ae-2

Analysis: ^{19}F NMR
Solvent: $CDCl_3$
Field strength: 565 MHz

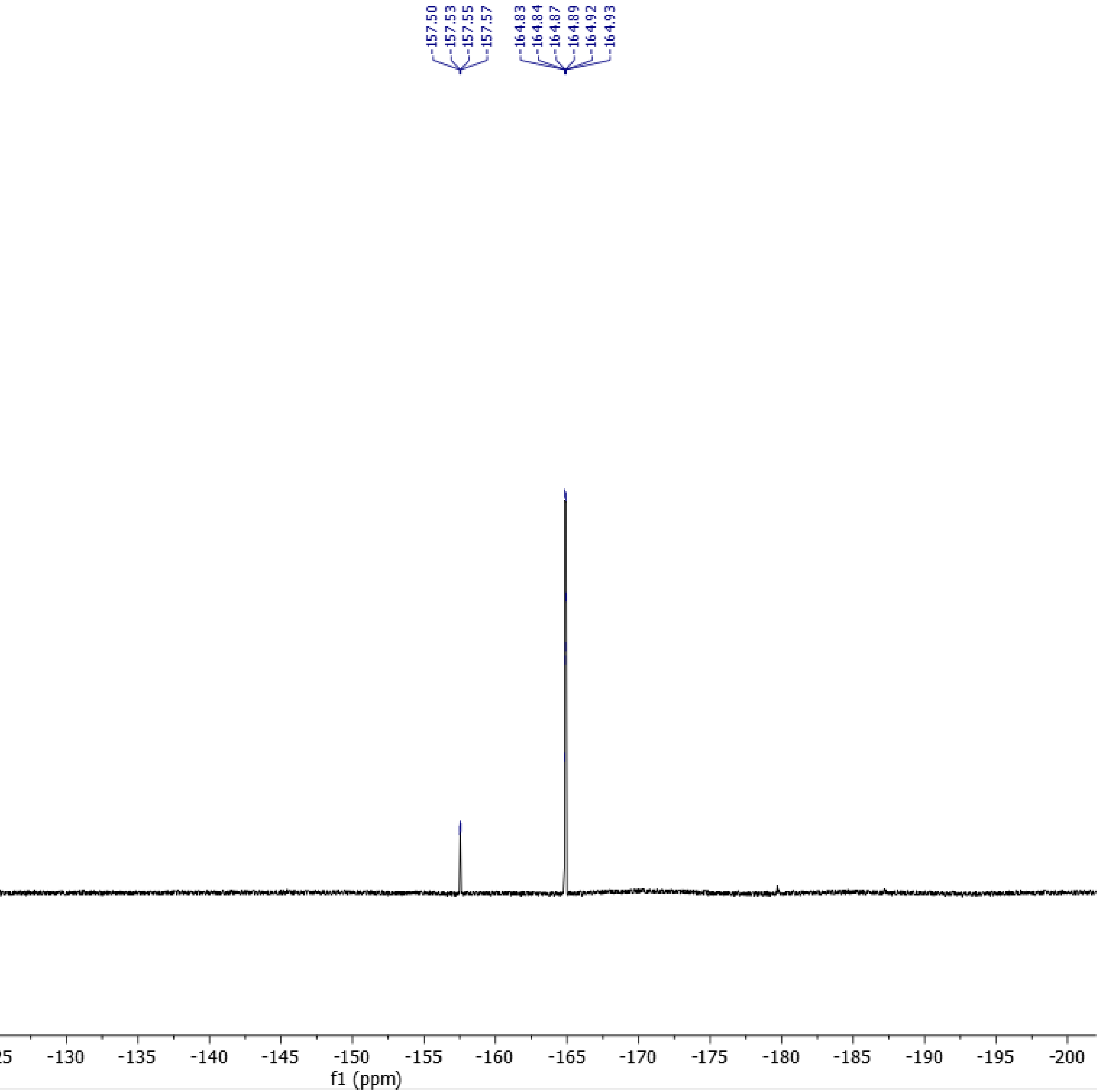

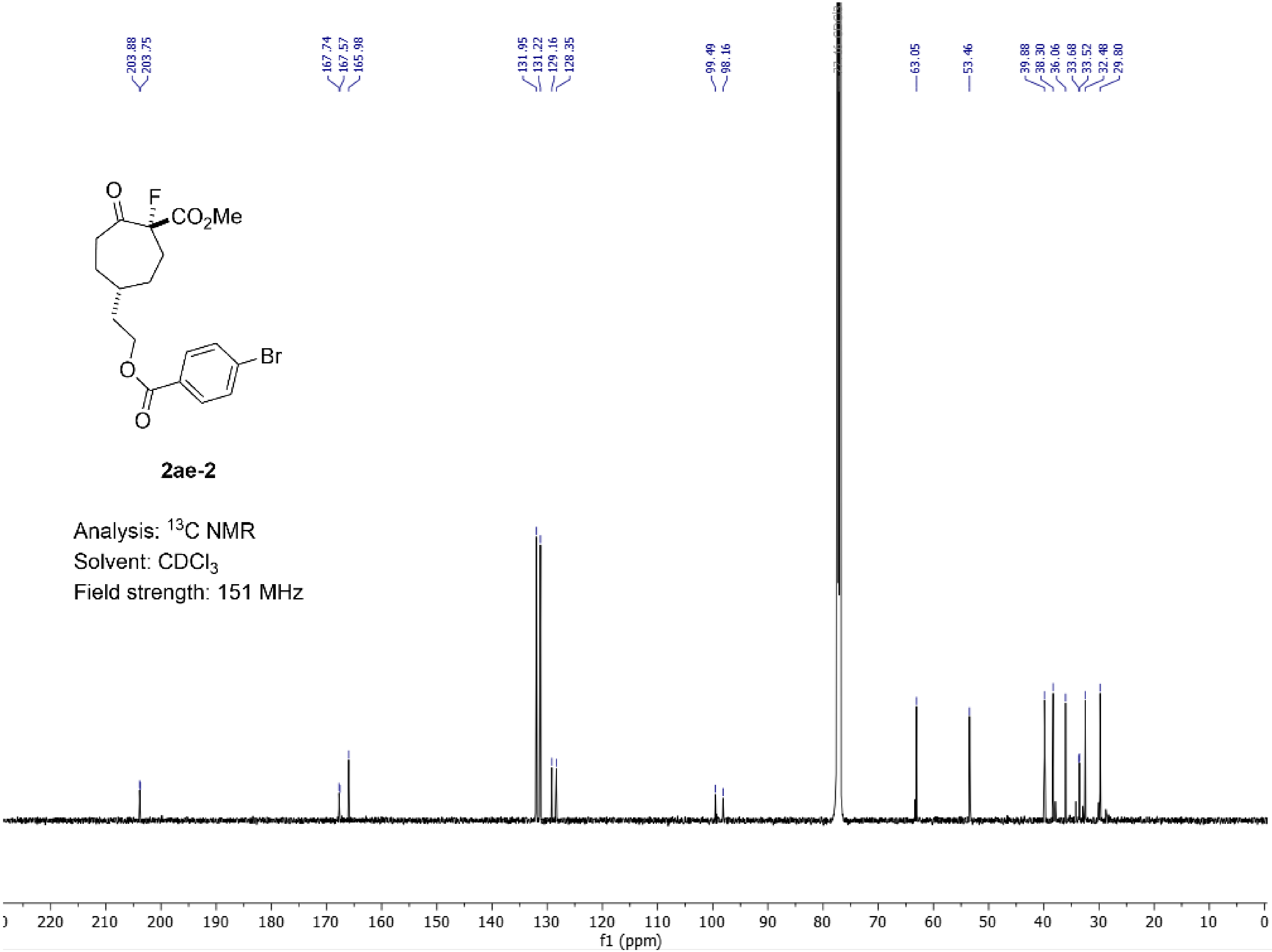
O
F
CO2Me
O
Br
O
2ae-2
Analysis: 13C NMR
Solvent: CDCl3
Field strength: 151 MHz
203.88
203.75
167.74
167.57
165.98
131.95
131.22
129.16
128.35
99.49
98.16
63.05
53.46
39.88
38.30
36.06
33.68
33.52
32.48
29.80
220 210 200 190 180 170 160 150 140 130 120 110 100 90 80 70 60 50 40 30 20 10 0
f1 (ppm)

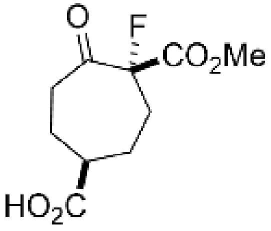

2af, dr 3.4:1

Analysis: ^{1}H NMR
Solvent: $CDCl_3$
Field strength: 400 MHz

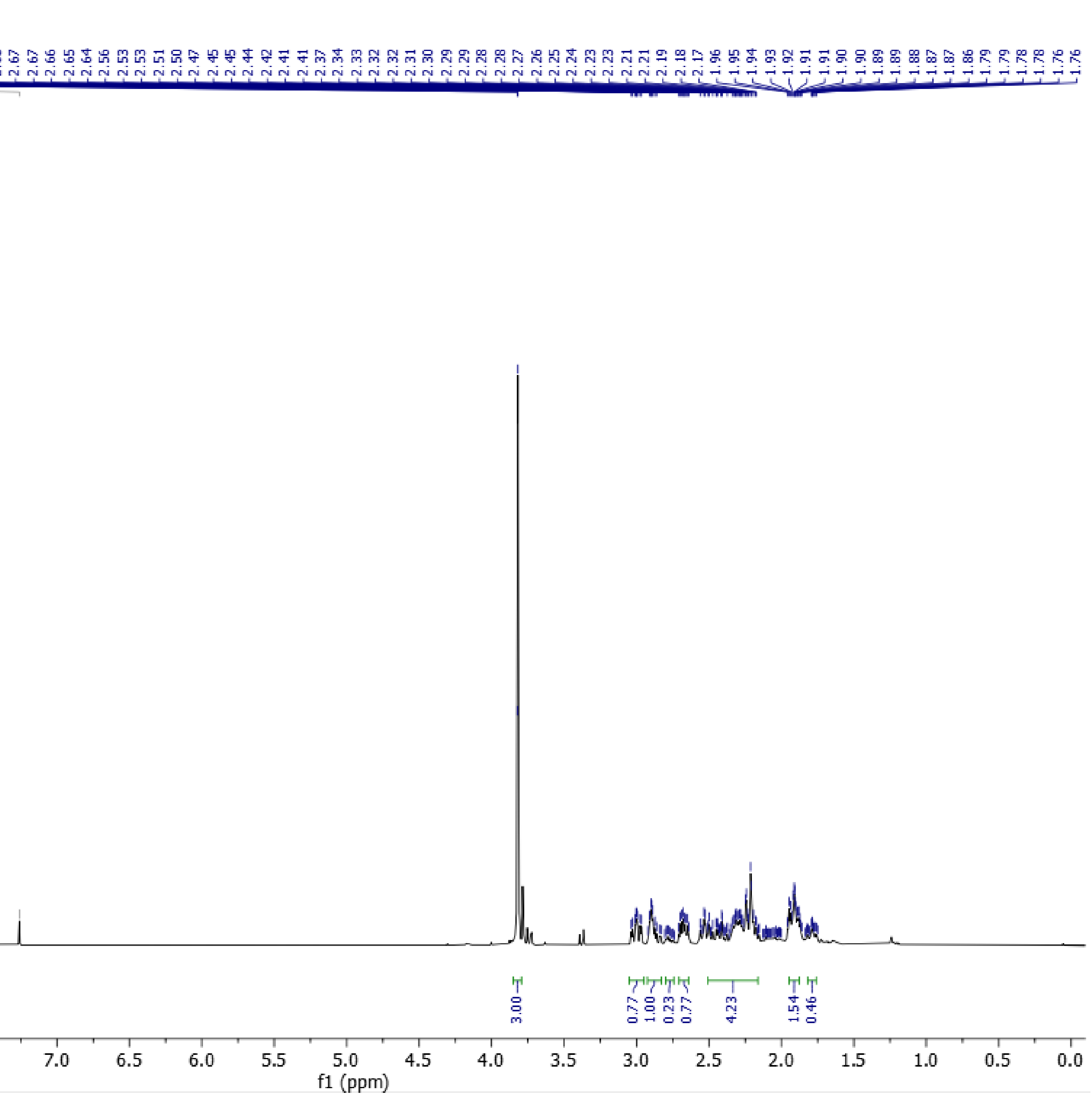

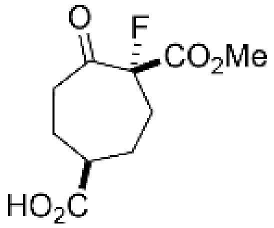

2af, dr 3.4:1

Analysis: ^{19}F NMR
Solvent: $CDCl_3$
Field strength: 377 MHz

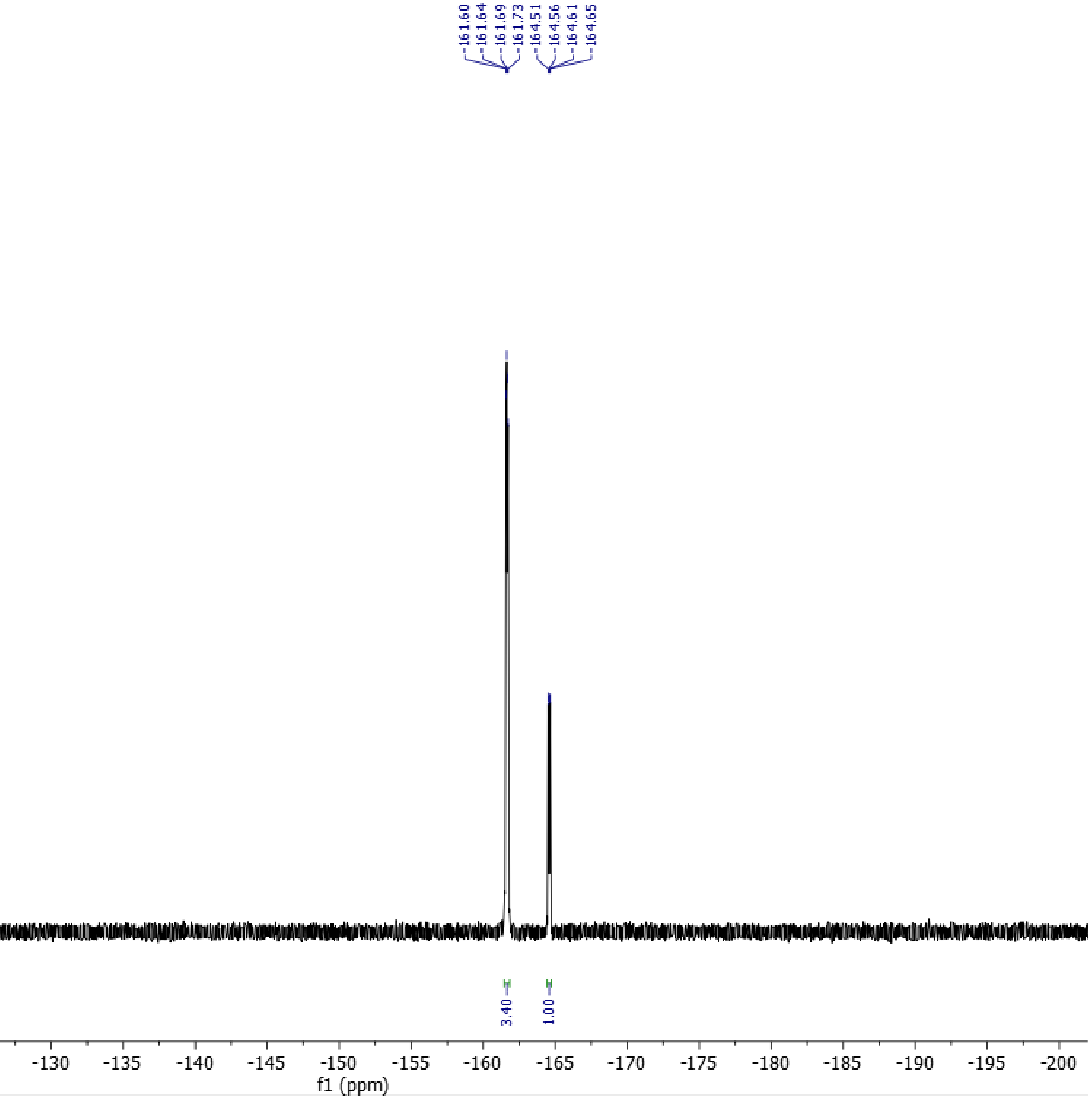

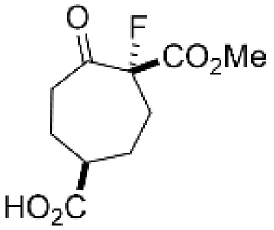

2af, dr 3.4:1

Analysis: ^{13}C NMR
Solvent: $CDCl_3$
Field strength: 101 MHz

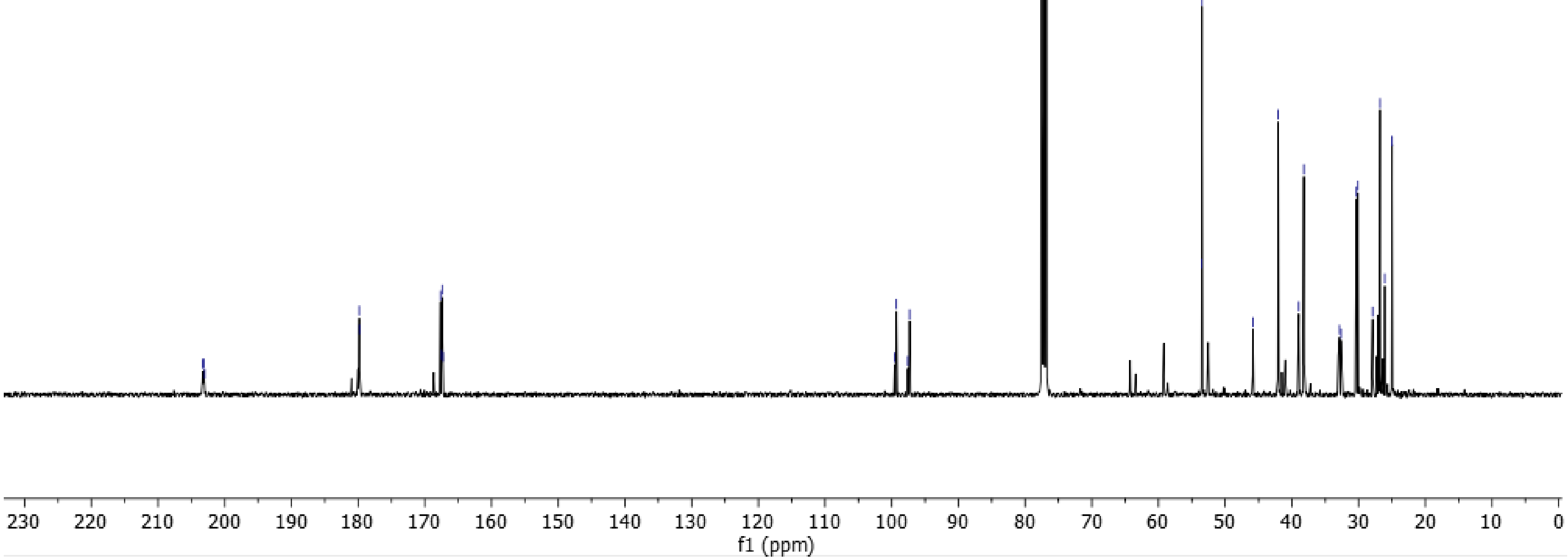

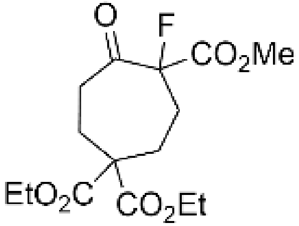

2ag

Analysis: ^{1}H NMR
Solvent: $CDCl_3$
Field strength: 400 MHz

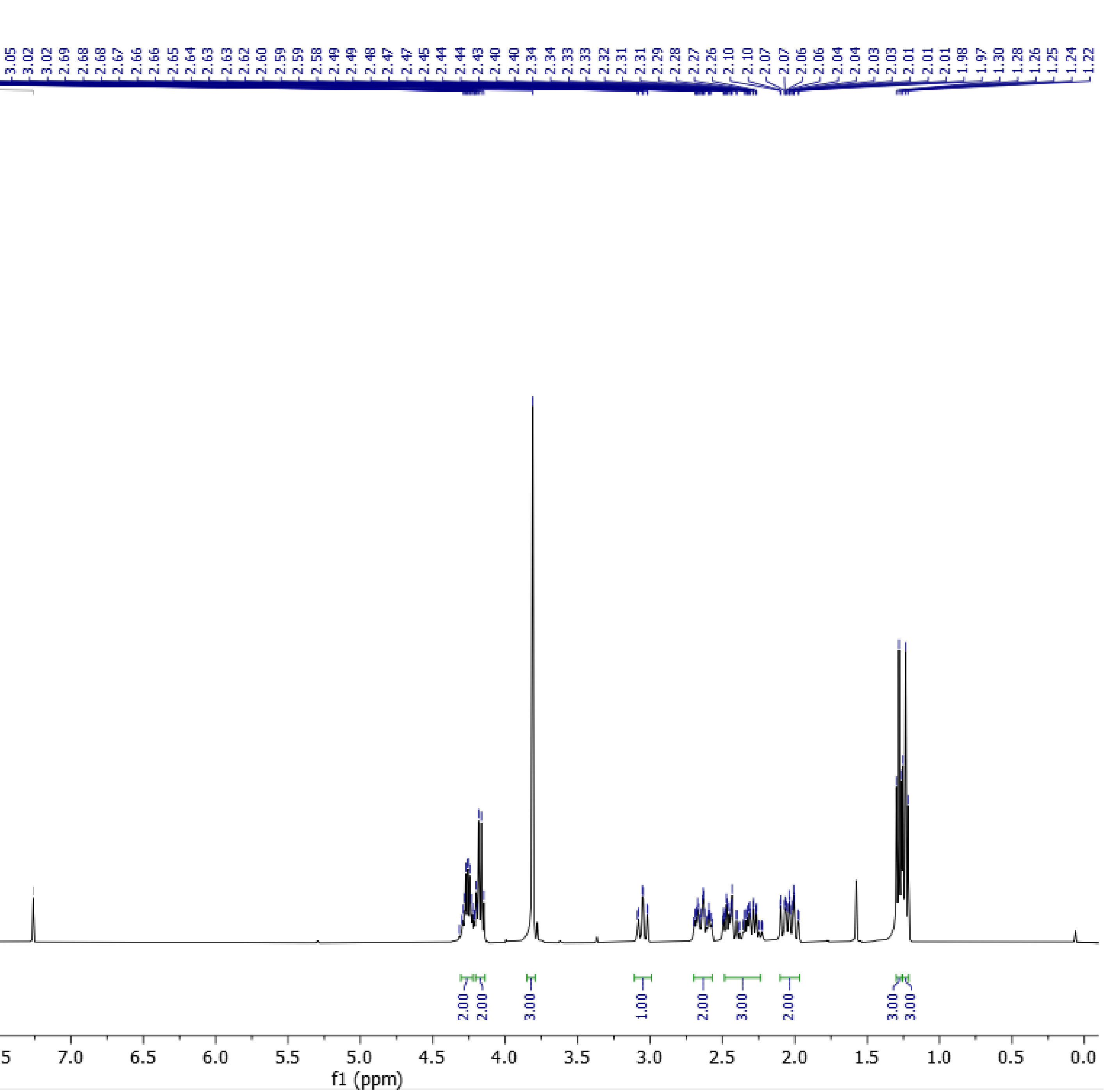

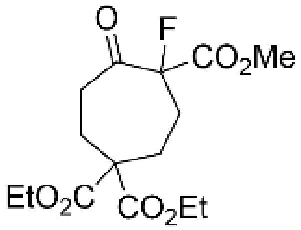

2ag

Analysis: ^{19}F NMR
Solvent: $CDCl_3$
Field strength: 377 MHz

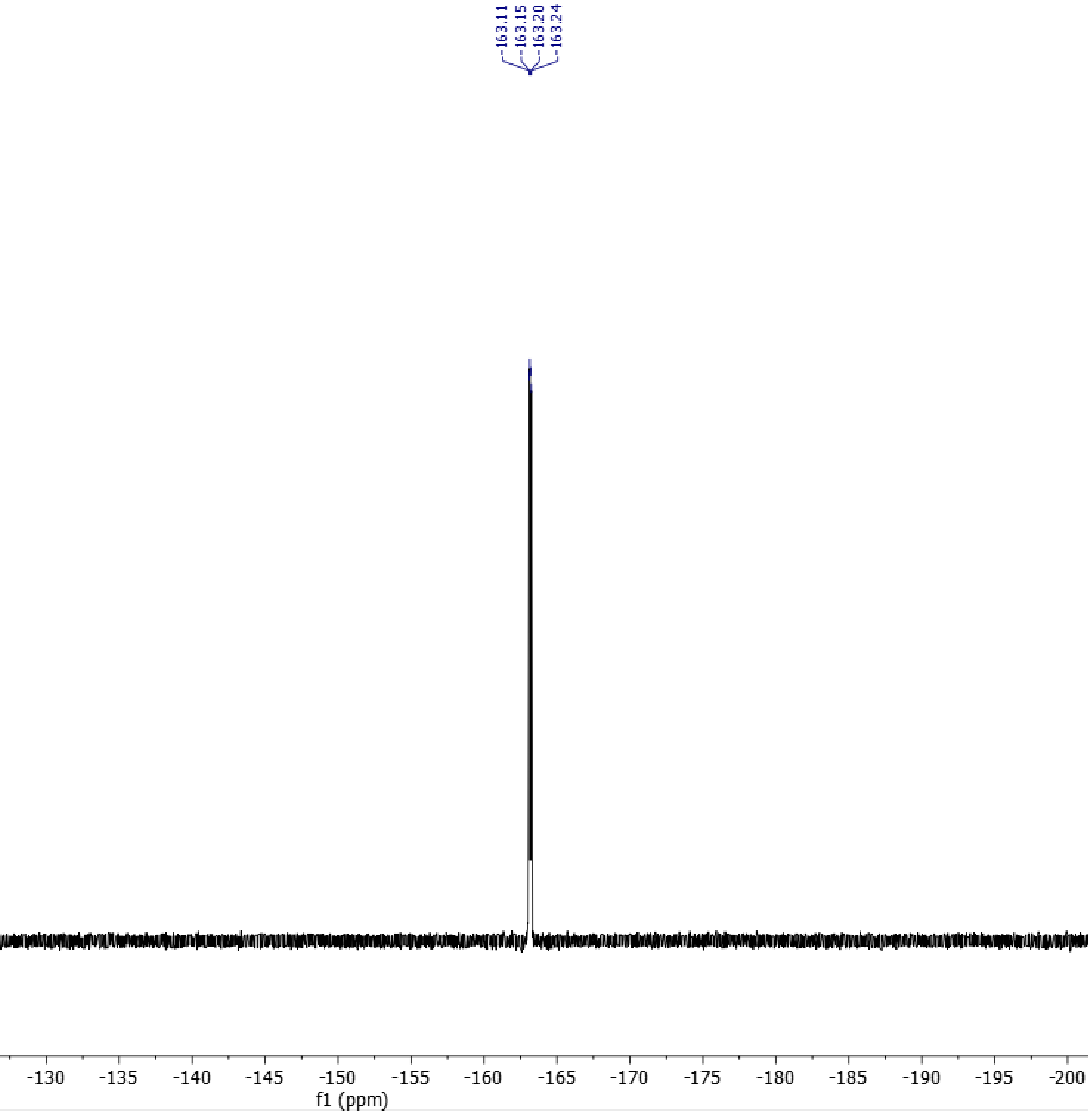

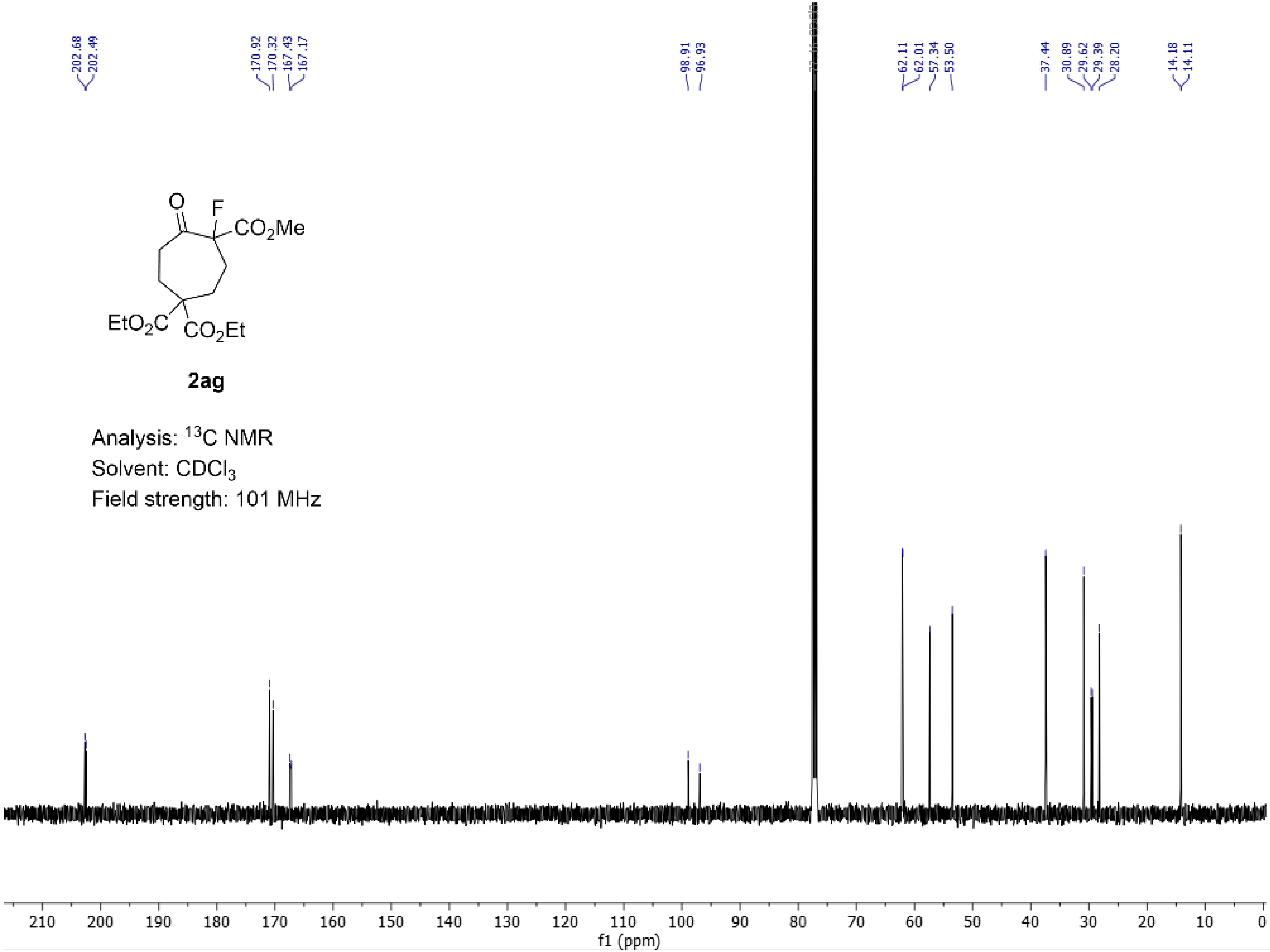

2ag

Analysis: ^{13}C NMR
Solvent: $CDCl_3$
Field strength: 101 MHz

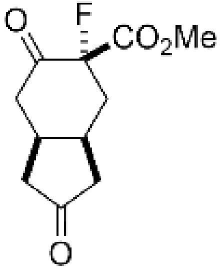

2ah-1

Analysis: ^{1}H NMR
Solvent: $CDCl_3$
Field strength: 400 MHz

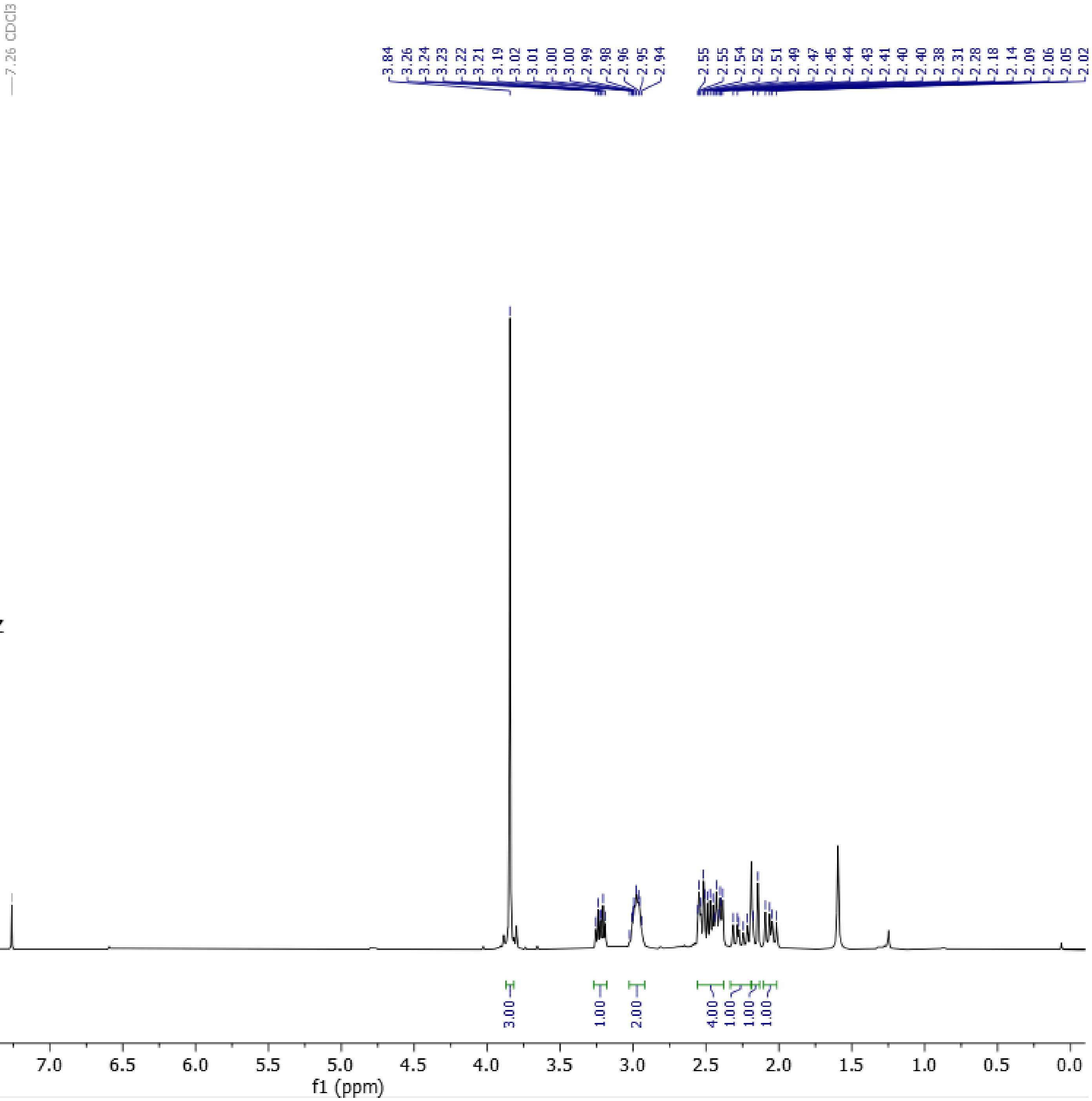

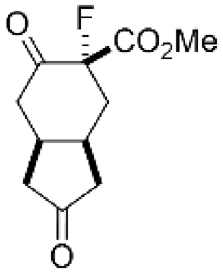

2ah-1

Analysis: ^{19}F NMR
Solvent: $CDCl_3$
Field strength: 377 MHz

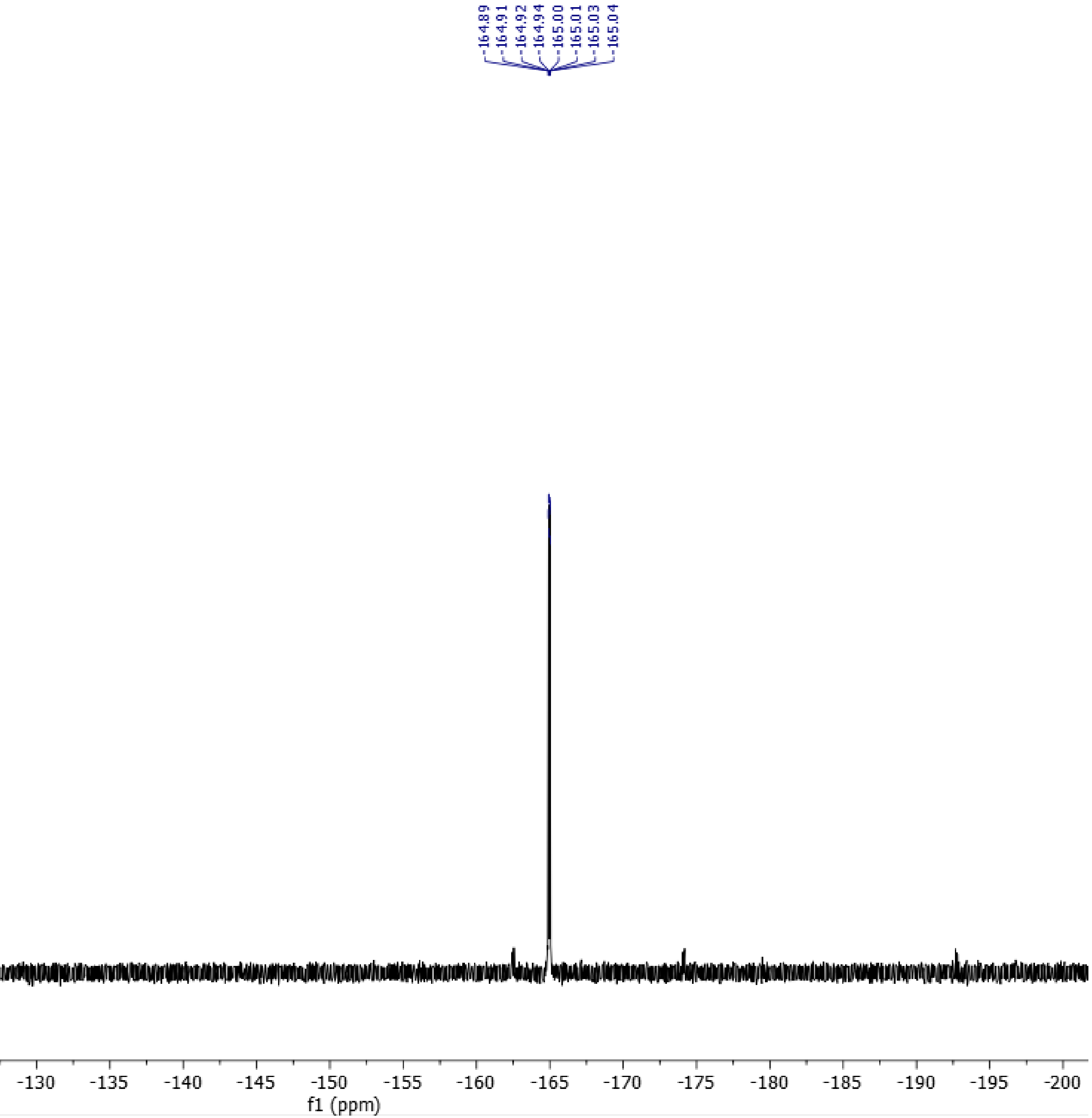

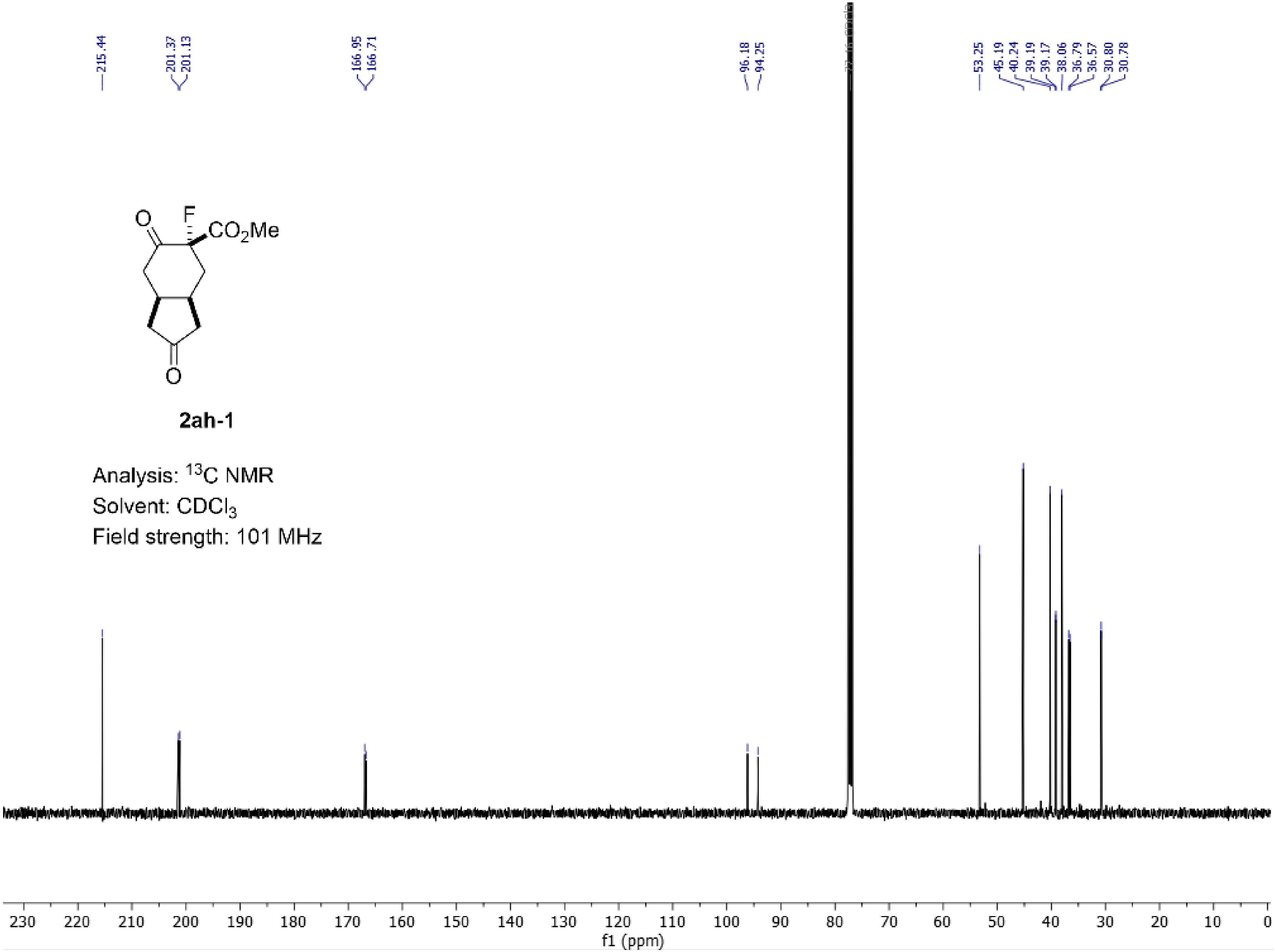

2ah-1

Analysis: ^{13}C NMR

Solvent: $CDCl_3$

Field strength: 101 MHz

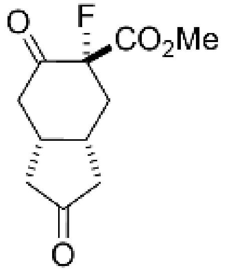

2ah-2

Analysis: ^{1}H NMR
Solvent: $CDCl_3$
Field strength: 400 MHz

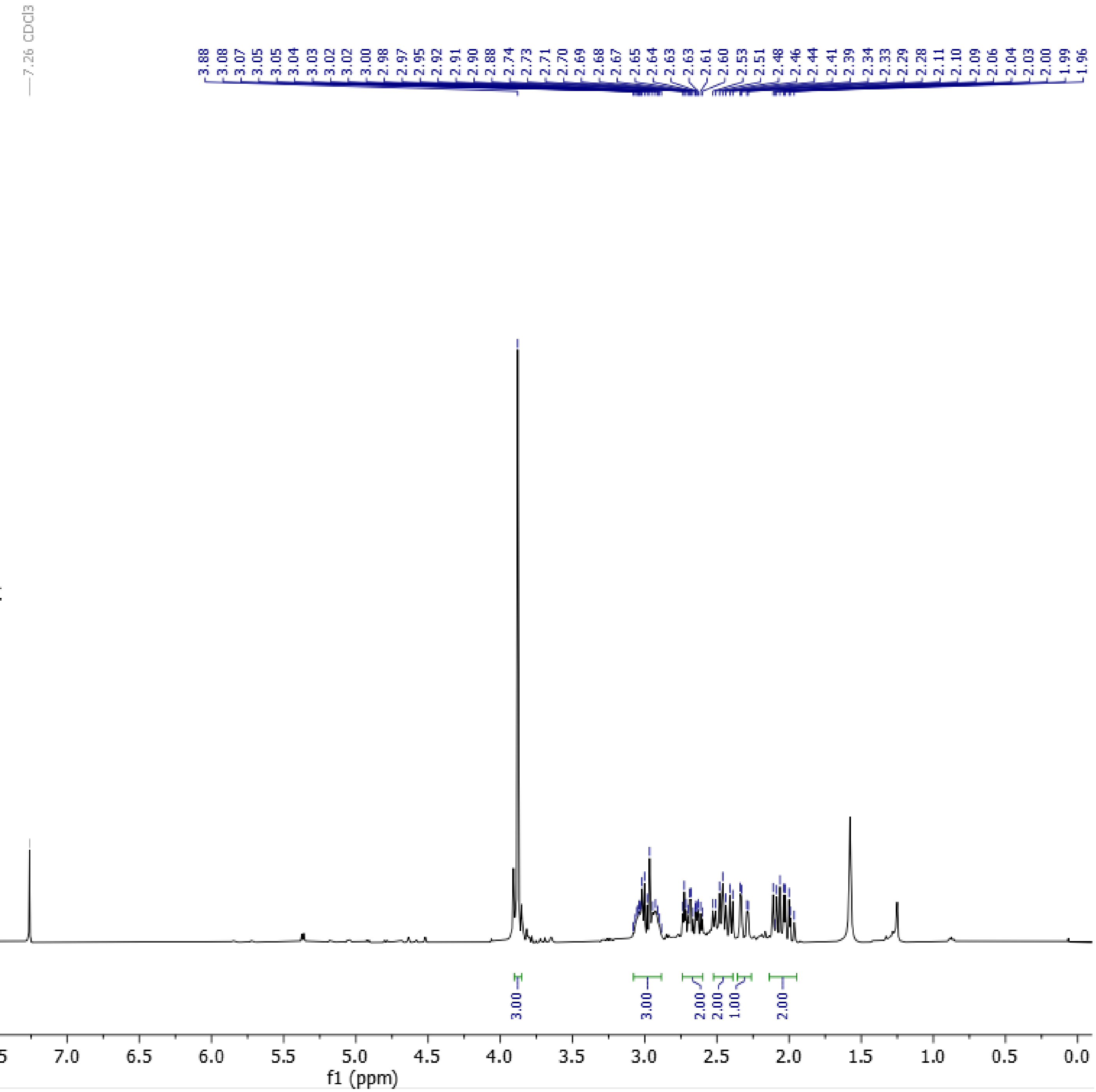

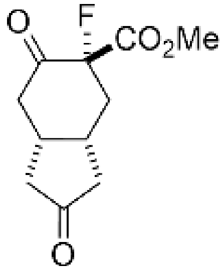

2ah-2

Analysis: ^{19}F NMR
Solvent: $CDCl_3$
Field strength: 377 MHz

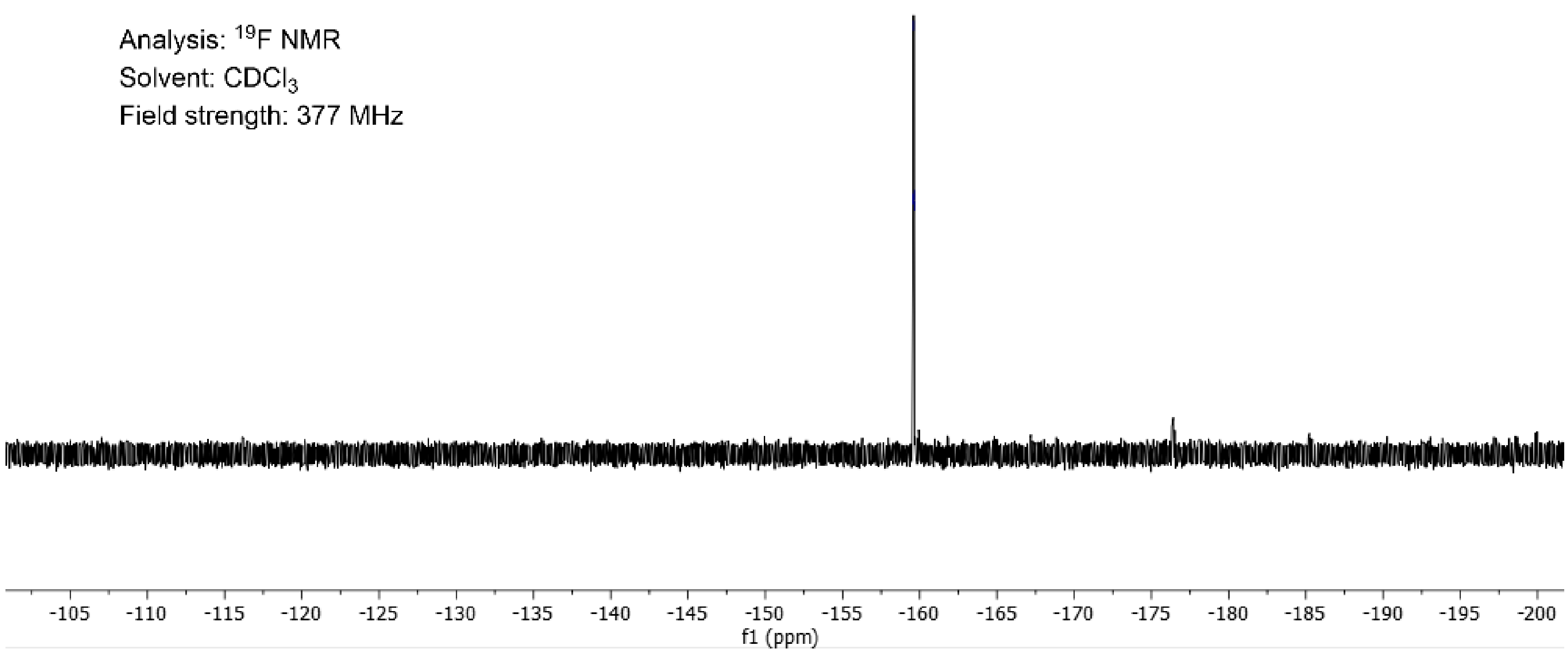

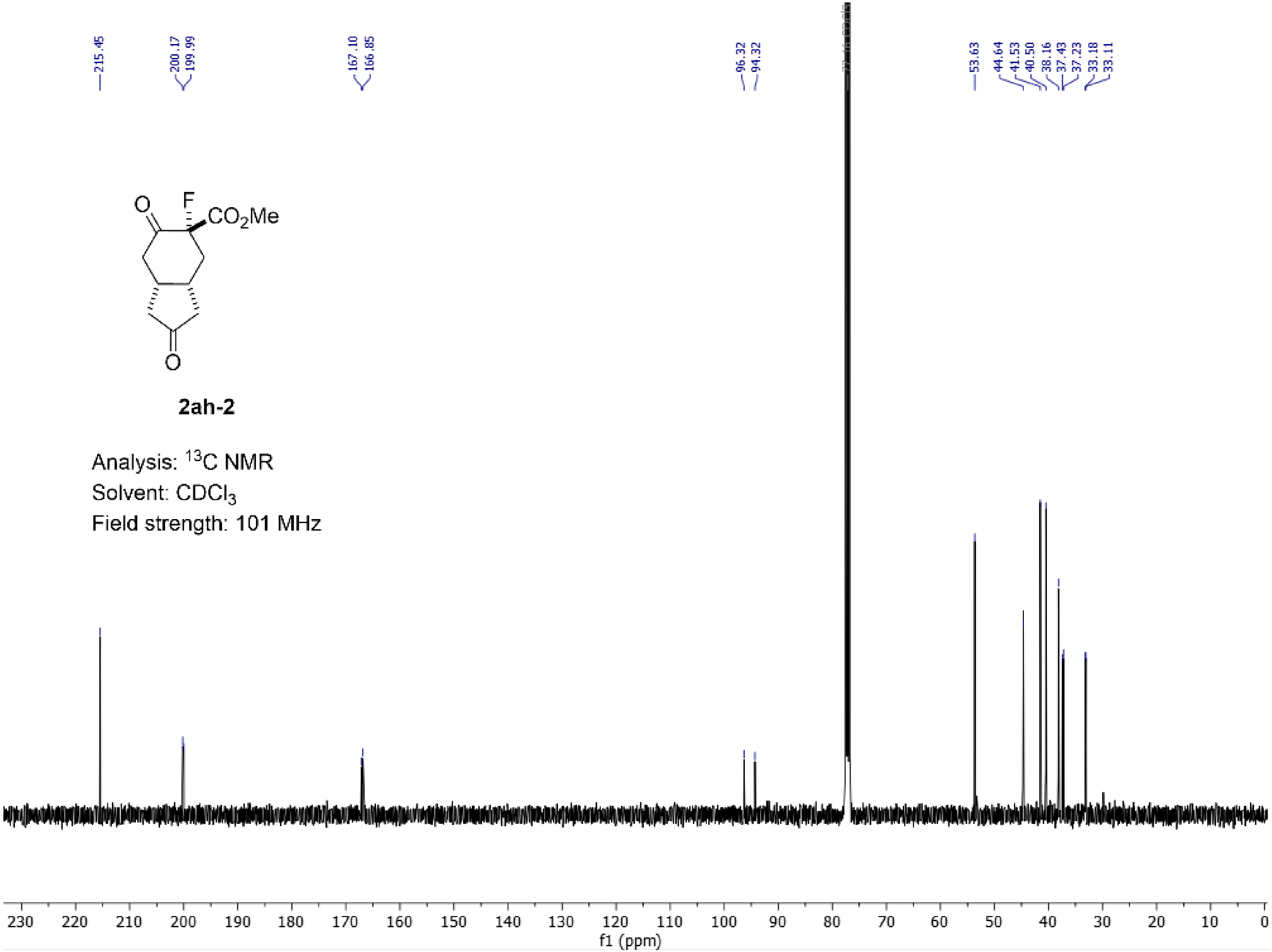
215.45
200.17
199.99
167.10
166.85
96.32
94.32
53.63
44.64
41.53
40.50
38.16
37.43
37.23
33.18
33.11
F
CO2Me
O
O
O
2ah-2
Analysis: 13C NMR
Solvent: CDCl3
Field strength: 101 MHz
230
220
210
200
190
180
170
160
150
140
130
120
110
100
90
80
70
60
50
40
30
20
10
0
f1 (ppm)

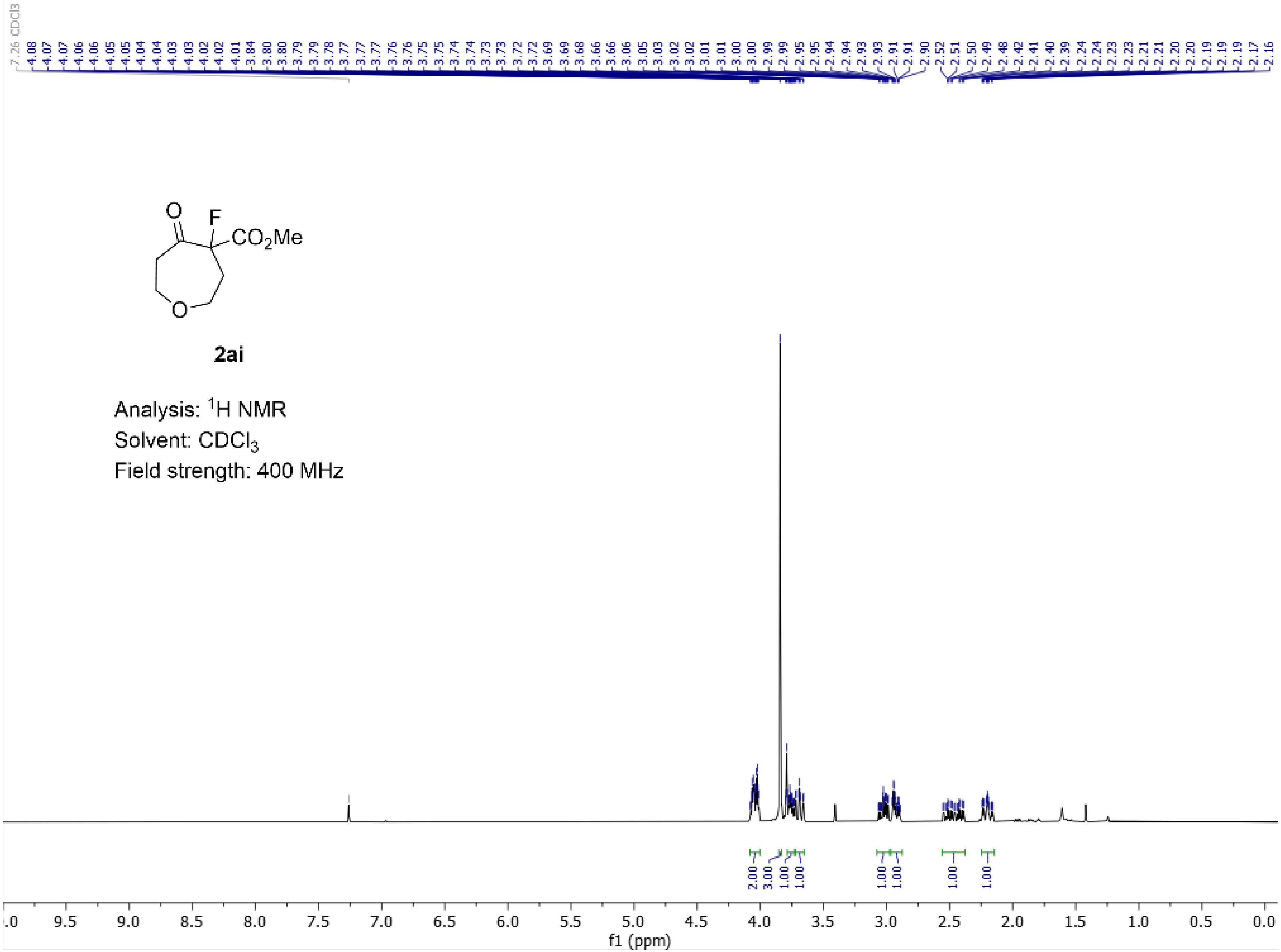

2ai

Analysis: ^{1}H NMR
Solvent: $CDCl_3$
Field strength: 400 MHz

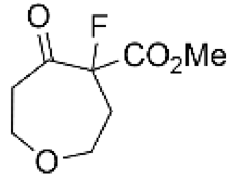

2ai

Analysis: ^{19}F NMR
Solvent: $CDCl_3$
Field strength: 377 MHz

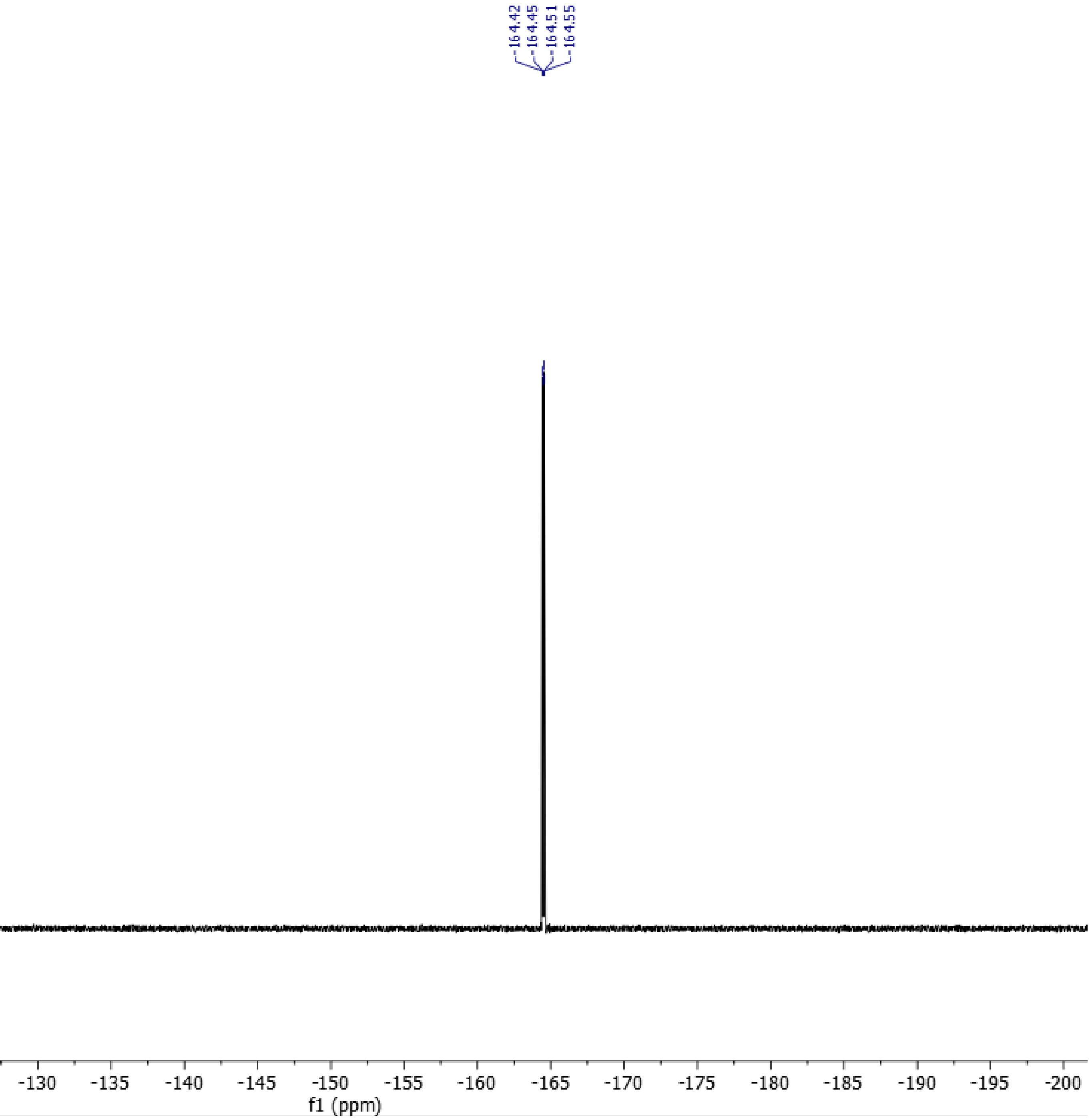

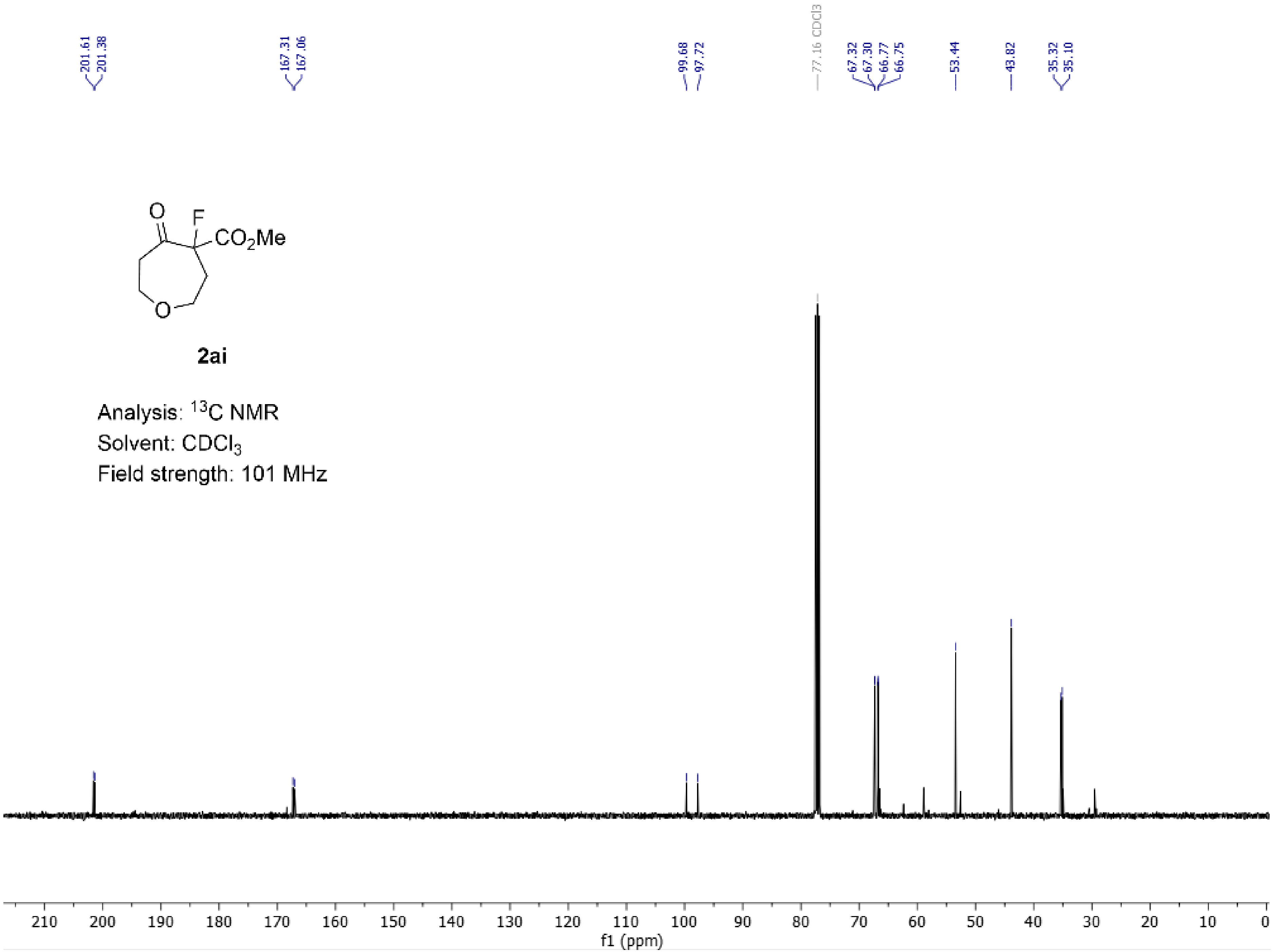

2ai

Analysis: ^{13}C NMR
Solvent: $CDCl_3$
Field strength: 101 MHz

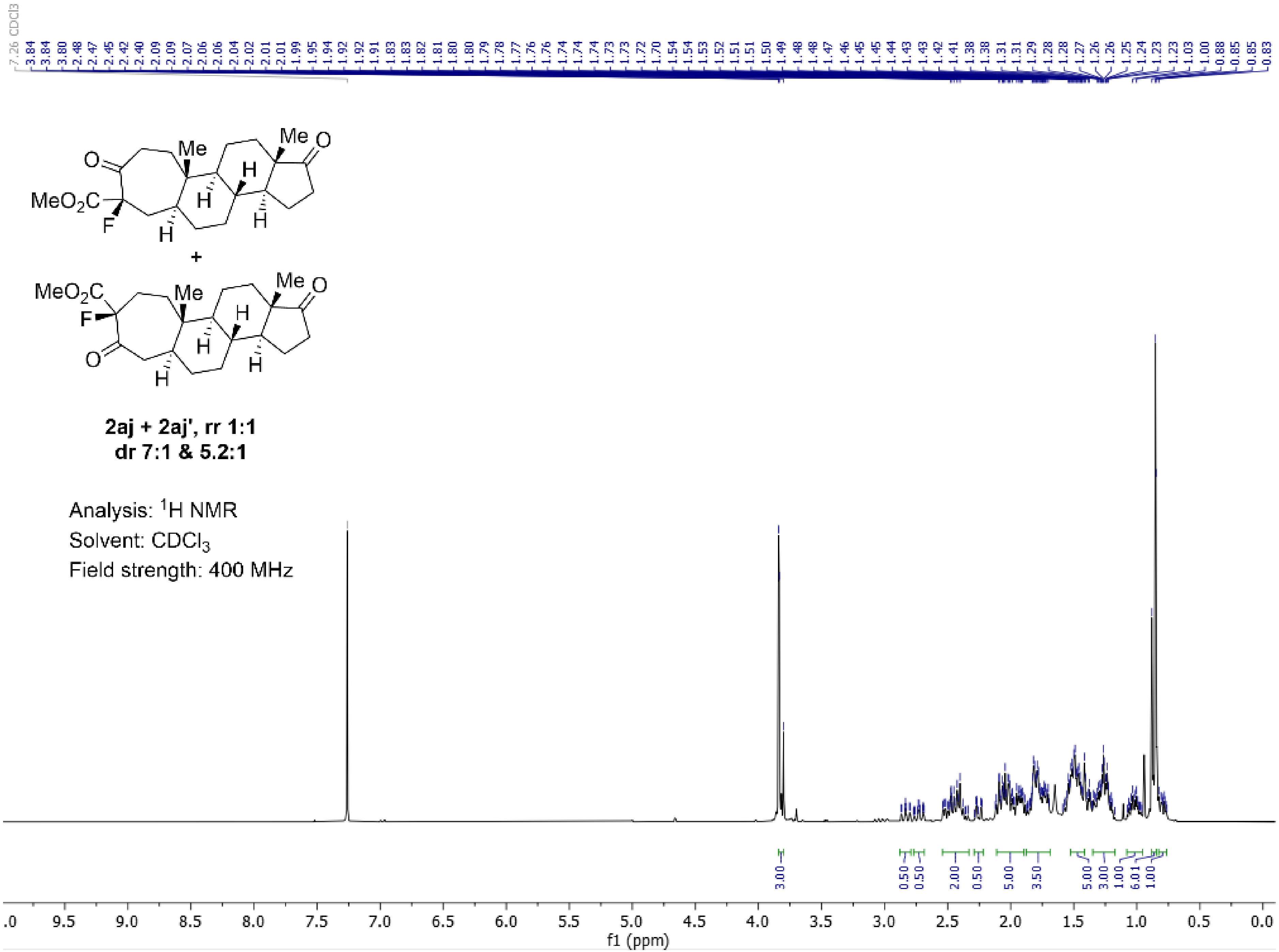

2aj + 2aj', rr 1:1
dr 7:1 & 5.2:1

Analysis: ^{1}H NMR
Solvent: $CDCl_3$
Field strength: 400 MHz

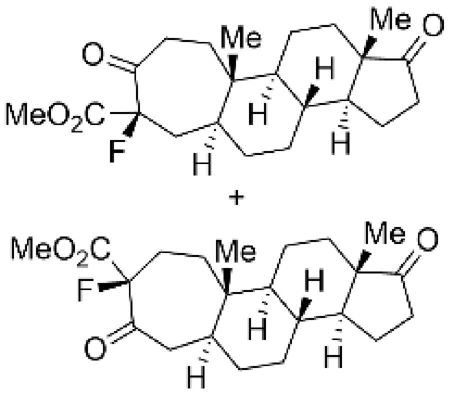

2aj + 2aj', rr 1:1
dr 7:1 & 5.2:1

Analysis: ^{19}F NMR
Solvent: $CDCl_3$
Field strength: 377 MHz

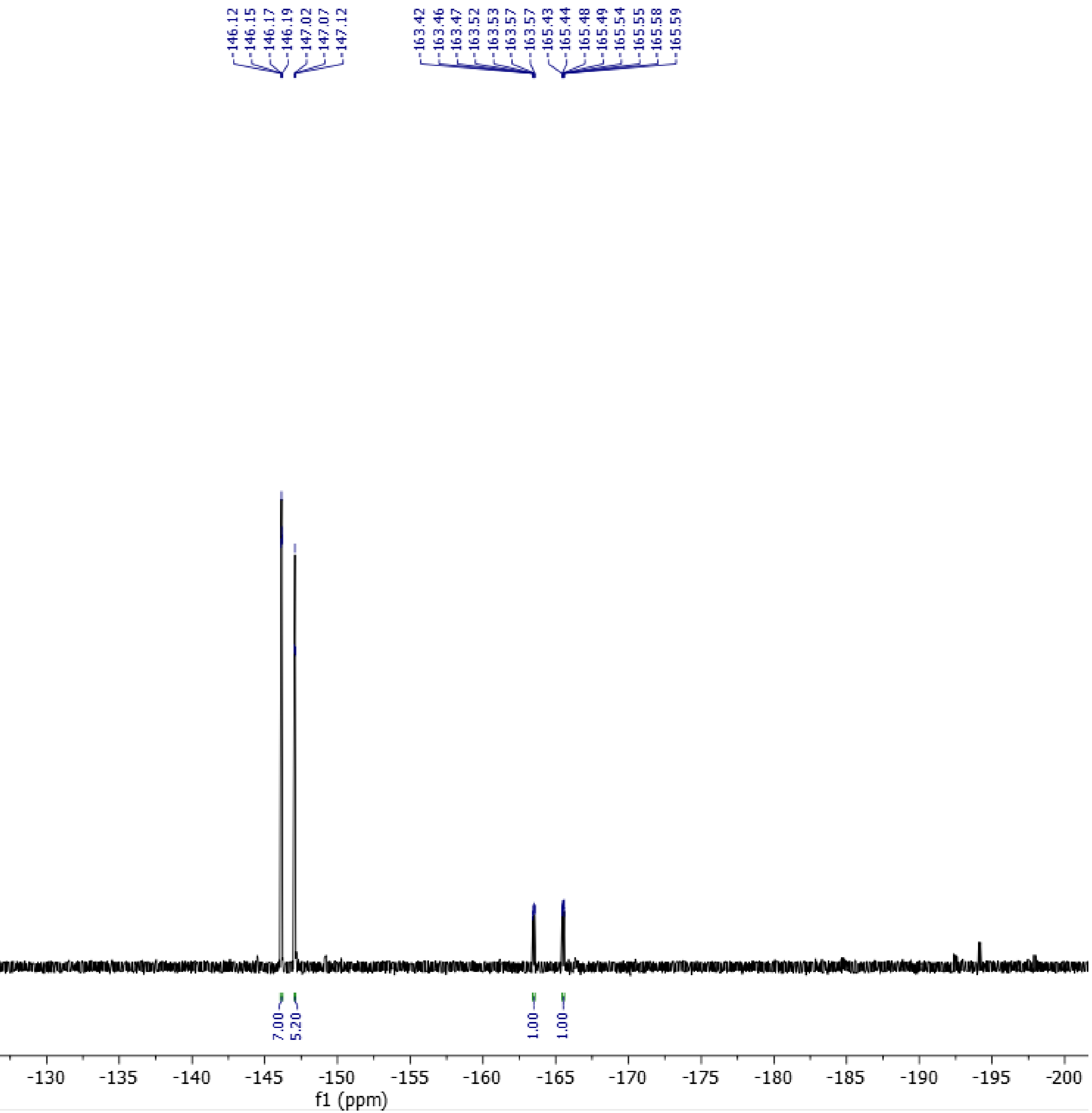

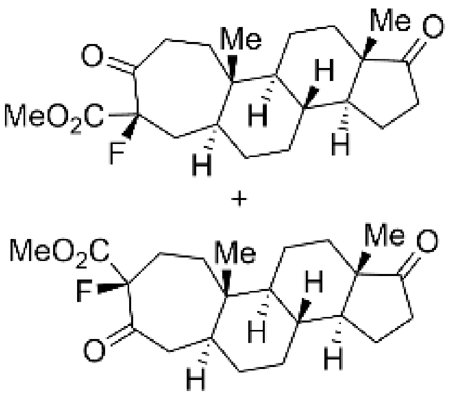

2aj + 2aj', rr 1:1
dr 7:1 & 5.2:1

Analysis: ^{13}C NMR
Solvent: $CDCl_3$
Field strength: 101 MHz

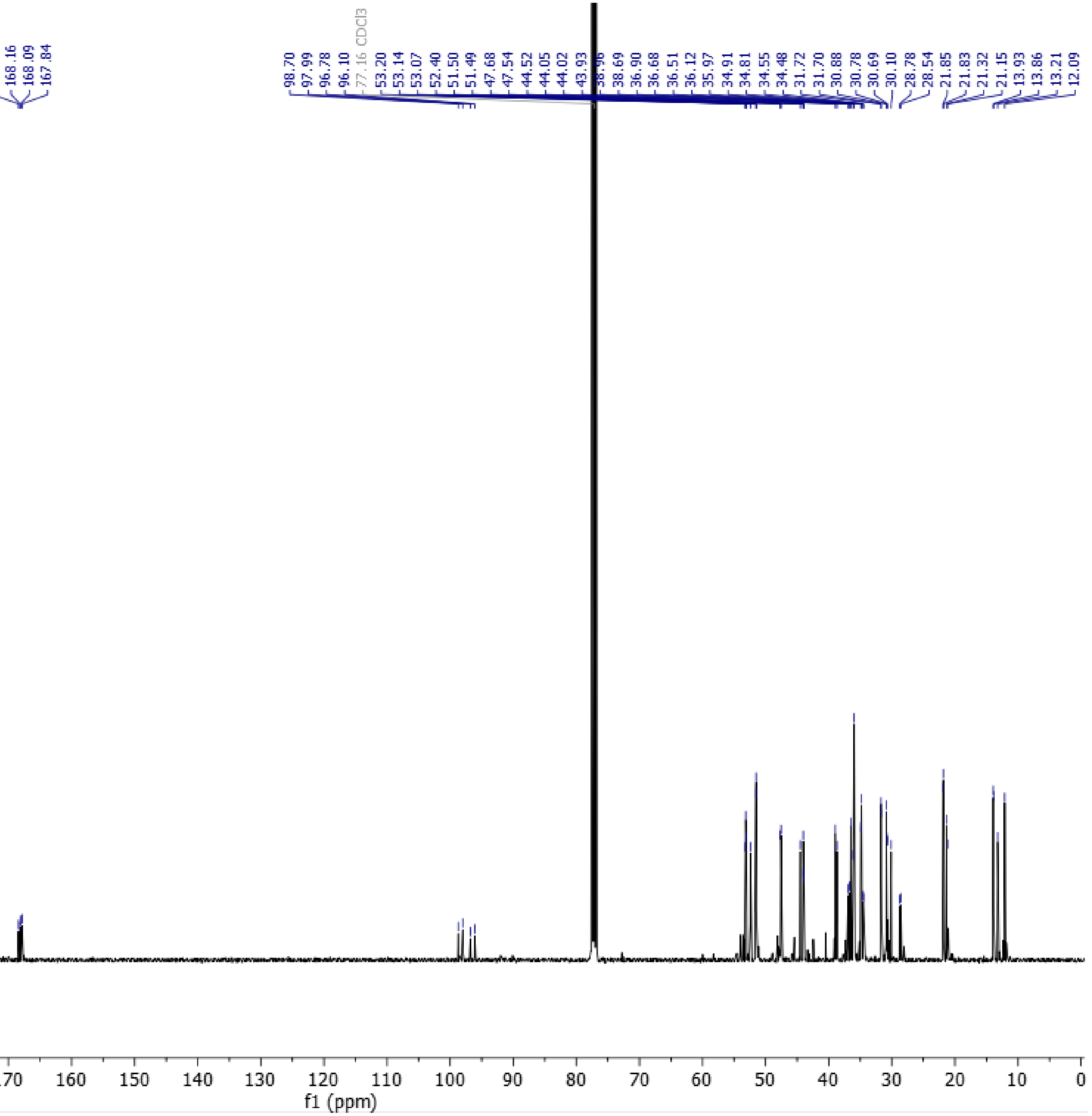

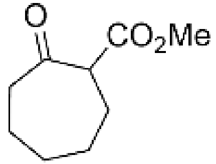

3a, keto:enol 9:1

Analysis: ^{1}H NMR
Solvent: $CDCl_3$
Field strength: 400 MHz

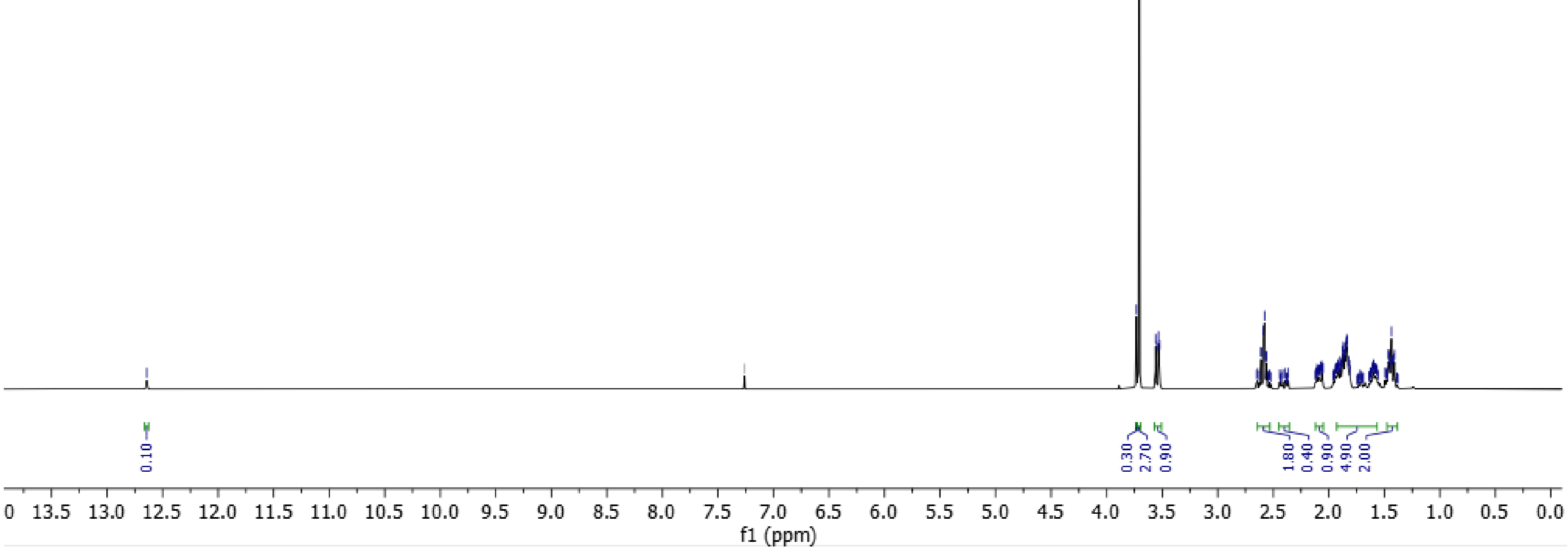

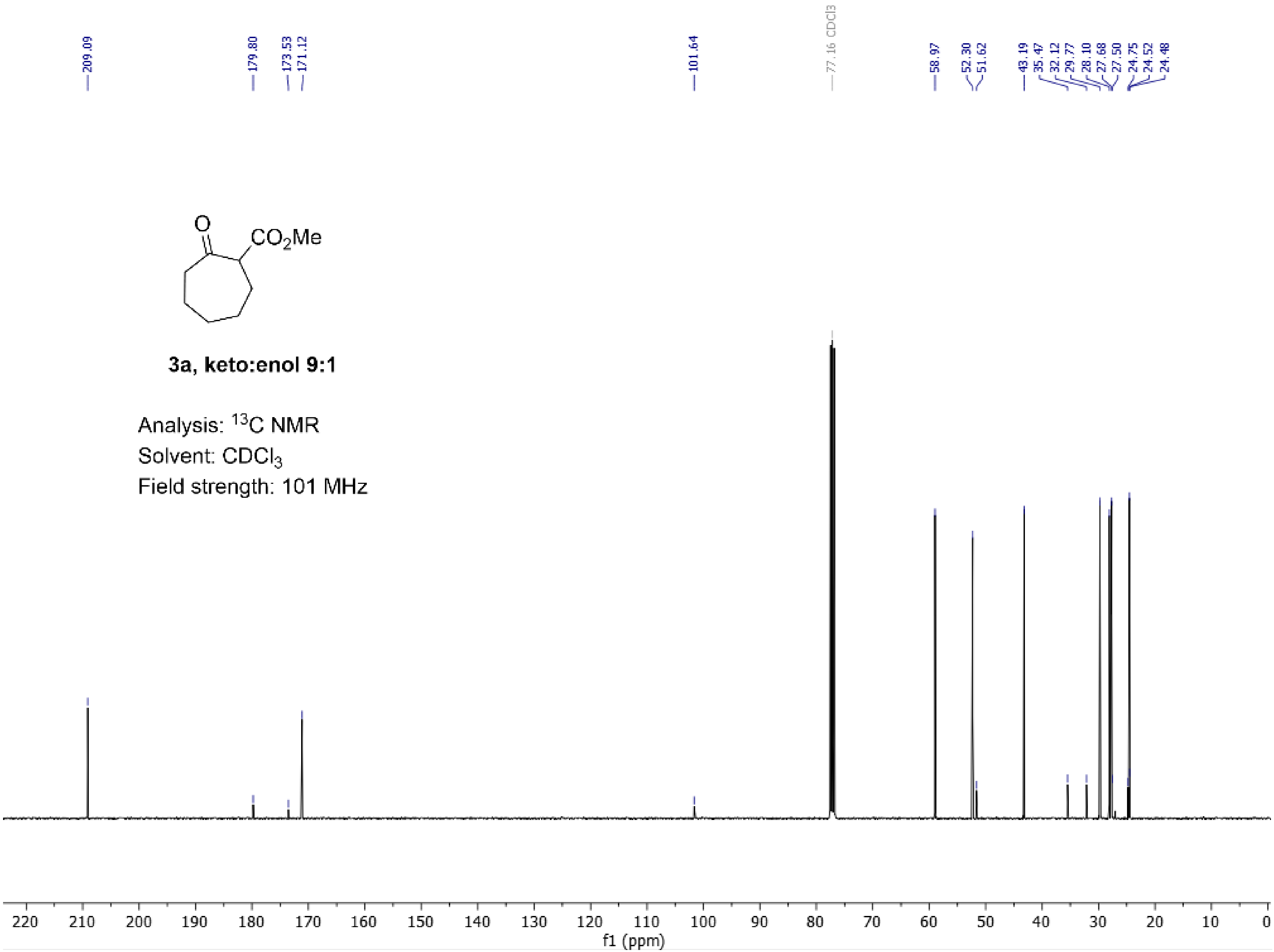

3a, keto:enol 9:1

Analysis: ^{13}C NMR
Solvent: $CDCl_3$
Field strength: 101 MHz

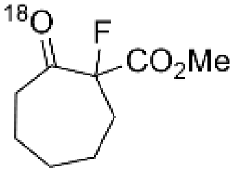

2a-^{18}O/^{16}O 64/36

Analysis: ^{1}H NMR
Solvent: $CDCl_3$
Field strength: 600 MHz

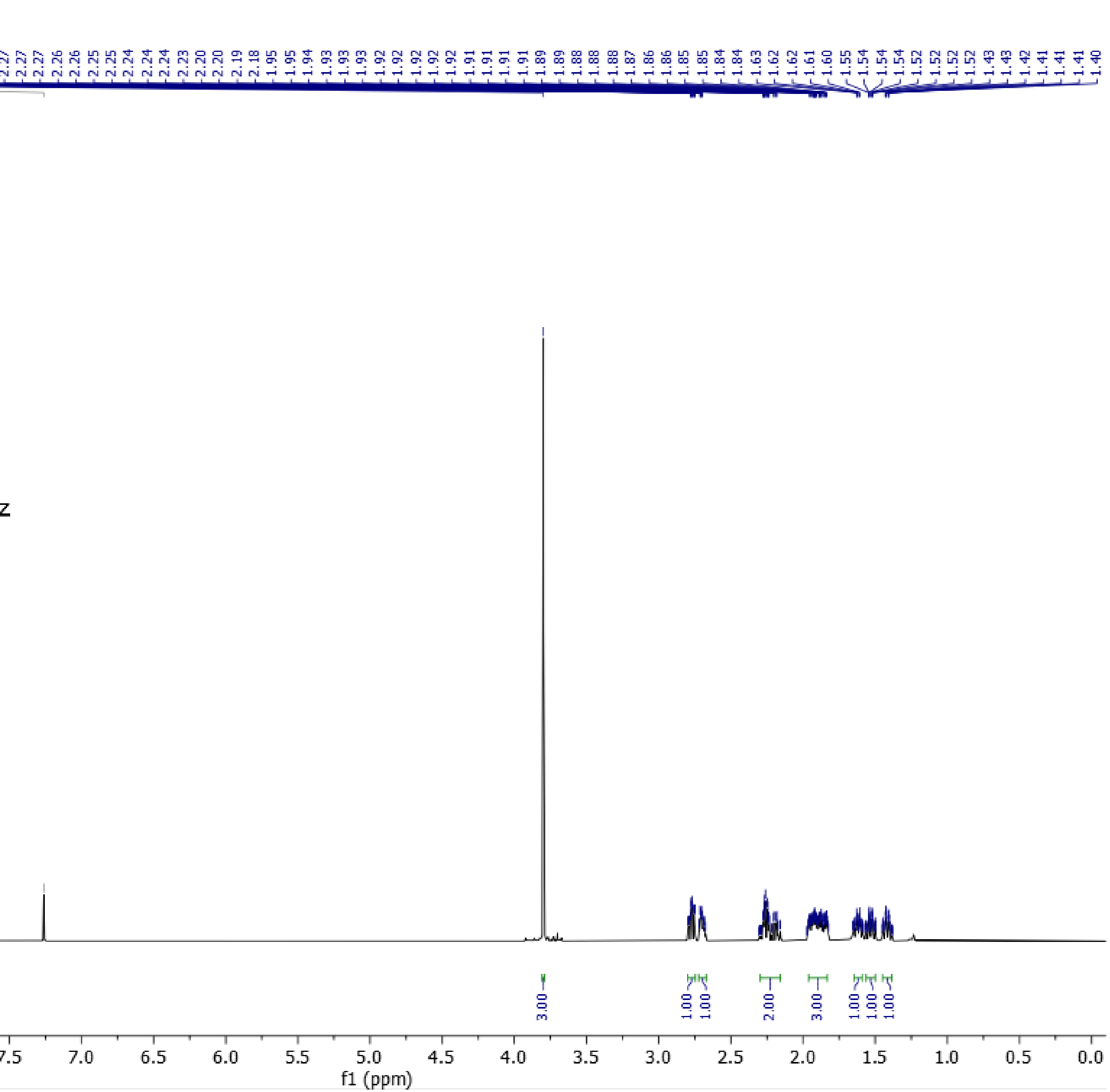

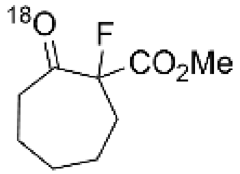

2a-^{18}O/^{16}O 64/36

Analysis: ^{19}F {^{1}H} NMR
Solvent: $CDCl_3$
Field strength: 376 MHz

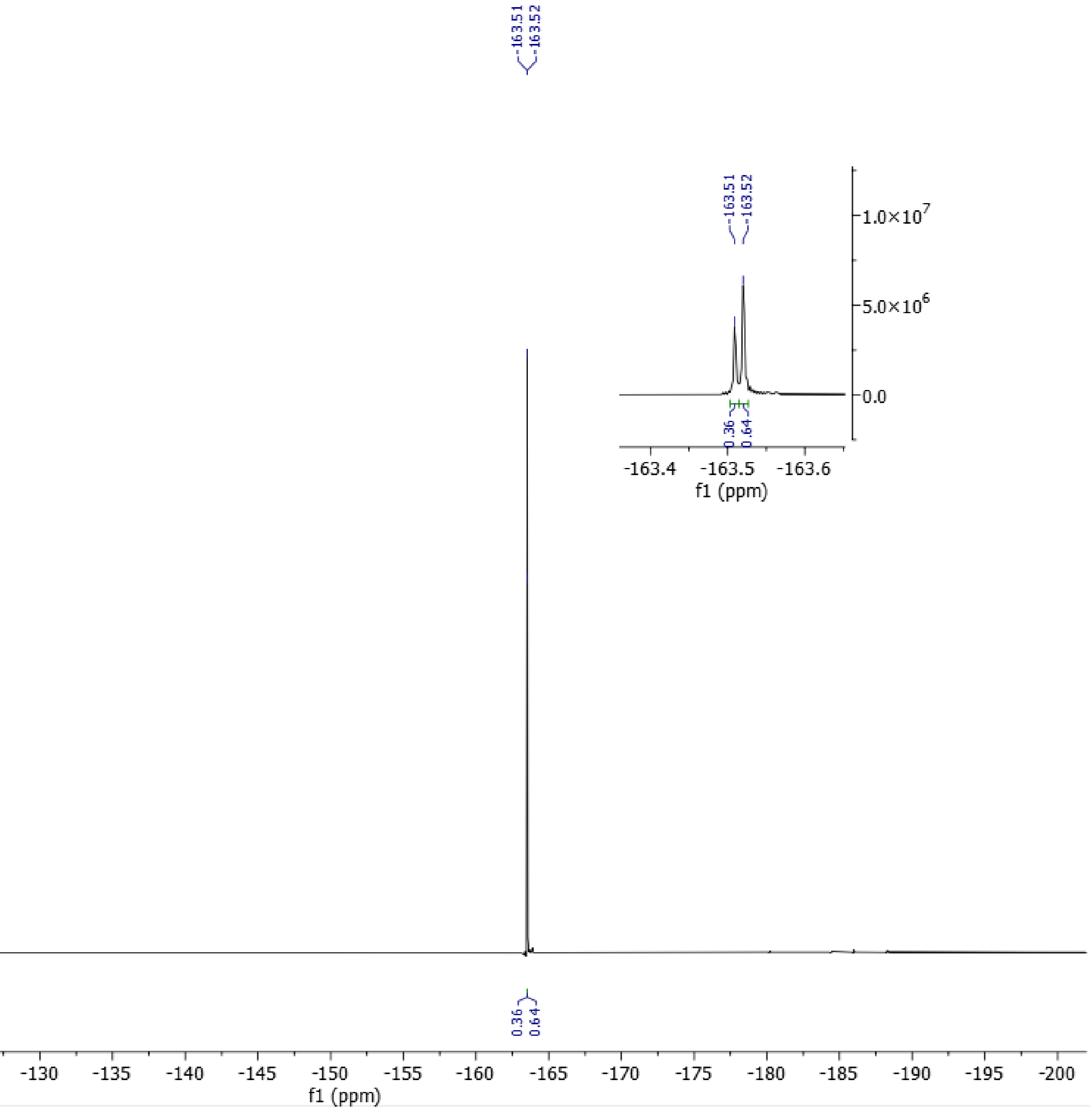

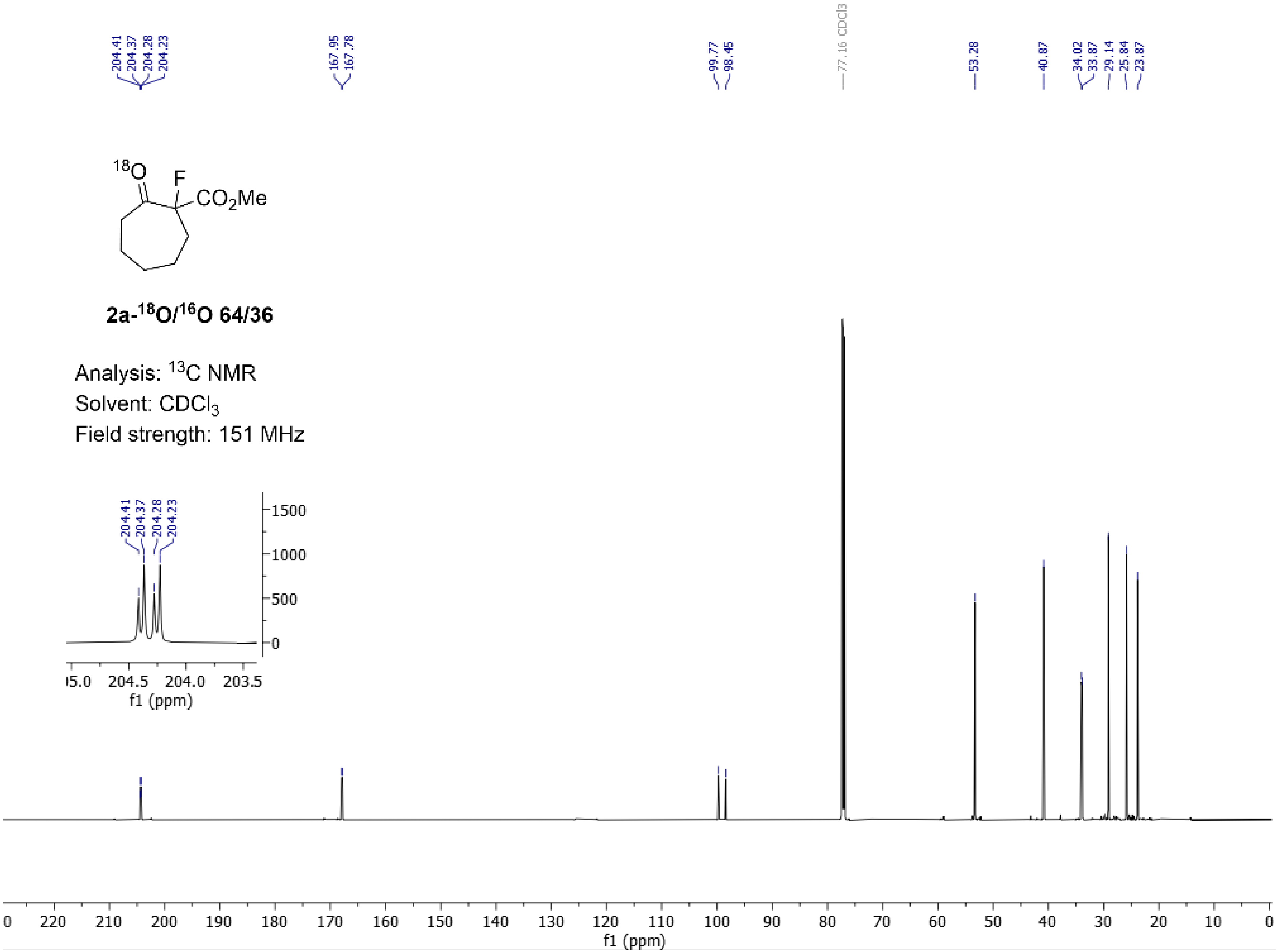

2a-^{18}O/^{16}O 64/36

Analysis: ^{13}C NMR
Solvent: $CDCl_3$
Field strength: 151 MHz

2a-$^{18}O/^{16}O$ 64/36

Analysis: ^{13}C {^{1}H, ^{19}F} NMR
Solvent: $CDCl_3$
Field strength: 101 MHz

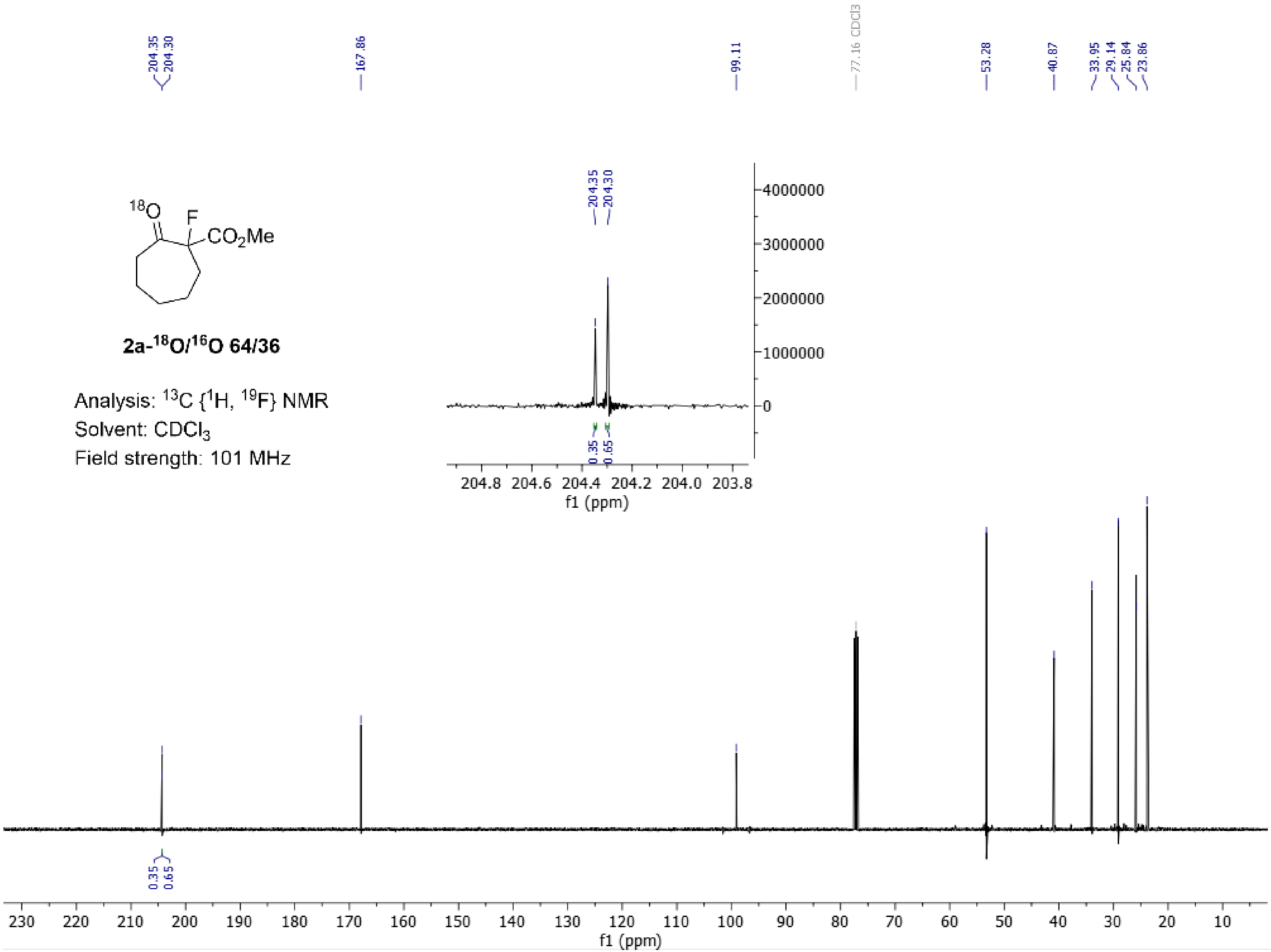

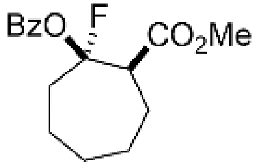

5

Analysis: ^{1}H NMR
Solvent: $CDCl_3$
Field strength: 400 MHz

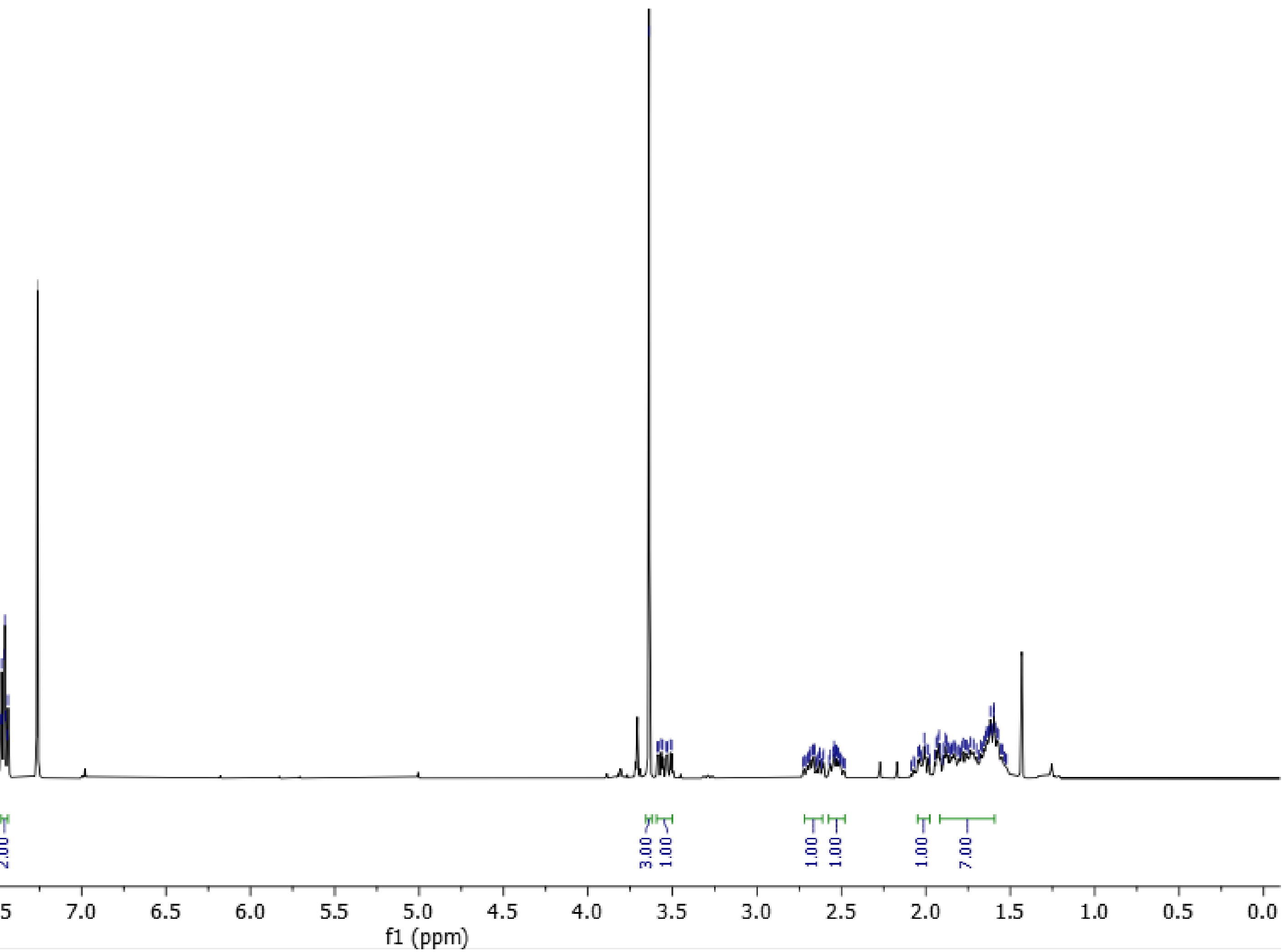

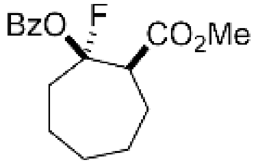

5

Analysis: ^{19}F NMR
Solvent: $CDCl_3$
Field strength: 376 MHz

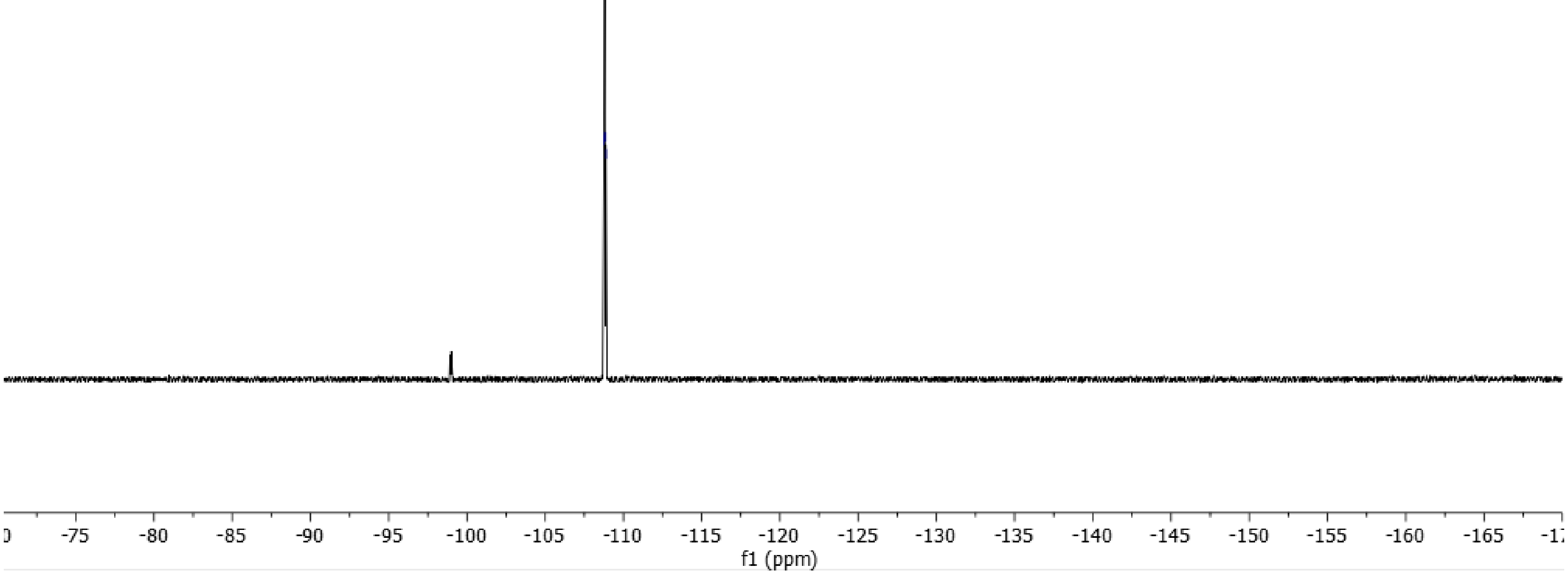

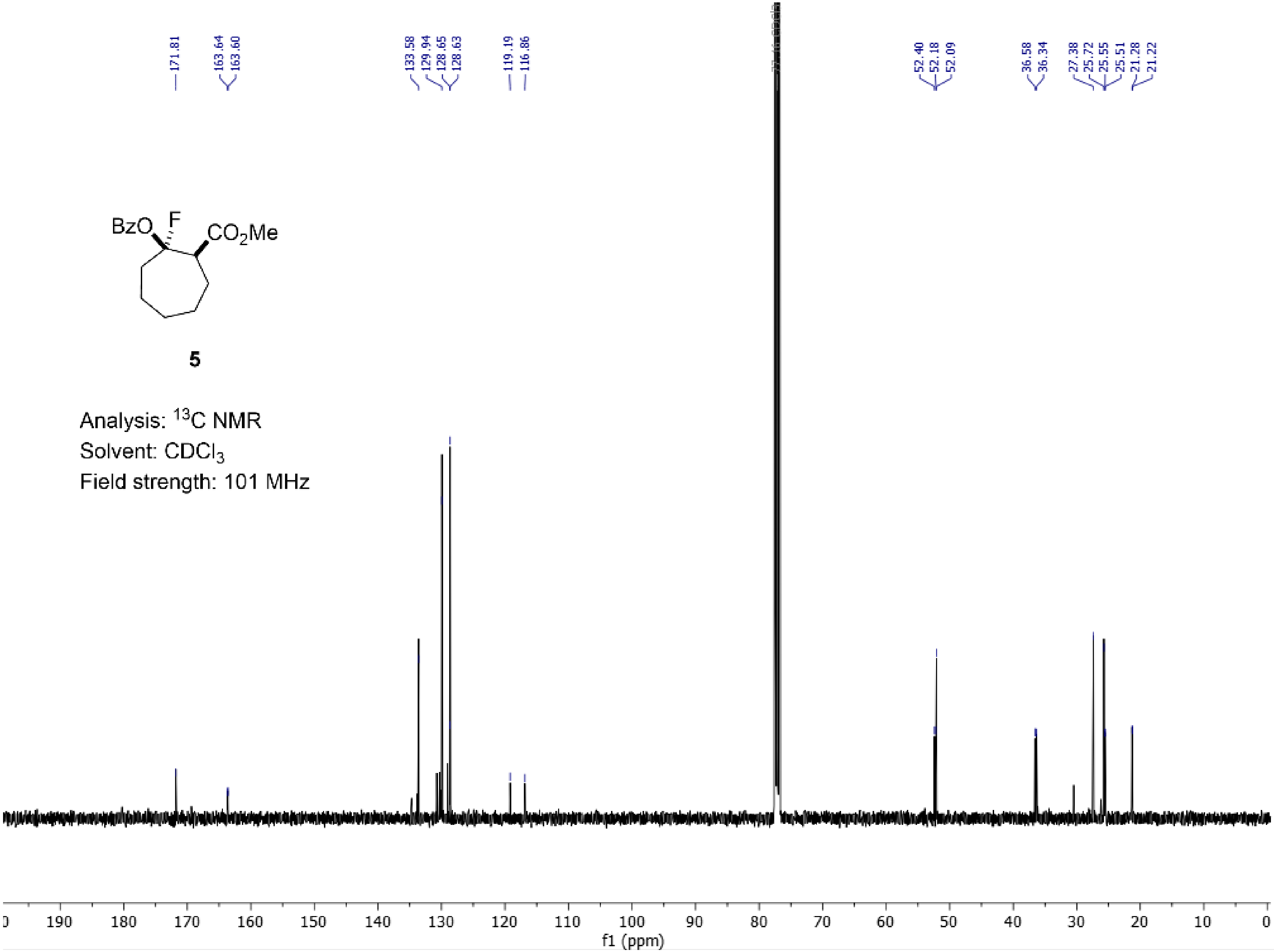

Analysis: ^{13}C NMR
Solvent: $CDCl_3$
Field strength: 101 MHz

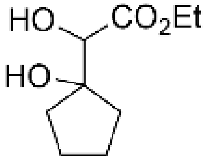

4j

Analysis: ^{1}H NMR
Solvent: $CDCl_3$
Field strength: 400 MHz

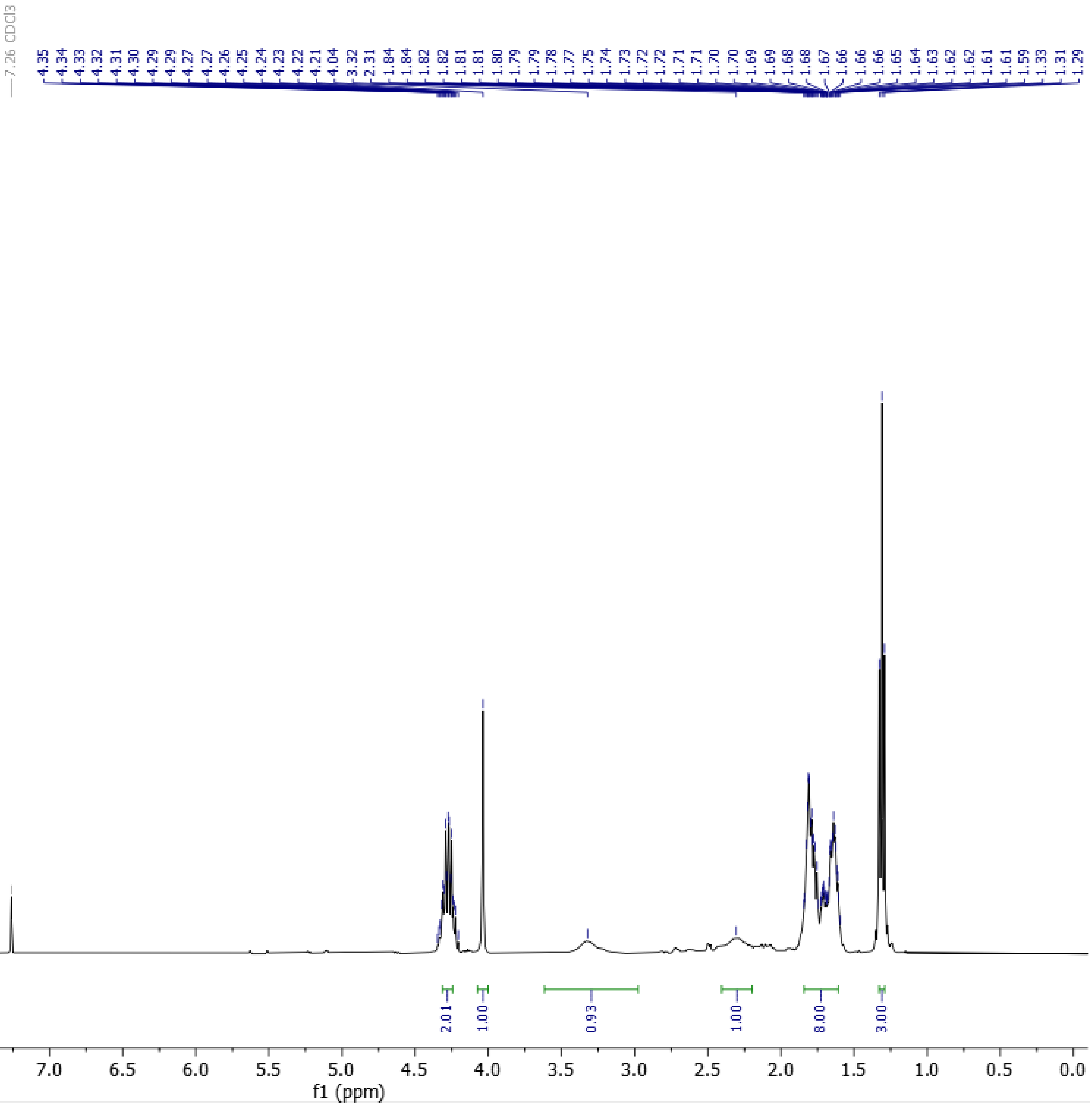

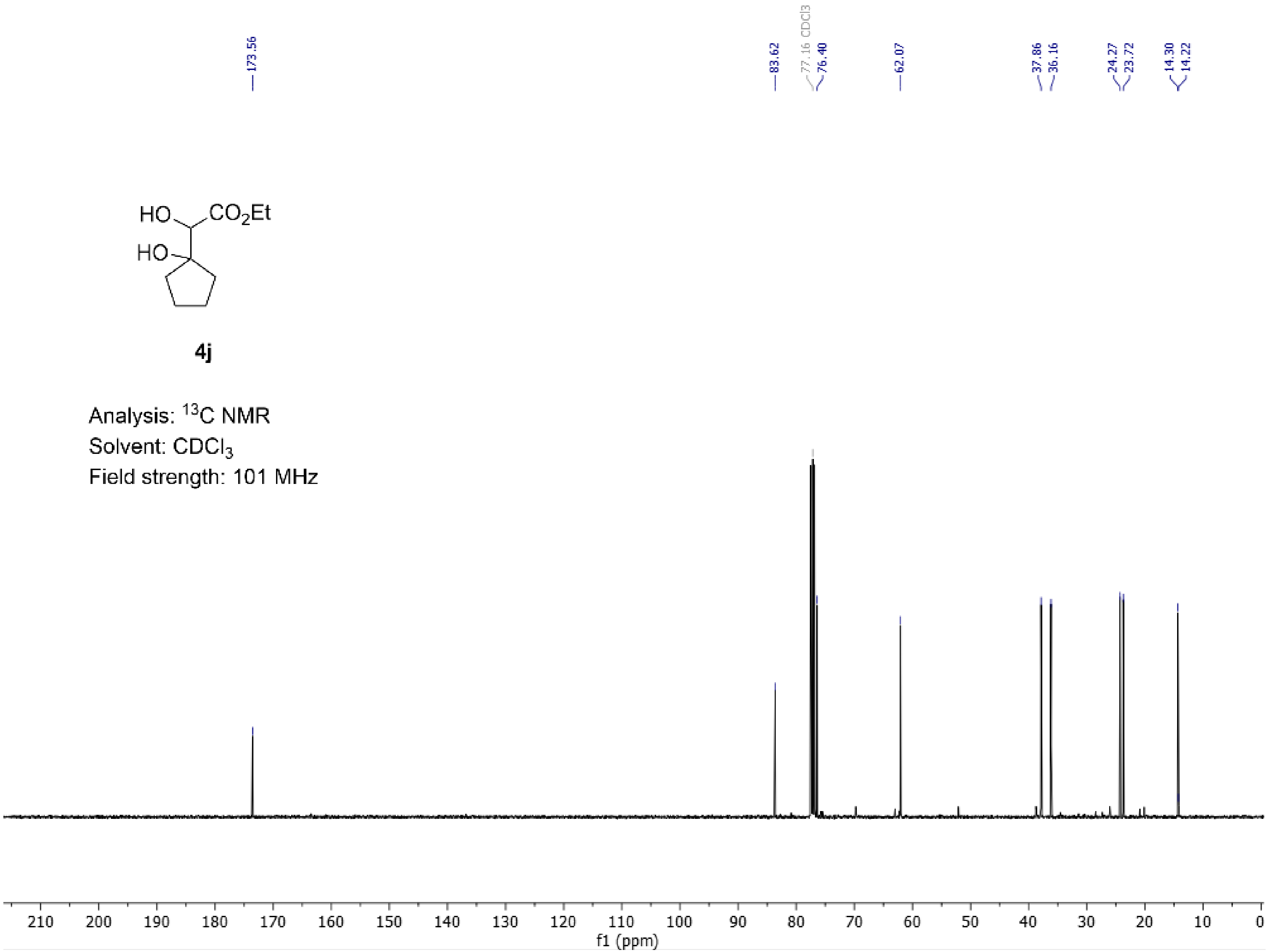
173.56
83.62
77.16 CDCl3
76.40
62.07
37.86
36.16
24.27
23.72
14.30
14.22
HO
CO2Et
HO
4j
Analysis: 13C NMR
Solvent: CDCl3
Field strength: 101 MHz
210 200 190 180 170 160 150 140 130 120 110 100 90 80 70 60 50 40 30 20 10 0
f1 (ppm)

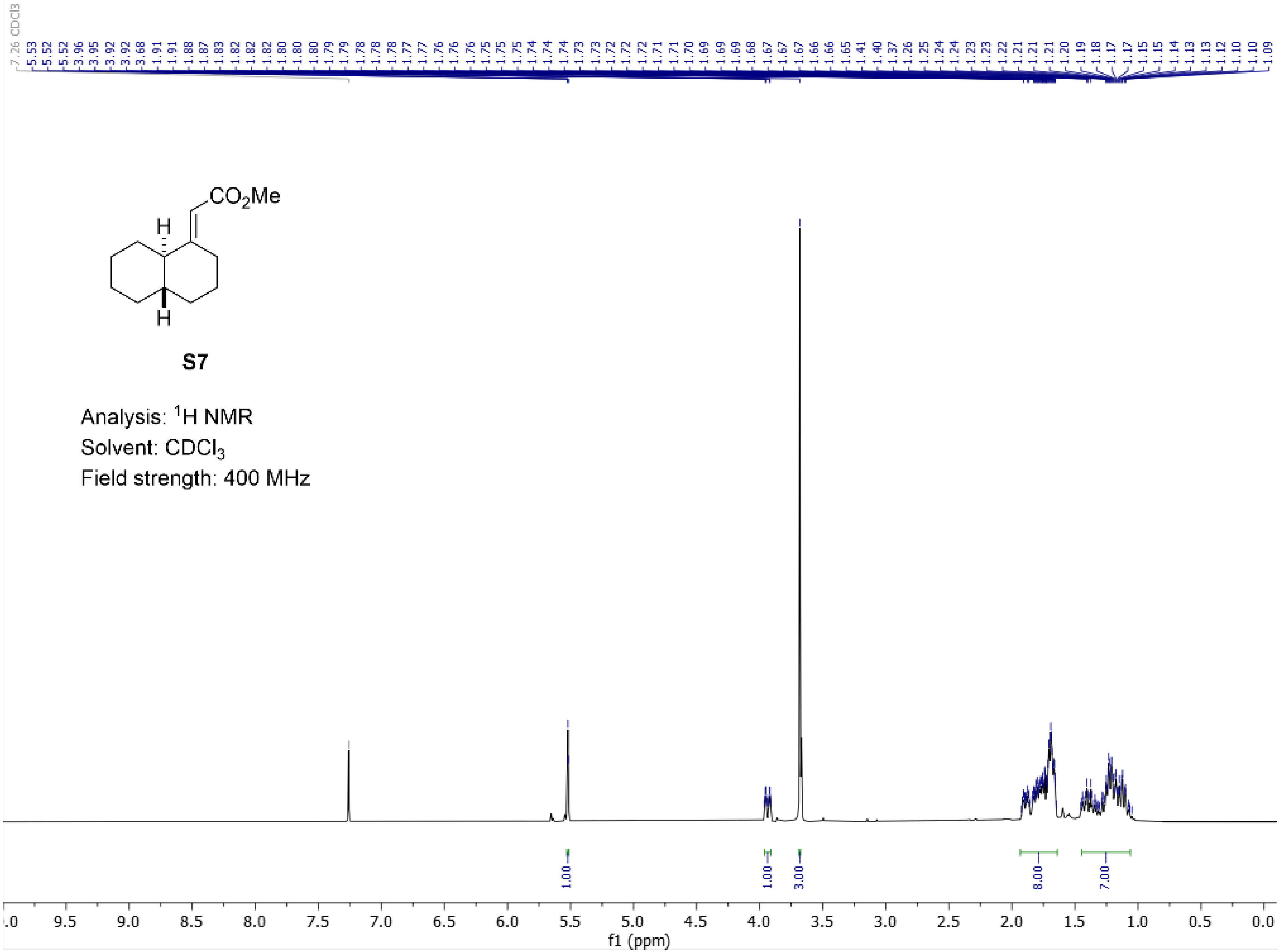
7.26 CDCl3
5.53 5.52 3.96 3.95 3.92 3.92 3.68 1.91 1.91 1.88 1.87 1.83 1.82 1.82 1.82 1.80 1.80 1.80 1.79 1.79 1.78 1.78 1.78 1.77 1.77 1.76 1.76 1.75 1.75 1.75 1.74 1.74 1.74 1.73 1.73 1.72 1.72 1.72 1.71 1.71 1.70 1.69 1.69 1.69 1.68 1.67 1.67 1.67 1.66 1.66 1.65 1.41 1.40 1.37 1.26 1.25 1.24 1.24 1.23 1.23 1.22 1.21 1.21 1.21 1.20 1.19 1.18 1.17 1.17 1.15 1.15 1.14 1.13 1.13 1.12 1.10 1.09
CO2Me
H
H
S7
Analysis: 1H NMR
Solvent: CDCl3
Field strength: 400 MHz
1.00
1.00
3.00
8.00
7.00
.0 9.5 9.0 8.5 8.0 7.5 7.0 6.5 6.0 5.5 5.0 4.5 4.0 3.5 3.0 2.5 2.0 1.5 1.0 0.5 0.0
f1 (ppm)

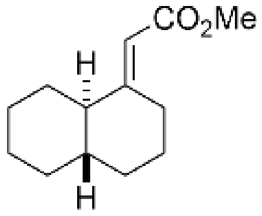

S7

Analysis: ^{13}C NMR
Solvent: $CDCl_3$
Field strength: 101 MHz

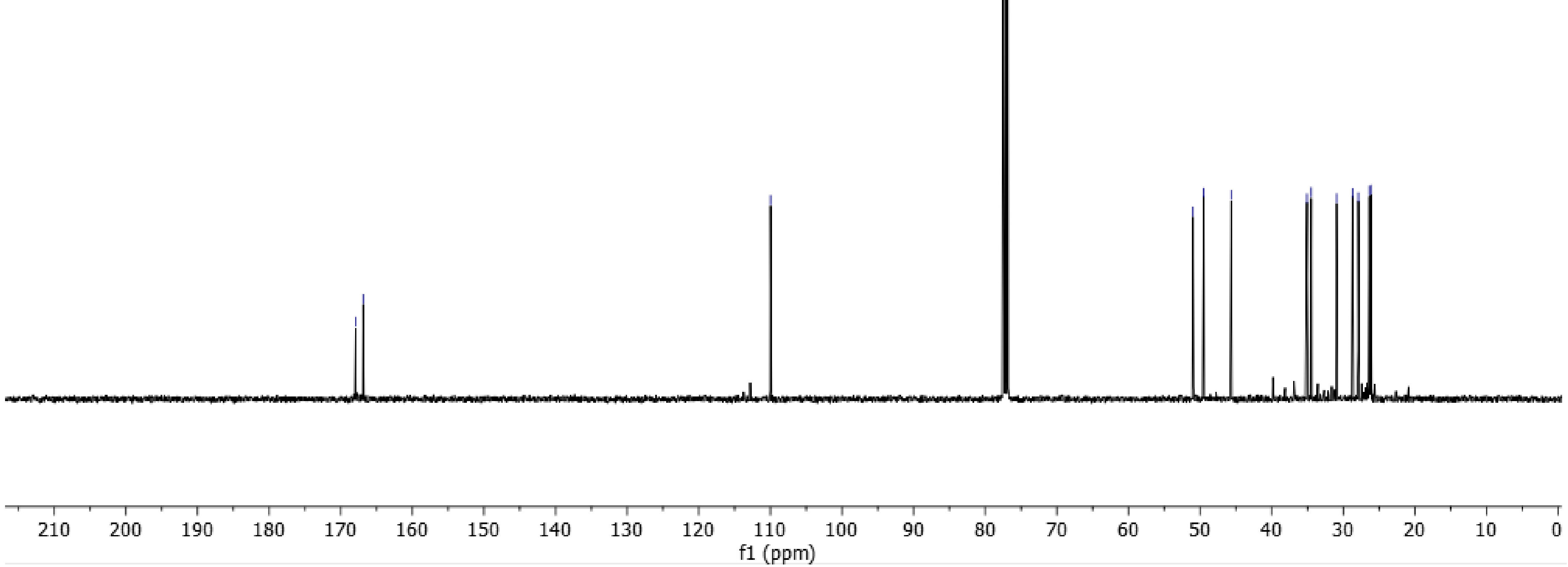

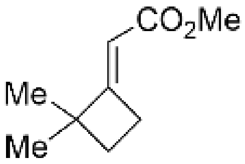

S8

Analysis: ^{1}H NMR
Solvent: $CDCl_3$
Field strength: 400 MHz

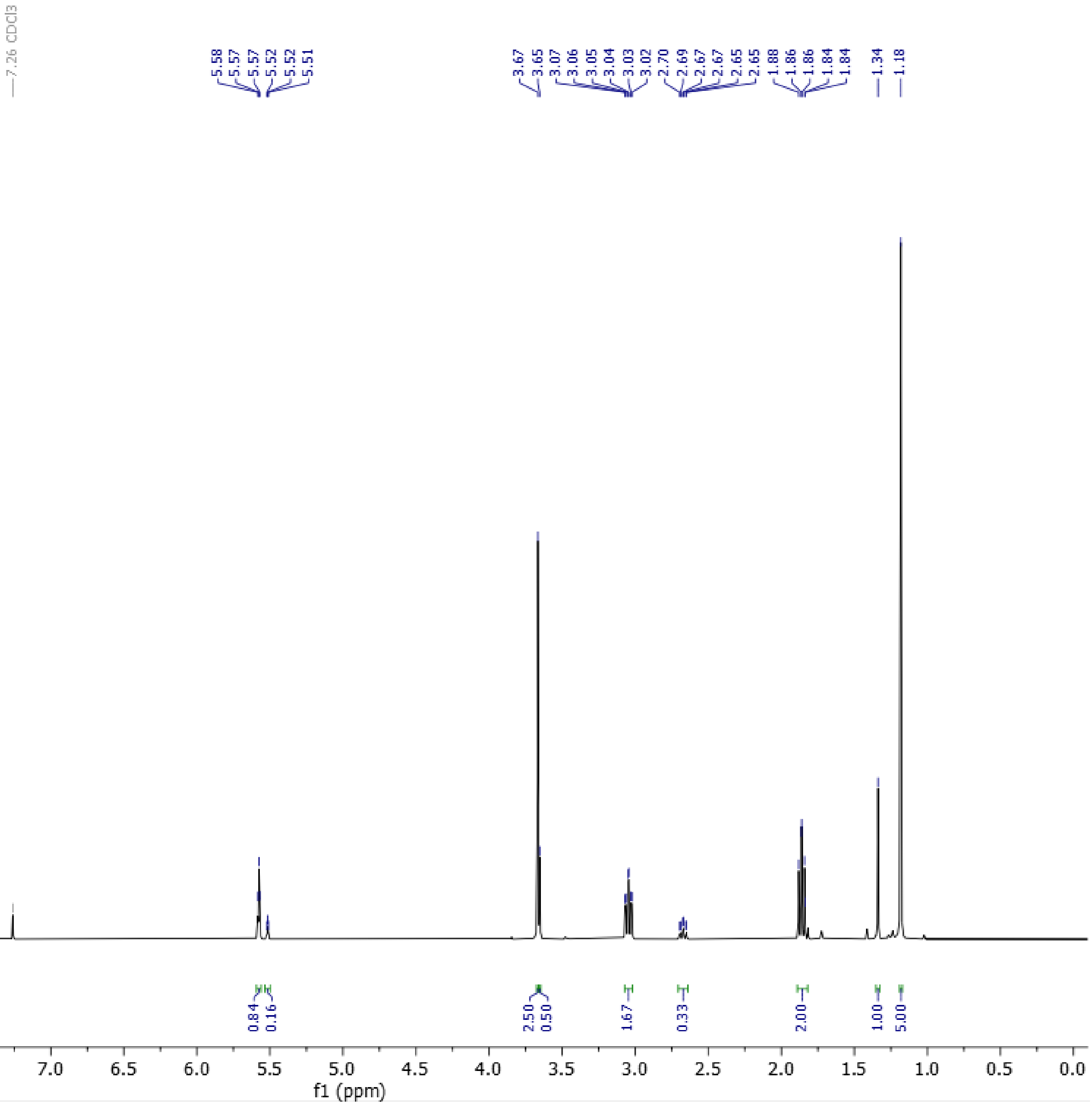

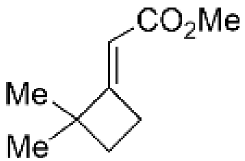

S8

Analysis: ^{13}C NMR
Solvent: $CDCl_3$
Field strength: 101 MHz

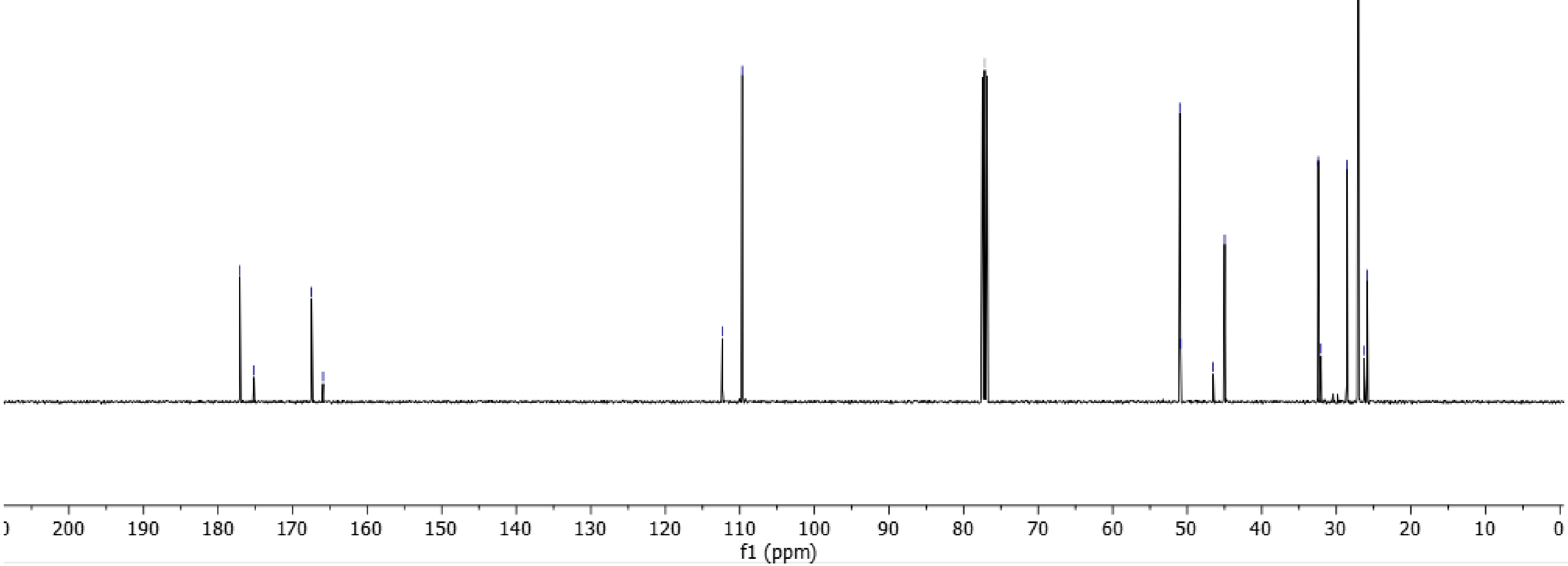

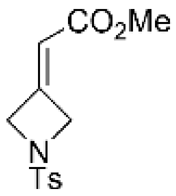

S9

Analysis: ^{1}H NMR
Solvent: $CDCl_3$
Field strength: 400 MHz

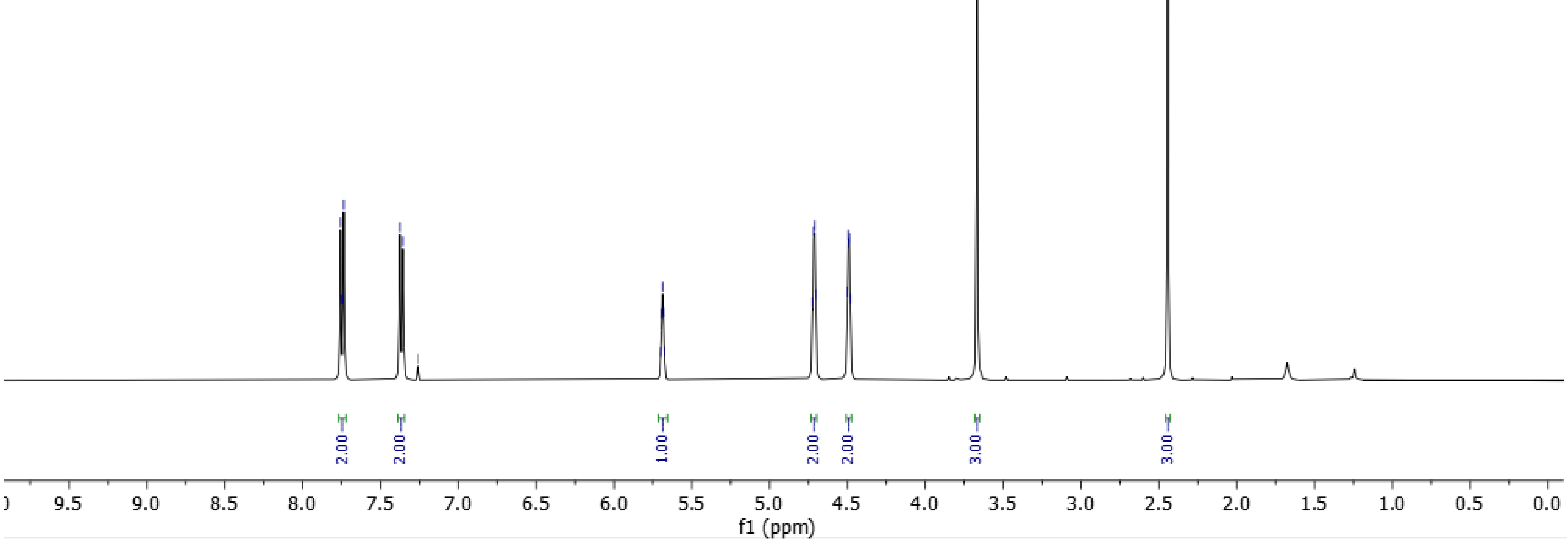

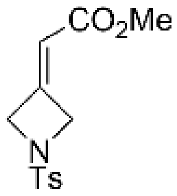

S9

Analysis: ^{13}C NMR
Solvent: $CDCl_3$
Field strength: 101 MHz

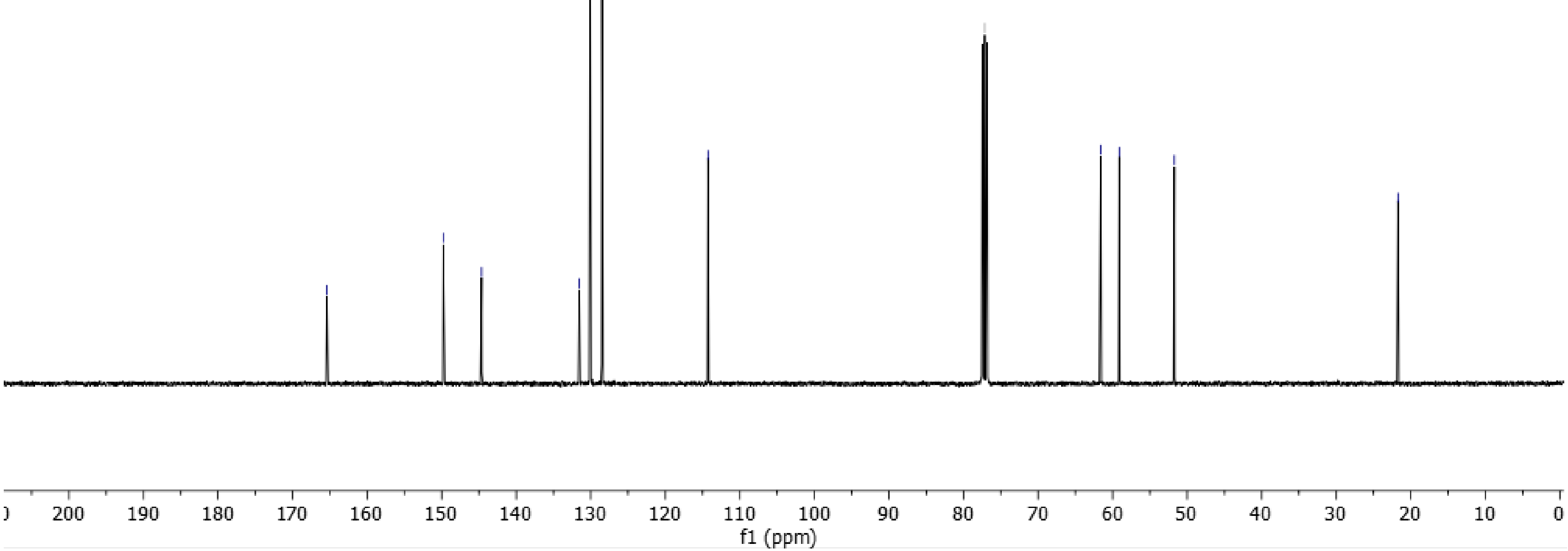

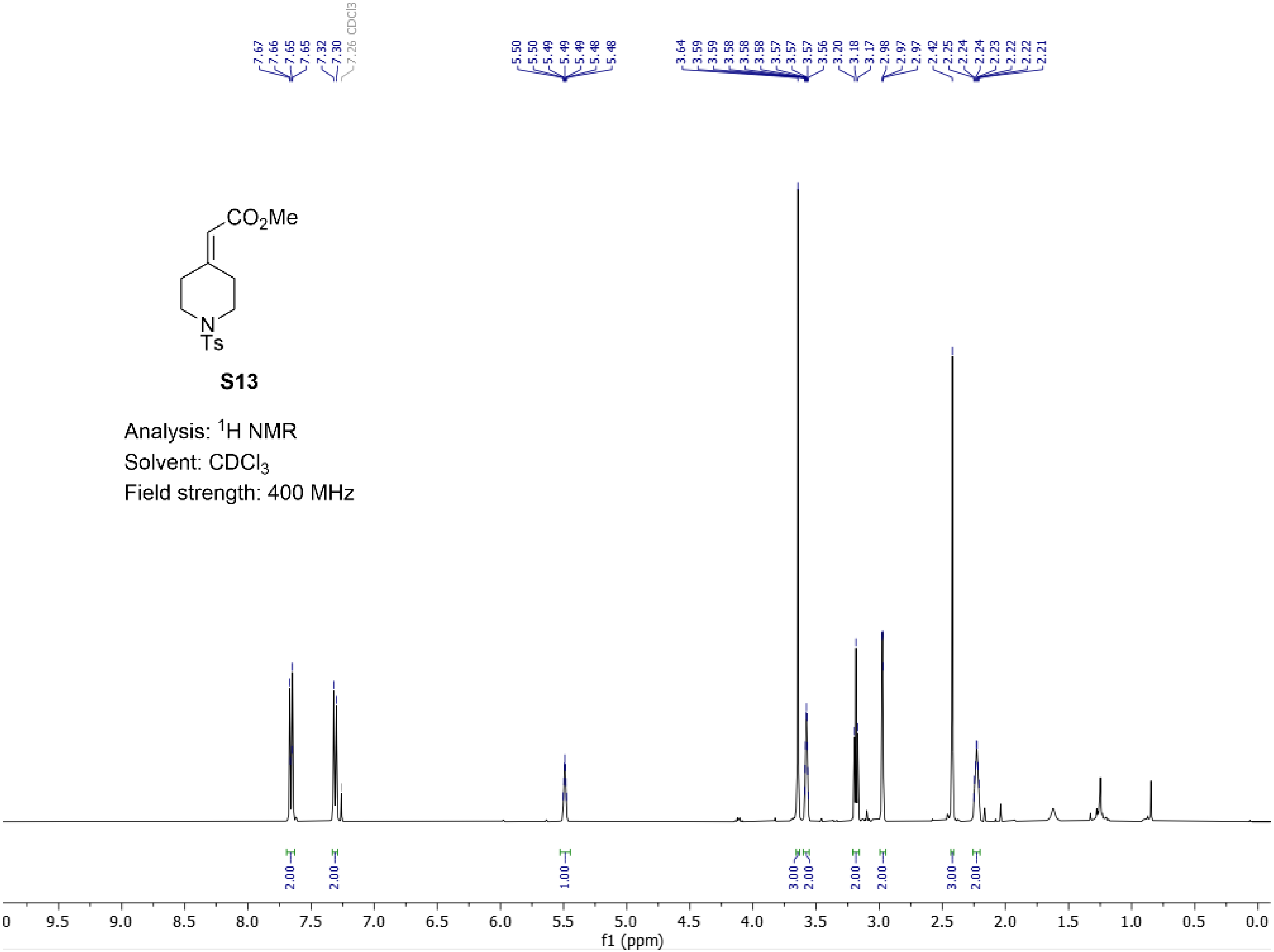

S13

Analysis: ^{1}H NMR
Solvent: $CDCl_3$
Field strength: 400 MHz

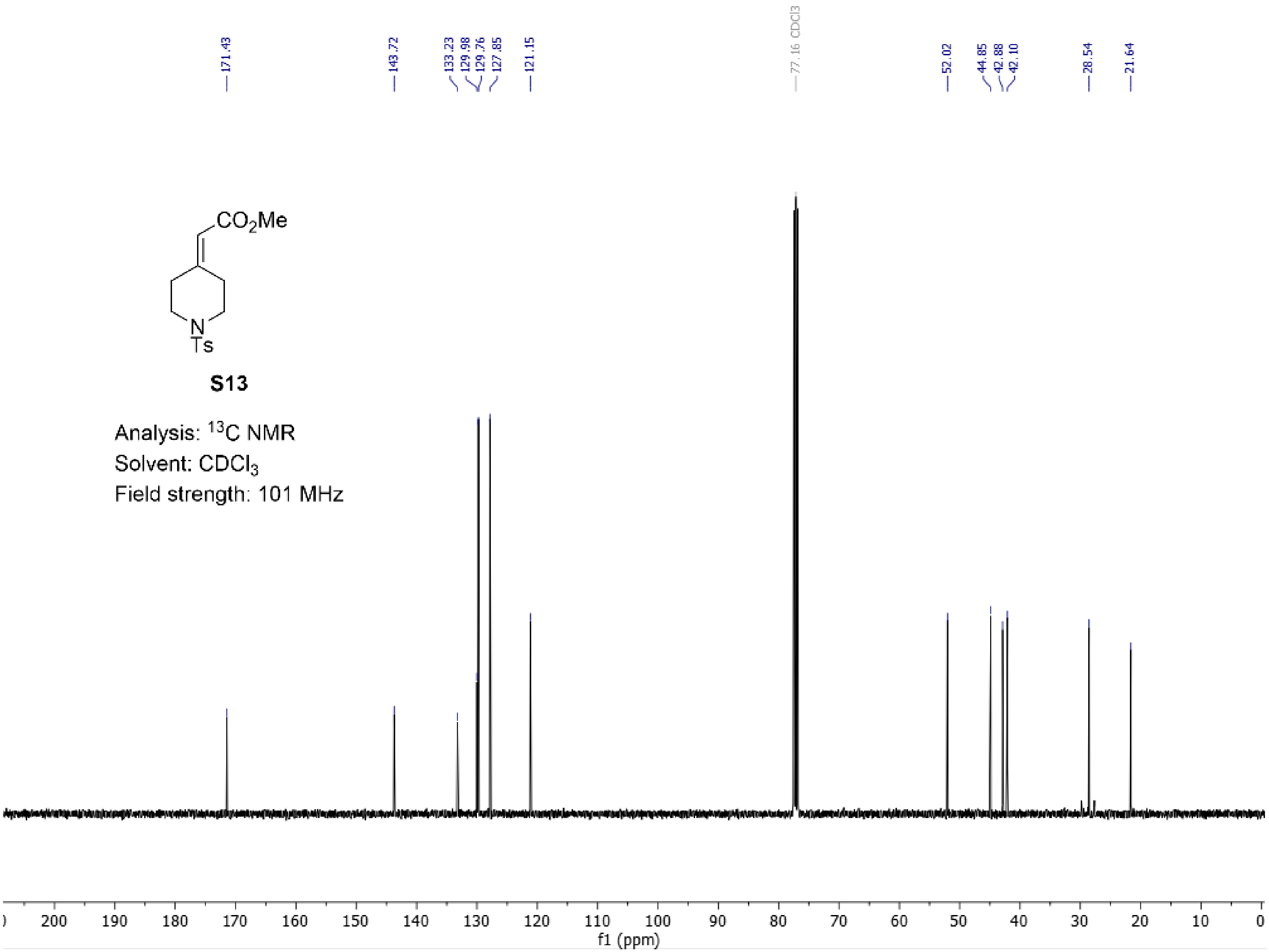
CO2Me
N
Ts
S13
Analysis: 13C NMR
Solvent: CDCl3
Field strength: 101 MHz
171.43
143.72
133.23
129.98
129.76
127.85
121.15
77.16 CDCl3
52.02
44.85
42.88
42.10
28.54
21.64
200 190 180 170 160 150 140 130 120 110 100 90 80 70 60 50 40 30 20 10 0
f1 (ppm)

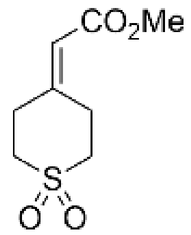

S14

Analysis: ^{1}H NMR
Solvent: $CDCl_3$
Field strength: 400 MHz

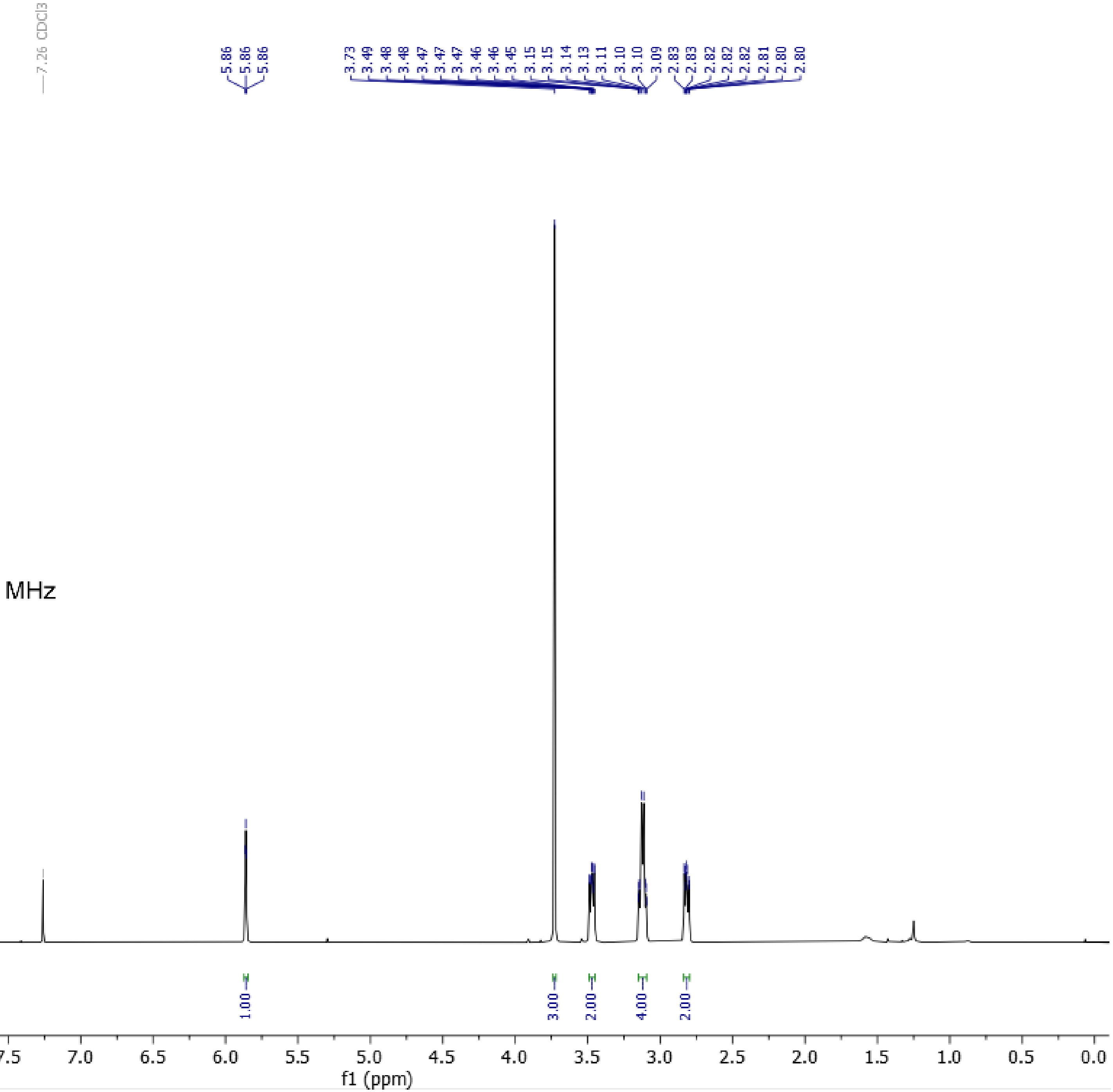

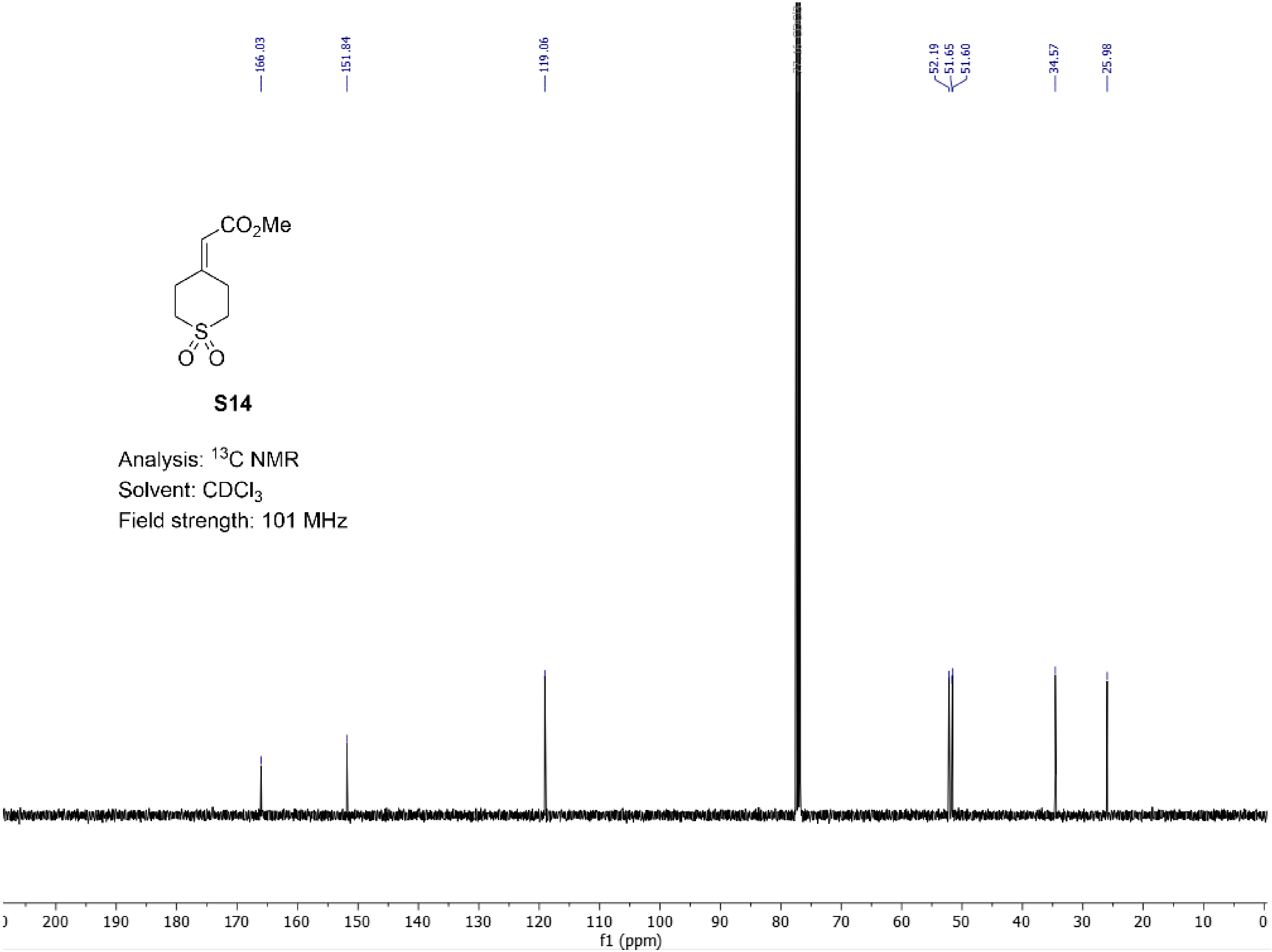
166.03
151.84
119.06
52.19
51.65
51.60
34.57
25.98
CO2Me
S
O
O
S14
Analysis: 13C NMR
Solvent: CDCl3
Field strength: 101 MHz
200
190
180
170
160
150
140
130
120
110
100
90
80
70
60
50
40
30
20
10
0
f1 (ppm)

4) X-Ray crystallographic data

X-Ray crystallographic data of **2q** (CCDC 2391108): **VG-4-103**.

Solved by: **Rosario Scopelliti**

Report created with ReportPlus

R_1=7.61%

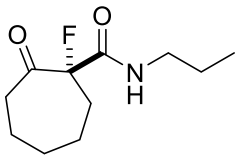

2q

Crystal Data and Experimental

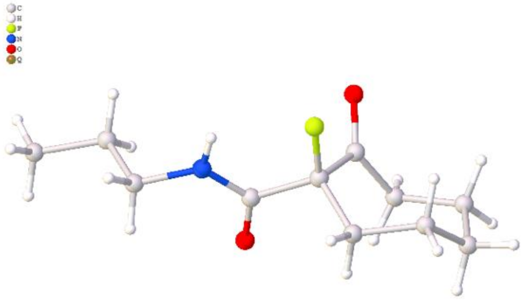

*X-Ray structure of **2p** (50% ellipsoid probability level)*

Experimental. Single clear pale colourless plate-shaped crystals of **vg-4-103** were used as supplied. A suitable crystal with dimensions 0.16 × 0.06 × 0.03 mm^3 was selected and mounted on a XtaLAB Synergy R, DW system, HyPix-Arc 150 diffractometer. The crystal was kept at a steady T = 139.99(10) K during data collection. The structure was solved with the ShelXT (Sheldrick, 2015) solution program using dual methods and by using Olex2 1.5 (Dolomanov et al., 2009) as the graphical interface. The model was refined with ShelXL 2019/3 (Sheldrick, 2015) using full matrix least squares minimisation on $\boldsymbol{F^2}$.

Crystal Data. $C_{11}H_{18}FNO_2$, M_r = 215.26, monoclinic, $P2_1/c$ (No. 14), a = 9.5318(7) Å, b = 9.6029(4) Å, c = 13.0101(6) Å, β = 98.680(5)°, $\alpha = \gamma$ = 90°, V = 1177.21(11) Å^3, T = 139.99(10) K, Z = 4, Z' = 1, μ(Cu K$_\alpha$) = 0.775, 13050 reflections measured, 2378 unique (R_{int} = 0.0459) which were used in all calculations. The final wR_2 was 0.2453 (all data) and R_1 was 0.0761 (I≥2 σ(I)).

**Compound**	**vg-4-103**
Formula	$C_{11}H_{18}FNO_2$
$D_{calc.}$/ g cm^{-3}	1.215
μ/mm^{-1}	0.775
Formula Weight	215.26
Colour	clear pale colourless
Shape	plate
Size/mm^3	0.16×0.06×0.03
T/K	139.99(10)
Crystal System	monoclinic
Space Group	$P2_1/c$
a/Å	9.5318(7)
b/Å	9.6029(4)
c/Å	13.0101(6)
α/°	90
β/°	98.680(5)
γ/°	90
V/Å^3	1177.21(11)
Z	4
Z'	1
Wavelength/Å	1.54184
Radiation type	Cu K$_\alpha$
Θ_{min}/°	4.693
Θ_{max}/°	75.356
Measured Refl's.	13050
Indep't Refl's	2378
Refl's I≥2 σ(I)	1346
R_{int}	0.0459
Parameters	141
Restraints	0
Largest Peak	0.520
Deepest Hole	-0.272
GooF	1.047
wR_2 (all data)	0.2453
wR_2	0.2067
R_1 (all data)	0.1371
R_1	0.0761

Structure Quality Indicators

**Reflections:**	d min (CuKα) 2Θ=150.7° 0.80	I/σ(I) 19.4	Rint m=5.77 4.59%	Full 135.4° 98% to 150.7° 99.9
**Refinement:**	Shift 0.000	Max Peak 0.5	Min Peak -0.3	GooF 1.047

A clear pale colourless plate-shaped crystal with dimensions 0.16 × 0.06 × 0.03 mm^3 was mounted. Data were collected using a XtaLAB Synergy R, DW system, HyPix-Arc 150 diffractometer operating at T = 139.99(10) K.

Data were measured using ω scans with Cu K_α radiation. The diffraction pattern was indexed and the total number of runs and images was based on the strategy calculation from the program CrysAlisPro system (CCD 43.130a 64-bit (release 05-07-2024)). The maximum resolution that was achieved was Θ = 75.356° (0.80 Å).

The unit cell was refined using CrysAlisPro 1.171.43.131a (Rigaku OD, 2024) on 3310 reflections, 25% of the observed reflections.

Data reduction, scaling and absorption corrections were performed using CrysAlisPro 1.171.43.131a (Rigaku OD, 2024). The final completeness is 99.90 % out to 75.356° in Θ. An analytical absorption correction was performed using CrysAlisPro 1.171.43.131a (Rigaku Oxford Diffraction, 2024). The analytical numeric absorption correction was done using a multifaceted crystal model based on expressions derived by R.C. Clark & J.S. Reid. (Clark, R. C. & Reid, J. S. (1995). Acta Cryst. A51, 887-897). The empirical absorption correction was carried out using spherical harmonics, implemented in SCALE3 ABSPACK scaling algorithm. The absorption coefficient μ of this crystal is 0.775 mm^{-1} at this wavelength (λ = 1.54184Å) and the minimum and maximum transmissions are 0.922 and 0.981.

The structure was solved and the space group $P2_1/c$ (# 14) determined by the ShelXT (Sheldrick, 2015) structure solution program using dual methods and refined by full matrix least squares minimisation on $\boldsymbol{F^2}$ using version 2019/3 of ShelXL (Sheldrick, 2015). All non-hydrogen atoms were refined anisotropically. Most hydrogen atom positions were calculated geometrically and refined using the riding model, but some hydrogen atoms were refined freely.

There is a single formula unit in the asymmetric unit, which is represented by the reported sum formula. In other words: Z is 4 and Z' is 1. The moiety formula is C11 H18 F N O2.

Data Plots: Diffraction Data

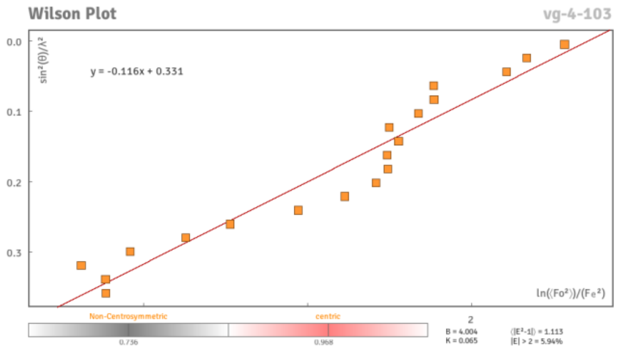

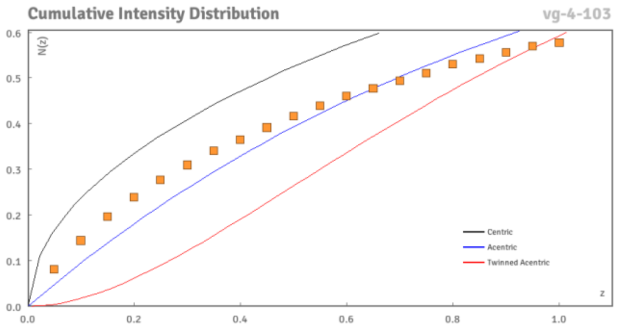

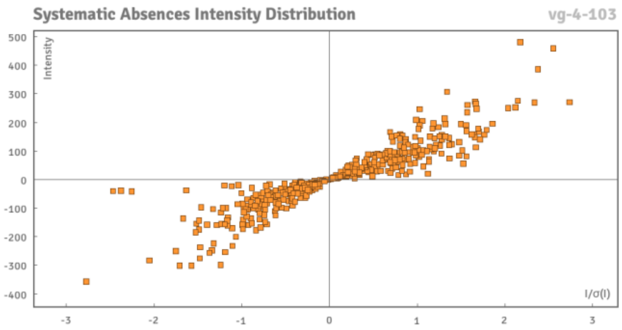

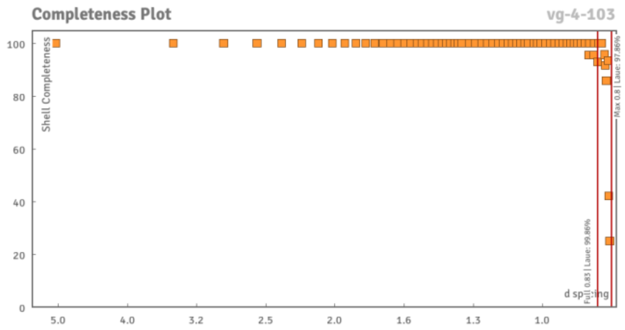

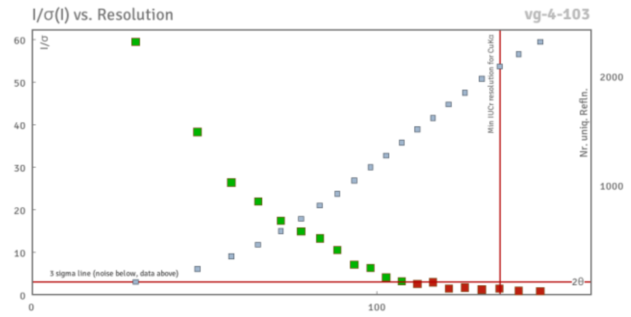

Data Plots: Refinement and Data

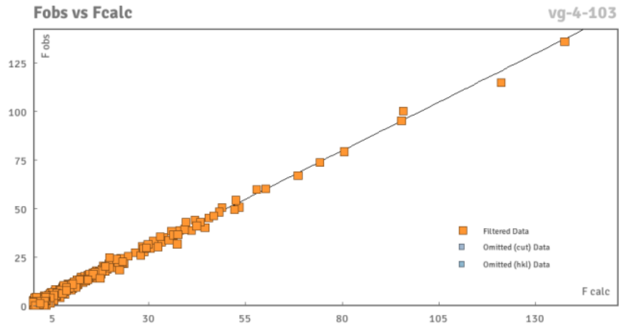

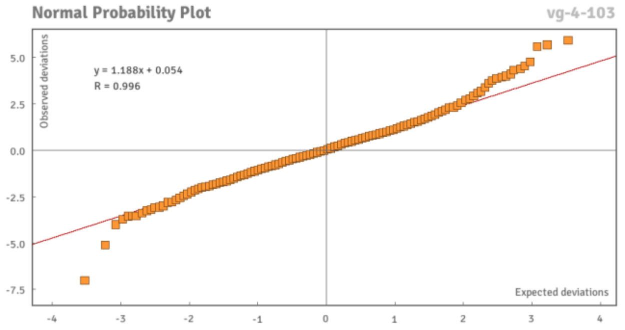

Reflection Statistics

Total reflections (after filtering)	13719	Unique reflections	2378
Completeness	0.979	Mean I/σ	12.03
hkl_{max} collected	(11, 11, 15)	hkl_{min} collected	(-11, -11, -13)
hkl_{max} used	(11, 11, 15)	hkl_{min} used	(-11, 0, 0)
Lim d_{max} collected	100.0	Lim d_{min} collected	0.77
d_{max} used	12.86	d_{min} used	0.8
Friedel pairs	1699	Friedel pairs merged	1
Inconsistent equivalents	0	R_{int}	0.0459
R_{sigma}	0.0516	Intensity transformed	0
Omitted reflections	0	Omitted by user (OMIT hkl)	0
Multiplicity	(2924, 1319, 806, 367, 201, 129, 85, 58, 44, 38, 20, 14, 12, 7, 1)	Maximum multiplicity	26
Removed systematic absences	669	Filtered off (Shel/OMIT)	0

Table S1: Fractional Atomic Coordinates (×10⁴) and Equivalent Isotropic Displacement Parameters (Å²×10³) for **vg-4-103**. U_{eq} is defined as 1/3 of the trace of the orthogonalised U_{ij}.

Atom	x	y	z	U_{eq}
F1	6562(3)	4437.9(17)	6417.9(18)	72.7(8)
O1	5785(3)	7757(2)	7332(2)	61.4(8)
O2	8717(4)	5111(4)	7754(3)	100.3(12)
N1	4673(3)	5651(3)	7316(2)	51.8(9)
C1	6818(4)	5878(3)	6533(3)	49.3(10)
C2	8269(4)	6051(4)	7175(3)	54.8(10)
C3	9125(4)	7302(4)	7027(3)	59.0(10)
C4	10002(4)	7083(4)	6140(3)	62.5(11)
C5	9147(4)	7137(4)	5061(3)	57.3(10)
C6	7898(4)	6160(4)	4843(3)	59.7(11)
C7	6686(4)	6491(4)	5444(3)	54.4(10)
C8	5694(4)	6497(3)	7110(3)	49.2(9)
C9	3517(4)	6097(3)	7847(3)	52.1(10)
C10	3866(4)	5896(4)	8994(3)	57.7(10)
C11	2623(5)	6290(4)	9560(4)	80.9(15)

Table S2: Anisotropic Displacement Parameters (×10⁴) for **vg-4-103.** The anisotropic displacement factor exponent takes the form: $-2\pi^2[h^2a^{*2} \times U_{11}+ ... +2hka^* \times b^* \times U_{12}]$

Atom	U_{11}	U_{22}	U_{33}	U_{23}	U_{13}	U_{12}
F1	113.3(19)	10.8(9)	106.9(18)	-7.1(9)	58.6(15)	-2.8(10)
O1	86.4(19)	9.1(10)	98.2(19)	-3.0(11)	44.8(15)	1.3(11)
O2	125(3)	82(2)	93(2)	44.3(19)	11(2)	27(2)
N1	74(2)	13.6(13)	74(2)	-2.0(12)	33.4(17)	-2.9(13)
C1	84(3)	6.7(13)	63(2)	-0.3(13)	30(2)	-0.7(15)
C2	83(3)	41(2)	43.1(18)	11.5(16)	18.9(18)	17.9(19)
C3	77(3)	55(2)	44.9(19)	-8.0(18)	10.7(18)	-1(2)
C4	73(3)	62(3)	56(2)	-9.7(19)	19.3(19)	-2(2)
C5	82(3)	45(2)	49(2)	2.9(16)	24.5(19)	-3(2)
C6	92(3)	43(2)	44.8(19)	-5.0(16)	10.8(19)	-2(2)
C7	76(3)	35.8(19)	50.0(19)	-2.6(16)	4.8(17)	-3.7(18)
C8	75(2)	15.9(15)	62(2)	3.5(14)	26.2(18)	3.4(16)
C9	66(2)	23.5(16)	67(2)	1.4(15)	12.0(18)	-3.6(16)
C10	85(3)	27.3(17)	61(2)	-6.7(16)	12(2)	1.0(18)
C11	123(4)	40(2)	95(3)	3(2)	63(3)	8(2)

Table S3: Bond Lengths in Å for **vg-4-103**.

Atom	Atom	Length/Å
F1	C1	1.408(3)
O1	C8	1.244(4)
O2	C2	1.212(4)
N1	C8	1.326(4)
N1	C9	1.451(4)
C1	C2	1.512(6)
C1	C7	1.522(5)
C1	C8	1.519(5)

Atom	Atom	Length/Å
C2	C3	1.481(5)
C3	C4	1.538(5)
C4	C5	1.514(5)
C5	C6	1.508(5)
C6	C7	1.523(5)
C9	C10	1.491(5)
C10	C11	1.534(5)

Table S4: Bond Angles in ° for **vg-4-103**.

Atom	Atom	Atom	Angle/°
C8	N1	C9	123.3(3)
F1	C1	C2	107.2(3)
F1	C1	C7	107.0(3)
F1	C1	C8	108.2(3)
C2	C1	C7	114.3(3)
C2	C1	C8	109.8(3)
C8	C1	C7	110.1(3)
O2	C2	C1	117.9(4)
O2	C2	C3	122.4(4)
C3	C2	C1	119.6(3)

Atom	Atom	Atom	Angle/°
C2	C3	C4	111.0(3)
C5	C4	C3	114.4(3)
C6	C5	C4	116.9(3)
C5	C6	C7	114.3(3)
C1	C7	C6	115.8(3)
O1	C8	N1	125.2(3)
O1	C8	C1	117.7(3)
N1	C8	C1	117.0(3)
N1	C9	C10	111.6(3)
C9	C10	C11	112.1(4)

Table S5: Torsion Angles in ° for **vg-4-103**.

Atom	Atom	Atom	Atom	Angle/°
F1	C1	C2	O2	-25.1(4)
F1	C1	C2	C3	151.0(3)
F1	C1	C7	C6	-72.9(4)
F1	C1	C8	O1	176.6(3)
F1	C1	C8	N1	-4.9(4)
O2	C2	C3	C4	89.4(4)
N1	C9	C10	C11	-176.9(3)
C1	C2	C3	C4	-86.4(4)
C2	C1	C7	C6	45.6(4)
C2	C1	C8	O1	59.8(4)
C2	C1	C8	N1	-121.7(3)
C2	C3	C4	C5	74.3(4)
C3	C4	C5	C6	-55.2(5)
C4	C5	C6	C7	67.5(4)
C5	C6	C7	C1	-85.4(4)
C7	C1	C2	O2	-143.4(3)
C7	C1	C2	C3	32.6(4)
C7	C1	C8	O1	-66.8(4)
C7	C1	C8	N1	111.7(3)
C8	N1	C9	C10	-91.4(4)
C8	C1	C2	O2	92.3(4)
C8	C1	C2	C3	-91.6(3)
C8	C1	C7	C6	169.7(3)
C9	N1	C8	O1	-0.7(6)
C9	N1	C8	C1	-179.1(3)

Table S6: Hydrogen Fractional Atomic Coordinates (×10[4]) and Equivalent Isotropic Displacement Parameters (Å[2]×10[3]) for **vg-4-103.** U_{eq} is defined as 1/3 of the trace of the orthogonalised U_{ij}.

Atom	x	y	z	U_{eq}
H3A	9770.81	7506.72	7679.2	71
H3B	8487.94	8112.52	6864.03	71
H4A	10749.49	7806.21	6191.32	75
H4B	10480.05	6166.58	6234.96	75
H5A	8793.69	8100.42	4932.5	69
H5B	9796.16	6940.03	4551.9	69
H6A	8231.04	5199.84	5014.78	72
H6B	7531.24	6188.57	4089.53	72
H7A	6608.45	7515.96	5498.05	65
H7B	5791.29	6150.15	5035.79	65

Atom	x	y	z	U_{eq}
H9A	2652.72	5557.62	7581.34	62
H9B	3313.71	7092.94	7696.75	62
H10A	4700.44	6474.16	9264.16	69
H10B	4122.03	4908.81	9141.25	69
H11A	1773.27	5773.2	9256.24	121
H11B	2439.89	7291.49	9485.1	121
H11C	2859.89	6055.49	10298.51	121
H1	4760(40)	4810(40)	7200(30)	52(10)

Table S7: Hydrogen Bond information for **vg-4-103**.

D	H	A	d(D-H)/Å	d(H-A)/Å	d(D-A)/Å	D-H-A/deg
C9	H9B	F1^{1}	0.99	2.52	3.352(4)	141.0
N1	H1	F1	0.83(4)	2.15(4)	2.569(4)	111(3)
N1	H1	O1^{2}	0.83(4)	2.15(4)	2.860(3)	144(3)

––––

^{1}1-x,1/2+y,3/2-z; ^{2}1-x,-1/2+y,3/2-z

Citations

CrysAlisPro Software System, Rigaku Oxford Diffraction, (2024).

O.V. Dolomanov and L.J. Bourhis and R.J. Gildea and J.A.K. Howard and H. Puschmann, Olex2: A complete structure solution, refinement and analysis program, *J. Appl. Cryst.*, (2009), **42**, 339-341.

Sheldrick, G.M., Crystal structure refinement with ShelXL, *Acta Cryst.*, (2015), **C71**, 3-8.

Sheldrick, G.M., ShelXT-Integrated space-group and crystal-structure determination, *Acta Cryst.*, (2015), **A71**, 3-8.

X-Ray crystallographic data of **2f-2** (CCDC 2391722): **VG-4-062-2**.

Solved by: **Farzaneh Fadaei Tirani**

Report created with ReportPlus

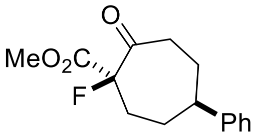

2f-2

R_1=7.59%

Crystal Data and Experimental

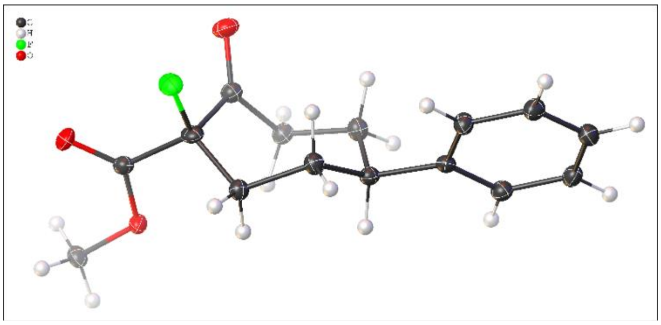

*X-Ray structure of **2f-2** (50% ellipsoid probability level)*

Experimental. Single colourless needle-shaped crystals of **vg-4-062-2** were used as supplied. A suitable crystal with dimensions 0.21 × 0.02 × 0.01 mm³ was selected and mounted on an XtaLAB Synergy R, DW system, HyPix-Arc 150 diffractometer. The crystal was kept at a steady T = 139.99(10) K during data collection. The structure was solved with the ShelXT 2018/2 (Sheldrick, 2015) solution program using dual methods and by using Olex2 1.5 (Dolomanov et al., 2009) as the graphical interface. The model was refined with ShelXL 2019/3 (Sheldrick, 2015) using full matrix least squares minimisation on F^2.

Crystal Data. $C_{15}H_{17}O_3F$, M_r = 264.28, monoclinic, $P2_1/n$ (No. 14), a = 8.9197(5) Å, b = 5.6777(3) Å, c = 25.3460(18) Å, β = 91.248(6)°, α = γ = 90°, V = 1283.31(14) Å³, T = 139.99(10) K, Z = 4, Z' = 1, μ(Cu K_α) = 0.863, 8519 reflections measured, 2496 unique (R_{int} = 0.0421) which were used in all calculations. The final wR_2 was 0.2440 (all data) and R_1 was 0.0759 (I≥2σ(I)).

**Compound**	**VG-4-062-2**
Formula	$C_{15}H_{17}O_3F$
$D_{calc.}$/ g cm^{-3}	1.368
μ/mm^{-1}	0.863
Formula Weight	264.28
Colour	colourless
Shape	needle-shaped
Size/mm³	0.21×0.02×0.01
T/K	139.99(10)
Crystal System	monoclinic
Space Group	$P2_1/n$
a/Å	8.9197(5)
b/Å	5.6777(3)
c/Å	25.3460(18)
α/°	90
β/°	91.248(6)
γ/°	90
V/Å³	1283.31(14)
Z	4
Z'	1
Wavelength/Å	1.54184
Radiation type	Cu$K\alpha$
Θ_{min}/°	3.488
Θ_{max}/°	74.549
Measured Refl's.	8519
Indep't Refl's	2496
Refl's I≥2σ(I)	1710
R_{int}	0.0421
Parameters	174
Restraints	0
Largest Peak/e Å^{-3}	0.477
Deepest Hole/e Å^{-3}	-0.343
GooF	1.066
wR_2 (all data)	0.2440
wR_2	0.2263
R_1 (all data)	0.1072
R_1	0.0759
CCDC number	2391722

Structure Quality Indicators

**Reflections:**	d min (CuKα) 2Θ=149.1° 0.80	I/σ(I) 18.5	Rint m=3.69 4.21%	Full 135.4° 96% to 149.1° 99.5
**Refinement:**	Shift 0.000	Max Peak 0.5	Min Peak -0.3	GooF 1.066

A colourless needle-shaped crystal with dimensions 0.21 × 0.02 × 0.01 mm^3 was mounted. Data were collected using an XtaLAB Synergy R, DW system, HyPix-Arc 150 diffractometer operating at T = 139.99(10) K.

Data were measured using ω scans with CuK$_\alpha$ radiation. The diffraction pattern was indexed and the total number of runs and images was based on the strategy calculation from the program CrysAlisPro system (CCD 43.137a 64-bit (release 10-09-2024)). The maximum resolution achieved was Θ = 74.549° (0.80 Å).

The unit cell was refined using CrysAlisPro 1.171.43.135a (Rigaku OD, 2024) on 2504 reflections, 29% of the observed reflections.

Data reduction, scaling and absorption corrections were performed using CrysAlisPro 1.171.43.135a (Rigaku OD, 2024). The final completeness is 99.50 % out to 74.549° in Θ. A Gaussian absorption correction was performed using CrysAlisPro 1.171.43.135a (Rigaku Oxford Diffraction, 2024) Numerical absorption correction based on Gaussian integration over a multifaceted crystal model. Empirical absorption correction using spherical harmonics as implemented in SCALE3 ABSPACK scaling algorithm. The absorption coefficient μ of this material is 0.863 mm^{-1} at this wavelength (λ = 1.54184Å) and the minimum and maximum transmissions are 0.801 and 1.000.

The structure was solved in the space group $P2_1/n$ (# 14) by the ShelXT 2018/2 (Sheldrick, 2015) structure solution program using dual methods and refined by full matrix least squares minimisation on $\boldsymbol{F}^2$ using version 2019/3 of ShelXL 2019/3 (Sheldrick, 2015). All non-hydrogen atoms were refined anisotropically. Hydrogen atom positions were calculated geometrically and refined using the riding model.

There is a single formula unit in the asymmetric unit, which is represented by the reported sum formula. In other words: Z is 4 and Z' is 1. The moiety formula is C15 H17 F O3.

Citations

CrysAlis^{Pro} Software System, Rigaku Oxford Diffraction, (2024).

Sheldrick, G.M., ShelXT-Integrated space-group and crystal-structure determination, *Acta Cryst.*, (2015), **A71**, 3-8.

Sheldrick, G.M., Crystal structure refinement with ShelXL, *Acta Cryst.*, (2015), **C71**, 3-8.

O.V. Dolomanov and L.J. Bourhis and R.J. Gildea and J.A.K. Howard and H. Puschmann, **Olex2**: A complete structure solution, refinement and analysis program, *J. Appl. Cryst.*, (2009), **42**, 339-341.

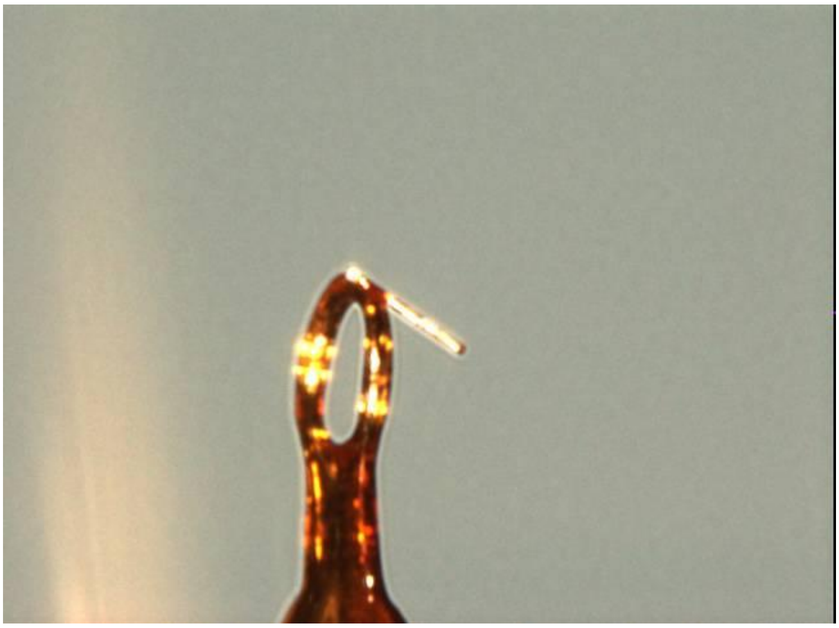

Figure S1 Image of the Crystal on the Diffractometer.

Data Plots: Diffraction Data

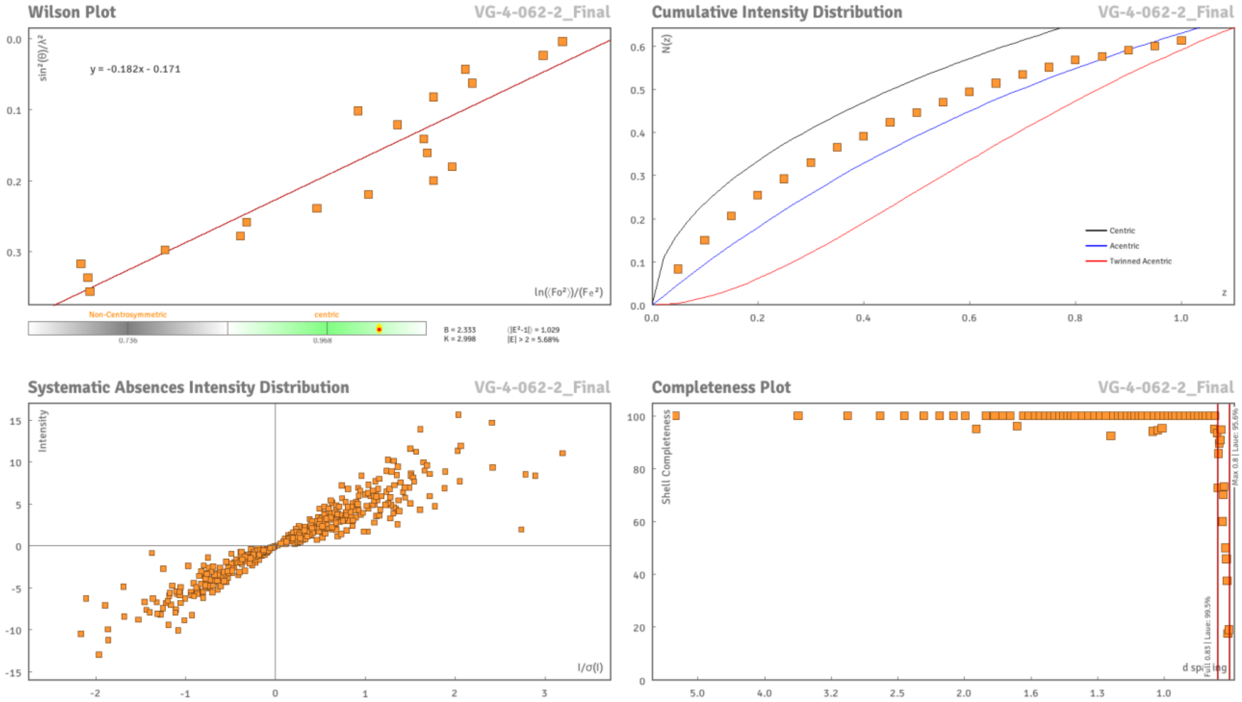

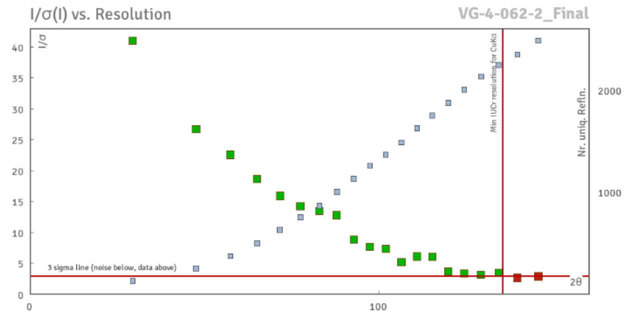

Data Plots: Refinement and Data

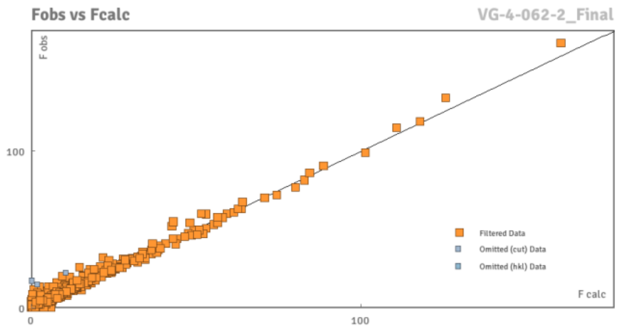

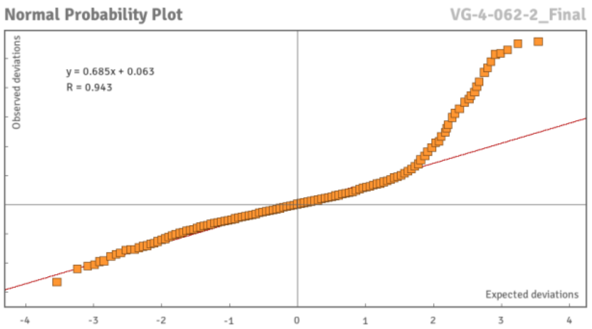

Reflection Statistics

Total reflections (after filtering)	9198	Unique reflections	2496
Completeness	0.956	Mean I/σ	11.67
hkl_{max} collected	(10, 7, 30)	hkl_{min} collected	(-9, -6, -30)
hkl_{max} used	(10, 7, 30)	hkl_{min} used	(-10, 0, 0)
Lim d_{max} collected	100.0	Lim d_{min} collected	0.77
d_{max} used	25.34	d_{min} used	0.8
Friedel pairs	1210	Friedel pairs merged	1
Inconsistent equivalents	1	R_{int}	0.0421
R_{sigma}	0.054	Intensity transformed	0
Omitted reflections	19	Omitted by user (OMIT hkl)	0
Multiplicity	(3300, 1504, 418, 148, 82, 39, 25, 8, 8, 4, 4, 2)	Maximum multiplicity	15
Removed systematic absences	679	Filtered off (Shel/OMIT)	0

Table S8: Fractional Atomic Coordinates (×10^4) and Equivalent Isotropic Displacement Parameters (Å^2×10^3) for **vg-4-062-2.** U_{eq} is defined as 1/3 of the trace of the orthogonalised U_{ij}.

Atom	x	y	z	U_{eq}
F1	7767(3)	6831(4)	4233.2(11)	36.2(7)
O1	4961(4)	6170(6)	4409.1(14)	38.7(9)
O2	8471(4)	5695(5)	5220.2(13)	35.8(8)
O3	8072(3)	1841(5)	5052.9(11)	28.1(7)
C1	7410(5)	4514(7)	4372.6(19)	28.4(10)
C2	5694(5)	4366(8)	4393.4(18)	28.4(10)
C3	5007(5)	1975(8)	4331.6(17)	27.9(10)
C4	4621(5)	1564(8)	3741.8(17)	30.6(10)
C5	5962(5)	1023(7)	3390.4(17)	24.4(9)
C6	7226(5)	2856(8)	3428.0(17)	28.8(10)

Atom	x	y	z	U_{eq}
C7	8066(5)	2865(8)	3964.0(16)	25.8(9)
C8	8057(5)	4126(7)	4933.5(18)	26.7(10)
C9	8640(5)	1292(8)	5572.2(18)	30.8(10)
C10	5344(5)	676(7)	2830.4(17)	25.6(9)
C11	5505(5)	2336(8)	2433.3(17)	30.1(10)
C12	4815(5)	2023(8)	1943.6(18)	32.5(10)
C13	3951(5)	61(8)	1836.8(19)	32.1(10)
C14	3788(5)	-1631(8)	2225.9(18)	30.5(10)
C15	4484(5)	-1331(8)	2715.5(18)	29.7(10)

Table S9: Anisotropic Displacement Parameters (×10[4]) for **vg-4-062-2.** The anisotropic displacement factor exponent takes the form: $-2\pi^2[h^2a^{*2} \times U_{11} + ... + 2hka^* \times b^* \times U_{12}]$

Atom	U_{11}	U_{22}	U_{33}	U_{23}	U_{13}	U_{12}
F1	38.5(15)	21.6(13)	48.1(17)	7.7(12)	-6.9(12)	-6.6(11)
O1	40.1(19)	24.6(17)	52(2)	-4.3(15)	2.9(15)	6.4(14)
O2	43.0(19)	24.3(17)	39.7(19)	-11.2(14)	-8.8(15)	-2.1(14)
O3	35.9(17)	21.6(15)	26.4(16)	-0.9(12)	-7.6(12)	0.2(13)
C1	32(2)	14.6(19)	39(3)	4.2(18)	-1.8(19)	-1.2(17)
C2	30(2)	25(2)	30(2)	-0.5(18)	0.0(18)	0.6(19)
C3	30(2)	24(2)	30(2)	-1.2(18)	2.0(18)	-1.9(18)
C4	29(2)	34(2)	29(2)	0.9(19)	-0.5(18)	-1.7(19)
C5	26(2)	21(2)	26(2)	0.6(17)	0.3(16)	1.5(17)
C6	29(2)	31(2)	27(2)	1.8(18)	-0.9(17)	-1.6(18)
C7	27(2)	26(2)	24(2)	1.1(18)	0.6(16)	-1.4(18)
C8	25(2)	23(2)	31(2)	-0.6(18)	-1.4(17)	1.9(17)
C9	33(2)	29(2)	30(2)	5.0(19)	-8.3(18)	-2.7(19)
C10	25(2)	24(2)	27(2)	-1.1(18)	-2.0(16)	4.8(17)
C11	35(2)	25(2)	30(2)	1.4(18)	-4.2(18)	-3.9(19)
C12	41(3)	29(2)	27(2)	-0.5(19)	-1.1(19)	1(2)
C13	33(2)	34(2)	30(2)	-1(2)	-4.0(18)	3(2)
C14	29(2)	27(2)	35(2)	-6.4(19)	-1.6(18)	-1.9(18)
C15	31(2)	24(2)	34(3)	0.9(19)	-0.8(18)	-0.5(18)

Table S10: Bond Lengths in Å for **vg-4-062-2**.

Atom	Atom	Length/Å
F1	C1	1.401(5)
O1	C2	1.217(5)
O2	C8	1.203(5)
O3	C8	1.333(5)
O3	C9	1.434(5)
C1	C2	1.535(6)
C1	C7	1.523(6)
C1	C8	1.538(6)
C2	C3	1.496(6)
C3	C4	1.544(6)

Atom	Atom	Length/Å
C4	C5	1.538(6)
C5	C6	1.536(6)
C5	C10	1.524(6)
C6	C7	1.537(6)
C10	C11	1.388(6)
C10	C15	1.401(6)
C11	C12	1.385(6)
C12	C13	1.377(6)
C13	C14	1.387(7)
C14	C15	1.386(6)

Table S11: Bond Angles in ° for **vg-4-062-2**.

Atom	Atom	Atom	Angle/°
C8	O3	C9	114.9(3)
F1	C1	C2	107.0(3)
F1	C1	C7	108.3(4)
F1	C1	C8	106.6(3)
C2	C1	C8	108.3(4)
C7	C1	C2	112.8(4)
C7	C1	C8	113.5(3)
O1	C2	C1	119.5(4)
O1	C2	C3	123.2(4)
C3	C2	C1	116.9(4)
C2	C3	C4	108.7(4)
C5	C4	C3	115.5(4)
C6	C5	C4	114.1(4)
C10	C5	C4	107.2(3)
C10	C5	C6	113.3(4)
C5	C6	C7	113.6(4)
C1	C7	C6	114.6(4)
O2	C8	O3	125.6(4)
O2	C8	C1	123.8(4)
O3	C8	C1	110.5(3)
C11	C10	C5	123.1(4)
C11	C10	C15	117.8(4)
C15	C10	C5	118.9(4)
C12	C11	C10	120.8(4)
C13	C12	C11	121.0(4)
C12	C13	C14	119.2(4)
C15	C14	C13	120.0(4)
C14	C15	C10	121.2(4)

Table S12: Torsion Angles in ° for **vg-4-062-2**.

Atom	Atom	Atom	Atom	Angle/°
F1	C1	C2	O1	-15.7(6)
F1	C1	C2	C3	157.6(4)
F1	C1	C7	C6	-73.8(4)
F1	C1	C8	O2	14.7(6)
F1	C1	C8	O3	-166.0(3)
O1	C2	C3	C4	80.9(5)
C1	C2	C3	C4	-92.1(5)
C2	C1	C7	C6	44.4(5)
C2	C1	C8	O2	-100.1(5)
C2	C1	C8	O3	79.2(4)
C2	C3	C4	C5	75.2(5)
C3	C4	C5	C6	-53.8(5)
C3	C4	C5	C10	179.9(4)
C4	C5	C6	C7	68.6(5)
C4	C5	C10	C11	105.8(5)
C4	C5	C10	C15	-69.8(5)
C5	C6	C7	C1	-90.0(4)
C5	C10	C11	C12	-174.5(4)
C5	C10	C15	C14	174.3(4)
C6	C5	C10	C11	-20.9(6)
C6	C5	C10	C15	163.5(4)
C7	C1	C2	O1	-134.7(4)
C7	C1	C2	C3	38.6(5)
C7	C1	C8	O2	133.8(4)
C7	C1	C8	O3	-46.9(5)
C8	C1	C2	O1	98.8(5)
C8	C1	C2	C3	-87.9(5)
C8	C1	C7	C6	168.2(4)
C9	O3	C8	O2	0.0(6)
C9	O3	C8	C1	-179.3(3)
C10	C5	C6	C7	-168.4(4)
C10	C11	C12	C13	0.0(7)
C11	C10	C15	C14	-1.4(6)
C11	C12	C13	C14	-0.6(7)
C12	C13	C14	C15	0.2(7)
C13	C14	C15	C10	0.8(7)
C15	C10	C11	C12	1.0(6)

Table S13: Hydrogen Fractional Atomic Coordinates (×10^4) and Equivalent Isotropic Displacement Parameters (Å^2×10^3) for **vg-4-062-2.** U_{eq} is defined as 1/3 of the trace of the orthogonalised U_{ij}.

Atom	x	y	z	U_{eq}
H3A	4086.46	1869.28	4541.52	34
H3B	5719.5	755.93	4460.23	34
H4A	3903.86	236.76	3713.39	37
H4B	4106.38	2984.22	3601.86	37
H5	6392.93	-518.14	3509.9	29
H6A	6794.31	4439.8	3363.73	35
H6B	7951.75	2540.44	3146.66	35
H7A	8068.27	1243.11	4107.67	31
H7B	9121.93	3319.47	3907.18	31
H9A	8655.66	-420.46	5620.31	46
H9B	7993.52	2007.69	5835.53	46
H9C	9660.53	1914.18	5615.45	46
H11	6095.98	3702.15	2498.24	36
H12	4938.26	3178.89	1677.12	39
H13	3474.14	-130.21	1500.8	39
H14	3199.03	-2994.75	2156.88	37
H15	4376.54	-2508.7	2978.17	36
